# Application of ^**19**^F NMR Spectroscopy for Determining the Absolute Configuration of α‑Chiral Amines and Secondary Alcohols Using Trifluoromethylbenzoimidazolylbenzoic Acid

**DOI:** 10.1021/acs.joc.5c00879

**Published:** 2025-07-09

**Authors:** David Profous, Michal Kriegelstein, Petr Jurečka, Petr Cankař

**Affiliations:** † Department of Organic Chemistry, Faculty of Science, 98735 Palacký University Olomouc, 17. listopadu 1192/12, Olomouc 779 00, Czech Republic; ‡ Department of Physical Chemistry, Faculty of Science, Palacký University Olomouc, 17. listopadu 1192/12, Olomouc 779 00, Czech Republic

## Abstract

Axially chiral 2-(2-(trifluoromethyl)-1*H*-benzo­[*d*]­imidazol-1-yl)­benzoic acid (TBBA) was employed as a chiral derivatizing agent for determining the absolute α-configuration of primary amines and secondary alcohols via ^19^F NMR spectroscopy. The method utilizes the trifluoromethyl group as a sensor, detecting the shielding effects induced by individual alcohol or amine substituents. This approach was tested on 46 alcohols and amines, demonstrating greater efficiency compared to the standard MTPA method.

## Graphical abstract

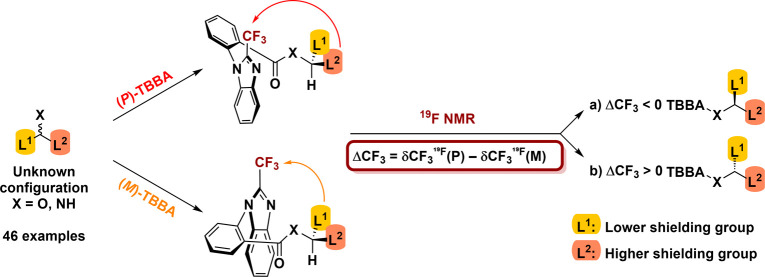

## Introduction

The application of NMR spectroscopy to determine the absolute configuration has been extensively studied since the 1970s, following the introduction of α-methoxy-α-trifluoromethylphenylacetic acid (**1**; MTPA) by Mosher and Dale in 1969 (Figure 1).^1,2^ The most commonly used methods involve transforming a chiral molecule into two distinct diastereomers using chiral derivatizing agents (CDAs)^3,4^ and comparing their ^1^H NMR,^5–8^ ^13^C NMR,^9^ or ^19^F NMR spectra.^10,11^ ^1^H NMR spectroscopy is the most frequently employed and is generally considered more reliable. For correct determination, it is crucial that both diastereomers exhibit a preference for a particular conformation (NMR-significant conformer), with the shielding group projecting its magnetic anisotropy in a directional and selective manner to one part of the chiral molecule. However, in some cases, anomalous conformational equilibria lead to incorrect configuration determinations, which can be further complicated by the commercial unavailability of certain CDAs.^12–14^

**Figure 1:**
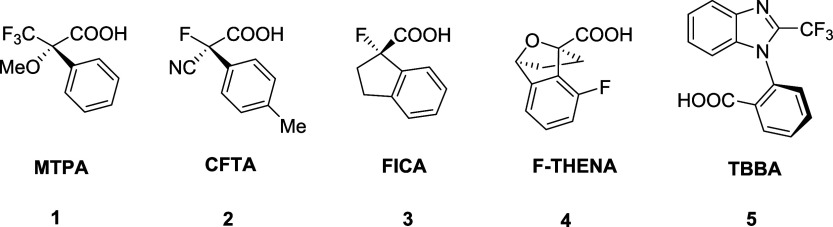
Structures of α-methoxy-α-trifluoromethylphenylacetic acid (**1**; MTPA),^10^ α-cyano-α-fluoro-*p*-tolylacetic acid (**2**; CFTA),^11,15^ 1-fluoroindan-1-carboxylic acid (**3**; FICA),^16^ 8-fluoro-1,2,3,4-tetrahydro-1,4-epoxynaphthalene-1-carboxylic acid (**4**; F-THENA),^17^ and trifluoromethylbenzimidazolylbenzoic acid (**5**; TBBA).^5^.

**Figure 2:**
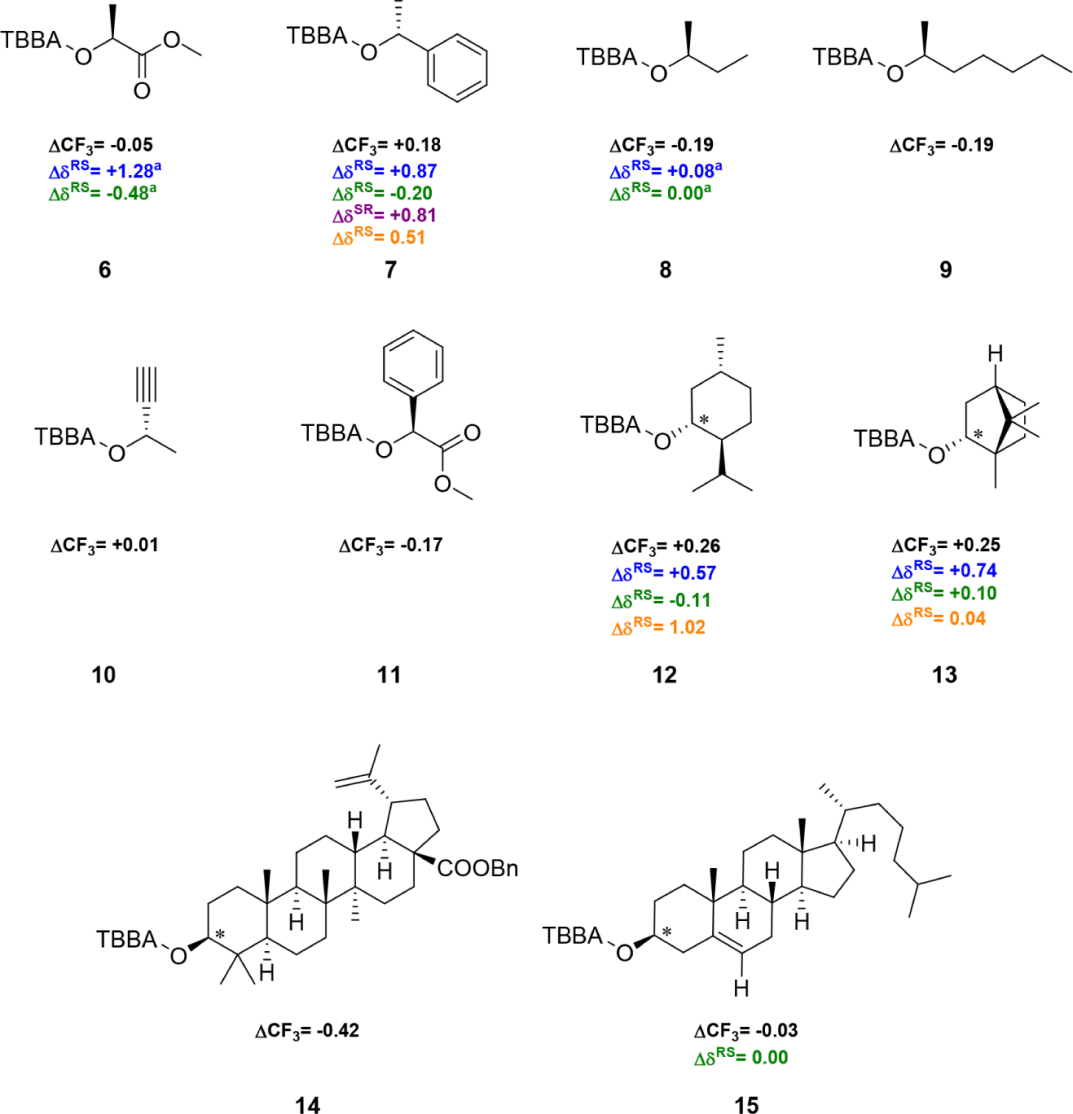
Differences in the ^19^F NMR chemical shifts for diastereoisomeric TBBA esters are represented as ΔCF_3_. The α-chiral position is marked with an asterisk in compounds that contain multiple stereocenters. The samples were analyzed in CDCl_3_ as the solvent, with CFCl_3_ as the internal standard. The calculation was performed using the equation: ΔCF_3_ = δCF_3_^19^F­(*P*) – δCF_3_^19^F­(*M*). The ΔCF_3_ differences are shown for TBBA (black), CFTA (blue), MTPA (green), F-THENA (purple), and FICA (orange). ^a^The analyzed alcohol possessed the opposite configuration.

The first MTPA-based methods were also developed to compare ^19^F NMR spectra, as these spectra are straightforward to interpret and the probability of signal overlap is low.^1,10^ However, these methods proved unreliable for determining absolute configuration, given that the assignment accuracy based on the conformational model was less than 50%.^12^ A new generation of CDAs has since been developed to effectively address most of these limitations. Nevertheless, it is important to note that absolute configuration is often assigned based on a single data point. These advanced CDAs (Figure 1) include α-cyano-α-fluoro-*p*-tolylacetic acid (**2**; CFTA),^11,15^ 1-fluoroindan-1- carboxylic acid (**3**; FICA),^16^ and 8-fluoro-1,2,3,4-tetrahydro-1,4-epoxynaphthalene-1-carboxylic acid (**4**; F-THENA).^17^ However, these CDAs were evaluated only preliminarily with a limited library of chiral compounds, and their structural variety was often low.

As the use of chiral ^19^F-labeled probes in ^19^F NMR spectroscopy offers high sensitivity and very low background noise in complex mixture analysis, this analytical technique is promising for the development of novel chiral chemosensors. Palladium complexes with trifluoromethyl groups were used for chirality sensing and detection of amines. Similarly, rhodium complexes can be used as well.^18–22^ Furthermore, the importance of ^19^F NMR spectroscopy is also highlighted by applications of fluorine labeling to study structures, dynamics, and small molecule interactions of molecular complexes and biological molecules.^23,24^

Recently, we developed an axially chiral CDA, trifluoromethylbenzimidazolylbenzoic acid (**5**; TBBA). Its application has been investigated for determining the absolute configuration by ^1^H NMR of primary amines, secondary alcohols,^5^ and β-chiral alcohols,^25^ including their limitations.^26^

In our initial studies, we were not able to propose a reliable general conformational model for the determination of absolute configuration using ^19^F NMR spectroscopy. However, we did not take into account the influence of magnetic anisotropy contributions from specific bonds^27^ within substituents L_1_ and L_2_ on the shielding of the trifluoromethyl group (Figure 6).

To test our hypothesis regarding the projection of the shielding effect of specific substituents on the trifluoromethyl group in TBBA, we conducted a study on an extended library of TBBA derivatives. Understanding the magnitude of shielding induced by these substituents should enable the assignment and prediction of absolute configuration according to the general conformational model disclosed in this report.

## Results and Discussion

TBBA derivatives were synthesized following a previously reported procedure.^5^ Initially, we compared chiral secondary alcohols using ^19^F NMR spectroscopy. l-Lactate **6** was derivatized into TBBA diastereomers, showing an ^19^F chemical shift difference (ΔCF_3_) of −0.05 ppm. This difference is notably smaller than that observed for the *D*-lactate derivatives of CFTA (Δδ^RS^ = +1.28 ppm)^11^ and MTPA derivatives (Δδ^RS^ = −0.48 ppm).^10^

In the case of (*R*)-1-phenylethan-1-ol **7**, the ΔCF_3_ increased to +0.18 ppm, which is lower than that of CFTA (Δδ^RS^ = +0.87 ppm),^11^ MTPA (Δδ^RS^ = −0.2 ppm),^10^ FICA (Δδ^RS^ = 0.51 ppm),^16^ and F-THENA (Δδ^SR^ = +0.81 ppm).^17^ However, when the phenyl substituent was replaced by an ethyl group in (*S*)-2-butanol **8**, the ΔCF_3_ increased to −0.19 ppm, which was higher than the values observed for its esters with CFTA (Δδ^RS^ = +0.08 ppm)^11^ and MTPA (Δδ^RS^ = 0 ppm).^10^

The chain length had no influence on the resulting ΔCF_3_, as (*S*)-2-heptyl (**9**) exhibited the same ΔCF_3_ as (*S*)-2-butanol **8**. The methyl group of alcohols **6**–**9** exhibited a weaker shielding effect compared to the ester group, phenyl group, and longer aliphatic chains. In contrast, (*S*)-but-3-yn-2-ol **10** showed a ΔCF_3_ of +0.01 ppm, indicating that the methyl group provides greater shielding than the acetylene group. These experimental observations were consistent with the calculated magnitudes of the magnetic anisotropy for the individual groups.^27^

Further, the ester group in derivative **11** was compared with the phenyl group. A ΔCF_3_ difference of −0.17 ppm demonstrated a stronger shielding by the ester group. ΔCF_3_ values of +0.26 ppm and +0.25 ppm were measured for menthol **12** and borneol **13**, respectively. As predicted, the more branched parts of both molecules produced stronger shielding of the CF_3_ group. TBBA esters of menthol **12** provided a smaller ΔCF_3_ than both CFTA (Δδ^RS^ = +0.57 ppm)^11^ and FICA (Δδ^RS^ = 1.02 ppm) esters,^16^ but a higher Δδ^RS^ than MTPA ester (Δδ^RS^ = −0.11 ppm).^10^ Similarly, TBBA esters of borneol **13** showed a lower ΔCF_3_ than CFTA (Δδ^RS^ = +0.74 ppm);^11^ however, in the case of FICA, the Δδ^RS^ was notably smaller (Δδ^RS^ = 0.04 ppm),^16^ and MTPA even reported the opposite enantiomer (Δδ^RS^ = +0.10 ppm).^12^

We also compared TBBA esters of more complex natural substances derived from benzyl-protected betulinic acid **14** and cholesterol **15**. The adjacent dimethyl group to the secondary alcohol in betulinic acid **14** showed significantly greater shielding (ΔCF_3_ of −0.42 ppm) compared to the methylene group. Remote branching did not lead to any difference in the chemical shift for MTPA esters of cholesterol,^12^ while TBBA esters **15** exhibited a ΔCF_3_ of −0.03 ppm, which is consistent with the proposed general conformational model. As expected, branching closer to the chiral center of the secondary alcohol resulted in stronger shielding of the CF_3_ group in TBBA derivatives.

With several TBBA esters of secondary alcohols in hand, we shifted our focus to amino acid derivatives (Figure 3). Our initial goal was to evaluate whether the *tert*-butyl substituent provides stronger shielding than the ester substituent. TBBA amides from methyl (*S*)-2-amino-3,3-dimethylbutanoate **16** were prepared, providing a ΔCF_3_ of −0.34 ppm, which indicated a considerably stronger shielding effect of the ester on the trifluoromethyl group. The ΔCF_3_ of TBBA amides was significantly smaller than that reported for CFTA (Δδ^RS^ = −4.074 ppm)^15^ but higher than that reported for MTPA (Δδ^RS^ = −0.171 ppm).^15^

**Figure 3:**
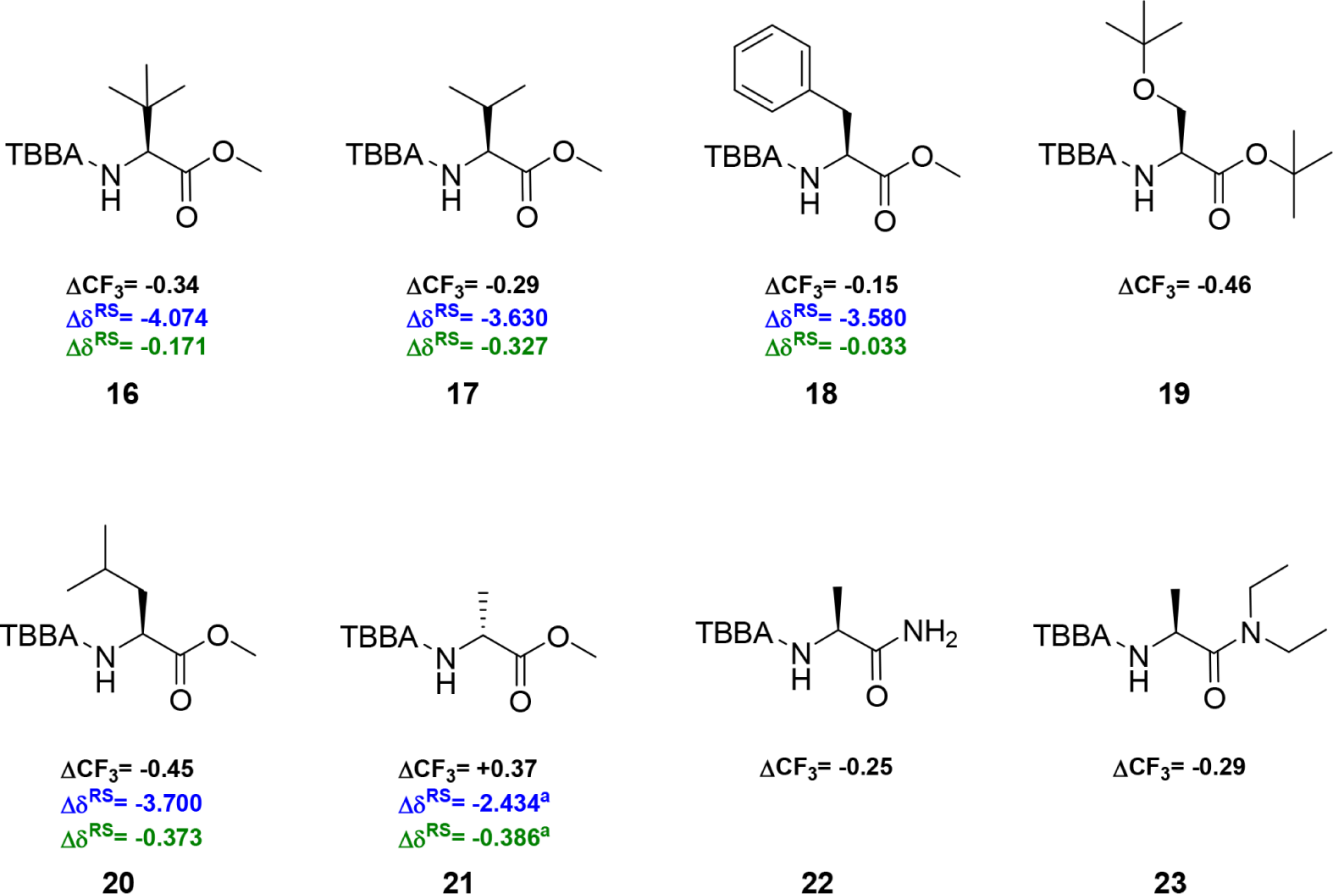
Differences in the ^19^F NMR chemical shifts for diastereoisomeric TBBA amides are represented as ΔCF_3_. The samples were analyzed in CDCl_3_ as the solvent, with CFCl_3_ as the internal standard. The calculation was performed using the equation: ΔCF_3_ = δCF_3_^19^F­(*P*) – δCF_3_^19^F­(*M*). The ΔCF_3_ differences are shown for TBBA (black), CFTA (blue), and MTPA (green). ^a^The analyzed amine possessed the opposite configuration.

Interestingly, replacing the *tert*-butyl group with isopropyl in l-Val-OMe **17** reduced the ΔCF_3_ to −0.29 ppm. A similar trend was observed for CFTA (Δδ^RS^ = −3.630 ppm),^15^ whereas for MTPA, the Δδ^RS^ increased to −0.327 ppm.^15^ A further decrease in ΔCF_3_ occurred in the case of l-Phe-OMe **18**, which showed a value of −0.15 ppm. Both related esters of CFTA and MTPA also showed a decrease in the Δδ^RS^ to −3.580 ppm for CFTA^15^ and −0.033 ppm for MTPA.^15^

l-Ser­(tBu)-OtBu **19** provided the highest ΔCF_3_ (−0.46 ppm) for the TBBA amides among the synthesized amino acid derivatives. There was a slight decrease in the ΔCF_3_ to −0.45 ppm for l-Leu-OMe **20**. The leucine amide with MTPA exhibited a lower Δδ^RS^ of −0.373 ppm,^15^ while CFTA showed a Δδ^RS^ of −3.700 ppm.^15^ The stronger shielding effect of the ester compared to the methyl group was also expected for the TBBA amide of *D*-Ala-OMe **21**, which displayed a ΔCF_3_ of +0.37 ppm. The opposite enantiomer, l-Ala-OMe, was derivatized with MTPA to show a slightly greater Δδ^RS^, reaching −0.386 ppm.^15^ The CFTA amide outperformed both MTPA and TBBA amides, showing an overall Δδ^RS^ of −2.434 ppm.^15^

The transformation of the ester group into the amide forms l-Ala-NH_2_ **22** and the tertiary amide l-Ala-N­(CH_2_CH_3_)_2_ **23** confirmed the preservation of a still strong shielding effect of these functionalities, which was evident from the ΔCF_3_ values at −0.25 and −0.29 ppm, respectively. In general, the study of TBBA amides from amino acids and their functional derivatives further indicated the powerful shielding effect of the ester and amide groups.

Then, it was necessary to compare the shielding effect of other substituents in chiral primary amines in addition to derivatives of amino acids (Figure 4). The *t*-butyl substituent in derivative **24** provided significantly stronger shielding than the methyl group. The cyclohexyl ring, resembling the branching of the isopropyl group in TBBA amide **25**, was still more effective at shielding compared to the methyl group. Interestingly, the isopropyl group in **26** was capable of projecting a stronger shielding effect than the phenyl group. However, as expected, the shielding activity of the phenyl group was stronger than that of methyl, as exemplified by TBBA amide **27**. This derivative was also synthesized as an MTPA amide with nearly the same ΔCF_3_ value (Δδ^RS^ = −0.25 ppm).^10^

**Figure 4:**
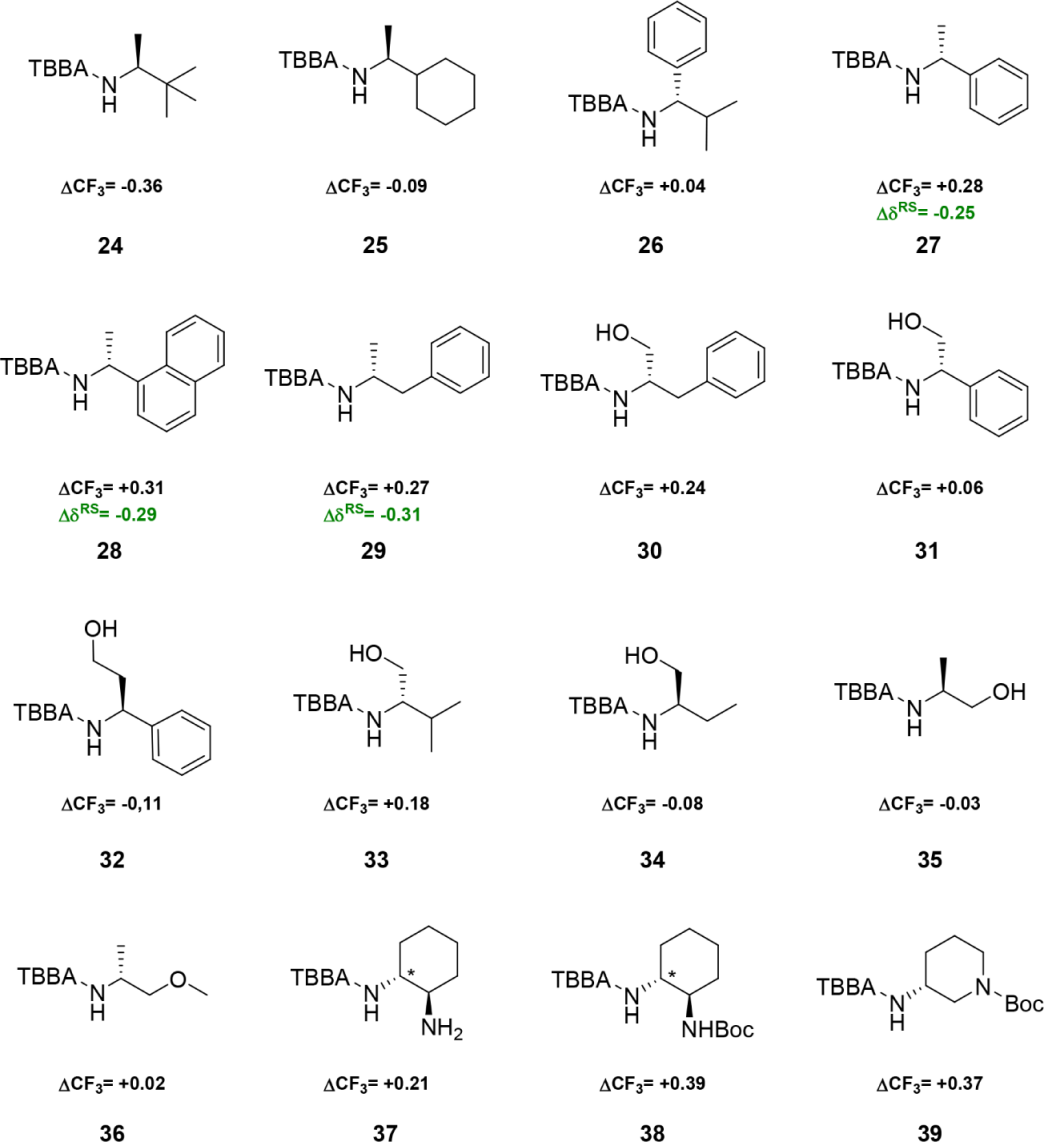
Differences in the ^19^F NMR chemical shifts for diastereoisomeric TBBA amides are represented as ΔCF_3_. The α-chiral position is marked with an asterisk in compounds that contain multiple stereocenters. The samples were analyzed in CDCl_3_ as the solvent, with CFCl_3_ as the internal standard. The calculation was performed using the equation: ΔCF_3_ = δCF_3_^19^F­(*P*) – δCF_3_^19^F­(*M*). The ΔCF_3_ differences are shown for TBBA (black) and MTPA (green).

The addition of the benzene ring, represented by naphthalene derivative **28**, maintained the stronger shielding effect for the aromatic system, and the more remote benzene ring in amphetamine **29** was still able to exert greater shielding than methyl. Comparable differences in ΔCF_3_ were reported for the naphthyl (Δδ^RS^ = −0.29 ppm) and amphetamine (Δδ^RS^ = −0.31 ppm) MTPA amides.^10^ The hydroxy group in phenylalaninol **30** was not able to reverse the sign of the ΔCF_3_ value either. The same trend was observed for phenylglycinol **31**, where the benzene ring is closer to the chiral carbon. The extension of phenylglycinol with a methylene group, resulting in amide **32**, did not surpass the stronger shielding effect of the benzene ring.

The isopropyl group in valinol amide **33**, as well as the ethyl group in amide **34**, demonstrates a more effective shielding activity compared to the hydroxylmethyl group. However, the hydroxymethyl group and its *O*-methylated analog were more efficient than the methyl group, as evidenced by alaninol amides **35** and **36** (with opposite configurations), respectively.

The incorporation of a chiral center in a ring system was studied with cyclohexanediamine and aminopiperidine derivatives. The TBBA amide of diamine **37** showed that the substituted part of the cyclohexane ring exerts more intense shielding, and the Boc group in **38** further strengthened this effect. This trend was observed even with the more remote Boc group in the piperidine derivative **39.**

Interestingly, indene **40** in Figure 5 was not in accordance with the conformational model in Figure 6 and showed the opposite configuration. The observed values suggest that the aliphatic ring with the hydroxyl group provides stronger shielding than the aromatic ring. Similarly, derivatives of leucinol **42** and chloramphenicol **44** exhibited a comparable anomalous behavior.

**Figure 5:**
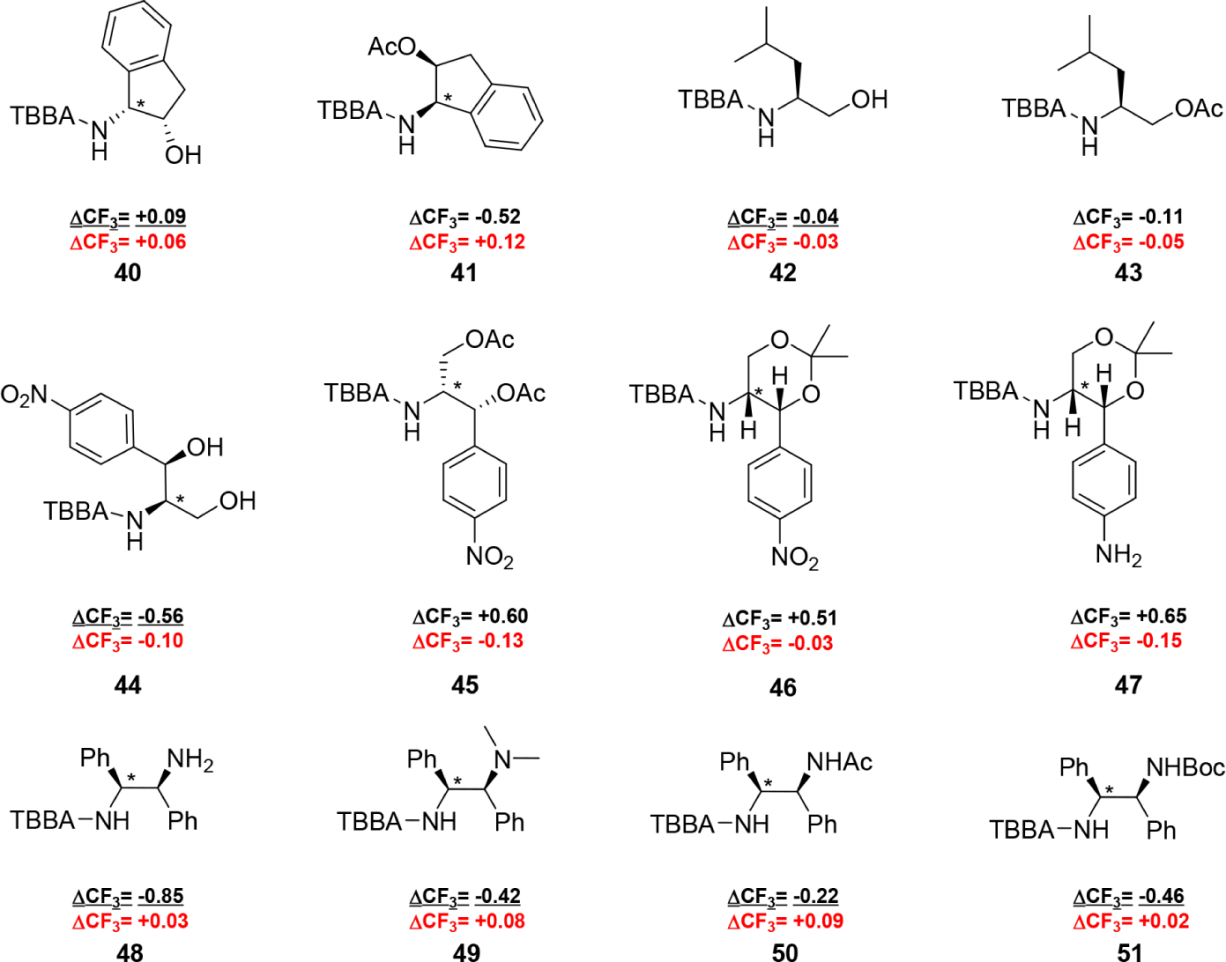
Differences in the ^19^F NMR chemical shifts for diastereoisomeric TBBA amides are represented as ΔCF_3_. The α-chiral position is marked with an asterisk in compounds that contain multiple stereocenters. The samples were analyzed in CDCl_3_ (black) or DMSO-*d*_6_ (red) as the solvent, with CFCl_3_ as the internal standard. The calculation was performed using the equation: ΔCF_3_ = δCF_3_^19^F­(P) – δCF_3_^19^F­(M). Anomalous values in CDCl_3_ are underlined.

We anticipated that the observed deviations from our conformational model were predominantly affected by hydrogen bonding between the hydroxyl group and the amide carbonyl group, which resulted in a change in the conformational equilibrium. Our assumption was subsequently confirmed by the synthesis of acetylated derivatives **41**, **43**, and **45**, where all ΔCF_3_ differences were significantly changed in favor of the conformational model. Furthermore, the cyclic ketal-protected chloramphenicol **46** exhibited the correct configuration as well.

Upon reduction of the nitro group in **46** to obtain amino derivative **47**, ΔCF_3_ increased only slightly. This structural change confirmed that substituents that are remote from the analyzed chiral center had only a minimal impact on the resulting ΔCF_3_.

Incorrect determination was also observed in the case of the TBBA amide **48** derived from (1*S*,2*S*)-1,2-diphenylethane-1,2-diamine. Modifications of amide **48** through methylation, acetylation, and Boc protection to eliminate intramolecular hydrogen bonding did not reverse the ΔCF_3_ differences to align with the suggested conformational model, as is evident from the ΔCF_3_ values of derivatives **49**, **50**, and **51**.

Since the described substitutions did not resolve the discrepancies observed for compounds **48**–**51**, computational chemistry was employed to investigate their most stable conformations. Conformations of compounds **48**–**51** were sampled using Spartan’24 software,^28^ employing the machine-learning-corrected MMFF force field. The default sampling algorithm (“Conformer distribution”) was used to generate up to 100 conformations per compound, with an energy cutoff of 40 kJ/mol. The ten lowest-energy conformers for each molecule were then reoptimized using the def2-TZVP basis set^29^ and the B97 DFT functional with the D3 empirical dispersion term and BJ-damping,^30,31^ all performed with the TurboMole 7.2 program.^32,33^ Solvation effects were modeled with the conductor-like screening model COSMO,^34^ using ε = 4.81 (chloroform). From the DFT-optimized conformers, the lowest-energy structures corresponding to each atropisomeric form (*P* and *M*) were selected to compare with the model geometries, as shown in Figure S1. Notably, none of these compounds adopt geometries consistent with the model shown in Figure 6. In particular, the model assumes that the X atoms (here, NH) orient toward the CF_3_ group, with the carbonyl oxygen pointing in the opposite direction. However, in the lowest-energy conformers of **48**–**51**, the NH group does not orient toward the CF_3_ group in some atropisomers (either *M* or *P*). It is important to note that, for such conformationally rich molecules, the energy differences between individual conformers calculated by DFT are relatively small, and thus higher-energy conformers should also be considered. Even so, most of these also deviate from the model shown in Figure 6. Therefore, our conclusions should remain valid even if such conformers contribute to the observed NMR signal. These unexpected geometries may arise from intramolecular interactions involving bulky substituents, which stabilize alternative conformations. These deviations also explain why methylation **49**, acetylation **50**, and Boc-protection **51** of compound **48** did not bring the conformation into agreement with our model. Coordinates of the minimum-energy structures are provided in the Supporting Information.

Furthermore, we decided to measure all compounds shown in Figure 5 in DMSO-*d*_6_, which is capable of acting as a hydrogen bond acceptor. In this solvent, there is competition between intramolecular and intermolecular hydrogen bonding. Consequently, the formation of conformers stabilized by intramolecular hydrogen bonding is suppressed, and alternative conformers arise that preferentially interact with DMSO-*d*_6_ molecules through intermolecular hydrogen bonding. This polar solvent shifts the conformational equilibria away from our proposed model observed in CDCl_3_, as depicted in Figure 6.

Our proposed general NMR-significant conformational model in a solution of CDCl_3_ for ^19^F NMR spectroscopy (Figure 6a,b) is very similar to the general conformational model for ^1^H NMR spectroscopy,^5^ but this time, a shielding effect of a substituent is projected onto the trifluoromethyl group, taking into account magnetic anisotropy contributions from particular bonds within the substituent.

A comparison of ^19^F NMR chemical shifts between the (*P*)- and (*M*)-TBBA isomers in the diastereomeric pair, by subtraction according to the equation in Figure 6c, gives a difference ΔCF_3_, whose sign assigns the absolute configuration as depicted in Figure 6d.

A library of ΔCF_3_ differences presented in Figures 2–5 enabled the basic classification of substituents according to the magnitude of their shielding effect in CDCl_3_ (Figure 6e). As a result, we noticed that ester and amide groups exert a dominant shielding effect compared to other substituents.

**Figure 6:**
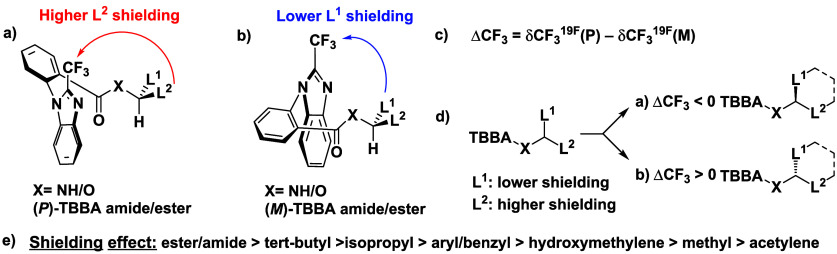
Simplified general NMR-significant conformational model for derivatives measured in CDCl_3_. (a) NMR-significant conformer of the (*P*)-TBBA amide/ester. (b) NMR-significant conformer of the (*M*)-TBBA amide/ester. (c) Equation for the calculation of the differences in chemical shifts between (*P*)- and (*M*)-TBBA amides/esters. (d) Simplified projection for assigning the absolute configuration. (e) Order of substituents according to their shielding effect projected toward the CF_3_ group.

At the opposite end of substituents, the acetylene group emerged. This can be explained by an inversed shielding effect due to magnetic anisotropy. Furthermore, there is an obvious trend that more branched alkyl groups show a higher shielding effect, which can be attributed to a greater number of specific bonds participating in the overall effect.

The shielding effect of the benzene ring was not affected by para-substitution, which was confirmed by derivatives of chloramphenicol.

Finally, we evaluated the robustness of the chemical shifts. Analysis of a mixture of TBBA diastereomers **(** *P* **)-34** and **(** *M* **)-34** in a 2:1 molar ratio revealed that the ^19^F NMR chemical shifts were consistent with those obtained from the pure samples (see Supporting Information).

## Conclusions

In summary, the scope and limitations of TBBA as an axially chiral derivatizing agent were demonstrated for determining the absolute configuration of α-chiral primary amines and secondary alcohols using ^19^F NMR spectroscopy. The method was evaluated on 46 structurally diverse diastereomeric pairs, which enabled the basic classification of substituents according to the magnitude of their shielding effect on the trifluoromethyl group.

The proposed simplified general NMR-significant conformational model reliably assigned the absolute configuration for 39 TBBA derivatives. Intramolecular hydrogen bonding altered the conformational equilibrium and led to incorrect assignments for three TBBA amides. Their modification to eliminate intramolecular hydrogen bonding yielded derivatives in accordance with the conformational model. The TBBA amide based on the 1,2-diphenylethane-1,2-diamine motif was not compatible with the conformational model even after derivatization, indicating a significant change in the conformational equilibrium due to the bulkiness and conformational complexity of its substituents.

Although some limitations of using TBBA as a chiral derivatizing agent were described in this study, the method yielded promising results and can be significantly more reliable than the use of Mosher’s acid (MTPA) in ^19^F NMR spectroscopy. The advantage of ^19^F NMR spectra is their straightforward interpretation and the low probability of overlapping signals, as fluorine atoms are not usually present in the analyzed compounds. However, an assignment based on a single data point obtained from a ΔCF_3_ difference can be potentially misleading, but complementary ^1^H NMR spectra of TBBA amides or esters can always provide additional data points to further support the determination of absolute configuration.

## Experimental Section

### General Information

All reactions were performed under normal conditions without any specific precautions to exclude moisture or air from the reaction, except where stated otherwise. Reaction workup and column chromatography were performed with commercial-grade solvents without further purification. ^1^H NMR, ^13^C NMR, and ^19^F NMR spectra were measured on a Jeol ECA400II (400 MHz) or Jeol ECX-500SS (500 MHz) instrument in CDCl_3_, DMSO-*d*_6_, or D_2_O as a solvent. ^1^H and ^13^C spectra were referenced to residual nondeuterated solvent signals (7.26 and 77.16 ppm for CDCl_3_, 2.50 and 39.52 ppm for DMSO-*d*_6_). ^19^F spectra were referenced to a CFCl_3_ standard (δ = 0.0 ppm). All ^13^C NMR spectra were acquired with broadband ^1^H decoupling. ^1^H NMR data are reported as follows: δ (chemical shift, ppm), coupling constants (*J*, Hz), and integration. Abbreviations used to denote signal multiplicity were as follows: s (singlet), d (doublet), t (triplet), q (quartet), m (multiplet), app (appears as), and br (broad).

Analytical thin-layer chromatography (TLC) was performed using Kieselgel 60 F_254_ plates (Merck). Compounds were detected under UV light (255 nm) and then by staining with basic KMnO_4_ solution or ninhydrin solution.

Flash chromatography was performed using silica gel (35–70 μm particle size range).

HRMS analyses were carried out using an Exactive Plus Orbitrap high-resolution mass spectrometer with electrospray ionization (Thermo Fisher Scientific, MA, USA). Chromatographic preseparation was performed using an HPLC system Dionex Ultimate 3000 (Thermo Fisher Scientific, MA, USA) equipped with a Phenomenex Gemini column (C18, 50 × 2 mm, 3.0 μm). The samples were dissolved in MeOH or acetonitrile and injected via an autosampler. Mobile phase composition: isocratic elution with MeOH/water 95:5 + 0.1% (v/v) HCOOH at a flow rate of 0.3 mL/min.

### Synthesis of TBBA Derivatives

Compounds **6**–**8**, **10**–**15**, **18**, **21**, **24**, **27**–**28**, **31**, and **33** were prepared according to previously reported procedures and their characterization data were consistent with reported values.^5^

### General Procedure A: Synthesis of TBBA Amides **16**–**40**; **42**; **44**; **46**–**49**; **51**

(*P*)- or (*M*)-TBBA (0.13 mmol, 1 equiv, 40 mg) was dissolved in DMF (2 mL). Then, EDCI (0.26 mmol, 2 equiv, 50 mg), HOBt (0.26 mmol, 2 equiv, 40 mg), and amine (0.13 mmol, 1 equiv) were subsequently added to the solution. The reaction was stirred at room temperature for 12 h. After completion, the reaction was diluted with EtOAc (10 mL), forming a cloudy white solution, which was washed with 10% aq. HCl (3 × 10 mL), 10% K_2_CO_3_ (3 × 10 mL), and brine (1 × 10 mL). The organic layer was dried over MgSO_4_ and evaporated under reduced pressure. The residue was purified by column chromatography (CC).

### General Procedure B: Synthesis of TBBA Esters **6**–**15**

(*P*)- or (*M*)-TBBA (0.13 mmol, 1 equiv, 40 mg) was dissolved in dry CH_2_Cl_2_ (6 mL). Subsequently, alcohol (0.13 mmol, 1 equiv), DCC (0.13 mmol, 1 equiv, 27 mg), and DMAP (0.13 mmol, 1 equiv, 16 mg) were added to the solution. The reaction was left to stir for 16 h at room temperature. Subsequently, the reaction mixture was filtered through a syringe filter, which was washed with CH_2_Cl_2_ (10 mL). The filtrate was washed with 10% aq. HCl (3 × 10 mL), 10% K_2_CO_3_ (3 × 10 mL), and brine (1 × 10 mL). The organic layer was dried over MgSO_4_ and evaporated under reduced pressure. The residue was purified by column chromatography (CC).

### General Procedure C: Synthesis of TBBA Acetyl Amides **41**; **43**; **45**; **50**

Alcohol or amine (0.13 mmol, 1 equiv) was dissolved in CH_2_Cl_2_ (8 mL). Acetic anhydride (0.26 mmol, 2 equiv, 25 μL), trimethylamine (0.39 mmol, 3 equiv, 54 μL), and DMAP (0.013 mmol, 0.1 equiv, 1.6 mg) were added to the solution. The reaction was stirred at room temperature for 2 h. Subsequently, it was diluted with CH_2_Cl_2_ (10 mL) and washed with 10% aq. HCl (3 × 10 mL), 10% K_2_CO_3_ (3 × 10 mL), and brine (1 × 10 mL). The organic layer was dried over MgSO_4_ and evaporated under reduced pressure. The residue was purified by column chromatography (CC).

#### (*R*)-Heptan-2-yl 2-((*P*)-2-(trifluoromethyl)-1*H*-benzo­[*d*]­imidazol-1-yl)­benzoate **(** *P* **)-9**

Following the general procedure **B**, (*P*)-TBBA and (*S*)-heptan-2-ol (0.13 mmol, 1 equiv, 19 μL) were used, followed by purification by CC (hexane/EtOAc 18:1, column dimensions: 2 × 10 cm), yielding 19 mg (36%) of a white solid^.**1**^ **H NMR** (400 MHz, CDCl_3_) δ 8.32–8.24 (m, 1H), 7.97–7.91 (m, 1H), 7.76 (td, *J* = 7.5, 1.9 Hz, 1H), 7.71 (td, *J* = 7.6, 1.6 Hz, 1H), 7.47 (dd, *J* = 7.5, 1.5 Hz, 1H), 7.41–7.37 (m, 1H), 7.34 (ddd, *J* = 8.4, 7.2, 1.3 Hz, 1H), 6.98 (ddd, *J* = 7.9, 1.3, 0.8 Hz, 1H), 4.79–4.70 (m, 1H), 1.14–0.92 (m, 4H), 0.89 (d, *J* = 6.3 Hz, 3H), 0.88–0.81 (m, 1H), 0.79 (t, *J* = 7.3 Hz, 3H), 0.76–0.55 (m, 3H); ^**13**^ **C­{** ^**1**^ **H} NMR** (101 MHz, CDCl_3_) δ 164.1, 141.2 (q, *J* = 38.5 Hz), 140.8, 138.1, 133.7, 133.5, 132.9, 130.7, 130.2, 130.2, 125.9, 124.0, 121.5, 119.0 (q, *J* = 271.4 Hz), 111.0, 73.0, 35.1, 31.5, 24.9, 22.5, 19.4, 14.1; ^**19**^ **F NMR** (376 MHz, CDCl_3_) δ −62.04 (s); **HRMS** (ESI) *m*/*z* [M + H]^+^ Calcd for C_22_H_24_F_3_N_2_O_2_ 405.1784; Found 405.1783; ${[\alpha ]}_{d}^{22}$ = −29.09° (*c* = 0.11, CHCl_3_).

#### (*R*)-Heptan-2-yl 2-((*P*)-2-(trifluoromethyl)-1*H*-benzo­[*d*]­imidazol-1-yl)­benzoate **(** *M* **)-9**

Following the general procedure **B**, (*M*)-TBBA and (*S*)-heptan-2-ol (0.13 mmol, 1 equiv, 19 μL) were used, followed by purification by CC (hexane/EtOAc 18:1, column dimensions: 2 × 10 cm), yielding 20 mg (38%) of a white solid. ^**1**^ **H NMR** (400 MHz, CDCl_3_) δ 8.29–8.20 (m, 1H), 7.94 (ddd, *J* = 8.1, 1.2, 0.8 Hz, 1H), 7.75 (td, *J* = 7.6, 1.9 Hz, 1H), 7.70 (td, *J* = 7.6, 1.5 Hz, 1H), 7.48 (dd, *J* = 7.6, 1.3 Hz, 1H), 7.38 (ddd, *J* = 8.1, 7.2, 1.4 Hz, 1H), 7.33 (ddd, *J* = 8.4, 7.2, 1.3 Hz, 1H), 6.97 (ddd, *J* = 7.9, 1.3, 0.8 Hz, 1H), 4.76–4.67 (m, 1H), 1.22–0.99 (m, 8H), 0.83 (t, *J* = 7.1 Hz, 3H), 0.39 (d, *J* = 6.3 Hz, 3H); ^**13**^ **C­{** ^**1**^ **H} NMR** (101 MHz, CDCl_3_) δ 164.3, 141.1 (q, *J* = 38.6 Hz), 140.8, 138.0, 133.7, 133.4, 132.7, 130.6, 130.2, 130.0, 125.9, 124.0, 121.5, 119.0 (q, *J* = 271.9 Hz), 111.0, 73.0, 35.4, 31.6, 25.0, 22.6, 18.6, 14.1; ^**19**^ **F NMR** (376 MHz, CDCl_3_) δ −61.85 (s); **HRMS** (ESI) *m*/*z* [M + H]^+^ Calcd for C_22_H_24_F_3_N_2_O_2_ 405.1784; Found 405.1777; ${[\alpha ]}_{d}^{22}$ = +135.00° (*c* = 0.10, CHCl_3_).

#### Methyl (*S*)-3,3-dimethyl-2-(2-((*P*)-2-(trifluoromethyl)-1*H*-benzo­[*d*]­imidazol-1-yl)­benzamido)­butanoate **(** *P* **)-16**

Following the general procedure **A**, (*P*)-TBBA and l-tert-leucine methyl ester hydrochloride (0.13 mmol, 1 equiv, 24 mg), and triethylamine (0.14 mmol, 1.1 equiv, 20 μL) were used, followed by purification by CC (CH_2_Cl_2_/MeOH 99:1, column dimensions: 2 × 10 cm), yielding 48 mg (88%) of a white foamy solid. ^**1**^ **H NMR** (400 MHz, CDCl_3_) δ 7.95–7.88 (m, 1H), 7.88–7.84 (m, 1H), 7.71–7.65 (m, 2H), 7.43–7.37 (m, 3H), 7.12–7.07 (m, 1H), 6.08 (d, *J* = 9.2 Hz, 1H), 4.29 (d, *J* = 9.3 Hz, 1H), 3.56 (s, 3H), 0.67 (s, 9H); ^**13**^ **C­{** ^**1**^ **H} NMR** (101 MHz, CDCl_3_) δ 171.3, 165.2, 141.2 (q, *J* = 38.3 Hz), 140.9, 137.6, 135.0, 132.0, 131.8, 130.9, 129.9, 126.4, 124.4, 121.8, 119.0 (q, *J* = 272.1 Hz), 111.1, 60.5, 52.0, 34.7, 26.3; ^**19**^ **F NMR** (376 MHz, CDCl_3_) δ −61.58 (s); **HRMS** (ESI) *m*/*z* [M + H]^+^ Calcd for C_22_H_23_F_3_N_3_O_3_ 434.1686; Found 434.1685; ${[\alpha ]}_{d}^{22}$ = −130.00° (*c* = 0.1, MeOH).

#### Methyl (*S*)-3,3-dimethyl-2-(2-((*M*)-2-(trifluoromethyl)-1*H*-benzo­[*d*]­imidazol-1-yl)­benzamido)­butanoate **(** *M* **)-16**

Following the general procedure **A**, (*M*)-TBBA and l-tert-leucine methyl ester hydrochloride (0.13 mmol, 1 equiv, 24 mg) and triethylamine (0.14 mmol, 1.1 equiv, 20 μL) were used, followed by purification by CC (CH_2_Cl_2_/MeOH 99:1, column dimensions: 2 × 10 cm), yielding 46 mg (84%) of a white foamy solid. ^**1**^ **H NMR** (400 MHz, CDCl_3_) δ 7.94–7.87 (m, 2H), 7.72–7.65 (m, 2H), 7.41–7.34 (m, 3H), 7.12–7.07 (m, 1H), 6.08 (d, *J* = 9.0 Hz, 1H), 4.26 (d, *J* = 9.1 Hz, 1H), 3.64 (s, 3H), 0.59 (s, 9H); ^**13**^ **C­{** ^**1**^ **H} NMR** (101 MHz, CDCl_3_) δ 171.5, 165.3, 140.8, 140.8 (q, *J* = 38.3 Hz), 137.5, 134.8, 132.0, 131.5, 130.9, 130.0, 129.8, 126.4, 124.4, 121.7, 119.0 (q, *J* = 272.1 Hz), 111.4, 60.7, 52.0, 34.4, 26.1; ^**19**^ **F NMR** (376 MHz, CDCl_3_) δ −61.24 (s); **HRMS** (ESI) *m*/*z* [M + H]^+^ Calcd for C_22_H_23_F_3_N_3_O_3_ 434.1686; Found 434.1682; ${[\alpha ]}_{d}^{22}$ = +66.67° (*c* = 0.1, MeOH).

#### Methyl (2-((*P*)-2-(trifluoromethyl)-1*H*-benzo­[*d*]­imidazol-1-yl)­benzoyl)-l-valinate **(** *P* **)-17**

Following the general procedure **A**, (*P*)-TBBA and l-valine methyl ester hydrochloride (0.13 mmol, 1 equiv, 22 mg) and triethylamine (0.14 mmol, 1.1 equiv, 20 μL) were used, followed by purification by CC (CH_2_Cl_2_/MeOH 99:1, column dimensions: 2 × 10 cm), yielding 44 mg (81%) of a white foamy solid. ^**1**^ **H NMR** (400 MHz, CDCl_3_) δ 7.95–7.91 (m, 1H), 7.90–7.85 (m, 1H), 7.72–7.66 (m, 2H), 7.44–7.37 (m, 3H), 7.13–7.07 (m, 1H), 6.01 (d, *J* = 8.4 Hz, 1H), 4.39 (dd, *J* = 8.7, 4.6 Hz, 1H), 3.59 (s, 3H), 1.89 (pd, *J* = 6.9, 4.6 Hz, 1H), 0.59 (d, *J* = 6.9 Hz, 3H), 0.55 (d, *J* = 6.9 Hz, 3H); ^**13**^ **C­{** ^**1**^ **H} NMR** (101 MHz, CDCl_3_) δ 171.7, 165.3, 141.2 (q, *J* = 38.7 Hz), 140.9, 137.6, 135.0, 132.0, 131.9, 130.9, 129.8, 126.4, 124.4, 121.8, 118.9 (q, *J* = 272.1 Hz), 111.1, 57.5, 52.3, 31.2, 18.5, 17.3; ^**19**^ **F NMR** (376 MHz, CDCl_3_) δ −61.63 (s); **HRMS** (ESI) *m*/*z* [M + H]^+^ Calcd for C_21_H_21_F_3_N_3_O_3_ 420.1530; Found 420.1534; ${[\alpha ]}_{d}^{22}$ = −98.40° (*c* = 0.07, MeOH).

#### Methyl (2-((*M*)-2-(trifluoromethyl)-1*H*-benzo­[*d*]­imidazol-1-yl)­benzoyl)-l-valinate **(** *M* **)-17**

Following the general procedure **A**, (*M*)-TBBA and l-valine methyl ester hydrochloride (0.13 mmol, 1 equiv, 22 mg) and triethylamine (0.14 mmol, 1.1 equiv, 20 μL) were used, followed by purification by CC (CH_2_Cl_2_/MeOH 99:1, column dimensions: 2 × 10 cm), yielding 46 mg (85%) of a white foamy solid. ^**1**^ **H NMR** (400 MHz, CDCl_3_) δ 7.94–7.87 (m, 2H), 7.72–7.65 (m, 2H), 7.43–7.35 (m, 3H), 7.12–7.07 (m, 1H), 6.04 (d, *J* = 8.4 Hz, 1H), 4.37 (dd, *J* = 8.5, 4.4 Hz, 1H), 3.65 (s, 3H), 1.83 (pd, *J* = 6.9, 4.4 Hz, 1H), 0.51 (d, *J* = 6.9 Hz, 3H), 0.44 (d, *J* = 6.9 Hz, 3H); ^**13**^ **C­{** ^**1**^ **H} NMR** (101 MHz, CDCl_3_) δ 171.8, 165.5, 140.8, 140.8 (q, *J* = 38.4 Hz), 137.5, 134.9, 132.0, 131.6, 130.9, 130.0, 129.8, 126.4, 124.4, 121.7, 119.0 (q, *J* = 271.8 Hz), 111.5, 57.5, 52.3, 31.0, 18.3, 17.2; ^**19**^ **F NMR** (376 MHz, CDCl_3_) δ −61.23 (s); **HRMS** (ESI) *m*/*z* [M + H]^+^ Calcd for C_21_H_21_F_3_N_3_O_3_ 420.1530; Found 420.1528; ${[\alpha ]}_{d}^{22}$ = +41.88° (*c* = 0.13, MeOH).

#### *tert*-Butyl *o*-(*tert*-butyl)-*N*-(2-((*P*)-2-(trifluoromethyl)-1*H*-benzo­[*d*]­imidazol-1-yl)­benzoyl)-l-serinate **(** *P* **)-19**

Following the general procedure **A**, (*P*)-TBBA and *H*-Ser­(*t*Bu)-O*t*Bu·HCl (0.13 mmol, 1 equiv, 33 mg) and triethylamine (0.14 mmol, 1.1 equiv, 20 μL) were used, followed by purification by CC (CH_2_Cl_2_/MeOH 99:1, column dimensions: 2 × 10 cm), yielding 49 mg (75%) of a white solid. ^**1**^ **H NMR** (400 MHz, CDCl_3_) δ 7.93–7.86 (m, 2H), 7.70–7.65 (m, 2H), 7.44–7.40 (m, 1H), 7.39–7.32 (m, 2H), 7.12–7.03 (m, 1H), 6.50 (d, *J* = 8.1 Hz, 1H), 4.41 (dt, *J* = 8.1, 2.8 Hz, 1H), 3.56 (dd, *J* = 8.8, 2.8 Hz, 1H), 3.19 (dd, *J* = 8.8, 2.9 Hz, 1H), 1.39 (s, 9H), 1.02 (s, 9H); ^**13**^ **C­{** ^**1**^ **H} NMR** (101 MHz, CDCl_3_) δ 168.9, 164.9, 141.4 (q, *J* = 38.3 Hz), 140.9, 137.6, 134.8, 132.5, 131.9, 130.7, 129.9, 129.5, 126.0, 124.0, 121.7, 119.0 (q, *J* = 271.9 Hz), 111.1, 82.1, 73.1, 62.0, 53.5, 28.1, 27.3; ^**19**^ **F NMR** (376 MHz, CDCl_3_) δ −61.65 (s); **HRMS** (ESI) *m*/*z* [M + H]^+^ Calcd for C_26_H_31_F_3_N_3_O_4_ 506.2261; Found 506.2263; ${[\alpha ]}_{d}^{22}$ = +66.15° (*c* = 0.13, CHCl_3_).

#### *tert*-Butyl *O*-(*tert*-butyl)-*N*-(2-((*M*)-2-(trifluoromethyl)-1*H*-benzo­[*d*]­imidazol-1-yl)­benzoyl)-l-serinate **(** *M* **)-19**

Following the general procedure **A**, (*M*)-TBBA and H-Ser­(*t*Bu)-O*t*Bu·HCl (0.13 mmol, 1 equiv, 33 mg) and triethylamine (0.14 mmol, 1.1 equiv, 20 μL) were used, followed by purification by CC (CH_2_Cl_2_/MeOH 99:1, column dimensions: 2 × 10 cm), yielding 45 mg (69%) of a white solid. ^**1**^ **H NMR** (400 MHz, CDCl_3_) δ 7.92–7.86 (m, 2H), 7.70–7.64 (m, 2H), 7.44–7.40 (m, 1H), 7.39–7.33 (m, 2H), 7.16–7.08 (m, 1H), 6.46 (d, *J* = 8.5 Hz, 1H), 4.35 (dt, *J* = 8.7, 2.8 Hz, 1H), 3.55 (dd, *J* = 8.7, 2.6 Hz, 1H), 2.97 (dd, *J* = 8.7, 2.9 Hz, 1H), 1.41 (s, 9H), 1.06 (s, 9H); ^**13**^ **C­{** ^**1**^ **H} NMR** (101 MHz, CDCl_3_) δ 169.0, 165.3, 140.7 (q, *J* = 38.3 Hz), 140.6, 137.8, 135.1, 132.0, 131.7, 130.7, 129.7, 129.5, 126.1, 124.1, 121.4, 119.1 (q, *J* = 271.8 Hz), 111.8, 82.0, 73.2, 61.9, 53.3, 28.1, 27.2; ^**19**^ **F NMR** (376 MHz, CDCl_3_) δ −61.19 (s); **HRMS** (ESI) *m*/*z* [M + H]^+^ Calcd for C_26_H_31_F_3_N_3_O_4_ 506.2261; Found 506.2267; ${[\alpha ]}_{d}^{22}$ = +170.00° (*c* = 0.14, CHCl_3_).

#### Methyl (2-((*P*)-2-(trifluoromethyl)-1*H*-benzo­[*d*]­imidazol-1-yl)­benzoyl)-l-leucinate **(** *P* **)-20**

Following the general procedure **A**, (*P*)-TBBA, l-leucine methyl ester hydrochloride (0.13 mmol, 1 equiv, 22 mg), and triethylamine (0.14 mmol, 1.1 equiv, 20 μL) were used, followed by purification by CC (hexane/EtOAc 5:2, column dimensions: 2 × 10 cm), yielding 30 mg (53%) of a yellow solid. ^**1**^ **H NMR** (400 MHz, CDCl_3_) δ (ppm 7.96–7.91 (m, 1H), 7.89–7.83 (m, 1H), 7.71–7.66 (m, 2H), 7.45–7.36 (m, 3H), 7.11–7.06 (m, 1H), 5.87 (d, *J* = 8.0 Hz, 1H), 4.44 (td, *J* = 8.8, 5.2 Hz, 1H), 3.58 (s, 3H), 1.43–1.33 (m, 1H), 1.18–1.02 (m, 2H), 0.72 (d, *J* = 6.5 Hz, 3H), 0.68 (d, *J* = 6.5 Hz, 3H); ^**13**^ **C­{** ^**1**^ **H} NMR** (126 MHz, CDCl_3_) δ (ppm) 172.7, 164.9, 141.3 (q, *J* = 38.5 Hz), 140.8, 137.5, 134.9, 131.9, 131.9, 130.8, 129.7, 129.7, 126.2, 124.3, 121.7, 118.9 (q, *J* = 272.1 Hz), 110.9, 52.4, 50.9, 41.5, 24.6, 22.7, 21.6; ^**19**^ **F NMR** (376 MHz, CDCl_3_) δ (ppm) −61.67 (s); **HRMS** (ESI) *m*/*z* [M + H]^+^ Calcd for C_23_H_23_F_3_N_3_O_2_ 434.1686; Found 434.1691; ${[\alpha ]}_{d}^{22}$ = −123.81° (*c* = 0.07, CHCl_3_).

#### Methyl (2-((*M*)-2-(trifluoromethyl)-1*H*-benzo­[*d*]­imidazol-1-yl)­benzoyl)-l-leucinate **(** *M* **)-20**

Following the general procedure **A**, (*M*)-TBBA, l-leucine methyl ester hydrochloride (0.13 mmol, 1 equiv, 22 mg) and triethylamine (0.14 mmol, 1.1 equiv, 20 μL) were used, followed by purification by CC (hexane/EtOAc 5:2, column dimensions: 2 × 10 cm), yielding 34 mg (60%) of a white solid. ^**1**^ **H NMR** (400 MHz, CDCl_3_) δ (ppm) 7.96–7.85 (m, 2H), 7.72–7.65 (m, 2H), 7.45–7.35 (m, 3H), 7.13–7.06 (m, 1H), 5.85 (d, *J* = 8.0 Hz, 1H), 4.45–4.33 (m, 1H), 3.63 (s, 3H), 1.30–1.22 (m, 1H), 1.01 (ddd, *J* = 13.8, 9.1, 5.9 Hz, 1H), 0.92–0.81 (m, 1H), 0.67 (d, *J* = 6.6 Hz, 3H), 0.60 (d, *J* = 6.5 Hz, 3H); ^**13**^ **C­{** ^**1**^ **H} NMR** (101 MHz, CDCl_3_) δ (ppm) 172.8, 165.2, 140.7, 140.7 (q, *J* = 38.5 Hz), 137.5 (q, *J* = 1.3 Hz), 134.9, 132.0, 131.6, 130.9, 130.0, 129.7 (app. d, *J* = 0.9 Hz), 126.4, 124.5, 121.7, 119.1 (q, *J* = 272.0 Hz), 111.6, 52.5, 51.0, 41.4, 24.5, 22.6, 21.6; ^**19**^ **F NMR** (376 MHz, CDCl_3_) δ (ppm) −61.22 (s); **HRMS** (ESI) *m*/*z* [M + H]^+^ Calcd for C_23_H_23_F_3_N_3_O_2_ 434.1686; Found 434.1688; ${[\alpha ]}_{d}^{22}$ = +129.63° (*c* = 0.09, CHCl_3_).

#### *N*-((*S*)-1-Amino-1-oxopropan-2-yl)-2-((*P*)-2-(trifluoromethyl)-1*H*-benzo­[*d*]­imidazol-1-yl)­benzamide **(** *P* **)-22**

The procedure was inspired from the literature.^35^

To a solution of 7 M ammonia in MeOH (2 mL) was added H-Ala-OMe·HCl (0.13 mmol, 1 equiv, 18 mg) and KOH (0.13 mmol, 1 equiv, 7.5 mg). The mixture was stirred at room temperature for 20 h. Afterward, the solvent was evaporated under reduced pressure. Subsequently, MeOH (3 mL) was added to the residue and evaporated again. This process was repeated three times, followed by two more repetitions with EtOH (3 mL), and three more repetitions with CHCl_3_ (3 mL). The residue was dissolved in DMF (2 mL). (*P*)-TBBA (0.13 mmol, 1 equiv, 40 mg), EDCl (0.26 mmol, 2 equiv, 50 mg), and HOBt (0.26 mmol, 2 equiv, 40 mg) were gradually added. The reaction was stirred at room temperature for 12 h. After completion, the reaction mixture was diluted with EtOAc (10 mL), which was washed with 10% K_2_CO_3_ (6 × 10 mL) and brine (1 × 10 mL). The organic layer was dried over MgSO_4_ and evaporated under reduced pressure. The residue was purified by CC (CH_2_Cl_2_/MeOH 20:1, column dimensions: 2 × 10 cm), yielding 34 mg (71%) of a white solid. ^**1**^ **H NMR** (400 MHz, CDCl_3_) δ 7.96–7.89 (m, 1H), 7.84–7.77 (m, 1H), 7.73–7.65 (m, 2H), 7.51–7.46 (m, 1H), 7.43–7.36 (m, 2H), 7.11–7.03 (m, 1H), 6.33 (d, *J* = 7.3 Hz, 1H), 5.79 (br. s, 1H), 5.06 (br. s, 1H), 4.26 (p, *J* = 7.0 Hz, 1H), 0.92 (d, *J* = 6.9 Hz, 3H); ^**13**^ **C­{** ^**1**^ **H} NMR** (101 MHz, DMSO-*d*_6_) δ 173.6, 164.7, 140.4 (q, *J* = 38.3 Hz), 140.2, 136.8, 134.7, 131.7, 131.3, 130.2, 129.1, 129.1, 125.4, 123.6, 120.6, 118.7 (q, *J* = 271.9 Hz), 111.5, 48.0, 17.4; ^**19**^ **F NMR** (376 MHz, CDCl_3_) δ −61.70 (s); **HRMS** (ESI) *m*/*z* [M + H]^+^ Calcd for C_18_H_16_F_3_N_4_O_2_ 377.1220; Found 377.1220; ${[\alpha ]}_{d}^{22}$ = −170.37° (*c* = 0.09, MeOH).

#### *N*-((*S*)-1-Amino-1-oxopropan-2-yl)-2-((*M*)-2-(trifluoromethyl)-1*H*-benzo­[*d*]­imidazol-1-yl)­benzamide **(** *M* **)-22**

Following the same procedure as for **(** *P* **)-22**, the reaction using (*M*)-TBBA (0.13 mmol, 1 equiv, 40 mg) yielded 35 mg (73%) of a white solid. ^**1**^ **H NMR** (400 MHz, CDCl_3_) δ 7.94–7.90 (m, 1H), 7.82–7.78 (m, 1H), 7.73–7.66 (m, 2H), 7.51–7.46 (m, 1H), 7.42–7.34 (m, 2H), 7.07–7.01 (m, 1H), 6.30 (d, *J* = 7.7 Hz, 1H), 5.57 (br. s, 1H), 4.99 (br. s, 1H), 4.23 (p, *J* = 7.0 Hz, 1H), 1.01 (d, *J* = 7.0 Hz, 3H); ^**13**^ **C­{** ^**1**^ **H} NMR** (101 MHz, DMSO-*d*_6_) δ 173.5, 164.4, 140.0, 139.8 (q, *J* = 37.8 Hz), 137.5, 134.0, 131.9, 131.6, 130.3, 129.5, 125.7, 123.6, 120.6, 118.8 (q, *J* = 271.7 Hz), 111.6, 48.4, 17.7; ^**19**^ **F NMR** (376 MHz, CDCl_3_) δ −61.44 (s); **HRMS** (ESI) *m*/*z* [M + H]^+^ Calcd for C_18_H_16_F_3_N_4_O_2_ 377.1220; Found 377.1222; ${[\alpha ]}_{d}^{22}$ = +45.56° (*c* = 0.09, MeOH).

#### *N*-((*S*)-1-(Diethylamino)-1-oxopropan-2-yl)-2-((*P*)-2-(trifluoromethyl)-1*H*-benzo­[*d*]­imidazol-1-yl)­benzamide **(** *P* **)-23**

The procedure was inspired by the literature.^36^

Diethylamide **60** (0.13 mmol, 1 equiv, 47 mg) was dissolved in THF (3 mL) and KOH (0.39 mmol, 3 equiv, 7 mg) was added. The mixture was stirred at room temperature for 30 min. The pH of the reaction mixture was adjusted to 7 with 5 M HCl, and the solvent was evaporated under a stream of nitrogen. The residue was dissolved in DMF (2 mL). (*P*)-TBBA (0.13 mmol, 1 equiv, 40 mg), EDCl (0.26 mmol, 2 equiv, 50 mg), and HOBt (0.26 mmol, 2 equiv, 40 mg) were gradually added. The reaction mixture was stirred at room temperature for 12 h. After completion, the reaction mixture was diluted with EtOAc (10 mL), which was washed with 10% aq. HCl (3 × 10 mL), 10% K_2_CO_3_ (3 × 10 mL), and brine (1 × 10 mL). The organic layer was dried over MgSO_4_ and evaporated under reduced pressure. The residue was purified by CC (CH_2_Cl_2_/MeOH 98:2, column dimensions 2 × 10 cm), yielding 45 mg (80%) of a light-yellow solid. ^**1**^ **H NMR** (400 MHz, CDCl_3_) δ 7.92–7.88 (m, 1H), 7.80–7.75 (m, 1H), 7.68–7.62 (m, 2H), 7.48–7.43 (m, 1H), 7.38–7.34 (m, 2H), 7.10–7.04 (m, 1H), 6.83 (d, *J* = 7.3 Hz, 1H), 4.57 (p, *J* = 6.7 Hz, 1H), 3.45–3.36 (m, 1H), 3.24–3.06 (m, 3H), 1.06 (t, *J* = 7.1 Hz, 3H), 1.04 (t, *J* = 7.2 Hz, 3H), 0.91 (d, *J* = 6.7 Hz, 3H); ^**13**^ **C­{** ^**1**^ **H} NMR** (101 MHz, CDCl_3_) δ 171.0, 164.4, 141.4 (q, *J* = 38.5 Hz), 140.8, 137.6, 135.0, 132.4, 131.7, 130.6, 129.6, 128.9, 126.0, 124.0, 121.6, 119.0 (q, *J* = 272.3 Hz), 111.2, 45.7, 41.7, 40.4, 18.9, 14.5, 12.9; ^**19**^ **F NMR** (376 MHz, CDCl_3_) δ −61.67 (s); **HRMS** (ESI) *m*/*z* [M + H]^+^ Calcd for C_22_H_24_F_3_N_4_O_2_ 433.1846; Found 433.1844; ${[\alpha ]}_{d}^{22}$ = −170.08° (*c* = 0.13, MeOH).

#### *N*-((*S*)-1-(Diethylamino)-1-oxopropan-2-yl)-2-((*M*)-2-(trifluoromethyl)-1*H*-benzo­[*d*]­imidazol-1-yl)­benzamide **(** *M* **)-23**

Following the same procedure as for **(** *P* **)-23**, the reaction using (*M*)-TBBA (0.13 mmol, 1 equiv, 40 mg) yielded 38 mg (68%) of light-yellow solid. **1H NMR** (400 MHz, CDCl_3_) δ 7.91–7.88 (m, 1H), 7.80–7.75 (m, 1H), 7.68–7.63 (m, 2H), 7.48–7.44 (m, 1H), 7.37–7.32 (m, 2H), 7.09–7.04 (m, 1H), 6.84 (d, *J* = 7.4 Hz, 1H), 4.58 (p, *J* = 6.8 Hz, 1H), 3.49–3.39 (m, 1H), 3.24–3.05 (m, 3H), 1.06 (t, *J* = 7.1 Hz, 3H), 1.02 (t, *J* = 7.1 Hz, 3H), 0.92 (d, *J* = 6.7 Hz, 3H); ^**13**^ **C­{** ^**1**^ **H} NMR** (101 MHz, CDCl_3_) δ 171.0, 164.5, 141.0 (q, *J* = 38.3 Hz), 140.7, 137.7, 135.1, 132.3, 131.7, 130.7, 129.7, 129.0, 126.0, 124.0, 121.5, 119.0 (q, *J* = 272.1 Hz), 111.5, 45.6, 41.6, 40.3, 18.8, 14.4, 12.9; ^**19**^ **F NMR** (376 MHz, CDCl_3_) δ −61.39 (s); **HRMS** (ESI) *m*/*z* [M + H]^+^ Calcd for C_22_H_24_F_3_N_4_O_2_ 433.1846; Found 433.1844; ${[\alpha ]}_{d}^{22}$ = +35.04° (*c* = 0.13, MeOH).

#### *N*-((*S*)-1-Cyclohexylethyl)-2-((*P*)-2-(trifluoromethyl)-1*H*-benzo­[*d*]­imidazol-1-yl)­benzamide **(** *P* **)-25**

Following the general procedure **A**, (*P*)-TBBA and (*S*)-(+)-1-cyclohexylethylamine (0.13 mmol, 1 equiv, 20 μL) were used, followed by purification by CC (hexane/EtOAc 3:1, column dimensions: 2 × 10 cm), yielding 44 mg (76%) of a colorless oil. ^**1**^ **H NMR** (400 MHz, CDCl_3_) δ (ppm) 7.99–7.86 (m, 2H), 7.70–7.61 (m, 2H), 7.46–7.35 (m, 3H), 7.15–7.08 (m, 1H), 5.16 (d, *J* = 8.7 Hz, 1H), 3.72–3.61 (m, 1H), 1.57–1.44 (m, 3H), 1.23–1.05 (m, 2H), 1.01–0.79 (m, 4H), 0.76 (d, *J* = 6.8 Hz, 3H), 0.55–0.29 (m, 2H); ^**13**^ **C­{** ^**1**^ **H} NMR** (101 MHz, CDCl_3_) δ (ppm) 164.7, 140.9 (q, *J* = 38.4 Hz), 137.5 (q, *J* = 1.2 Hz), 135.8, 131.6, 131.1, 130.9, 130.4, 129.5 (q, *J* = 0.8 Hz), 126.5, 124.6, 121.9, 119.1 (q, *J* = 271.7 Hz), 111.3, 50.0, 42.6, 28.6, 28.3, 26.2, 26.1, 26.1, 17.4; ^**19**^ **F NMR** (376 MHz, CDCl_3_) δ (ppm) −61.31 (s); **HRMS** (ESI) *m*/*z* [M + H]^+^ Calcd for C_23_H_25_F_3_N_3_O 416.1944; Found 416.1940; ${[\alpha ]}_{d}^{22}$ = −87.04° (*c* = 0.09, CHCl_3_).

#### *N*-((*S*)-1-Cyclohexylethyl)-2-((*M*)-2-(trifluoromethyl)-1*H*-benzo­[*d*]­imidazol-1-yl)­benzamide **(** *M* **)-25**

Following the general procedure **A**, (*M*)-TBBA and (*S*)-(+)-1-cyclohexylethylamine (0.13 mmol, 1 equiv, 20 μL) were used, followed by purification by CC (hexane/EtOAc 3:1, column dimensions: 2 × 10 cm), yielding 36 mg (62%) of a colorless oil. ^**1**^ **H NMR** (400 MHz, CDCl_3_) δ (ppm) 7.96–7.90 (m, 1H), 7.87–7.83 (m, 1H), 7.70–7.62 (m, 2H), 7.44–7.36 (m, 3H), 7.13–7.08 (m, 1H), 5.20 (d, *J* = 9.2 Hz, 1H), 3.64 (dp, *J* = 9.0, 6.7 Hz, 1H), 1.65–1.55 (m, 3H), 1.45–1.29 (m, 2H), 1.14–0.93 (m, 4H), 0.78–0.57 (m, 2H), 0.53 (d, *J* = 6.7 Hz, 3H); ^**13**^ **C­{** ^**1**^ **H} NMR** (101 MHz, CDCl_3_) δ (ppm) 164.7, 140.7, 140.6 (q, *J* = 38.2 Hz), 137.5 (q, *J* = 1.2 Hz), 135.9, 131.5, 131.3, 130.8, 129.9, 129.5 (app. d, *J* = 0.8 Hz), 126.4, 124.4, 121.7, 119.0 (q, *J* = 272.0 Hz), 111.4, 49.9, 42.7, 28.8, 28.5, 26.3, 26.1, 26.1, 17.0; ^**19**^ **F NMR** (376 MHz, CDCl_3_) δ (ppm) −61.22 (s); **HRMS** (ESI) *m*/*z* [M + H]^+^ Calcd for C_23_H_25_F_3_N_3_O 416.1944; Found 416.1944; ${[\alpha ]}_{d}^{22}$ = +102.08° (*c* = 0.08, CHCl_3_).

#### *N*-((*S*)-2-Methyl-1-phenylpropyl)-2-((*P*)-2-(Trifluoromethyl)-1*H*-benzo­[*d*]­imidazol-1-yl)­benzamide **(** *P* **)-26**

Following the general procedure **A**, (*P*)-TBBA and (*S*)-2-methyl-1-phenylpropan-1-amine hydrochloride (0.13 mmol, 1 equiv, 24 mg) were used, followed by purification by CC (CH_2_Cl_2_/MeOH 99:1, column dimensions: 2 × 10 cm), yielding 49 mg (86%) of a white foamy solid. ^**1**^ **H NMR** (400 MHz, CDCl_3_) δ 7.94 (d, *J* = 8.0 Hz, 1H), 7.89–7.81 (m, 1H), 7.69–7.60 (m, 2H), 7.44–7.33 (m, 3H), 7.26–7.16 (m, 3H), 7.12–7.07 (m, 1H), 6.89 (dd, *J* = 7.7, 1.7 Hz, 2H), 5.76 (d, *J* = 8.4 Hz, 1H), 4.56 (t, *J* = 8.0 Hz, 1H), 1.61 (dh, *J* = 13.2, 6.5 Hz, 1H), 0.51 (d, *J* = 6.7 Hz, 3H), 0.47 (d, *J* = 6.7 Hz, 3H); ^**13**^ **C­{** ^**1**^ **H} NMR** (101 MHz, CDCl_3_) δ 164.7, 140.8, 140.8 (q, *J* = 38.3 Hz), 140.7, 137.5, 135.4, 131.7, 131.3, 130.9, 130.2, 129.7, 128.6, 127.4, 126.7, 126.6, 124.6, 121.8, 119.0 (q, *J* = 272.1 Hz), 111.4, 59.8, 33.2, 19.3, 18.3; ^**19**^ **F NMR** (376 MHz, CDCl_3_) δ −61.25 (s); **HRMS** (ESI) *m*/*z* [M + H]^+^ Calcd for C_25_H_23_F_3_N_3_O 438.1788; Found 438.1783; ${[\alpha ]}_{d}^{22}$ = +2.78° (*c* = 0.08, MeOH).

#### *N*-((*S*)-2-Methyl-1-phenylpropyl)-2-((*M*)-2-(trifluoromethyl)-1*H*-benzo­[*d*]­imidazol-1-yl)­benzamide **(** *M* **)-26**

Following the general procedure **A**, (*M*)-TBBA and (*S*)-2-methyl-1-phenylpropan-1-amine hydrochloride (0.13 mmol, 1 equiv, 24 mg) were used, followed by purification by CC (CH_2_Cl_2_/MeOH 99:1, column dimensions: 2 × 10 cm), yielding 46 mg (81%) of a white foamy solid. ^**1**^ **H NMR** (400 MHz, CDCl_3_) δ 7.95–7.88 (m, 2H), 7.72–7.58 (m, 2H), 7.41 (td, *J* = 8.1, 1.2 Hz, 1H), 7.37–7.32 (m, 2H), 7.15–7.06 (m, 3H), 7.04 (dd, *J* = 8.1, 1.1 Hz, 1H), 6.63–6.58 (m, 2H), 5.66 (d, *J* = 8.2 Hz, 1H), 4.54 (t, *J* = 8.1 Hz, 1H), 1.68 (dh, *J* = 13.4, 6.7 Hz, 1H), 0.68 (d, *J* = 6.7 Hz, 3H), 0.56 (d, *J* = 6.7 Hz, 3H); ^**13**^ **C­{** ^**1**^ **H} NMR** (101 MHz, CDCl_3_) δ 164.6, 140.8 (q, *J* = 38.5 Hz), 140.8, 140.4, 137.4, 135.3, 131.8, 131.2, 131.0, 130.5, 129.6, 128.5, 127.2, 126.6, 126.5, 124.5, 122.0, 119.0 (q, *J* = 272.9 Hz), 111.2, 60.0, 33.3, 19.4, 18.6; ^**19**^ **F NMR** (376 MHz, CDCl_3_) δ −61.29 (s); **HRMS** (ESI) *m*/*z* [M + H]^+^ Calcd for C_25_H_23_F_3_N_3_O 438.1788; Found 438.1793; ${[\alpha ]}_{d}^{22}$ = −177.78° (*c* = 0.08, MeOH).

#### *N*-((*R*)-1-Phenylpropan-2-yl)-2-((*P*)-2-(trifluoromethyl)-1*H*-benzo­[*d*]­imidazol-1-yl)­benzamide **(** *P* **)-29**

Following the general procedure **A**, (*P*)-TBBA and (*R*)-1-phenylpropan-2-amine hydrogen sulfate **57** (0.13 mmol, 1 equiv, 18 mg) and triethylamine (0.14 mmol, 1.1 equiv, 20 μL) were used, followed by purification by CC (CH_2_Cl_2_/MeOH 99:1, column dimensions: 2 × 10 cm), yielding 40 mg (63%) of a white solid. ^**1**^ **H NMR** (400 MHz, CDCl_3_) δ 7.96–7.90 (m, 1H), 7.72–7.67 (m, 1H), 7.66–7.62 (m, 2H), 7.44–7.36 (m, 3H), 7.27–7.17 (m, 3H), 7.12–7.07 (m, 1H), 7.06–7.01 (m, 2H), 5.18 (d, *J* = 8.3 Hz, 1H), 4.08–3.95 (m, 1H), 2.55 (dd, *J* = 13.6, 6.4 Hz, 1H), 2.44 (dd, *J* = 13.6, 7.2 Hz, 1H), 0.57 (d, *J* = 6.6 Hz, 3H); ^**13**^ **C­{** ^**1**^ **H} NMR** (101 MHz, CDCl_3_) δ 164.7, 140.6, 140.5 (q, *J* = 38.3 Hz), 137.6, 137.5, 135.6, 131.5, 131.5, 130.8, 129.6, 129.5, 129.2, 128.5, 126.6, 126.4, 124.4, 121.6, 119.0 (q, *J* = 272.0 Hz), 111.5, 46.6, 42.2, 19.2; ^**19**^ **F NMR** (376 MHz, CDCl_3_) δ −61.16 (s); **HRMS** (ESI) *m*/*z* [M + H]^+^ Calcd for C_24_H_21_F_3_N_3_O 424.1631; Found 424.1626; ${[\alpha ]}_{d}^{22}$ = −73.67° (*c* = 0.30, CHCl_3_).

#### *N*-((*R*)-1-Phenylpropan-2-yl)-2-((*M*)-2-(trifluoromethyl)-1*H*-benzo­[*d*]­imidazol-1-yl)­benzamide **(** *M* **)-29**

Following the general procedure **A**, (*M*)-TBBA and (*R*)-1-phenylpropan-2-amine hydrogen sulfate **57** (0.13 mmol, 1 equiv, 30 mg) and triethylamine (0.14 mmol; 1.1 equiv; 20 μL) were used; with purification by CC (CH_2_Cl_2_/MeOH 99:1; column dimensions 2 × 10 cm), 39 mg (71%) of white solid was obtained. ^**1**^ **H NMR** (400 MHz, CDCl_3_) δ 7.98–7.93 (m, 1H), 7.81–7.75 (m, 1H), 7.68–7.64 (m, 2H), 7.45–7.36 (m, 3H), 7.24–7.15 (m, 3H), 7.08–7.04 (m, 1H), 6.96–6.90 (m, 2H), 5.13 (d, *J* = 7.5 Hz, 1H), 4.06–3.89 (m, 1H), 2.29 (dd, *J* = 13.5, 5.8 Hz, 1H), 2.11 (dd, *J* = 13.5, 7.9 Hz, 1H), 0.72 (d, *J* = 6.6 Hz, 3H); ^**13**^ **C­{** ^**1**^ **H} NMR** (101 MHz, CDCl_3_) δ 164.5, 141.1 (q, *J* = 38.5 Hz), 140.8, 137.6, 137.5, 135.5, 131.7, 131.5, 130.9, 129.9, 129.5, 129.2, 128.5, 126.6, 126.4, 124.5, 121.8, 118.9 (q, *J* = 272.0 Hz), 111.1, 46.8, 41.9, 19.3; ^**19**^ **F NMR** (376 MHz, CDCl_3_) δ −61.44 (s); **HRMS** (ESI) *m*/*z* [M + H]^+^ Calcd for C_24_H_21_F_3_N_3_O 424.1631; Found 424.1630; ${[\alpha ]}_{d}^{22}$ = +105.00° (*c* = 0.28, CHCl_3_).

#### *N*-((*S*)-1-Hydroxy-3-phenylpropan-2-yl)-2-((*P*)-2-(trifluoromethyl)-1*H*-benzo­[*d*]­imidazol-1-yl)­benzamide **(** *P* **)-30**

Following the general procedure **A**, (*P*)-TBBA and (*S*)-(−)-2-amino-3-phenyl-1-propanol (0.13 mmol, 1 equiv, 20 mg) were used, followed by purification by CC (CH_2_Cl_2_/MeOH 96:4, column dimensions: 2 × 10 cm), yielding 40 mg (70%) of a white foamy solid. ^**1**^ **H NMR** (400 MHz, CDCl_3_) δ 7.96–7.88 (m, 2H), 7.69–7.63 (m, 2H), 7.44 (ddd, *J* = 8.3, 7.2, 1.2 Hz, 1H), 7.41–7.33 (m, 2H), 7.21–7.11 (m, 3H), 7.05 (dt, *J* = 8.2, 1.0 Hz, 1H), 6.70–6.61 (m, 2H), 6.10 (d, *J* = 7.6 Hz, 1H), 4.94 (td, *J* = 9.0, 4.3 Hz, 1H), 3.44 (ddd, *J* = 11.5, 4.9, 4.1 Hz, 1H), 3.28 (ddd, *J* = 11.5, 10.2, 3.4 Hz, 1H), 2.25 (br. s, 1H), 1.81 (ddt, *J* = 14.5, 9.4, 4.7 Hz, 1H), 1.56 (ddt, *J* = 14.2, 9.2, 3.7 Hz, 1H); ^**13**^ **C­{** ^**1**^ **H} NMR** (101 MHz, CDCl_3_) δ 165.5, 141.0 (q, *J* = 38.3 Hz), 140.8, 140.4, 137.4, 134.7, 132.0, 131.5, 130.9, 130.4, 129.6, 128.9, 127.7, 126.6, 126.1, 124.6, 122.0, 118.9 (q, *J* = 271.9 Hz), 111.2, 58.8, 51.9, 38.3; ^**19**^ **F NMR** (376 MHz, CDCl_3_) δ −61.29 (s); **HRMS** (ESI) *m*/*z* [M + H]^+^ Calcd for C_24_H_21_F_3_N_3_O_2_ 440.1580; Found 440.1579; ${[\alpha ]}_{d}^{22}$ = −172.50° (*c* = 0.12, CHCl_3_).

#### *N*-((*S*)-1-Hydroxy-3-phenylpropan-2-yl)-2-((*M*)-2-(trifluoromethyl)-1*H*-benzo­[*d*]­imidazol-1-yl)­benzamide **(** *M* **)-30**

Following the general procedure **A**, (*M*)-TBBA and (*S*)-(−)-2-amino-3-phenyl-1-propanol (0.13 mmol, 1 equiv, 20 mg) were used, followed by purification by CC (CH_2_Cl_2_/MeOH 96:4, column dimensions: 2 × 10 cm), yielding 44 mg (77%) of a white foamy solid. ^**1**^ **H NMR** (400 MHz, CDCl_3_) δ 7.91 (dd, *J* = 7.0, 1.4 Hz, 1H), 7.88–7.83 (m, 1H), 7.68–7.63 (m, 2H), 7.44–7.36 (m, 3H), 7.26–7.19 (m, 3H), 7.11 (dd, *J* = 7.0, 1.5 Hz, 1H), 7.00–6.92 (m, 2H), 6.28 (d, *J* = 7.6 Hz, 1H), 4.95 (td, *J* = 8.3, 4.4 Hz, 1H), 3.29 (dt, *J* = 11.4, 4.5 Hz, 1H), 3.03 (td, *J* = 11.3, 3.4 Hz, 1H), 2.23 (br. s, 1H), 1.58 (ddt, *J* = 14.5, 9.4, 4.7 Hz, 1H), 1.42 (ddt, *J* = 14.3, 8.7, 3.7 Hz, 1H); ^**13**^ **C­{** ^**1**^ **H} NMR** (101 MHz, CDCl_3_) δ 165.5, 140.8 (q, *J* = 38.4 Hz), 140.7, 140.5, 137.4, 135.0, 131.9, 131.4, 130.9, 130.2, 129.5, 129.0, 127.8, 126.6, 126.3, 124.6, 121.8, 119.0 (q, *J* = 272.2 Hz), 111.4, 58.7, 51.9, 38.0; ^**19**^ **F NMR** (376 MHz, CDCl_3_) δ −61.18 (s); **HRMS** (ESI) *m*/*z* [M + H]^+^ Calcd for C_24_H_21_F_3_N_3_O_2_ 440.1580; Found 440.1575; ${[\alpha ]}_{d}^{22}$ = +52.86° (*c* = 0.14, CHCl_3_).

#### *N*-((*S*)-3-Hydroxy-1-phenylpropyl)-2-((*P*)-2-(trifluoromethyl)-1*H*-benzo­[*d*]­imidazol-1-yl)­benzamide **(** *P* **)-32**

Following the general procedure **A**, (*P*)-TBBA and (*S*)-beta-phenylalaninol (0.13 mmol, 1 equiv, 20 mg) were used, followed by purification by CC (CH_2_Cl_2_/MeOH 96:4, column dimensions: 2 × 10 cm), yielding 46 mg (80%) of a white solid. ^**1**^ **H NMR** (400 MHz, CDCl_3_) δ 7.97–7.89 (m, 1H), 7.74–7.69 (m, 1H), 7.68–7.62 (m, 2H), 7.46–7.35 (m, 3H), 7.27–7.16 (m, 3H), 7.13–7.04 (m, 3H), 5.73 (d, *J* = 8.2 Hz, 1H), 3.95 (ddq, *J* = 11.6, 7.9, 3.7 Hz, 1H), 3.14 (dt, *J* = 10.8, 3.9 Hz, 1H), 2.91 (ddd, *J* = 10.8, 5.4, 3.7 Hz, 1H), 2.68–2.52 (m, 2H), 1.42 (t, *J* = 4.9 Hz, 1H); ^**13**^ **C­{** ^**1**^ **H} NMR** (101 MHz, CDCl_3_) δ 165.4, 140.8 (q, *J* = 38.2 Hz), 140.6, 137.5, 135.2, 131.7, 131.5, 130.8, 129.7, 129.4, 129.2, 128.7, 126.7, 126.4, 124.5, 121.5, 119.0 (q, *J* = 271.9 Hz), 111.5, 62.5, 52.4, 36.7; ^**19**^ **F NMR** (376 MHz, CDCl_3_) δ −61.21 (s); **HRMS** (ESI) *m*/*z* [M + H]^+^ Calcd for C_24_H_21_F_3_N_3_O_2_ 440.1580; Found 440.1572; ${[\alpha ]}_{d}^{22}$ = −98.61° (*c* = 0.08, MeOH).

#### *N*-((*S*)-3-Hydroxy-1-phenylpropyl)-2-((*M*)-2-(trifluoromethyl)-1*H*-benzo­[*d*]­imidazol-1-yl)­benzamide **(** *M* **)-32**

Following the general procedure **A**, (*M*)-TBBA and (*S*)-beta-phenylalaninol (0.13 mmol, 1 equiv, 20 mg) were used, followed by purification by CC (CH_2_Cl_2_/MeOH 96:4, column dimensions: 2 × 10 cm), yielding 44 mg (77%) of a white solid. ^**1**^ **H NMR** (400 MHz, CDCl_3_) δ 8.0–7.9 (m, 1H), 7.8–7.7 (m, 1H), 7.7–7.6 (m, 2H), 7.5–7.4 (m, 1H), 7.4–7.4 (m, 2H), 7.3–7.1 (m, 3H), 7.1–7.1 (m, 1H), 7.0–7.0 (m, 2H), 5.7 (d, *J* = 7.7 Hz, 1H), 3.9 (ddt, *J* = 11.6, 7.8, 3.9 Hz, 1H), 3.3 (dd, *J* = 10.9, 3.9 Hz, 1H), 3.2 (dd, *J* = 11.0, 3.6 Hz, 1H), 2.4–2.3 (m, 2H), 1.5 (br. s, 1H); ^**13**^ **C­{** ^**1**^ **H} NMR** (101 MHz, CDCl_3_) δ 165.4, 141.1 (q, *J* = 38.5 Hz), 140.8, 137.4, 135.1, 131.8, 131.7, 130.8, 129.7, 129.5, 129.2, 128.6, 126.7, 126.3, 124.5, 121.7, 118.9 (q, *J* = 272.1 Hz), 111.2, 62.3, 52.6, 36.2; ^**19**^ **F NMR** (376 MHz, CDCl_3_) δ −61.45 (s); **HRMS** (ESI) *m*/*z* [M + H]^+^ Calcd for C_24_H_21_F_3_N_3_O_2_ 440.1580; Found 440.1578; ${[\alpha ]}_{d}^{22}$ = +23.08° (*c* = 0.13, MeOH).

#### *N*-((*R*)-1-Hydroxybutan-2-yl)-2-((*P*)-2-(trifluoromethyl)-1*H*-benzo­[*d*]­imidazol-1-yl)­benzamide **(** *P* **)-34**

Following the general procedure **A**, (*P*)-TBBA and (*R*)-2-aminobutan-1-ol (0.13 mmol, 1 equiv, 11.5 mg) were used, followed by purification by CC (CH_2_Cl_2_/MeOH 96:4, column dimensions: 2 × 10 cm), yielding 40 mg (82%) of a white solid. ^**1**^ **H NMR** (400 MHz, CDCl_3_) δ 7.97–7.91 (m, 1H), 7.89–7.83 (m, 1H), 7.70–7.64 (m, 2H), 7.47–7.37 (m, 3H), 7.17–7.06 (m, 1H), 5.62 (d, *J* = 7.7 Hz, 1H), 3.62 (ddq, *J* = 11.6, 8.0, 4.1 Hz, 1H), 3.41–3.25 (m, 2H), 1.72 (t, *J* = 5.3 Hz, 1H), 1.23–1.12 (m, 1H), 1.09–0.92 (m, 1H), 0.59 (t, *J* = 7.5 Hz, 3H); ^**13**^ **C­{** ^**1**^ **H} NMR** (101 MHz, CDCl_3_) δ 165.8, 141.0 (q, *J* = 38.4 Hz), 140.8, 137.5, 135.4, 131.8, 131.6, 130.9, 129.9, 129.6, 126.4, 124.5, 121.8, 119.0 (q, *J* = 272.0 Hz), 111.3, 64.0, 53.2, 23.7, 10.1; ^**19**^ **F NMR** (376 MHz, CDCl_3_) δ −61.39 (s); **HRMS** (ESI) *m*/*z* [M + H]^+^ Calcd for C_19_H_19_F_3_N_3_O_2_ 378.1424; Found 378.1422; ${[\alpha ]}_{d}^{22}$ = −112.14° (*c* = 0.14, MeOH).

#### *N*-((*R*)-1-Hydroxybutan-2-yl)-2-((*M*)-2-(trifluoromethyl)-1*H*-benzo­[*d*]­imidazol-1-yl)­benzamide **(** *M* **)-34**

Following the general procedure **A**, (*M*)-TBBA and (*R*)-2-aminobutan-1-ol (0.13 mmol, 1 equiv, 11.5 mg) were used, followed by purification by CC (CH_2_Cl_2_/MeOH 96:4, column dimensions: 2 × 10 cm), yielding 39 mg (80%) of a white solid. ^**1**^ **H NMR** (400 MHz, CDCl_3_) δ 7.96–7.91 (m, 1H), 7.89–7.84 (m, 1H), 7.71–7.65 (m, 2H), 7.46–7.37 (m, 3H), 7.15–7.09 (m, 1H), 5.61 (d, *J* = 7.9 Hz, 1H), 3.74–3.50 (m, 1H), 3.18 (dt, *J* = 10.8, 3.9 Hz, 1H), 2.99 (dt, *J* = 10.7, 4.4 Hz, 1H), 1.55 (t, *J* = 4.8 Hz, 1H), 1.37–1.26 (m, 1H), 1.29–1.10 (m, 1H), 0.71 (t, *J* = 7.5 Hz, 3H); ^**13**^ **C­{** ^**1**^ **H} NMR** (101 MHz, CDCl_3_) δ 165.7, 140.9 (q, *J* = 38.4 Hz), 140.6, 137.5, 135.4, 131.8, 131.4, 130.9, 129.9, 129.5, 126.5, 124.6, 121.6, 119.0 (q, *J* = 271.9 Hz), 111.4, 63.8 (d, *J* = 3.9 Hz), 52.9, 23.9, 10.3; ^**19**^ **F NMR** (376 MHz, CDCl_3_) δ −61.31 (s); **HRMS** (ESI) *m*/*z* [M + H]^+^ Calcd for C_19_H_19_F_3_N_3_O_2_ 378.1424; Found 378.1423; ${[\alpha ]}_{d}^{22}$ = +137.69° (*c* = 0.13, MeOH).

#### *N*-((*S*)-1-Hydroxypropan-2-yl)-2-((*P*)-2-(trifluoromethyl)-1*H*-benzo­[*d*]­imidazol-1-yl)­benzamide **(** *P* **)-35**

Following the general procedure **A**, (*P*)-TBBA and l-alaninol (0.13 mmol, 1 equiv, 10 μL) were used, followed by purification by CC (hexane/EtOAc 1:2, column dimensions: 2 × 10 cm), yielding 24 mg (51%) of a brown oil. ^**1**^ **H NMR** (400 MHz, CDCl_3_) δ (ppm) 7.97–7.91 (m, 1H), 7.90–7.84 (m, 1H), 7.72–7.63 (m, 2H), 7.48–7.37 (m, 3H), 7.15–7.09 (m, 1H), 5.56 (d, *J* = 7.2 Hz, 1H), 3.80 (dtt, *J* = 11.2, 6.9, 3.6 Hz, 1H), 3.08 (dd, *J* = 10.8, 4.6 Hz, 1H), 3.03 (dd, *J* = 10.8, 3.8 Hz, 1H), 0.84 (d, *J* = 6.8 Hz, 3H); ^**13**^ **C­{** ^**1**^ **H} NMR** (126 MHz, CDCl_3_) δ (ppm) 165.4, 141.0 (q, *J* = 38.5 Hz), 140.7, 137.4, 135.3, 131.8, 131.4, 131.0, 130.1, 129.4, 126.6, 124.6, 121.7, 119.0 (q, *J* = 272.1 Hz), 111.4, 65.9, 47.5, 16.6; ^**19**^ **F NMR** (376 MHz, CDCl_3_) δ (ppm) −61.36 (s); **HRMS** (ESI) *m*/*z* [M + H]^+^ Calcd for C_18_H_17_F_3_N_3_O_2_ 364.1267; Found 364.1265; ${[\alpha ]}_{d}^{22}$ = −37.03° (*c* = 0.09 CHCl_3_).

#### *N*-((*S*)-1-Hydroxypropan-2-yl)-2-((*M*)-2-(trifluoromethyl)-1*H*-benzo­[*d*]­imidazol-1-yl)­benzamide **(** *M* **)-35**

Following the general procedure **A**, (*M*)-TBBA and l-alaninol (0.13 mmol, 1 equiv, 10 μL) were used, followed by purification by CC (hexane/EtOAc 1:2, column dimensions: 2 × 10 cm), yielding 26 mg (55%) of a brown oil. ^**1**^ **H NMR** (400 MHz, CDCl_3_) δ (ppm) 7.97–7.90 (m, 1H), 7.89–7.82 (m, 1H), 7.70–7.64 (m, 2H), 7.50–7.35 (m, 3H), 7.14–7.07 (m, 1H), 5.61 (d, *J* = 6.9 Hz, 1H), 3.84–3.73 (m, 1H), 3.33 (dd, *J* = 10.9, 3.8 Hz, 1H), 3.24 (dd, *J* = 10.9, 4.9 Hz, 1H), 0.65 (d, *J* = 6.8 Hz, 3H); ^**13**^ **C­{** ^**1**^ **H} NMR** (101 MHz, CDCl_3_) δ (ppm) 165.5, 140.9 (q, *J* = 38.4 Hz), 140.7, 137.5 (q, *J* = 1.0 Hz), 135.3, 131.8, 131.6, 130.9, 129.9, 129.5 (app. d, *J* = 0.8 Hz), 126.4, 124.6, 121.7, 119.0 (q, *J* = 272.0 Hz), 111.4, 65.9, 47.6, 16.2; ^**19**^ **F NMR** (376 MHz, CDCl_3_) δ (ppm) −61.33 (s); **HRMS** (ESI) *m*/*z* [M + H]^+^ Calcd for C_18_H_17_F_3_N_3_O_2_ 364.1267; Found 364.1266; ${[\alpha ]}_{d}^{22}$ = +147.92° (*c* = 0.08, CHCl_3_).

#### *N*-((*R*)-1-Methoxypropan-2-yl)-2-((*P*)-2-(trifluoromethyl)-1*H*-benzo­[*d*]­imidazol-1-yl)­benzamide **(** *P* **)-36**

Following the general procedure **A**, (*P*)-TBBA and (*R*)-1-methoxypropan-2-amine **54** (0.13 mmol, 1 equiv, 12 mg) were used, followed by purification by CC (hexane/EtOAc 3:1, column dimensions: 2 × 10 cm), yielding 28 mg (57%) of a white oil. ^**1**^ **H NMR** (400 MHz, CDCl_3_) δ (ppm) 7.96–7.92 (m, 1H), 7.94–7.83 (m, 1H), 7.70–7.63 (m, 2H), 7.45–7.36 (m, 3H), 7.15–7.07 (m, 1H), 5.68 (d, *J* = 7.2 Hz, 1H), 3.93–3.83 (m, 1H), 3.17 (s, 3H), 3.06 (dd, *J* = 9.4, 3.9 Hz, 1H), 2.99 (dd, *J* = 9.4, 4.0 Hz, 1H), 0.60 (d, *J* = 6.7 Hz, 3H); ^**13**^ **C­{** ^**1**^ **H} NMR** (101 MHz, CDCl_3_) δ (ppm) 164.7, 140.8, 140.7 (q, *J* = 38.3 Hz), 137.5 (q, *J* = 1.2 Hz), 135.4, 131.7, 131.6, 130.8, 130.1, 129.4 (app. d, *J* = 0.9 Hz), 126.4, 124.4, 121.7, 119.0 (q, *J* = 271.6 Hz), 111.4, 74.8, 59.0, 45.4, 16.7; ^**19**^ **F NMR** (376 MHz, CDCl_3_) δ (ppm) −61.38 (s); **HRMS** (ESI) *m*/*z* [M + H]^+^ Calcd for C_19_H_19_F_3_N_3_O_2_ 378.1424; Found 378.1421; ${[\alpha ]}_{d}^{22}$ = −116.67° (*c* = 0.08, CHCl_3_).

#### *N*-((*R*)-1-Methoxypropan-2-yl)-2-((*M*)-2-(trifluoromethyl)-1*H*-benzo­[*d*]­imidazol-1-yl)­benzamide **(** *M* **)-36**

Following the general procedure **A**, (*M*)-TBBA and (*R*)-1-methoxypropan-2-amine **54** (0.13 mmol, 1 equiv, 12 mg) were used, followed by purification by CC (hexane/EtOAc 3:1, column dimensions: 2 × 10 cm), yielding 26 mg (53%) of a white oil. ^**1**^ **H NMR** (400 MHz, CDCl_3_) δ (ppm) 7.98–7.90 (m, 1H), 7.90–7.83 (m, 1H), 7.71–7.61 (m, 2H), 7.46–7.34 (m, 3H), 7.16–7.07 (m, 1H), 5.70 (d, *J* = 6.9 Hz, 1H), 3.91–3.80 (m, 1H), 3.10 (s, 3H), 2.87 (dd, *J* = 9.2, 3.7 Hz, 1H), 2.68 (dd, *J* = 9.2, 4.0 Hz, 1H), 0.92 (d, *J* = 6.8 Hz, 3H); ^**13**^ **C­{** ^**1**^ **H} NMR** (101 MHz, CDCl_3_) δ (ppm) 165.5, 140.9 (q, *J* = 38.4 Hz), 140.7, 137.5 (q, *J* = 1.0 Hz), 135.3, 131.8, 131.6, 130.9, 129.9, 129.5 (app. d, *J* = 0.8 Hz), 126.4, 124.6, 121.7, 119.0 (q, *J* = 272.0 Hz), 111.4, 74.8, 65.9, 47.6, 16.2; ^**19**^ **F NMR** (376 MHz, CDCl_3_) δ (ppm) −61.40 (s); **HRMS** (ESI) *m*/*z* [M + H]^+^ Calcd for C_19_H_19_F_3_N_3_O_2_ 378.1424; Found 378.1421; ${[\alpha ]}_{d}^{22}$ = +95.83° (*c* = 0.08, CHCl_3_).

#### *N*-((1*R*,2*R*)-2-Aminocyclohexyl)-2-((*P*)-2-(trifluoromethyl)-1*H*-benzo­[*d*]­imidazol-1-yl)­benzamide **(** *P* **)-37**

To **(** *P* **)-38** (0.06 mmol, 1 equiv, 30 mg) was added CH_2_Cl_2_:TFA (10:2, 0.5 mL). The reaction mixture was stirred at room temperature for 3 h, and then, the pH was adjusted to 12 with 2 M NaOH. The product was extracted into CH_2_Cl_2_ (3 × 10 mL). The organic phases were combined, dried with MgSO_4_, and evaporated under reduced pressure, yielding 20 mg (83%) of a white solid. ^**1**^ **H NMR** (400 MHz, CDCl_3_) δ 7.9–7.9 (m, 1H), 7.9–7.8 (m, 1H), 7.7–7.6 (m, 2H), 7.5–7.4 (m, 1H), 7.4–7.4 (m, 2H), 7.1–7.1 (m, 1H), 5.5 (d, *J* = 7.8 Hz, 1H), 3.3–3.1 (m, 1H), 2.0 (td, *J* = 10.4, 4.0 Hz, 1H), 1.8–1.7 (m, 1H), 1.6–1.5 (m, 1H), 1.5–1.4 (m, 2H), 1.2 (d, *J* = 5.3 Hz, 2H), 1.1–0.9 (m, 3H), 0.5 (qd, *J* = 12.8, 12.0, 3.1 Hz, 1H); ^**13**^ **C­{** ^**1**^ **H} NMR** (101 MHz, CDCl_3_) δ 165.8, 140.8 (q, *J* = 38.4 Hz), 140.7, 137.5, 135.6, 131.7, 130.8, 129.6, 129.6, 126.3, 124.4, 121.7, 119.0 (q, *J* = 271.9 Hz), 111.4, 56.5, 55.2, 35.8, 31.4, 24.9, 24.7; ^**19**^ **F NMR** (376 MHz, CDCl_3_) δ −61.73 (s); **HRMS** (ESI) *m*/*z* [M + H]^+^ Calcd for C_21_H_22_F_3_N_4_O 403.1740; Found 403.1740; ${[\alpha ]}_{d}^{22}$ = −155.46° (*c* = 0.33, CHCl_3_).

#### *N*-((1*R*,2*R*)-2-Aminocyclohexyl)-2-((*M*)-2-(trifluoromethyl)-1*H*-benzo­[*d*]­imidazol-1-yl)­benzamide **(** *M* **)-37**

Following the same procedure as for **(** *P* **)-37**, the reaction using **(** *M* **)-38** (0.06 mmol, 1 equiv, 30 mg) yielded 19 mg (79%) of a white solid. ^**1**^ **H NMR** (400 MHz, CDCl_3_) δ 8.0–7.9 (m, 1H), 7.9–7.8 (m, 1H), 7.7–7.6 (m, 2H), 7.4–7.4 (m, 3H), 7.1–7.1 (m, 1H), 5.5 (d, *J* = 6.1 Hz, 1H), 3.3–3.1 (m, 1H), 1.8 (td, *J* = 10.4, 4.0 Hz, 1H), 1.8–1.7 (m, 2H), 1.6–1.5 (m, 2H), 1.2 (br. s, 2H), 1.1–0.9 (m, 3H), 0.6 (qd, *J* = 12.5, 3.7 Hz, 1H); ^**13**^ **C­{** ^**1**^ **H} NMR** (101 MHz, CDCl_3_) δ 165.6, 141.0 (q, *J* = 38.4 Hz), 140.7, 137.5, 135.6, 131.7, 131.3, 130.9, 130.1, 129.5, 126.5, 124.6, 121.8, 118.9 (q, *J* = 272.1 Hz), 111.2, 56.4, 55.0, 35.6, 31.7, 24.8, 24.7; ^**19**^ **F NMR** (376 MHz, CDCl_3_) δ −61.73 (s); **HRMS** (ESI) *m*/*z* [M + H]^+^ Calcd for C_21_H_22_F_3_N_4_O 403.1740; Found 403.1738; ${[\alpha ]}_{d}^{22}$ = +45.93° (*c* = 0.35, CHCl_3_).

#### *tert*-Butyl ((1*R*,2*R*)-2-(2-((*P*)-2-(trifluoromethyl)-1*H*-benzo­[*d*]­imidazol-1-yl)­benzamido)­cyclohexyl)­carbamate **(** *P* **)-38**

Following the general procedure **A**, (*P*)-TBBA and **63** (0.13 mmol, 1 equiv, 28 mg) were used, followed by purification by CC (CH_2_Cl_2_/MeOH 99:1, column dimensions: 2 × 10 cm), yielding 40 mg (62%) of a white solid. ^**1**^ **H NMR** (400 MHz, CDCl_3_) δ 7.94–7.87 (m, 1H), 7.78 (dd, *J* = 7.2, 2.0 Hz, 1H), 7.65–7.57 (m, 2H), 7.44–7.40 (m, 1H), 7.40–7.34 (m, 2H), 7.11–7.06 (m, 1H), 6.57 (d, *J* = 6.7 Hz, 1H), 4.52 (d, *J* = 7.7 Hz, 1H), 3.42–3.24 (m, 2H), 1.95–1.86 (m, 1H), 1.71–1.64 (m, 1H), 1.61–1.50 (m, 2H), 1.42 (s, 9H), 1.24–1.03 (m, 3H), 0.86–0.73 (m, 1H); ^**13**^ **C­{** ^**1**^ **H} NMR** (101 MHz, CDCl_3_) δ 165.2, 157.2, 141.5 (q, *J* = 38.5 Hz), 140.8, 137.6, 135.0, 132.7, 131.5, 130.4, 129.8, 128.8, 125.8, 123.9, 121.6, 119.0 (q, *J* = 272.4 Hz), 111.0, 80.0, 56.1, 53.2, 32.5, 31.6, 28.4, 25.1, 24.2; ^**19**^ **F NMR** (376 MHz, CDCl_3_) δ −61.36 (s); **HRMS** (ESI) *m*/*z* [M + H]^+^ Calcd for C_26_H_30_F_3_N_4_O_3_ 503.2265; Found 503.2263; ${[\alpha ]}_{d}^{22}$ = −49.09° (*c* = 0.11, CHCl_3_).

#### *tert*-Butyl ((1*R*,2*R*)-2-(2-((*M*)-2-(trifluoromethyl)-1*H*-benzo­[*d*]­imidazol-1-yl)­benzamido)­cyclohexyl)­carbamate **(** *M* **)-38**

Following the general procedure **A**, (*M*)-TBBA and **63** (0.13 mmol, 1 equiv, 28 mg) were used, followed by purification by CC (CH_2_Cl_2_/MeOH 99:1, column dimensions: 2 × 10 cm), yielding 34 mg (52%) of a white solid. ^**1**^ **H NMR** (400 MHz, CDCl_3_) δ 7.96–7.88 (m, 1H), 7.77 (dd, *J* = 7.5, 1.7 Hz, 1H), 7.64 (td, *J* = 7.6, 1.8 Hz, 1H), 7.59 (td, *J* = 7.5, 1.5 Hz, 1H), 7.45 (dd, *J* = 7.8, 1.1 Hz, 1H), 7.41–7.33 (m, 2H), 7.07–7.00 (m, 1H), 6.68 (d, *J* = 6.9 Hz, 1H), 4.57 (d, *J* = 8.3 Hz, 1H), 3.35 (tdd, *J* = 11.0, 7.1, 3.8 Hz, 1H), 3.29–3.19 (m, 1H), 1.93–1.84 (m, 1H), 1.72–1.63 (m, 1H), 1.54–1.43 (m, 2H), 1.40 (s, 9H), 1.19–1.02 (m, 3H), 0.62 (qd, *J* = 13.2, 12.7, 3.7 Hz, 1H); ^**13**^ **C­{** ^**1**^ **H} NMR** (101 MHz, CDCl_3_) δ 165.5, 157.0, 140.6 (q, *J* = 38.3 Hz), 140.6, 138.1, 135.3, 132.4, 131.4, 130.5, 129.8, 128.8, 125.9, 124.0, 121.5, 119.0 (q, *J* = 272.0 Hz), 111.7, 79.9, 56.0, 53.3, 32.6, 31.7, 28.5, 25.0, 24.3; ^**19**^ **F NMR** (376 MHz, CDCl_3_) δ −61.75 (s); **HRMS** (ESI) *m*/*z* [M + H]^+^ Calcd for C_26_H_30_F_3_N_4_O_3_503.2265; Found 503.2261; ${[\alpha ]}_{d}^{22}$ = +118.75° (*c* = 0.16, CHCl_3_).

#### *tert*-Butyl (*R*)-3-(2-((*P*)-2-(trifluoromethyl)-1*H*-benzo­[*d*]­imidazol-1-yl)­benzamido)­piperidine-1-carboxylate **(** *P* **)-39**

Following the general procedure **A**, (*P*)-TBBA and *tert*-butyl (*R*)-3-aminopyrrolidine-1-carboxylate (0.13 mmol, 1 equiv, 24 mg) were used, followed by purification by CC (CH_2_Cl_2_/MeOH 99:1, column dimensions: 2 × 10 cm), yielding 48 mg (78%) of a white solid. ^**1**^ **H NMR** (400 MHz, CDCl_3_) δ 7.99–7.86 (m, 1H), 7.85–7.78 (m, 1H), 7.70–7.64 (m, 2H), 7.45–7.37 (m, 3H), 7.16–7.08 (m, 1H), 5.60 (br. s, 1H), 3.75 (br. s, 1H), 3.54–2.62 (m, 4H), 1.42 (s, 9H), 1.36–1.18 (m, 3H), 0.88 (br. s, 1H); ^**13**^ **C­{** ^**1**^ **H} NMR** (101 MHz, CDCl_3_) δ 164.9, 155.1, 140.6, 140.5 (q, *J* = 38.2, 37.6 Hz), 137.6, 135.3, 131.7, 131.6, 130.9, 129.6, 126.4, 124.5, 121.6, 119.1 (q, *J* = 272.1 Hz), 111.6, 80.1, 48.2, 45.4, 44.1, 29.3, 28.4, 22.1; ^**19**^ **F NMR** (376 MHz, CDCl_3_) δ −61.19 (s); **HRMS** (ESI) *m*/*z* [M + H]^+^ Calcd for C_25_H_28_F_3_N_4_O_3_ 489.2108; Found 489.2096; ${[\alpha ]}_{d}^{22}$ = −100.83° (*c* = 0.12, CHCl_3_).

#### *tert*-Butyl (*R*)-3-(2-((*M*)-2-(trifluoromethyl)-1*H*-benzo­[*d*]­imidazol-1-yl)­benzamido)­piperidine-1-carboxylate **(** *M* **)-39**

Following the general procedure **A**, (*M*)-TBBA and *tert*-butyl (*R*)-3-aminopyrrolidine-1-carboxylate (0.13 mmol, 1 equiv, 24 mg) were used, followed by purification by CC (CH_2_Cl_2_/MeOH 99:1, column dimensions: 2 × 10 cm), yielding 50 mg (81%) of a white solid. ^**1**^ **H NMR** (400 MHz, CDCl_3_) δ 7.98–7.91 (m, 1H), 7.86–7.81 (m, 1H), 7.70–7.65 (m, 2H), 7.51–7.40 (m, 1H), 7.43–7.37 (m, 2H), 7.10–7.04 (m, 1H), 5.46 (br. s, 1H), 3.77–3.68 (m, 1H), 3.30–3.17 (m, 2H), 3.16–3.06 (m, 1H), 2.76 (br. s, 1H), 1.55–1.47 (m, 1H), 1.40 (s, 9H), 1.33–1.26 (m, 1H), 1.22–1.13 (m, 1H), 1.06–0.96 (m, 1H); ^**13**^ **C­{** ^**1**^ **H} NMR** (101 MHz, CDCl_3_) δ 164.7, 155.1, 141.1 (q, *J* = 39.8 Hz), 140.8, 137.5, 135.2, 131.9, 131.8, 130.9, 129.7, 126.4, 124.5, 121.9, 118.9 (q, *J* = 271.9 Hz), 111.0, 80.0, 48.1, 45.6, 43.9, 43.8, 29.3, 28.4, 22.3; ^**19**^ **F NMR** (376 MHz, CDCl_3_) δ −61.56 (s); **HRMS** (ESI) *m*/*z* [M + H]^+^ Calcd for C_25_H_28_F_3_N_4_O_3_ 489.2108; Found 489.2107; ${[\alpha ]}_{d}^{22}$ = +106.67° (*c* = 0.12, CHCl_3_).

#### *N*-((1*R*,2*S*)-2-Hydroxy-2,3-dihydro-1*H*-inden-1-yl)-2-((*P*)-2-(trifluoromethyl)-1*H*-benzo­[*d*]­imidazol-1-yl)­benzamide **(** *P* **)-40**

Following the general procedure **A**, (*P*)-TBBA and (1*R*,2*S*)-1-amino-2,3-dihydro-1*H*-inden-2-ol (0.13 mmol, 1 equiv, 20 mg) were used, followed by purification by CC (hexane/EtOAc 2:1, column dimensions: 2 × 10 cm), yielding 36 mg (63%) of a yellow solid. ^**1**^ **H NMR** (400 MHz, CDCl_3_) δ (ppm) 7.97–7.87 (m, 2H), 7.72–7.66 (m, 2H), 7.48–7.36 (m, 3H), 7.18–7.10 (m, 3H), 7.05–6.98 (m, 1H), 6.54 (d, *J* = 7.5 Hz, 1H), 6.28 (d, *J* = 7.9 Hz, 1H), 5.17 (dd, *J* = 8.0, 5.1 Hz, 1H), 4.37 (td, *J* = 5.2, 2.2 Hz, 1H), 3.03 (dd, *J* = 16.6, 5.2 Hz, 1H), 2.74 (dd, *J* = 16.6, 2.1 Hz, 1H); ^**13**^ **C­{** ^**1**^ **H} NMR** (101 MHz, CDCl_3_) δ (ppm) 166.1, 141.0 (q, *J* = 35.1 Hz), 140.9, 139.8, 139.5, 137.6, 135.1, 131.9, 131.8, 130.9, 129.9, 129.7, 128.3, 127.4, 126.3, 125.3, 124.5, 124.4, 119.0 (q, *J* = 271.6 Hz), 111.4, 73.4, 57.9, 39.8; ^**19**^ **F NMR** (471 MHz, CDCl_3_) δ (ppm) −61.30 (s); ^**19**^ **F NMR** (376 MHz, DMSO-*d*_6_) δ (ppm) −59.44 (s); **HRMS** (ESI) *m*/*z* [M + H]^+^ Calcd for C_24_H_19_F_3_N_3_O_2_ 438.1424; Found 438.1416; ${[\alpha ]}_{d}^{22}$ = +70.37° (*c* = 0.09, CHCl_3_).

#### *N*-((1*R*,2*S*)-2-Hydroxy-2,3-dihydro-1*H*-inden-1-yl)-2-((*M*)-2-(trifluoromethyl)-1*H*-benzo­[*d*]­imidazol-1-yl)­benzamide **(** *M* **)-40**

Following the general procedure **A**, (*M*)-TBBA and (1*R*,2*S*)-1-amino-2,3-dihydro-1*H*-inden-2-ol (0.13 mmol, 1 equiv, 20 mg) were used, followed by purification by CC (hexane/EtOAc 2:1, column dimensions: 2 × 10 cm), yielding 40 mg (69%) of a yellow solid. ^**1**^ **H NMR** (400 MHz, CDCl_3_) δ (ppm) 7.97–7.90 (m, 2H), 7.73–7.66 (m, 2H), 7.49–7.35 (m, 3H), 7.20–7.05 (m, 4H), 6.79 (d, *J* = 7.5 Hz, 1H), 6.37 (d, *J* = 8.3 Hz, 1H), 5.10 (dd, *J* = 8.3, 5.1 Hz, 1H), 4.10 (td, *J* = 5.0, 1.8 Hz, 1H), 2.98 (dd, *J* = 16.6, 5.0 Hz, 1H), 2.72 (dd, *J* = 16.5, 1.5 Hz, 1H); ^**13**^ **C­{** ^**1**^ **H} NMR** (101 MHz, CDCl_3_) δ (ppm) 166.0, 141.3 (q, *J* = 38.2 Hz), 140.8, 140.2, 139.5, 137.4, 135.0, 131.9, 130.9, 129.9, 129.6, 128.3, 127.4, 126.4, 125.3, 124.6, 124.4, 120.1 (q, *J* = 271.9 Hz), 111.4, 73.3, 57.6, 40.1; ^**19**^ **F NMR** (376 MHz, CDCl_3_) δ (ppm) −61.39 (s); ^**19**^ **F NMR** (376 MHz, DMSO-*d*_6_) δ (ppm) −59.50 (s); **HRMS** (ESI) *m*/*z* [M + H]^+^ Calcd for C_24_H_19_F_3_N_3_O_2_ 438.1424; Found 438.1423; ${[\alpha ]}_{d}^{22}$ = −116.67° (*c* = 0.08, CHCl_3_).

#### (1*R*,2*S*)-1-(2-((*P*)-2-(Trifluoromethyl)-1*H*-benzo­[*d*]­imidazol-1-yl)­benzamido)-2,3-dihydro-1*H*-inden-2-yl Acetate **(** *P* **)-41**

Following the general procedure **C**, **(** *P* **)–40** (0.13 mmol, 1 equiv, 57 mg) was used, followed by purification by CC (CH_2_Cl_2_/MeOH 99:1, column dimensions: 2 × 10 cm), yielding 48 mg (77%) of a white solid. ^**1**^ **H NMR** (400 MHz, CDCl_3_) δ 7.96–7.89 (m, 2H), 7.74–7.67 (m, 2H), 7.48–7.39 (m, 3H), 7.17–7.07 (m, 3H), 6.94 (t, *J* = 7.3 Hz, 1H), 6.05 (d, *J* = 7.6 Hz, 1H), 5.93 (d, *J* = 8.5 Hz, 1H), 5.46 (dd, *J* = 8.6, 5.5 Hz, 1H), 5.39 (td, *J* = 5.4, 2.2 Hz, 1H), 3.07 (dd, *J* = 17.1, 5.3 Hz, 1H), 2.81 (dd, *J* = 17.1, 2.1 Hz, 1H), 1.96 (s, 3H); ^**13**^ **C­{** ^**1**^ **H} NMR** (101 MHz, CDCl_3_) δ 170.3, 165.6, 140.7, 140.3 (q, *J* = 38.2 Hz), 139.4, 138.9, 137.5, 134.9, 132.0, 131.3, 131.0, 130.2, 129.7, 128.4, 127.4, 126.6, 124.9, 124.6, 123.4, 121.8, 119.1 (q, *J* = 271.9 Hz), 111.5, 75.6, 55.5, 37.3, 20.9; ^**19**^ **F NMR** (376 MHz, CDCl_3_) δ −60.86 (s); ^**19**^ **F NMR** (376 MHz, DMSO-*d*_6_) δ (ppm) −59.45 (s); **HRMS** (ESI) *m*/*z* [M + H]^+^ Calcd for C_26_H_21_F_3_N_3_O_3_ 480.1530; Found 480.1525; ${[\alpha ]}_{d}^{22}$ = −12.86° (*c* = 0.14, CHCl_3_).

#### (1*R*,2*S*)-1-(2-((*M*)-2-(Trifluoromethyl)-1*H*-benzo­[*d*]­imidazol-1-yl)­benzamido)-2,3-dihydro-1*H*-inden-2-yl Acetate **(** *M* **)-41**

Following the general procedure **C**, **(** *M* **)–40** (0.13 mmol, 1 equiv, 57 mg) was used, followed by purification by CC (CH_2_Cl_2_/MeOH 99:1, column dimensions: 2 × 10 cm), yielding 46 mg (71%) of a white solid. ^**1**^ **H NMR** (400 MHz, CDCl_3_) δ 7.96–7.92 (m, 1H), 7.92–7.87 (m, 1H), 7.74–7.68 (m, 2H), 7.47 (dt, *J* = 7.8, 3.9 Hz, 1H), 7.45–7.37 (m, 2H), 7.20 (t, *J* = 7.4 Hz, 1H), 7.18–7.06 (m, 3H), 6.75 (d, *J* = 7.5 Hz, 1H), 5.97 (d, *J* = 8.9 Hz, 1H), 5.43 (dd, *J* = 9.0, 5.4 Hz, 1H), 5.11 (td, *J* = 5.3, 2.0 Hz, 1H), 3.06 (dd, *J* = 17.1, 5.2 Hz, 1H), 2.92–2.73 (m, 1H), 1.97 (s, 3H); ^**13**^ **C­{** ^**1**^ **H} NMR** (101 MHz, CDCl_3_) δ 170.1, 165.5, 141.1 (q, *J* = 38.6 Hz), 140.8, 140.0, 139.1, 137.5, 134.8, 132.1, 132.1, 130.9, 129.9, 129.6, 128.5, 127.4, 126.3, 125.1, 124.4, 123.8, 122.0, 119.0 (q, *J* = 272.1 Hz), 111.0, 75.8, 55.5, 37.5, 21.1; ^**19**^ **F NMR** (376 MHz, CDCl_3_) δ −61.34 (s); ^**19**^ **F NMR** (376 MHz, DMSO-*d*_6_) δ (ppm) −59.57 (s); **HRMS** (ESI) *m*/*z* [M + H]^+^ Calcd for C_26_H_21_F_3_N_3_O_3_ 480.1530; Found 480.1531; ${[\alpha ]}_{d}^{22}$ = +173.57° (*c* = 0.14, CHCl_3_).

#### *N*-((*S*)-1-Hydroxy-4-methylpentan-2-yl)-2-((*P*)-2-(trifluoromethyl)-1*H*-benzo­[*d*]­imidazol-1-yl)­benzamide **(** *P* **)-42**

Following the general procedure **A**, (*P*)-TBBA and (*S*)-2-amino-4-methylpentan-1-ol (0.13 mmol, 1 equiv, 15 mg) were used, followed by purification by CC (CH_2_Cl_2_/MeOH 96:4, column dimensions: 2 × 10 cm), yielding 43 mg (82%) of a white solid. ^**1**^ **H NMR** (400 MHz, CDCl_3_) δ 7.99–7.90 (m, 1H), 7.88–7.83 (m, 1H), 7.71–7.64 (m, 2H), 7.46–7.37 (m, 3H), 7.15–7.08 (m, 1H), 5.53 (d, *J* = 8.2 Hz, 1H), 3.90–3.75 (m, 1H), 3.15 (dt, *J* = 10.7, 4.0 Hz, 1H), 3.02 (dt, *J* = 10.7, 4.4 Hz, 1H), 1.58 (t, *J* = 5.0 Hz, 1H), 1.30–1.20 (m, 1H), 1.13–0.98 (m, 2H), 0.78 (d, *J* = 6.6 Hz, 3H), 0.74 (d, *J* = 6.5 Hz, 3H); ^**13**^ **C­{** ^**1**^ **H} NMR** (101 MHz, CDCl_3_) δ 165.6, 141.0 (q, *J* = 38.4 Hz), 140.7, 137.5, 135.5, 131.7, 131.4, 130.9, 129.8, 129.5 (q, *J* = 1.1 Hz), 126.5, 124.6, 121.6, 119.0 (q, *J* = 271.9 Hz), 111.4, 65.0, 49.7, 40.0, 24.8, 23.0, 22.1; ^**19**^ **F NMR** (376 MHz, CDCl_3_) δ −61.34 (s); ^**19**^ **F NMR** (376 MHz, DMSO-*d*_6_) δ (ppm) −59.55 (s); **HRMS** (ESI) *m*/*z* [M + H]^+^ Calcd for C_21_H_23_F_3_N_3_O_2_ 406.1737; Found 406.1737; ${[\alpha ]}_{d}^{22}$ = −133.63° (*c* = 0.11, CHCl_3_).

#### *N*-((*S*)-1-Hydroxy-4-methylpentan-2-yl)-2-((*M*)-2-(trifluoromethyl)-1*H*-benzo­[*d*]­imidazol-1-yl)­benzamide **(** *M* **)-42**

Following the general procedure **A**, (*M*)-TBBA and (*S*)-2-amino-4-methylpentan-1-ol (0.13 mmol, 1 equiv, 15 mg) were used, followed by purification by CC (CH_2_Cl_2_/MeOH 96:4, column dimensions: 2 × 10 cm), yielding 42 mg (80%) of a white solid. ^**1**^ **H NMR** (400 MHz, CDCl_3_) δ 7.99–7.90 (m, 1H), 7.89–7.84 (m, 1H), 7.71–7.64 (m, 2H), 7.45–7.38 (m, 3H), 7.15–7.07 (m, 1H), 5.48 (d, *J* = 7.9 Hz, 1H), 3.86–3.75 (m, 1H), 3.38–3.25 (m, 2H), 1.73 (t, *J* = 5.1 Hz, 1H), 1.01–0.89 (m, 2H), 0.84–0.76 (m, 1H), 0.69 (d, *J* = 6.3 Hz, 3H), 0.62 (d, *J* = 6.1 Hz, 3H); ^**13**^ **C­{** ^**1**^ **H} NMR** (101 MHz, CDCl_3_) δ 165.8, 140.9 (q, *J* = 38.5 Hz), 140.8, 137.4 (q, *J* = 0.9 Hz), 135.4, 131.7, 131.4, 130.9, 129.9, 129.5 (q, *J* = 1.0 Hz), 126.4, 124.5, 121.7, 119.0 (q, *J* = 272.0 Hz), 111.4, 65.0, 50.0, 39.8, 24.5, 22.9, 21.9; ^**19**^ **F NMR** (376 MHz, CDCl_3_) δ −61.30 (s); ^**19**^ **F NMR** (376 MHz, DMSO-*d*_6_) δ (ppm) −59.52 (s); **HRMS** (ESI) *m*/*z* [M + H]^+^ Calcd for C_21_H_23_F_3_N_3_O_2_ 406.1737; Found 406.1735; ${[\alpha ]}_{d}^{22}$ = +70.83° (*c* = 0.12, CHCl_3_).

#### ((*S*)-4-Methyl-2-(2-((*P*)-2-(trifluoromethyl)-1*H*-benzo­[*d*]­imidazol-1-yl)­benzamido)­pentyl Acetate **(** *P* **)-43**

Following the general procedure **C**, **(** *P* **)–42** (0.13 mmol, 1 equiv, 53 mg) was used, followed by purification by CC (CH_2_Cl_2_/MeOH 99:1, column dimensions: 2 × 10 cm), yielding 44 mg (76%) of a white solid. ^**1**^ **H NMR** (400 MHz, CDCl_3_) δ 7.94–7.88 (m, 1H), 7.83 (dd, *J* = 6.6, 2.5 Hz, 1H), 7.71–7.64 (m, 2H), 7.44–7.34 (m, 3H), 7.09 (d, *J* = 7.3 Hz, 1H), 5.44 (d, *J* = 8.8 Hz, 1H), 4.08 (ddq, *J* = 12.1, 8.3, 4.2 Hz, 1H), 3.78 (dd, *J* = 11.4, 4.6 Hz, 1H), 3.28 (dd, *J* = 11.4, 3.4 Hz, 1H), 1.98 (s, 3H), 1.27–1.18 (m, 1H), 1.03 (t, *J* = 7.3 Hz, 2H), 0.78 (d, *J* = 6.6 Hz, 3H), 0.73 (d, *J* = 6.5 Hz, 3H); ^**13**^ **C­{** ^**1**^ **H} NMR** (101 MHz, CDCl_3_) δ 171.0, 165.0, 140.8 (q, *J* = 38.4 Hz), 140.7, 137.5, 135.4, 131.8, 131.5, 130.9, 129.9, 129.7, 126.4, 124.5, 121.8, 119.1 (q, *J* = 271.9 Hz), 111.3, 66.1, 46.8, 40.4, 24.7, 22.9, 22.1, 20.7; ^**19**^ **F NMR** (376 MHz, CDCl_3_) δ −61.24 (s); ^**19**^ **F NMR** (376 MHz, DMSO-*d*_6_) δ (ppm) −59.61 (s); **HRMS** (ESI) *m*/*z* [M + H]^+^ Calcd for C_23_H_25_F_3_N_3_O_3_ 448.1843; Found 448.1839; ${[\alpha ]}_{d}^{22}$ = −158.00° (*c* = 0.10, CHCl_3_).

#### (*S*)-4-Methyl-2-(2-((*M*)-2-(trifluoromethyl)-1*H*-benzo­[*d*]­imidazol-1-yl)­benzamido)­pentyl Acetate **(** *M* **)-43**

Following the general procedure **C**, **(** *M* **)–42** (0.13 mmol, 1 equiv, 53 mg) was used, followed by purification by CC (CH_2_Cl_2_/MeOH 99:1, column dimensions: 2 × 10 cm), yielding 40 mg (69%) of a white solid. ^**1**^ **H NMR** (400 MHz, CDCl_3_) δ 7.93 (dd, *J* = 6.8, 1.7 Hz, 1H), 7.91–7.85 (m, 1H), 7.72–7.64 (m, 2H), 7.44–7.37 (m, 3H), 7.13–7.08 (m, 1H), 5.39 (d, *J* = 8.3 Hz, 1H), 4.15–3.95 (m, 1H), 3.84 (d, *J* = 3.8 Hz, 2H), 2.01 (s, 3H), 0.90–0.66 (m, 3H), 0.65 (d, *J* = 6.2 Hz, 3H), 0.58 (d, *J* = 6.0 Hz, 3H); ^**13**^ **C­{** ^**1**^ **H} NMR** (101 MHz, CDCl_3_) δ 171.0, 164.9, 140.8, 140.6 (q, *J* = 38.4 Hz), 137.4, 135.2, 131.8, 131.2, 131.0, 130.2, 129.6, 126.5, 124.6, 121.7, 119.1 (q, *J* = 271.9 Hz), 111.5, 65.8, 46.9, 40.0, 24.4, 22.7, 21.9, 20.7; ^**19**^ **F NMR** (376 MHz, CDCl_3_) δ −61.13 (s); ^**19**^ **F NMR** (376 MHz, DMSO-*d*_6_) δ (ppm) −59.56 (s); **HRMS** (ESI) *m*/*z* [M + H]^+^ Calcd for C_23_H_25_F_3_N_3_O_3_ 448.1843; Found 448.1837; ${[\alpha ]}_{d}^{22}$ = +103.00° (*c* = 0.10, CHCl_3_).

#### *N*-((1*R*,2*R*)-1,3-Dihydroxy-1-(4-nitrophenyl)­propan-2-yl)-2-((*P*)-2-(trifluoromethyl)-1*H*-benzo­[*d*]­imidazol-1-yl)­benzamide **(** *P* **)-44**

Following the general procedure **A**, (*P*)-TBBA and (1*R*,2*R*)-(−)-2-amino-1-(4-nitrophenyl)-1,3-propanediol (0.13 mmol, 1 equiv, 28 mg) were used, followed by purification by CC (CH_2_Cl_2_/MeOH 96:4, column dimensions: 2 × 10 cm), yielding 58 mg (89%) of a white solid. ^**1**^ **H NMR** (400 MHz, CDCl_3_) δ 8.17–8.12 (m, 2H), 7.89 (d, *J* = 8.0 Hz, 1H), 7.71–7.60 (m, 3H), 7.45–7.34 (m, 5H), 7.05 (dt, *J* = 7.9, 0.8 Hz, 1H), 6.22 (d, *J* = 8.3 Hz, 1H), 5.06 (s, 1H), 3.98–3.93 (m, 1H), 3.66 (br. s, 1H), 3.44 (d, *J* = 10.1 Hz, 1H), 3.03 (d, *J* = 13.3 Hz, 1H), 1.91 (br. s, 1H); ^**13**^ **C­{** ^**1**^ **H} NMR** (101 MHz, CDCl_3_) δ 165.5, 148.3, 147.4, 141.4 (q, *J* = 38.9 Hz), 140.8, 137.3, 134.2, 132.3, 131.6, 131.1, 130.0, 129.7, 126.5, 124.8, 123.6, 121.7, 118.7 (q, *J* = 272.3 Hz), 111.1, 72.4 (d, *J* = 5.5 Hz), 63.4, 55.8; ^**19**^ **F NMR** (376 MHz, CDCl_3_) δ −61.72 (s); ^**19**^ **F NMR** (376 MHz, DMSO-*d*_6_) δ (ppm) −59.62 (s); **HRMS** (ESI) *m*/*z* [M + H]^+^ Calcd for C_24_H_20_F_3_N_4_O_5_ 501.1380; Found 501.1387; ${[\alpha ]}_{d}^{22}$ = +101.71° (*c* = 0.13, MeOH).

#### *N*-((1*R*,2*R*)-1,3-Dihydroxy-1-(4-nitrophenyl)­propan-2-yl)-2-((*M*)-2-(trifluoromethyl)-1*H*-benzo­[*d*]­imidazol-1-yl)­benzamide **(** *M* **)-44**

Following the general procedure **A**, (*M*)-TBBA and (1*R*,2*R*)-(−)-2-amino-1-(4-nitrophenyl)-1,3-propanediol (0.13 mmol, 1 equiv, 28 mg) were used, followed by purification by CC (CH_2_Cl_2_/MeOH 96:4, column dimensions: 2 × 10 cm), yielding 54 mg (83%) of a white solid. ^**1**^ **H NMR** (400 MHz, CDCl_3_) δ 8.19–8.11 (m, 2H), 7.90 (ddd, *J* = 8.2, 1.2, 0.8 Hz, 1H), 7.71–7.60 (m, 3H), 7.46–7.34 (m, 5H), 7.05 (ddd, *J* = 8.0, 1.3, 0.8 Hz, 1H), 6.22 (d, *J* = 8.1 Hz, 1H), 5.06 (d, *J* = 2.6 Hz, 1H), 3.95 (ddt, *J* = 8.4, 4.2, 2.9 Hz, 1H), 3.56 (br. s, 1H), 3.45 (dd, *J* = 10.8, 3.0 Hz, 1H), 3.02 (dd, *J* = 10.8, 4.2 Hz, 1H), 1.61 (br. s, 1H); ^**13**^ **C­{** ^**1**^ **H} NMR** (101 MHz, CDCl_3_) δ 165.7, 148.4, 147.5, 140.7 (q, *J* = 38.2 Hz), 140.4, 137.4, 134.5, 132.2, 131.3, 131.1, 129.7, 129.5, 126.6, 124.8, 123.7, 121.2, 119.0 (q, *J* = 271.8 Hz), 111.7, 72.6, 63.5, 55.4; ^**19**^ **F NMR** (376 MHz, CDCl_3_) δ −61.16 (s); ^**19**^ **F NMR** (376 MHz, DMSO-*d*_6_) δ (ppm) −59.55 (s); **HRMS** (ESI) *m*/*z* [M + H]^+^ Calcd for C_24_H_20_F_3_N_4_O_5_ 501.1380; Found 500.1378; ${[\alpha ]}_{d}^{22}$ = −67.57° (*c* = 0.13, MeOH).

#### (1*R*,2*R*)-1-(4-Nitrophenyl)-2-(2-((*P*)-2-(trifluoromethyl)-1*H*-benzo­[*d*]­imidazol-1-yl)­benzamido)­propane-1,3-diyl Diacetate **(** *P* **)-45**

Following the slightly modified general procedure **C**, **(** *P* **)–44** (0.13 mmol, 1 equiv, 65 mg), acetic anhydride (0.52 mmol, 4 equiv, 50 μL), trimethylamine (0.78 mmol, 6 equiv, 108 μL), and DMAP (0.026 mmol, 0.2 equiv, 3.2 mg) were used, followed by purification by CC (CH_2_Cl_2_/MeOH 99:1, column dimensions: 2 × 10 cm), yielding 58 mg (76%) of a white solid. ^**1**^ **H NMR** (400 MHz, CDCl_3_) δ 8.18–8.12 (m, 2H), 7.93 (dt, *J* = 8.5, 0.8 Hz, 1H), 7.74–7.67 (m, 3H), 7.47–7.42 (m, 2H), 7.39 (td, *J* = 7.7, 7.3, 1.3 Hz, 1H), 7.31 (dd, *J* = 8.9, 2.0 Hz, 2H), 7.05 (dt, *J* = 7.9, 0.9 Hz, 1H), 5.87 (d, *J* = 4.6 Hz, 1H), 5.69 (d, *J* = 9.4 Hz, 1H), 4.46 (ddt, *J* = 9.4, 5.9, 4.7 Hz, 1H), 3.34 (dd, *J* = 11.5, 4.7 Hz, 1H), 3.15 (dd, *J* = 11.5, 6.0 Hz, 1H), 2.12 (s, 3H), 1.99 (s, 3H); ^**13**^ **C­{** ^**1**^ **H} NMR** (101 MHz, CDCl_3_) δ 170.2, 169.5, 165.1, 148.0, 143.9, 140.6, 140.2 (q, *J* = 38.3 Hz), 137.3, 133.8, 132.5, 131.3, 131.2, 130.2, 129.8, 127.1, 126.8, 124.9, 124.1, 121.9, 119.1 (q, *J* = 272.0 Hz), 111.3, 72.5, 62.3, 51.9, 20.7, 20.6; ^**19**^ **F NMR** (376 MHz, CDCl_3_) δ −60.81 (s); ^**19**^ **F NMR** (376 MHz, DMSO-*d*_6_) δ (ppm) −59.71 (s); **HRMS** (ESI) *m*/*z* [M + H]^+^ Calcd for C_28_H_24_F_3_N_4_O_7_ 585.1585; Found 585.1592; ${[\alpha ]}_{d}^{22}$ = −23.00° (*c* = 0.10, CHCl_3_).

#### (1*R*,2*R*)-1-(4-nitrophenyl)-2-(2-((*M*)-2-(Trifluoromethyl)-1*H*-benzo­[*d*]­imidazol-1-yl)­benzamido)­propane-1,3-diyl Diacetate **(** *M* **)-45**

Following the slightly modified general procedure **C**, **(** *M* **)–44** (0.13 mmol, 1 equiv, 65 mg), acetic anhydride (0.52 mmol, 4 equiv, 50 μL), trimethylamine (0.78 mmol, 6 equiv, 108 μL), and DMAP (0.026 mmol, 0.2 equiv, 3.2 mg) were used, followed by purification by CC (CH_2_Cl_2_/MeOH 99:1, column dimensions: 2 × 10 cm), yielding 60 mg (78%) of a white solid^.**1**^ **H NMR** (400 MHz, CDCl_3_) δ 8.05–7.99 (m, 2H), 7.94 (dt, *J* = 8.2, 1.0 Hz, 1H), 7.84–7.77 (m, 1H), 7.71–7.67 (m, 2H), 7.45 (ddd, *J* = 8.2, 7.2, 1.2 Hz, 1H), 7.41–7.34 (m, 2H), 7.11–7.07 (m, 2H), 7.00 (dt, *J* = 8.1, 1.0 Hz, 1H), 5.87 (d, *J* = 4.5 Hz, 1H), 5.78 (d, *J* = 9.3 Hz, 1H), 4.61 (dtd, *J* = 10.0, 5.5, 4.6 Hz, 1H), 3.91 (dd, *J* = 11.5, 5.6 Hz, 1H), 3.62 (dd, *J* = 11.5, 5.5 Hz, 1H), 2.08 (s, 3H), 2.04 (s, 3H); ^**13**^ **C­{** ^**1**^ **H} NMR** (101 MHz, CDCl_3_) δ 170.4, 169.7, 165.0, 147.8, 143.5, 141.1 (q, *J* = 38.4 Hz), 140.8, 137.6, 133.9, 132.5, 131.6, 131.2, 130.1, 130.1, 126.8, 126.5, 124.7, 124.0, 122.0, 118.9 (q, *J* = 272.3 Hz), 111.1, 72.6, 62.9, 52.1, 20.7; ^**19**^ **F NMR** (376 MHz, CDCl_3_) δ −61.41 (s); ^**19**^ **F NMR** (376 MHz, DMSO-*d*_6_) δ (ppm) −59.58 (s); **HRMS** (ESI) *m*/*z* [M + H]^+^ Calcd for C_28_H_24_F_3_N_4_O_7_ 585.1592; Found 585.1591; ${[\alpha ]}_{d}^{22}$ = +158.00° (*c* = 0.10, CHCl_3_).

#### *N*-((4*R*,5*R*)-2,2-Dimethyl-4-(4-nitrophenyl)-1,3-dioxan-5-yl)-2-((*P*)-2-(trifluoromethyl)-1*H*-benzo­[*d*]­imidazol-1-yl)­benzamide **(** *P* **)-46**

Following the general procedure **A**, (*P*)-TBBA and **62** (0.13 mmol, 1 equiv, 33 mg) were used, followed by purification by CC (CH_2_Cl_2_/MeOH 98:2, column dimensions: 2 × 10 cm), yielding 50 mg (71%) of a white solid. ^**1**^ **H NMR** (400 MHz, CDCl_3_) δ 8.16–8.08 (m, 2H), 7.91 (dt, *J* = 8.2, 0.9 Hz, 1H), 7.64–7.55 (m, 2H), 7.39–7.33 (m, 5H), 7.29 (ddd, *J* = 8.2, 7.2, 1.1 Hz, 1H), 6.95 (dt, *J* = 8.2, 0.9 Hz, 1H), 6.17 (d, *J* = 9.7 Hz, 1H), 5.10 (d, *J* = 1.9 Hz, 1H), 4.13 (dq, *J* = 9.7, 1.8 Hz, 1H), 3.98 (dd, *J* = 12.1, 1.9 Hz, 1H), 2.89 (dd, *J* = 12.2, 1.8 Hz, 1H), 1.47 (s, 3H), 1.45 (s, 3H); ^**13**^ **C­{** ^**1**^ **H} NMR** (101 MHz, CDCl_3_) δ 164.8, 147.4, 145.6, 140.7, 140.5 (q, *J* = 38.3 Hz), 137.4, 134.4, 131.9, 131.4, 130.9, 129.6, 129.5, 126.4, 126.3, 124.4, 123.5, 121.7, 119.1 (q, *J* = 271.5 Hz), 111.4, 100.1, 71.6, 64.2, 46.6, 29.4, 18.4; ^**19**^ **F NMR** (376 MHz, CDCl_3_) δ −61.06 (s); ^**19**^ **F NMR** (376 MHz, DMSO-*d*_6_) δ (ppm) −59.63 (s); **HRMS** (ESI) *m*/*z* [M + H]^+^ Calcd for C_27_H_24_F_3_N_4_O_5_ 541.1693; Found 541.1699; ${[\alpha ]}_{d}^{22}$ = −143.75° (*c* = 0.16, CHCl_3_).

#### *N*-((4*R*,5*R*)-2,2-Dimethyl-4-(4-nitrophenyl)-1,3-dioxan-5-yl)-2-((*M*)-2-(trifluoromethyl)-1*H*-benzo­[*d*]­imidazol-1-yl)­benzamide **(** *M* **)-46**

Following the general procedure **A**, (*M*)-TBBA and **62** (0.13 mmol, 1 equiv, 33 mg) were used, followed by purification by CC (CH_2_Cl_2_/MeOH 98:2, column dimensions: 2 × 10 cm), yielding 62 mg (89%) of a yellow solid. ^**1**^ **H NMR** (400 MHz, CDCl_3_) δ 8.08–7.97 (m, 2H), 7.87 (dd, *J* = 7.2, 1.3 Hz, 1H), 7.62–7.57 (m, 2H), 7.54–7.50 (m, 1H), 7.39–7.32 (m, 2H), 7.29–7.23 (m, 3H), 6.94 (dd, *J* = 7.3, 1.4 Hz, 1H), 6.18 (d, *J* = 9.3 Hz, 1H), 5.10 (d, *J* = 1.7 Hz, 1H), 4.24 (dq, *J* = 9.4, 1.8 Hz, 1H), 4.08 (dd, *J* = 12.2, 1.9 Hz, 1H), 3.42 (dd, *J* = 12.2, 1.8 Hz, 1H), 1.48 (s, 3H), 1.39 (s, 3H); ^**13**^ **C­{** ^**1**^ **H} NMR** (101 MHz, CDCl_3_) δ 164.9, 147.3, 145.1, 141.1 (q, *J* = 38.5 Hz), 140.8, 137.3, 134.3, 132.0, 131.6, 130.8, 129.8, 129.6, 126.3, 125.9, 124.3, 123.5, 121.8, 118.8 (q, *J* = 272.3 Hz), 110.9, 100.0, 71.4, 64.4, 46.7, 29.4, 18.5; ^**19**^ **F NMR** (376 MHz, CDCl_3_) δ −61.57 (s); ^**19**^ **F NMR** (376 MHz, DMSO-*d*_6_) δ (ppm) −59.60 (s); **HRMS** (ESI) *m*/*z* [M + H]^+^ Calcd for C_27_H_24_F_3_N_4_O_5_ 541.1693; Found 541.1712; ${[\alpha ]}_{d}^{22}$ = +120.63° (*c* = 0.16, CHCl_3_).

#### *N*-((4*R*,5*R*)-4-(4-Aminophenyl)-2,2-dimethyl-1,3-dioxan-5-yl)-2-((*P*)-2-(trifluoromethyl)-1*H*-benzo­[*d*]­imidazol-1-yl)­benzamide **(** *P* **)-47**

Following the general procedure **A**, (*P*)-TBBA and **61** (0.13 mmol, 1 equiv, 29 mg) were used, followed by purification by CC (CH_2_Cl_2_/EtOAc 4:1, column dimensions: 2 × 10 cm), yielding 28 mg (42%) of a white foamy solid. ^**1**^ **H NMR** (400 MHz, CDCl_3_) δ 7.93–7.88 (m, 1H), 7.62–7.55 (m, 2H), 7.41–7.30 (m, 4H), 7.03–6.97 (m, 3H), 6.63–6.59 (m, 2H), 6.20 (d, *J* = 9.5 Hz, 1H), 4.97 (d, *J* = 1.9 Hz, 1H), 3.96–3.90 (m, 2H), 3.63 (s, 2H), 2.98 (dd, *J* = 12.1, 1.9 Hz, 1H), 1.44 (d, *J* = 10.6 Hz, 6H); ^**13**^ **C­{** ^**1**^ **H} NMR** (101 MHz, CDCl_3_) δ 165.0, 145.9, 140.7, 140.5 (q, *J* = 38.4 Hz), 137.6, 135.4, 131.6, 131.4, 130.7, 129.5, 129.4, 128.4, 126.4, 126.2, 124.2, 121.6, 119.1 (q, *J* = 271.7 Hz), 115.1, 111.7, 99.6, 71.6, 64.2, 47.3, 29.6, 18.5; ^**19**^ **F NMR** (471 MHz, CDCl_3_) δ (ppm) −61.58 (s); ^**19**^ **F NMR** (376 MHz, DMSO-*d*_6_) δ (ppm) −59.56 (s); **HRMS** (ESI) *m*/*z* [M + H]^+^ Calcd for C_27_H_26_F_3_N_4_O_3_ 511.1952; Found 511.1956; ${[\alpha ]}_{d}^{22}$ = −81.67° (*c* = 0.18, CHCl_3_).

#### *N*-((4*R*,5*R*)-4-(4-Aminophenyl)-2,2-dimethyl-1,3-dioxan-5-yl)-2-((*M*)-2-(trifluoromethyl)-1*H*-benzo­[*d*]­imidazol-1-yl)­benzamide **(** *M* **)-47**

Following the general procedure **A**, (*M*)-TBBA and **61** (0.13 mmol, 1 equiv, 29 mg) were used, followed by purification by CC (CH_2_Cl_2_/EtOAc 4:1, column dimensions: 2 × 10 cm), yielding 30 mg (45%) of a white foamy solid. ^**1**^ **H NMR** (400 MHz, CDCl_3_) δ 7.95–7.90 (m, 1H), 7.63–7.55 (m, 2H), 7.48–7.44 (m, 1H), 7.41–7.34 (m, 3H), 7.05–7.01 (m, 1H), 6.93 (d, *J* = 8.1 Hz, 2H), 6.62–6.56 (m, 2H), 6.18 (d, *J* = 8.8 Hz, 1H), 5.01 (d, *J* = 1.8 Hz, 1H), 4.00–3.92 (m, 2H), 3.65 (s, 2H), 3.35–3.24 (m, 1H), 1.47 (s, 3H), 1.39 (s, 3H); ^**13**^ **C­{** ^**1**^ **H} NMR** (101 MHz, CDCl_3_) δ 164.8, 145.9, 141.5 (q, *J* = 38.6 Hz), 140.9, 137.4, 134.9, 132.2, 131.6, 130.6, 129.7, 129.1, 128.0, 126.1, 126.0, 124.1, 121.7, 118.8 (q, *J* = 272.4 Hz), 115.1, 111.1, 99.5, 71.4, 64.0, 47.3, 29.7, 18.6; ^**19**^ **F NMR** (376 MHz, CDCl_3_) δ −61.73 (s); ^**19**^ **F NMR** (376 MHz, DMSO-*d*_6_) δ (ppm) −59.41 (s); **HRMS** (ESI) *m*/*z* [M + H]^+^ Calcd for C_27_H_26_F_3_N_4_O_3_ 511.1952; Found 511.1950; ${[\alpha ]}_{d}^{22}$ = +5.00° (*c* = 0.18, CHCl_3_).

#### *N*-((1*S*,2S)-2-Amino-1,2-diphenylethyl)-2-((*P*)-2-(trifluoromethyl)-1*H*-benzo­[*d*]­imidazol-1-yl)­benzamide **(** *P* **)-48**

Following the slightly modified general procedure **A**, (*P*)-TBBA and (1*S*,2*S*)-(−)-1,2-diphenylethylenediamine (0.52 mmol, 4 equiv, 110 mg), EDCl (35 mg, 0.18 mmol, 1.4 equiv), and HOBt (28 mg, 0.18 mmol, 1.4 equiv) were used. Extraction was performed with water (3 × 10 mL), K_2_CO_3_ (3 × 10 mL), and brine (1 × 10 mL). Purification by CC (CH_2_Cl_2_/MeOH 98:1, column dimensions: 2 × 10 cm) yielded 49 mg (75%) of a white solid. ^**1**^ **H NMR** (400 MHz, CDCl_3_) δ 7.94–7.87 (m, 1H), 7.72–7.67 (m, 1H), 7.66–7.61 (m, 2H), 7.43–7.36 (m, 3H), 7.30–7.21 (m, 3H), 7.19–7.10 (m, 6H), 7.04 (d, *J* = 7.0 Hz, 1H), 6.88–6.82 (m, 2H), 4.92 (dd, *J* = 7.1, 3.9 Hz, 1H), 4.18 (d, *J* = 3.9 Hz, 1H), 1.25 (br. s, 2H); ^**13**^ **C­{** ^**1**^ **H} NMR** (101 MHz, CDCl_3_) δ 164.9, 141.9, 141.5 (q, *J* = 38.2 Hz), 141.0, 139.8, 137.7, 135.3, 132.1, 131.7, 130.7, 129.8, 129.7, 128.6, 127.7, 127.4, 126.3, 126.1, 126.1, 124.2, 121.8, 118.8 (q, *J* = 272.2 Hz), 111.3, 59.4; ^**19**^ **F NMR** (376 MHz, CDCl_3_) δ −61.63 (s); ^**19**^ **F NMR** (376 MHz, DMSO-*d*_6_) δ (ppm) −59.54 (s); **HRMS** (ESI) *m*/*z* [M + H]^+^ Calcd for C_29_H_24_F_3_N_4_O 501.1897; Found 501.1895; ${[\alpha ]}_{d}^{22}$ = −58.89° (*c* = 0.2, MeOH).

#### *N*-((1*S*,2*S*)-2-Amino-1,2-diphenylethyl)-2-((*M*)-2-(trifluoromethyl)-1*H*-benzo­[*d*]­imidazol-1-yl)­benzamide **(** *M* **)-48**

Following the slightly modified general procedure **A**, (*M*)-TBBA and (1*S*,2*S*)-(−)-1,2-diphenylethylenediamine (0.52 mmol, 4 equiv, 110 mg), EDCl (35 mg, 0.18 mmol, 1.4 equiv), and HOBt (28 mg, 0.18 mmol, 1.4 equiv) were used. Extraction was performed with water (3 × 10 mL), K_2_CO_3_ (3 × 10 mL), and brine (1 × 10 mL). Purification by CC (CH_2_Cl_2_/MeOH 98:1, column dimensions: 2 × 10 cm) yielded 53 mg (82%) of a white solid. ^**1**^ **H NMR** (400 MHz, CDCl_3_) δ 7.94 (d, *J* = 8.2 Hz, 1H), 7.72–7.67 (m, 1H), 7.66–7.61 (m, 2H), 7.41–7.34 (m, 2H), 7.31–7.22 (m, 6H), 7.13–7.04 (m, 4H), 7.01 (d, *J* = 8.3 Hz, 1H), 6.62–6.54 (m, 2H), 4.92 (dd, *J* = 7.1, 3.4 Hz, 1H), 4.18 (d, *J* = 3.4 Hz, 1H), 1.24 (br. s, 2H); ^**13**^ **C­{** ^**1**^ **H} NMR** (101 MHz, CDCl_3_) δ 165.1, 142.1, 140.8, 140.8 (q, *J* = 38.2, 37.5 Hz), 139.8, 135.1, 131.6, 131.5, 130.7, 130.2, 129.5, 128.6, 127.7, 127.2, 126.5, 126.3, 125.8, 124.4, 121.5, 119.1 (q, *J* = 272.2 Hz), 111.7, 59.4; ^**19**^ **F NMR** (376 MHz, CDCl_3_) δ −60.78 (s); ^**19**^ **F NMR** (376 MHz, DMSO-*d*_6_) δ (ppm) −59.57 (s); **HRMS** (ESI) *m*/*z* [M + H]^+^ Calcd for C_29_H_24_F_3_N_4_O 501.1897; Found 501.1891; ${[\alpha ]}_{d}^{22}$ = +98.29° (*c* = 0.13, MeOH).

#### *N*-((1*S*,2*S*)-2-(Dimethylamino)-1,2-diphenylethyl)-2-((*P*)-2-(trifluoromethyl)-1*H*-benzo­[*d*]­imidazol-1-yl)­benzamide **(** *P* **)-49**

Following the general procedure **A**, (*P*)-TBBA and **59** (0.13 mmol, 1 equiv, 31 mg) were used, followed by purification by CC (CH_2_Cl_2_/MeOH 99:1, column dimensions: 2 × 10 cm), yielding 56 mg (83%) of a light yellow solid. ^**1**^ **H NMR** (400 MHz, CDCl_3_) δ 8.04–7.95 (m, 2H), 7.73–7.59 (m, 2H), 7.47–7.41 (m, 2H), 7.32 (dd, *J* = 7.5, 1.3 Hz, 1H), 7.27 (d, *J* = 2.4 Hz, 1H), 7.20–7.12 (m, 4H), 7.04–6.96 (m, 3H), 6.88–6.82 (m, 4H), 4.89 (dd, *J* = 11.2, 3.3 Hz, 1H), 3.24 (d, *J* = 11.2 Hz, 1H), 1.72 (s, 6H); ^**13**^ **C­{** ^**1**^ **H} NMR** (101 MHz, CDCl_3_) δ 165.0, 141.2 (q, *J* = 38.5 Hz), 141.0, 140.2, 138.0, 135.3, 131.8, 131.7, 131.5, 130.9, 130.8, 129.8, 129.6, 128.0, 127.7, 127.3, 126.9, 126.4, 124.4, 121.7, 119.0 (q, *J* = 272.3 Hz), 111.6, 73.3, 55.0, 40.2; ^**19**^ **F NMR** (376 MHz, CDCl_3_) δ −61.44 (s); ^**19**^ **F NMR** (376 MHz, DMSO-*d*_6_) δ (ppm) −59.53 (s); **HRMS** (ESI) *m*/*z* [M + H]^+^ Calcd for C_31_H_28_F_3_N_4_O 529.2210; Found 529.2214; ${[\alpha ]}_{d}^{22}$ = −75.11° (*c* = 0.42, CHCl_3_).

#### *N*-((1*S*,2*S*)-2-(Dimethylamino)-1,2-diphenylethyl)-2-((*M*)-2-(trifluoromethyl)-1*H*-benzo­[*d*]­imidazol-1-yl)­benzamide **(** *M* **)-49**

Following the general procedure **A**, (*M*)-TBBA and **59** (0.13 mmol, 1 equiv, 31 mg) were used, followed by purification by CC (CH_2_Cl_2_/MeOH 99:1, column dimensions: 2 × 10 cm), yielding 60 mg (88%) of a light yellow solid. ^**1**^ **H NMR** (400 MHz, CDCl_3_) δ 8.16–8.11 (m, 1H), 8.08 (dt, *J* = 8.3, 0.9 Hz, 1H), 7.72–7.65 (m, 2H), 7.52 (ddd, *J* = 8.3, 7.2, 1.1 Hz, 1H), 7.42–7.34 (m, 3H), 7.17–7.12 (m, 3H), 7.04 (dt, *J* = 8.3, 0.9 Hz, 1H), 6.89–6.84 (m, 3H), 6.80–6.74 (m, 2H), 6.19 (dt, *J* = 8.5, 1.8 Hz, 2H), 4.83 (dd, *J* = 11.2, 2.6 Hz, 1H), 3.03 (d, *J* = 11.2 Hz, 1H), 1.97 (s, 6H); ^**13**^ **C­{** ^**1**^ **H} NMR** (101 MHz, CDCl_3_) δ 164.9, 140.9, 140.8 (q, *J* = 38.5 Hz), 140.0, 137.5, 134.2, 132.0, 131.7, 131.7, 131.4, 130.9, 129.7, 129.3, 127.8, 127.7, 127.0, 126.8, 126.6, 124.6, 121.7, 119.1 (q, *J* = 272.1 Hz), 111.8, 73.2, 55.2, 40.2; ^**19**^ **F NMR** (376 MHz, CDCl_3_) δ −61.02 (s); ^**19**^ **F NMR** (376 MHz, DMSO-*d*_6_) δ (ppm) −59.61 (s); **HRMS** (ESI) *m*/*z* [M + H]^+^ Calcd for C_31_H_28_F_3_N_4_O 529.2210; Found 529.2211; ${[\alpha ]}_{d}^{22}$ = +53.57° (*c* = 0.14, CHCl_3_).

#### *N*-((1*S*,2*S*)-2-Acetamido-1,2-diphenylethyl)-2-((*P*)-2-(trifluoromethyl)-1*H*-benzo­[*d*]­imidazol-1-Yl)­benzamide **(** *P* **)-50**

Following the general procedure **C**, **(** *P* **)–48** (0.13 mmol, 1 equiv, 65 mg) was used, followed by purification by CC (CH_2_Cl_2_/MeOH 99:1, column dimensions: 2 × 10 cm), yielding 55 mg (78%) of a white solid. ^**1**^ **H NMR** (400 MHz, CDCl_3_) δ 7.85–7.80 (m, 2H), 7.68–7.64 (m, 2H), 7.41–7.38 (m, 1H), 7.36–7.30 (m, 2H), 7.22 (ddd, *J* = 8.2, 7.2, 1.1 Hz, 1H), 7.16–7.11 (m, 3H), 7.09–6.99 (m, 5H), 6.96 (dt, *J* = 8.2, 0.9 Hz, 1H), 6.82–6.77 (m, 2H), 6.33 (d, *J* = 7.8 Hz, 1H), 5.20 (dd, *J* = 11.1, 7.9 Hz, 1H), 5.09 (dd, *J* = 11.2, 7.7 Hz, 1H), 1.93 (s, 3H); ^**13**^ **C­{** ^**1**^ **H} NMR** (101 MHz, CDCl_3_) δ 171.4, 165.6, 140.9 (q, *J* = 38.3 Hz), 140.6, 138.3, 138.1, 137.6, 134.7, 132.5, 131.8, 130.8, 130.0, 129.1, 128.7, 128.5, 128.1, 127.7, 127.5, 127.2, 126.1, 123.9, 121.6, 118.9 (q, *J* = 271.8 Hz), 111.1, 59.9, 59.1, 23.4; ^**19**^ **F NMR** (376 MHz, CDCl_3_) δ −61.66 (s); ^**19**^ **F NMR** (376 MHz, DMSO-*d*_6_) δ (ppm) −59.57 (s); **HRMS** (ESI) *m*/*z* [M + H]^+^ Calcd for C_24_H_19_F_3_N_3_O_2_ 543.2002; Found 543.1997; ${[\alpha ]}_{d}^{22}$ = −15.00° (*c* = 0.10, MeOH).

#### *N*-((1*S*,2*S*)-2-Acetamido-1,2-diphenylethyl)-2-((*P*)-2-(trifluoromethyl)-1*H*-benzo­[*d*]­imidazol-1-yl)­benzamide **(** *M* **)-50**

Following the general procedure **C**, **(** *M* **)–48** (0.13 mmol, 1 equiv, 65 mg) was used, followed by purification by CC (CH_2_Cl_2_/MeOH 99:1, column dimensions: 2 × 10 cm), yielding 52 mg (74%) of a white solid. ^**1**^ **H NMR** (400 MHz, CDCl_3_) δ 7.82 (ddd, *J* = 6.9, 4.2, 1.7 Hz, 2H), 7.69–7.63 (m, 2H), 7.39–7.30 (m, 3H), 7.13–6.98 (m, 9H), 6.85–6.67 (m, 3H), 6.45 (d, *J* = 7.6 Hz, 1H), 5.18 (dd, *J* = 10.9, 8.0 Hz, 1H), 5.04 (dd, *J* = 11.0, 8.0 Hz, 1H), 1.89 (s, 3H); ^**13**^ **C­{** ^**1**^ **H} NMR** (101 MHz, CDCl_3_) δ 170.8, 166.0, 140.7 (q, *J* = 35.7, 35.0 Hz), 140.6, 138.4, 137.7, 137.6, 134.6, 132.3, 132.0, 130.9, 130.0, 129.3, 128.8, 128.7, 128.1, 128.0, 127.5, 127.1, 126.1, 124.1, 121.8, 118.9 (q, *J* = 272.2 Hz), 111.2, 59.7, 59.2, 23.5; ^**19**^ **F NMR** (376 MHz, CDCl_3_) δ −61.44 (s); ^**19**^ **F NMR** (376 MHz, DMSO-*d*_6_) δ (ppm) −59.66 (s); **HRMS** (ESI) *m*/*z* [M + H]^+^ Calcd for C_24_H_19_F_3_N_3_O_2_ 543.2002; Found 543.2007; ${[\alpha ]}_{d}^{22}$ = +134.17° (*c* = 0.24, MeOH).

#### *tert*-Butyl ((1*S*,2*S*)-1,2-diphenyl-2-(2-((*P*)-2-(trifluoromethyl)-1*H*-benzo­[*d*]­imidazol-1-yl)­benzamido)­ethyl)­carbamate **(** *P* **)-51**

Following the general procedure **A**, (*P*)-TBBA and **58** (0.13 mmol, 1 equiv, 41 mg) were used, followed by purification by CC (CH_2_Cl_2_/MeOH 99:1, column dimensions: 2 × 10 cm), yielding 46 mg (59%) of a white solid. ^**1**^ **H NMR** (400 MHz, CDCl_3_) δ 7.90 (dt, *J* = 7.4, 3.7 Hz, 1H), 7.84 (d, *J* = 8.2 Hz, 1H), 7.64 (dt, *J* = 7.5, 4.0 Hz, 2H), 7.44–7.35 (m, 2H), 7.32 (t, *J* = 7.3 Hz, 1H), 7.21 (t, *J* = 7.8 Hz, 1H), 7.16–7.12 (m, 3H), 7.06–6.92 (m, 6H), 6.69 (d, *J* = 7.1 Hz, 2H), 5.21 (d, *J* = 7.6 Hz, 1H), 5.05 (dd, *J* = 10.7, 7.7 Hz, 1H), 4.88–4.68 (m, 1H), 1.37 (s, 9H); ^**13**^ **C­{** ^**1**^ **H} NMR** (101 MHz, CDCl_3_) δ 165.1, 141.0 (q, *J* = 38.7 Hz), 140.7, 138.7, 138.4, 137.6, 134.8, 132.6, 131.8, 130.6, 130.0, 129.3, 128.8, 128.4, 128.1, 127.5, 127.1, 126.0, 123.9, 121.6, 119.0 (q, *J* = 272.2 Hz), 111.0, 80.5, 60.6, 60.1, 28.3; ^**19**^ **F NMR** (376 MHz, CDCl_3_) δ −61.73 (s); ^**19**^ **F NMR** (376 MHz, DMSO-*d*_6_) δ (ppm) −59.61 (s); **HRMS** (ESI) *m*/*z* [M + H]^+^ Calcd for C_34_H_32_F_3_N_4_O_3_ 601.2421; Found 601.2422; ${[\alpha ]}_{d}^{22}$ = −79.23° (*c* = 0.13, CHCl_3_).

#### *tert*-Butyl ((1*S*,2*S*)-1,2-diphenyl-2-(2-((*M*)-2-(trifluoromethyl)-1*H*-benzo­[*d*]­imidazol-1-yl)­benzamido)­ethyl)­carbamate **(** *M* **)-51**

Following the general procedure **A**, (*M*)-TBBA and **58** (0.13 mmol, 1 equiv, 41 mg), followed by purification by CC (CH_2_Cl_2_/MeOH 99:1, column dimensions: 2 × 10 cm), yielding 52 mg (67%) of a white solid. ^**1**^ **H NMR** (400 MHz, CDCl_3_) δ 7.91–7.86 (m, 1H), 7.83–7.77 (m, 1H), 7.68–7.62 (m, 2H), 7.40–7.36 (m, 1H), 7.34–7.29 (m, 2H), 7.17–6.99 (m, 8H), 6.99–6.89 (m, 2H), 6.71 (d, *J* = 7.0 Hz, 2H), 5.21 (d, *J* = 7.7 Hz, 1H), 5.02–4.91 (m, 1H), 4.94–4.75 (m, 1H), 1.40 (s, 9H); ^**13**^ **C­{** ^**1**^ **H} NMR** (101 MHz, CDCl_3_) δ 165.5, 156.9, 140.5, 140.5 (q, *J* = 38.2 Hz), 138.5, 138.2, 137.4, 134.8, 132.1, 131.7, 130.5, 129.7, 129.3, 128.7, 128.5, 128.0, 127.6, 127.4, 126.9, 125.8, 123.9, 121.6, 118.9 (q, *J* = 272.0 Hz), 111.2, 80.4, 60.1, 59.9, 28.4; ^**19**^ **F NMR** (376 MHz, CDCl_3_) δ −61.27 (s); ^**19**^ **F NMR** (376 MHz, DMSO-*d*_6_) δ (ppm) −59.63 (s); **HRMS** (ESI) *m*/*z* [M + H]^+^ Calcd for C_34_H_32_F_3_N_4_O_3_ 601.2421; Found 601.2436; ${[\alpha ]}_{d}^{22}$ = +283.13° (*c* = 0.16, CHCl_3_).

## Supplementary Material



## Data Availability

The data underlying this study are available in the published article and its Supporting Information.

## Notes

Copies of spectra (^1^H NMR, ^13^C­{^1^H} NMR, and ^19^F NMR), HRMS, optical rotation, experimental procedures for starting materials, conformational sampling, and DFT calculations (PDF)
