# Supplementary material for: Application of 19 F NMR Spectroscopy for Determining the Absolute Configuration of α‑Chiral Amines and Secondary Alcohols Using Trifluoromethylbenzoimidazolylbenzoic Acid
Source: J Org Chem. 2025 Jul 9;90(28):9867–84. doi: 10.1021/acs.joc.5c00879 (PMC12281562; doi:10.1021/acs.joc.5c00879)
Supplement: Supplementary file 1 [file jo5c00879_si_001.pdf]

Supporting information

Application of ^{19}F NMR Spectroscopy for Determining the Absolute Configuration of α-Chiral Amines and Secondary Alcohols Using Trifluoromethylbenzoimidazolylbenzoic Acid

David Profous,[a] Michal Kriegelstein,[a] Petr Jurečka,[b] and Petr Cankař[a]*

[a]Department of Organic Chemistry, Faculty of Science, Palacký University Olomouc, 17. Listopadu 1192/12, 779 00 Olomouc, Czech Republic, email: petr.cankar@upol.cz

[b]Department of Physical Chemistry, Faculty of Science, Palacký University Olomouc, 17. listopadu 1192/12, 779 00 Olomouc, Czech Republic

Outline

References S3

General Information S5

TBBA Derivatives: Copies of ^{1}H and ^{13}C NMR Spectra S6

TBBA Derivatives: Copies of ^{19}F NMR Spectra S66

Evaluation of ^{19}F NMR chemical shifts of a mixture of TBBA amides 34 .... S124

Synthesis of starting materials S125

Starting Materials: Copies of ^{1}H and ^{13}C NMR Spectra S132

Conformational sampling and DFT calculations S144

References

(1) Crestey, F.; Witt, M.; Frydenvang, K.; Stærk, D.; Jaroszewski, J. W.; Franzyk, H. Microwave-Assisted Ring-Opening of Activated Aziridines with Resin-Bound Amines. *J. Org. Chem.* **2008**, *73* (9), 3566–3569. https://doi.org/10.1021/jo702612u.

(2) Gal, J. Synthesis of (R)- and (S)-amphetamine-d3 from the Corresponding Phenylalanines. *J. Label. Compd. Radiopharm.* **1977**, *13*, 1–9. https://doi.org/https://doi.org/10.1002/jlcr.2580130101.

(3) Berggren, K.; Johansson, B.; Fex, T.; Kihlberg, J.; Björck, L.; Luthman, K. Synthesis and Biological Evaluation of Reversible Inhibitors of IdeS, a Bacterial Cysteine Protease and Virulence Determinant. *Bioorg. Med. Chem.* **2009**, *17* (9), 3463–3470. https://doi.org/https://doi.org/10.1016/j.bmc.2009.03.026.

(4) Kochetkov, S. V; Kucherenko, A. S.; Zlotin, S. G. Asymmetric Synthesis of Warfarin and Its Analogs Catalyzed by C2-Symmetric Squaramide-Based Primary Diamines. *Org. Biomol. Chem.* **2018**, *16* (35), 6423–6429. https://doi.org/10.1039/C8OB01576G.

(5) Řezníčková, E.; Krajčovičová, S.; Peřina, M.; Kovalová, M.; Soural, M.; Kryštof, V. Modulation of FLT3-ITD and CDK9 in Acute Myeloid Leukaemia Cells by Novel Proteolysis Targeting Chimera (PROTAC). *Eur. J. Med. Chem.* **2022**, *243*, 114792. https://doi.org/https://doi.org/10.1016/j.ejmech.2022.114792.

(6) D'Arcy, T. D.; Elsegood, M. R. J.; Buckley, B. R. Organocatalytic Enantioselective Synthesis of Bicyclo[2.2.2]Octenones via Oxaziridinium Catalysed Ortho-Hydroxylative Phenol Dearomatization**. *Angew. Chemie Int. Ed.* **2022**, *61* (30), e202205278. https://doi.org/https://doi.org/10.1002/anie.202205278.

(7) Page, P. C. B.; Buckley, B. R.; Farah, M. M.; Blacker, A. J. Binaphthalene-Derived Iminium Salt Catalysts for Highly Enantioselective Asymmetric Epoxidation. *European J. Org. Chem.* **2009**, *2009* (20), 3413–3426. https://doi.org/https://doi.org/10.1002/ejoc.200900252.

(8) Dai, J.; Xiong, W.; Li, D.-Y.; Cai, Z.; Zhu, J.-B. Bifunctional Thiourea-Based Organocatalyst Promoted Kinetic Resolution Polymerization of Racemic Lactide to Isotactic Polylactide. *Chem. Commun.* **2023**, *59* (85), 12731–12734. https://doi.org/10.1039/D3CC04203K.

(9) Mari, M.; Boniburini, M.; Tosato, M.; Zanni, F.; Bonini, F.; Faglioni, F.; Cuoghi, L.; Belluti, S.; Imbriano, C.; Asti, M.; Ferrari, E. Bridging Pyrimidine Hemicurcumin and Cisplatin: Synthesis, Coordination Chemistry, and in Vitro Activity Assessment of a Novel Pt(II) Complex. *J. Inorg. Biochem.* **2024**, *260*, 112702. https://doi.org/https://doi.org/10.1016/j.jinorgbio.2024.112702.

(10) Wavefunction Inc. Spartan'24, Version 1.2.0. Wavefunction Inc: Irvine, CA 2024.

(11) Weigend, F.; Ahlrichs, R. Balanced Basis Sets of Split Valence, Triple Zeta Valence and Quadruple Zeta Valence Quality for H to Rn: Design and Assessment of Accuracy. *Phys. Chem. Chem. Phys.* **2005**, *7* (18), 3297–3305. https://doi.org/10.1039/B508541A.

(12) Grimme, S.; Antony, J.; Ehrlich, S.; Krieg, H. A Consistent and Accurate Ab Initio Parametrization of Density Functional Dispersion Correction (DFT-D) for the 94 Elements H-Pu. *J. Chem. Phys.* **2010**, *132* (15), 154104. https://doi.org/10.1063/1.3382344.

(13) Grimme, S.; Ehrlich, S.; Goerigk, L. Effect of the Damping Function in Dispersion Corrected Density Functional Theory. *J. Comput. Chem.* **2011**, *32* (7), 1456–1465. https://doi.org/https://doi.org/10.1002/jcc.21759.

(14) University of Karlsruhe; Forschungszentrum Karlsruhe GmbH; TURBOMOLE GmbH. TURBOMOLE, Version 7.2. TURBOMOLE GmbH 2017.

(15) Ahlrichs, R.; Bär, M.; Häser, M.; Horn, H.; Kölmel, C. Electronic Structure Calculations on Workstation Computers: The Program System Turbomole. *Chem. Phys. Lett.* **1989**, *162* (3), 165–169. https://doi.org/https://doi.org/10.1016/0009-2614(89)85118-8.

(16) Klamt, A.; Schüürmann, G. COSMO: A New Approach to Dielectric Screening in Solvents with Explicit Expressions for the Screening Energy and Its Gradient. *J. Chem. Soc. Perkin Trans. 2* **1993**, No. 5, 799–805. https://doi.org/10.1039/P29930000799.

General Information

All reactions were performed under normal conditions without any specific precautions to exclude moisture or air from the reaction, except where stated otherwise. Reaction workup and column chromatography were performed with commercial grade solvents without further purification. ^{1}H NMR, ^{13}C NMR, and ^{19}F NMR spectra were measured on a Jeol ECA400II (400 MHz) or Jeol ECX-500SS (500 MHz) instrument in $CDCl_3$, DMSO-d_6, or D_2O as a solvent. ^{1}H and ^{13}C spectra were referenced to residual non-deuterated solvent signals (7.26 and 77.16 ppm for $CDCl_3$, 2.50 and 39.52 ppm for DMSO-d_6). ^{19}F spectra were referenced to a $CFCl_3$ standard (δ = 0.0 ppm). All ^{13}C NMR spectra were acquired with broadband ^{1}H decoupling. ^{1}H NMR data are reported as follows: δ (chemical shift, ppm), coupling constants (*J*, Hz), and integration. Abbreviations used to denote signal multiplicity were as follows: s (singlet), d (doublet), t (triplet), q (quartet), m (multiplet), app (appears as), and br (broad).

Analytical thin-layer chromatography (TLC) was performed using Kieselgel 60 F_{254} plates (Merck). Compounds were detected under UV light (255 nm) and then by staining with basic $KMnO_4$ solution or ninhydrin solution.

Flash chromatography was performed using silica gel (35−70 µm particle size range).

HRMS analyses were carried out using an Exactive Plus Orbitrap high-resolution mass spectrometer with electrospray ionization (Thermo Fisher Scientific, MA, USA). Chromatographic pre-separation was performed using an HPLC system Dionex Ultimate 3000 (Thermo Fisher Scientific, MA, USA) equipped with a Phenomenex Gemini column (C18, 50 × 2 mm, 3.0 µm). The samples were dissolved in MeOH or acetonitrile and injected *via* an autosampler. Mobile phase compositions: isocratic elution with MeOH/water 95:5 + 0.1 % (v/v) HCOOH with a flow rate of 0.3 mL/min.

TBBA Derivatives: Copies of ^{1}H and ^{13}C NMR Spectra

(*R*)-Heptan-2-yl 2-((*P*)-2-(trifluoromethyl)-1*H*-benzo[*d*]imidazol-1-yl)benzoate **(*P*)-9**

^{1}H NMR (400 MHz, $CDCl_3$)

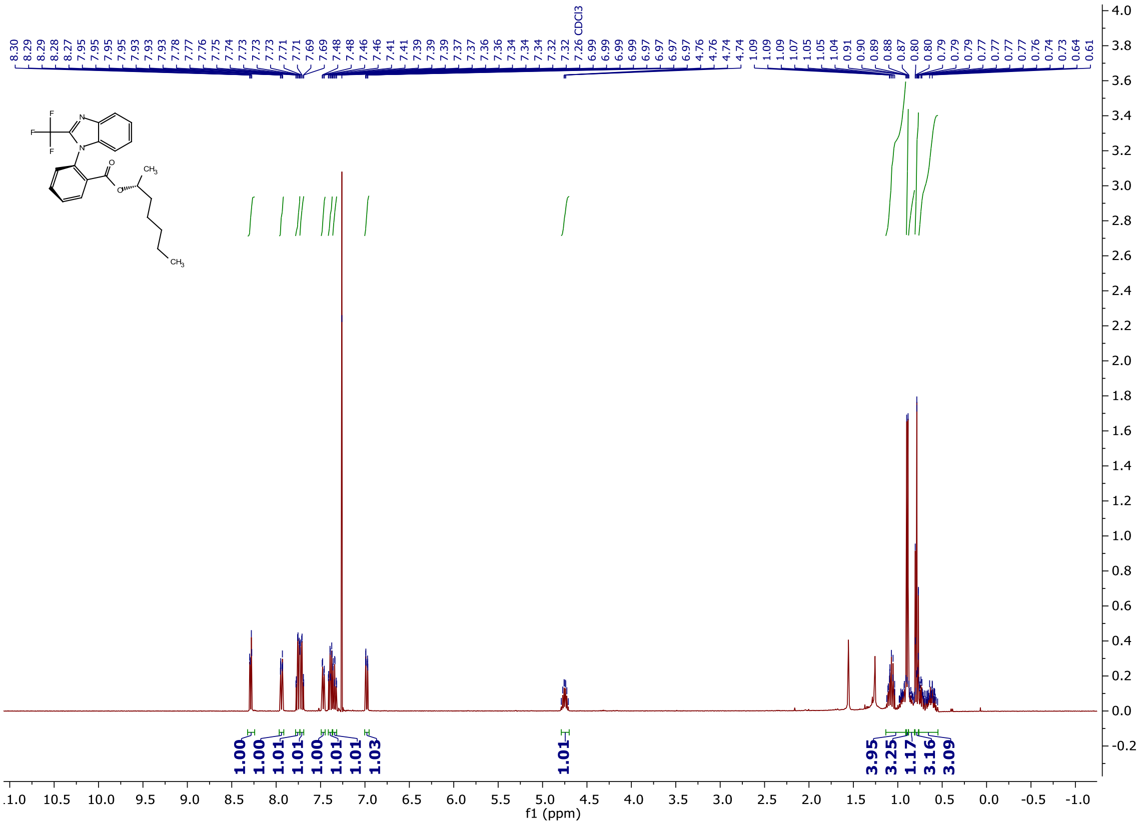

^{13}C{^{1}H} NMR (101 MHz, $CDCl_3$)

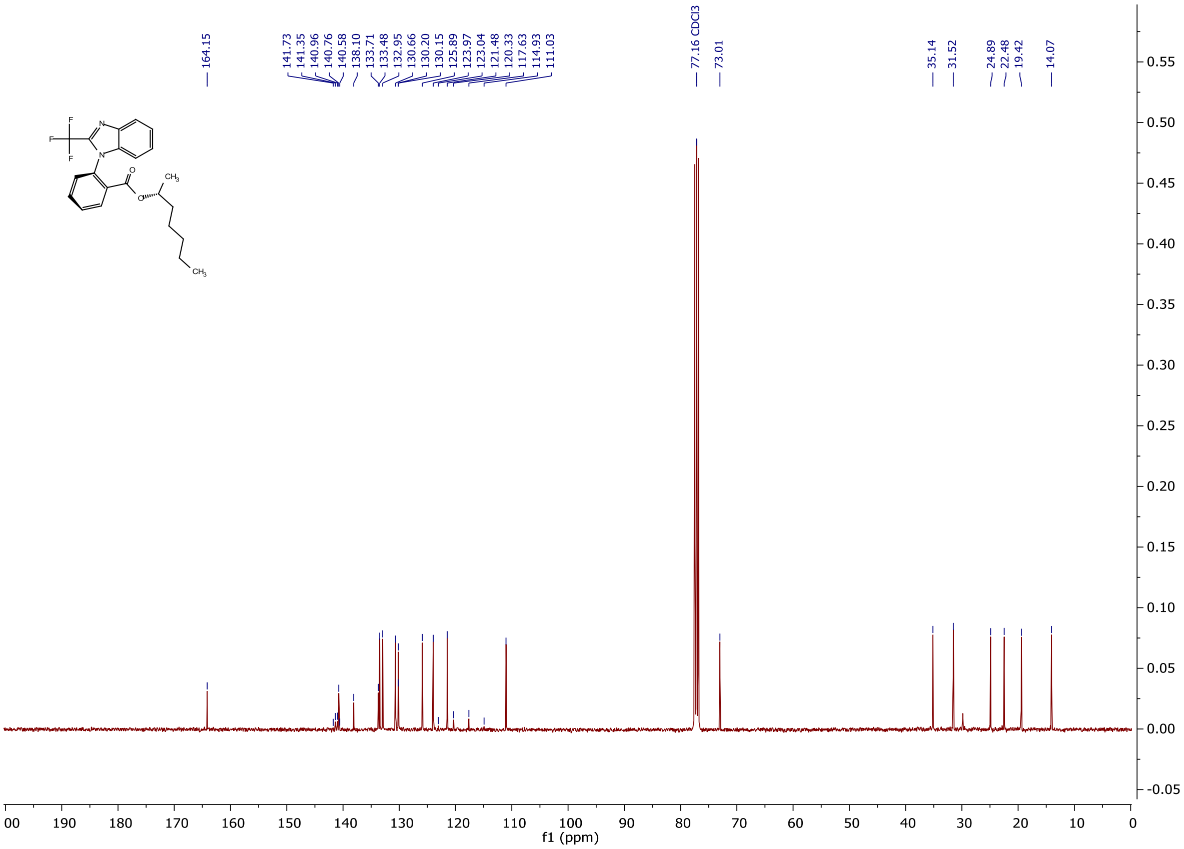

(*R*)-Heptan-2-yl 2-((*P*)-2-(trifluoromethyl)-1*H*-benzo[*d*]imidazol-1-yl)benzoate **(*M*)-9**

^{1}H NMR (400 MHz, $CDCl_3$)

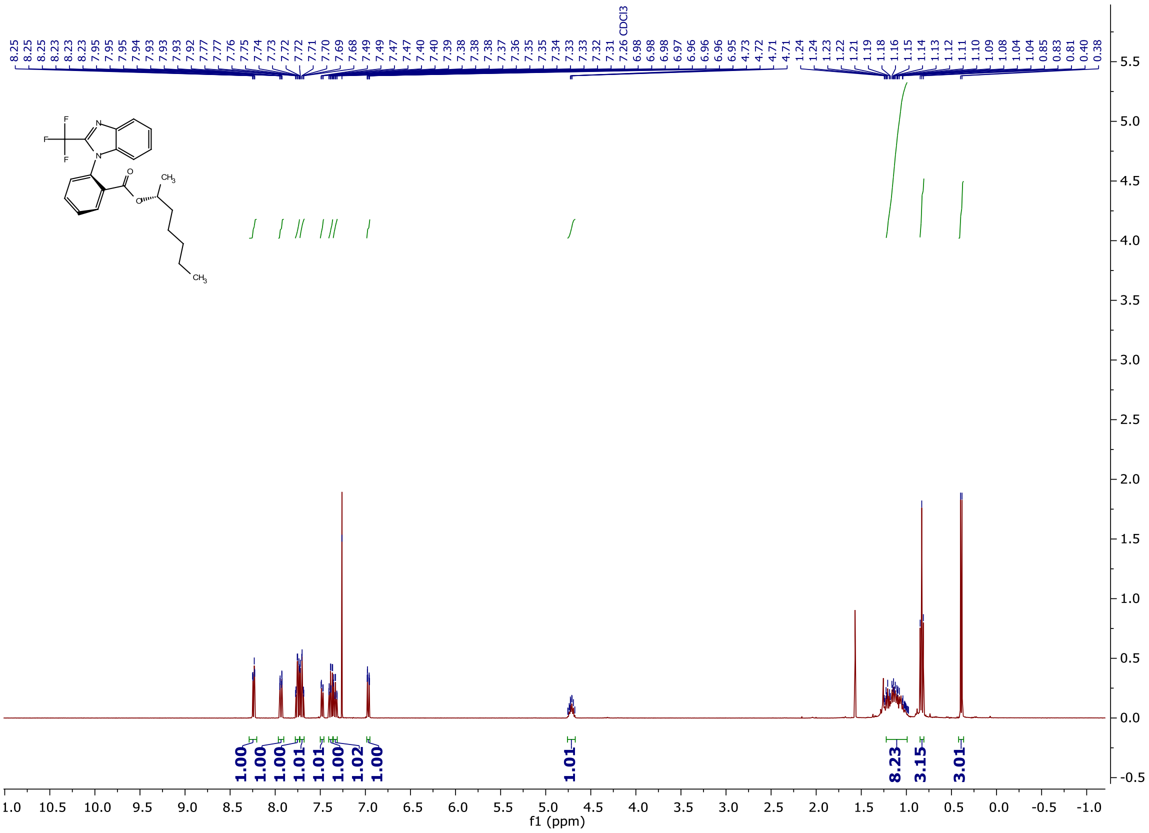

^{13}C{^{1}H} NMR (101 MHz, $CDCl_3$)

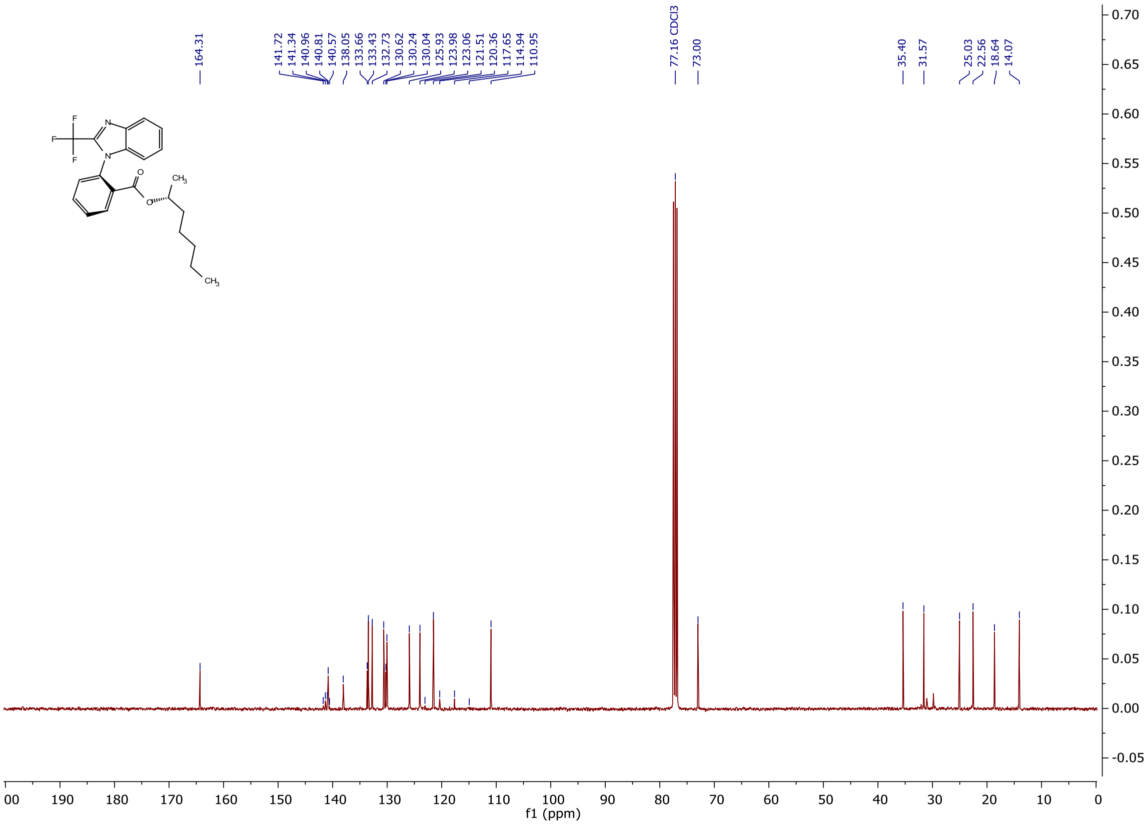

Methyl (*S*)-3,3-dimethyl-2-(2-((*P*)-2-(trifluoromethyl)-1*H*-benzo[*d*]imidazol-1-yl)benzamido)butanoate **(*P*)-16**

^{1}H NMR (400 MHz, $CDCl_3$)

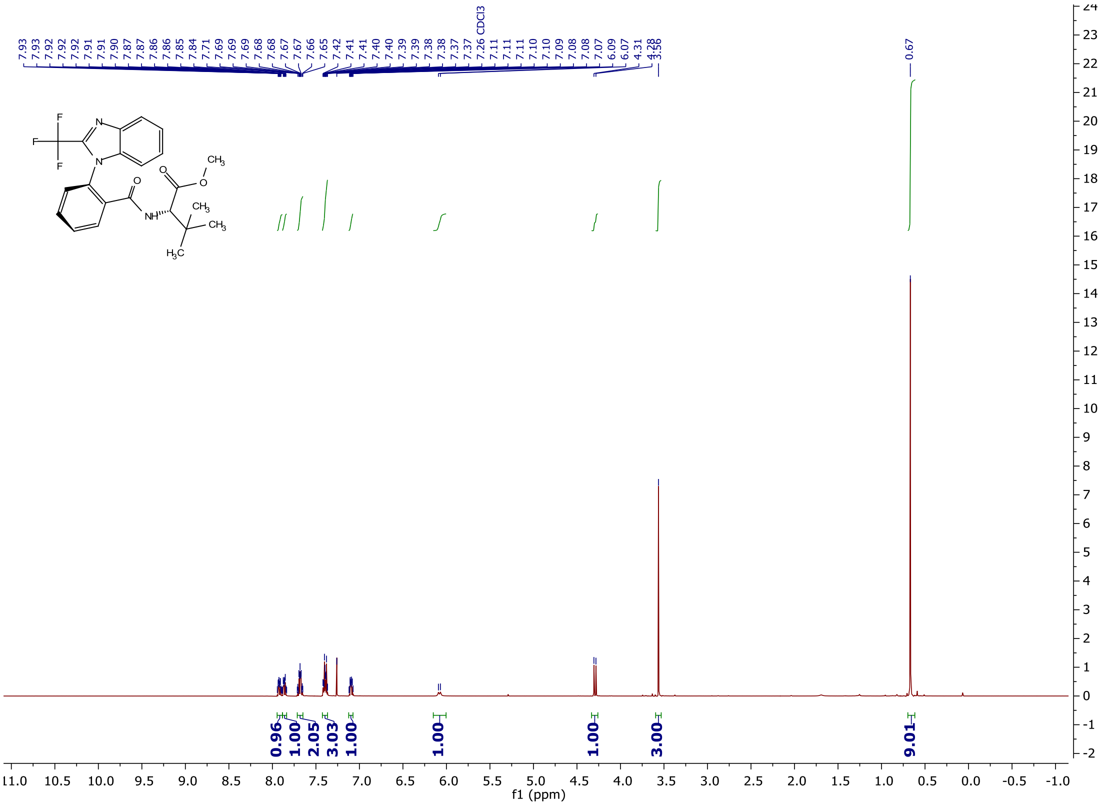

$^{13}C\{^1H\}$ NMR (101 MHz, $CDCl_3$)

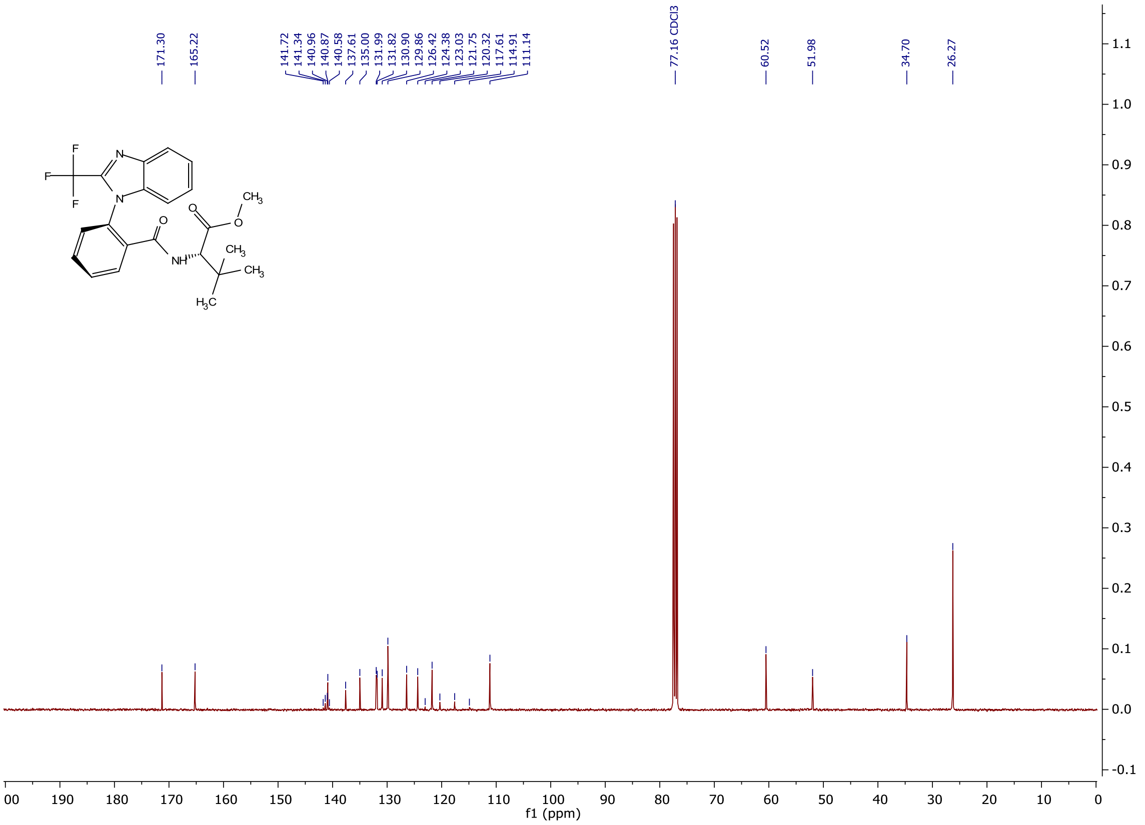

Methyl (*S*)-3,3-dimethyl-2-(2-((*M*)-2-(trifluoromethyl)-1*H*-benzo[*d*]imidazol-1-yl)benzamido)butanoate **(*M*)-16**

^{1}H NMR (400 MHz, $CDCl_3$)

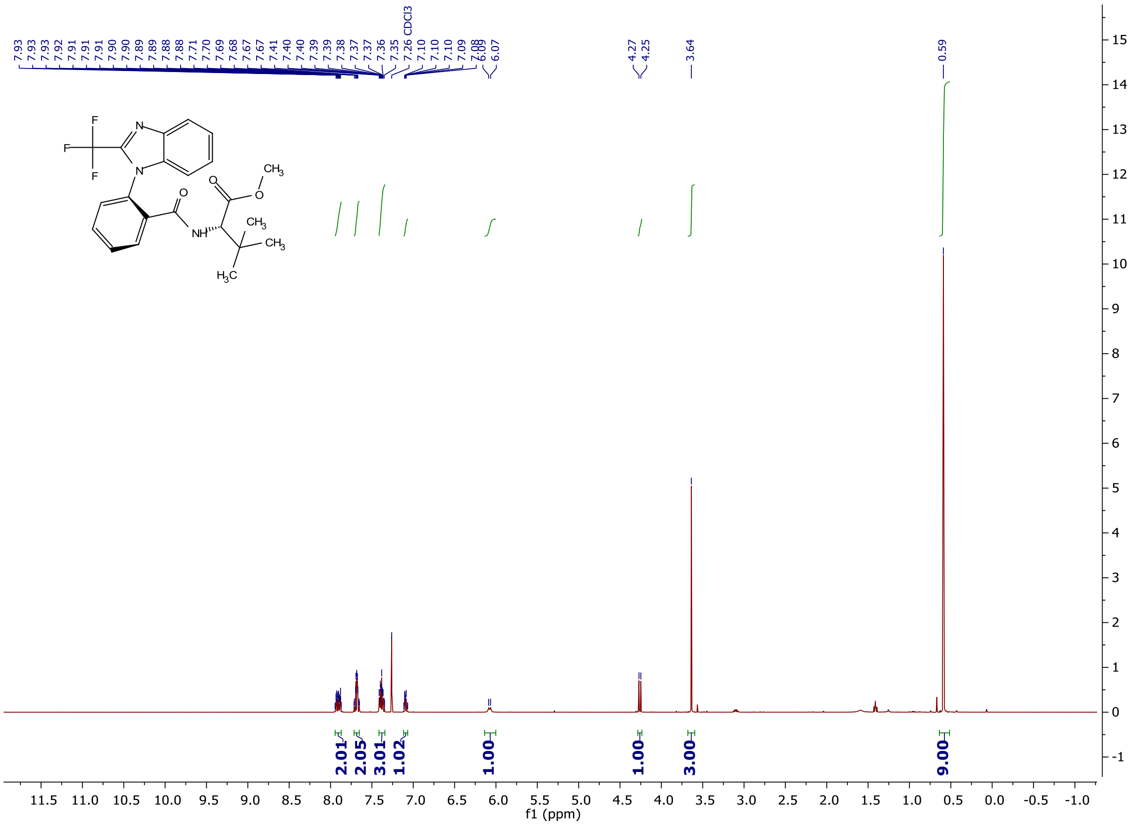

^{13}C{^{1}H} NMR (101 MHz, $CDCl_3$)

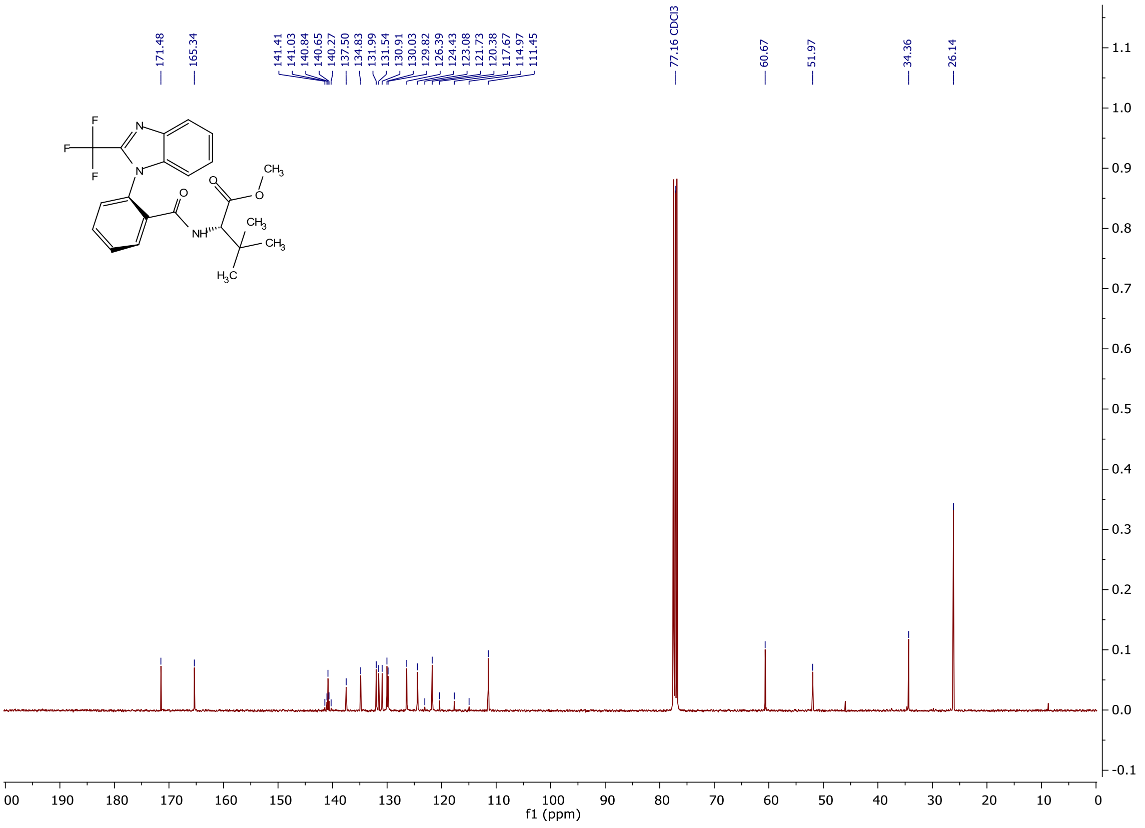

Methyl (2-((*P*)-2-(trifluoromethyl)-1*H*-benzo[*d*]imidazol-1-yl)benzoyl)-*L*-valinate **(*P*)-17**

^{1}H NMR (400 MHz, $CDCl_3$)

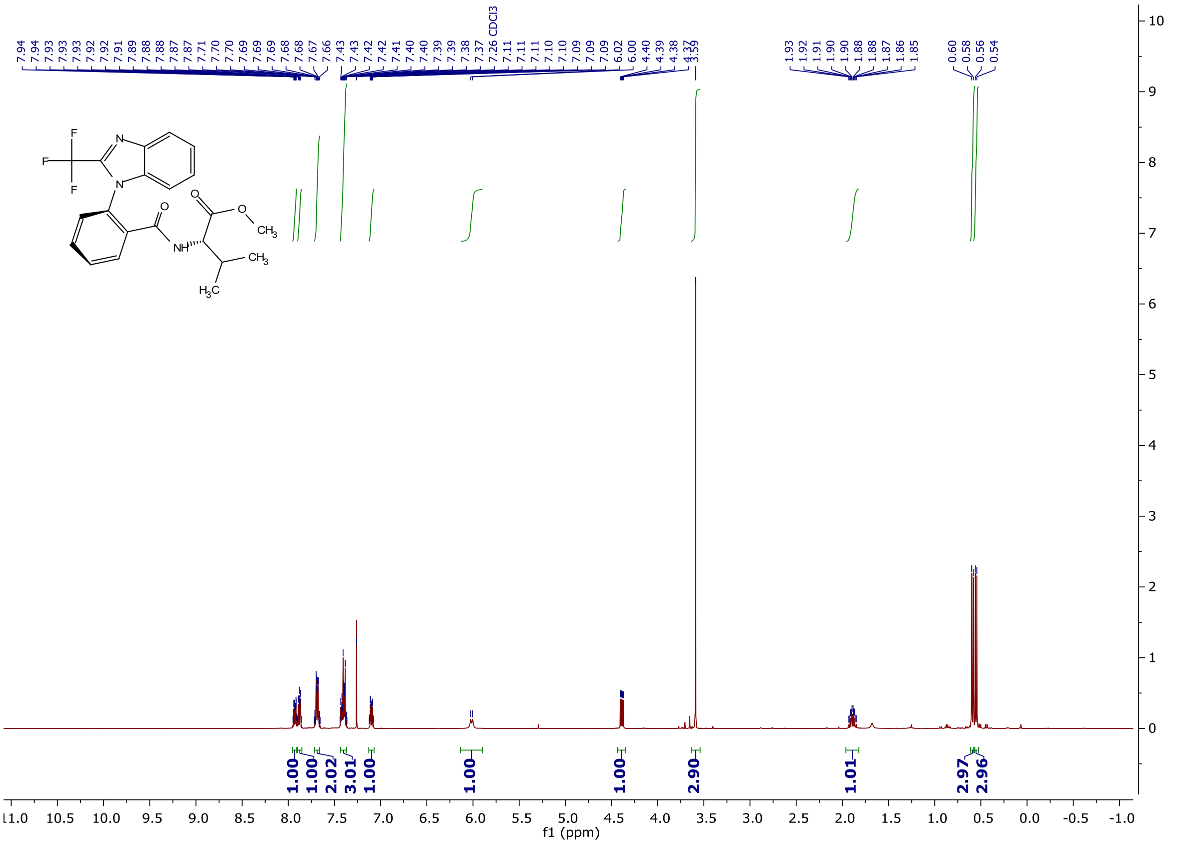

^{13}C{^{1}H} NMR (101 MHz, $CDCl_3$)

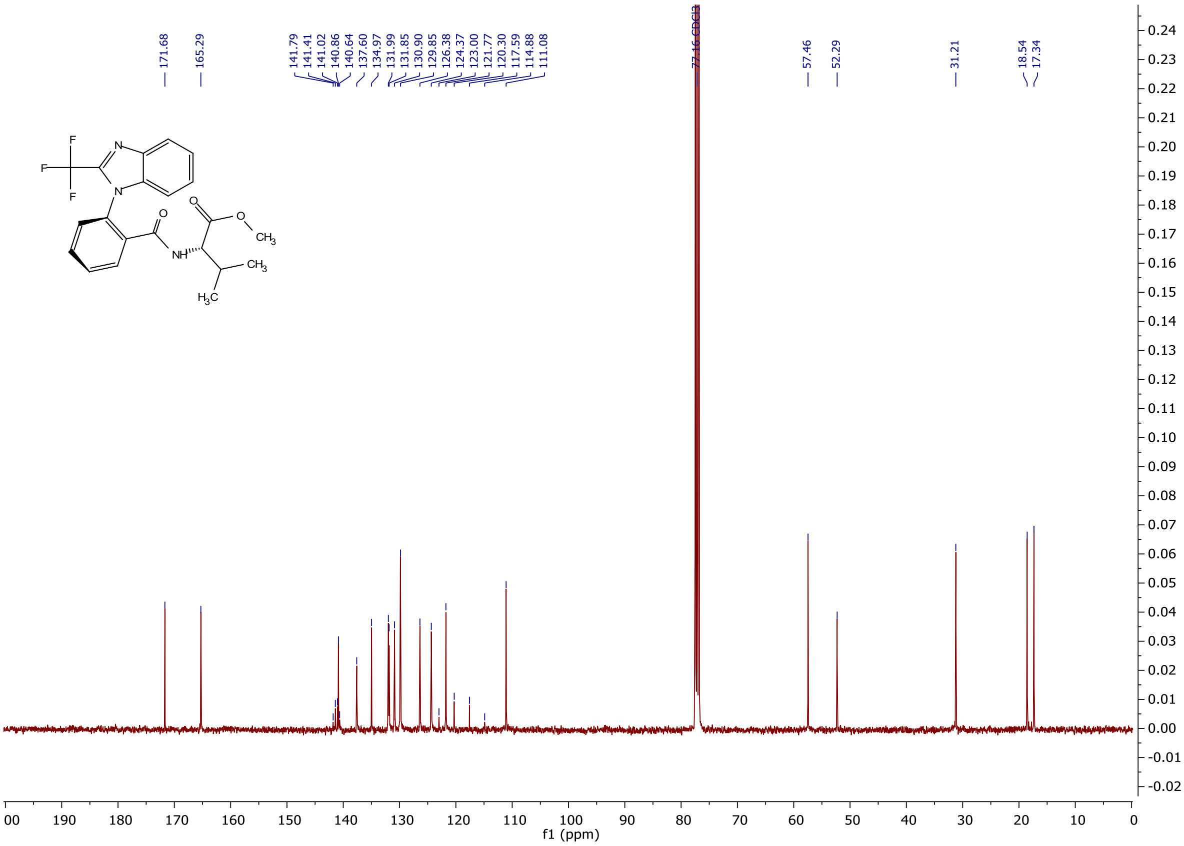

Methyl (2-((*M*)-2-(trifluoromethyl)-1*H*-benzo[*d*]imidazol-1-yl)benzoyl)-*L*-valinate **(*M*)-17**

^{1}H NMR (400 MHz, $CDCl_3$)

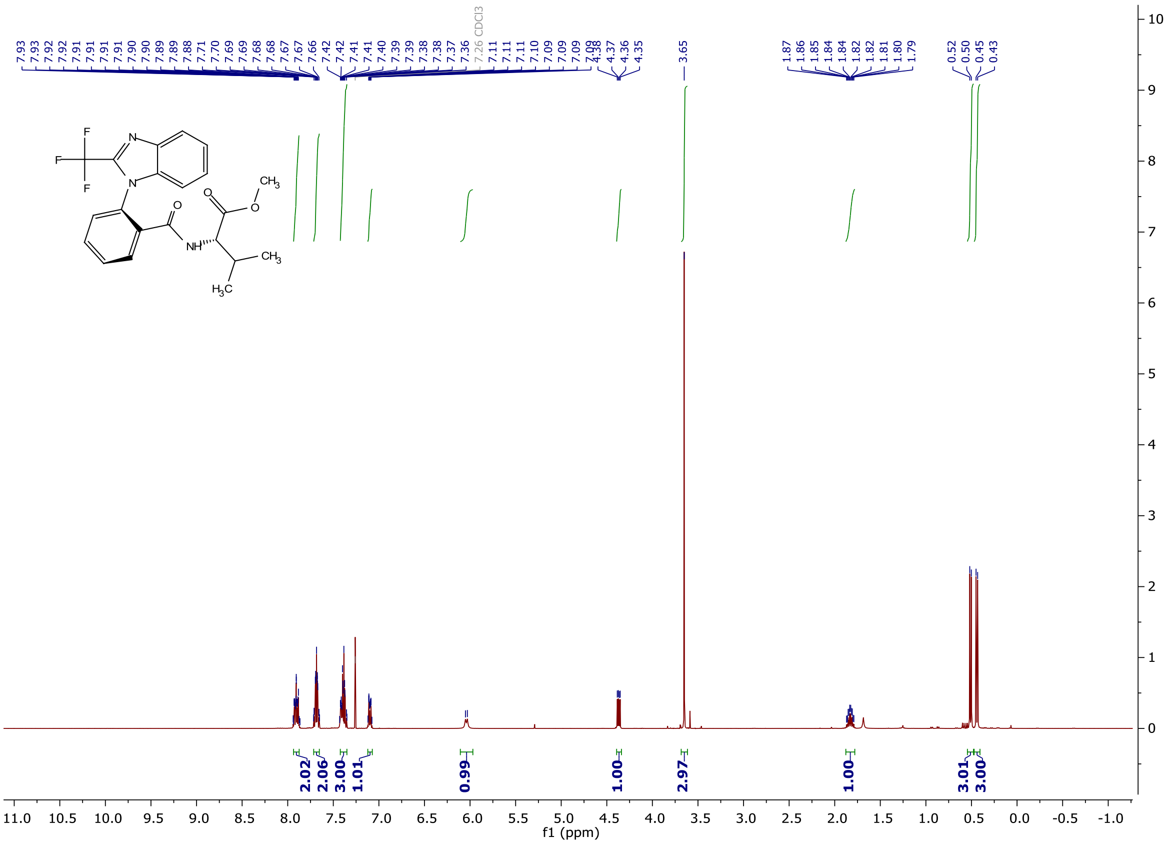

^{13}C{^{1}H} NMR (101 MHz, $CDCl_3$)

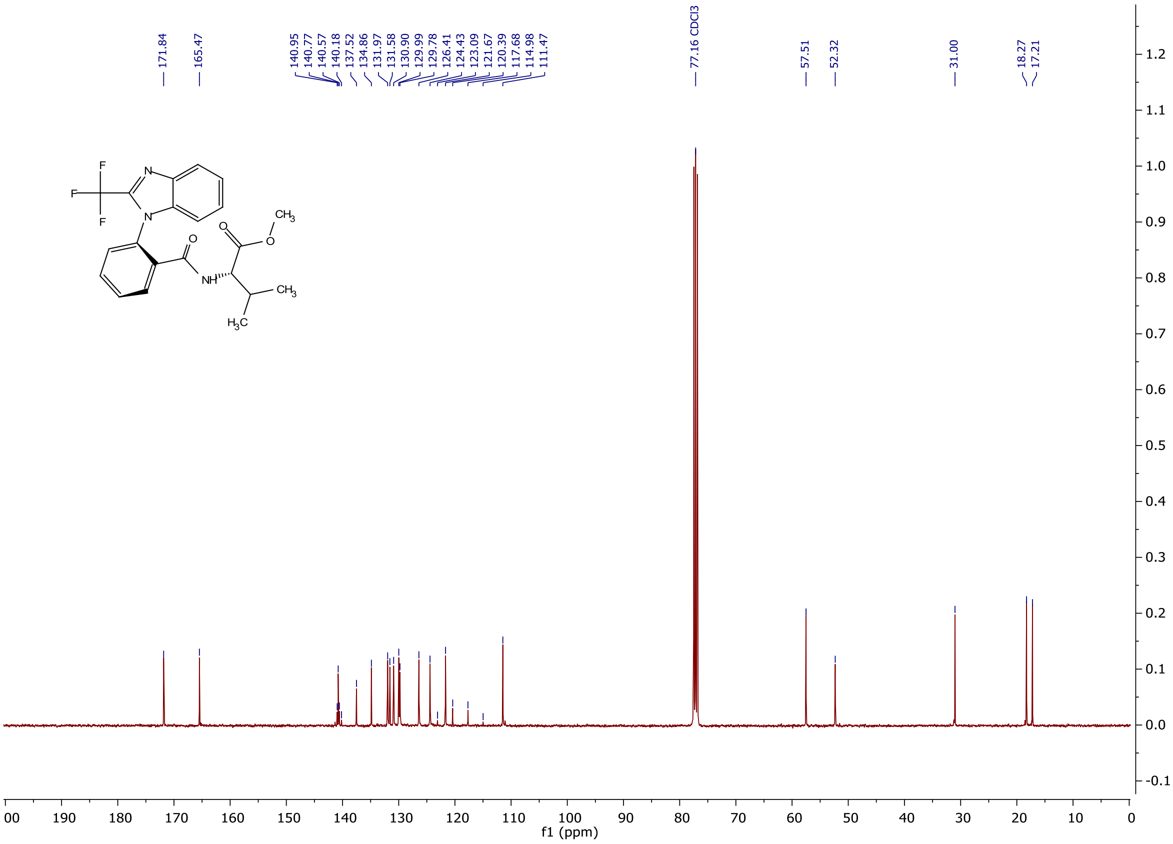

tert-Butyl *O*-(*tert*-butyl)-*N*-(2-((*P*)-2-(trifluoromethyl)-1*H*-benzo[*d*]imidazol-1-yl)benzoyl)-*L*-serinate **(*P*)-19**

^{1}H NMR (400 MHz, $CDCl_3$)

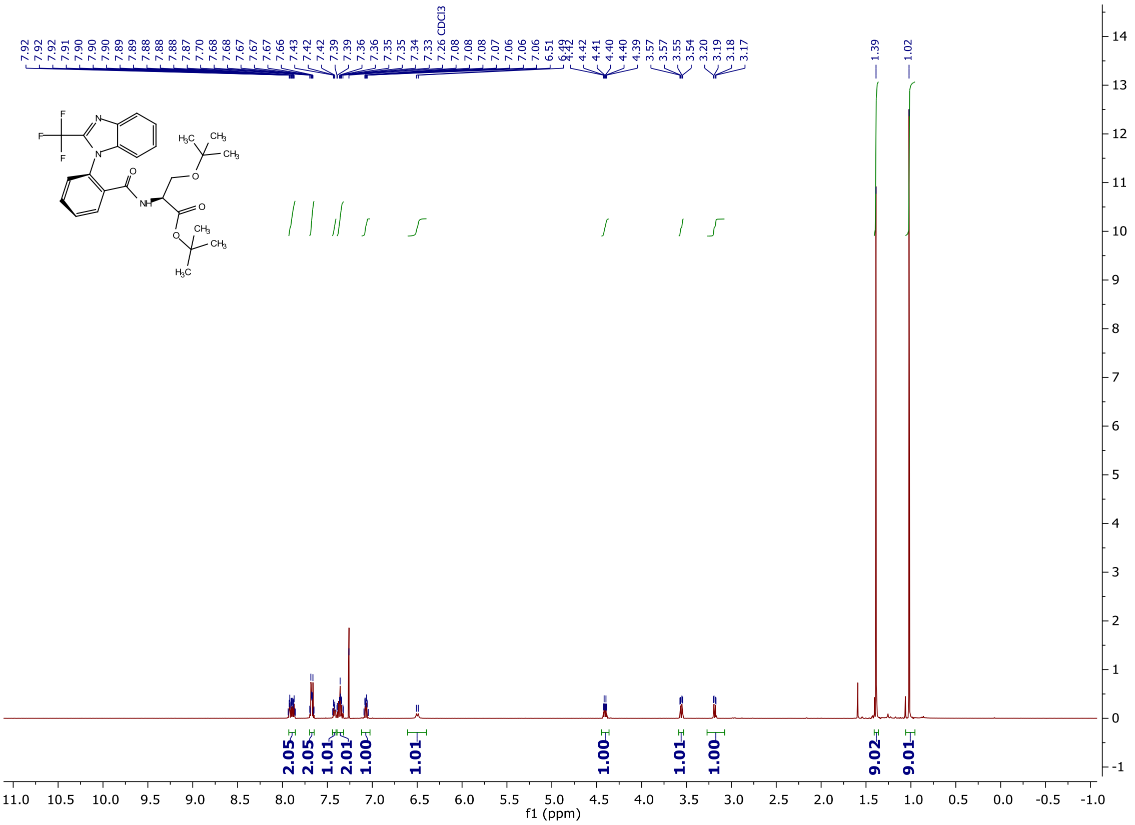

^{13}C{^{1}H} NMR (101 MHz, $CDCl_3$)

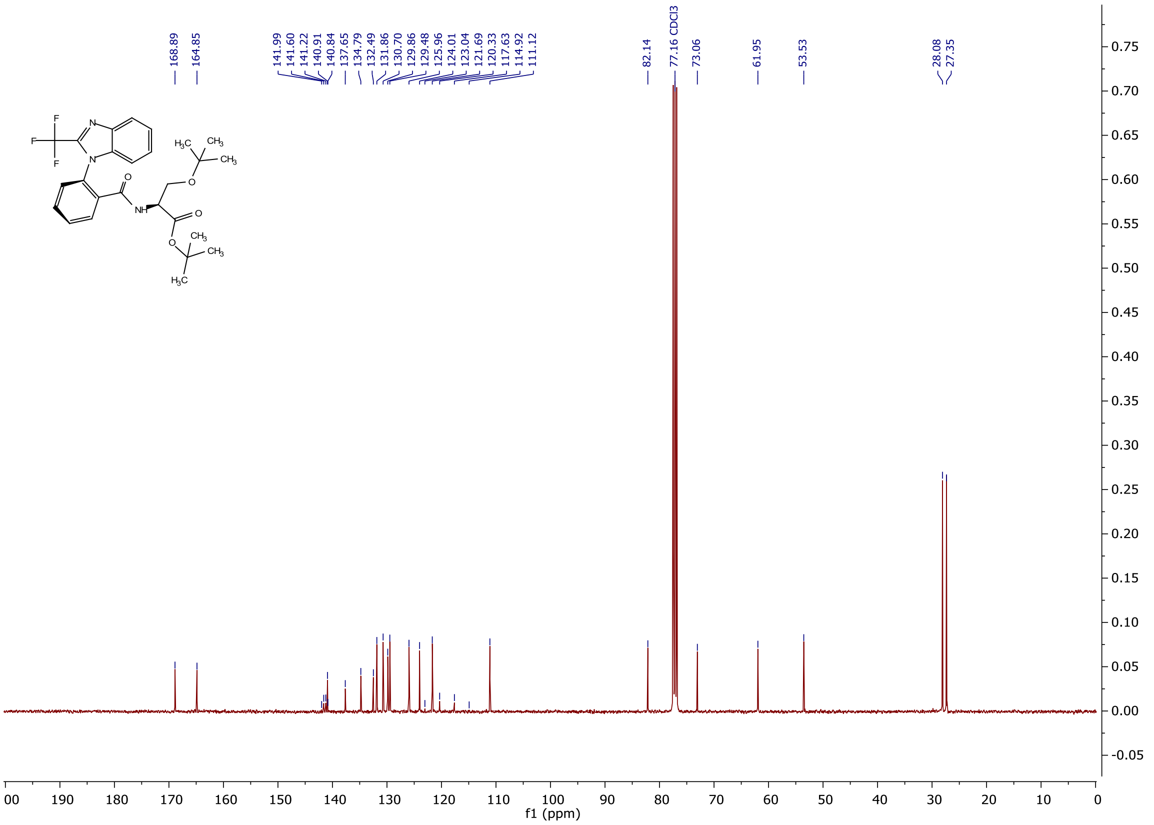

tert-Butyl *O*-(*tert*-butyl)-*N*-(2-((*M*)-2-(trifluoromethyl)-1*H*-benzo[*d*]imidazol-1-yl)benzoyl)-*L*-serinate **(*M*)-19**

^{1}H NMR (400 MHz, $CDCl_3$)

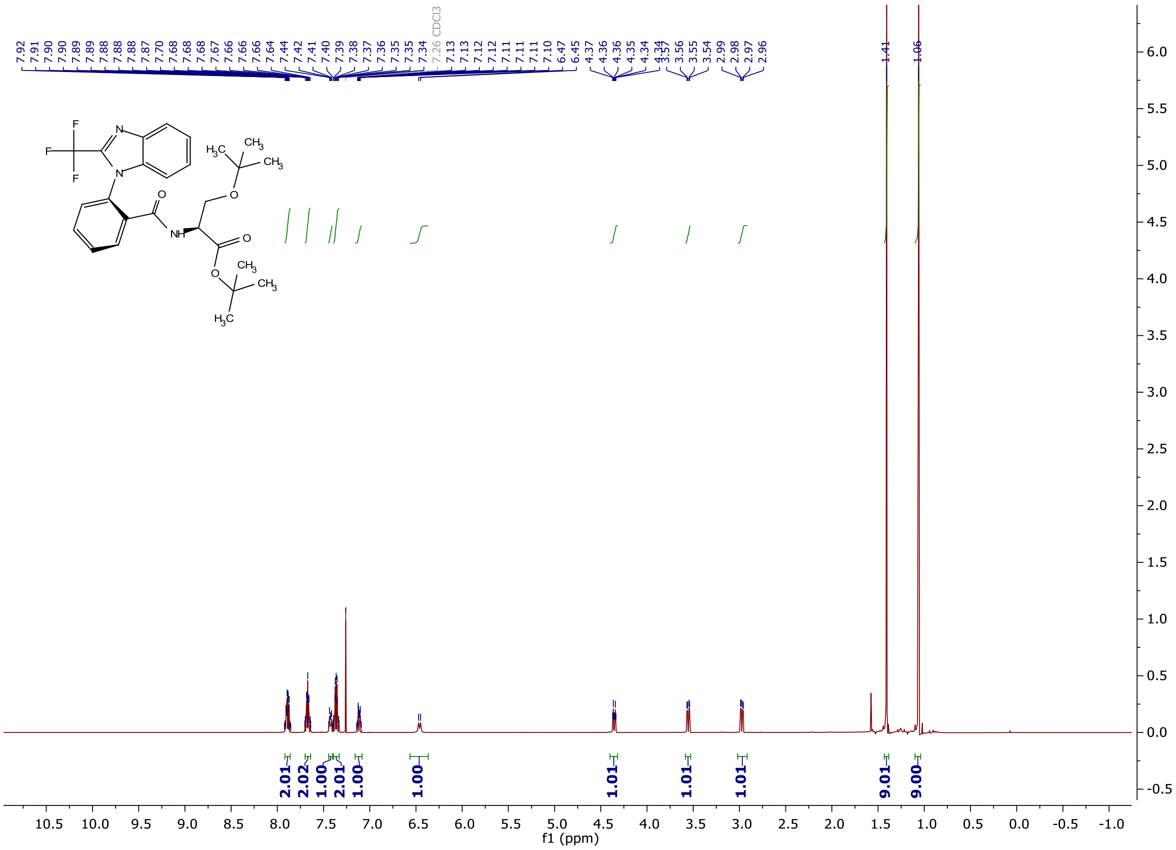

^{13}C{^{1}H} NMR (101 MHz, $CDCl_3$)

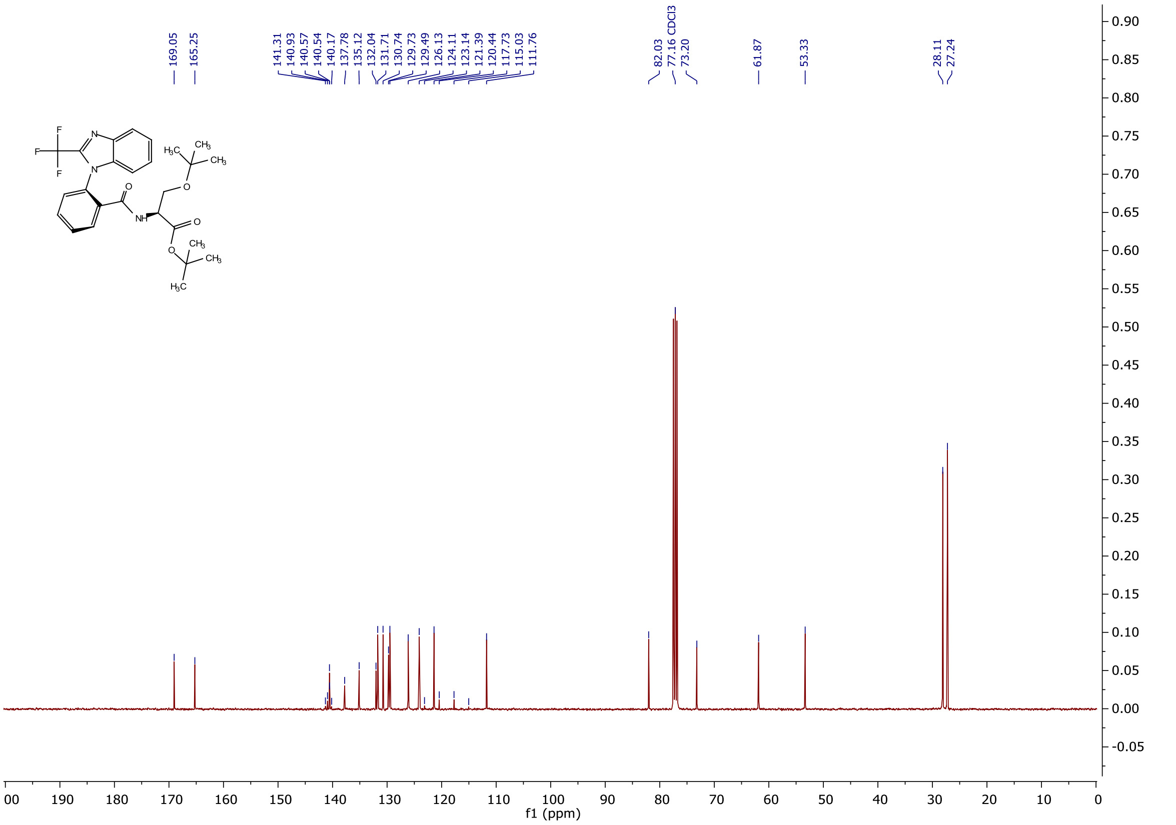

Methyl (2-((*P*)-2-(trifluoromethyl)-1*H*-benzo[*d*]imidazol-1-yl)benzoyl)-*L*-leucinate **(*P*)-20**

^{1}H NMR (400 MHz, $CDCl_3$)

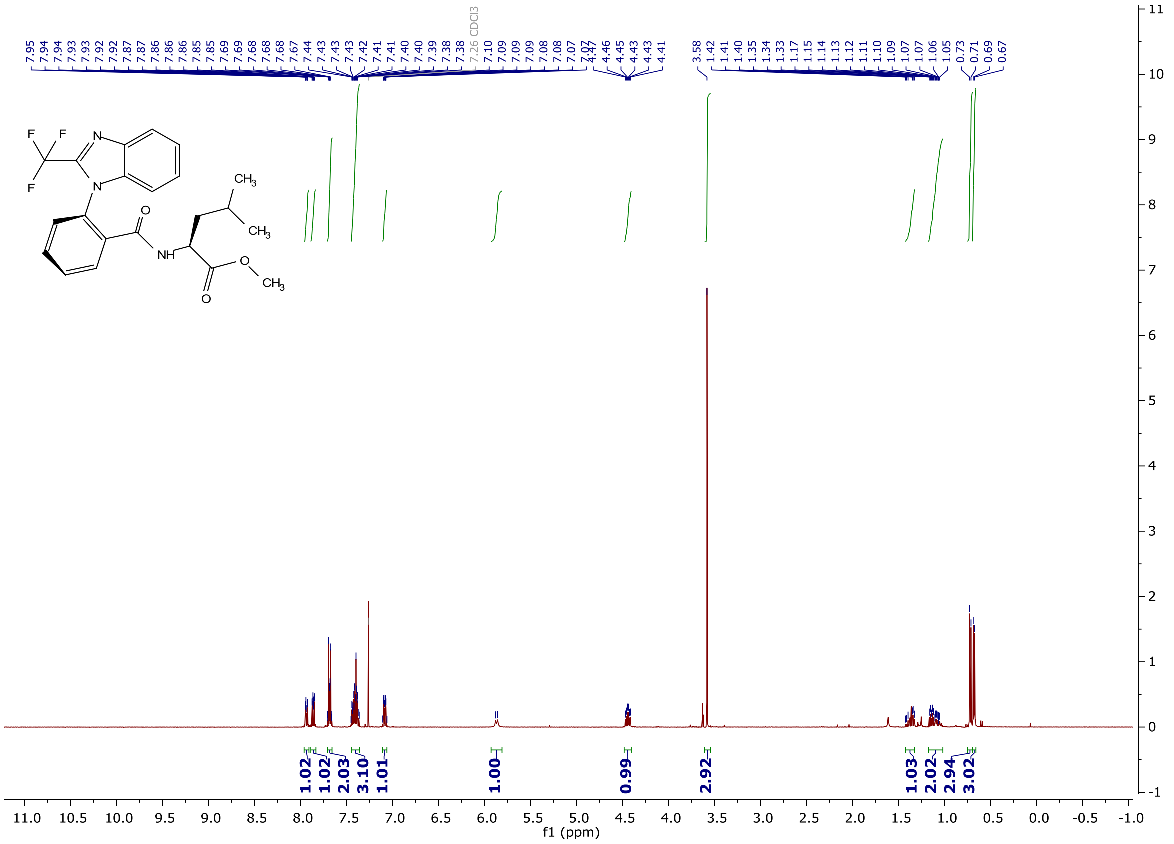

^{13}C{^{1}H} NMR (101 MHz, $CDCl_3$)

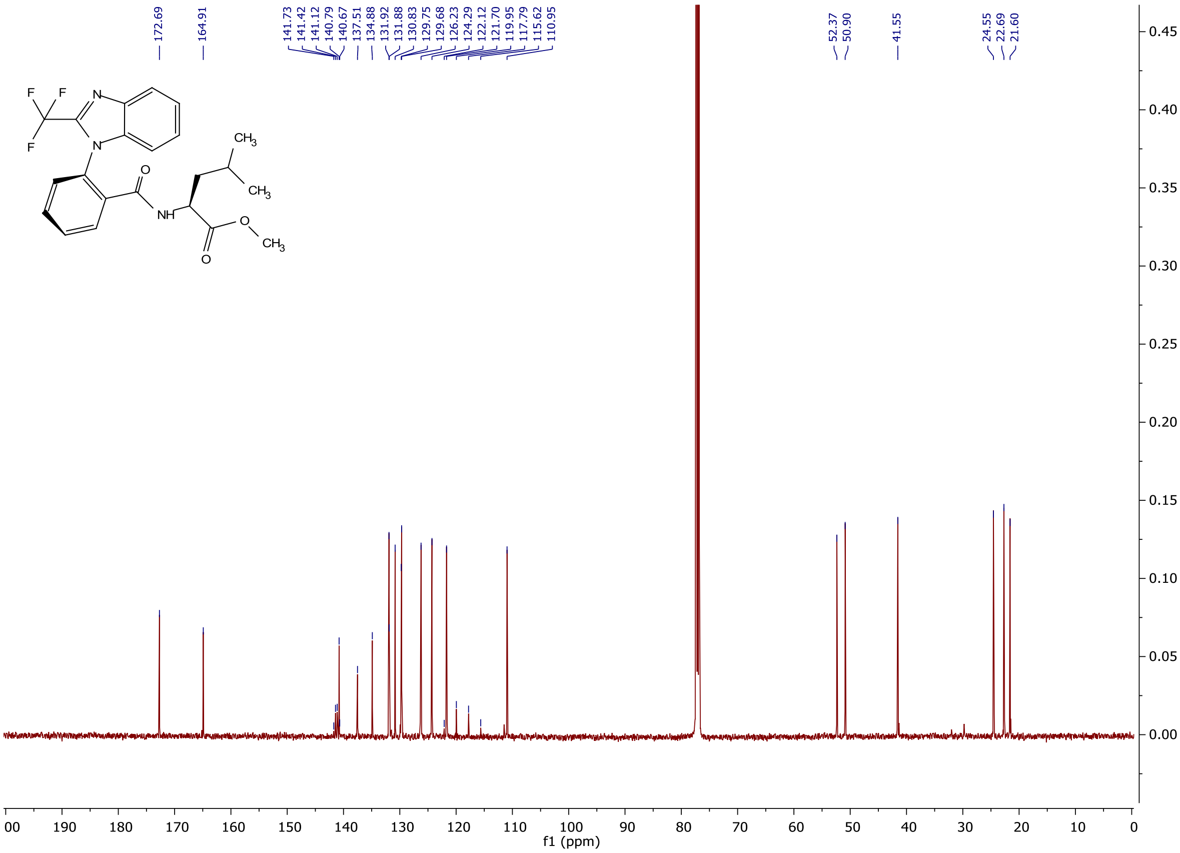

Methyl (2-((*M*)-2-(trifluoromethyl)-1*H*-benzo[*d*]imidazol-1-yl)benzoyl)-*L*-leucinate **(*M*)-20**

^{1}H NMR (400 MHz, $CDCl_3$)

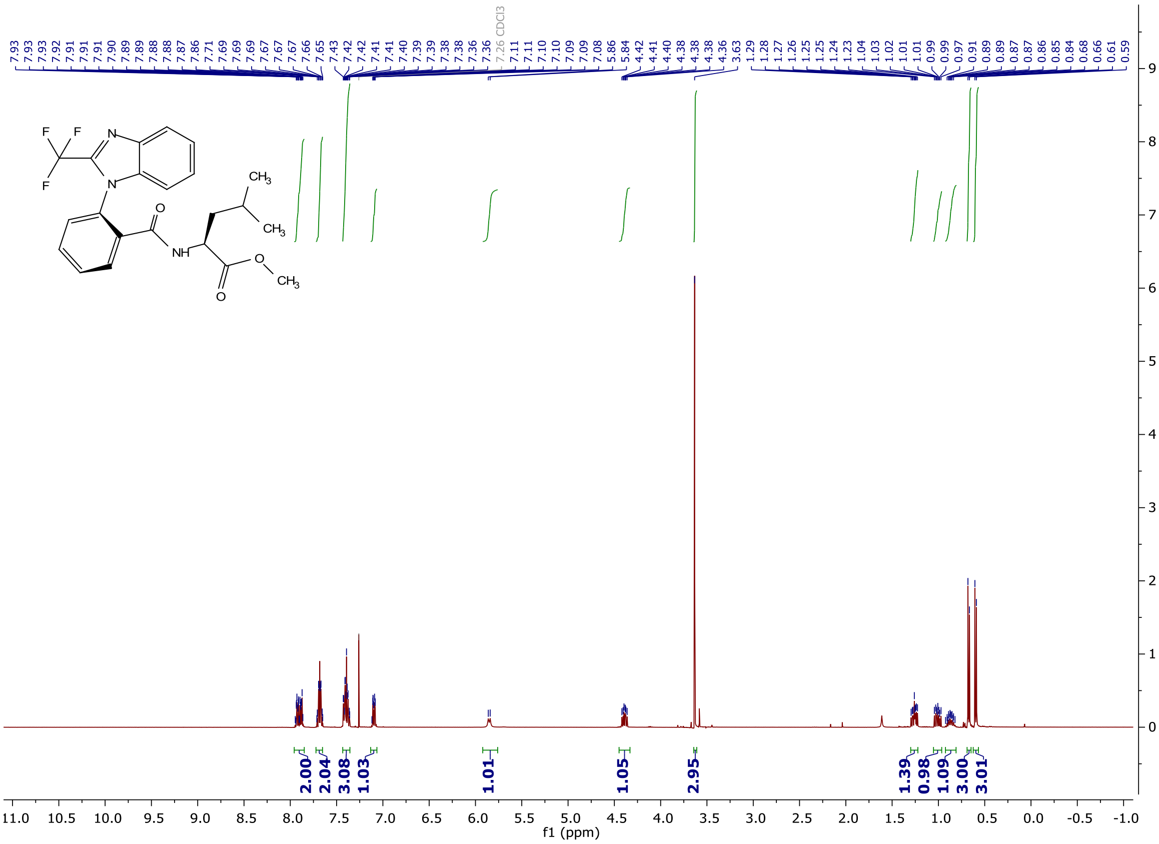

^{13}C{^{1}H} NMR (101 MHz, $CDCl_3$)

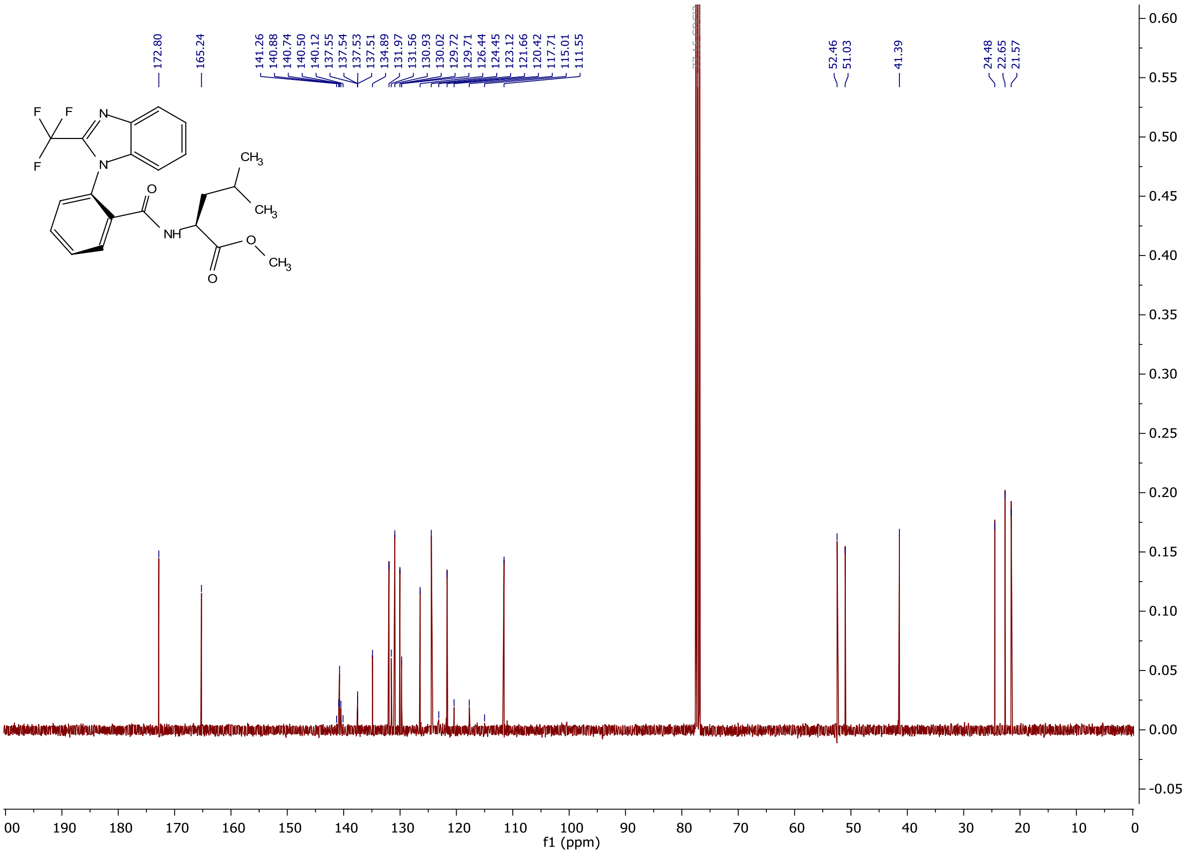

N-((*S*)-1-amino-1-oxopropan-2-yl)-2-((*P*)-2-(trifluoromethyl)-1*H*-benzo[*d*]imidazol-1-yl)benzamide **(*P*)-22**

^{1}H NMR (400 MHz, $CDCl_3$)

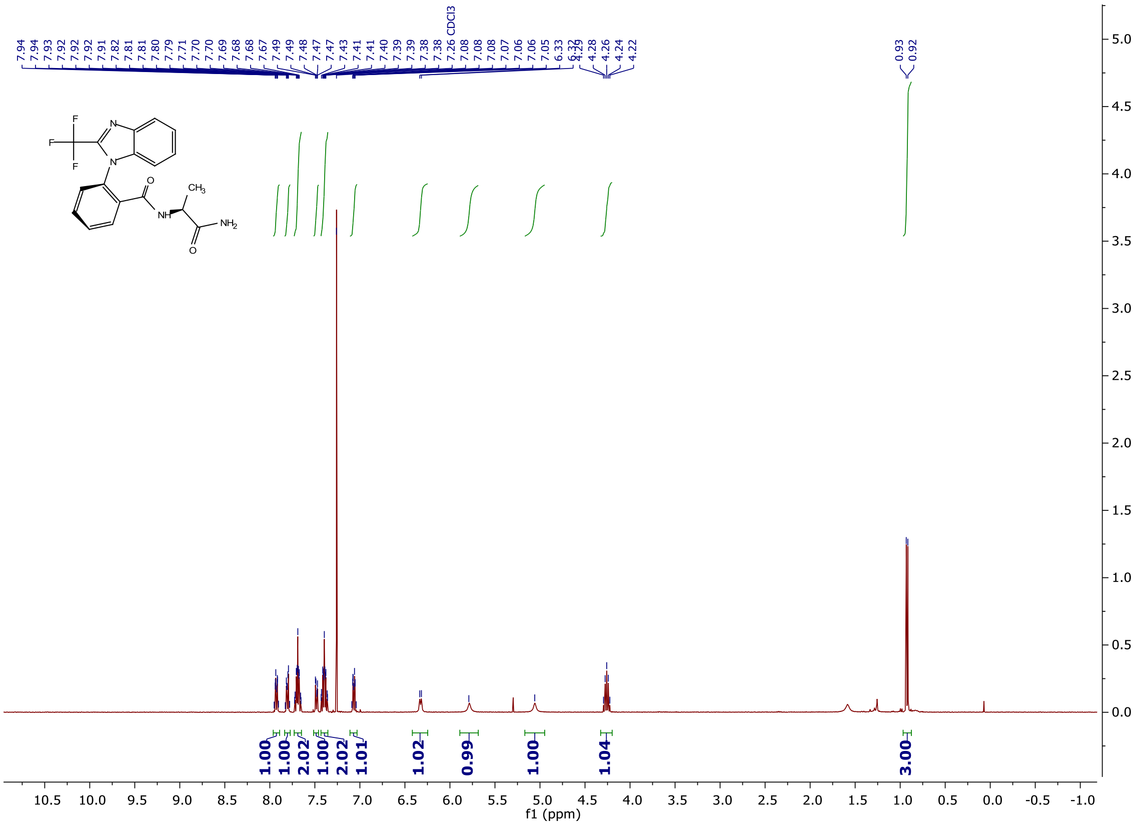

^{13}C{^{1}H} NMR (101 MHz, DMSO-d_6)

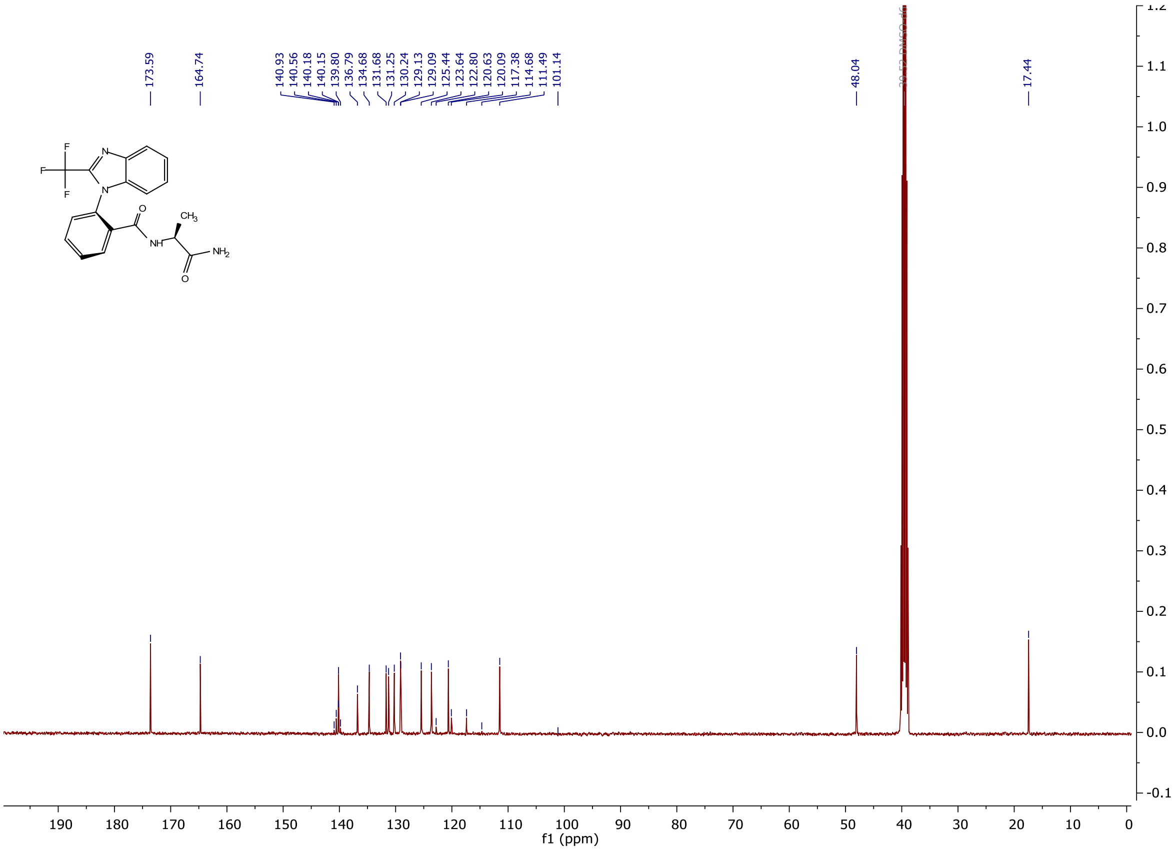

N-((*S*)-1-amino-1-oxopropan-2-yl)-2-((*M*)-2-(trifluoromethyl)-1*H*-benzo[*d*]imidazol-1-yl)benzamide **(*M*)-22**

^{1}H NMR (400 MHz, $CDCl_3$)

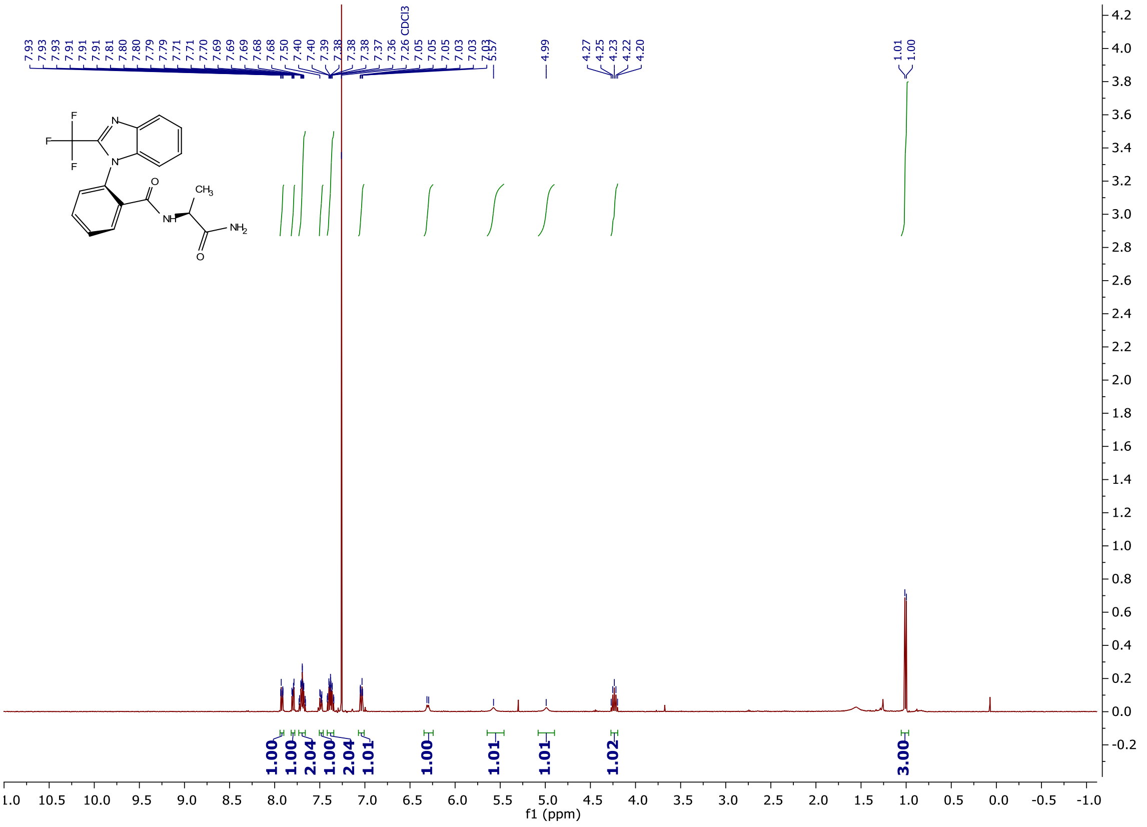

^{13}C{^{1}H} NMR (101 MHz, DMSO-d_6)

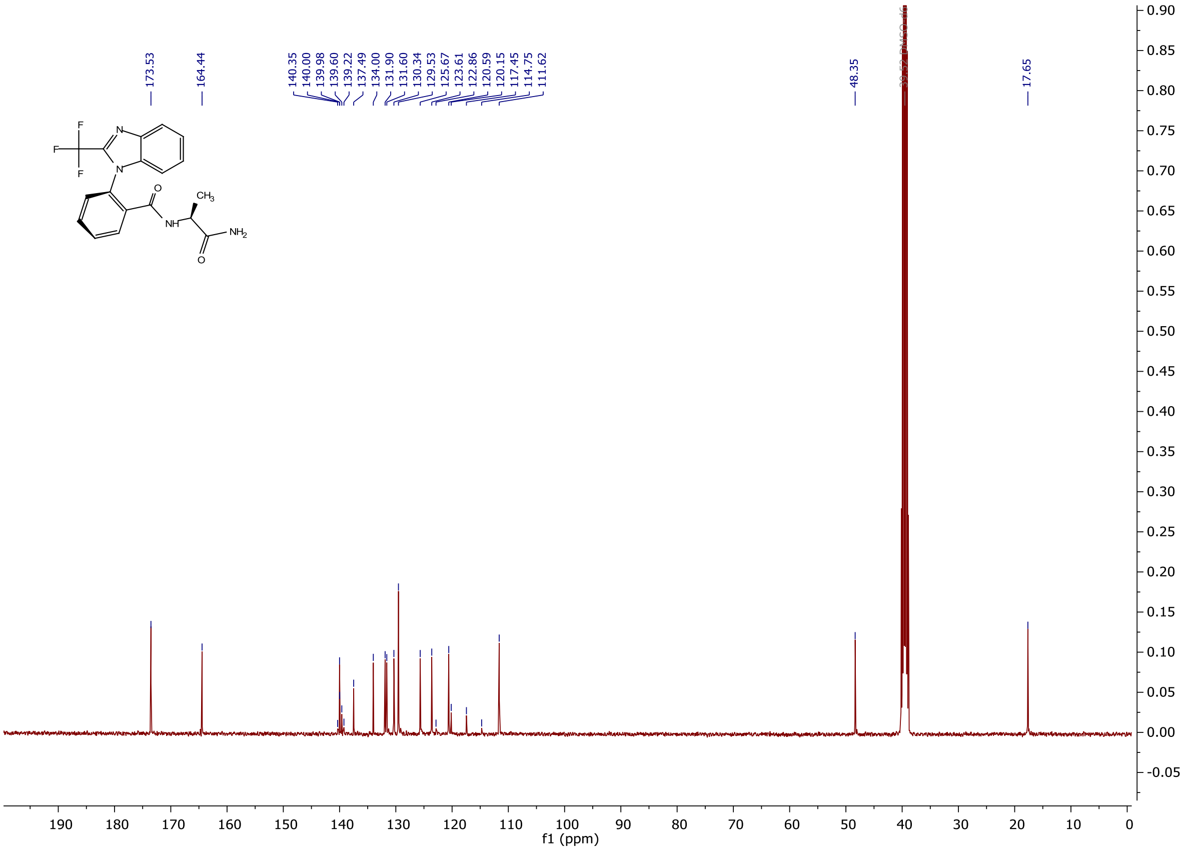

N-((*S*)-1-(Diethylamino)-1-oxopropan-2-yl)-2-((*P*)-2-(trifluoromethyl)-1*H*-benzo[*d*]imidazol-1-yl)benzamide **(*P*)-23**

^{1}**H NMR** (400 MHz, $CDCl_3$)

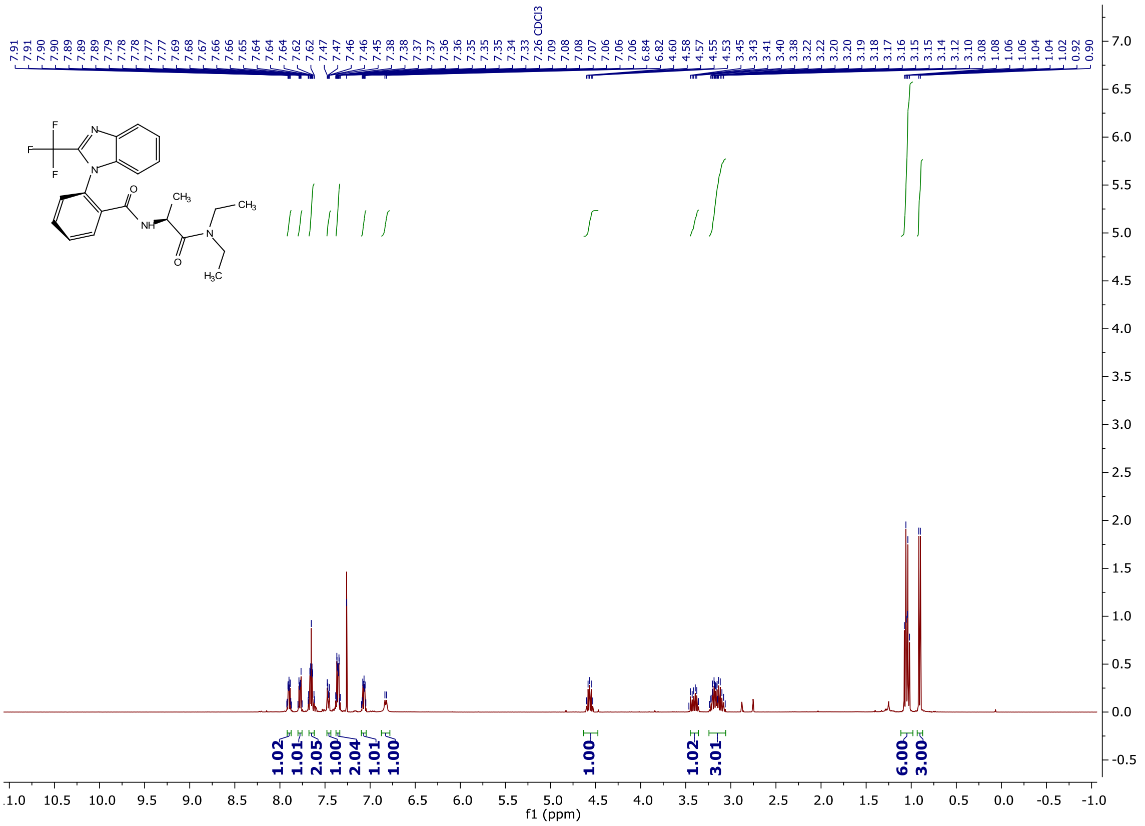

^{13}**C{^{1}H} NMR** (101 MHz, $CDCl_3$)

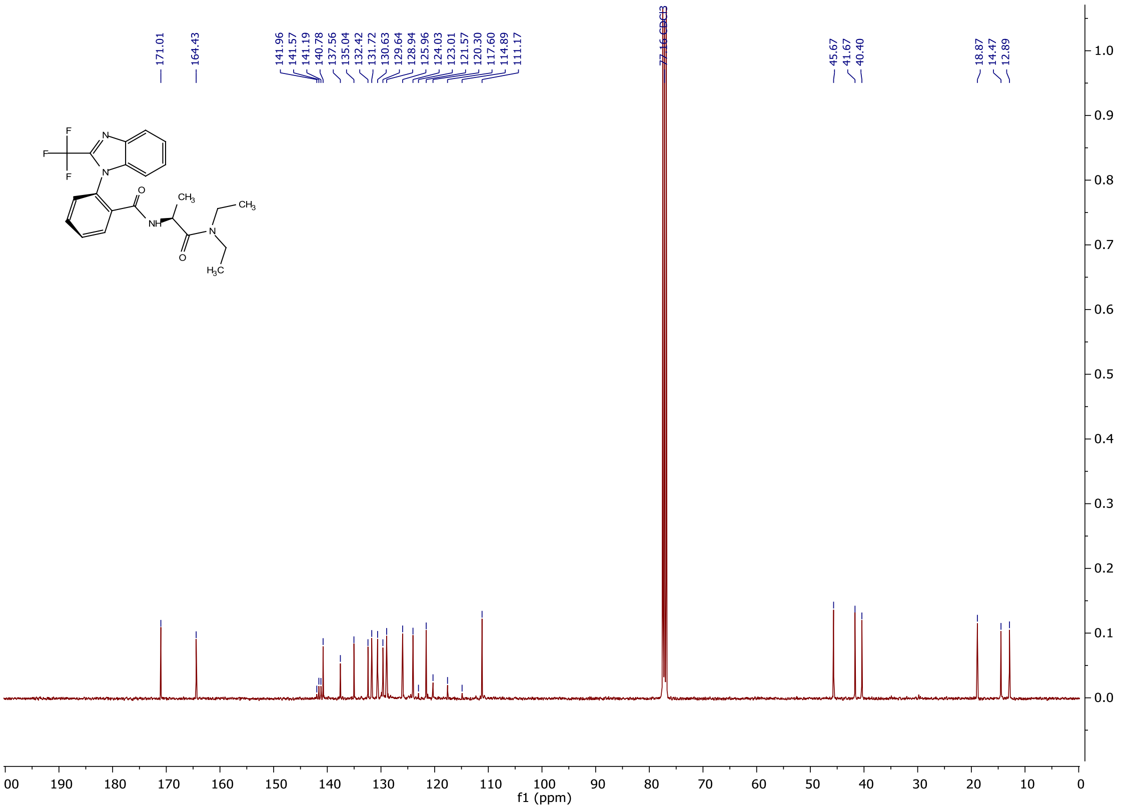

N-((*S*)-1-(Diethylamino)-1-oxopropan-2-yl)-2-((*M*)-2-(trifluoromethyl)-1*H*-benzo[*d*]imidazol-1-yl)benzamide **(*M*)-23**

^{1}H NMR (400 MHz, $CDCl_3$)

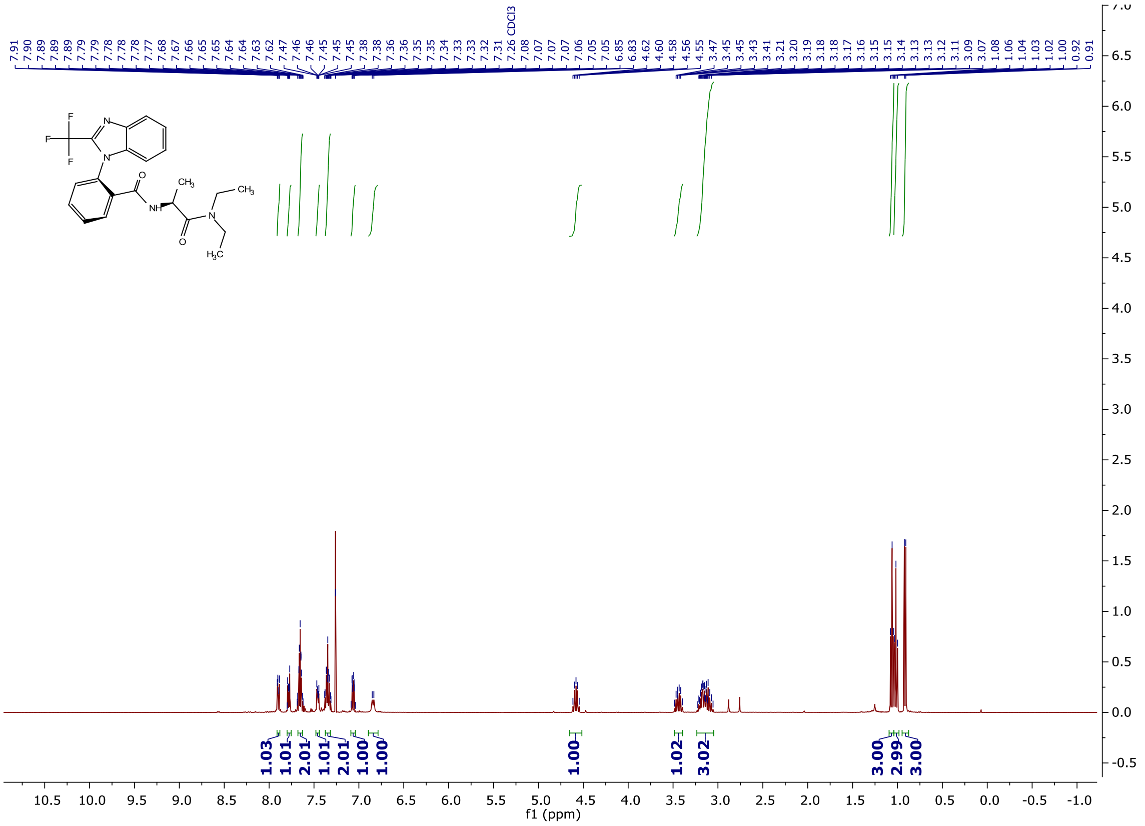

^{13}C{^{1}H} NMR (101 MHz, $CDCl_3$)

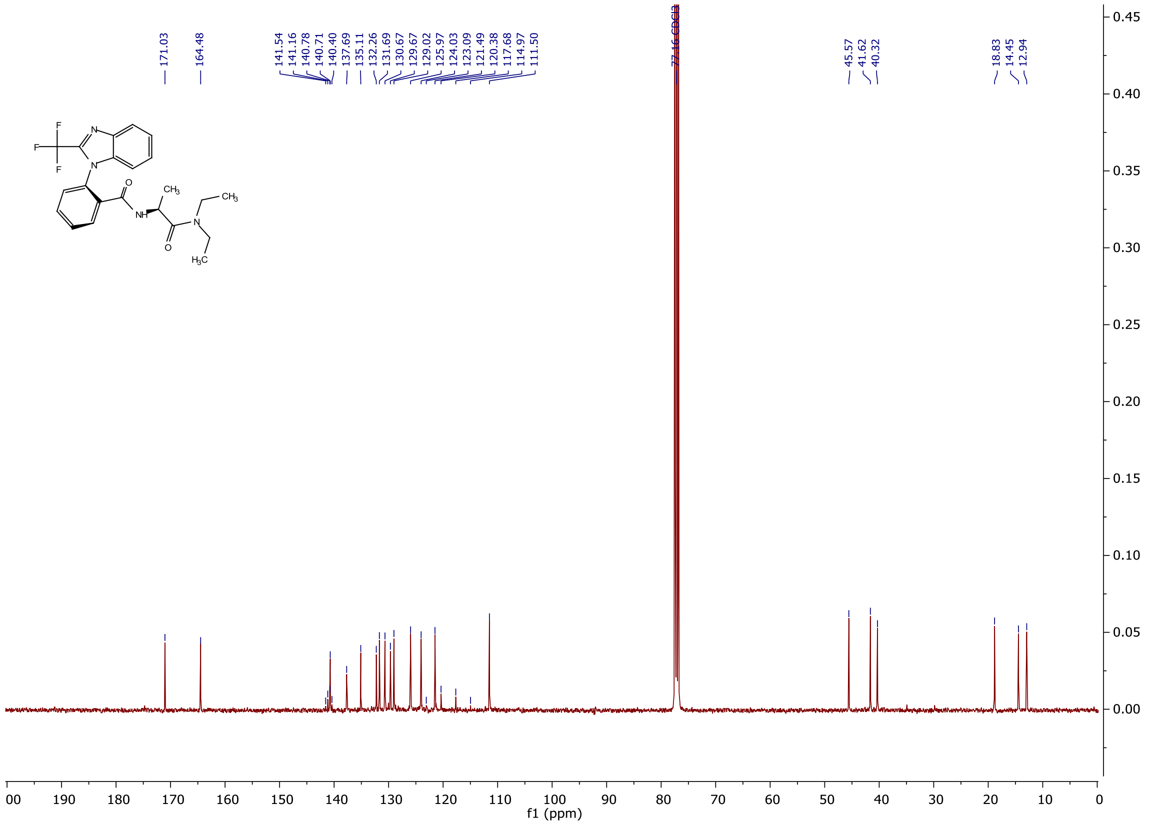

N-((*S*)-1-Cyclohexylethyl)-2-((*P*)-2-(trifluoromethyl)-1*H*-benzo[*d*]imidazol-1-yl)benzamide **(*P*)-25**

^{1}H NMR (400 MHz, $CDCl_3$)

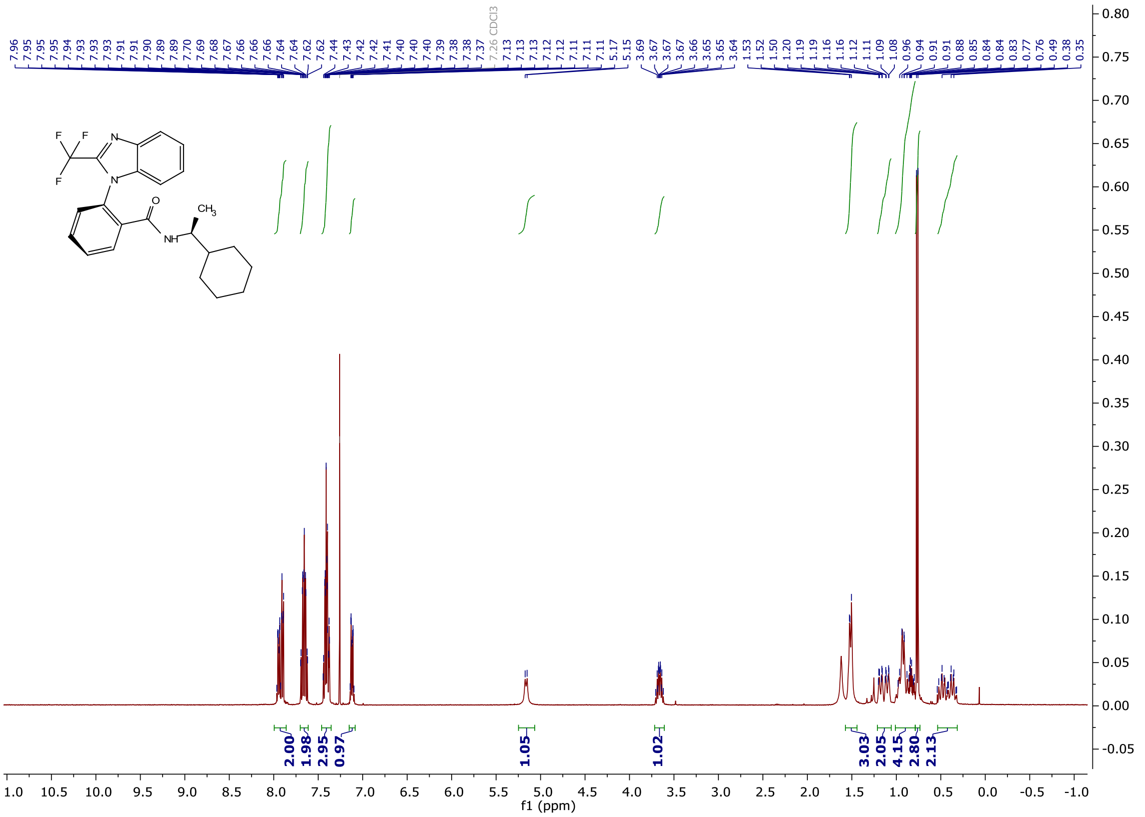

^{13}C{^{1}H} NMR (101 MHz, $CDCl_3$)

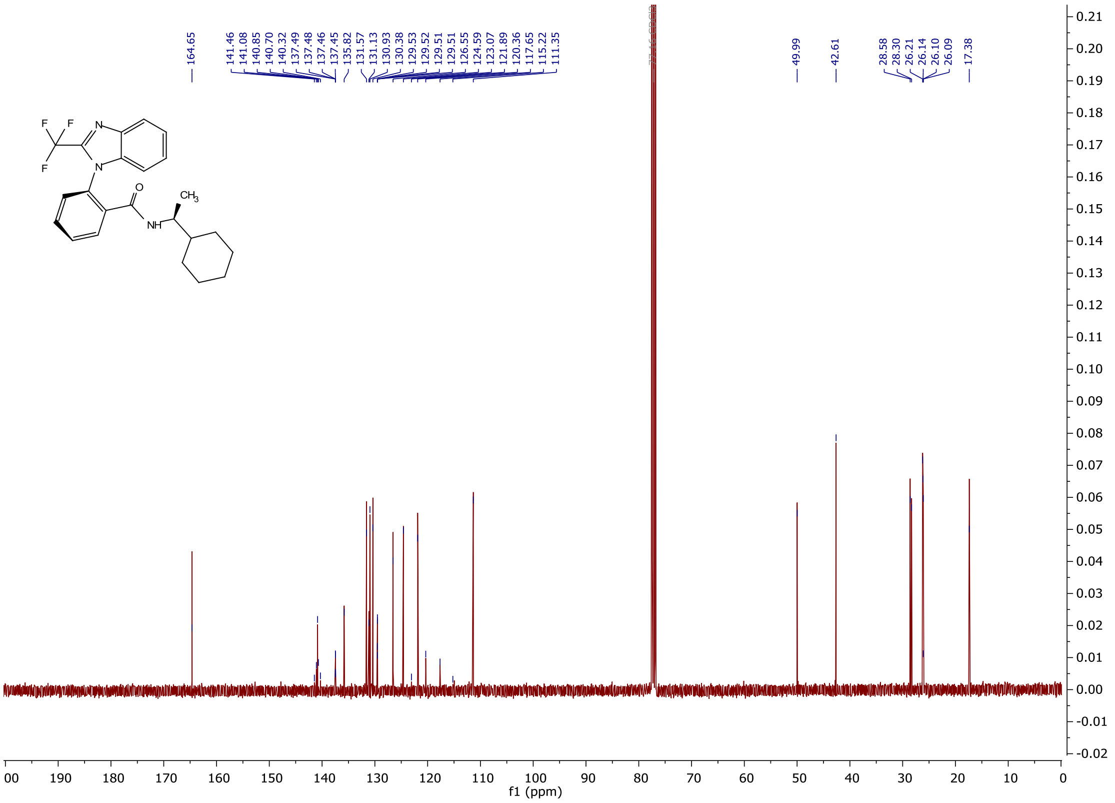

N-((*S*)-1-Cyclohexylethyl)-2-((*P*)-2-(trifluoromethyl)-1*H*-benzo[*d*]imidazol-1-yl)benzamide **(*M*)-25**

^{1}H NMR (400 MHz, $CDCl_3$)

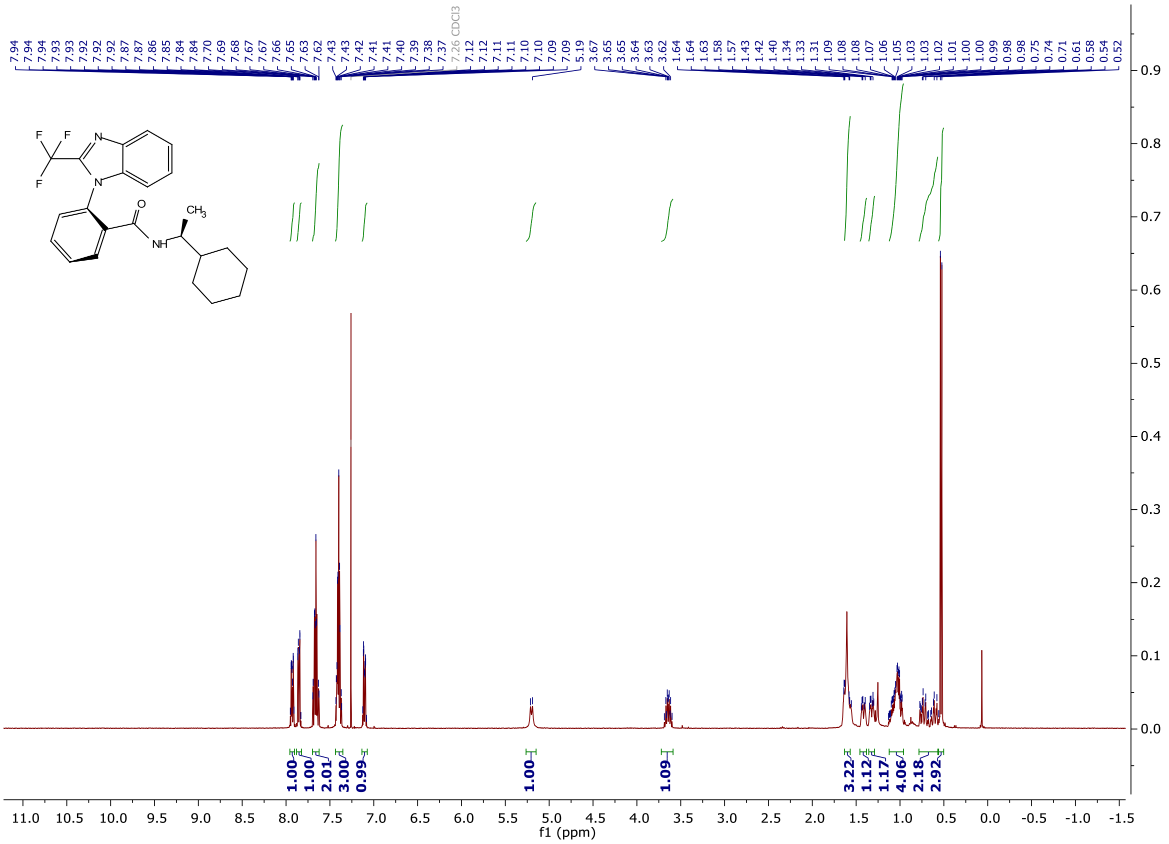

^{13}C{^{1}H} NMR (101 MHz, $CDCl_3$)

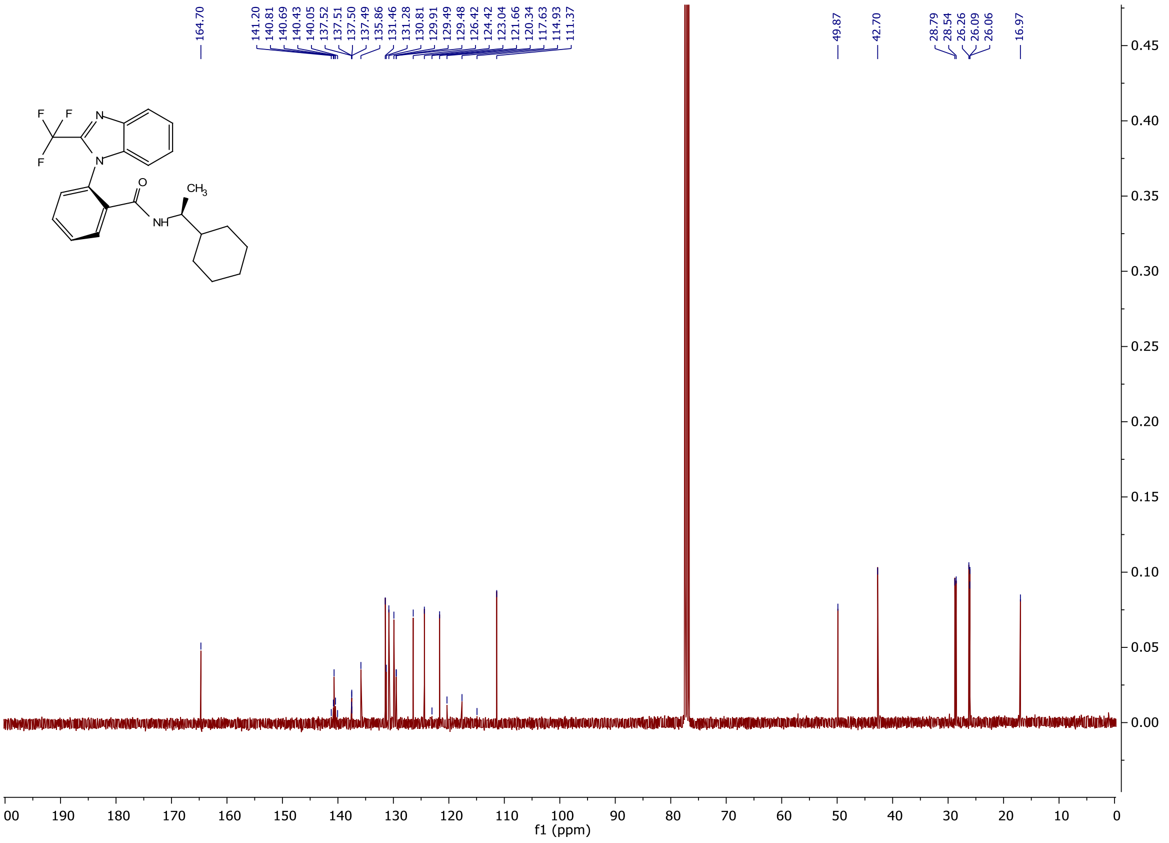

N-((*S*)-2-Methyl-1-phenylpropyl)-2-((*P*)-2-(trifluoromethyl)-1*H*-benzo[*d*]imidazol-1-yl)benzamide **(*P*)-26**

^{1}H NMR (400 MHz, $CDCl_3$)

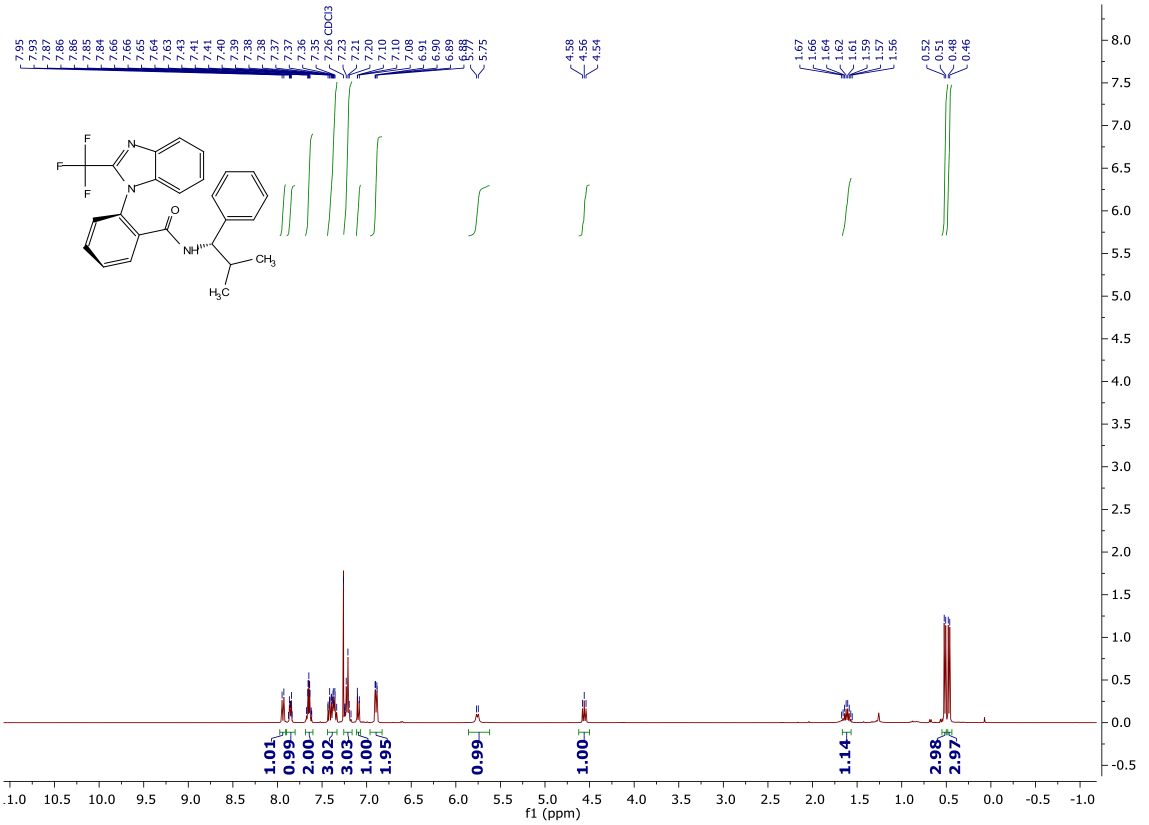

^{13}C{^{1}H} NMR (101 MHz, $CDCl_3$)

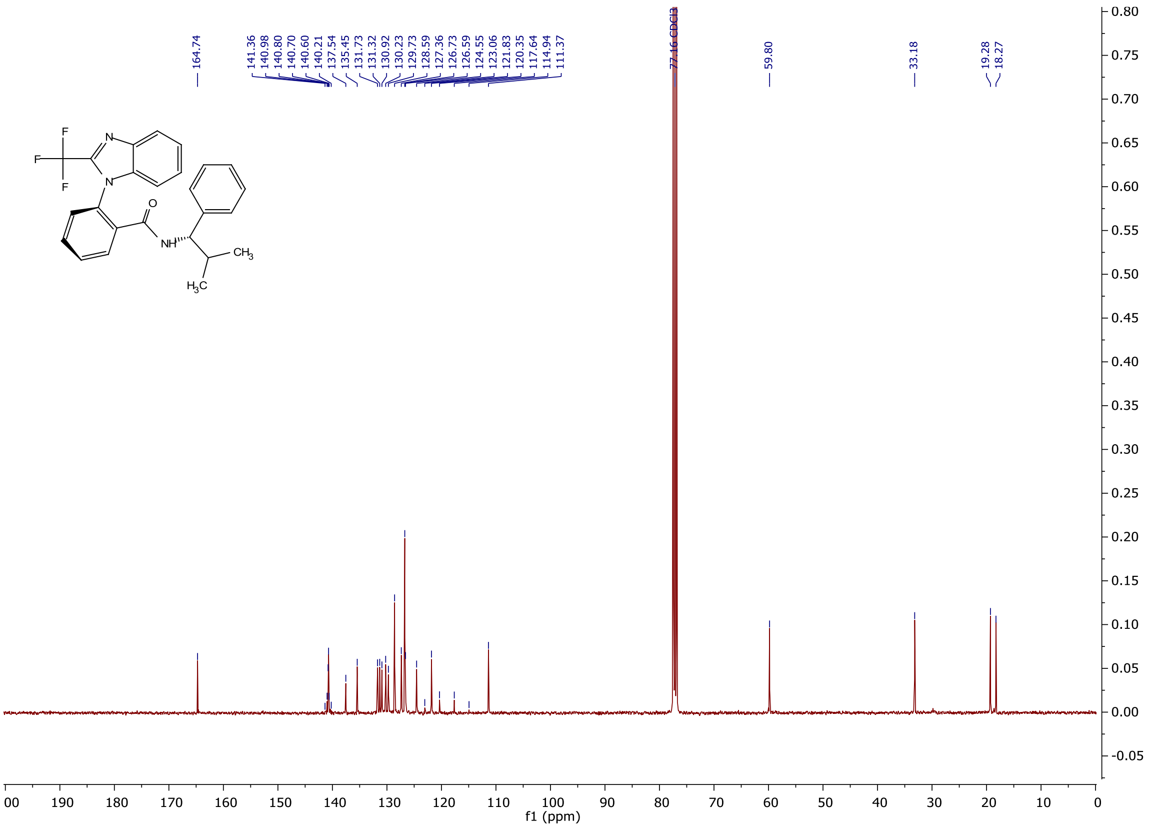

N-((*S*)-2-Methyl-1-phenylpropyl)-2-((*M*)-2-(trifluoromethyl)-1*H*-benzo[*d*]imidazol-1-yl)benzamide **(*M*)-26**

^{1}H NMR (400 MHz, $CDCl_3$)

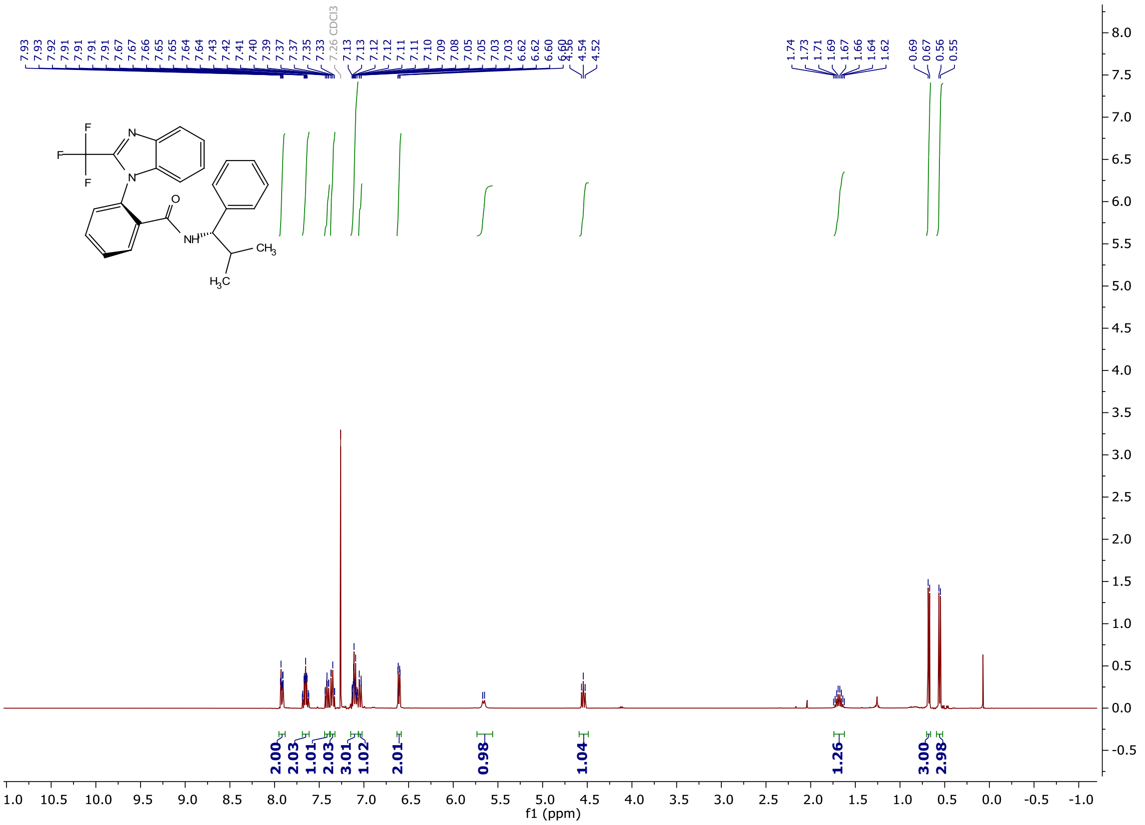

^{13}C{^{1}H} NMR (101 MHz, $CDCl_3$)

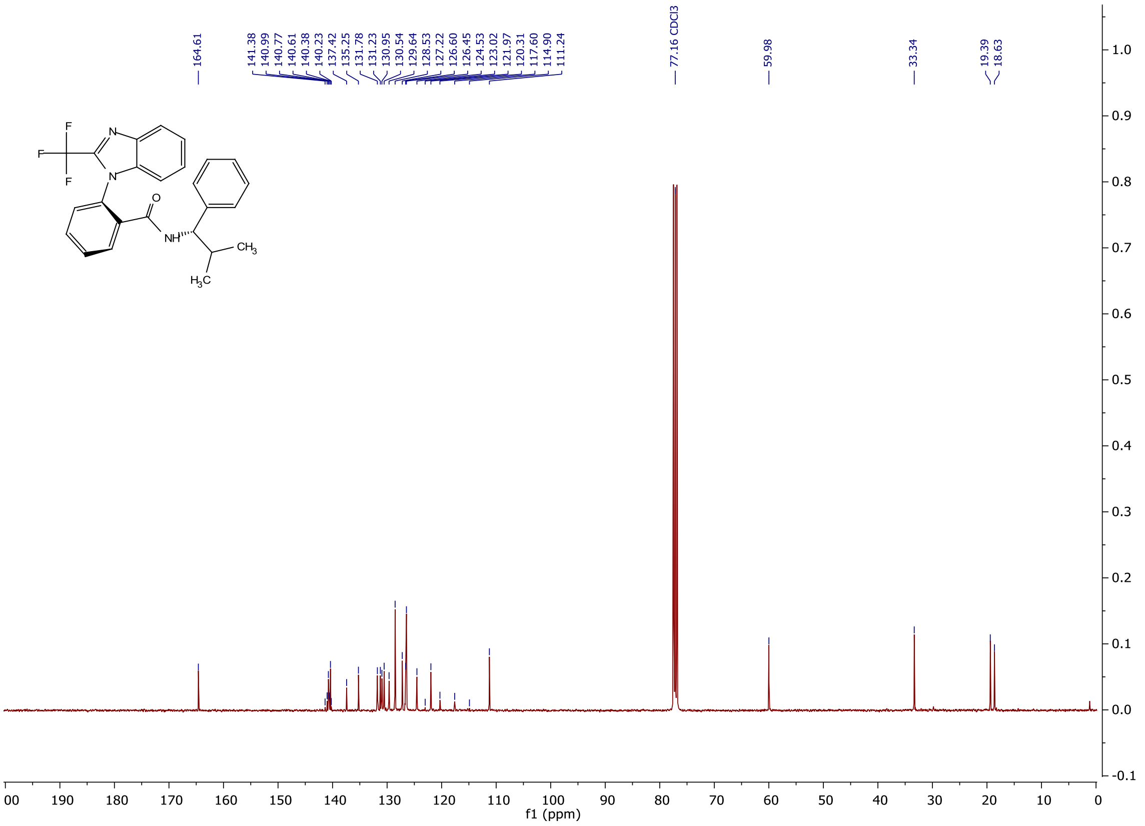

N-((*R*)-1-Phenylpropan-2-yl)-2-((*P*)-2-(trifluoromethyl)-1*H*-benzo[*d*]imidazol-1-yl)benzamide **(*P*)-29**

^{1}H NMR (400 MHz, $CDCl_3$)

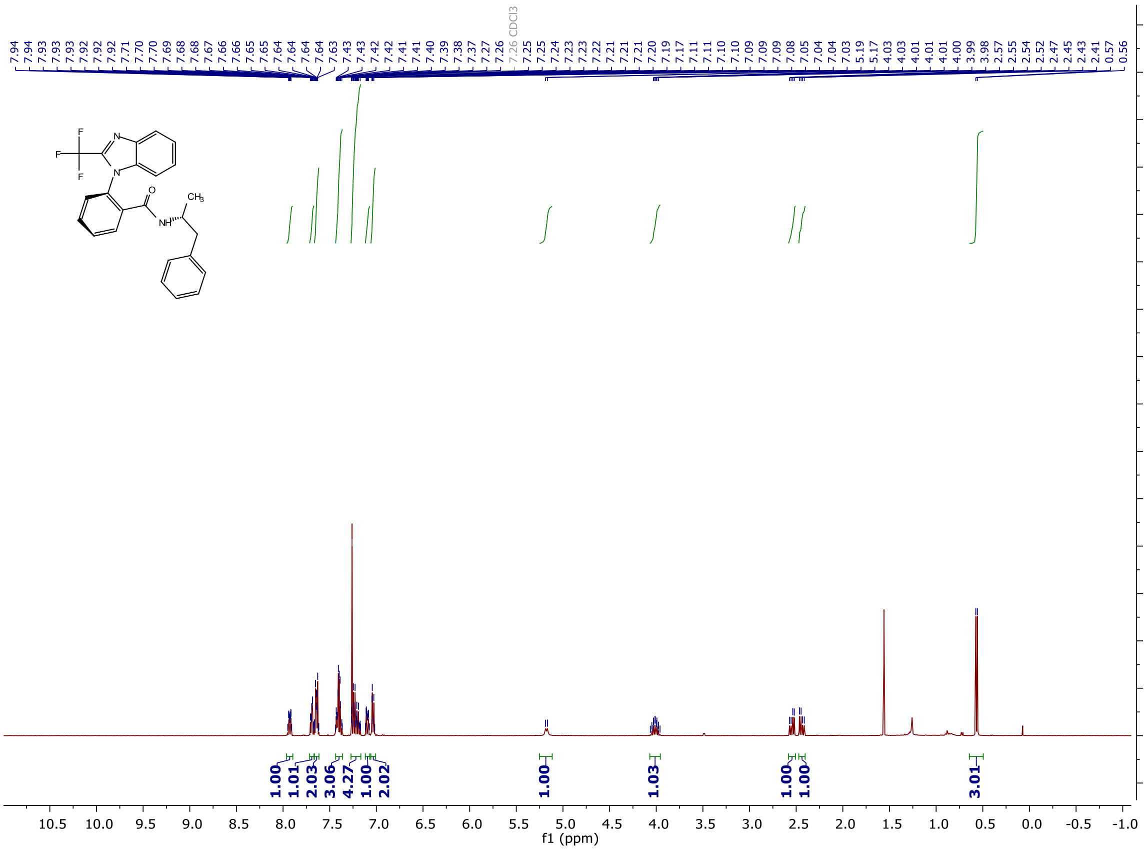

^{13}C{^{1}H} NMR (101 MHz, $CDCl_3$)

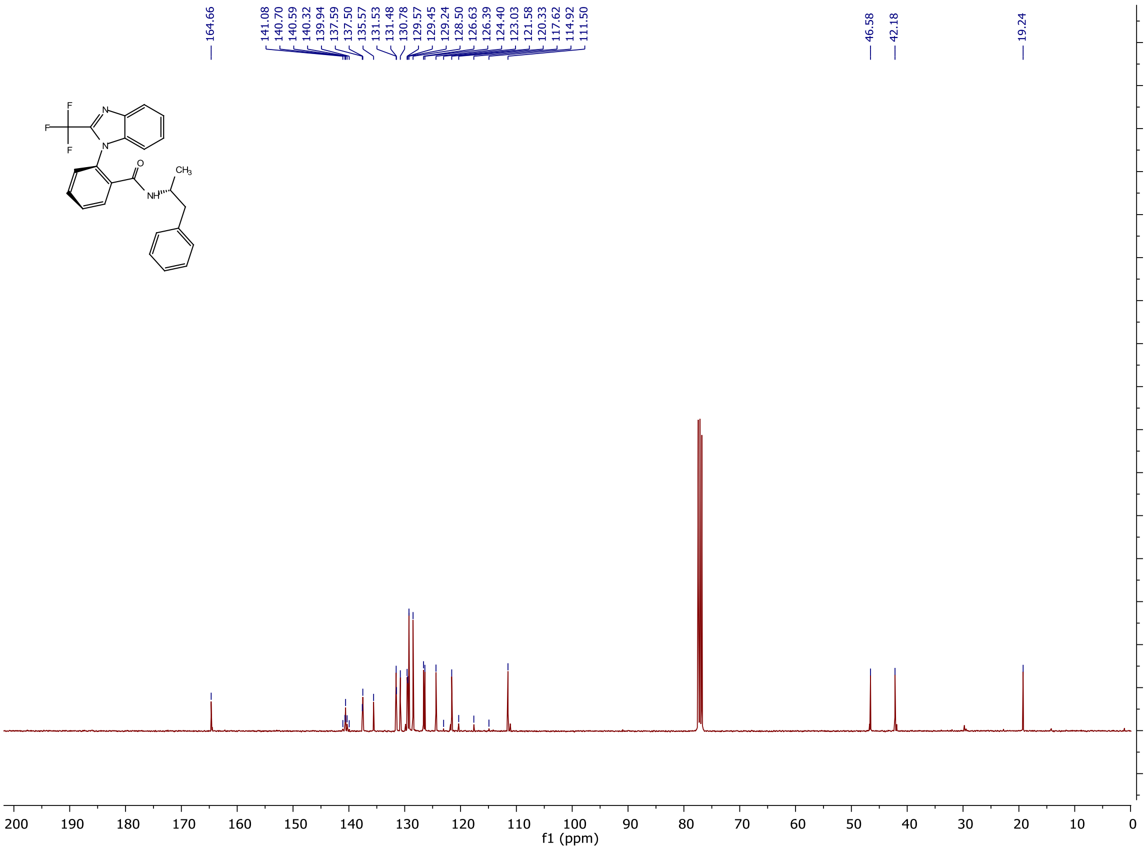

N-((*R*)-1-Phenylpropan-2-yl)-2-((*M*)-2-(trifluoromethyl)-1*H*-benzo[*d*]imidazol-1-yl)benzamide **(*M*)-29**

^{1}H NMR (400 MHz, $CDCl_3$)

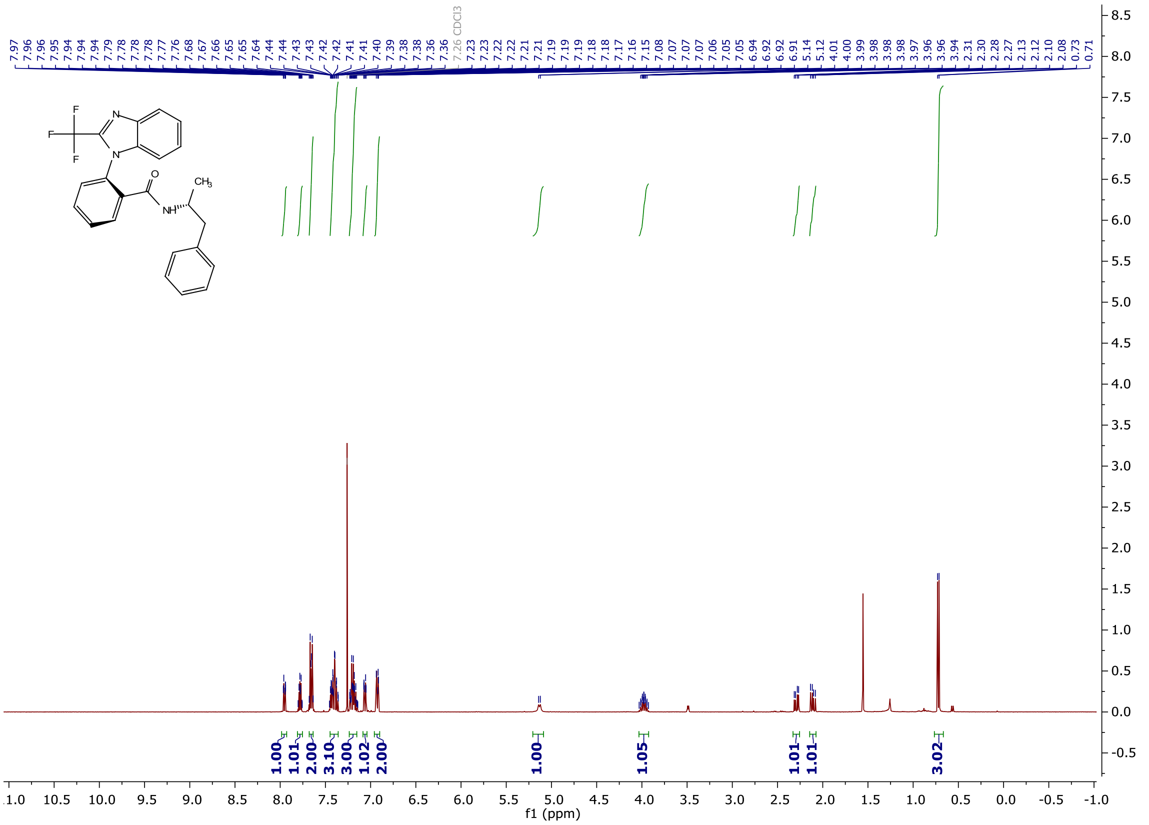

^{13}C{^{1}H} NMR (101 MHz, $CDCl_3$)

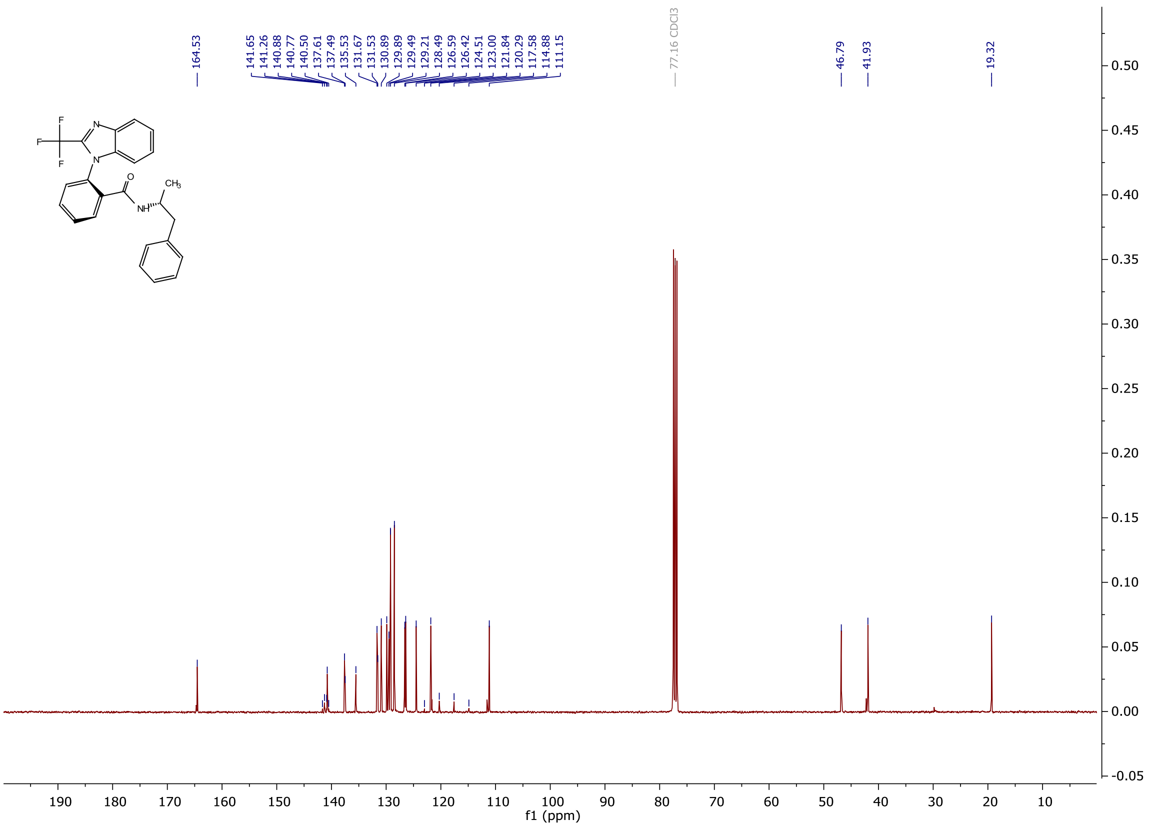

N-((*S*)-1-Hydroxy-3-phenylpropan-2-yl)-2-((*P*)-2-(trifluoromethyl)-1*H*-benzo[*d*]imidazol-1-yl)benzamide **(*P*)-30**

^{1}H NMR (400 MHz, $CDCl_3$)

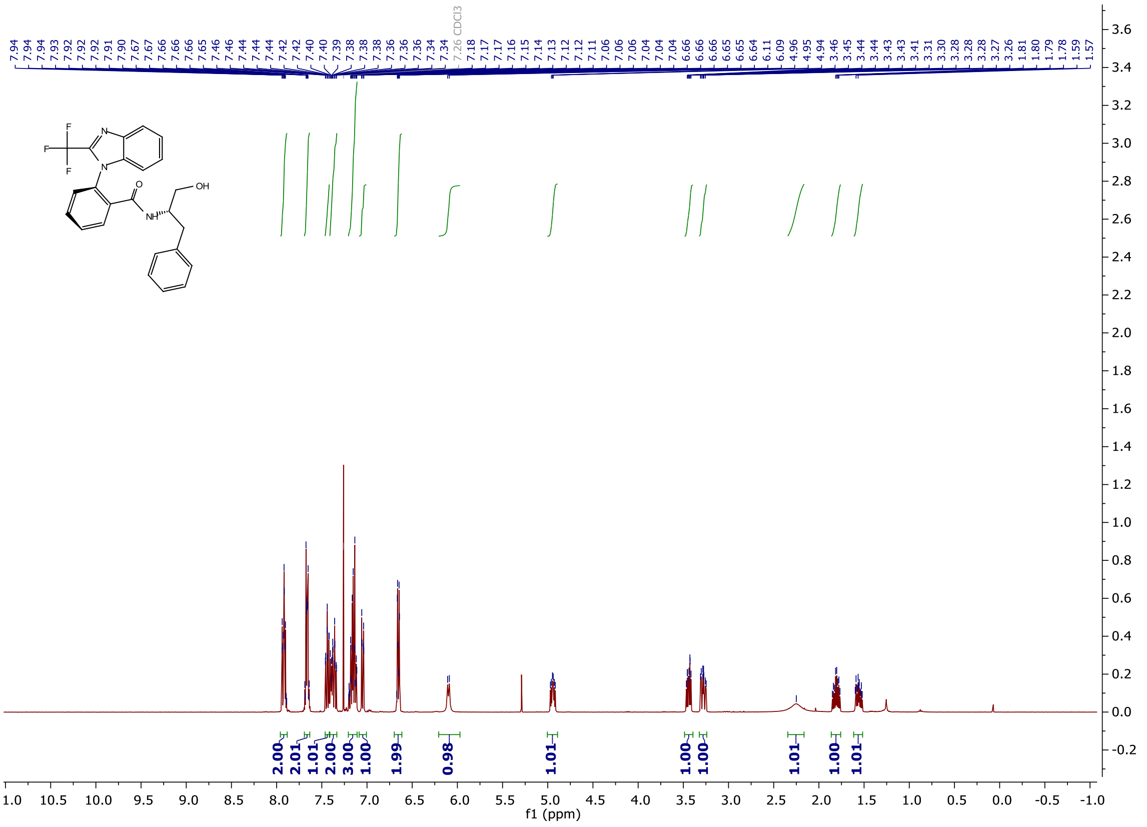

^{13}C{^{1}H} NMR (101 MHz, $CDCl_3$)

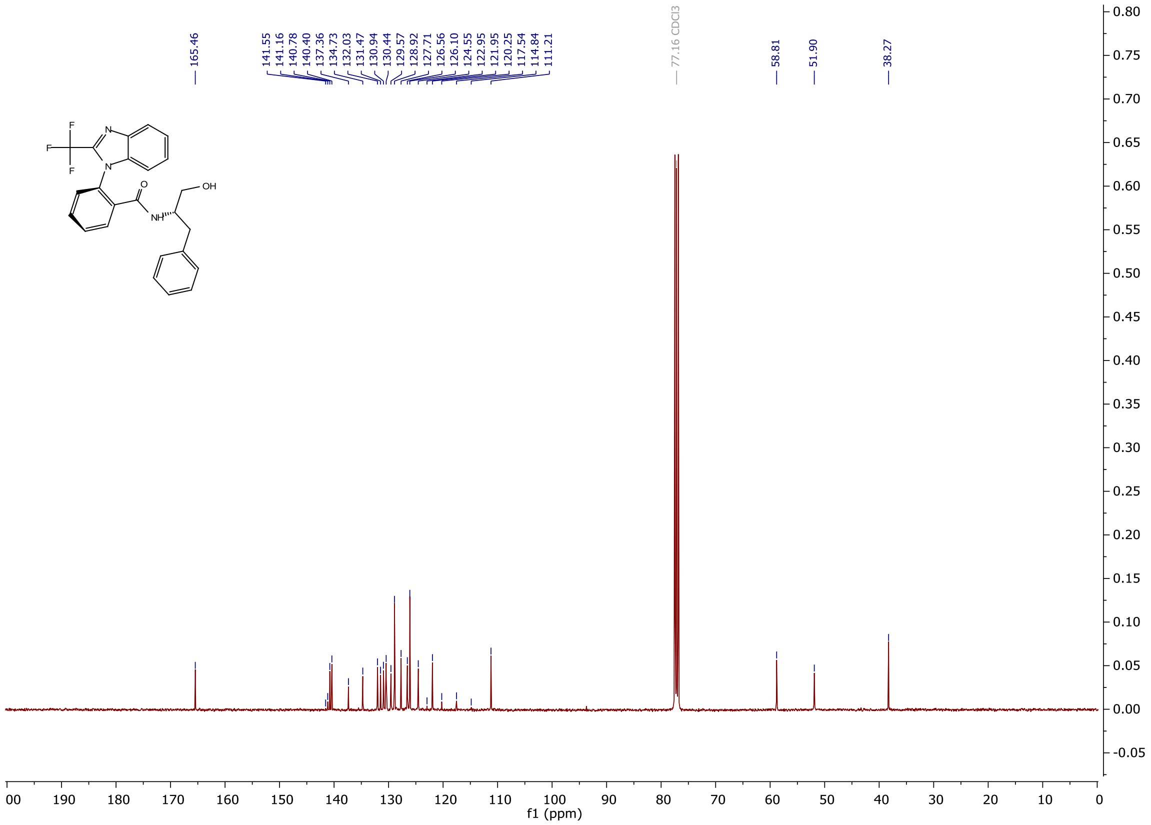

N-((*S*)-1-Hydroxy-3-phenylpropan-2-yl)-2-((*M*)-2-(trifluoromethyl)-1*H*-benzo[*d*]imidazol-1-yl)benzamide **(*M*)-30**

^{1}H NMR (400 MHz, $CDCl_3$)

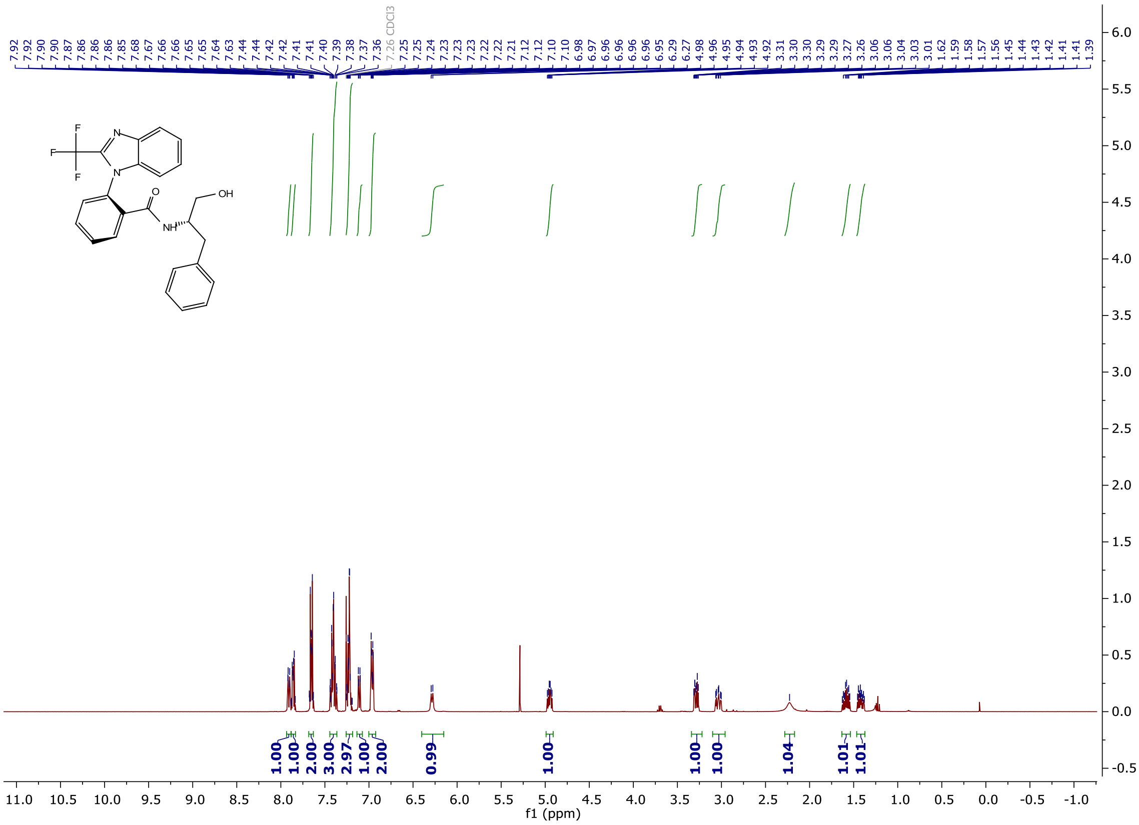

^{13}C{^{1}H} NMR (101 MHz, $CDCl_3$)

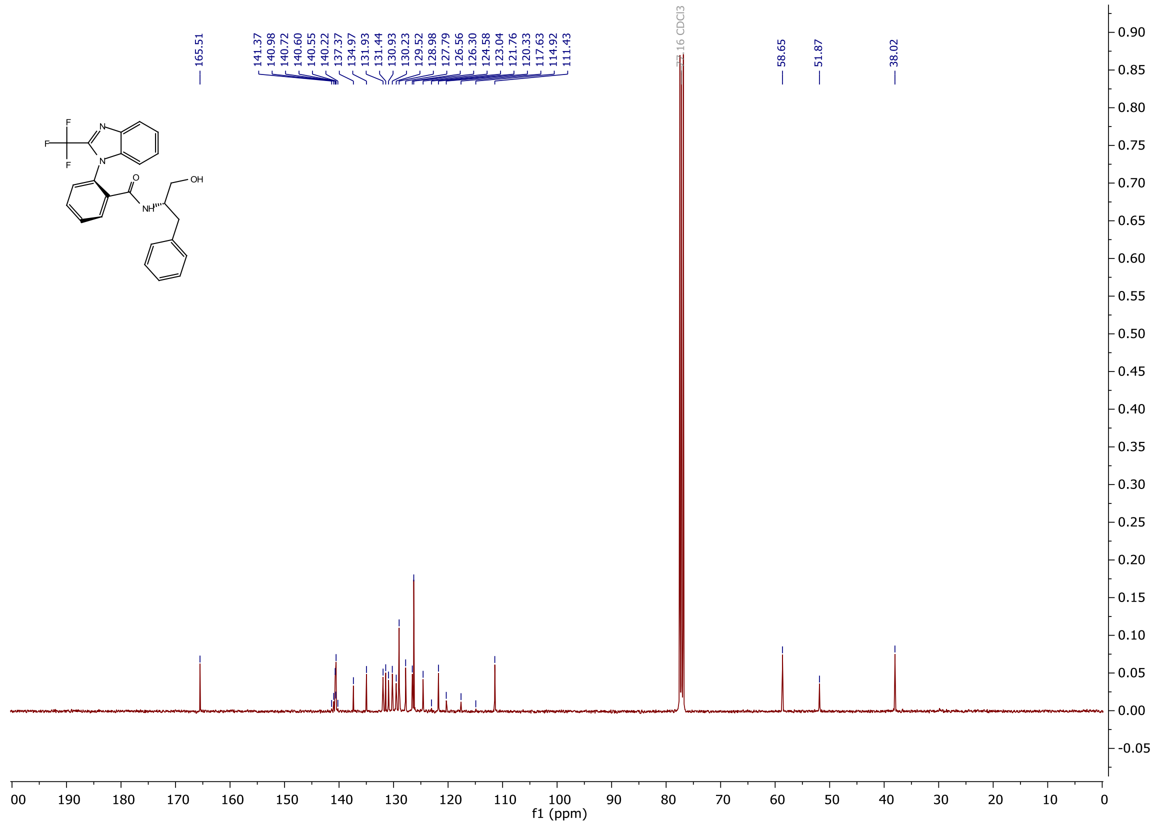

N-((*S*)-3-Hydroxy-1-phenylpropyl)-2-((*P*)-2-(trifluoromethyl)-1*H*-benzo[*d*]imidazol-1-yl)benzamide **(*P*)-32**

^{1}H NMR (400 MHz, $CDCl_3$)

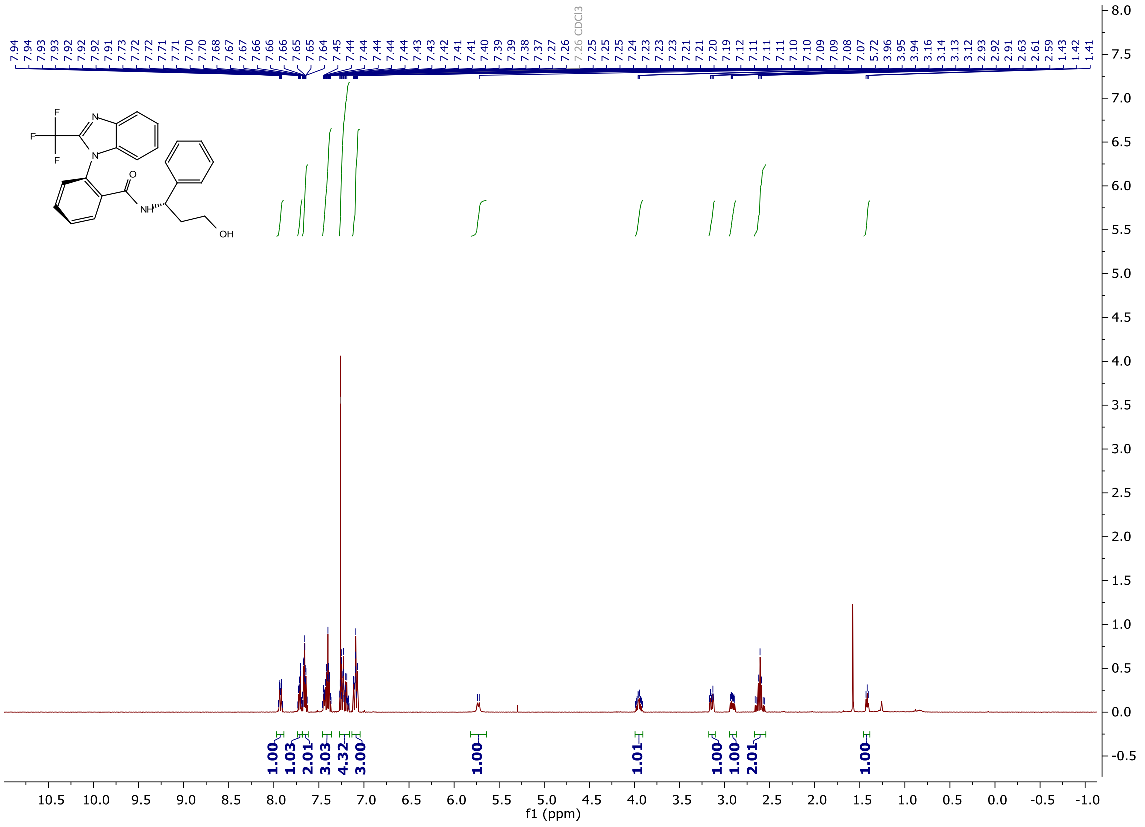

^{13}C{^{1}H} NMR (101 MHz, $CDCl_3$)

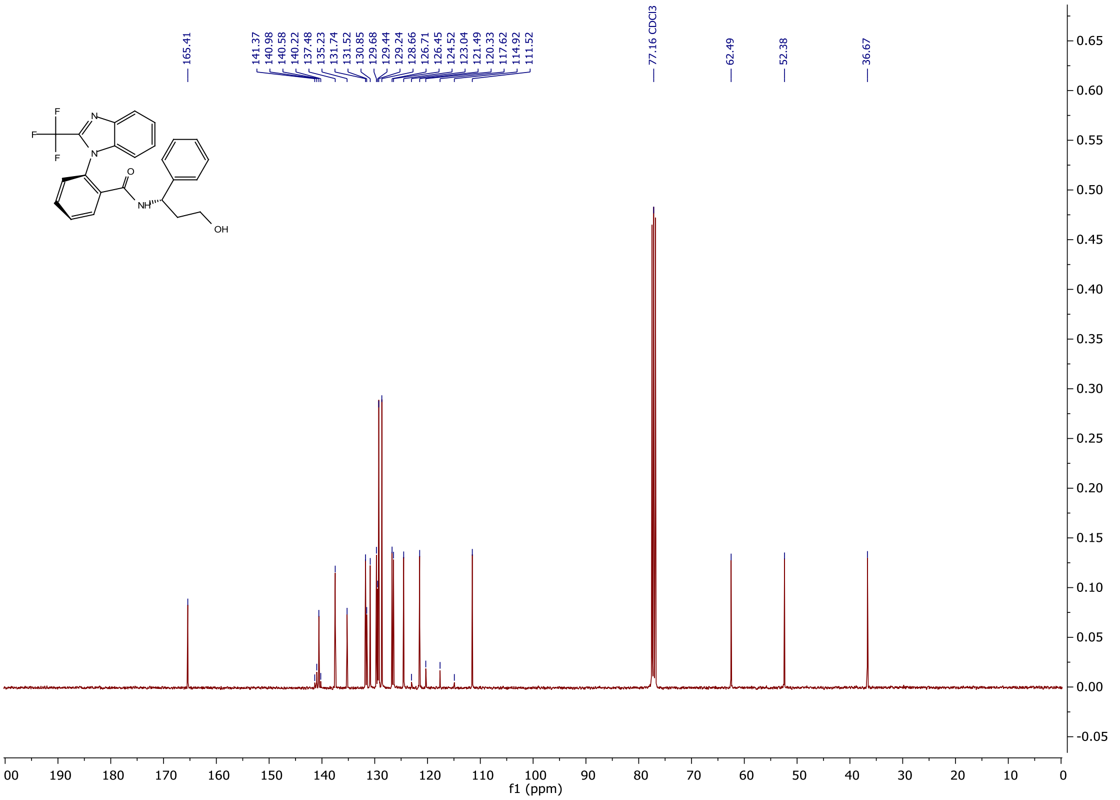

N-((*S*)-3-hydroxy-1-phenylpropyl)-2-((*M*)-2-(trifluoromethyl)-1*H*-benzo[*d*]imidazol-1-yl)benzamide

(*M*)-32

^{1}H NMR (400 MHz, $CDCl_3$)

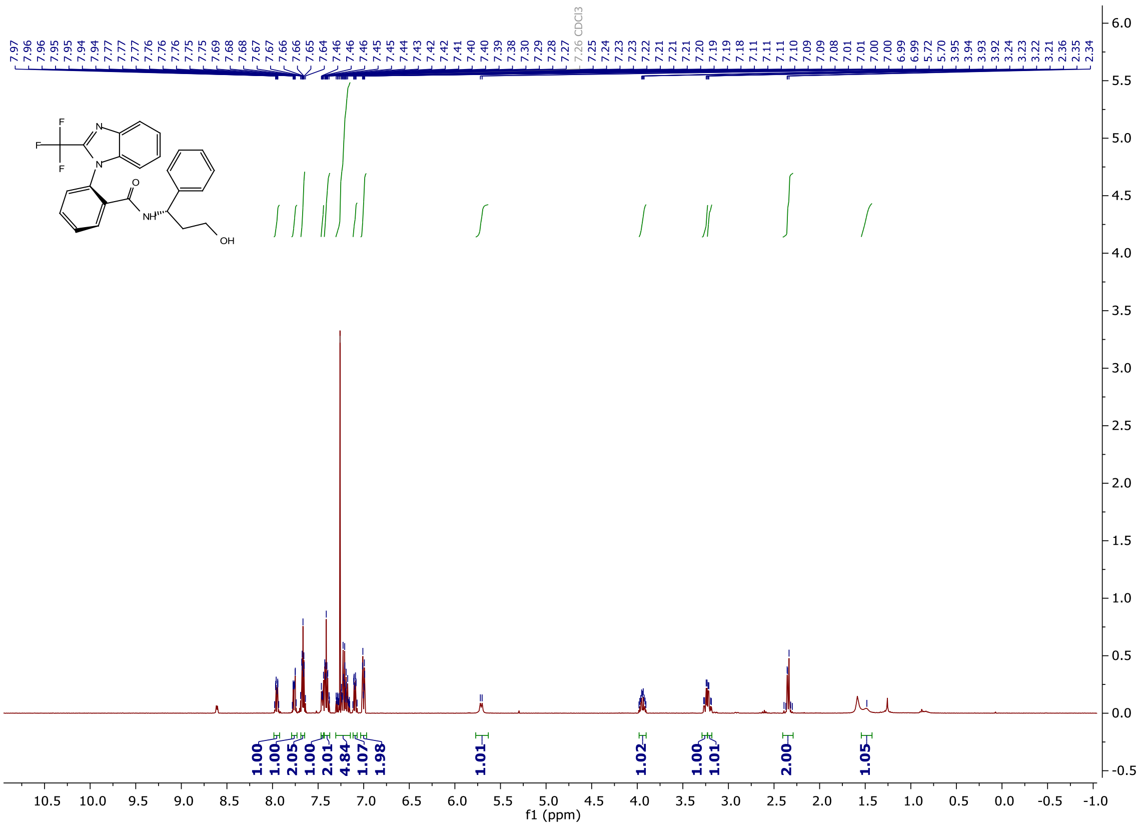

^{13}C{^{1}H} NMR (101 MHz, $CDCl_3$)

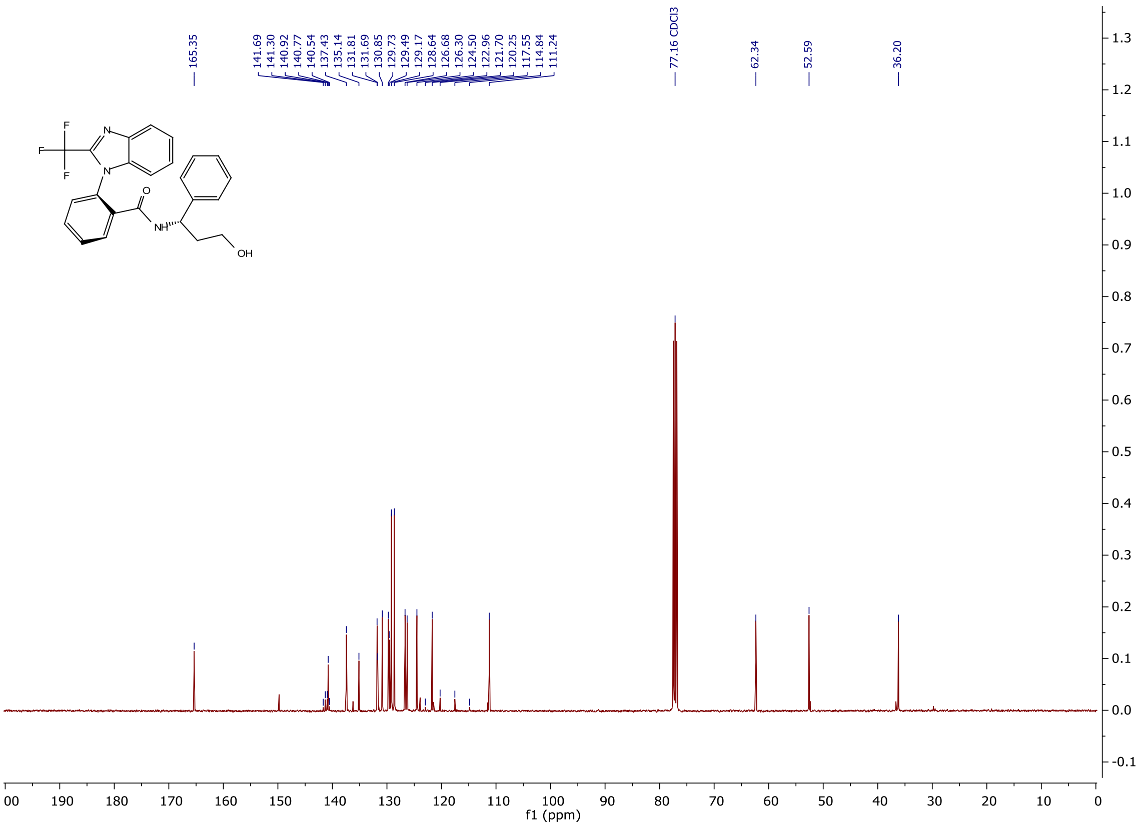

N-((*R*)-1-Hydroxybutan-2-yl)-2-((*P*)-2-(trifluoromethyl)-1*H*-benzo[*d*]imidazol-1-yl)benzamide ***(P)*-34**

^{1}H NMR (400 MHz, $CDCl_3$)

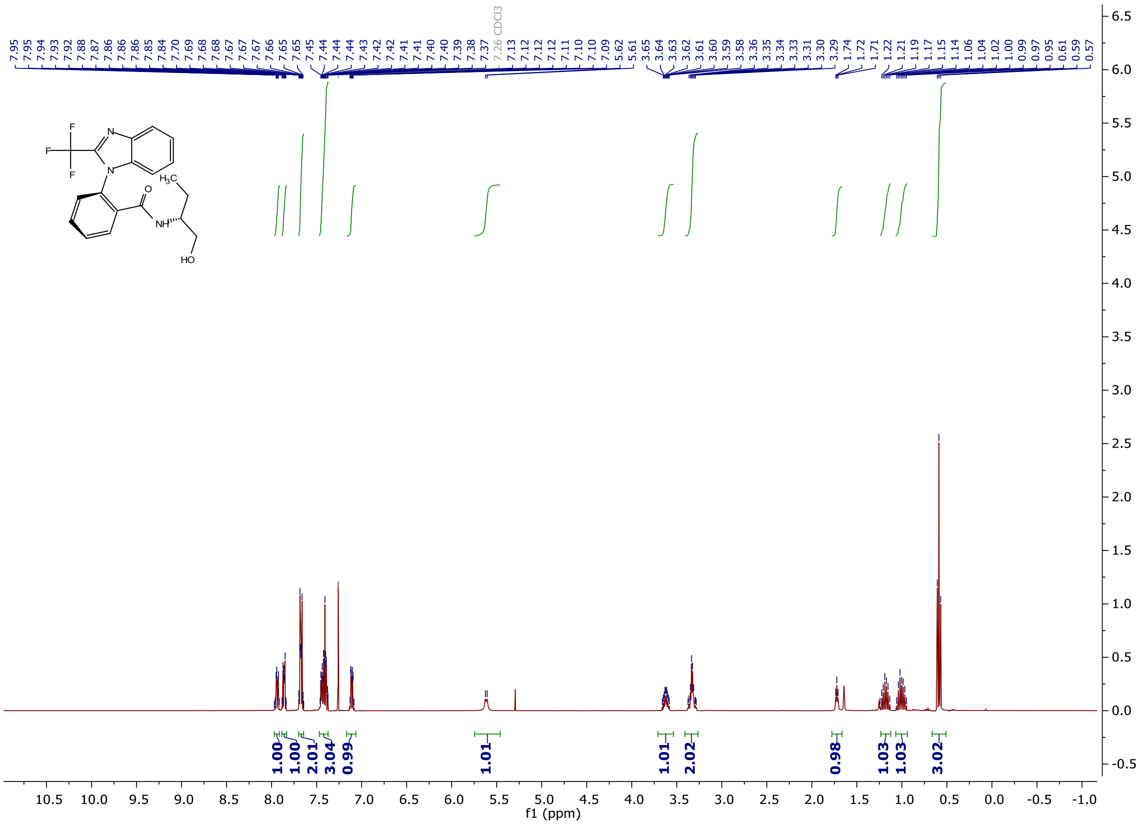

^{13}C{^{1}H} NMR (101 MHz, $CDCl_3$)

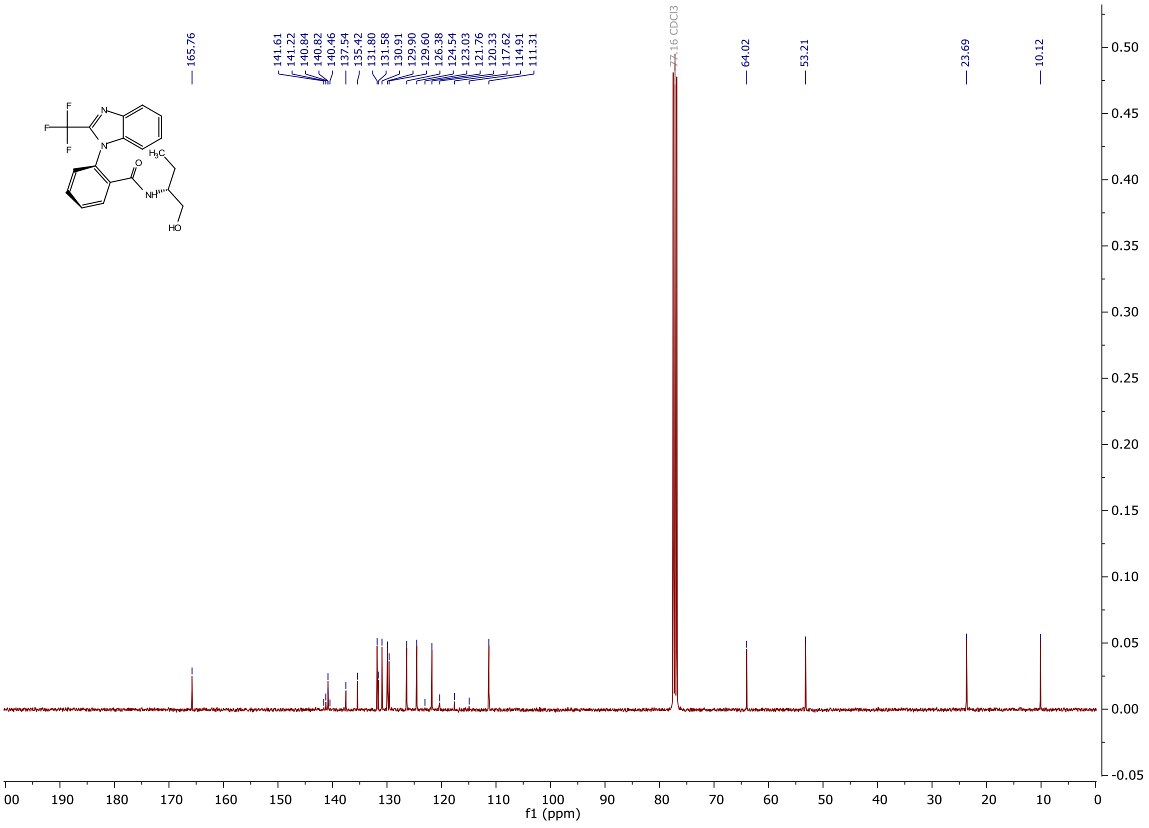

N-((*R*)-1-Hydroxybutan-2-yl)-2-((*M*)-2-(trifluoromethyl)-1*H*-benzo[*d*]imidazol-1-yl)benzamide **(*M*)-34**

^{1}H NMR (400 MHz, $CDCl_3$)

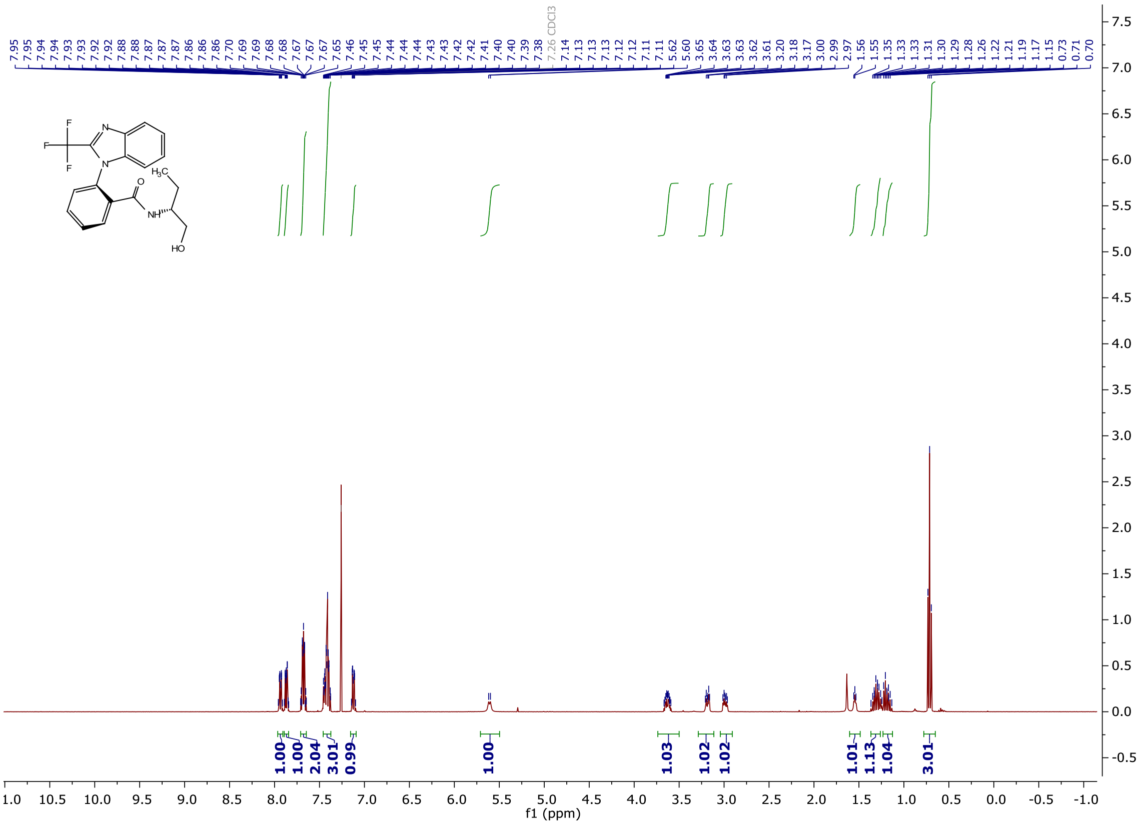

^{13}C{^{1}H} NMR (101 MHz, $CDCl_3$)

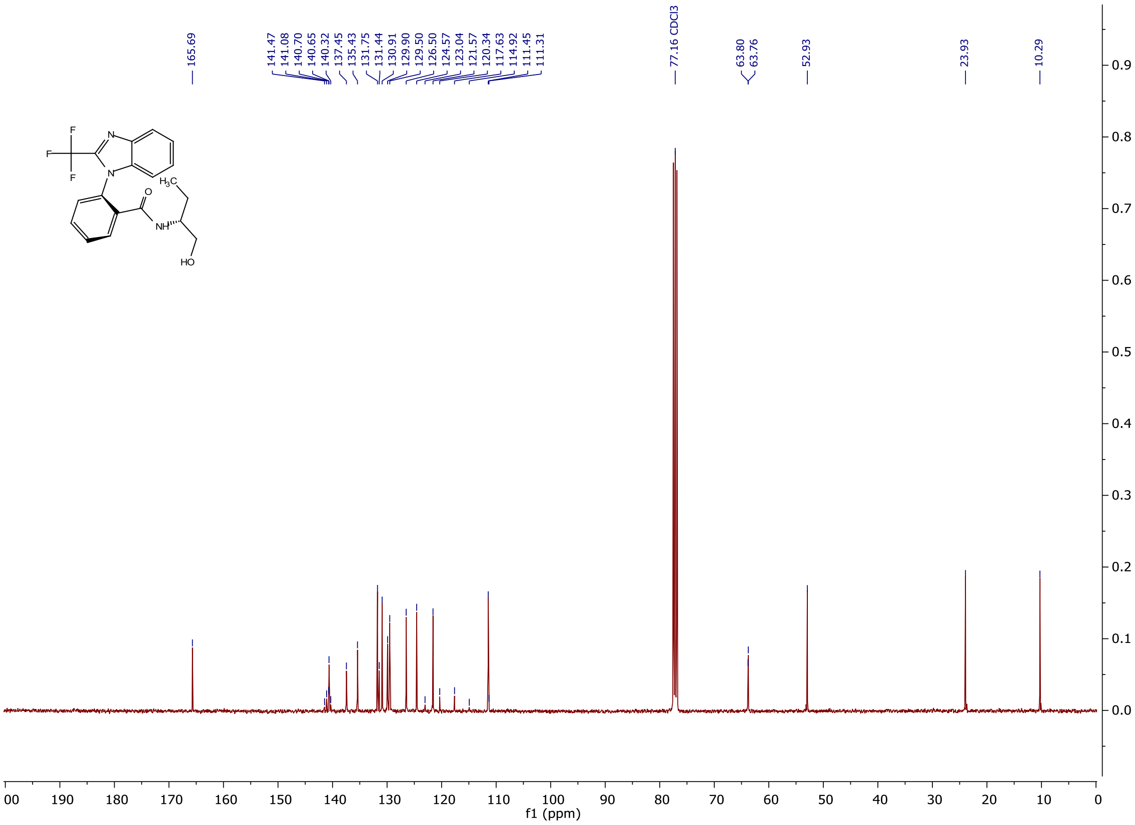

N-((*S*)-1-Hydroxypropan-2-yl)-2-((*P*)-2-(trifluoromethyl)-1*H*-benzo[*d*]imidazol-1-yl)benzamide **(*P*)-35**

^{1}H NMR (400 MHz, $CDCl_3$)

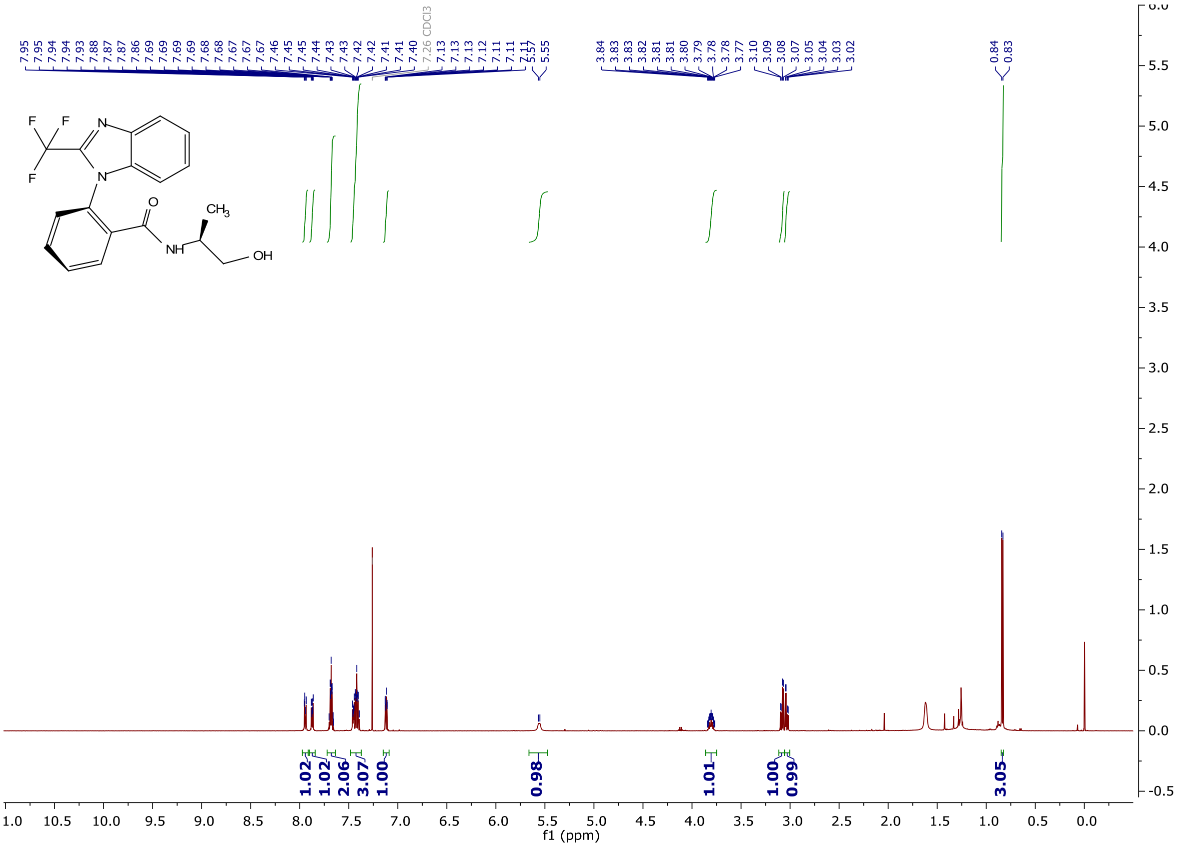

^{13}C{^{1}H} NMR (101 MHz, $CDCl_3$)

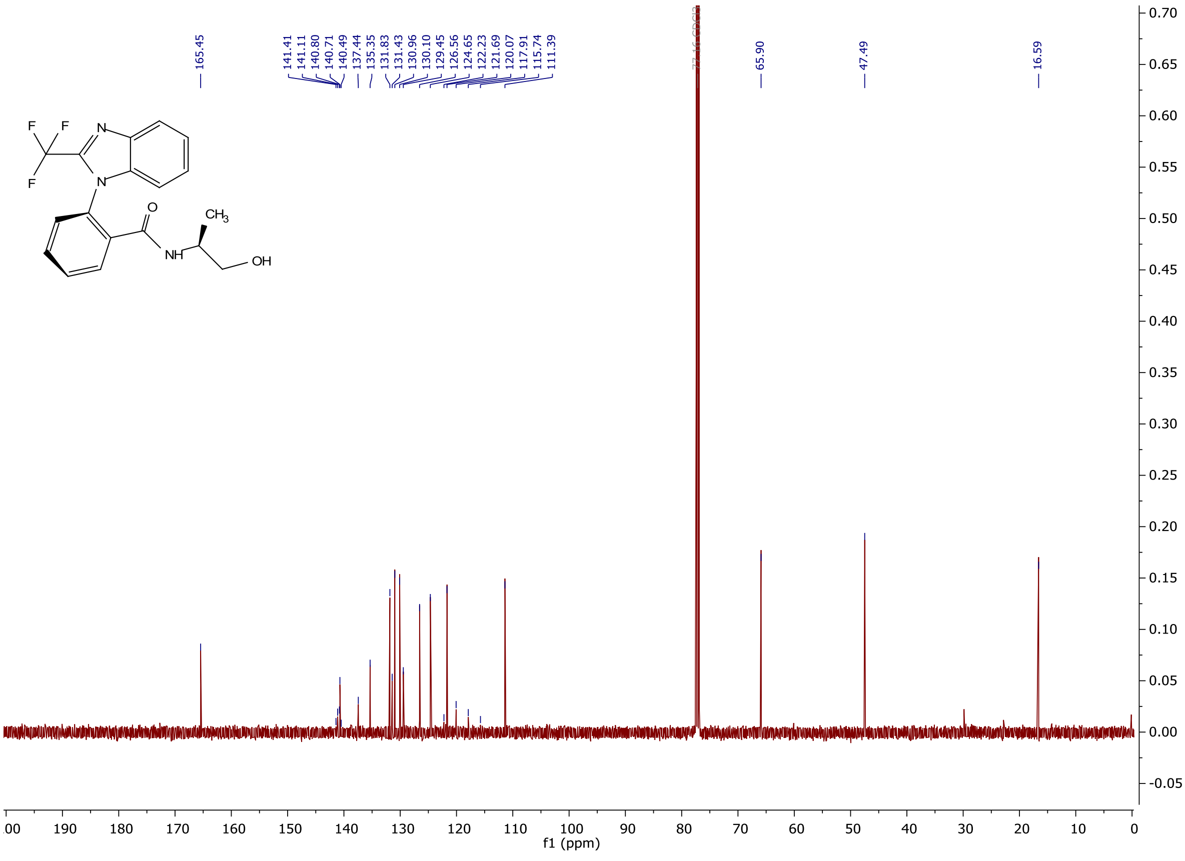

N-((*S*)-1-Hydroxypropan-2-yl)-2-((*P*)-2-(trifluoromethyl)-1*H*-benzo[*d*]imidazol-1-yl)benzamide **(*M*)-35**

^{1}H NMR (400 MHz, $CDCl_3$)

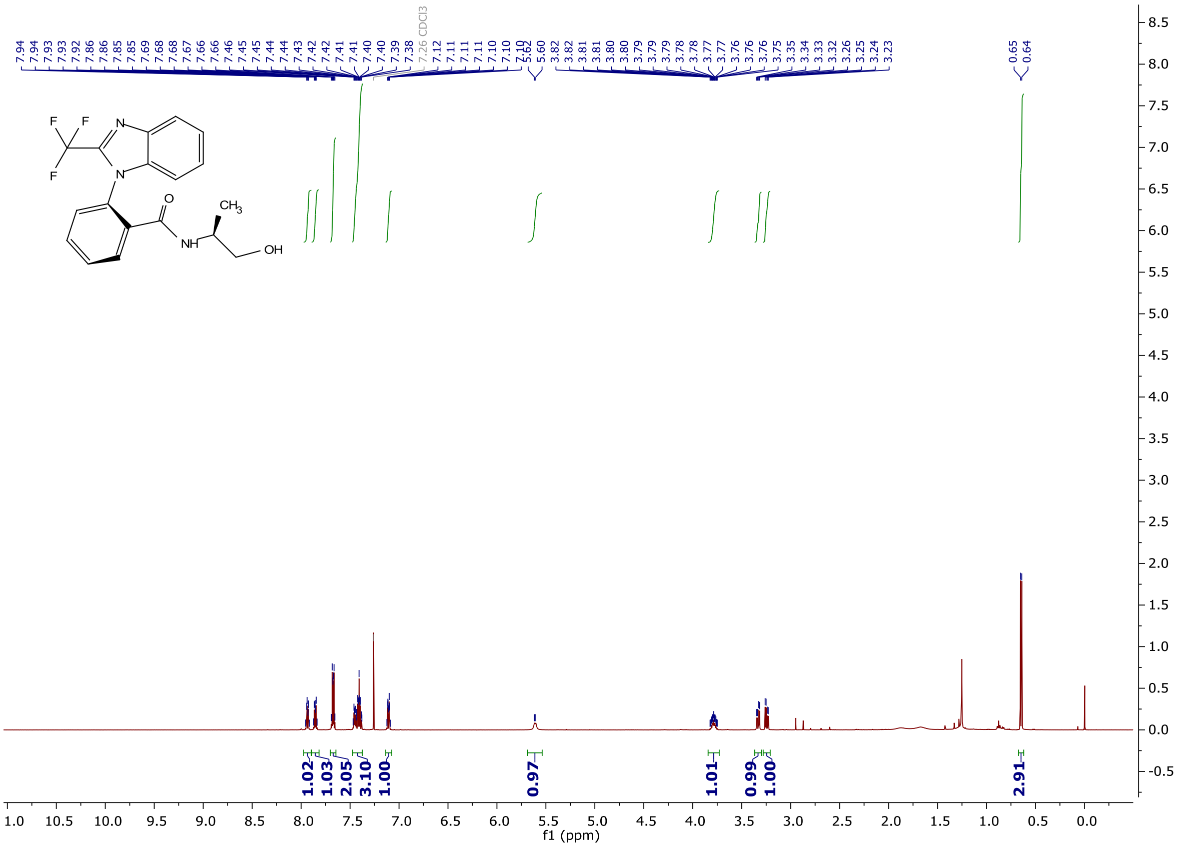

^{13}C{^{1}H} NMR (101 MHz, $CDCl_3$)

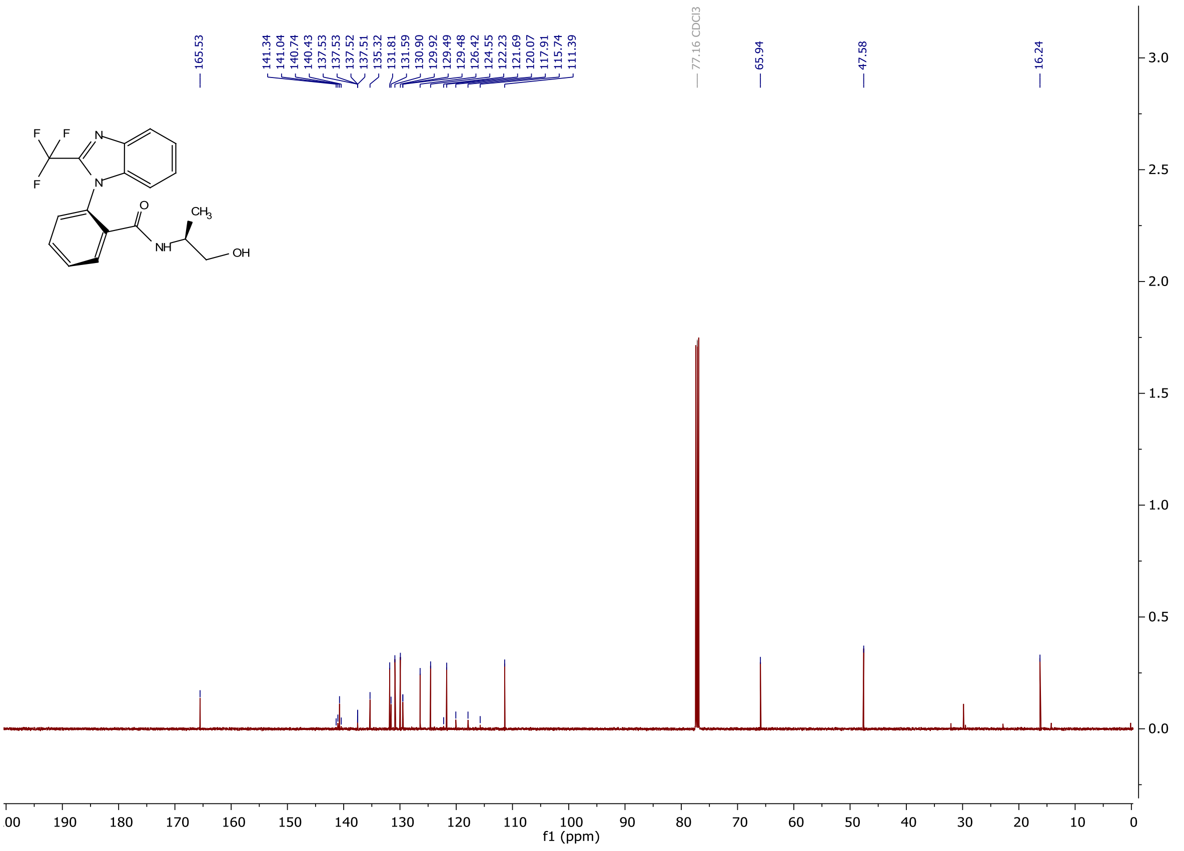

N-((*R*)-1-Methoxypropan-2-yl)-2-((*P*)-2-(trifluoromethyl)-1*H*-benzo[*d*]imidazol-1-yl)benzamide **(*P*)-36**

^{1}H NMR (400 MHz, $CDCl_3$)

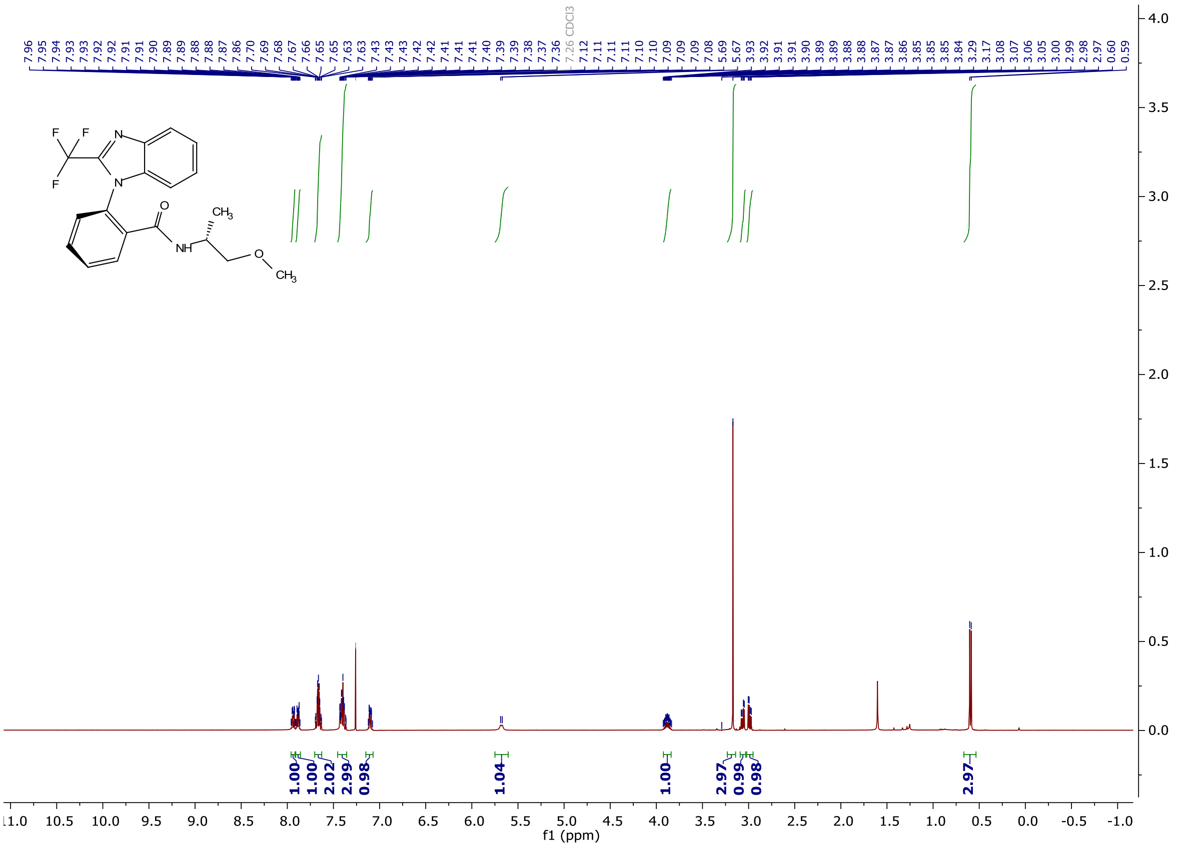

^{13}C{^{1}H} NMR (101 MHz, $CDCl_3$)

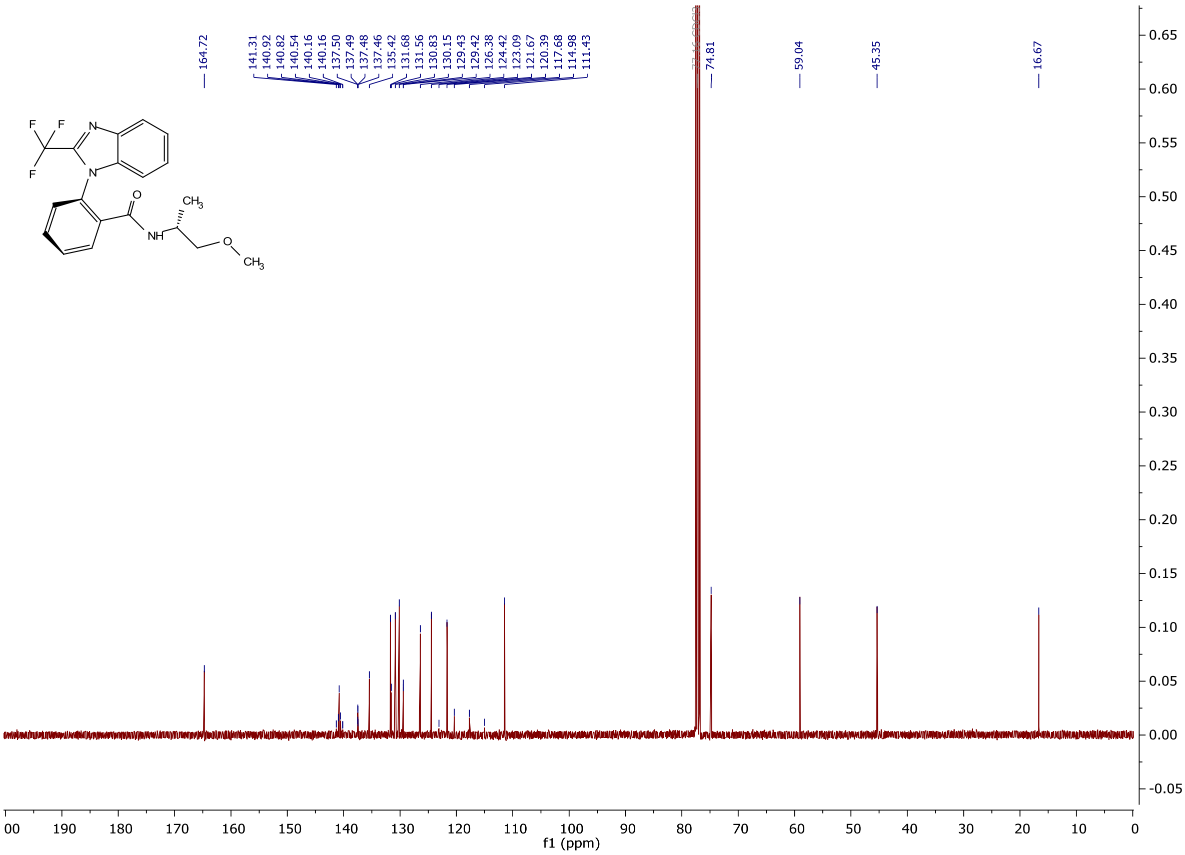

N-((*R*)-1-Methoxypropan-2-yl)-2-((*M*)-2-(trifluoromethyl)-1*H*-benzo[*d*]imidazol-1-yl)benzamide **(*M*)-36**

^{1}H NMR (400 MHz, $CDCl_3$)

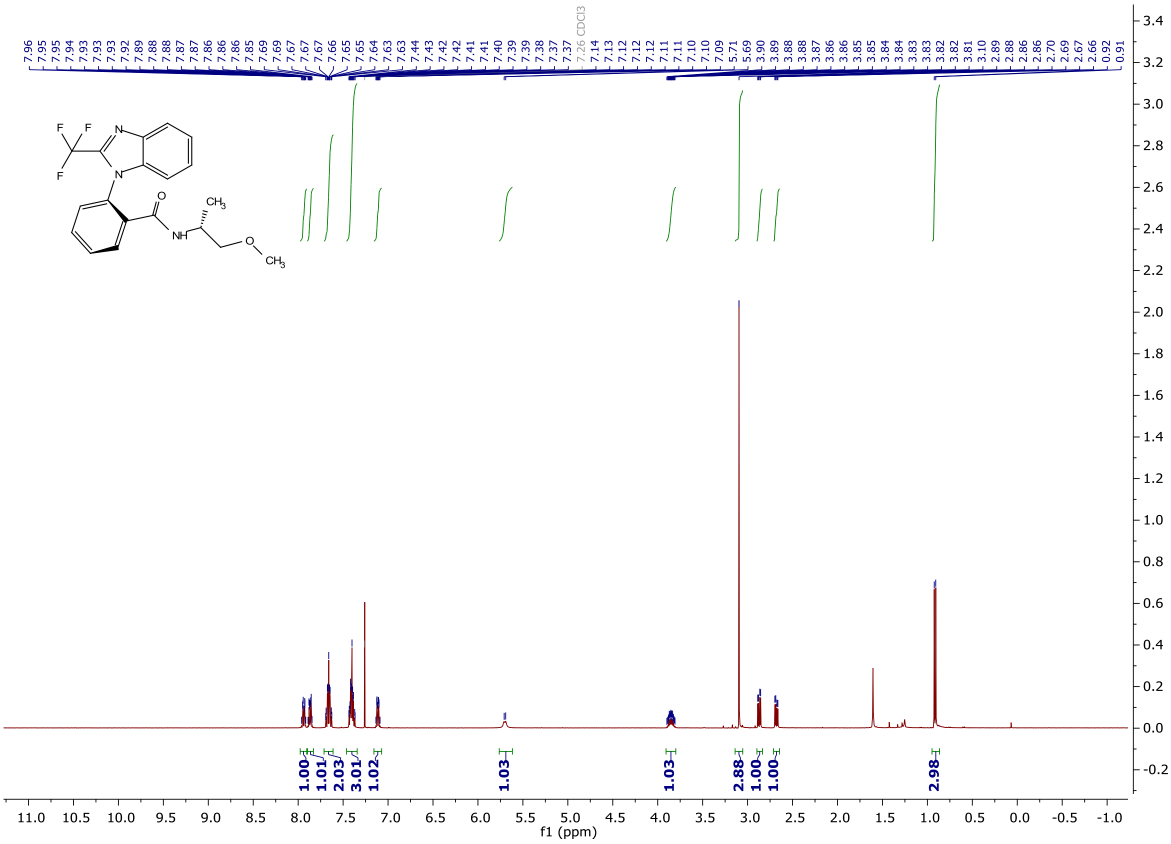

^{13}C{^{1}H} NMR (101 MHz, $CDCl_3$)

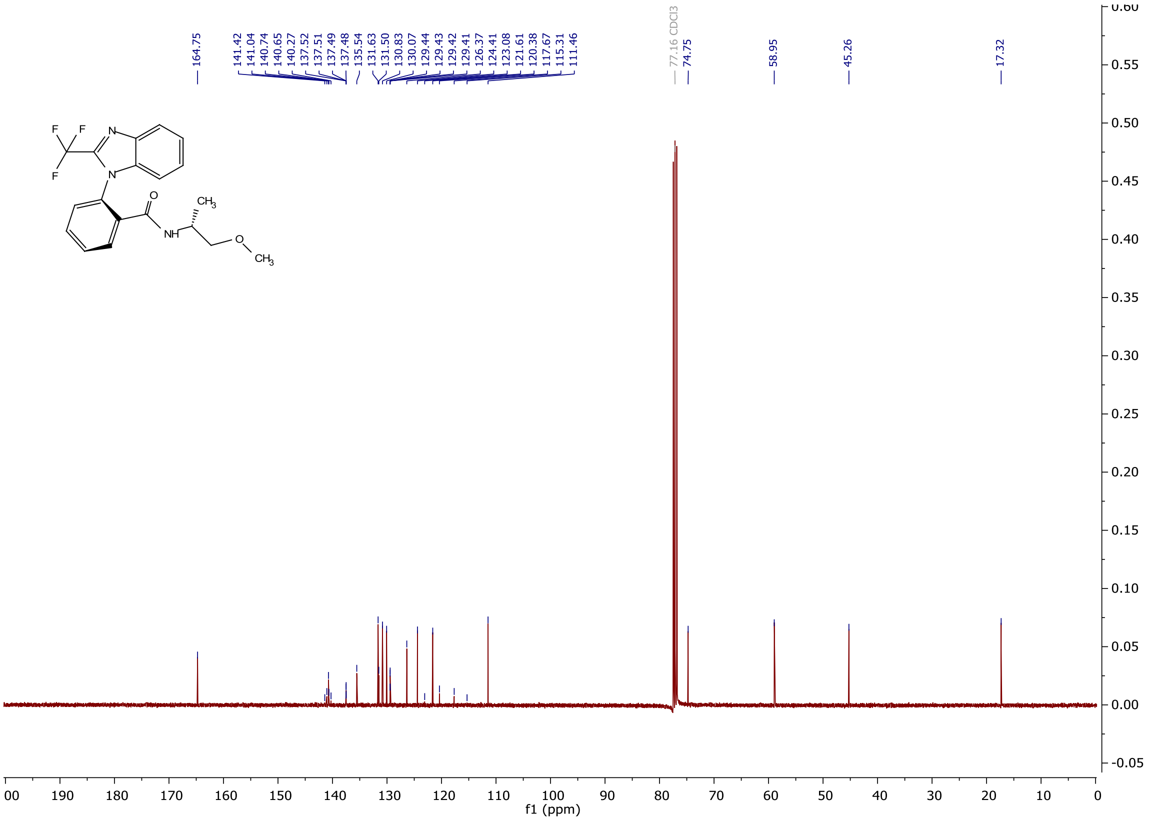

N-((1*R*,2*R*)-2-Aminocyclohexyl)-2-((*P*)-2-(trifluoromethyl)-1*H*-benzo[*d*]imidazol-1-yl)benzamide **(*P*)-37**

^{1}H NMR (400 MHz, $CDCl_3$)

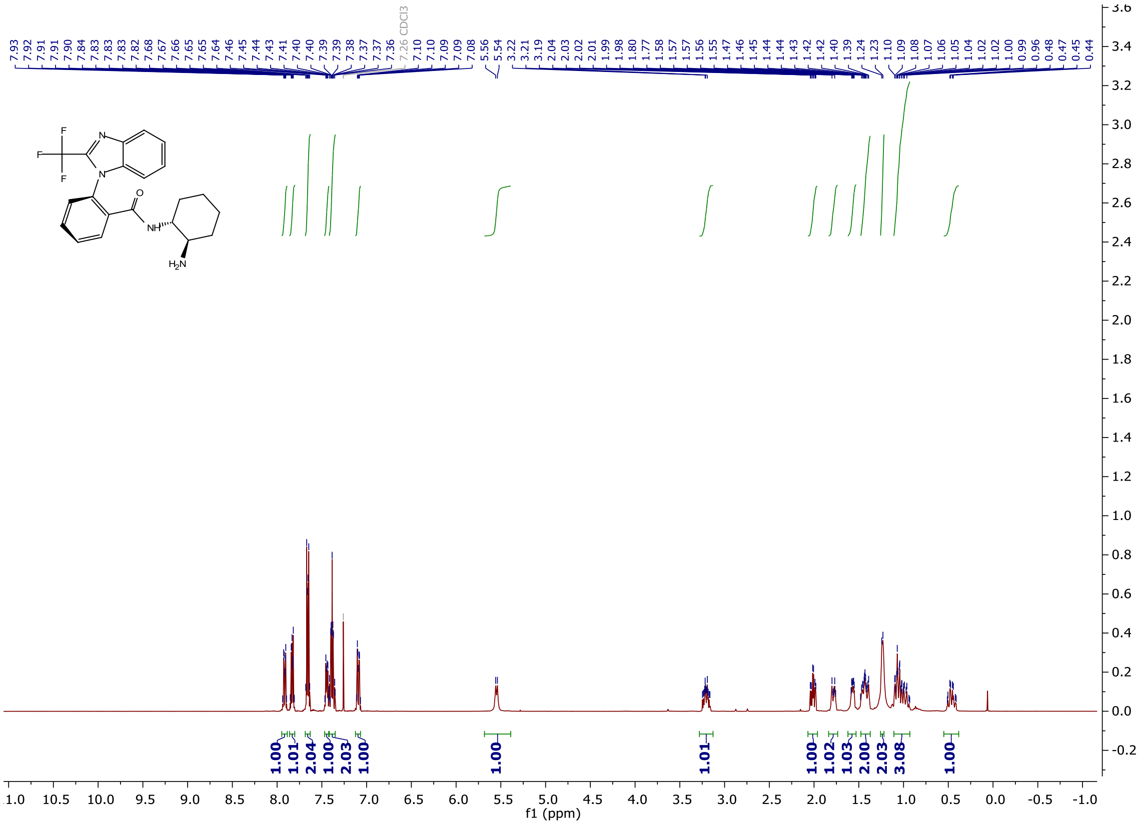

^{13} C{^{1}H} NMR (101 MHz, $CDCl_3$)

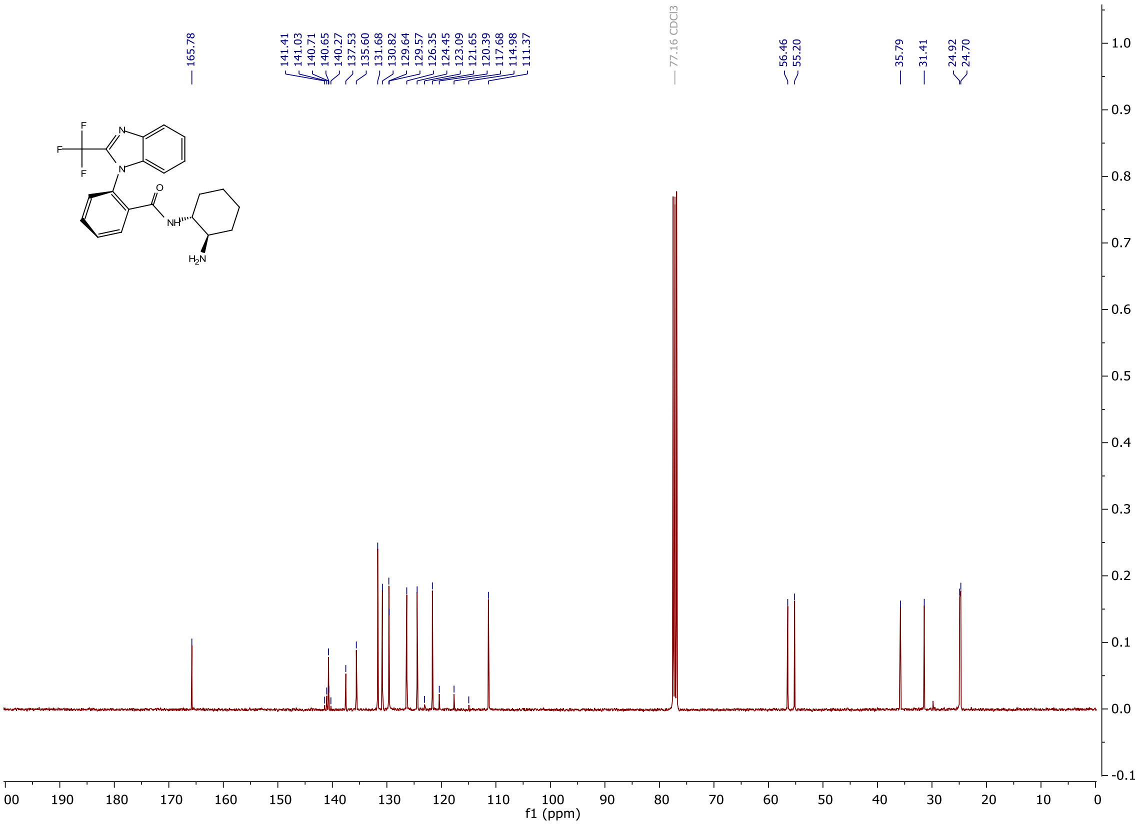

N-((1*R*,2*R*)-2-Aminocyclohexyl)-2-((*M*)-2-(trifluoromethyl)-1*H*-benzo[*d*]imidazol-1-yl)benzamide **(*M*)-37**

^{1}H NMR (400 MHz, $CDCl_3$)

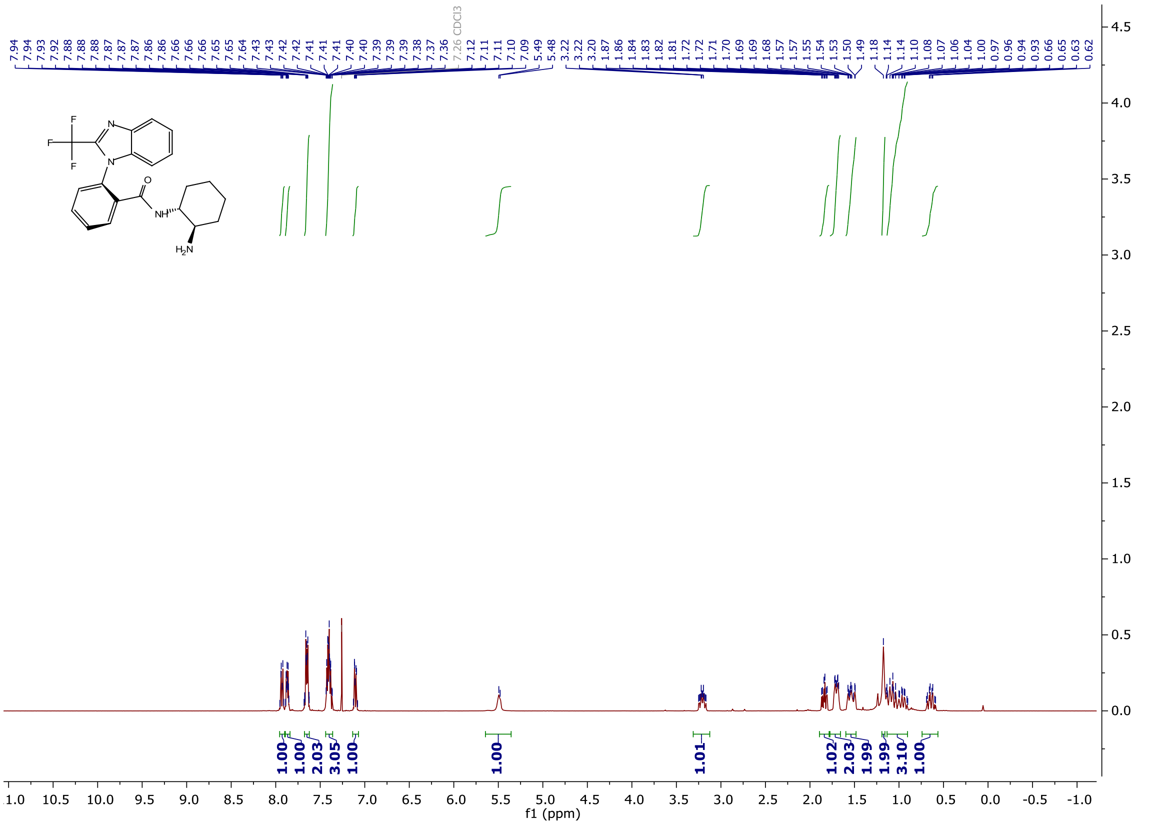

^{13}C{^{1}H} NMR (101 MHz, $CDCl_3$)

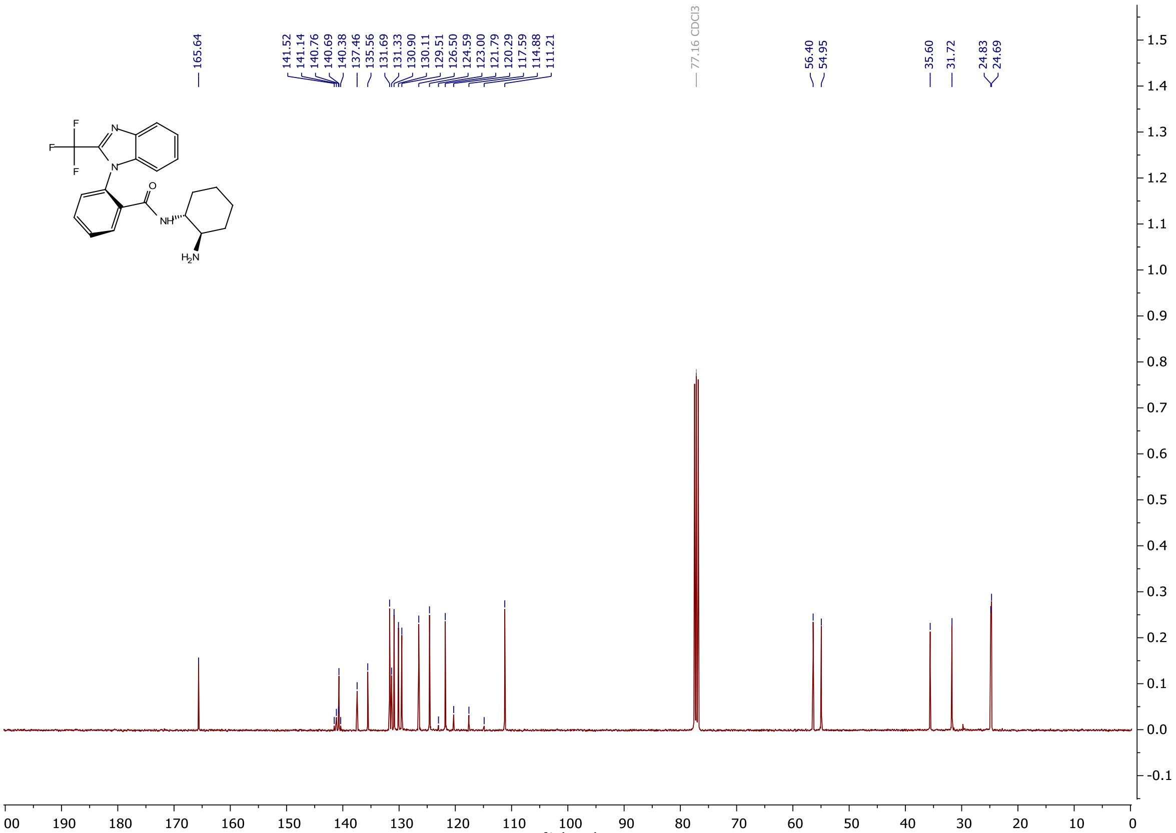

tert-Butyl ((1*R*,2*R*)-2-(2-((*P*)-2-(trifluoromethyl)-1*H*-benzo[*d*]imidazol-1-yl)benzamido)cyclohexyl)carbamate **(*P*)-38**

^{1}H NMR (400 MHz, $CDCl_3$)

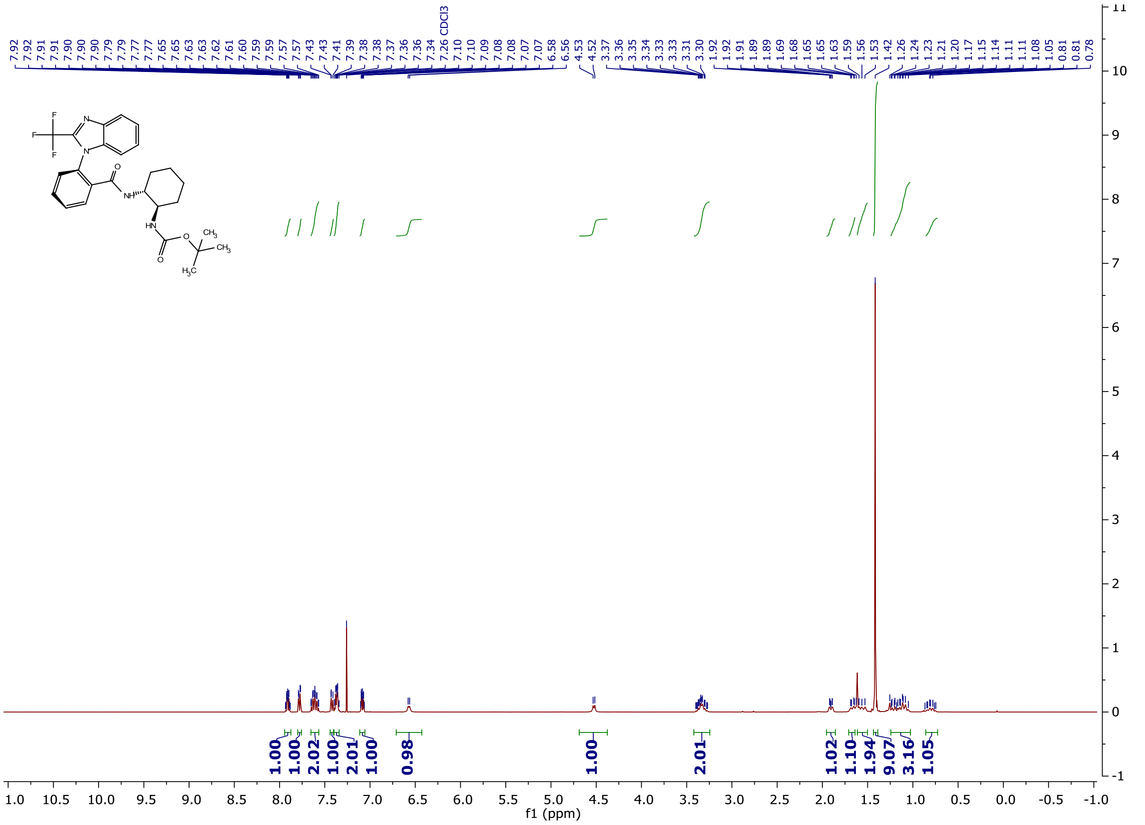

^{13}C{^{1}H} NMR (101 MHz, $CDCl_3$)

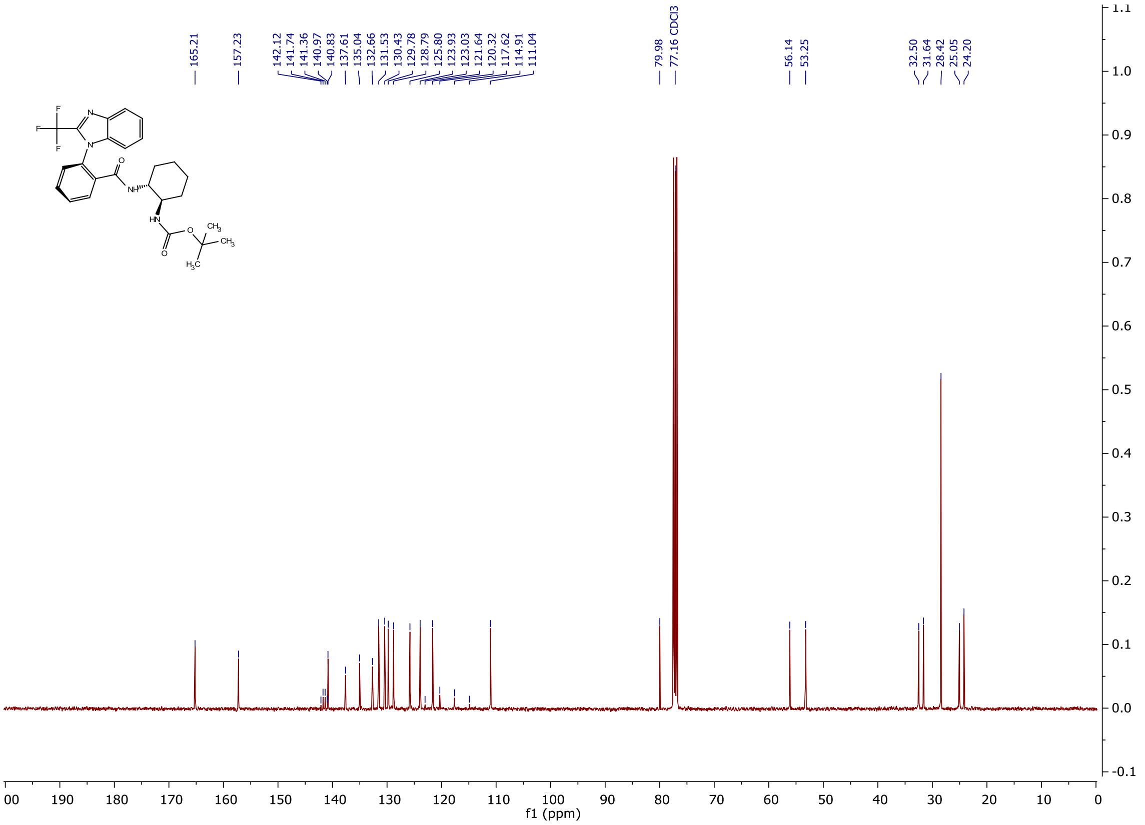

tert-Butyl ((1*R*,2*R*)-2-(2-((*M*)-2-(trifluoromethyl)-1*H*-benzo[*d*]imidazol-1-yl)benzamido)cyclohexyl)carbamate **(*M*)-38**

^{1}H NMR (400 MHz, $CDCl_3$)

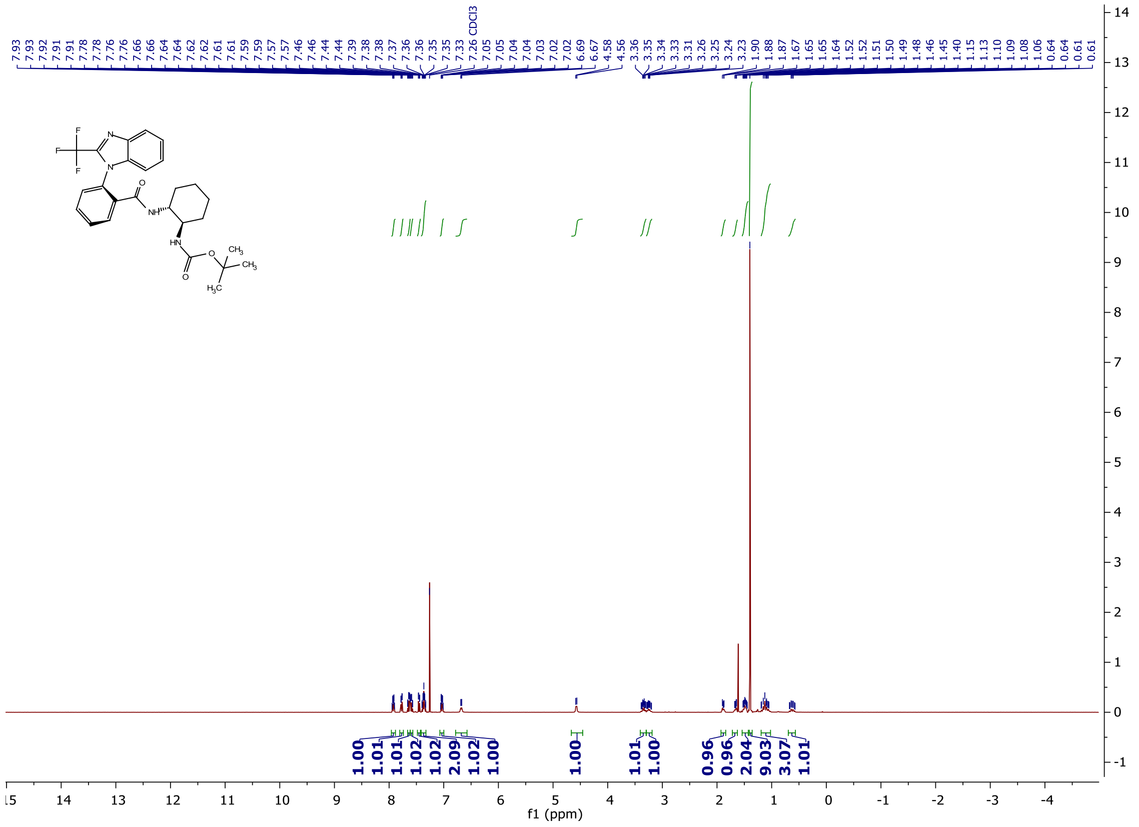

^{13}C{^{1}H} NMR (101 MHz, $CDCl_3$)

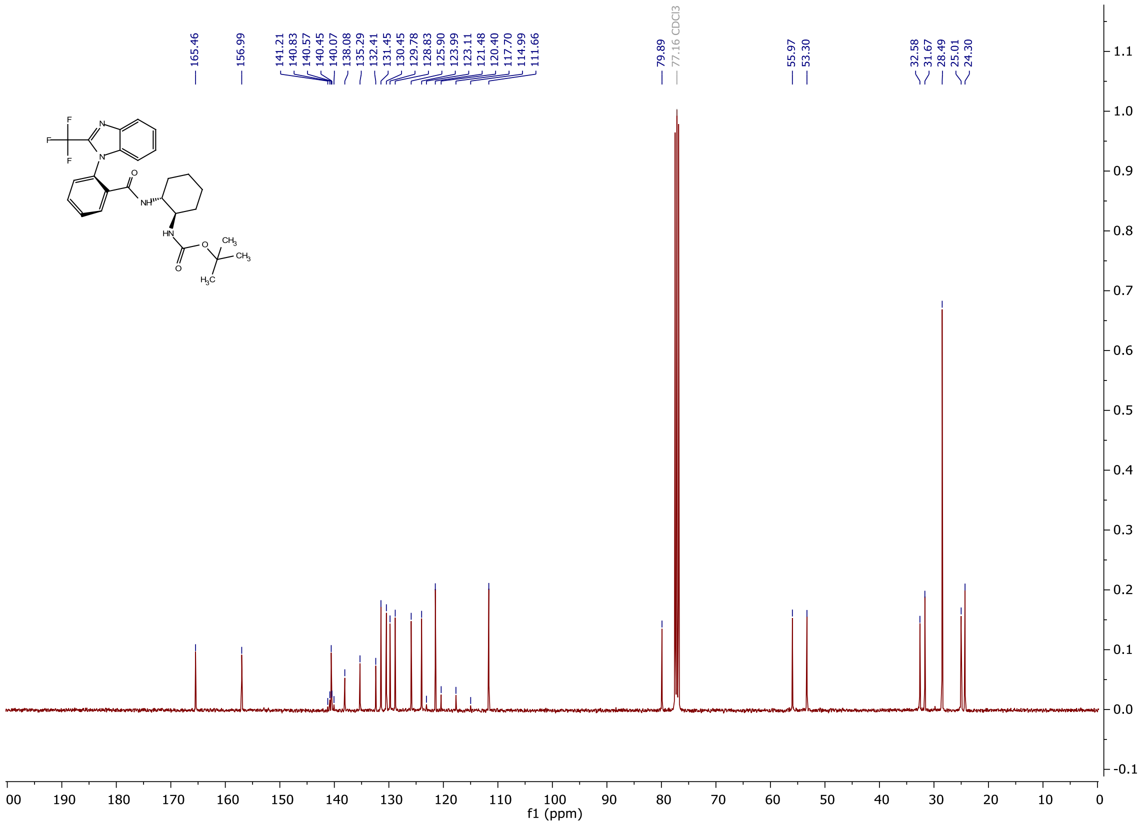

tert-Butyl (*R*)-3-(2-((*P*)-2-(trifluoromethyl)-1*H*-benzo[*d*]imidazol-1-yl)benzamido)piperidine-1-carboxylate **(*P*)-39**

^{1}H NMR (400 MHz, $CDCl_3$)

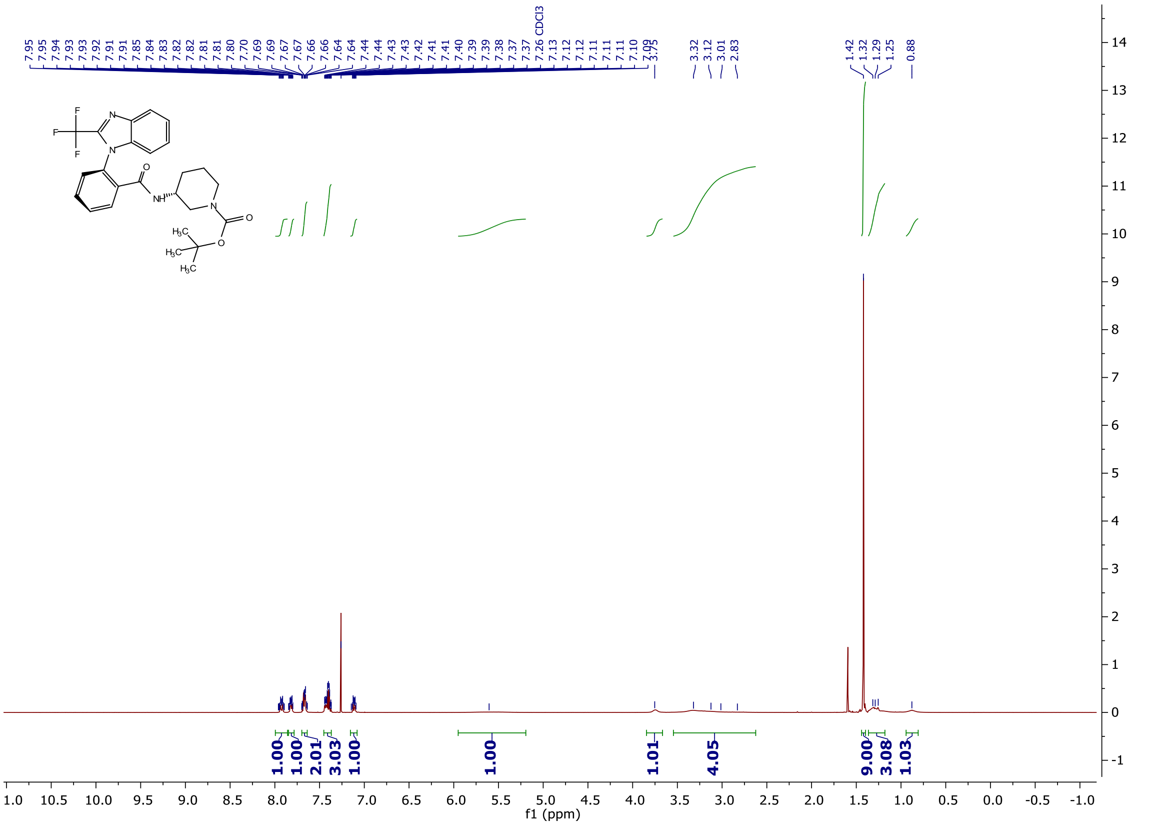

^{13}C{^{1}H} NMR (101 MHz, $CDCl_3$)

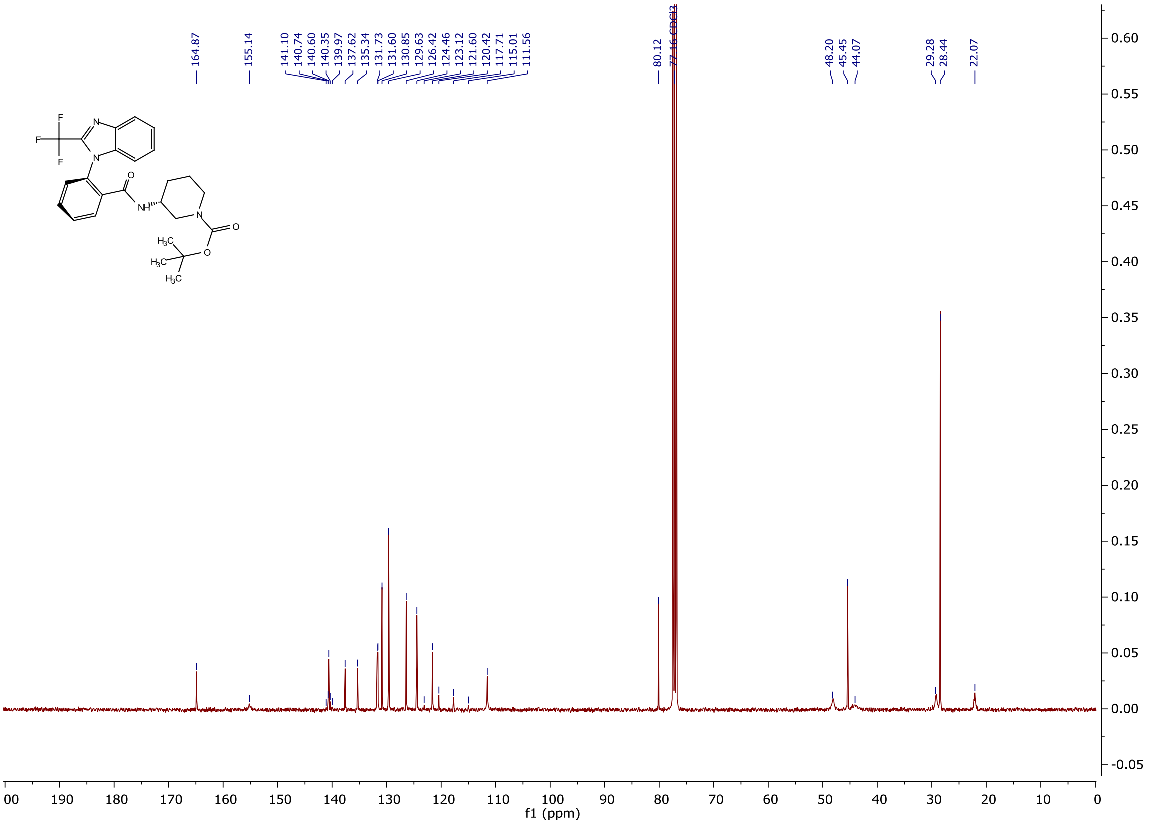

tert-Butyl (*R*)-3-(2-((*M*)-2-(trifluoromethyl)-1*H*-benzo[*d*]imidazol-1-yl)benzamido)piperidine-1-carboxylate **(*M*)-39**

^{1}H NMR (400 MHz, $CDCl_3$)

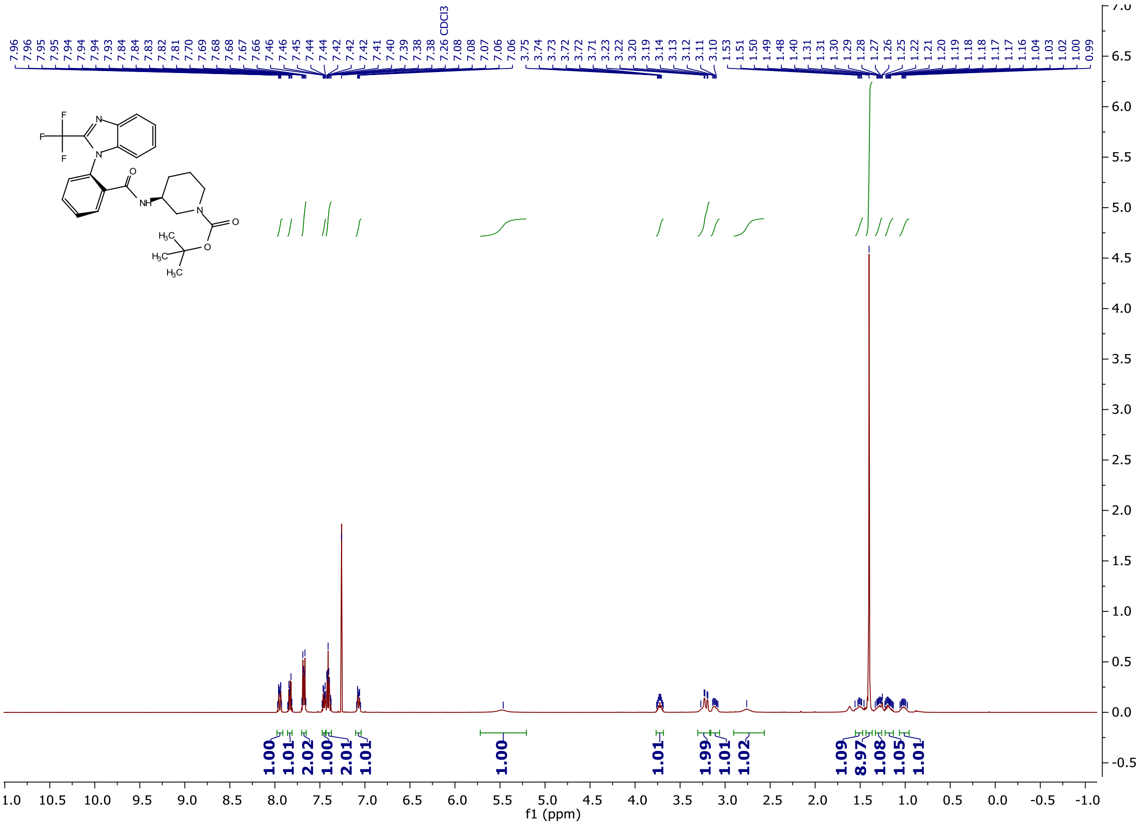

^{13}C{^{1}H} NMR (101 MHz, $CDCl_3$)

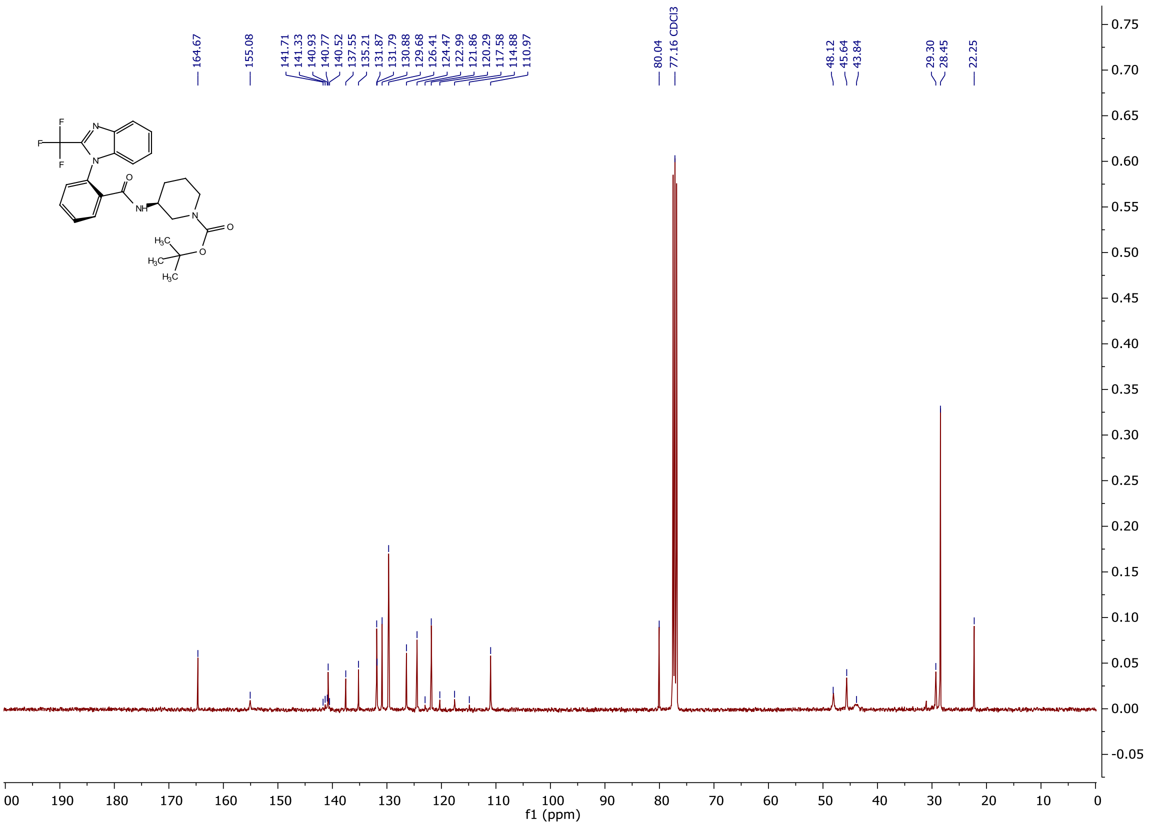

N-((1*R*,2*S*)-2-hydroxy-2,3-dihydro-1*H*-inden-1-yl)-2-((*P*)-2-(trifluoromethyl)-1*H*-benzo[*d*]imidazol-1-yl)benzamide **(*P*)-40**

^{1}H NMR (400 MHz, $CDCl_3$)

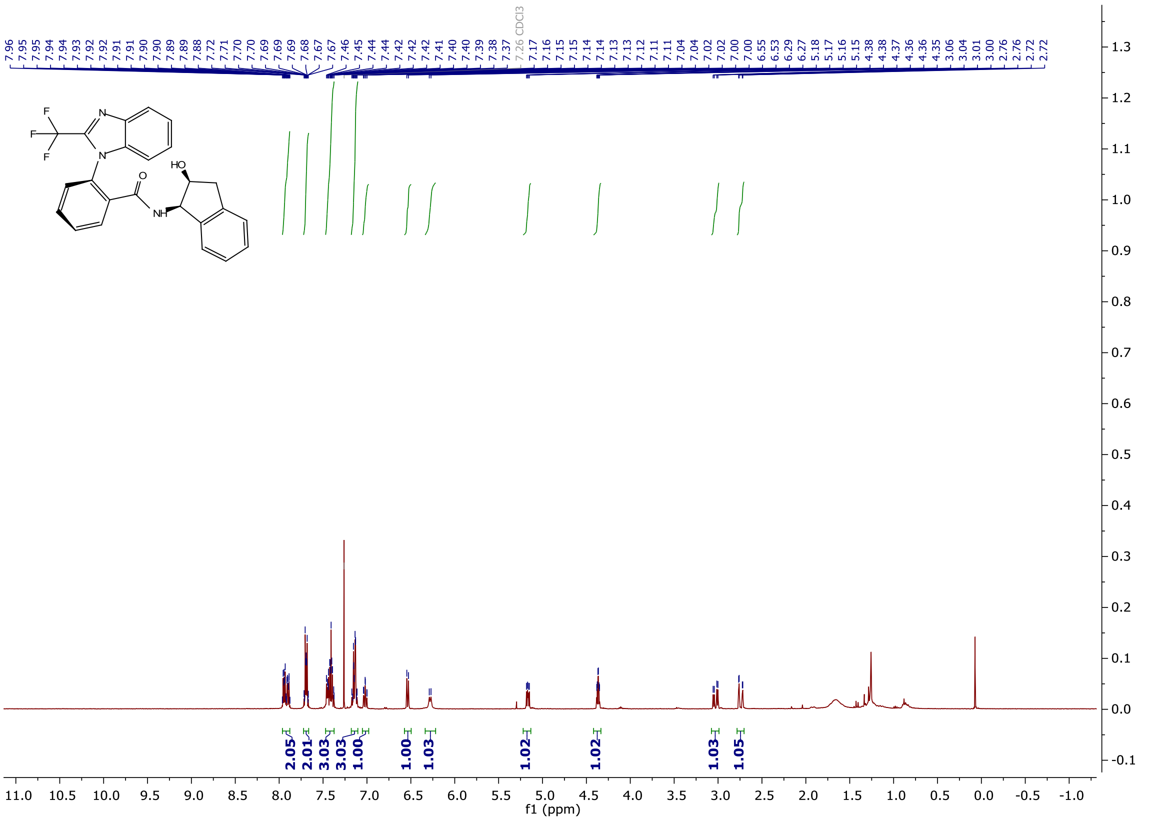

^{13}C{^{1}H} NMR (101 MHz, $CDCl_3$)

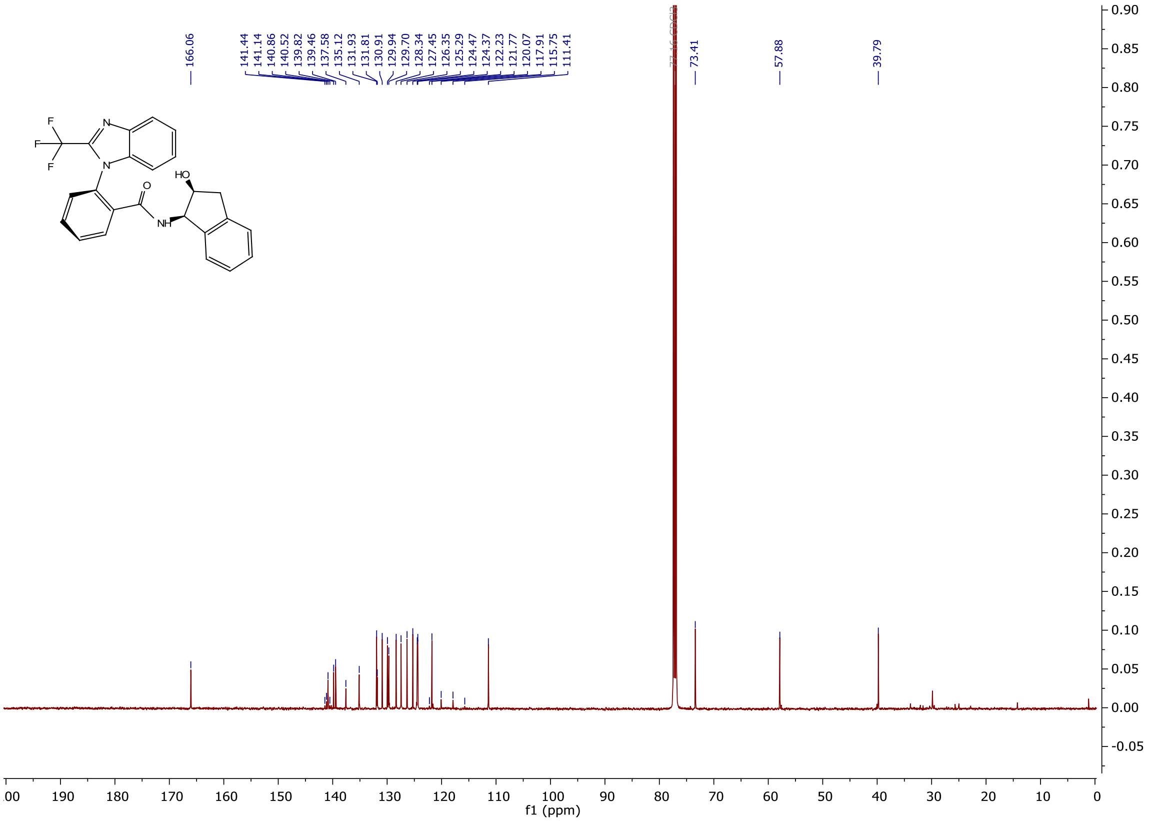

N-((1*R*,2*S*)-2-hydroxy-2,3-dihydro-1*H*-inden-1-yl)-2-((*M*)-2-(trifluoromethyl)-1*H*-benzo[*d*]imidazol-1-yl)benzamide **(*M*)-40**

^{1}H NMR (400 MHz, $CDCl_3$)

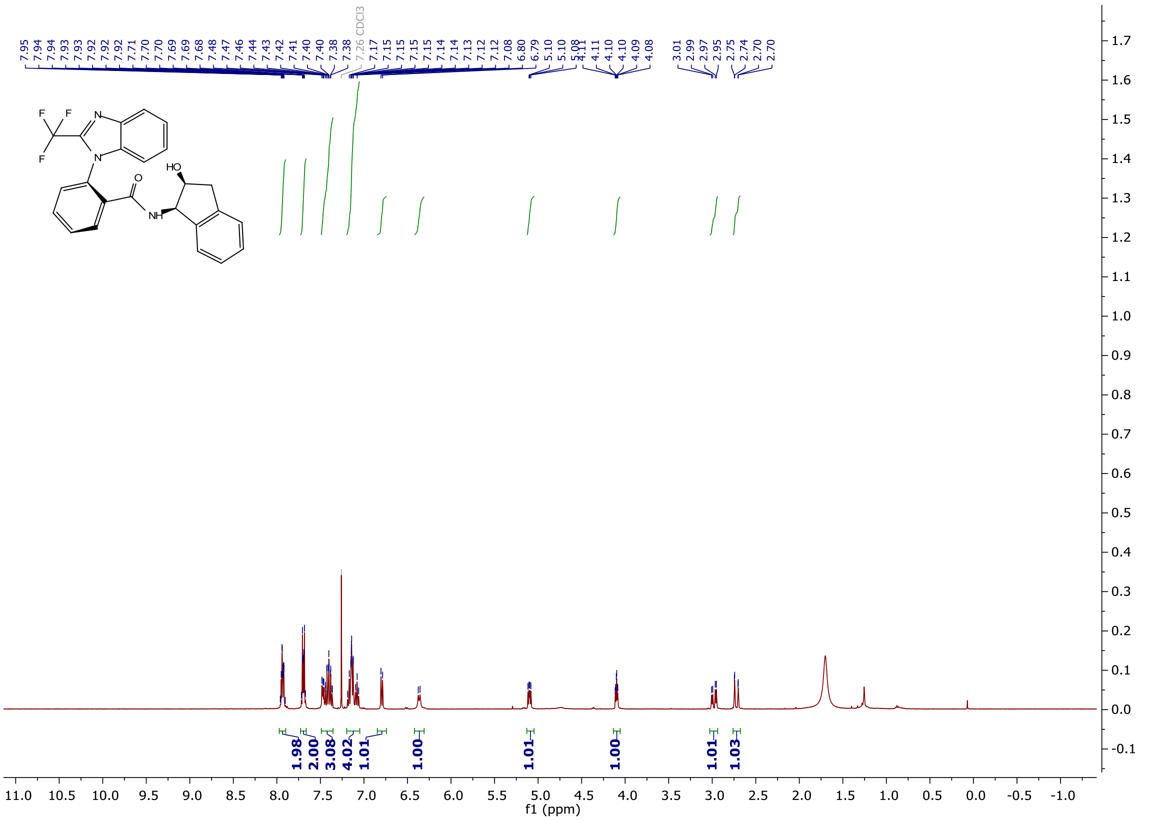

^{13}C{^{1}H} NMR (101 MHz, $CDCl_3$)

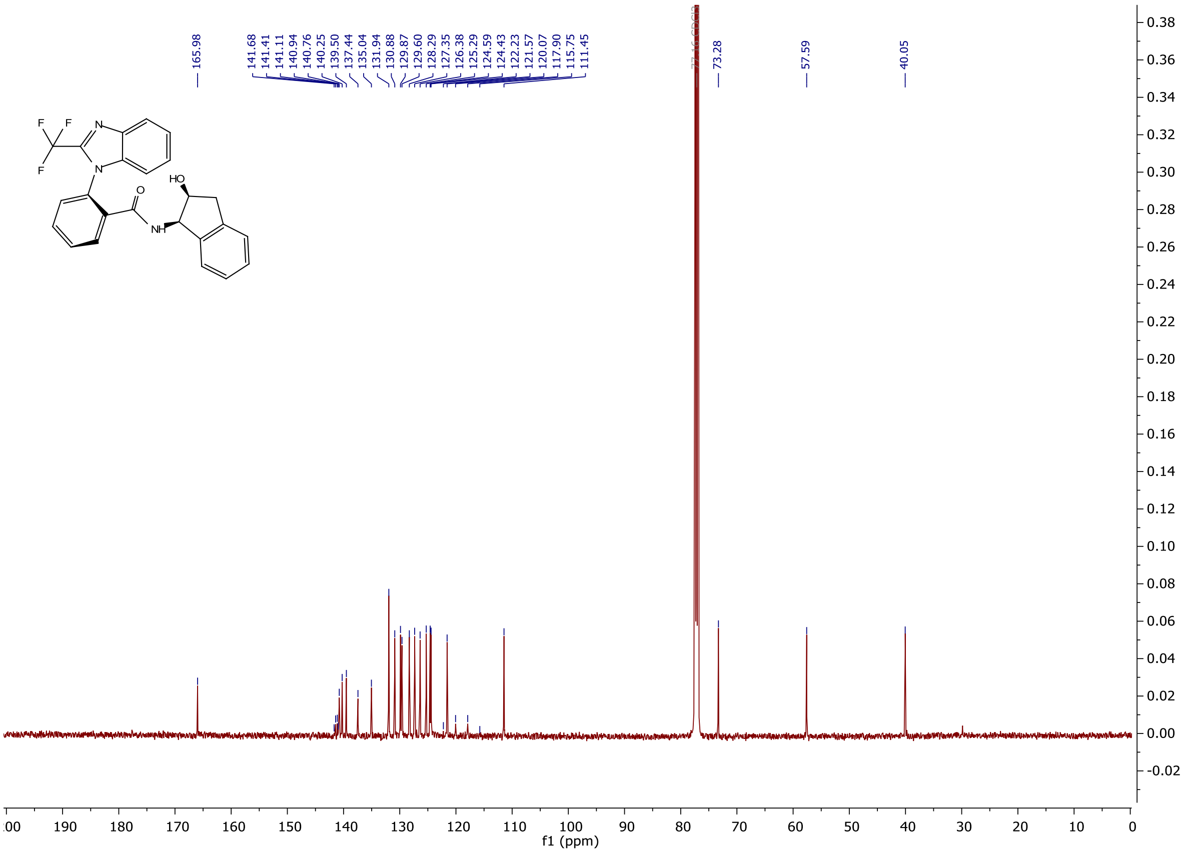

(1*R*,2*S*)-1-(2-((*P*)-2-(Trifluoromethyl)-1*H*-benzo[*d*]imidazol-1-yl)benzamido)-2,3-dihydro-1*H*-inden-2-yl acetate **(*P*)-41**

^{1}H NMR (400 MHz, $CDCl_3$)

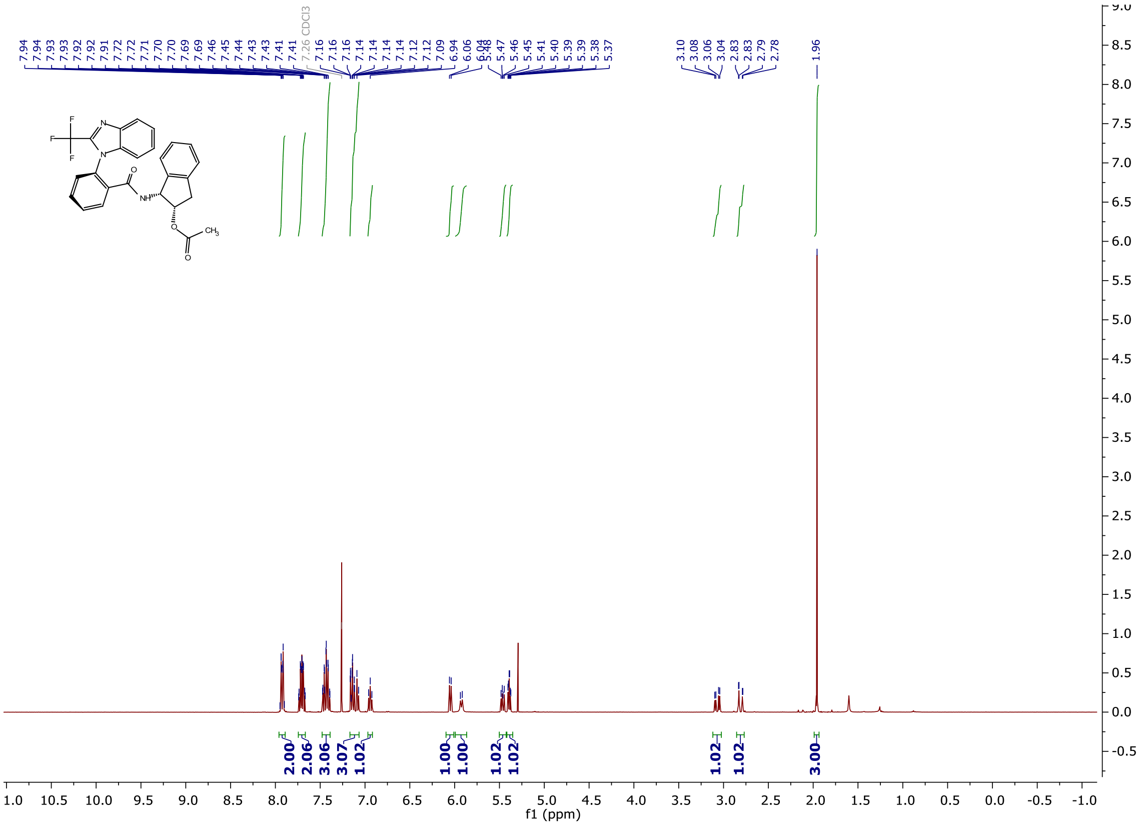

^{13}C{^{1}H} NMR (101 MHz, $CDCl_3$)

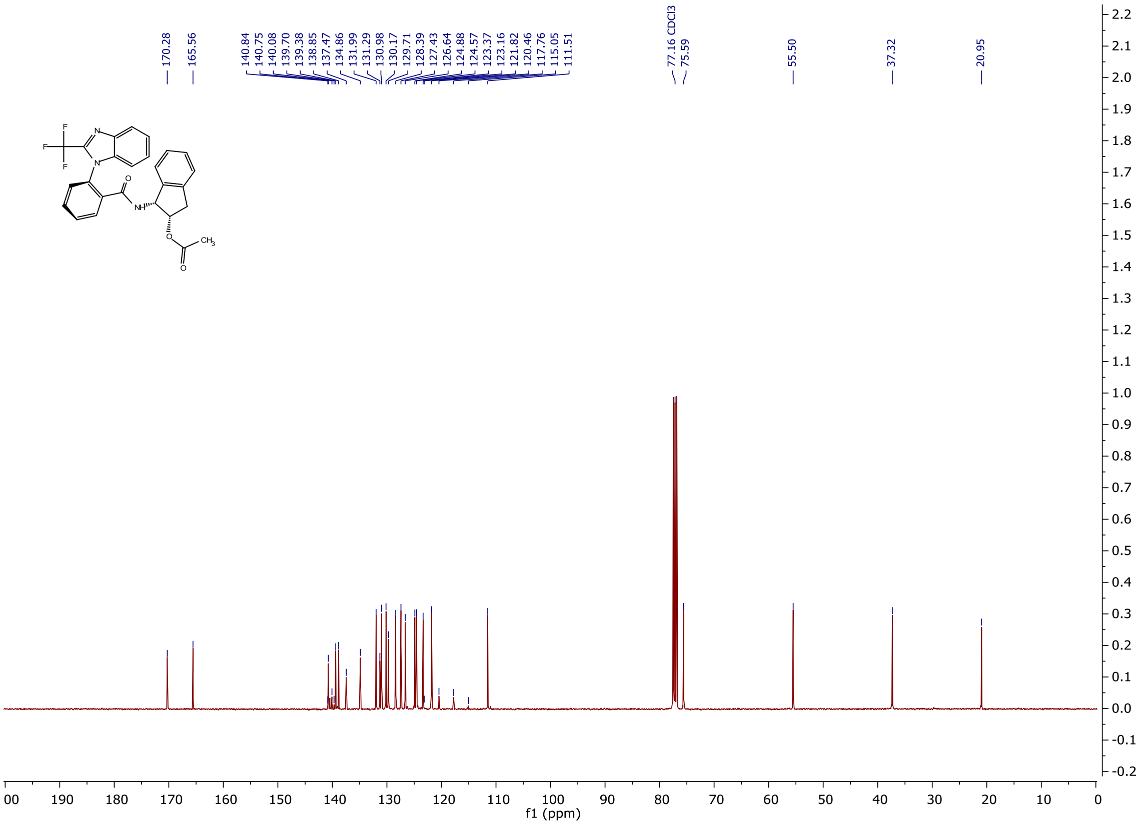

(1*R*,2*S*)-1-(2-((*M*)-2-(Trifluoromethyl)-1*H*-benzo[*d*]imidazol-1-yl)benzamido)-2,3-dihydro-1*H*-inden-2-yl acetate **(*M*)-41**

^{1}H NMR (400 MHz, $CDCl_3$)

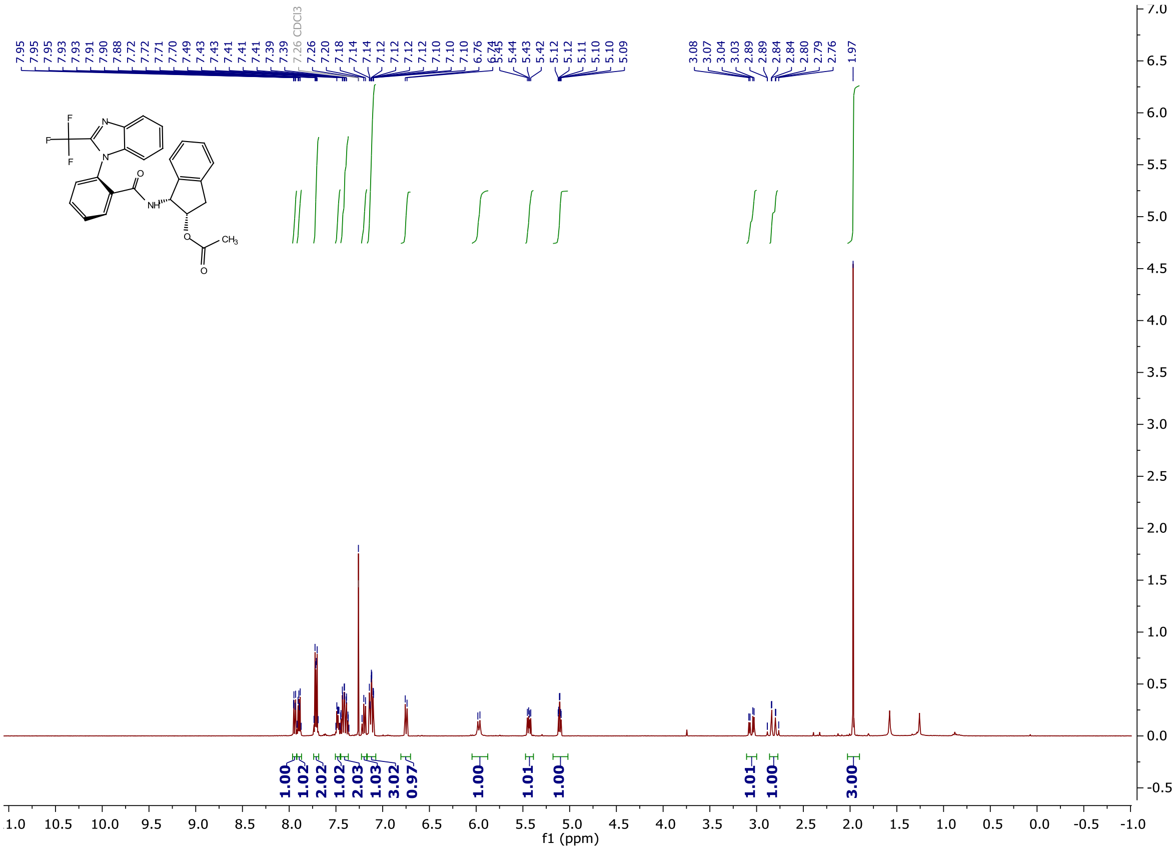

^{13}C{^{1}H} NMR (101 MHz, $CDCl_3$)

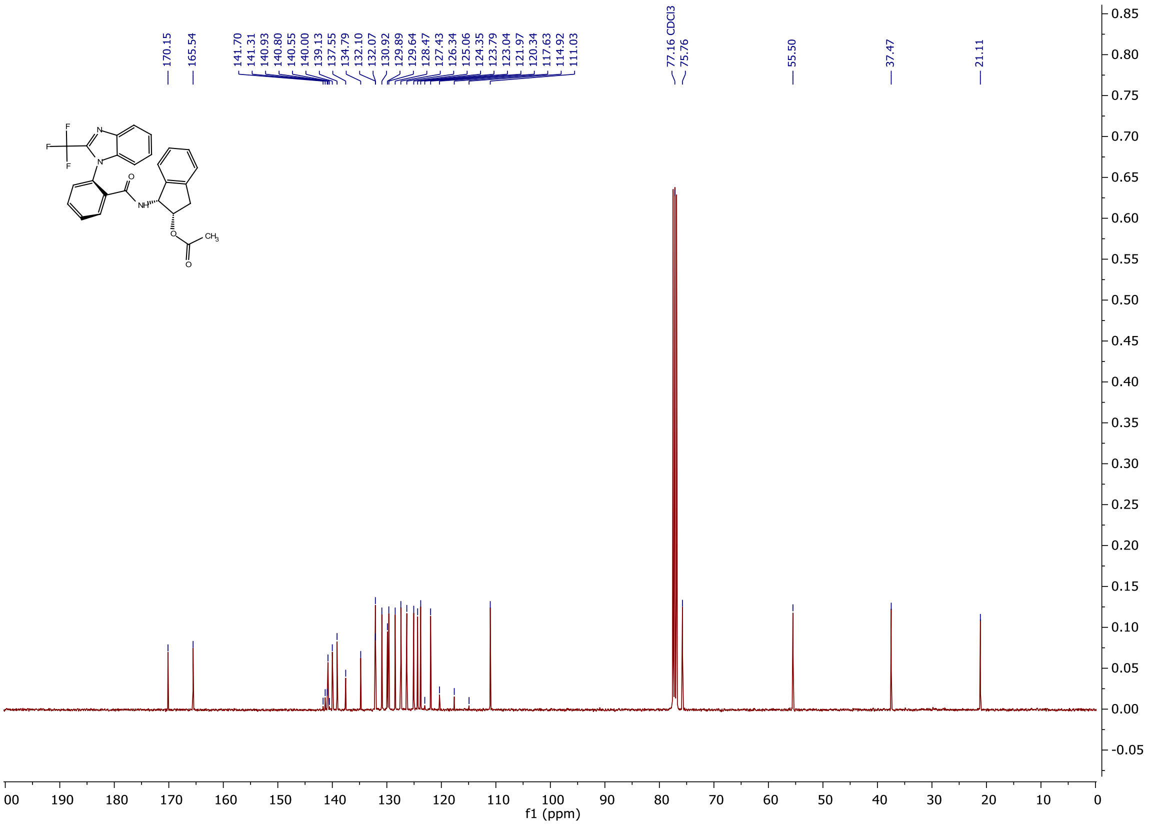

N-((*S*)-1-Hydroxy-4-methylpentan-2-yl)-2-((*P*)-2-(trifluoromethyl)-1*H*-benzo[*d*]imidazol-1-yl)benzamide **(*P*)-42**

^{1}H NMR (400 MHz, $CDCl_3$)

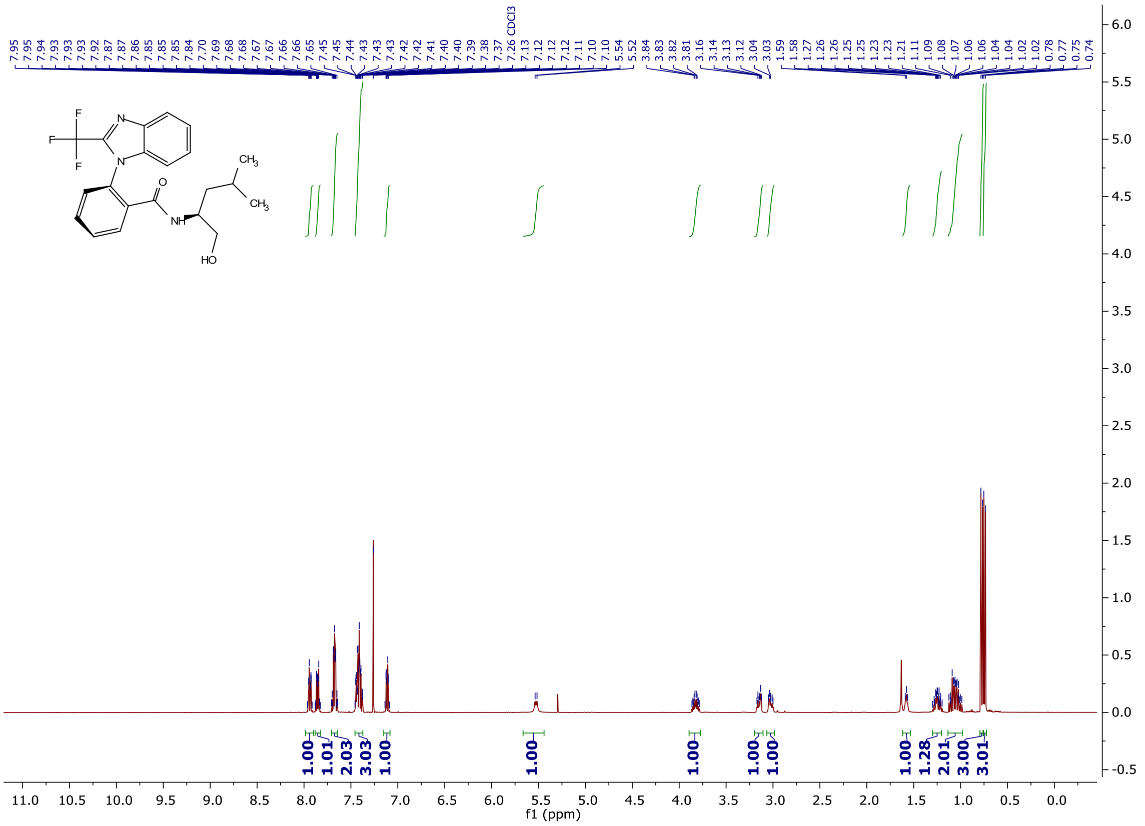

^{13}C{^{1}H} NMR (101 MHz, $CDCl_3$)

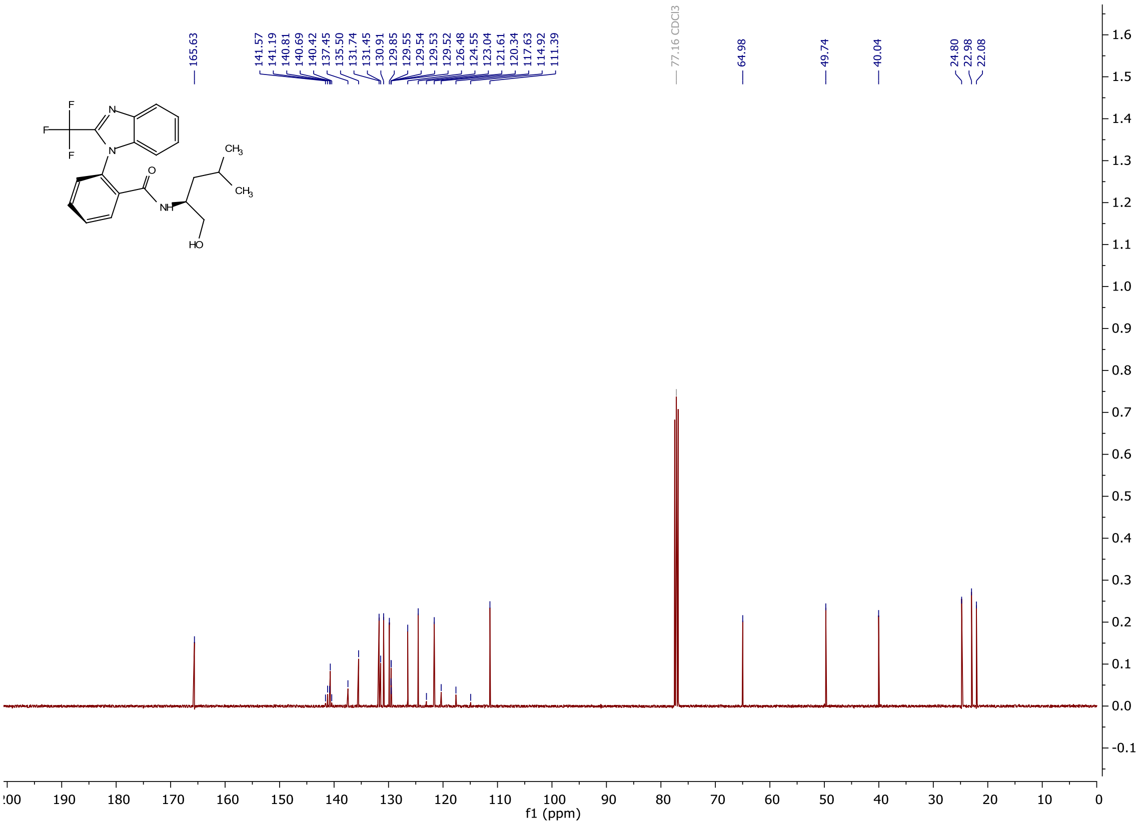

N-((*S*)-1-Hydroxy-4-methylpentan-2-yl)-2-((*M*)-2-(trifluoromethyl)-1*H*-benzo[*d*]imidazol-1-yl)benzamide **(*M*)-42**

^{1}H NMR (400 MHz, $CDCl_3$)

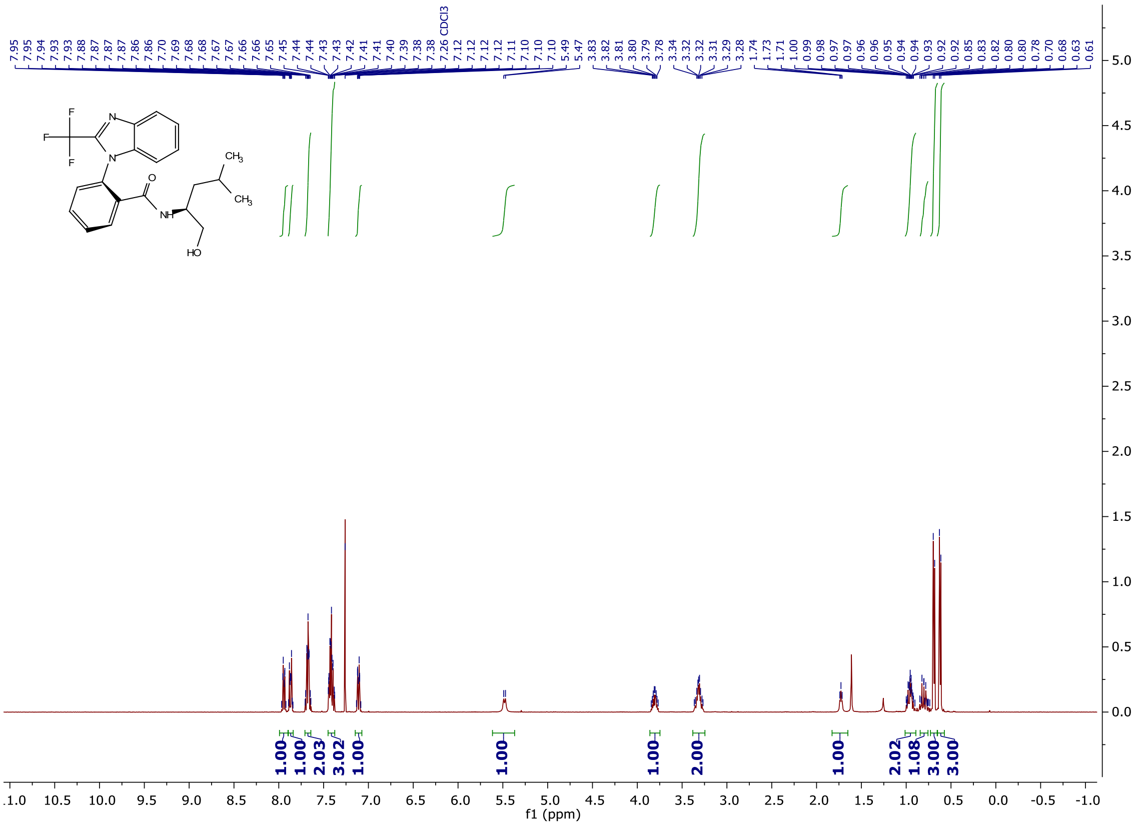

^{13}C{^{1}H} NMR (101 MHz, $CDCl_3$)

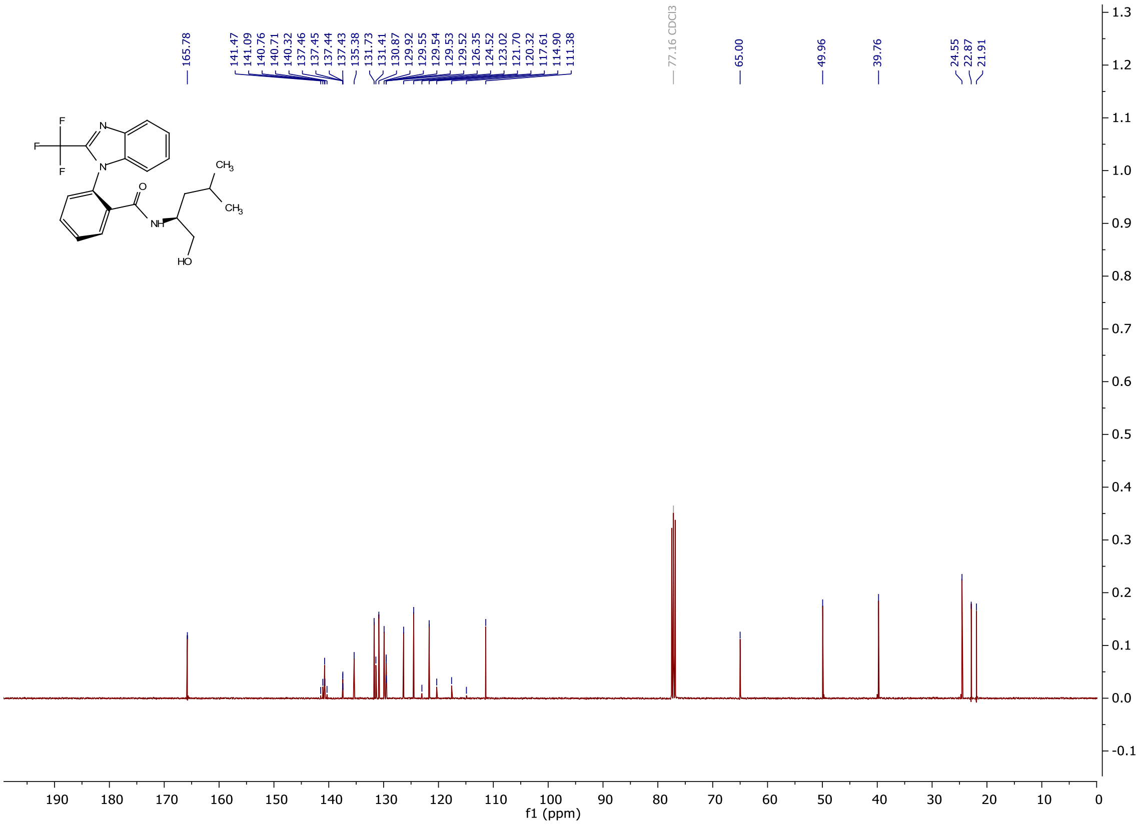

(*S*)-4-Methyl-2-(2-((*P*)-2-(trifluoromethyl)-1*H*-benzo[*d*]imidazol-1-yl)benzamido)pentyl acetate **(*P*)-43**

^{1}H NMR (400 MHz, $CDCl_3$)

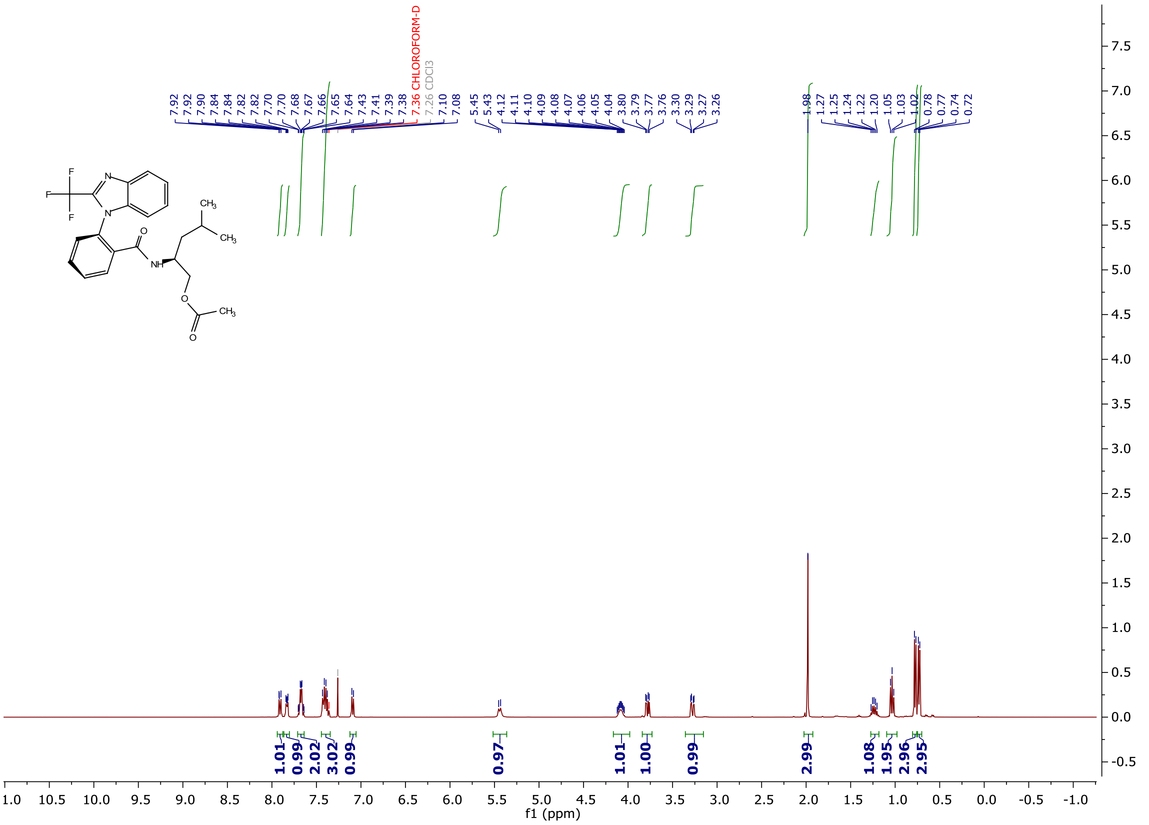

^{13}C{^{1}H} NMR (101 MHz, $CDCl_3$)

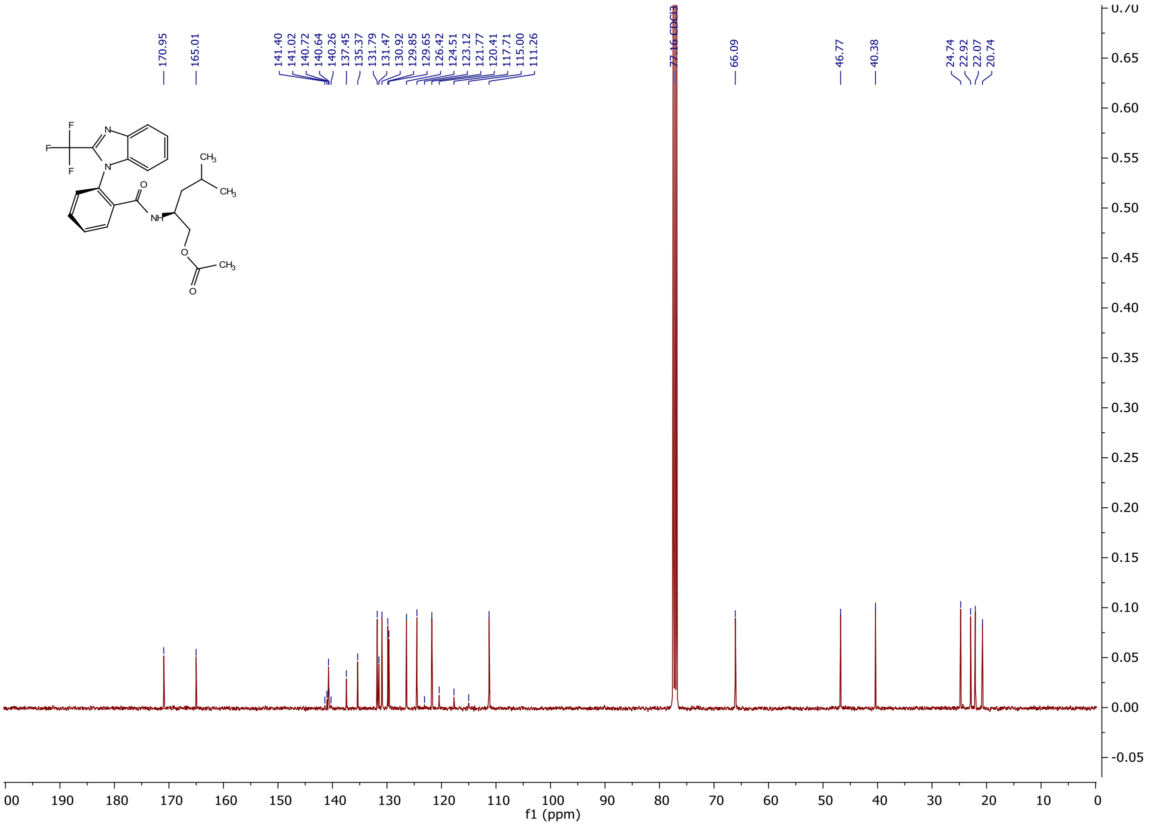

(*S*)-4-Methyl-2-(2-((*M*)-2-(trifluoromethyl)-1*H*-benzo[*d*]imidazol-1-yl)benzamido)pentyl acetate **(*M*)-43**

^{1}H NMR (400 MHz, $CDCl_3$)

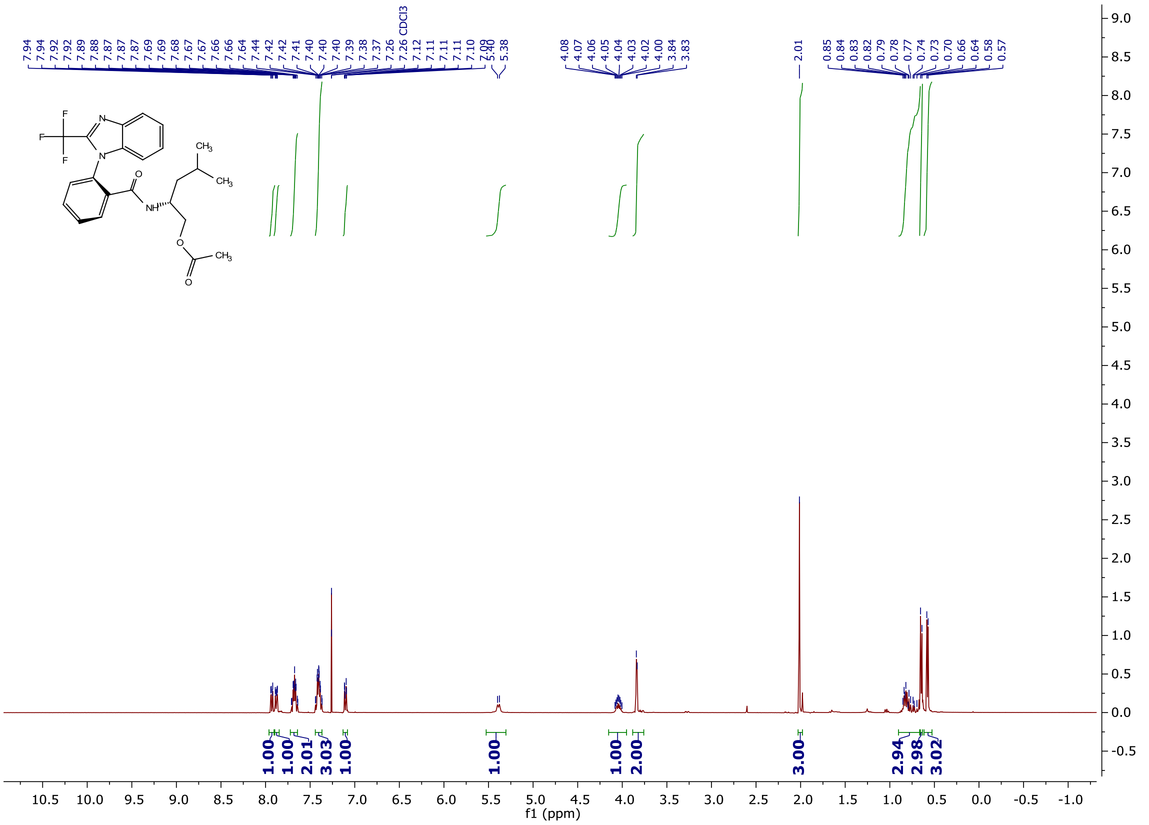

^{13}C{^{1}H} NMR (101 MHz, $CDCl_3$)

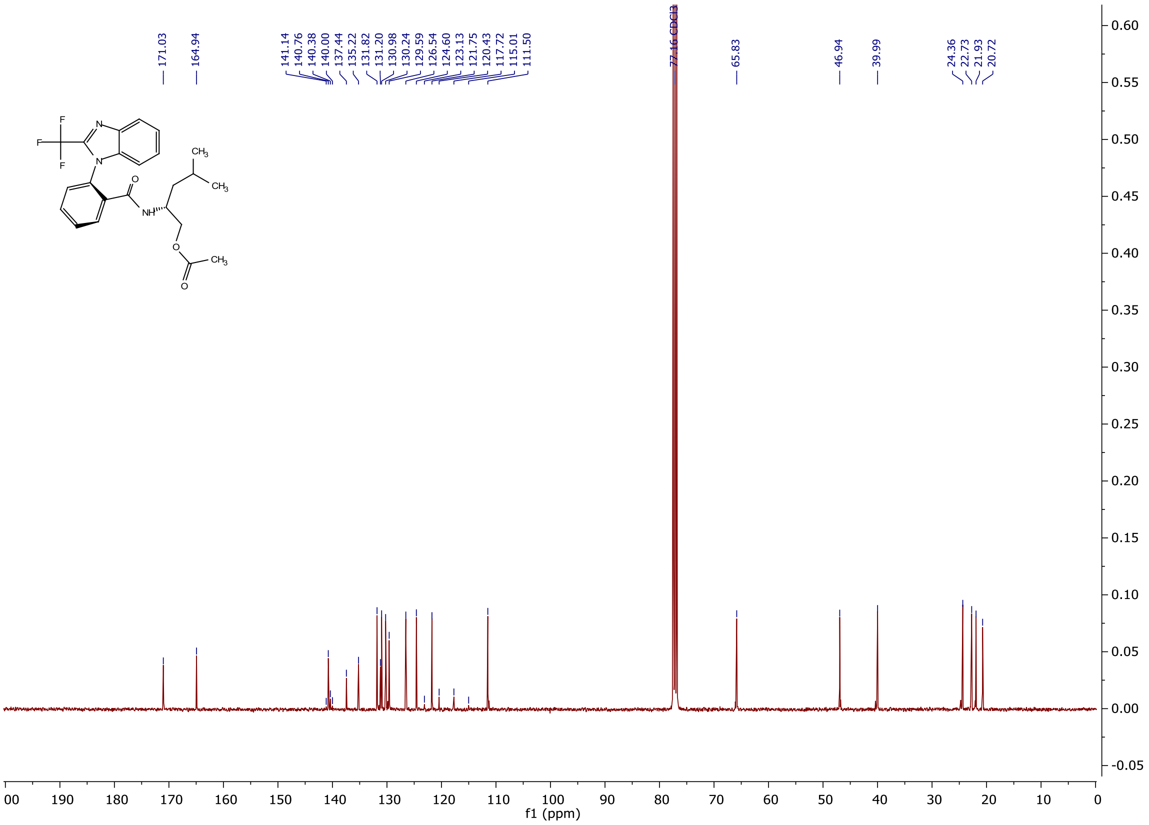

N-((1*R*,2*R*)-1,3-Dihydroxy-1-(4-nitrophenyl)propan-2-yl)-2-((*P*)-2-(trifluoromethyl)-1*H*-benzo[*d*]imidazol-1-yl)benzamide **(*P*)-44**

^{1}H NMR (400 MHz, $CDCl_3$)

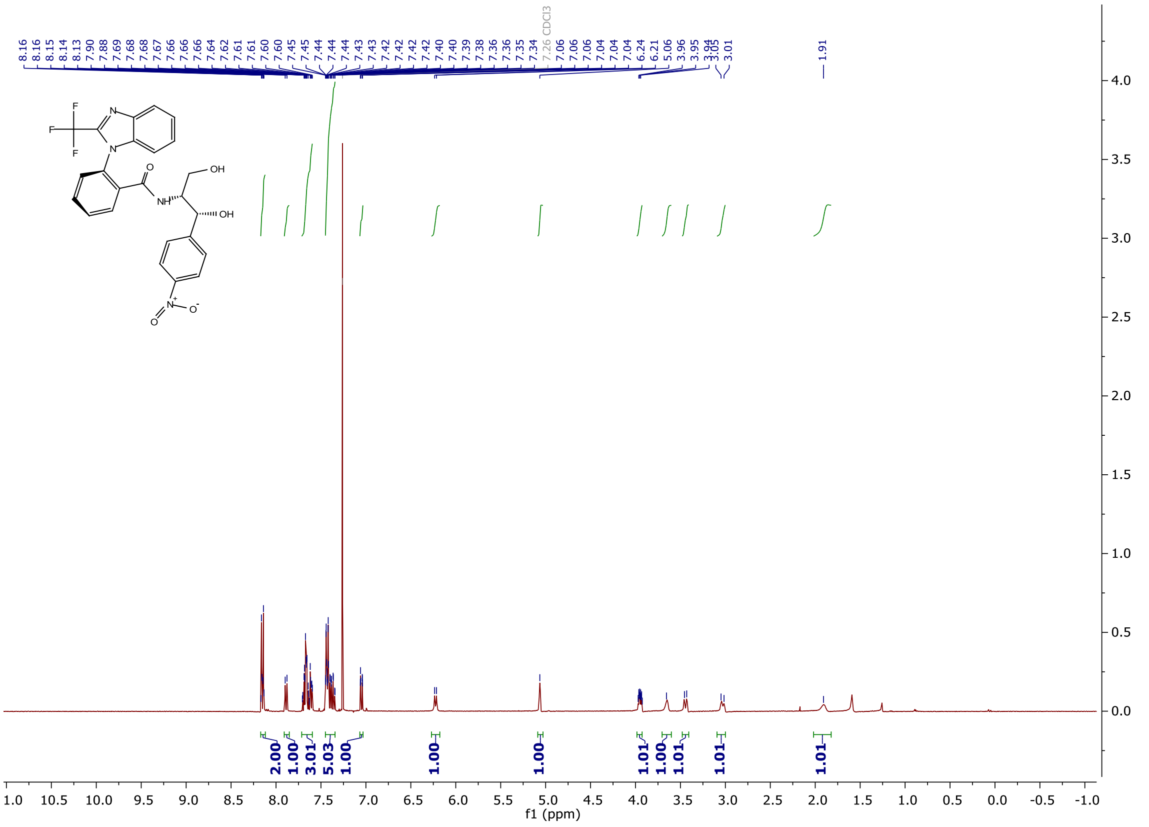

^{13}C{^{1}H} NMR (101 MHz, $CDCl_3$)

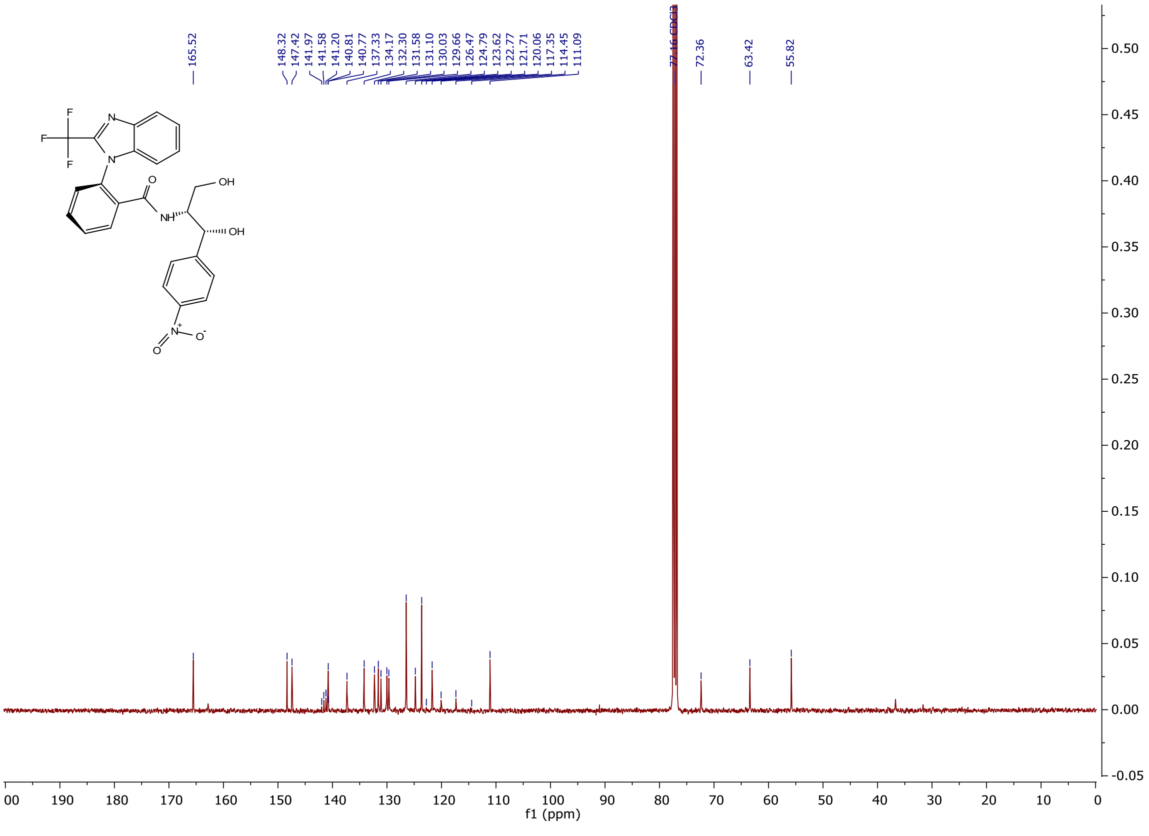

N-((1*R*,2*R*)-1,3-Dihydroxy-1-(4-nitrophenyl)propan-2-yl)-2-((*M*)-2-(trifluoromethyl)-1*H*-benzo[*d*]imidazol-1-yl)benzamide **(*M*)-44**

^{1}H NMR (400 MHz, $CDCl_3$)

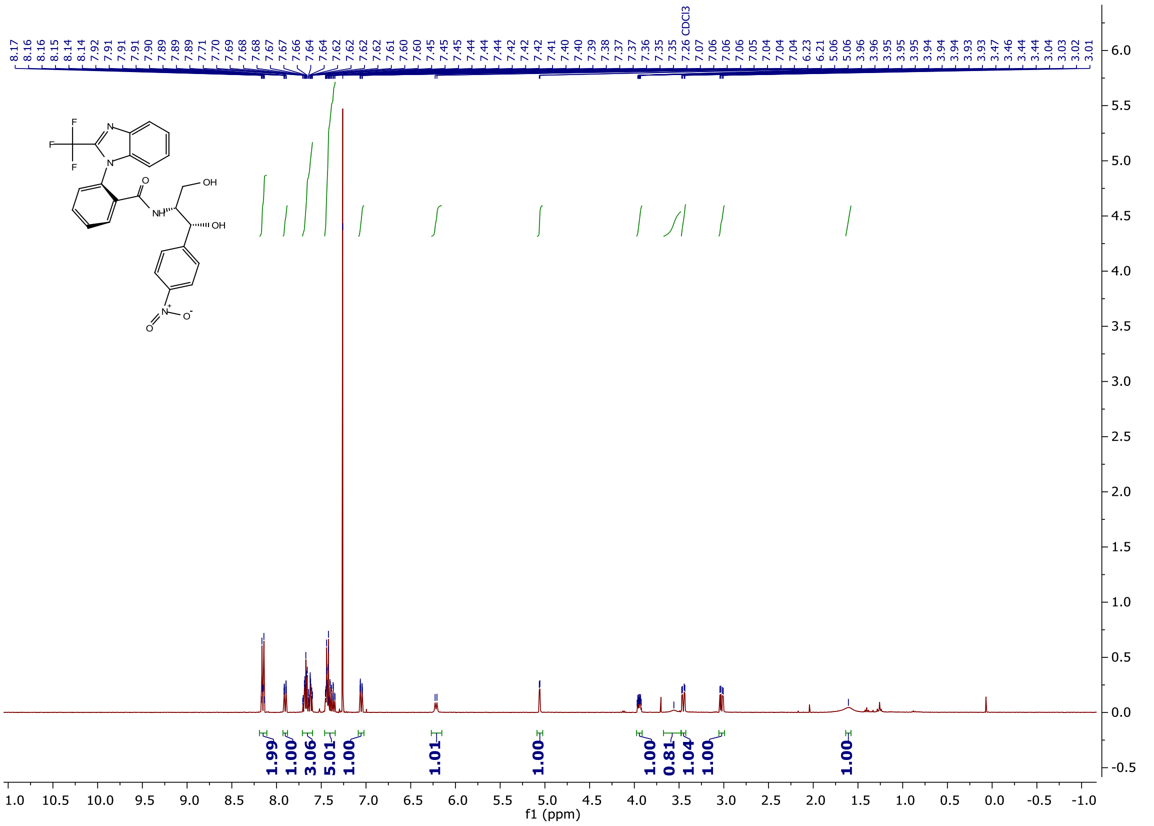

^{13}C{^{1}H} NMR (101 MHz, $CDCl_3$)

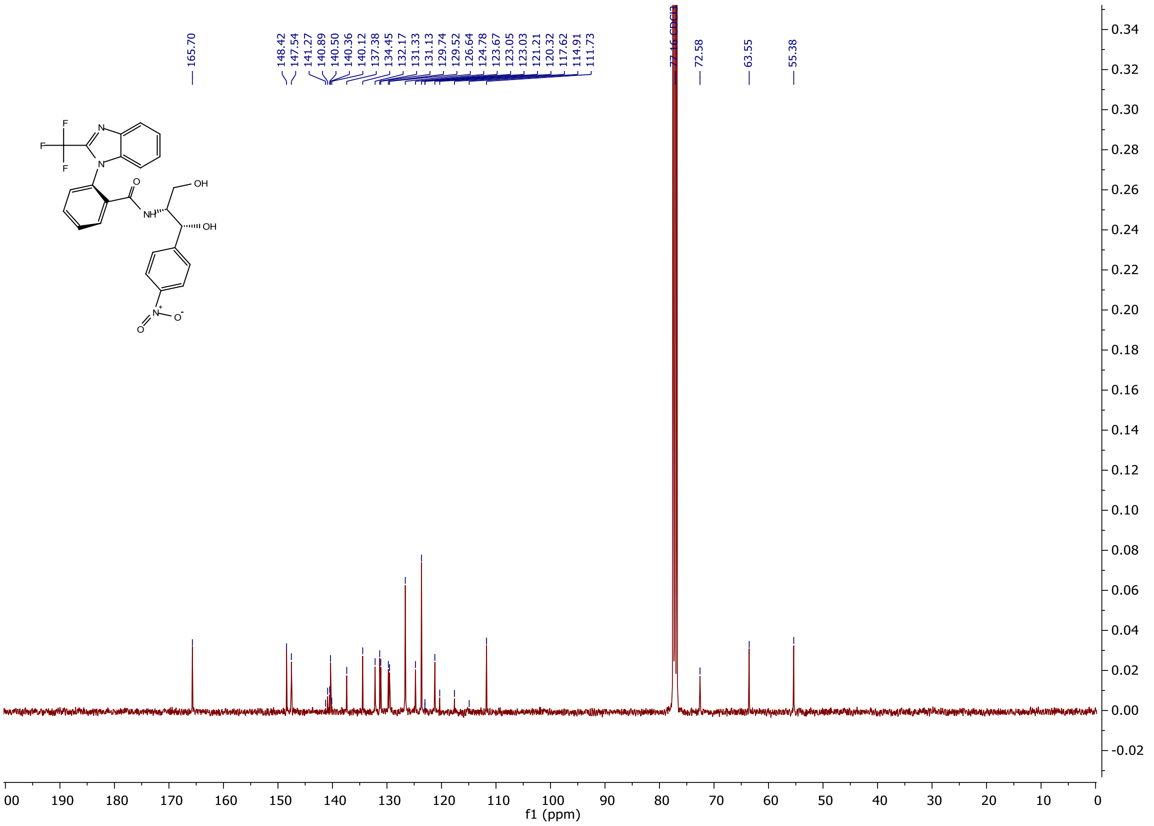

(1*R*,2*R*)-1-(4-Nitrophenyl)-2-(2-((*P*)-2-(trifluoromethyl)-1*H*-benzo[*d*]imidazol-1-yl)benzamido)propane-1,3-diyl diacetate **(*P*)-45**

^{1}H NMR (400 MHz, CDCl$_3$)

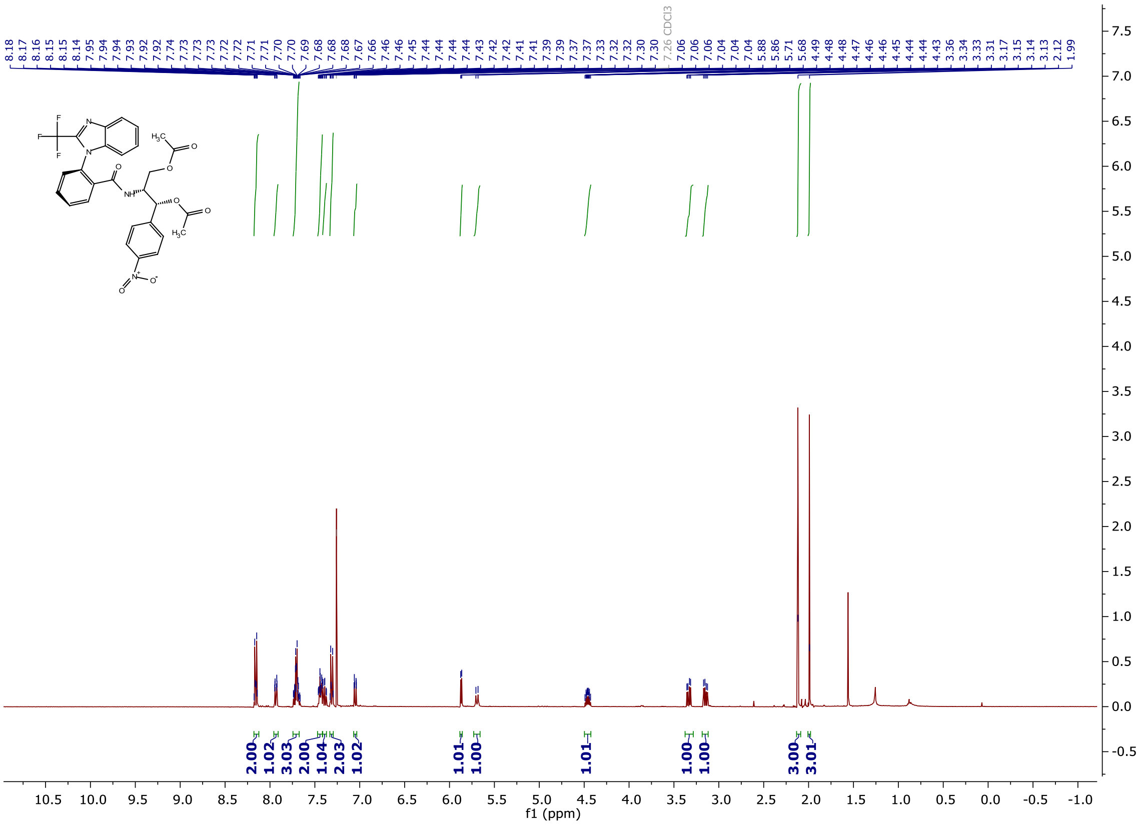

^{13}C{^{1}H} NMR (101 MHz, CDCl$_3$)

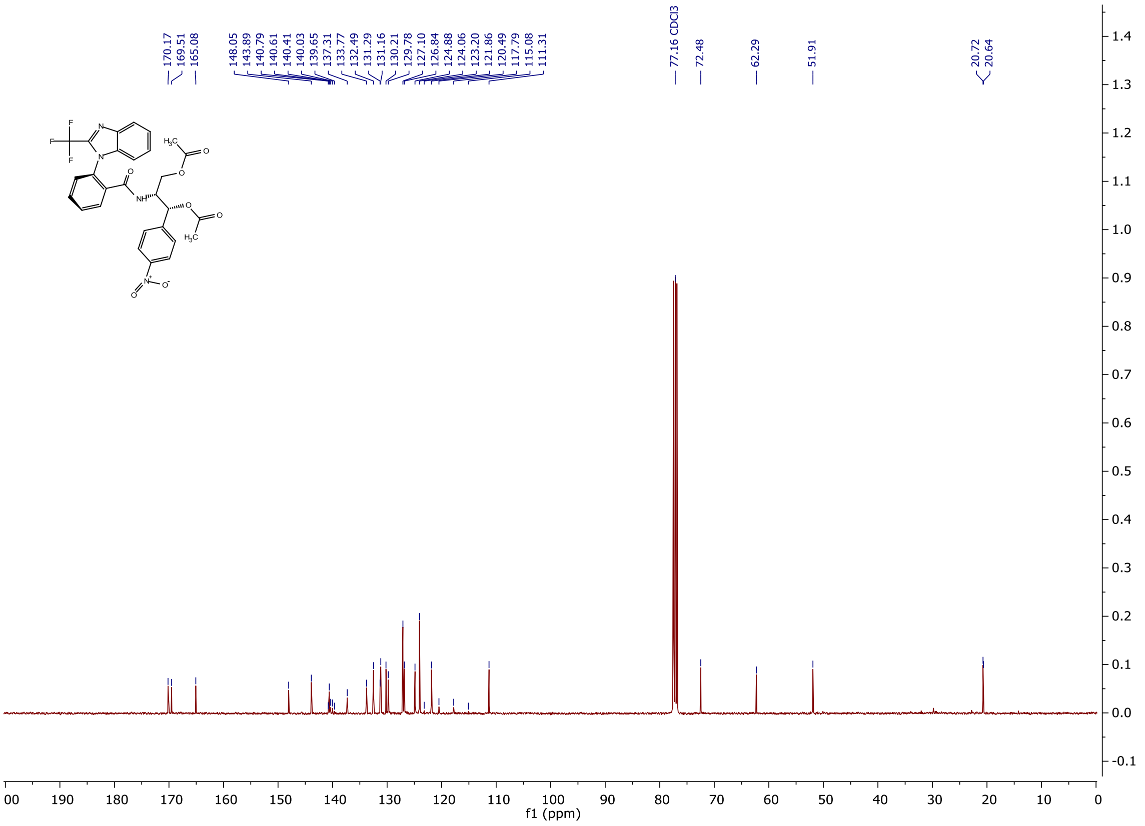

(1*R*,2*R*)-1-(4-Nitrophenyl)-2-(2-((*M*)-2-(trifluoromethyl)-1*H*-benzo[*d*]imidazol-1-yl)benzamido)propane-1,3-diyl diacetate **(*M*)-45**

^{1}H NMR (400 MHz, $CDCl_3$)

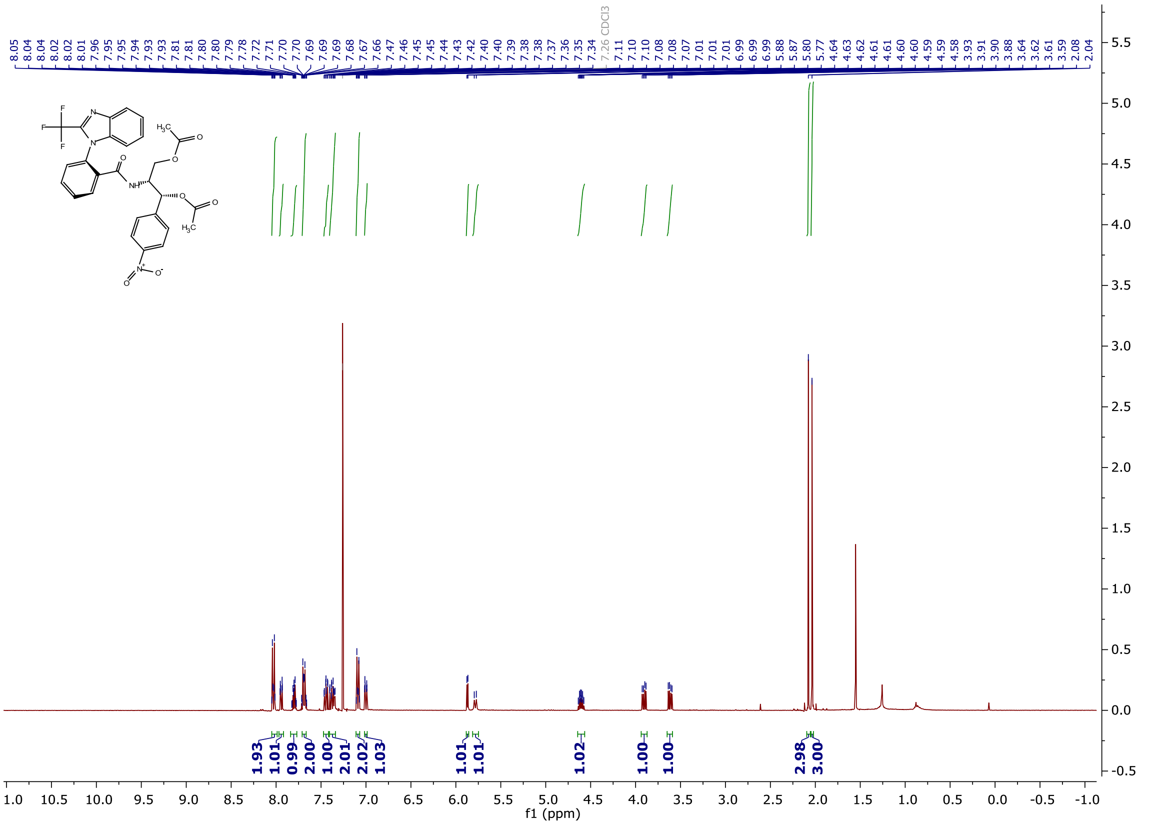

^{13}C{^{1}H} NMR (101 MHz, $CDCl_3$)

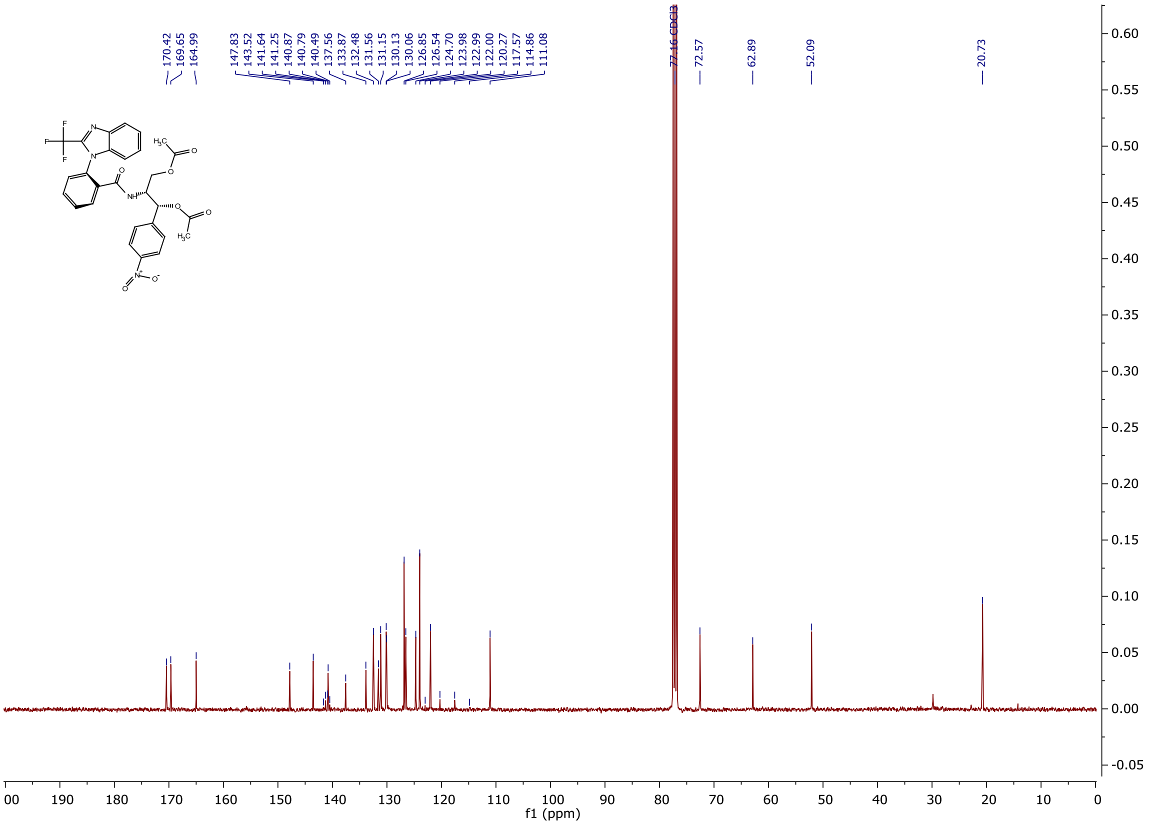

N-((4*R*,5*R*)-2,2-Dimethyl-4-(4-nitrophenyl)-1,3-dioxan-5-yl)-2-((*P*)-2-(trifluoromethyl)-1*H*-benzo[*d*]imidazol-1-yl)benzamide **(*P*)-46**

^{1}H NMR (400 MHz, $CDCl_3$)

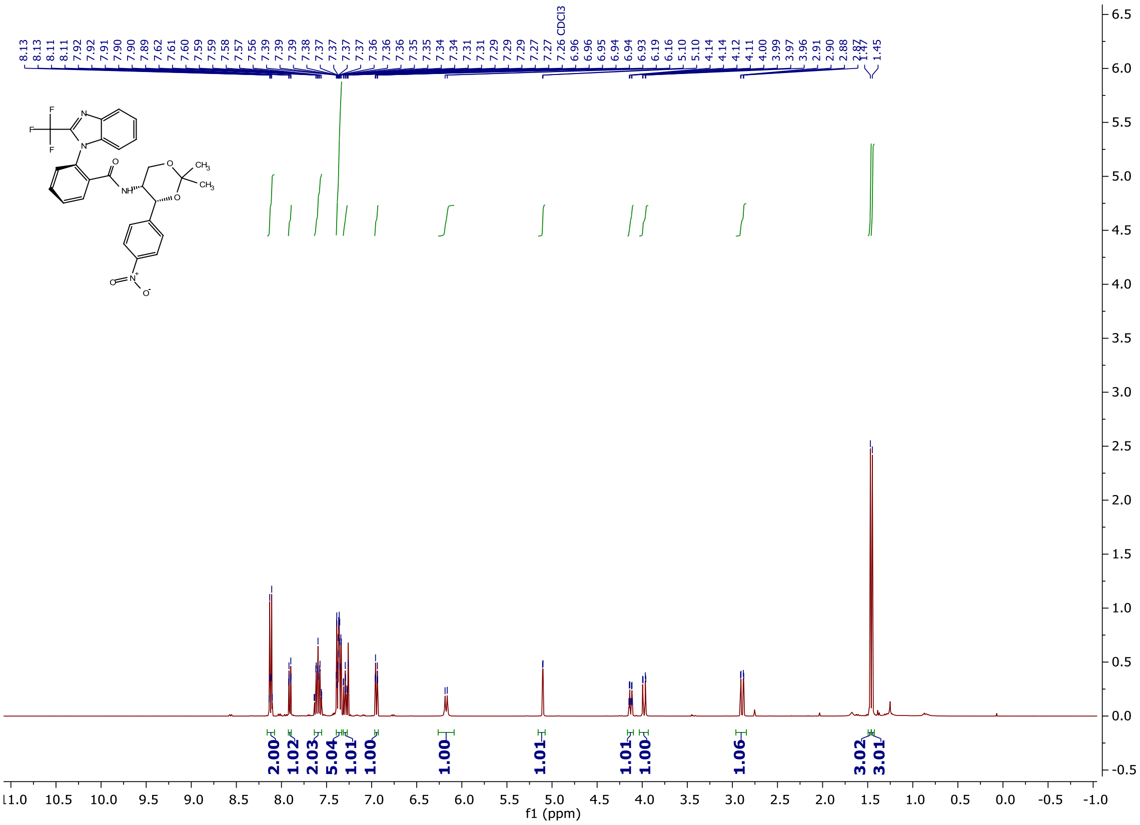

^{13}C{^{1}H} NMR (101 MHz, $CDCl_3$)

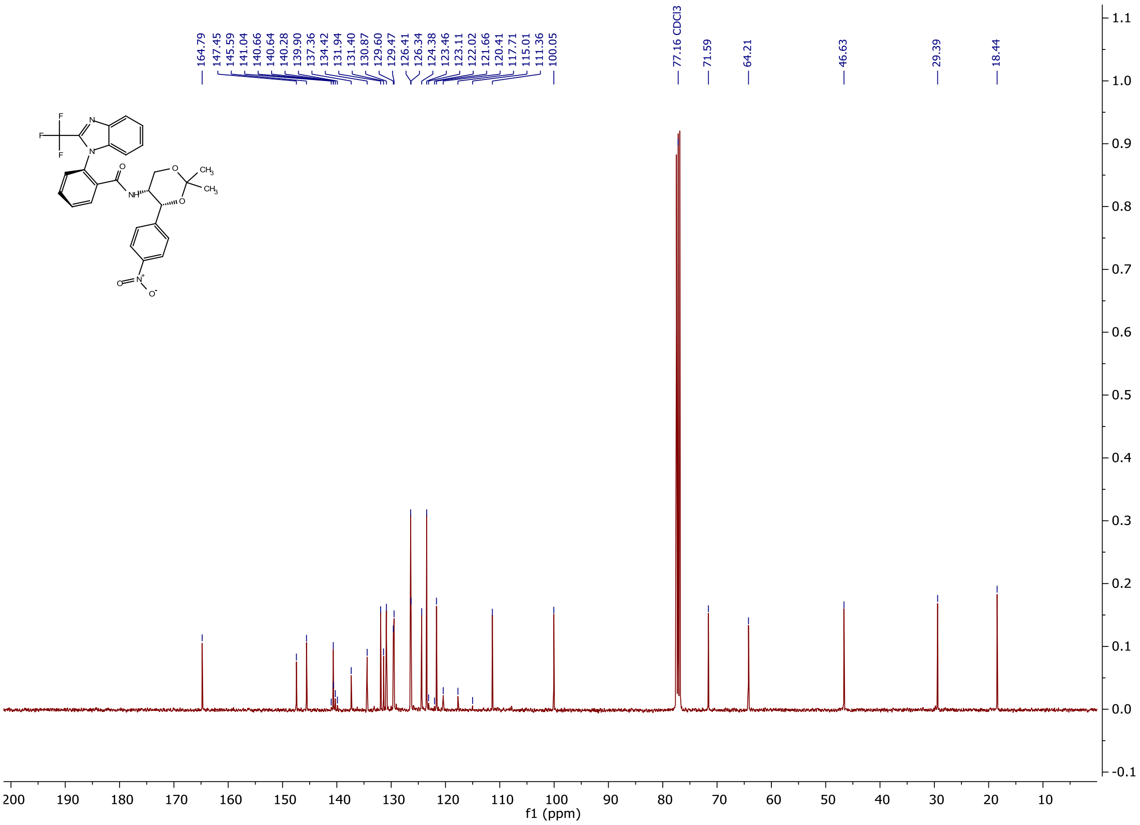

N-((4*R*,5*R*)-2,2-Dimethyl-4-(4-nitrophenyl)-1,3-dioxan-5-yl)-2-((*M*)-2-(trifluoromethyl)-1*H*-benzo[*d*]imidazol-1-yl)benzamide **(*M*)-46**

^{1}H NMR (400 MHz, $CDCl_3$)

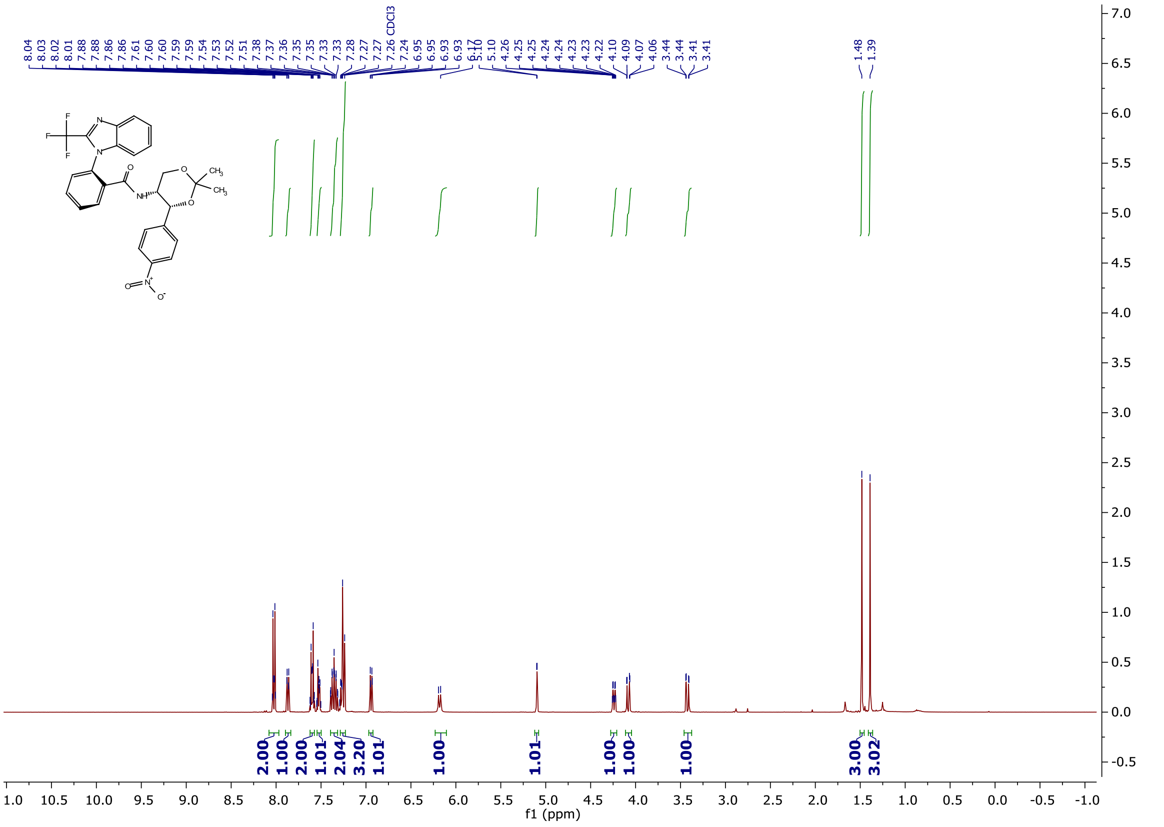

^{13}C{^{1}H} NMR (101 MHz, $CDCl_3$)

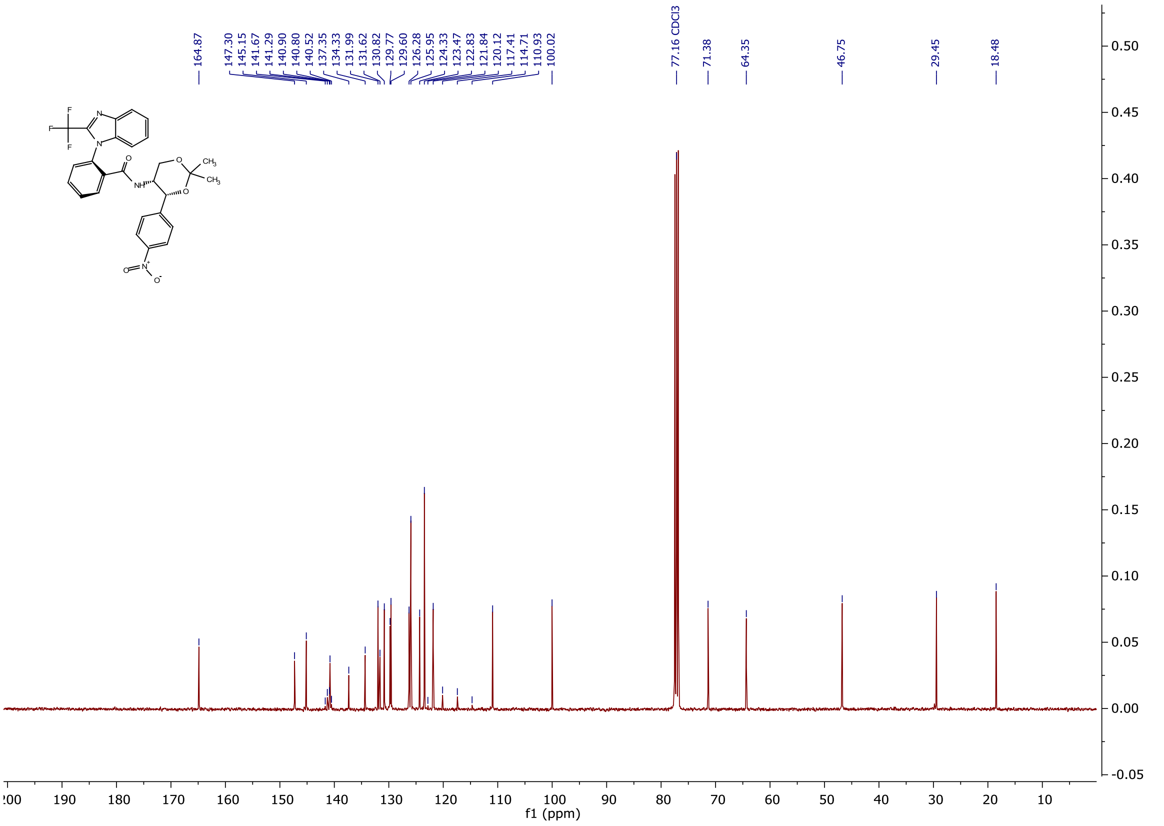

N-((4*R*,5*R*)-4-(4-Aminophenyl)-2,2-dimethyl-1,3-dioxan-5-yl)-2-((*P*)-2-(trifluoromethyl)-1*H*-benzo[*d*]imidazol-1-yl)benzamide **(*P*)-47**

^{1}H NMR (400 MHz, $CDCl_3$)

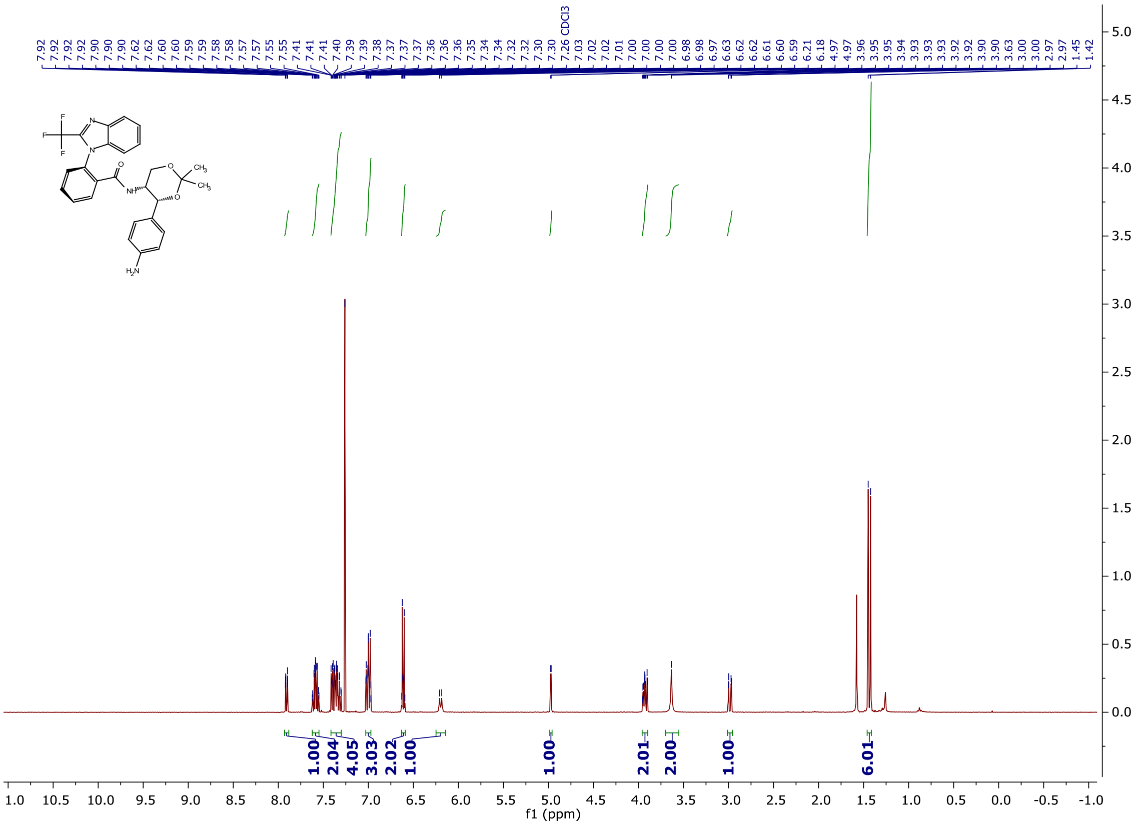

^{13}C{^{1}H} NMR (101 MHz, $CDCl_3$)

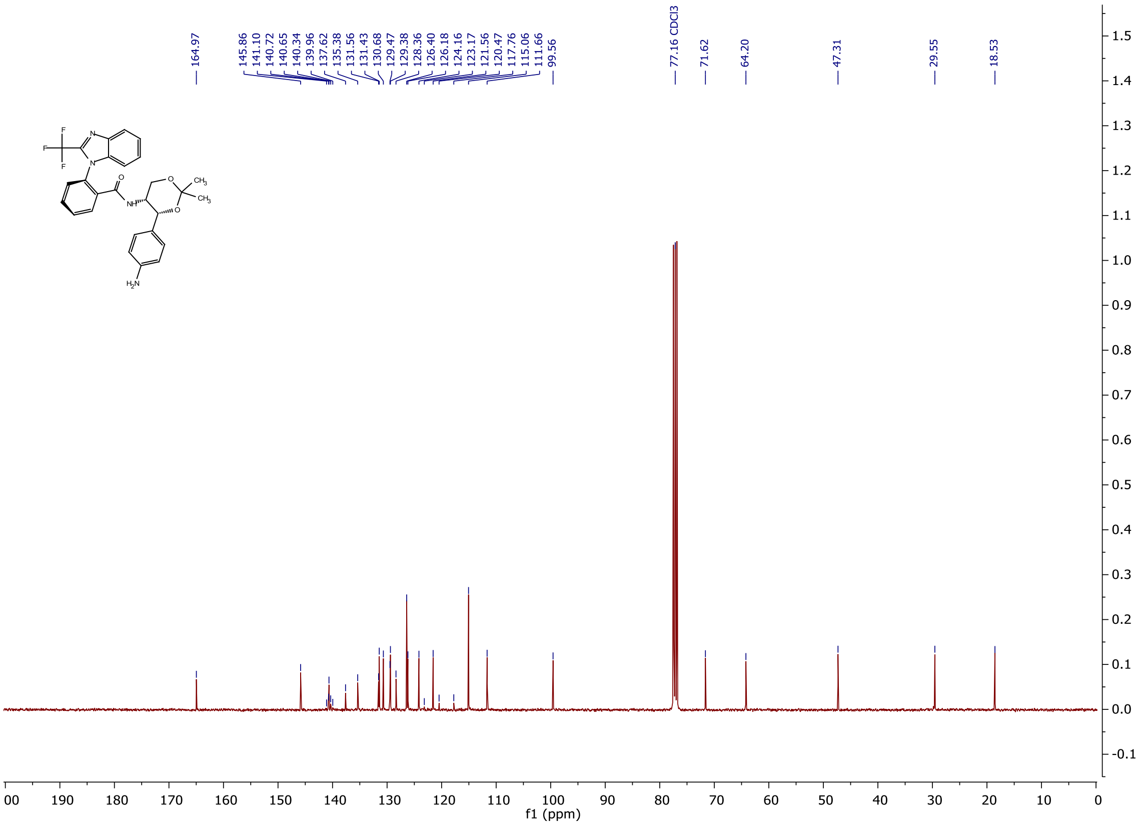

N-((4*R*,5*R*)-4-(4-Aminophenyl)-2,2-dimethyl-1,3-dioxan-5-yl)-2-((*M*)-2-(trifluoromethyl)-1*H*-benzo[*d*]imidazol-1-yl)benzamide **(*M*)-47**

^{1}H NMR (400 MHz, $CDCl_3$)

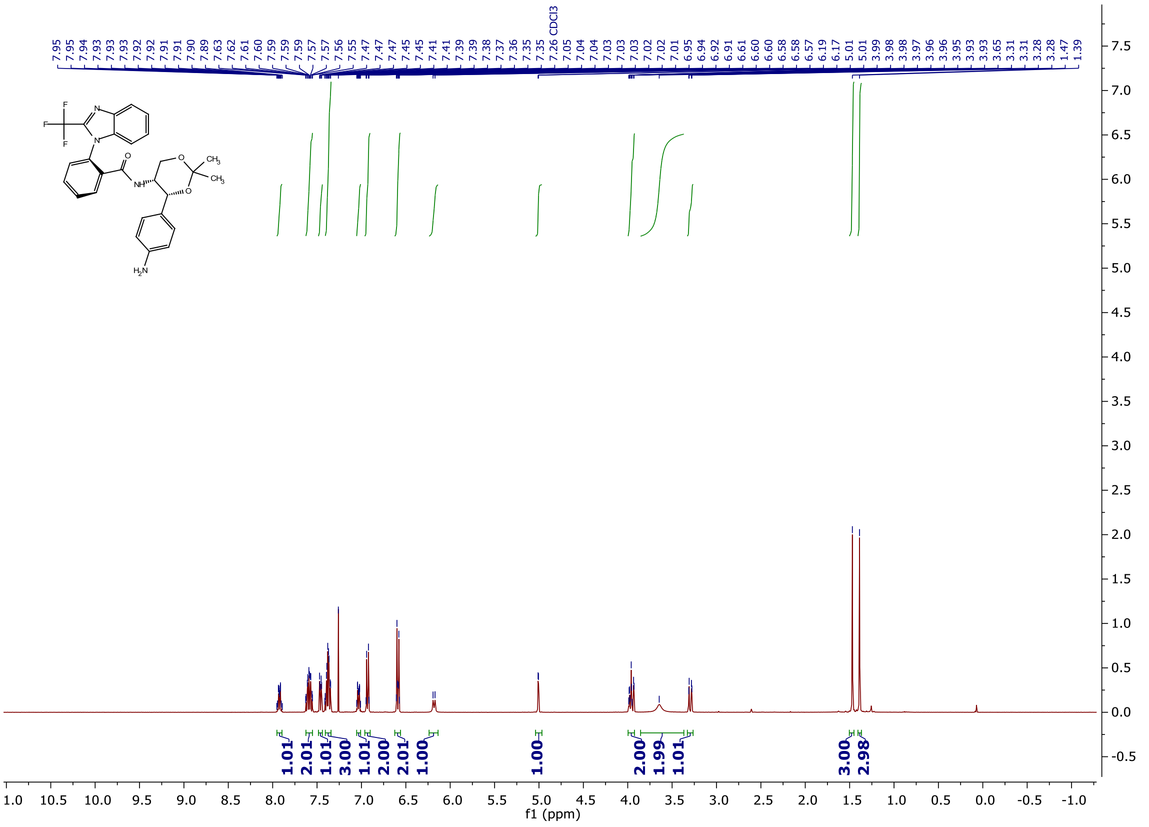

^{13}C{^{1}H} NMR (101 MHz, $CDCl_3$)

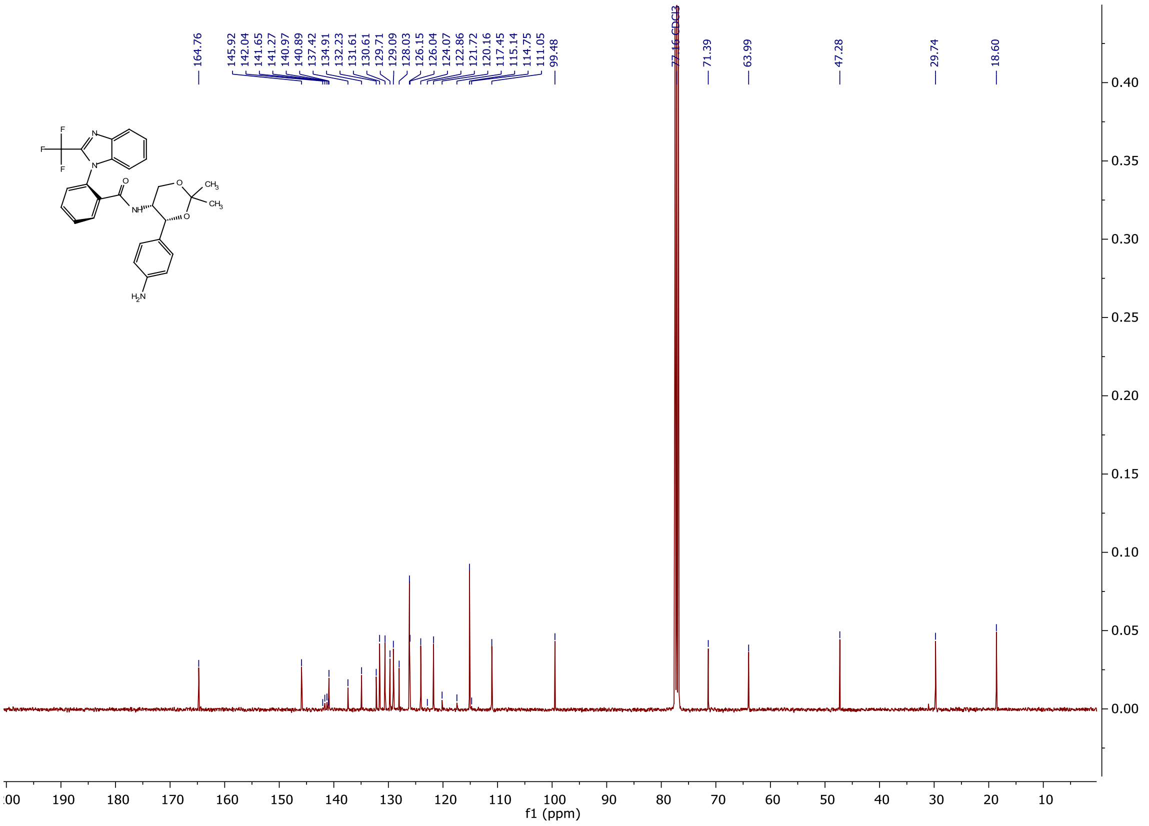

N-((1S,2*S*)-2-Amino-1,2-diphenylethyl)-2-((*P*)-2-(trifluoromethyl)-1*H*-benzo[*d*]imidazol-1-yl)benzamide **(*P*)-48**

^{1}H NMR (400 MHz, $CDCl_3$)

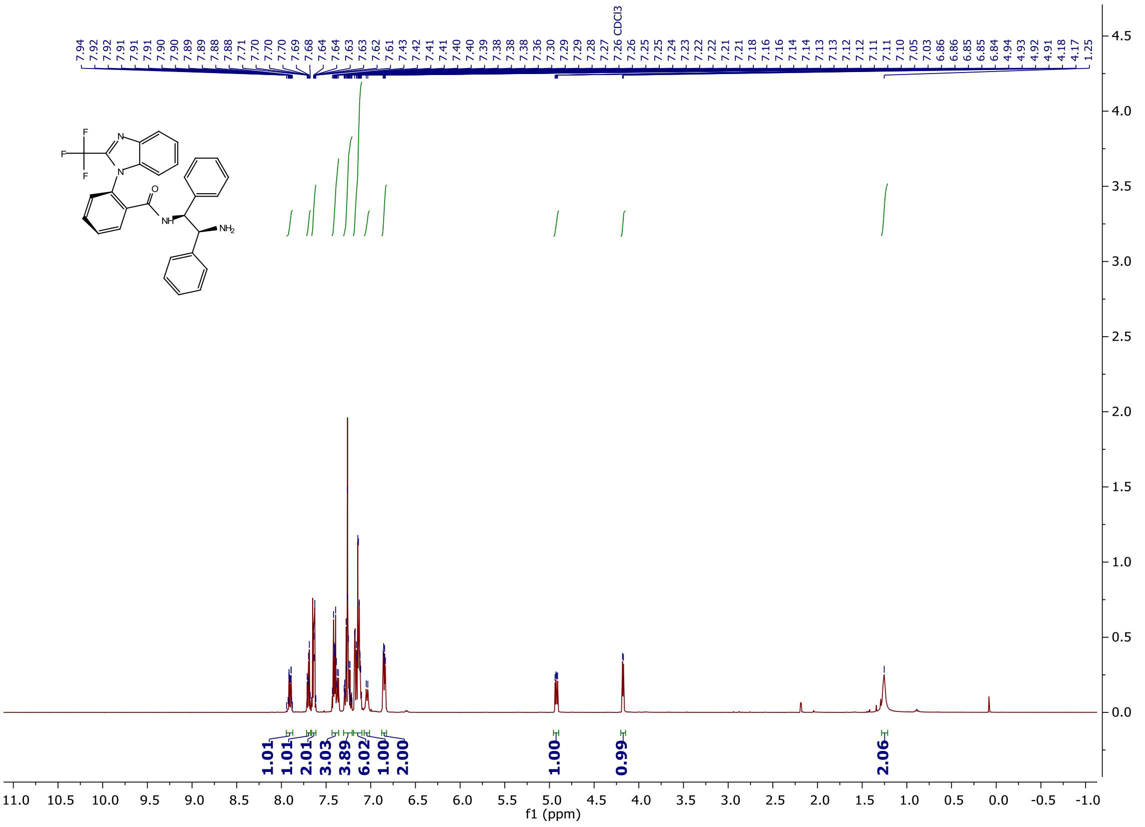

^{13}C{^{1}H} NMR (101 MHz, $CDCl_3$)

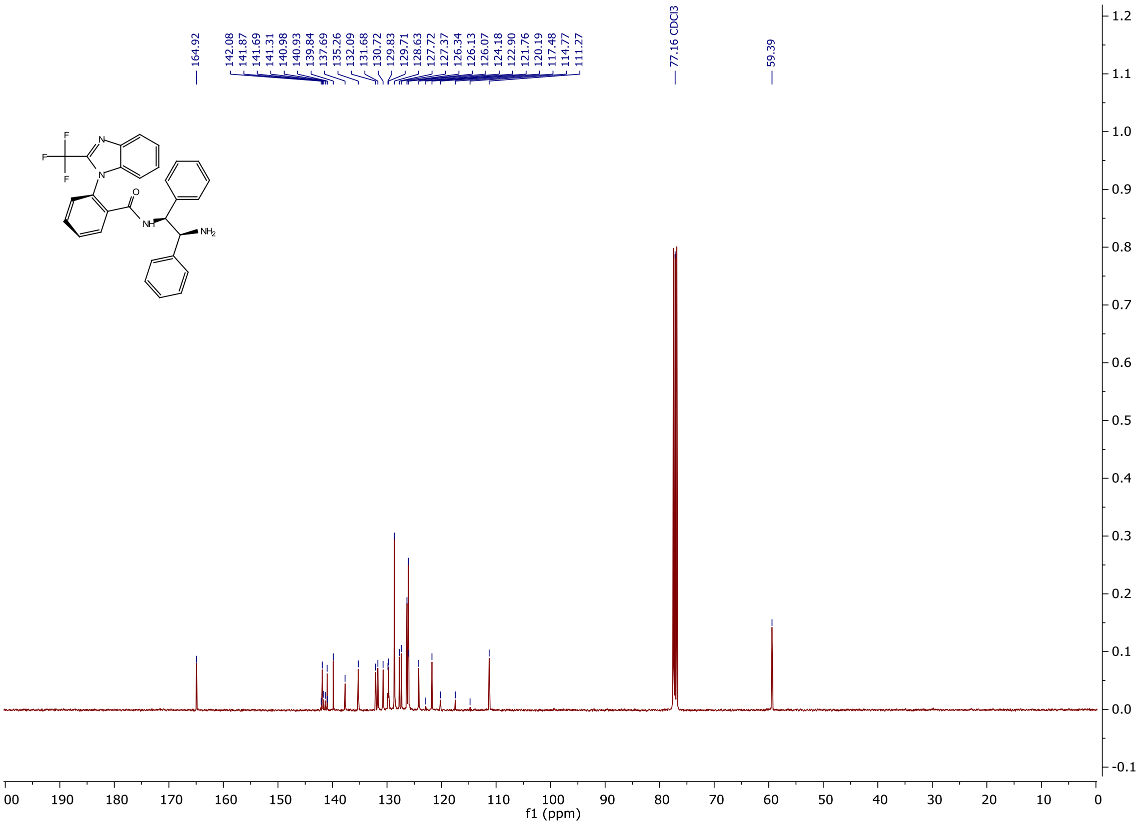

N-((1S,2*S*)-2-Amino-1,2-diphenylethyl)-2-((*M*)-2-(trifluoromethyl)-1*H*-benzo[*d*]imidazol-1-yl)benzamide **(*M*)-48**

^{1}H NMR (400 MHz, $CDCl_3$)

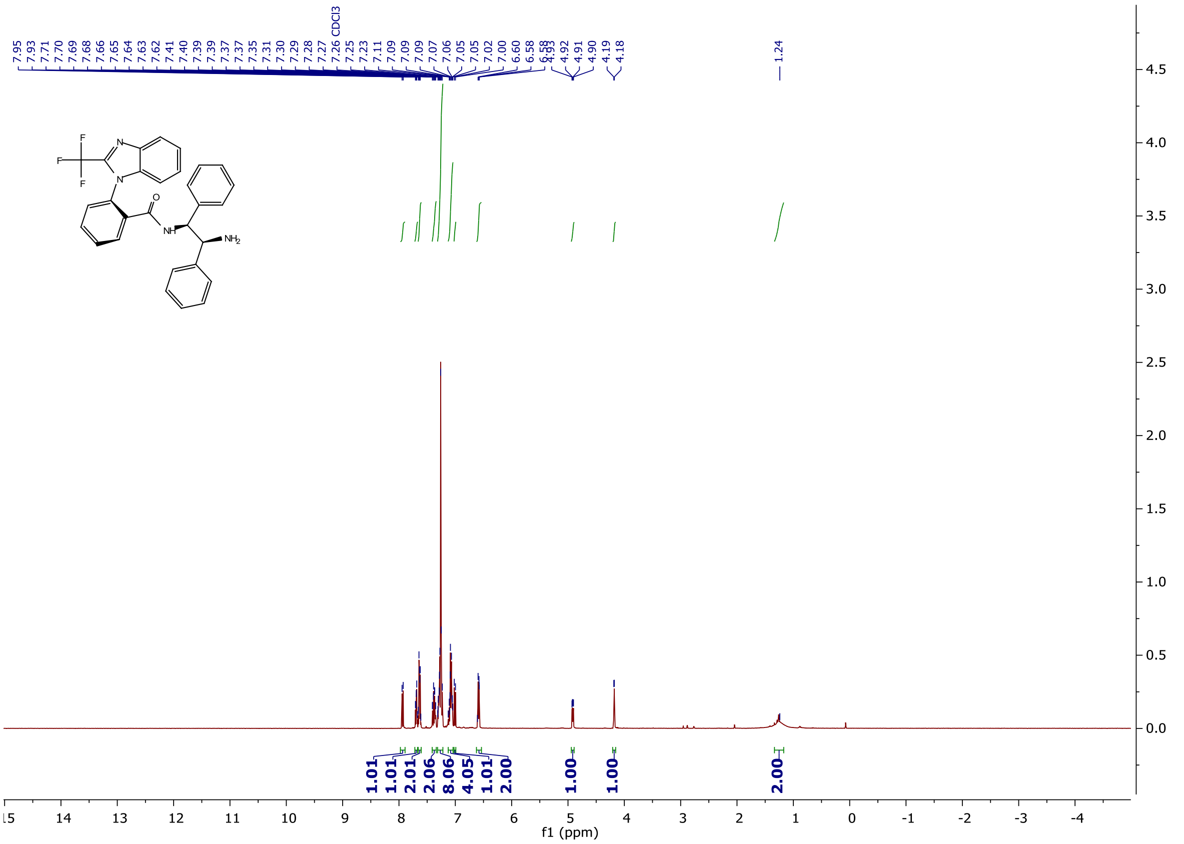

^{13}C{^{1}H} NMR (101 MHz, $CDCl_3$)

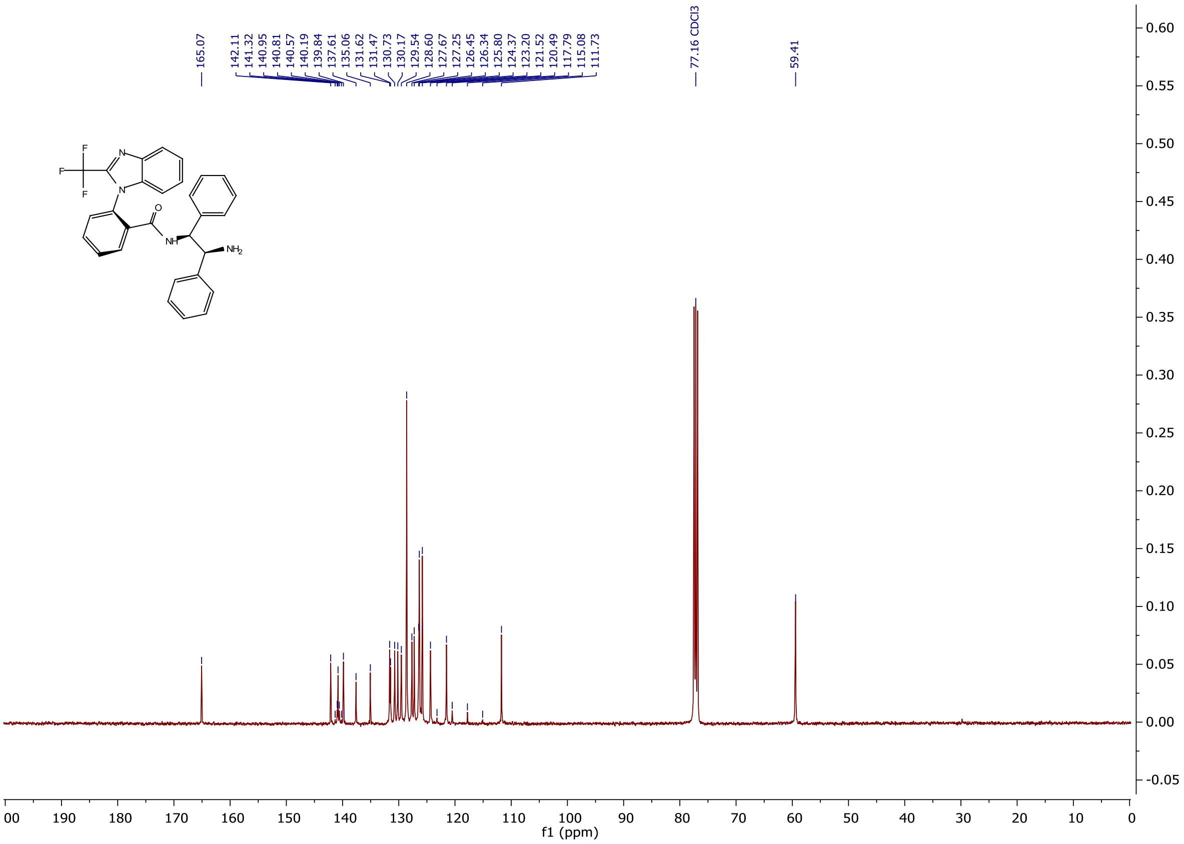

N-((1*S*,2*S*)-2-(Dimethylamino)-1,2-diphenylethyl)-2-((*P*)-2-(trifluoromethyl)-1*H*-benzo[*d*]imidazol-1-yl)benzamide **(*P*)-49**

^{1}H NMR (400 MHz, $CDCl_3$)

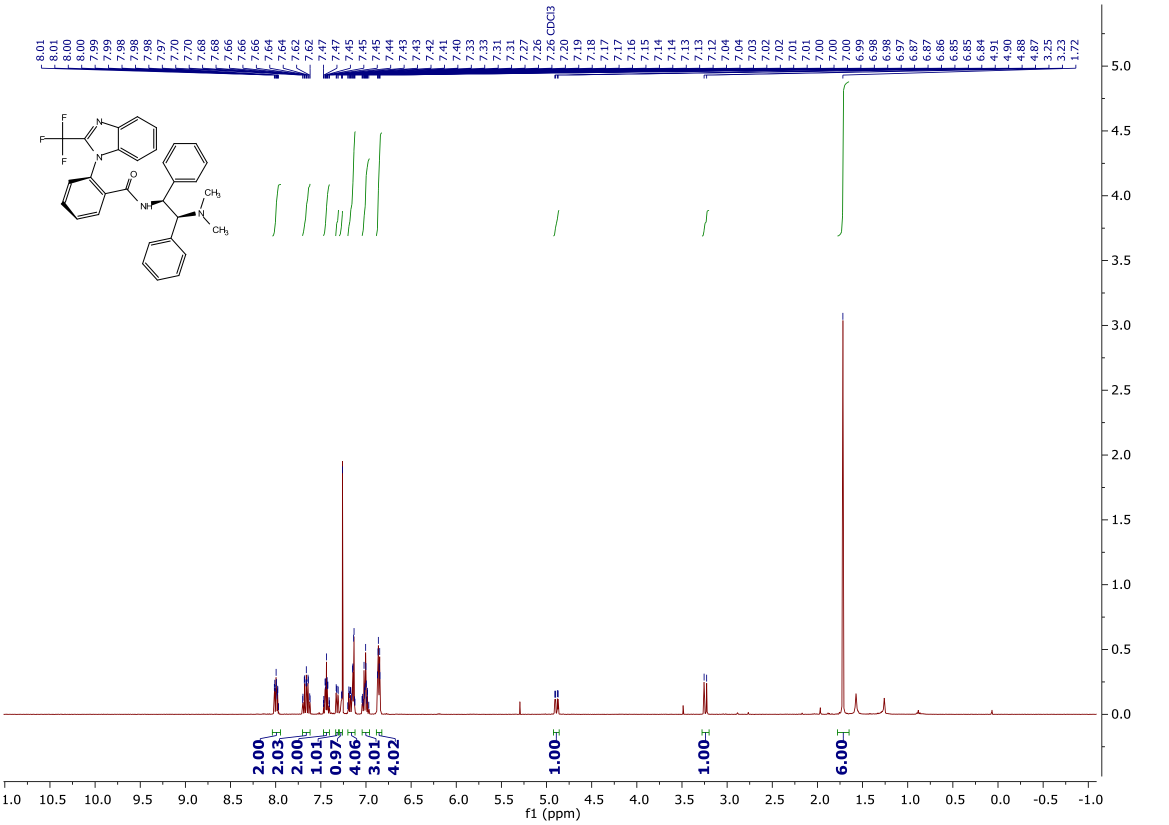

^{13}C{^{1}H} NMR (101 MHz, $CDCl_3$)

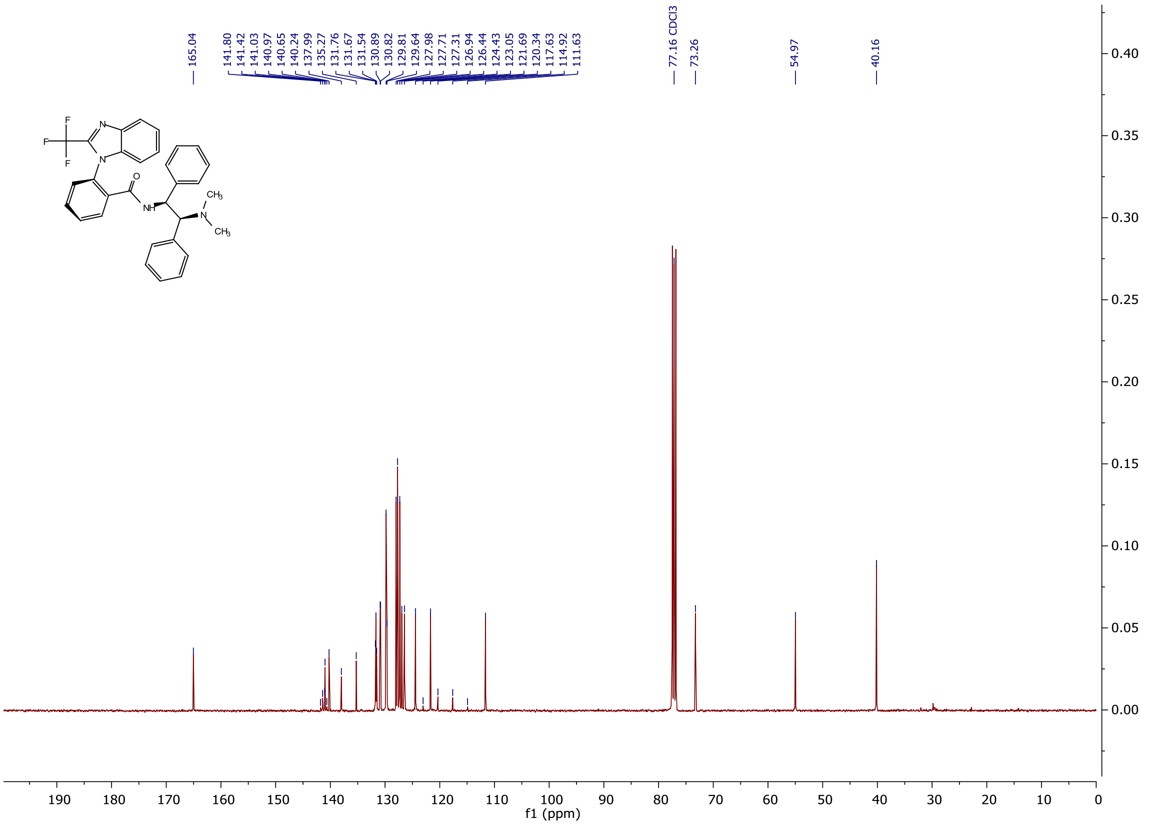

N-((1*S*,2*S*)-2-(Dimethylamino)-1,2-diphenylethyl)-2-((*M*)-2-(trifluoromethyl)-1*H*-benzo[*d*]imidazol-1-yl)benzamide **(*M*)-49**

^{1}H NMR (400 MHz, $CDCl_3$)

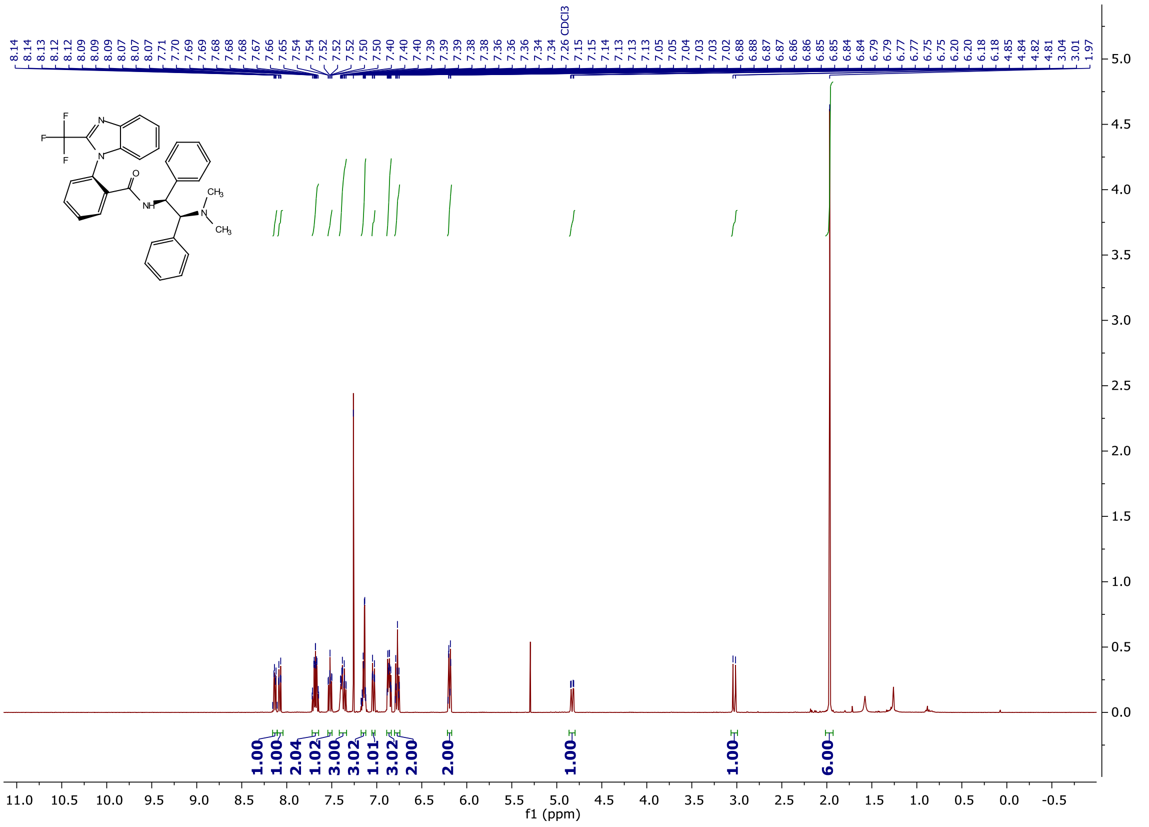

^{13}C{^{1}H} NMR (101 MHz, $CDCl_3$)

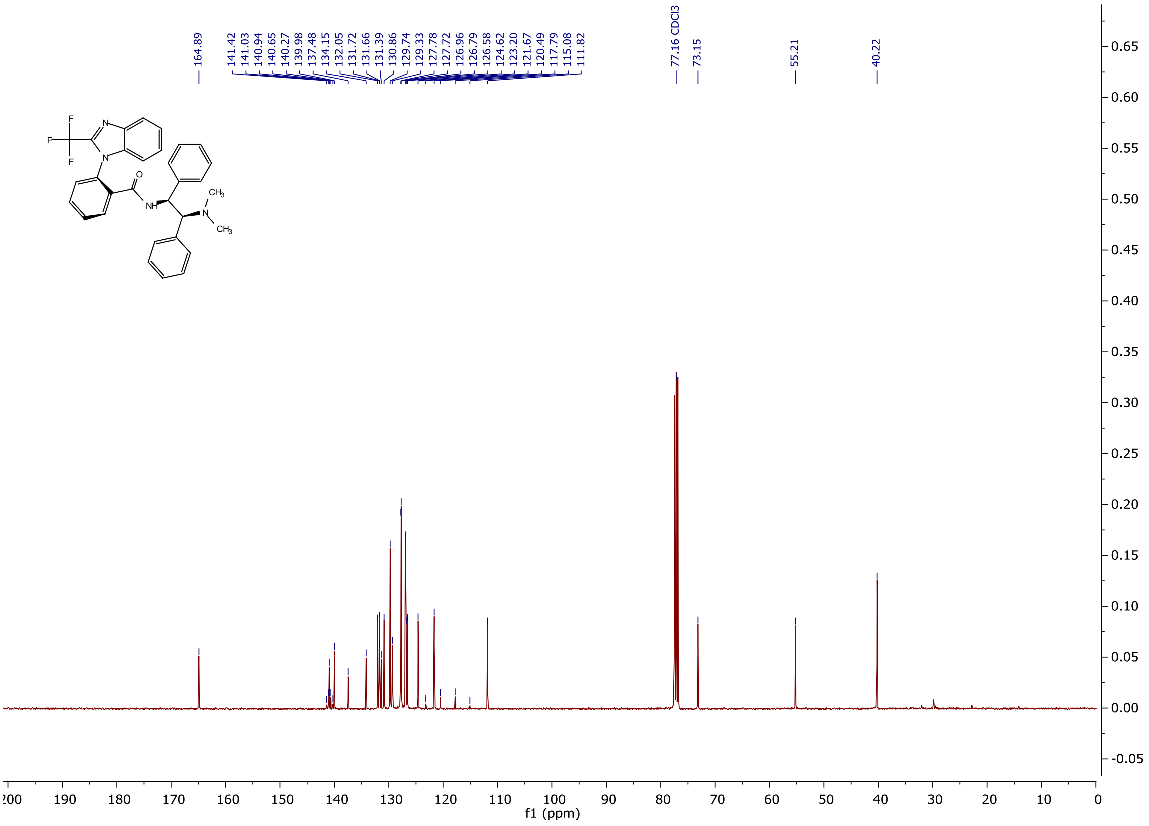

N-((1*S*,2*S*)-2-Acetamido-1,2-diphenylethyl)-2-((*P*)-2-(trifluoromethyl)-1*H*-benzo[*d*]imidazol-1-yl)benzamide **(*P*)-50**

^{1}H NMR (400 MHz, $CDCl_3$)

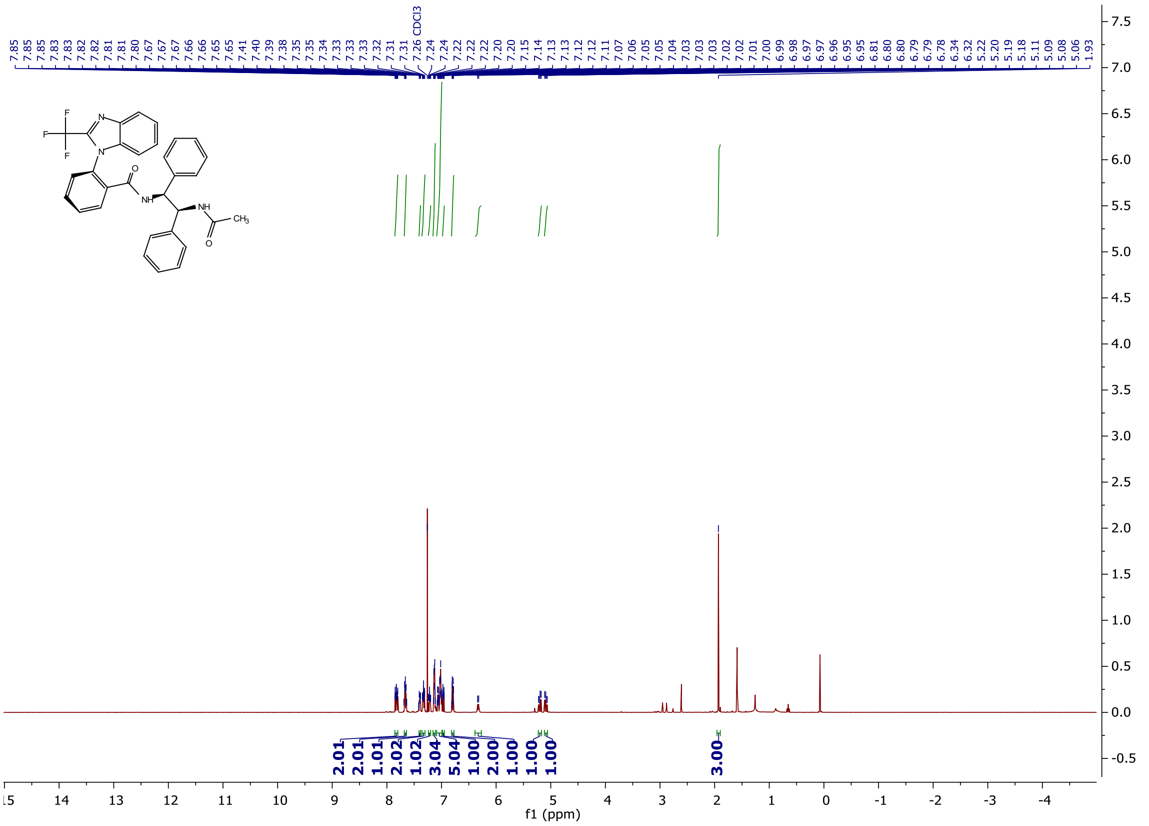

^{13}C{^{1}H} NMR (101 MHz, $CDCl_3$)

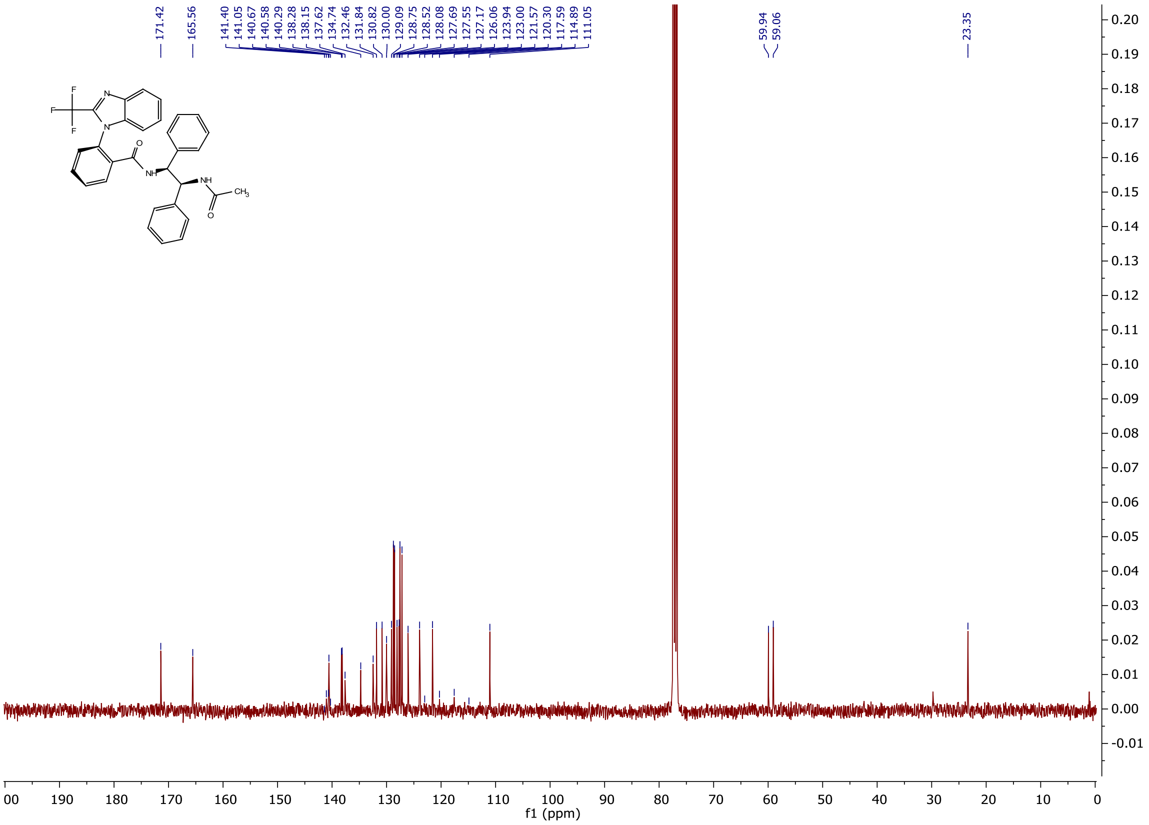

N-((1*S*,2*S*)-2-Acetamido-1,2-diphenylethyl)-2-((*M*)-2-(trifluoromethyl)-1*H*-benzo[*d*]imidazol-1-yl)benzamide **(*M*)-50**

^{1}H NMR (400 MHz, CDCl$_3$)

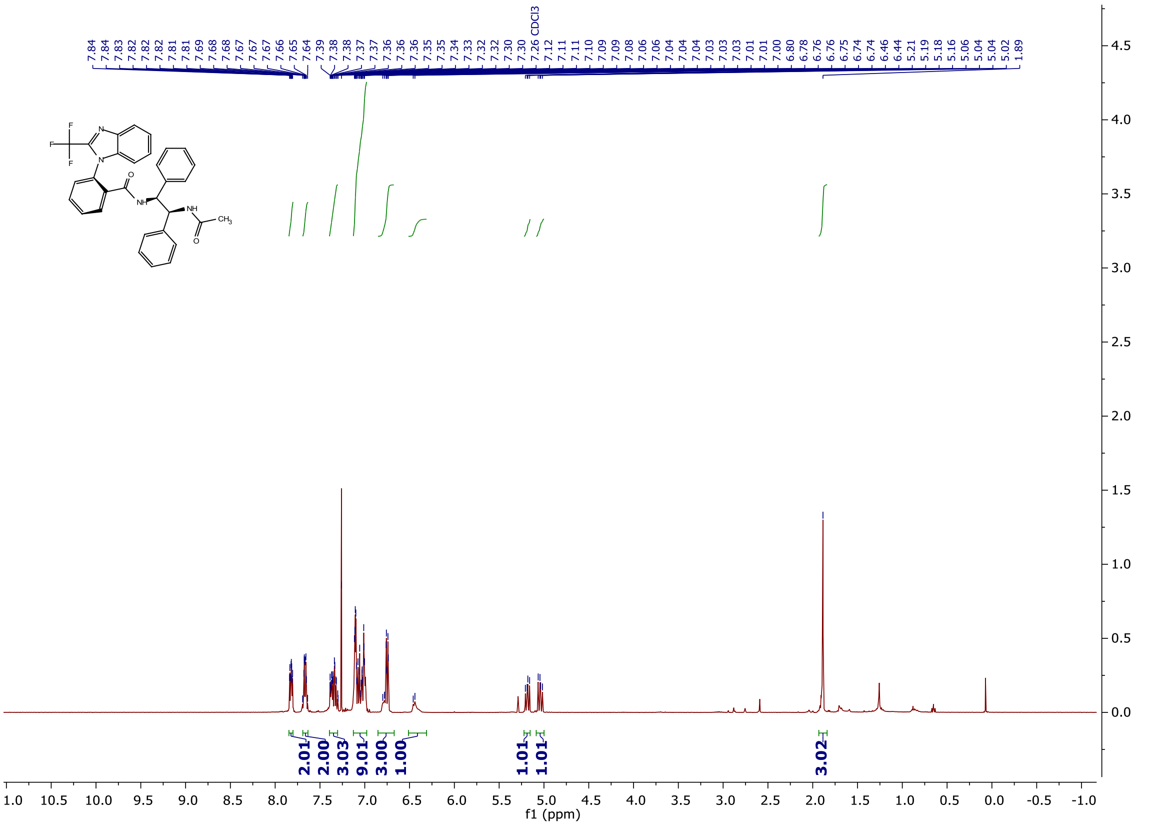

^{13}C{^{1}H} NMR (101 MHz, CDCl$_3$)

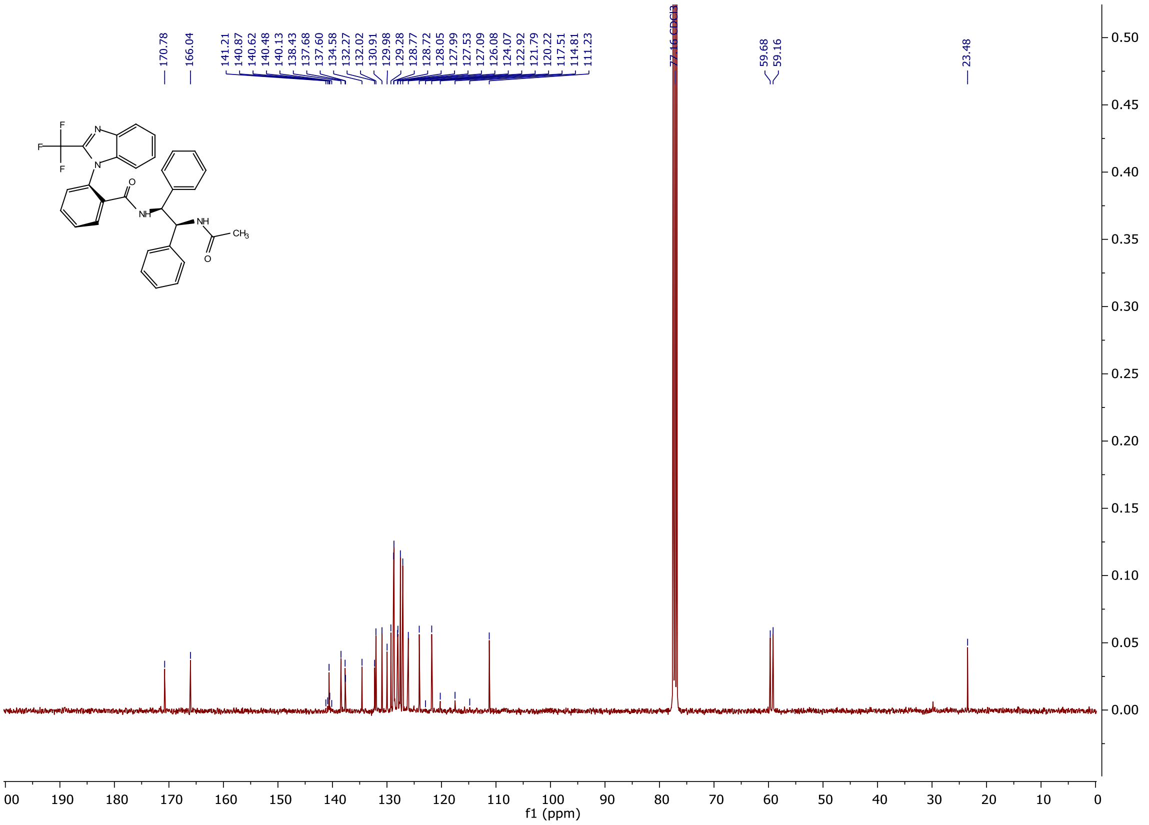

tert-Butyl ((1*S*,2*S*)-1,2-diphenyl-2-(2-((*P*)-2-(trifluoromethyl)-1*H*-benzo[*d*]imidazol-1-yl)benzamido)ethyl)carbamate **(*P*)-51**

^{1}H NMR (400 MHz, $CDCl_3$)

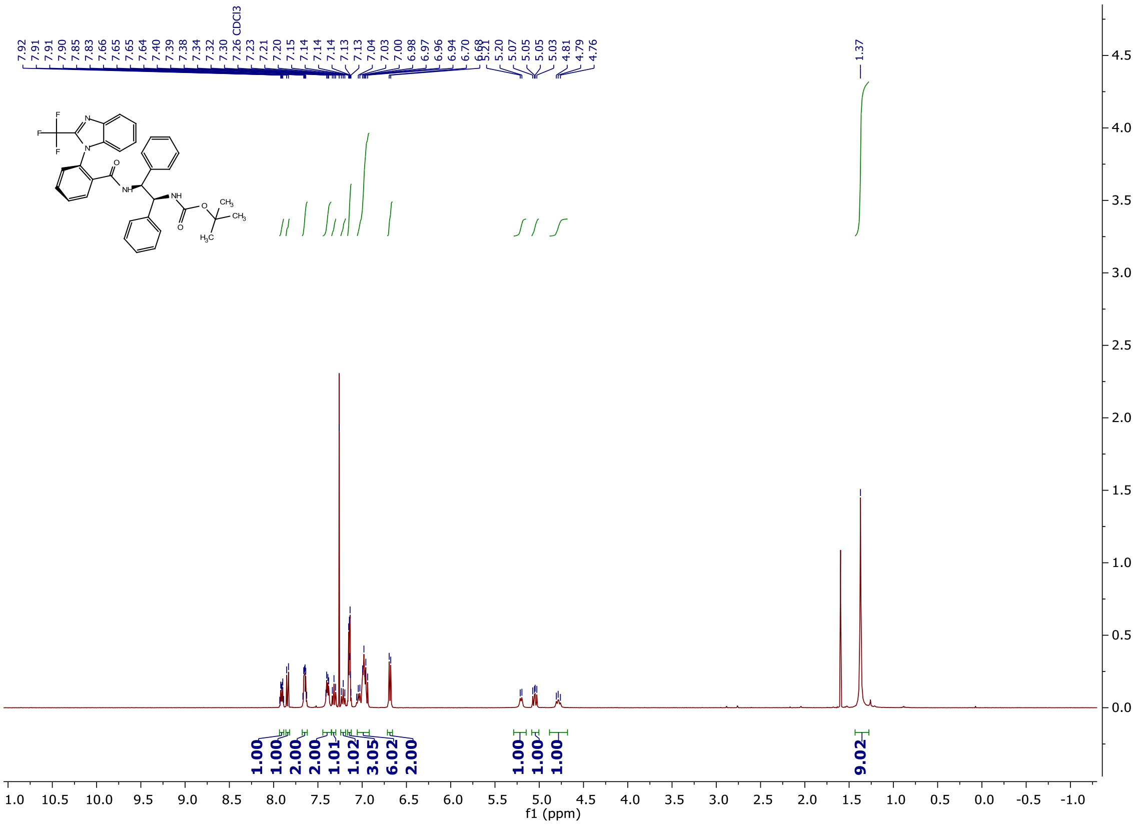

^{13}C{^{1}H} NMR (101 MHz, $CDCl_3$)

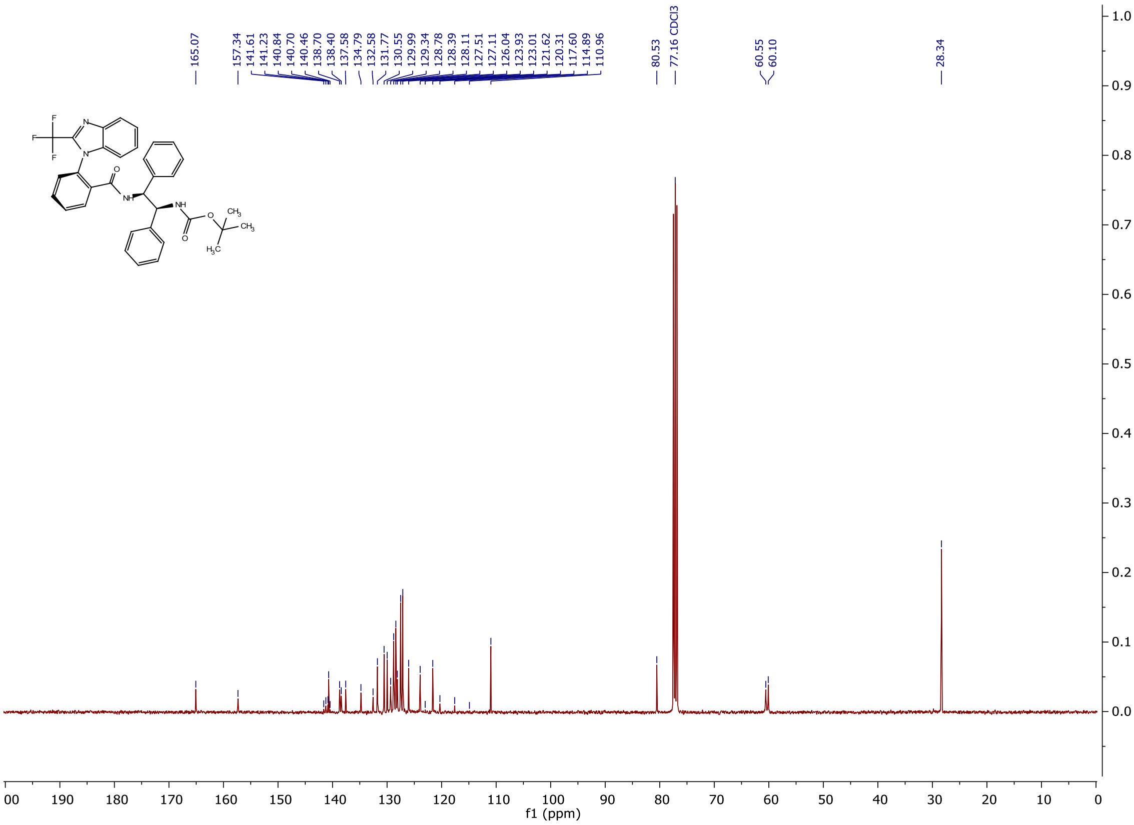

tert-Butyl ((1*S*,2*S*)-1,2-diphenyl-2-(2-((*M*)-2-(trifluoromethyl)-1*H*-benzo[*d*]imidazol-1-yl)benzamido)ethyl)carbamate **(*M*)-51**

^{1}H NMR (400 MHz, $CDCl_3$)

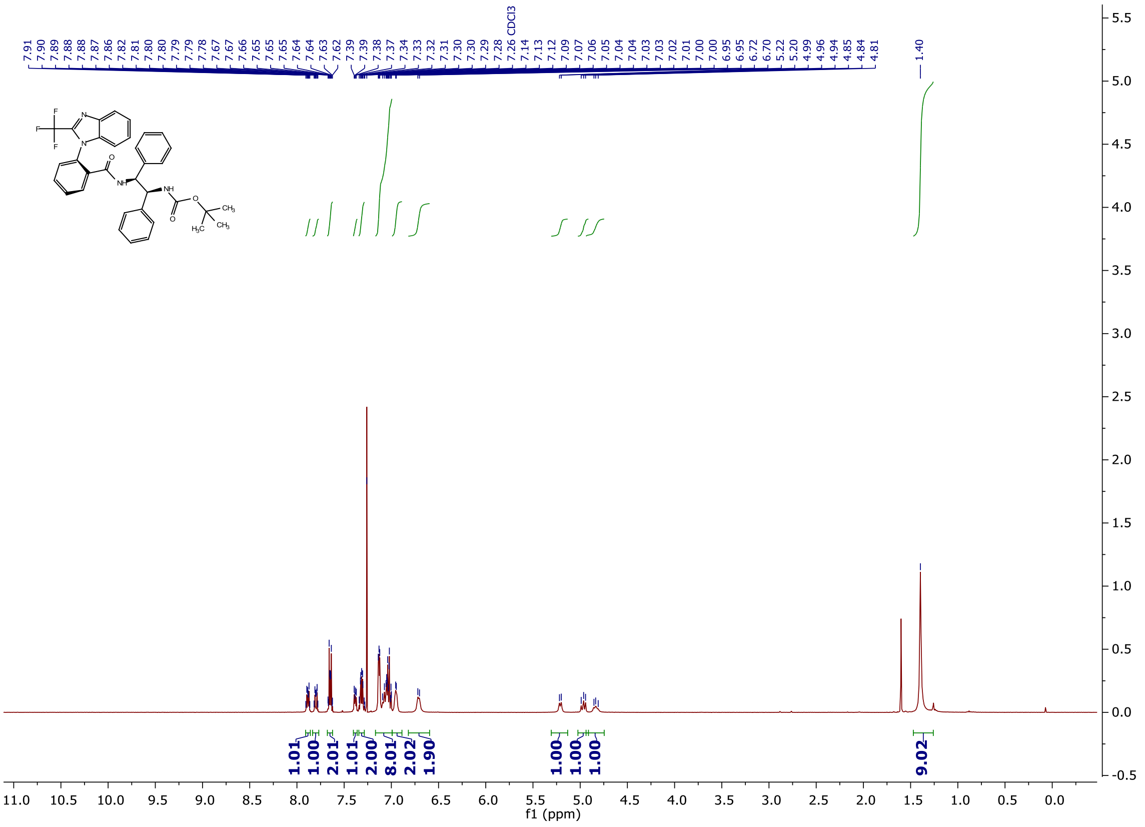

^{13}C{^{1}H} NMR (101 MHz, $CDCl_3$)

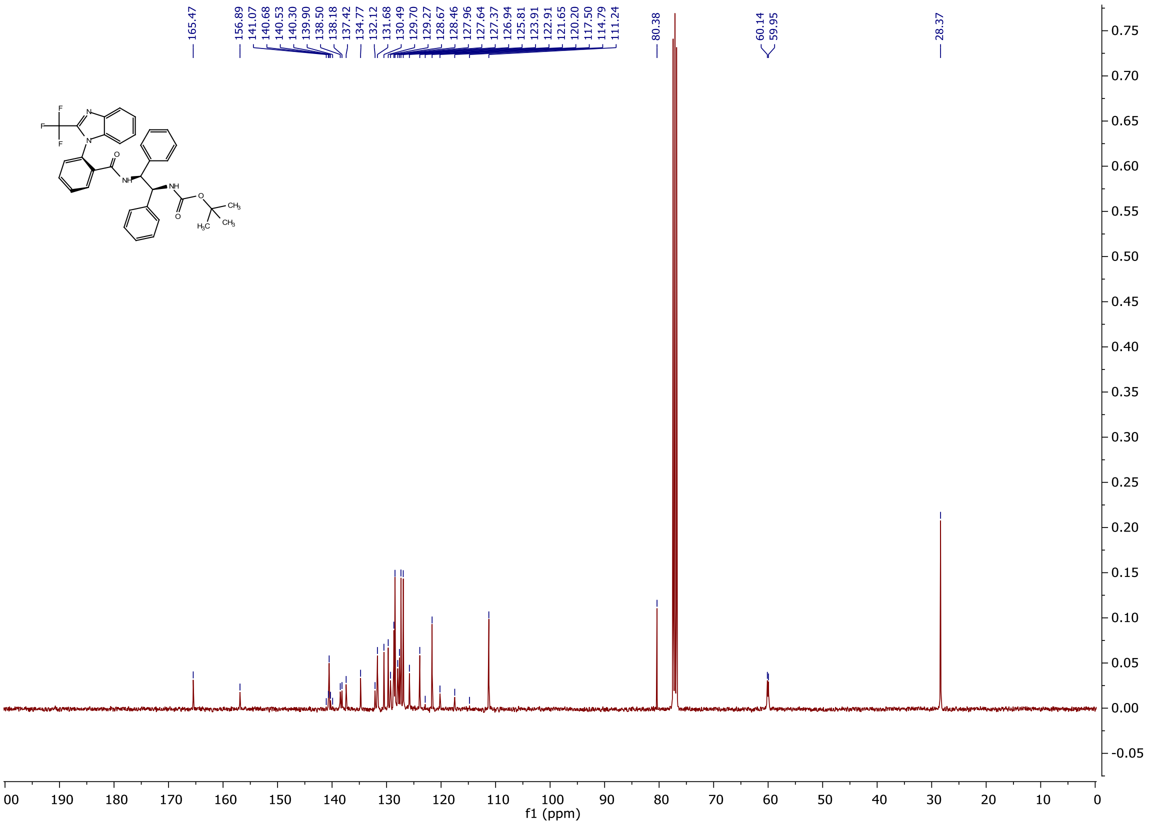

TBBA Derivatives: Copies of ^{19}F NMR Spectra

(*S*)-1-Methoxy-1-oxopropan-2-yl 2-((*P*)-2-(trifluoromethyl)-1*H*-benzo[*d*]imidazol-1-yl)benzoate **(*P*)-6**

^{19}F NMR (376 MHz, $CDCl_3$)

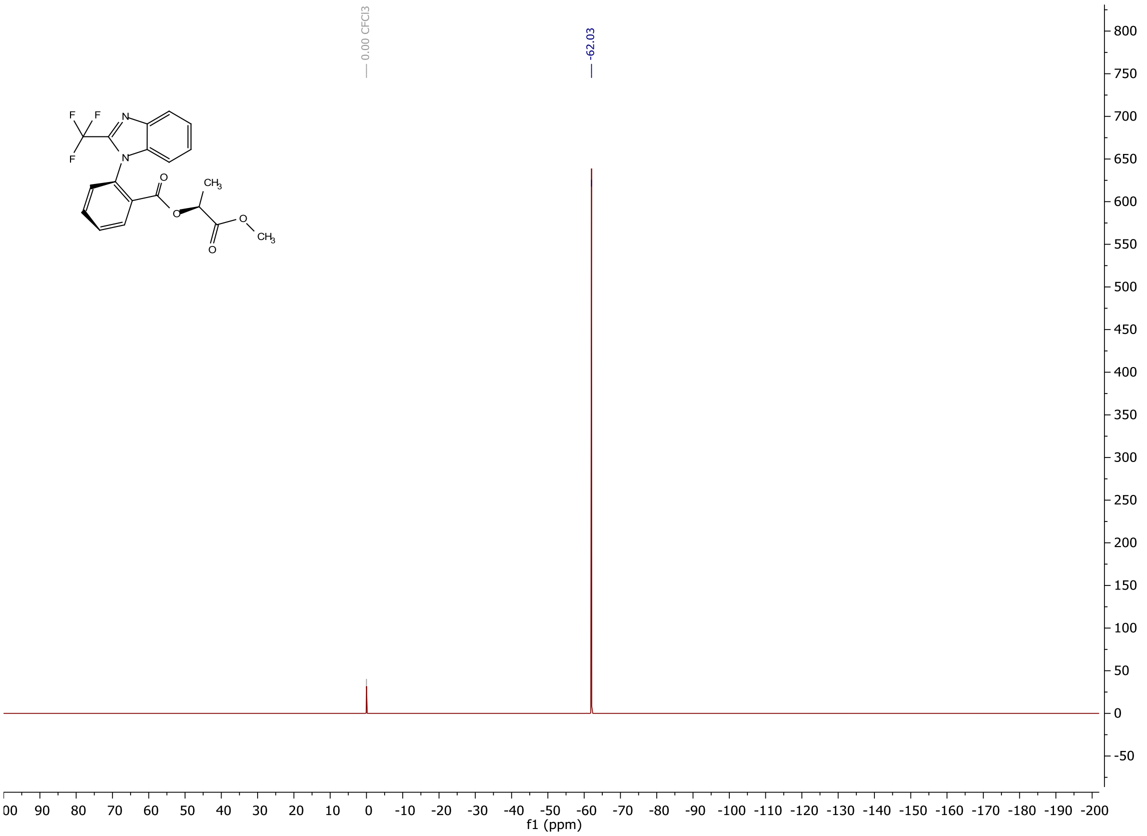

(*S*)-1-Methoxy-1-oxopropan-2-yl 2-((*M*)-2-(trifluoromethyl)-1*H*-benzo[*d*]imidazol-1-yl)benzoate **(*M*)-6**

^{19}F NMR (376 MHz, $CDCl_3$)

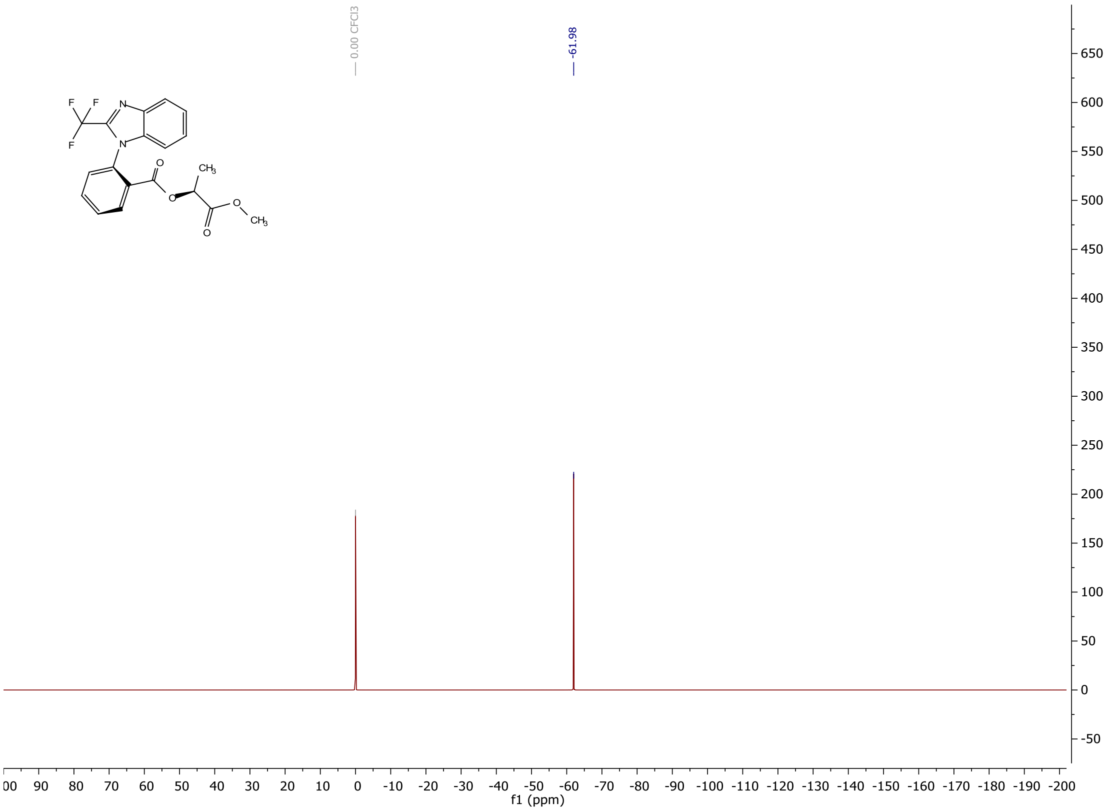

(*R*)-1-Phenylethyl 2-((*P*)-2-(trifluoromethyl)-1*H*-benzo[*d*]imidazol-1-yl)benzoate **(*P*)-7**

^{19}F NMR (376 MHz, $CDCl_3$)

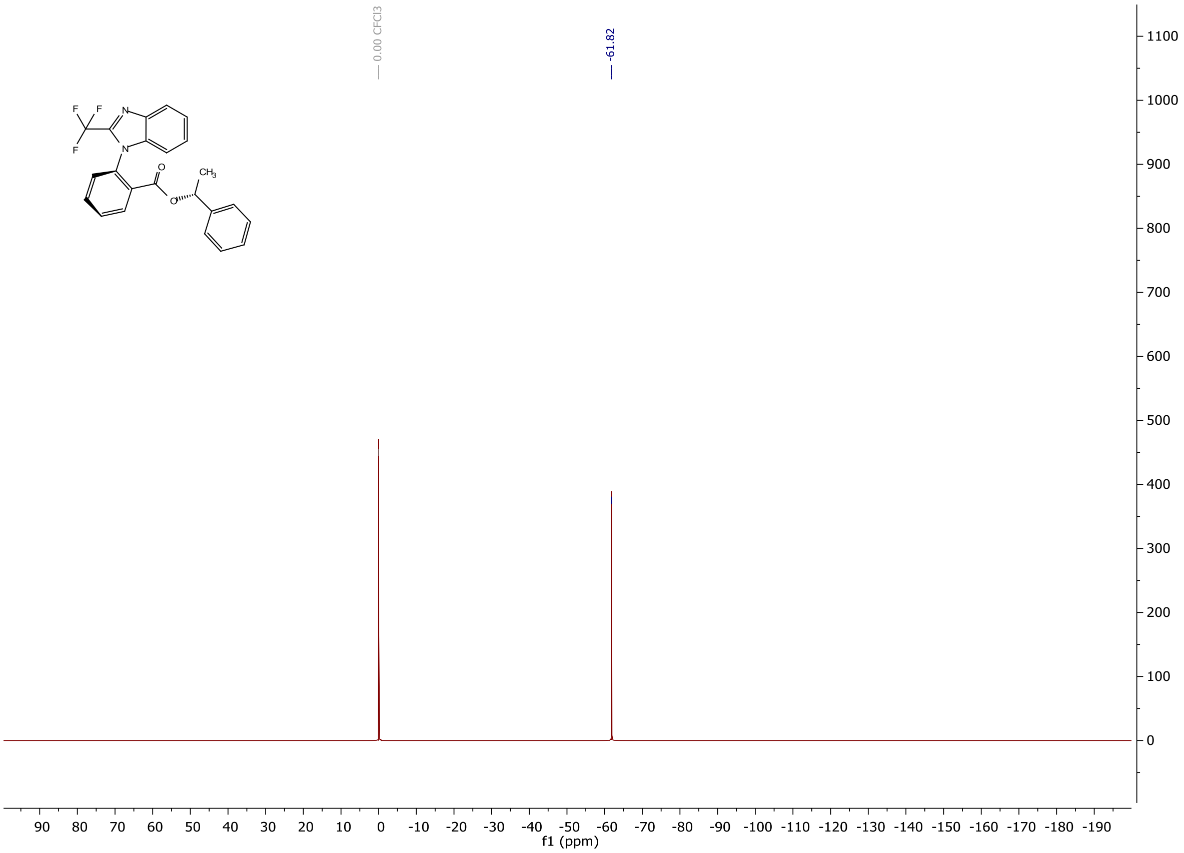

(*R*)-1-Phenylethyl 2-((*M*)-2-(trifluoromethyl)-1*H*-benzo[*d*]imidazol-1-yl)benzoate **(*M*)-7**

^{19}F NMR (376 MHz, $CDCl_3$)

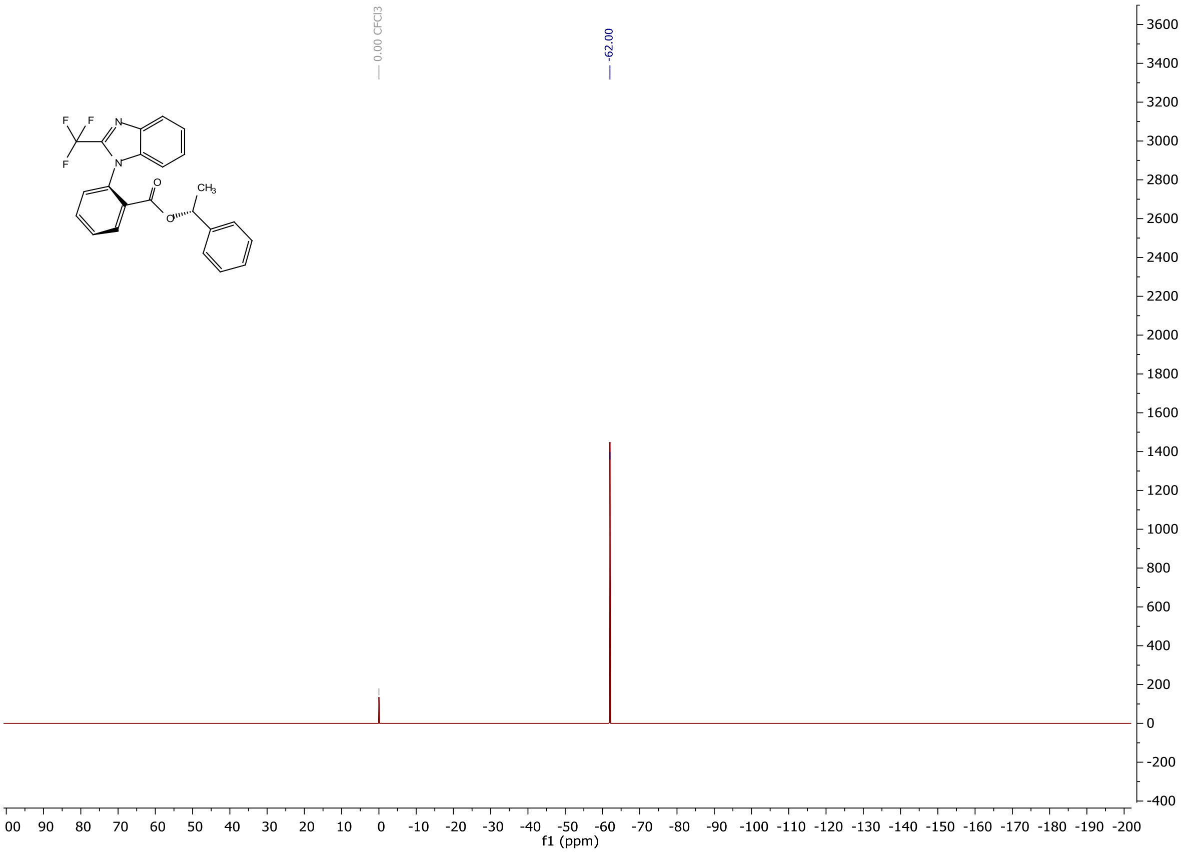

(*S*)-*sec*-Butyl 2-((*P*)-2-(trifluoromethyl)-1*H*-benzo[*d*]imidazol-1-yl)benzoate **(*P*)-8**

^{19}F NMR (376 MHz, $CDCl_3$)

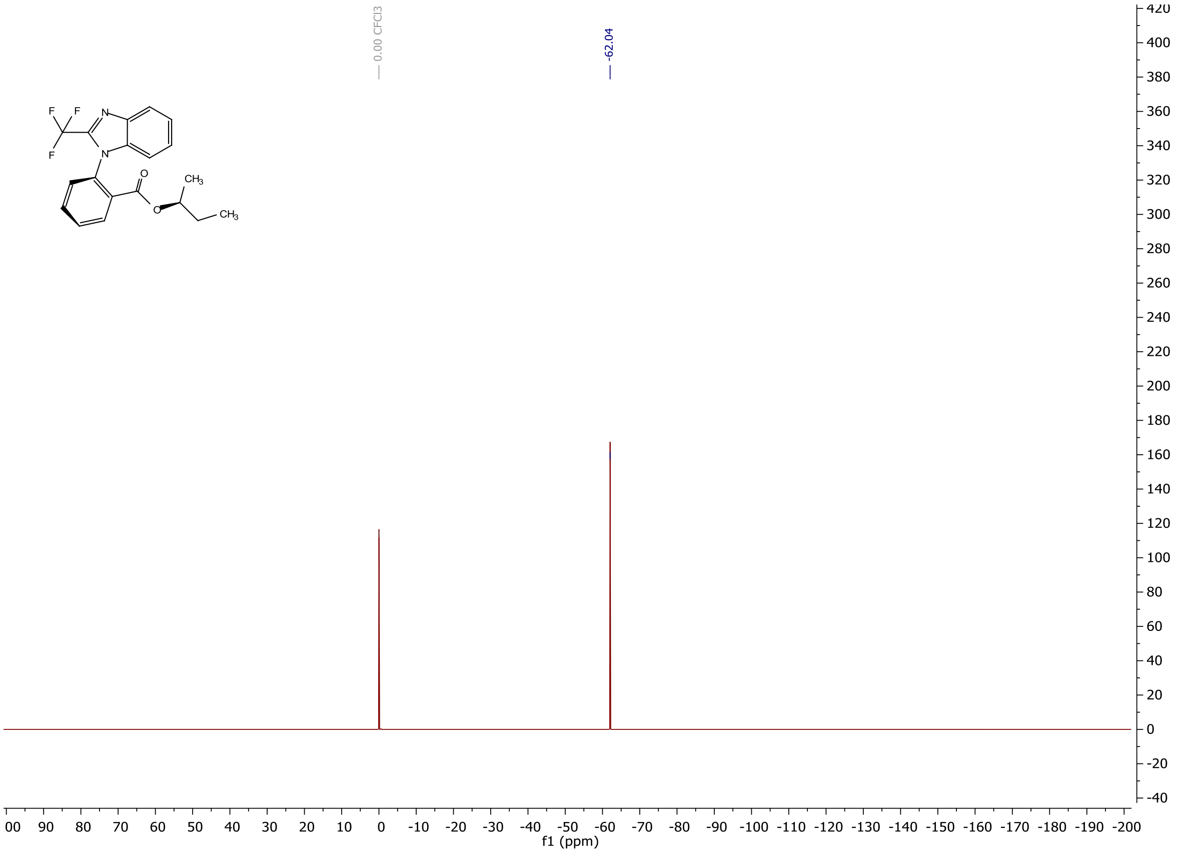

(*S*)-*sec*-Butyl 2-((*M*)-2-(trifluoromethyl)-1*H*-benzo[*d*]imidazol-1-yl)benzoate **(*M*)-8**

^{19}F NMR (376 MHz, $CDCl_3$)

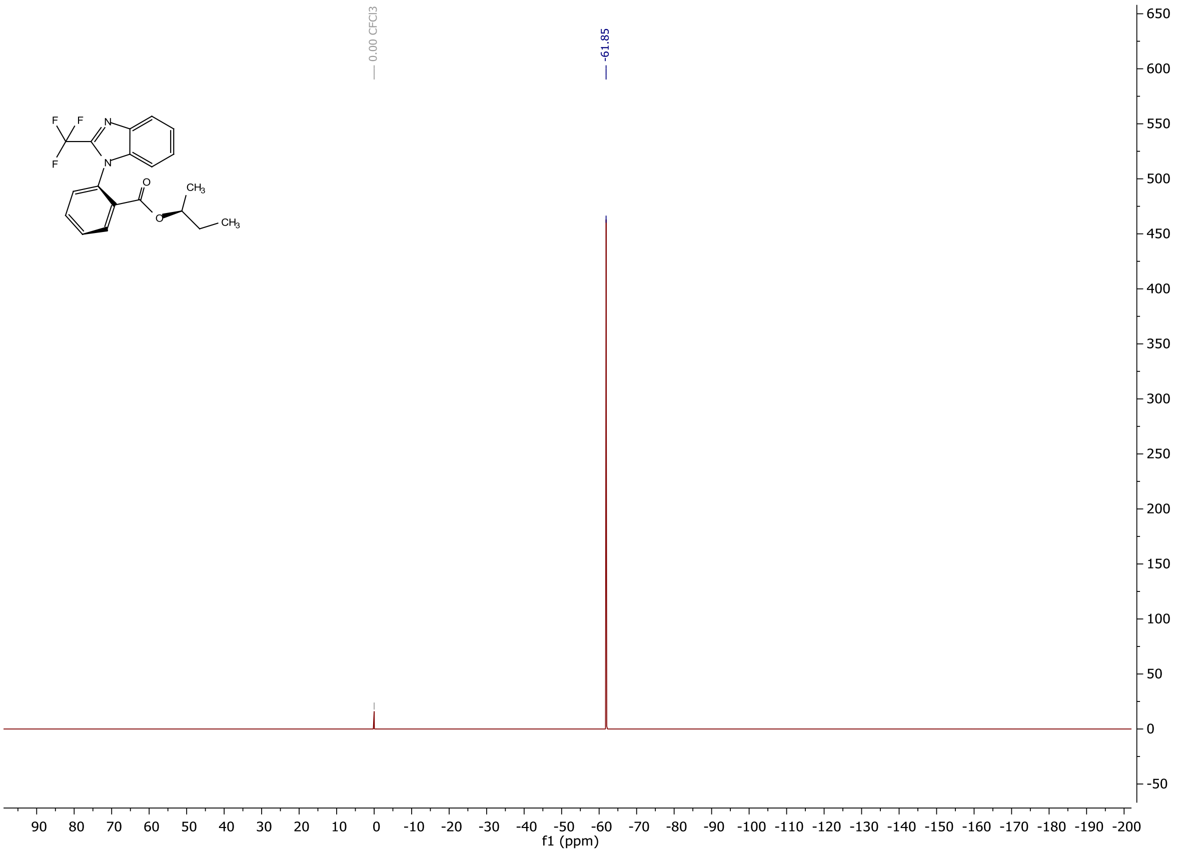

(*R*)-Heptan-2-yl 2-((*P*)-2-(trifluoromethyl)-1*H*-benzo[*d*]imidazol-1-yl)benzoate **(*P*)-9**

^{19}F NMR (376 MHz, $CDCl_3$)

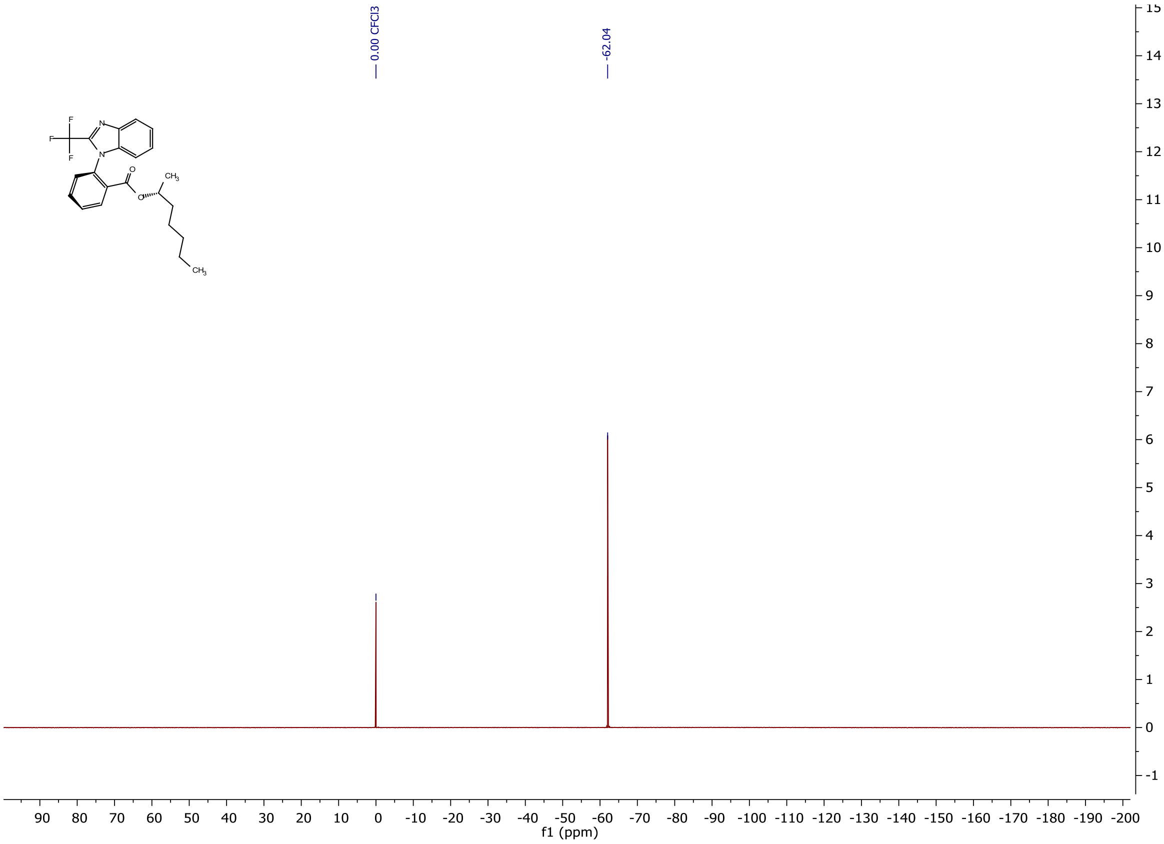

(*R*)-Heptan-2-yl 2-((*M*)-2-(trifluoromethyl)-1*H*-benzo[*d*]imidazol-1-yl)benzoate **(*M*)-9**

^{19}F NMR (376 MHz, $CDCl_3$)

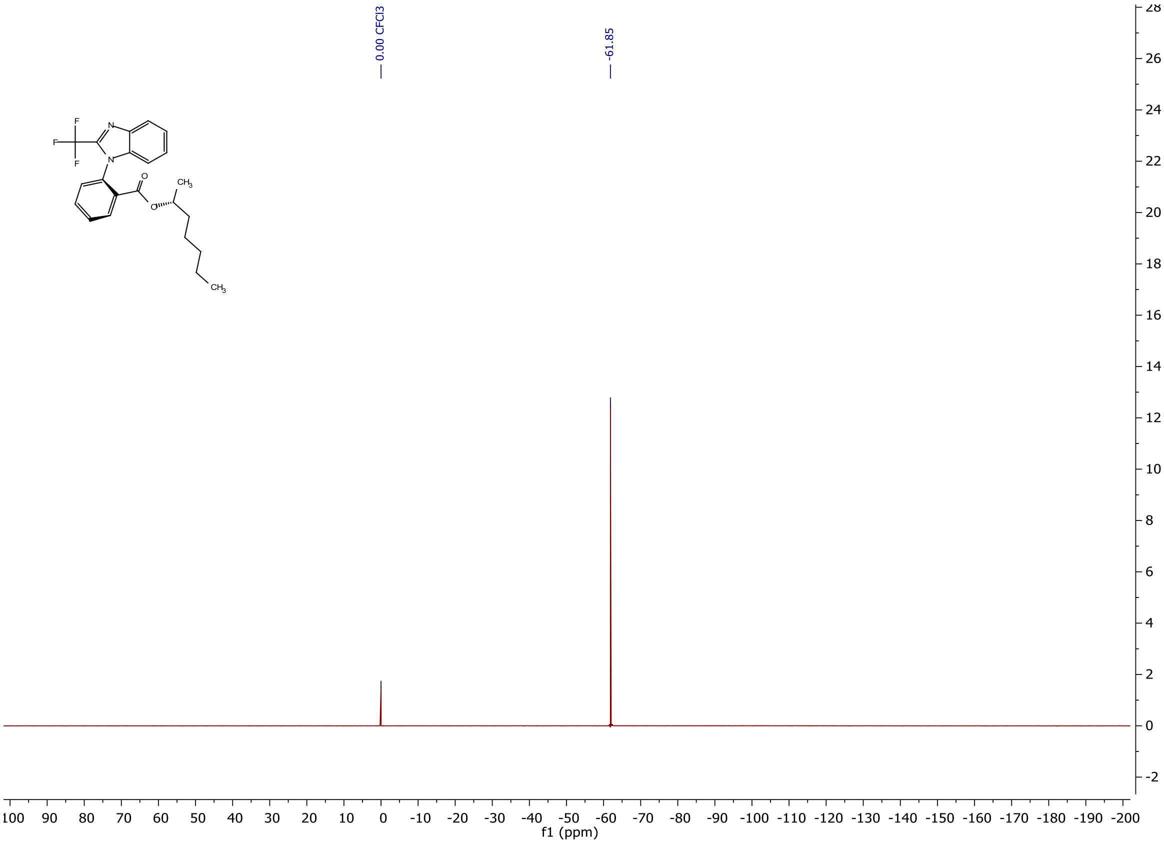

(*S*)-But-3-yn-2-yl 2-((*P*)-2-(trifluoromethyl)-1*H*-benzo[*d*]imidazol-1-yl)benzoate **(*P*)-10**

^{19}F NMR (376 MHz, $CDCl_3$)

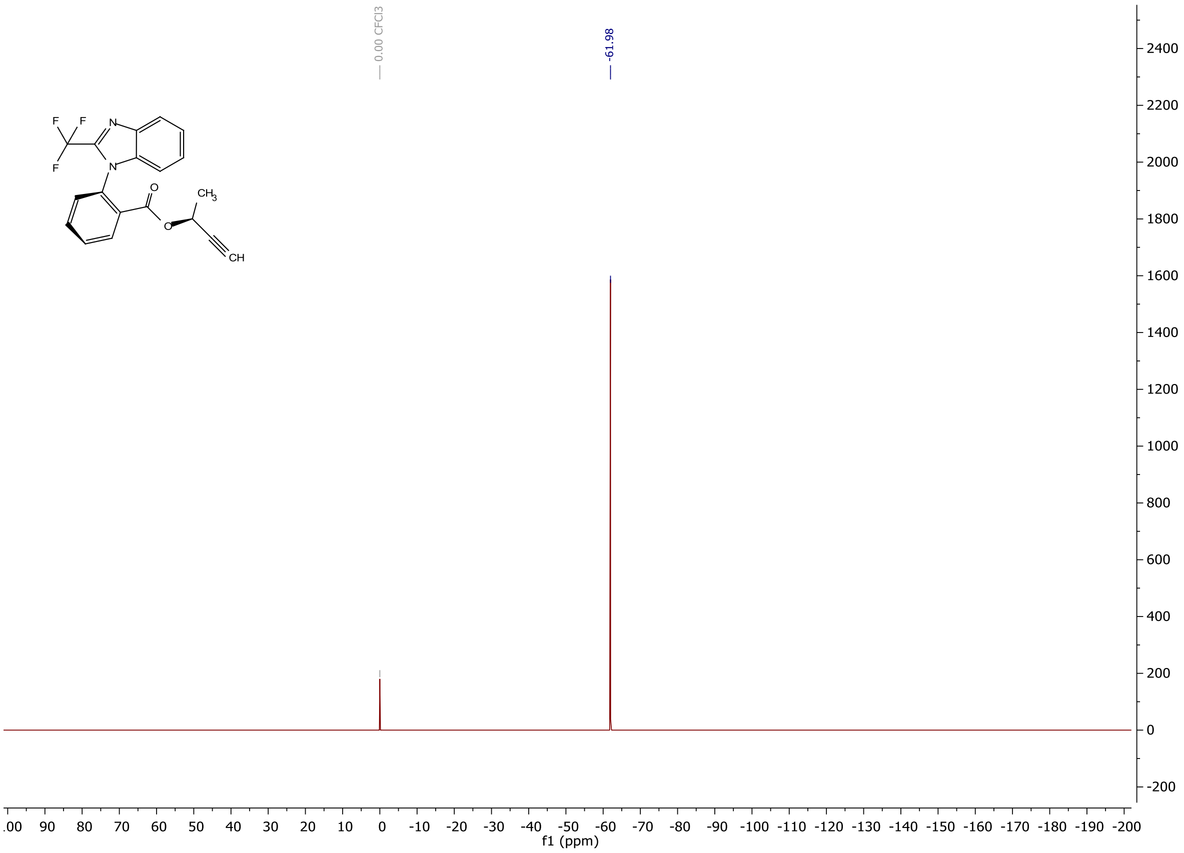

(*S*)-But-3-yn-2-yl 2-((*M*)-2-(trifluoromethyl)-1*H*-benzo[*d*]imidazol-1-yl)benzoate **(*M*)-10**

^{19}F NMR (376 MHz, $CDCl_3$)

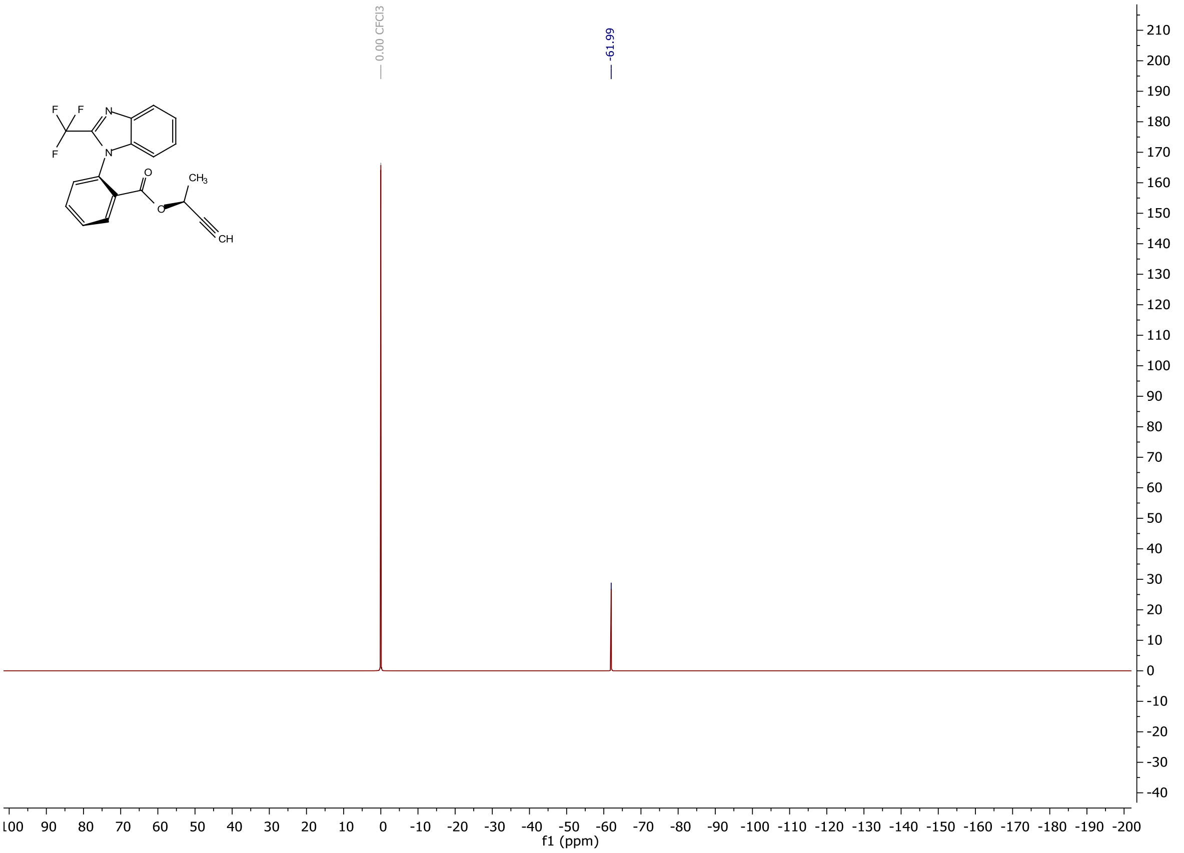

(*S*)-2-Methoxy-2-oxo-1-phenylethyl 2-((*P*)-2-(trifluoromethyl)-1*H*-benzo[*d*]imidazol-1-yl)benzoate **(*P*)-11**

^{19}F NMR (376 MHz, $CDCl_3$)

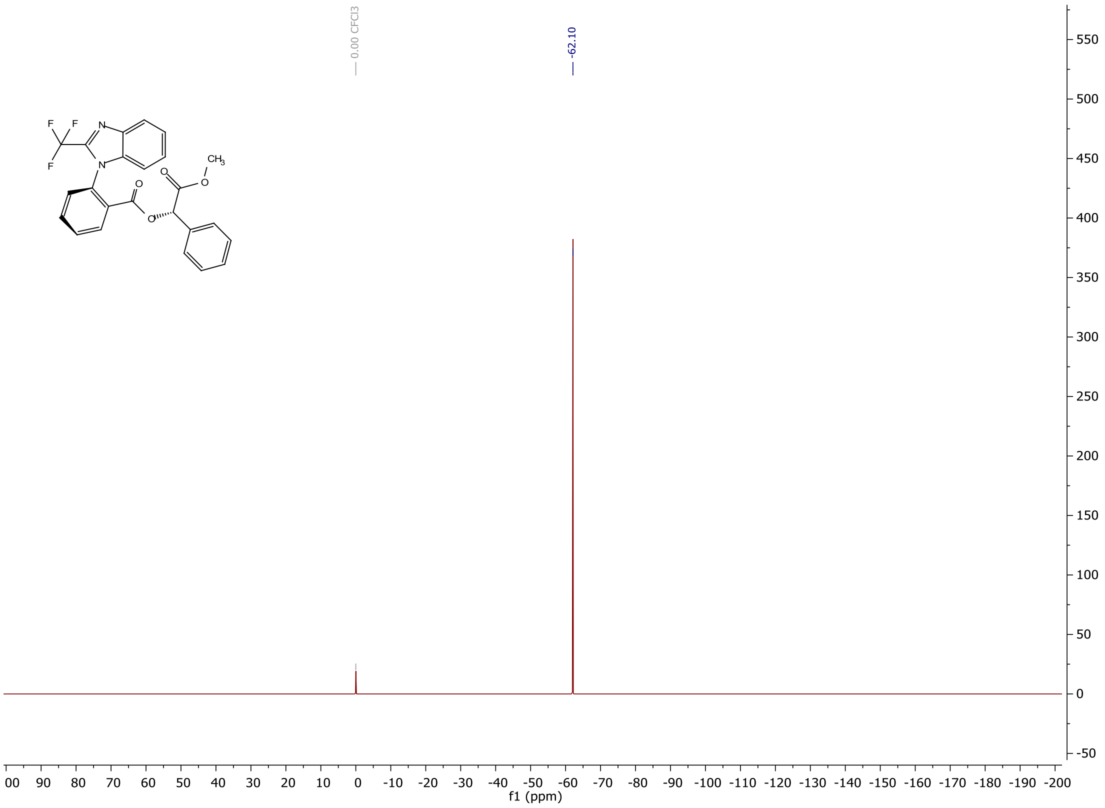

(*S*)-2-Methoxy-2-oxo-1-phenylethyl 2-((*M*)-2-(trifluoromethyl)-1*H*-benzo[*d*]imidazol-1-yl)benzoate **(*M*)-11**

^{19}F NMR (376 MHz, $CDCl_3$)

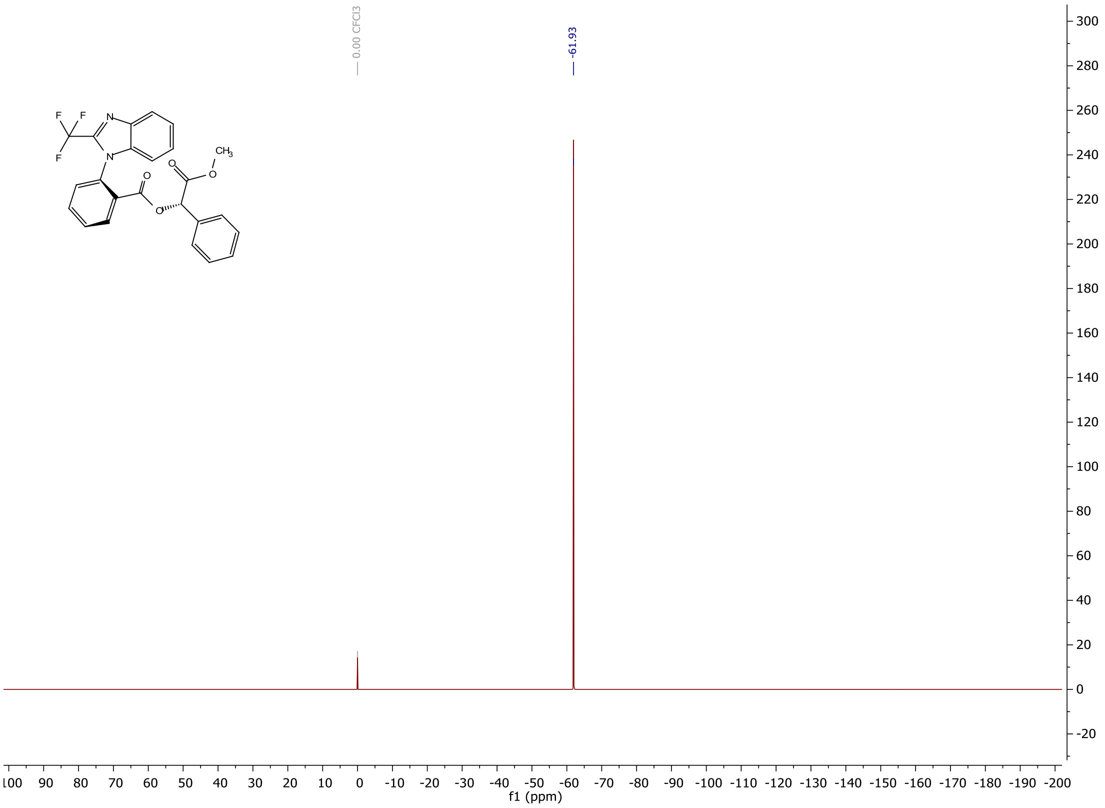

(1*R*,2*S*,5*R*)-2-Isopropyl-5-methylcyclohexyl 2-((*P*)-2-(trifluoromethyl)-1*H*-benzo[*d*]imidazol-1-yl)benzoate **(*P*)-12**

^{19}F NMR (376 MHz, $CDCl_3$)

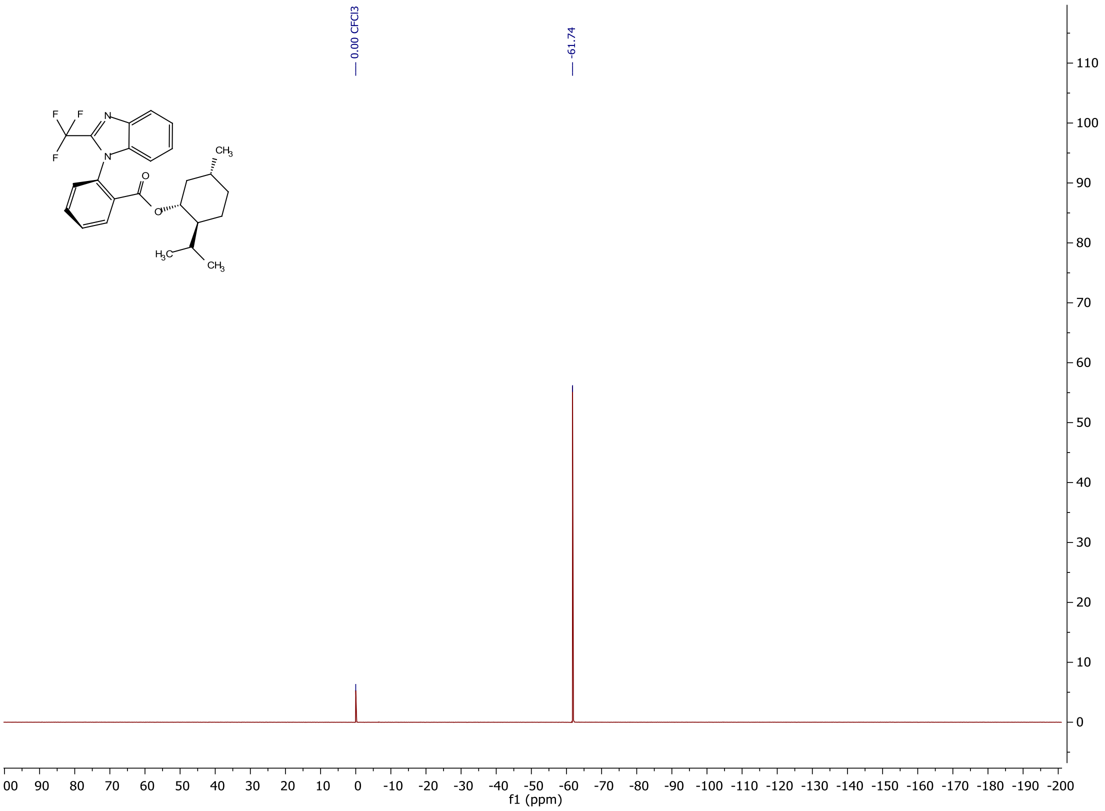

(1*R*,2*S*,5*R*)-2-Isopropyl-5-methylcyclohexyl 2-((*M*)-2-(trifluoromethyl)-1*H*-benzo[*d*]imidazol-1-yl)benzoate **(*M*)-12**

^{19}F NMR (376 MHz, $CDCl_3$)

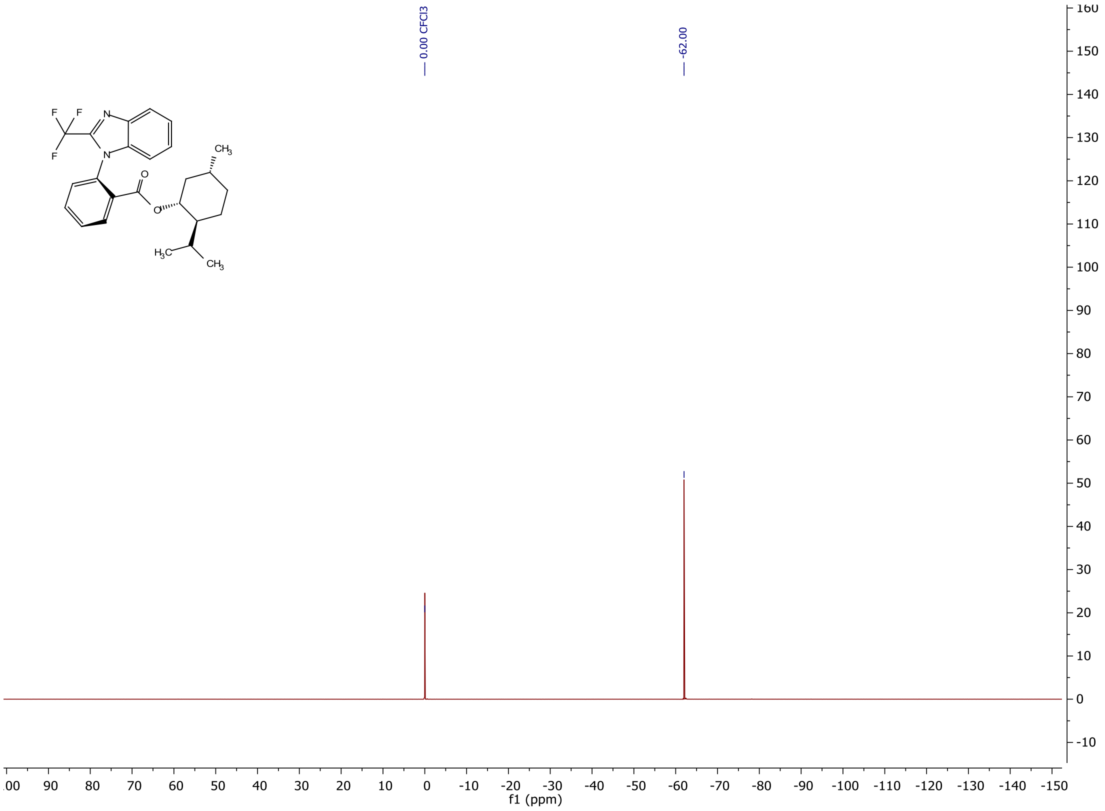

(1*S*,2*R*,4*S*)-1,7,7-Trimethylbicyclo[2.2.1]heptan-2-yl 2-((*P*)-2-(trifluoromethyl)-1*H*-benzo[*d*]imidazol-1-yl)benzoate **(*P*)-13**

^{19}F NMR (376 MHz, $CDCl_3$)

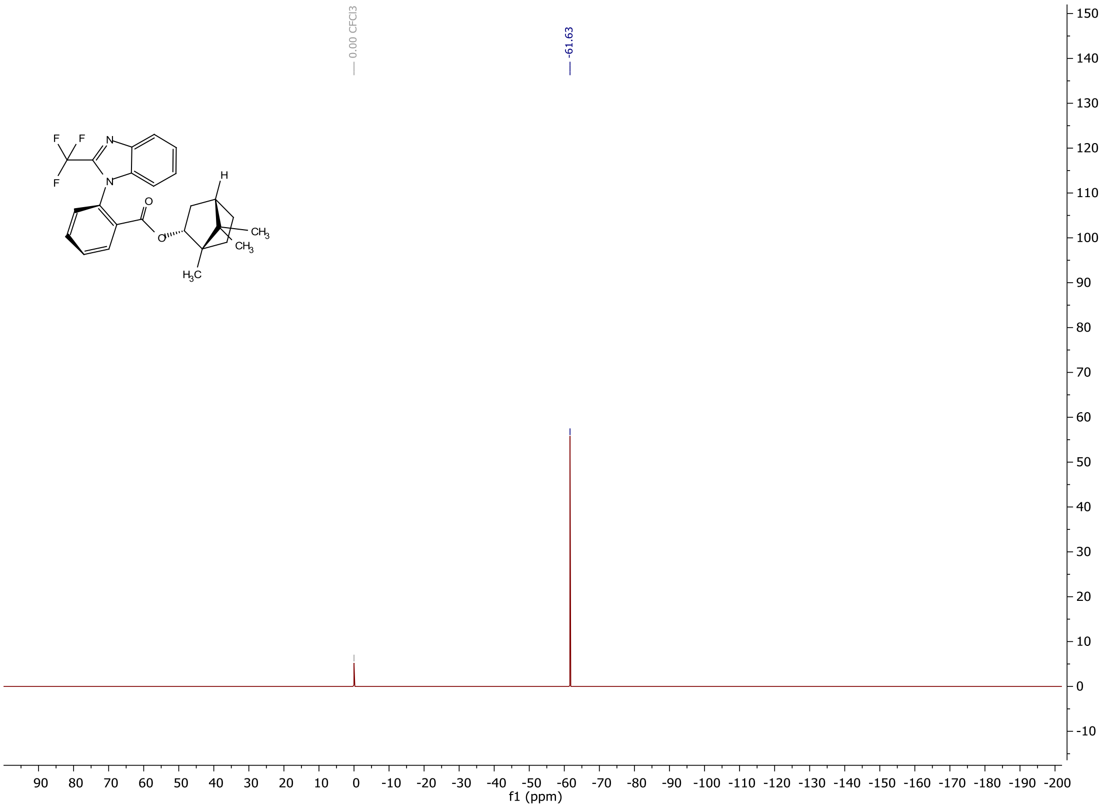

(1*S*,2*R*,4*S*)-1,7,7-Trimethylbicyclo[2.2.1]heptan-2-yl 2-((*M*)-2-(trifluoromethyl)-1*H*-benzo[*d*]imidazol-1-yl)benzoate **(*M*)-13**

^{19}F NMR (376 MHz, $CDCl_3$)

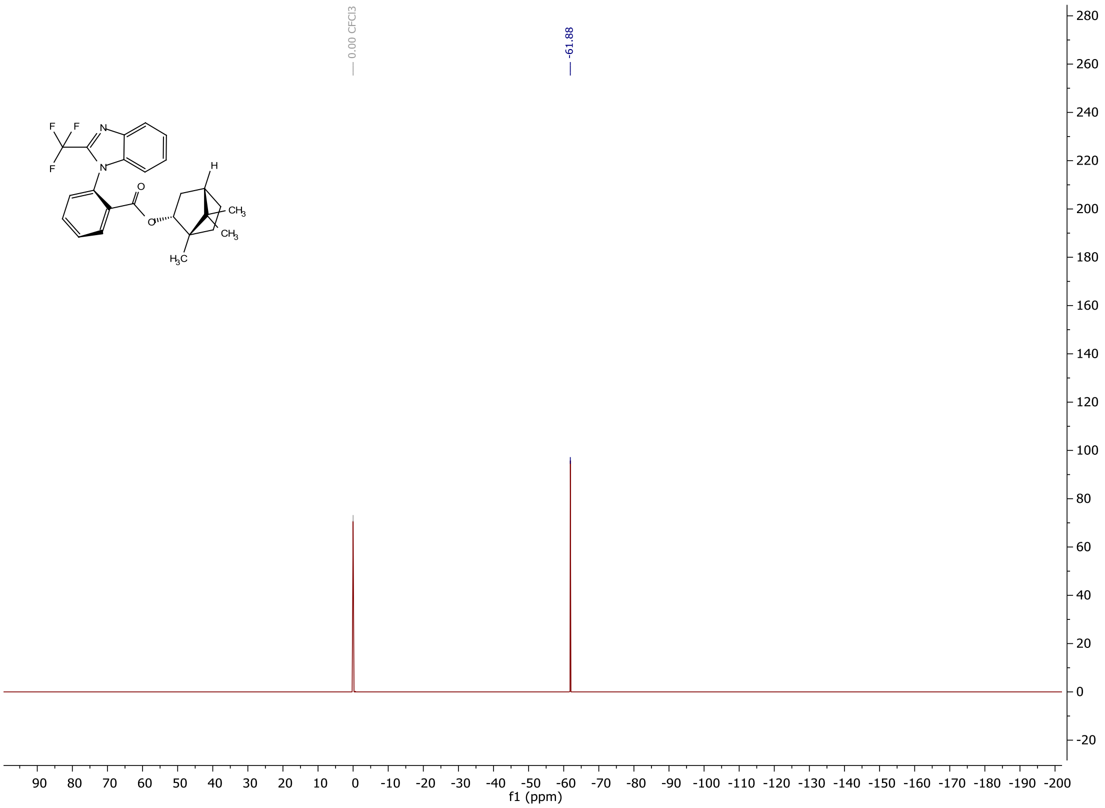

Benzyl (1*R*,3a*S*,5a*R*,5b*R*,7a*R*,9*S*,11a*R*,11b*R*,13a*R*,13b*R*)-5a,5b,8,8,11a-pentamethyl-1-(prop-1-en-2-yl)-9-(((*S*)-2-((*P*)-2-(trifluoromethyl)-1*H*-benzo[*d*]imidazol-1-yl)benzoyl)oxy)icosahydro-3a*H*-cyclopenta[*a*]chrysene-3a-carboxylate **(*P*)-14**

^{19}F NMR (376 MHz, $CDCl_3$)

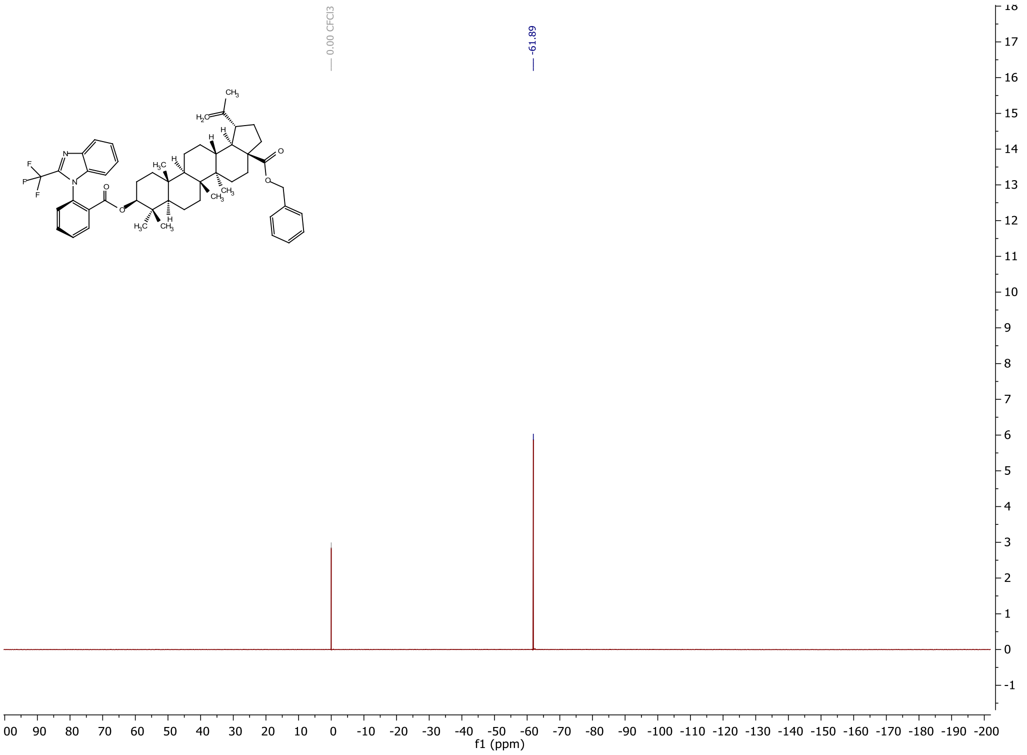

Benzyl (1*R*,3a*S*,5a*R*,5b*R*,7a*R*,9*S*,11a*R*,11b*R*,13a*R*,13b*R*)-5a,5b,8,8,11a-pentamethyl-1-(prop-1-en-2-yl)-9-(((*S*)-2-((*M*)-2-(trifluoromethyl)-1*H*-benzo[*d*]imidazol-1-yl)benzoyl)oxy)icosahydro-3a*H*-cyclopenta[*a*]chrysene-3a-carboxylate **(*M*)-14**

^{19}F NMR (376 MHz, $CDCl_3$)

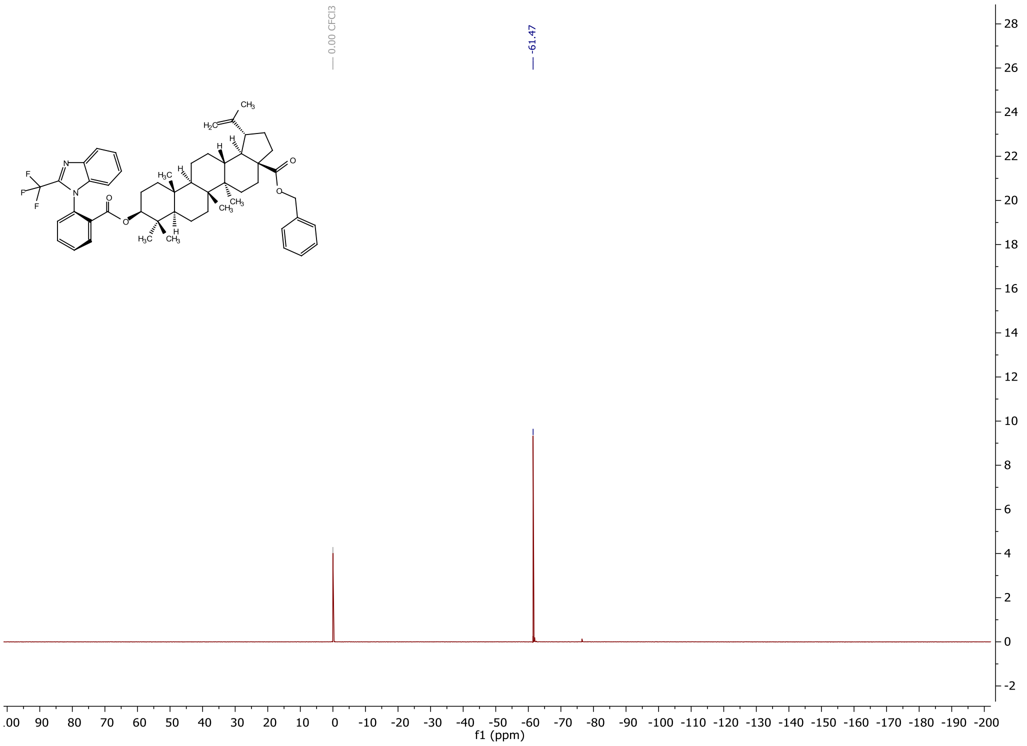

(3*S*,8*S*,9*S*,10*R*,13*R*,14*S*,17*R*)-10,13-Dimethyl-17-((*R*)-6-methylheptan-2-yl)-2,3,4,7,8,9,10,11,12,13,14,15,16,17-tetradecahydro-1*H*-cyclopenta[*a*]phenanthren-3-yl 2-((*P*)-2-(trifluoromethyl)-1*H*-benzo[*d*]imidazol-1-yl)benzoate **(*P*)-15**

^{19}F NMR (376 MHz, $CDCl_3$)

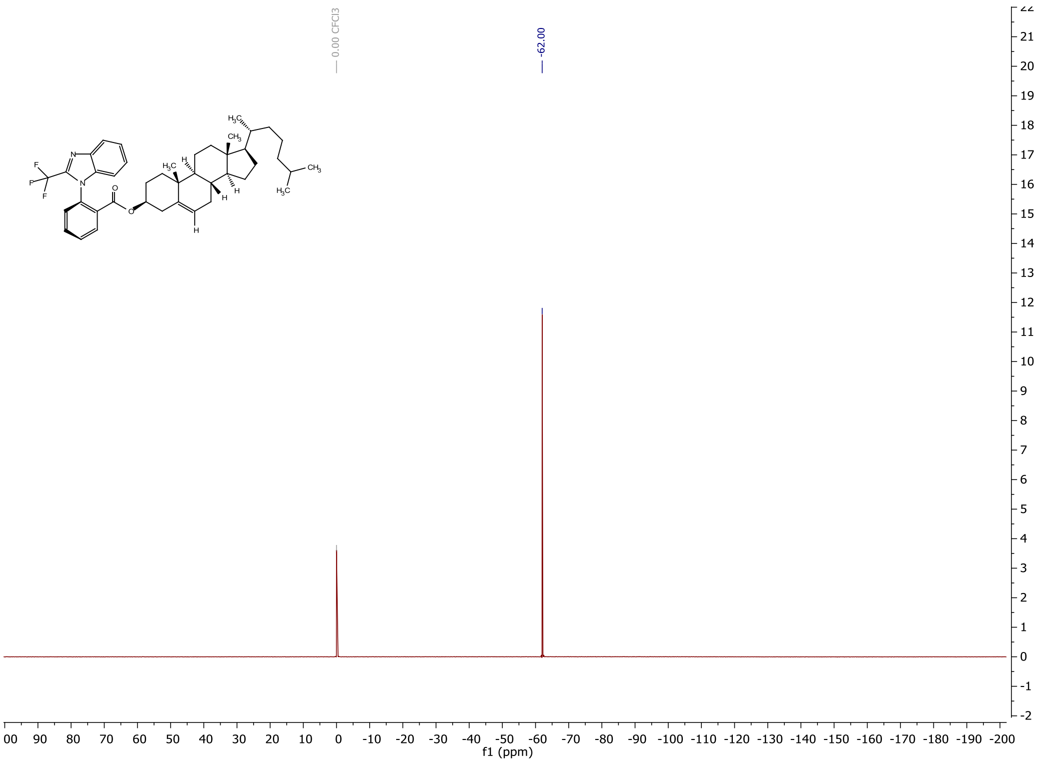

(3*S*,8*S*,9*S*,10*R*,13*R*,14*S*,17*R*)-10,13-Dimethyl-17-((*R*)-6-methylheptan-2-yl)-2,3,4,7,8,9,10,11,12,13,14,15,16,17-tetradecahydro-1*H*-cyclopenta[*a*]phenanthren-3-yl 2-((*M*)-2-(trifluoromethyl)-1*H*-benzo[*d*]imidazol-1-yl)benzoate **(*M*)-15**

^{19}F NMR (376 MHz, $CDCl_3$)

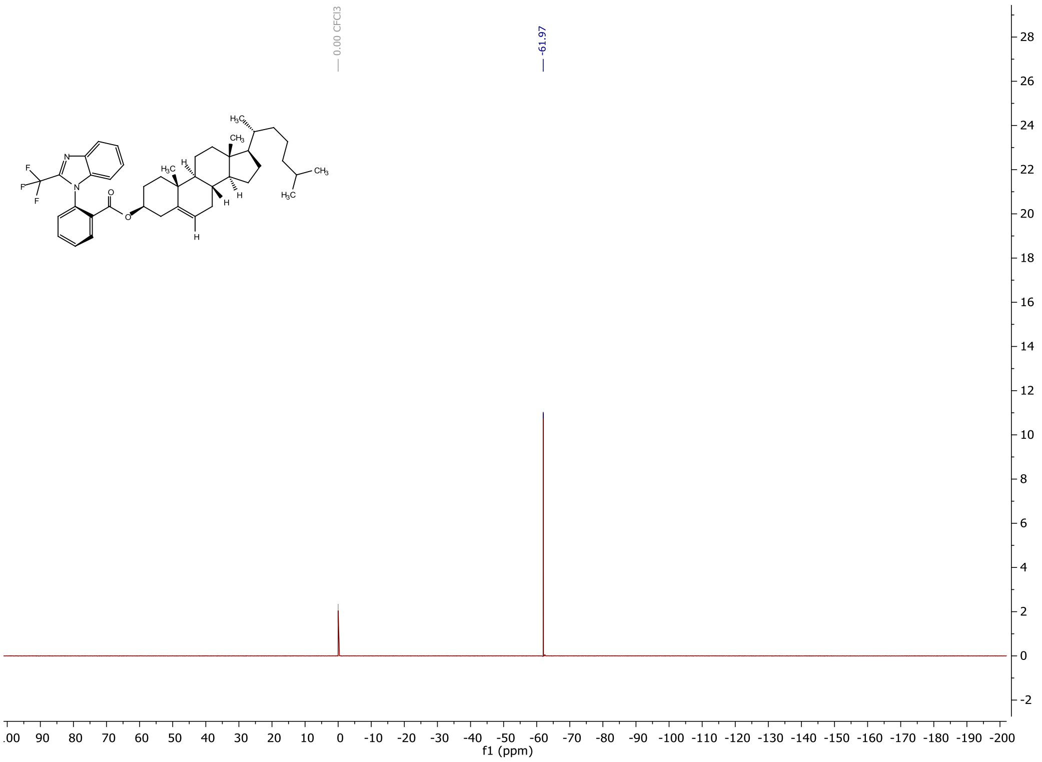

Methyl (*S*)-3,3-dimethyl-2-(2-((*P*)-2-(trifluoromethyl)-1*H*-benzo[*d*]imidazol-1-yl)benzamido)butanoate **(*P*)-16**

^{19}F NMR (376 MHz, $CDCl_3$)

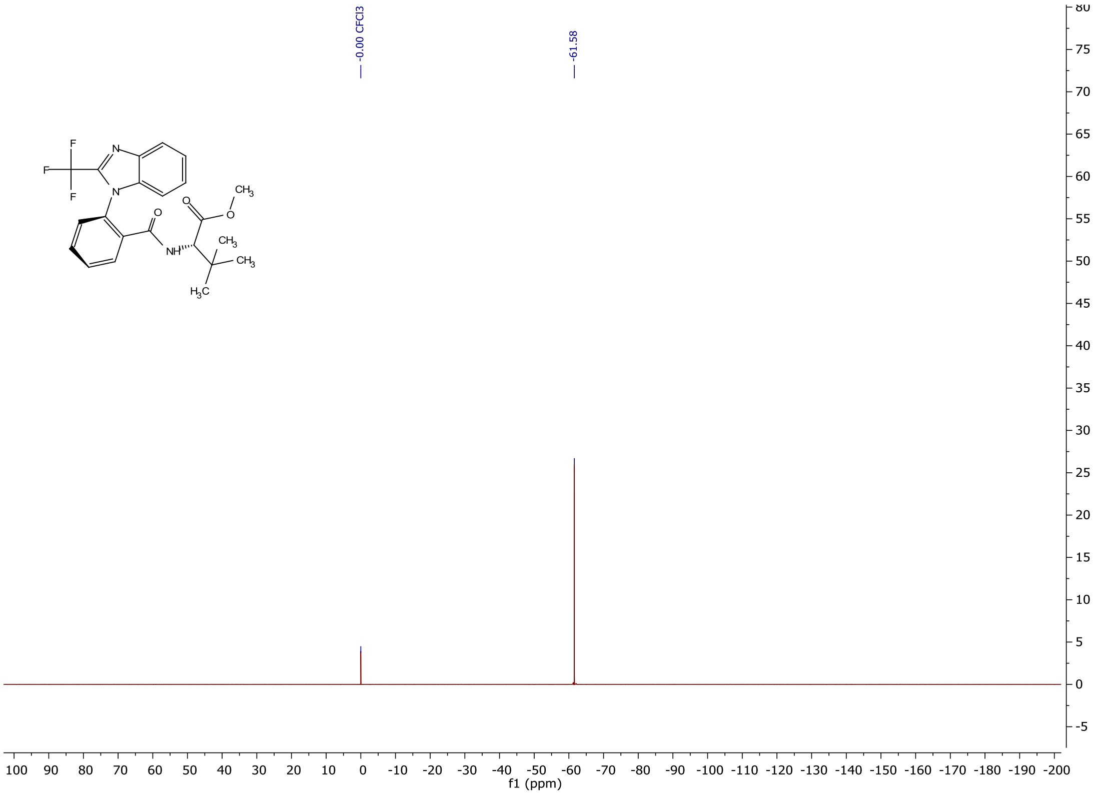

Methyl (*S*)-3,3-dimethyl-2-(2-((*M*)-2-(trifluoromethyl)-1*H*-benzo[*d*]imidazol-1-yl)benzamido)butanoate **(*M*)-16**

^{19}F NMR (376 MHz, $CDCl_3$)

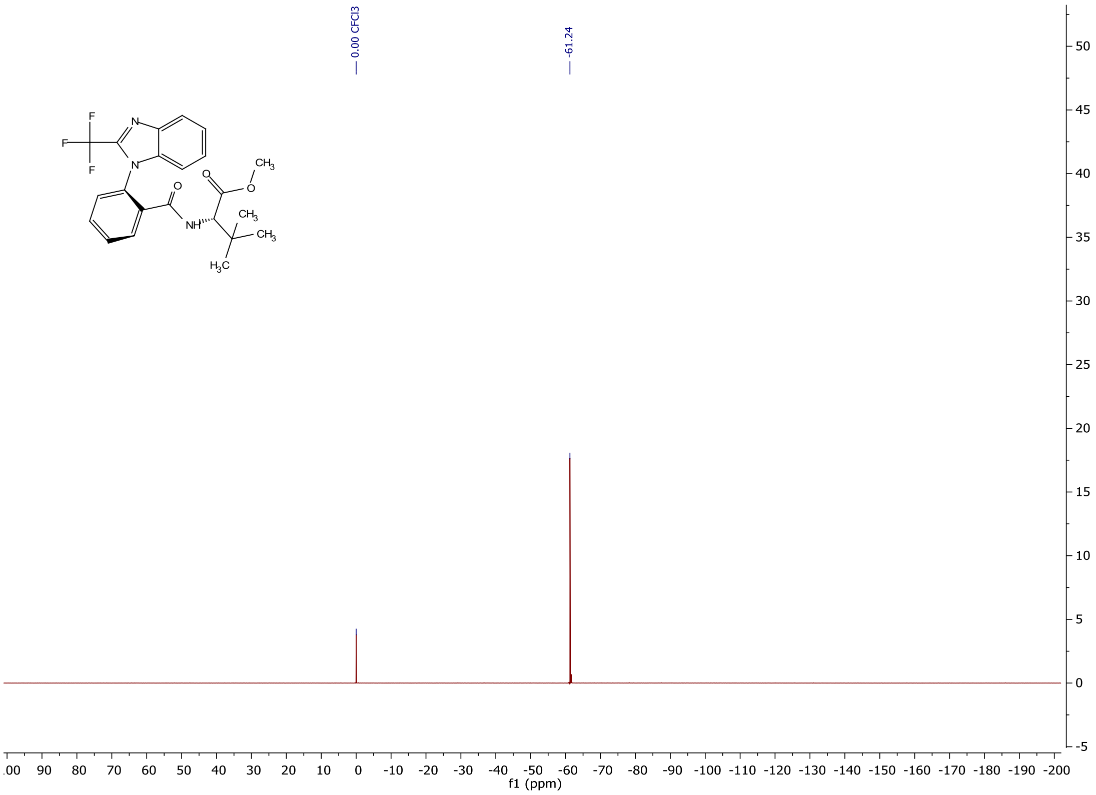

Methyl (2-((*P*)-2-(trifluoromethyl)-1*H*-benzo[*d*]imidazol-1-yl)benzoyl)-*L*-valinate **(*P*)-17**

^{19}F NMR (376 MHz, $CDCl_3$)

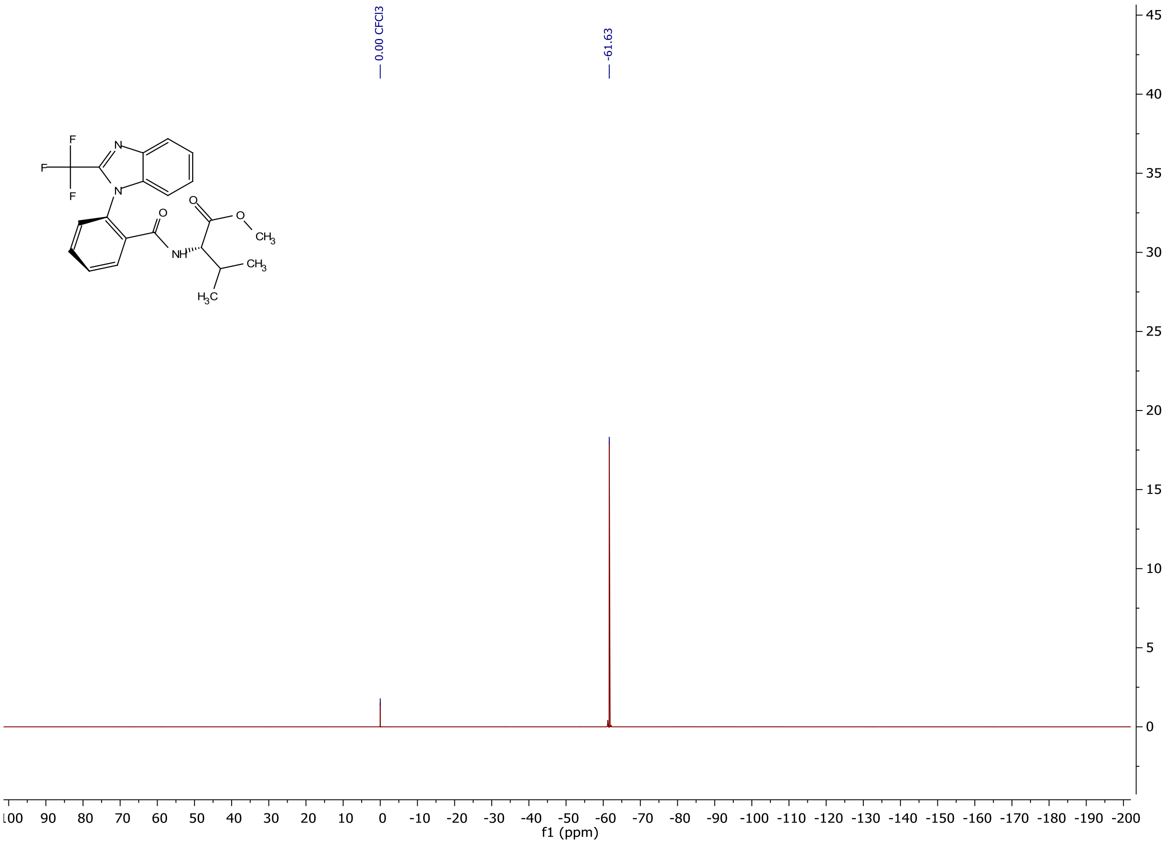

Methyl (2-((*M*)-2-(trifluoromethyl)-1*H*-benzo[*d*]imidazol-1-yl)benzoyl)-*L*-valinate **(*M*)-17**

^{19}F NMR (376 MHz, $CDCl_3$)

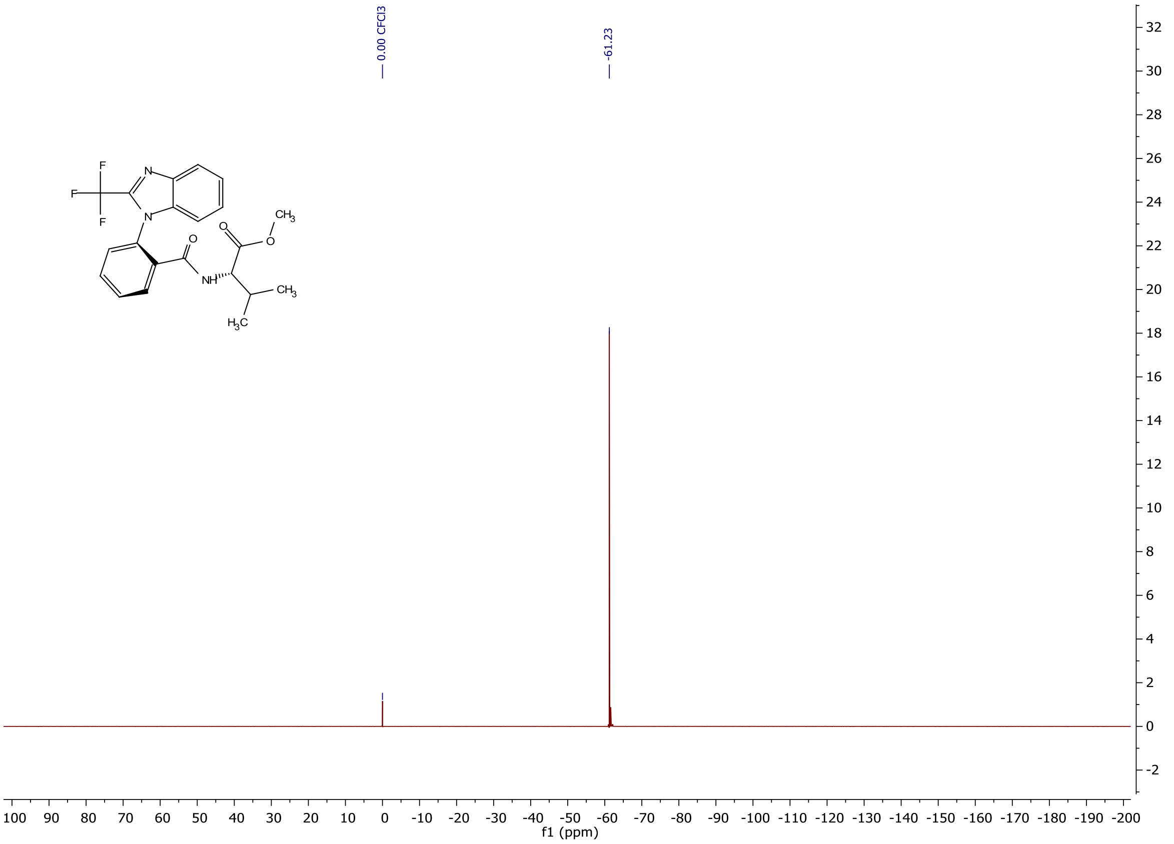

Methyl (2-((*P*)-2-(trifluoromethyl)-1*H*-benzo[*d*]imidazol-1-yl)benzoyl)-*L*-phenylalaninate **(*P*)-18**

^{19}F NMR (376 MHz, $CDCl_3$)

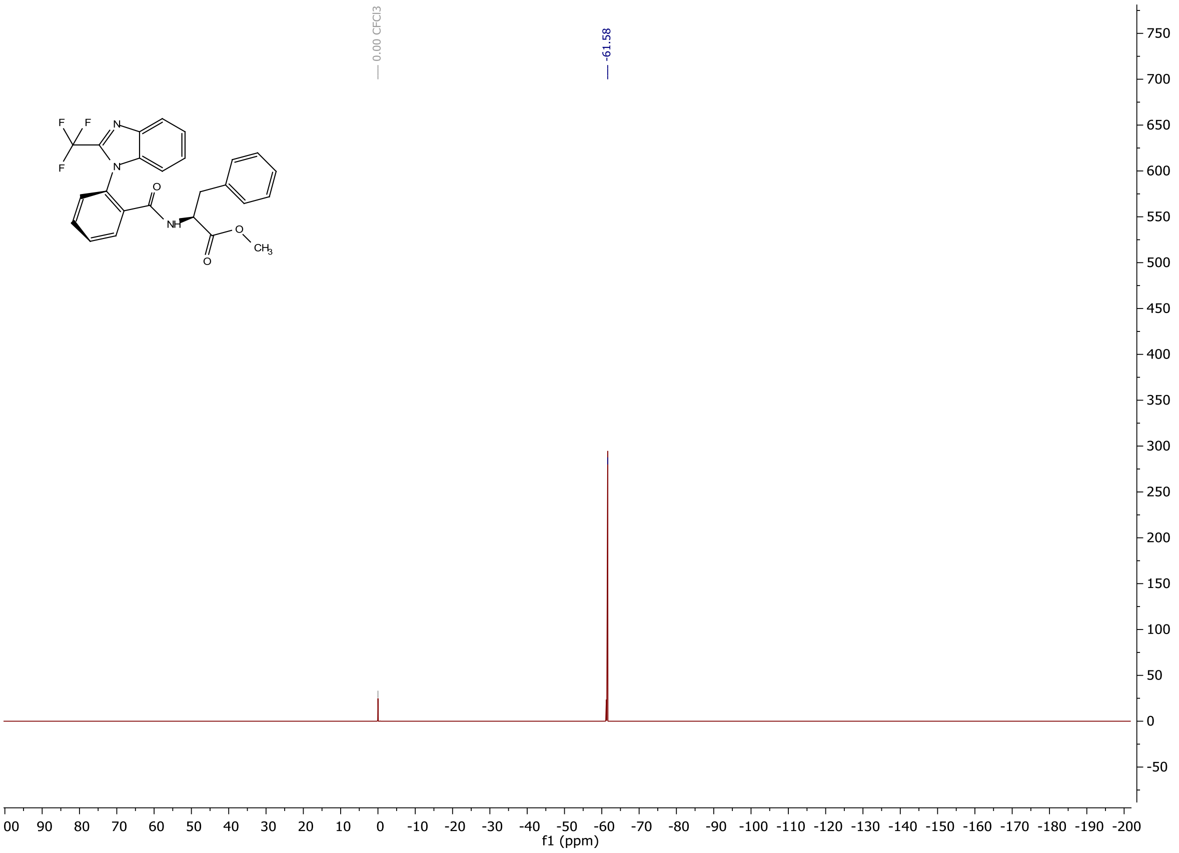

Methyl (2-((*M*)-2-(trifluoromethyl)-1*H*-benzo[*d*]imidazol-1-yl)benzoyl)-*L*-phenylalaninate **(*M*)-18**

^{19}F NMR (376 MHz, $CDCl_3$)

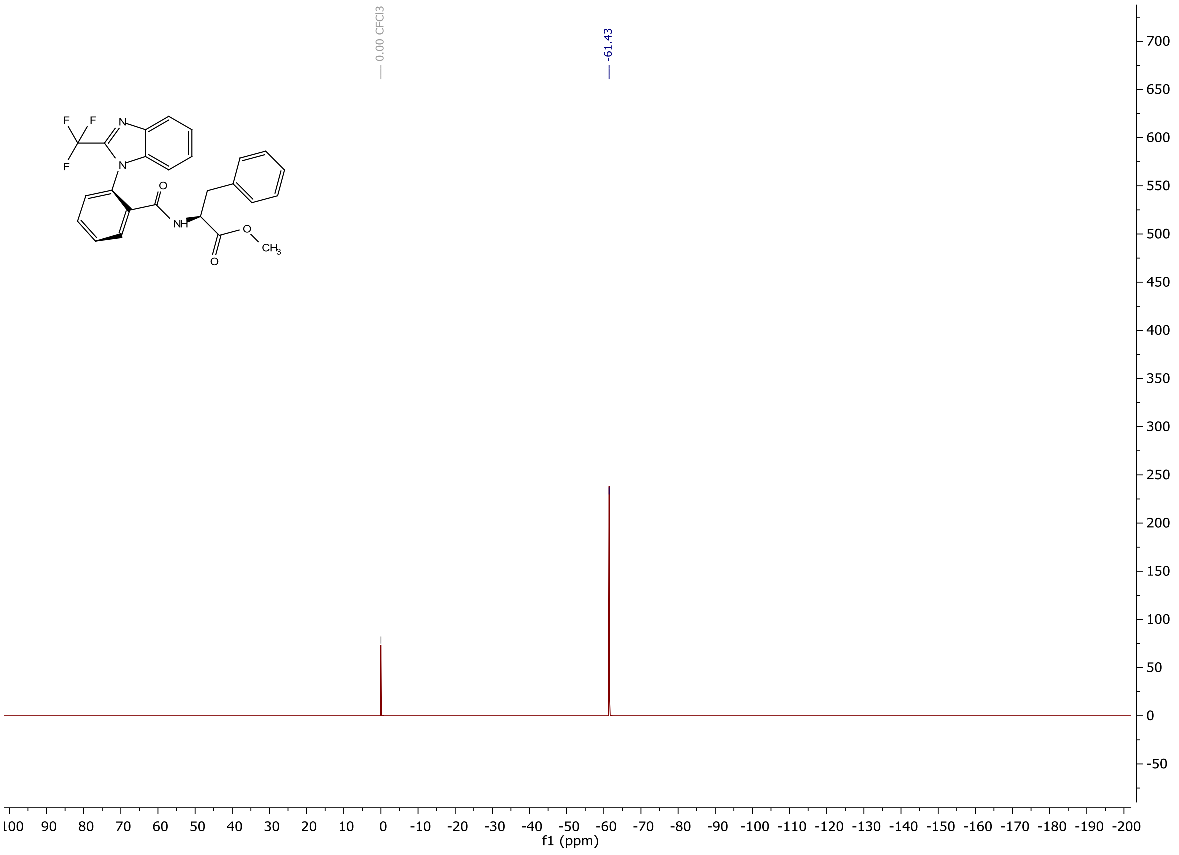

tert-Butyl *O*-(*tert*-butyl)-*N*-(2-((*P*)-2-(trifluoromethyl)-1*H*-benzo[d]imidazol-1-yl)benzoyl)-*L*-serinate **(*P*)-19**

^{19}F NMR (376 MHz, $CDCl_3$)

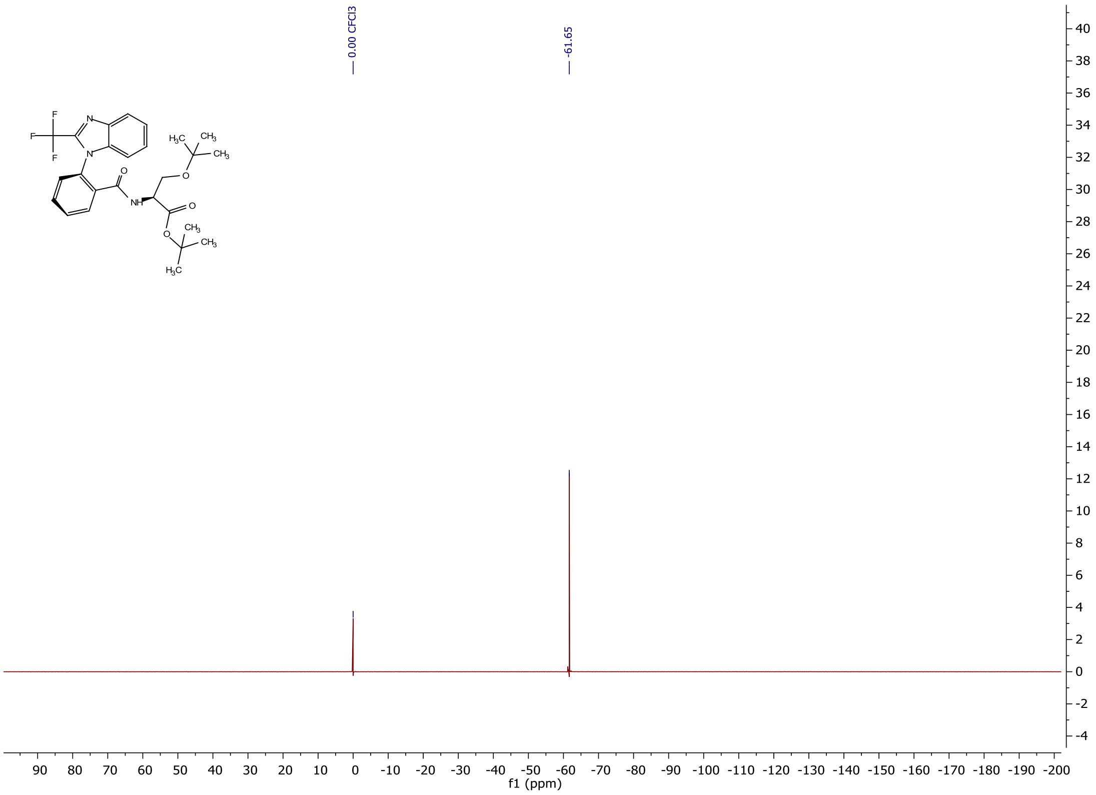

tert-Butyl *O*-(*tert*-butyl)-*N*-(2-((*M*)-2-(trifluoromethyl)-1*H*-benzo[d]imidazol-1-yl)benzoyl)-*L*-serinate **(*M*)-19**

^{19}F NMR (376 MHz, $CDCl_3$)

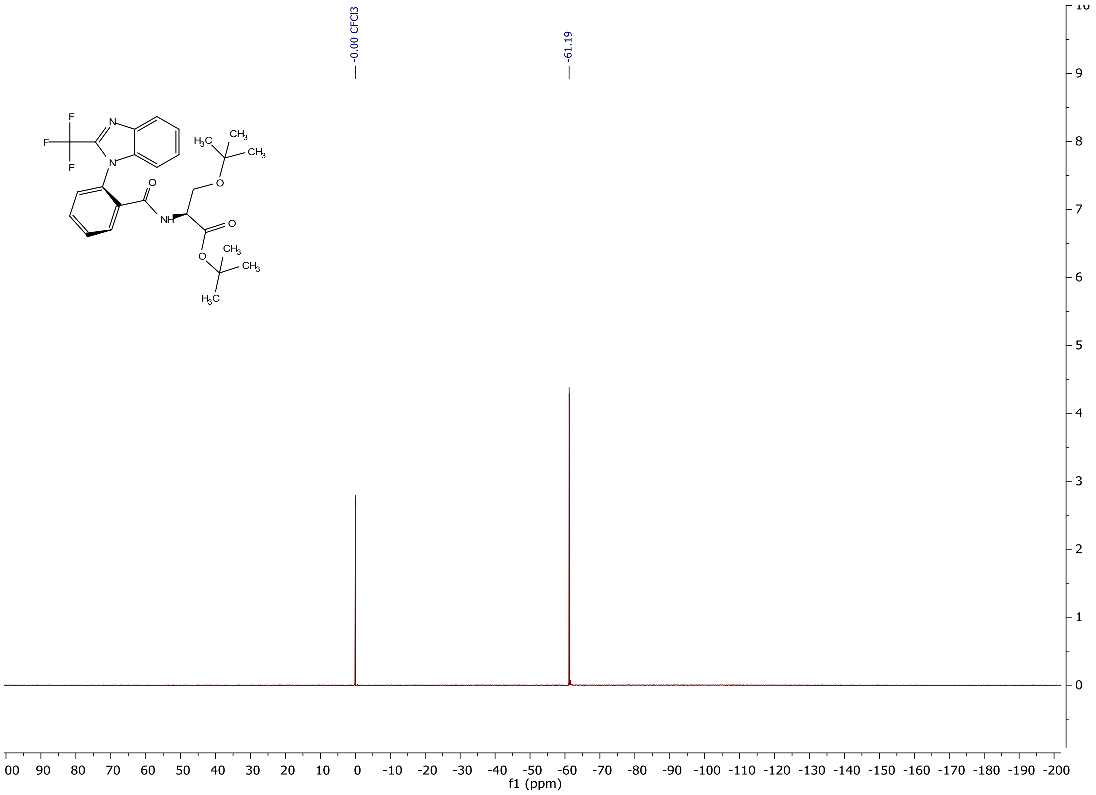

Methyl (2-((*P*)-2-(trifluoromethyl)-1*H*-benzo[*d*]imidazol-1-yl)benzoyl)-*L*-leucinate **(*P*)-20**

^{19}F NMR (376 MHz, $CDCl_3$)

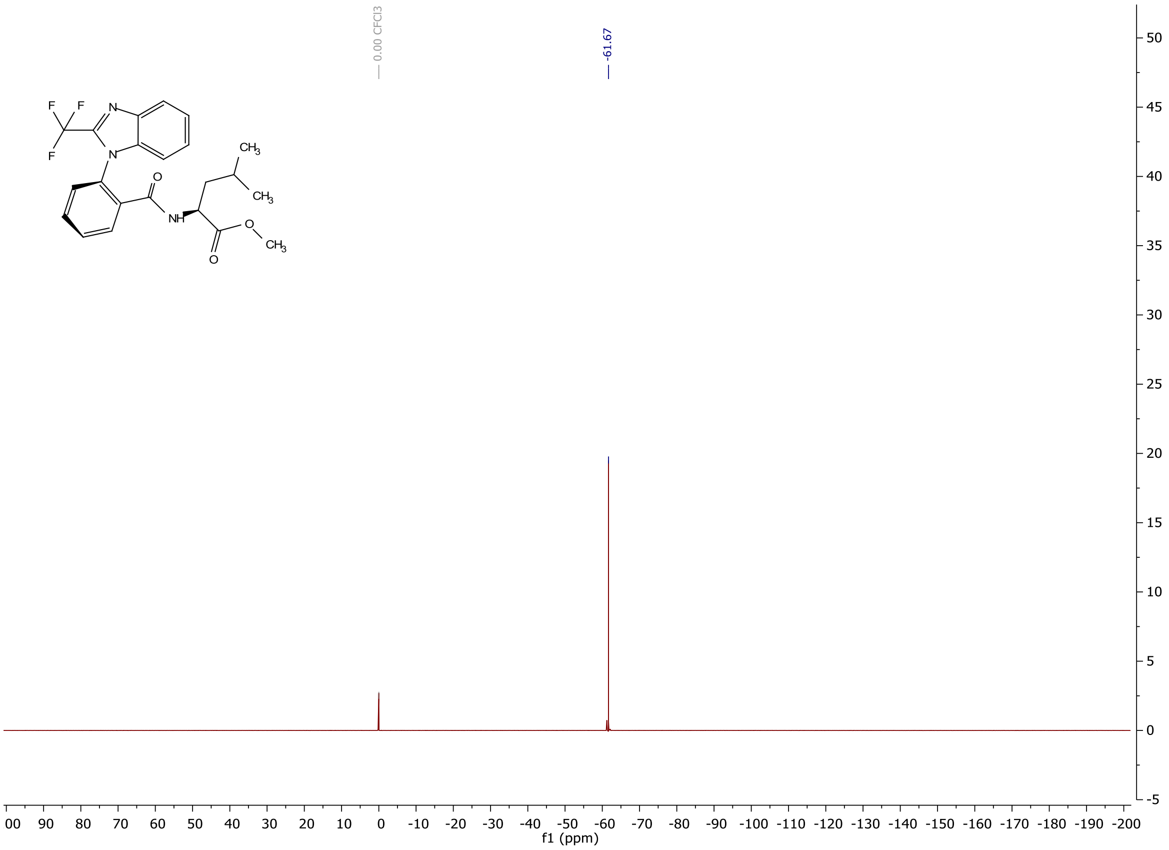

Methyl (2-((*M*)-2-(trifluoromethyl)-1*H*-benzo[*d*]imidazol-1-yl)benzoyl)-*L*-leucinate **(*M*)-20**

^{19}F NMR (376 MHz, $CDCl_3$)

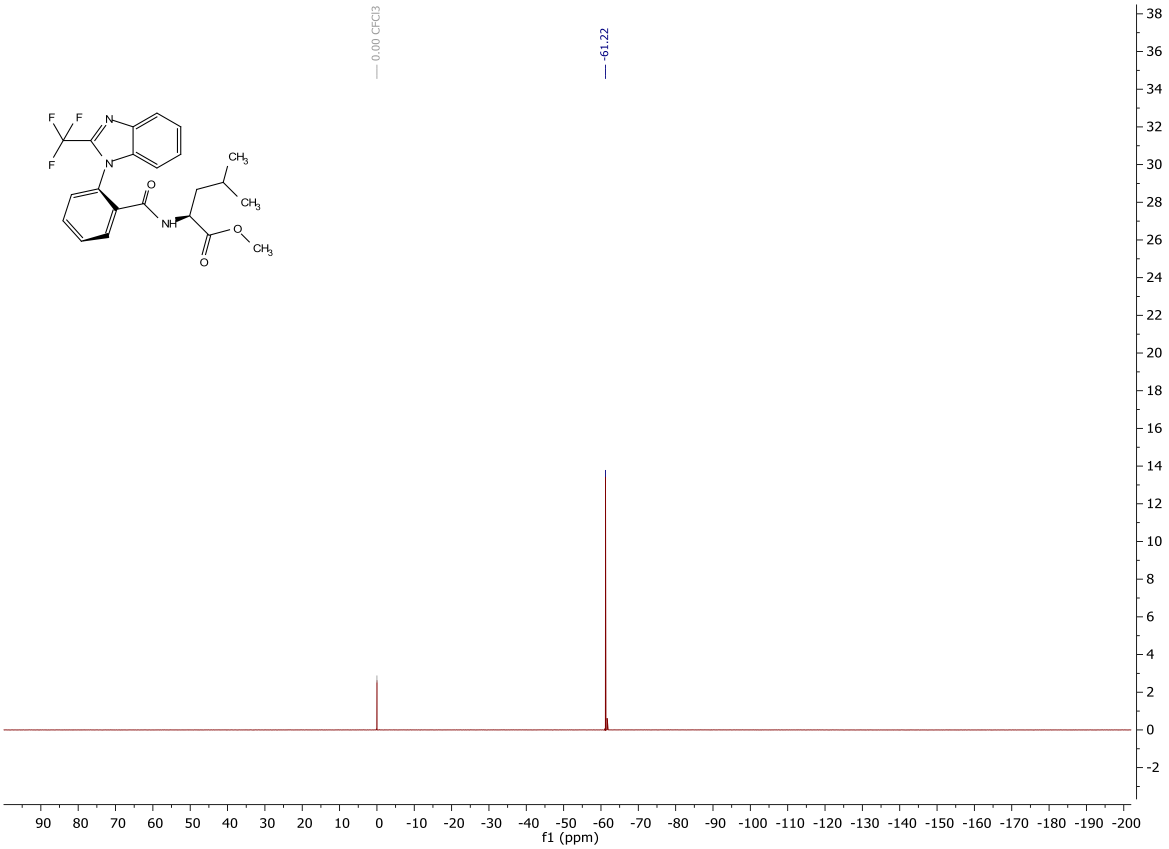

Methyl (2-((*P*)-2-(trifluoromethyl)-1*H*-benzo[*d*]imidazol-1-yl)benzoyl)-*L*-alaninate **(*P*)-21**

^{19}F NMR (376 MHz, $CDCl_3$)

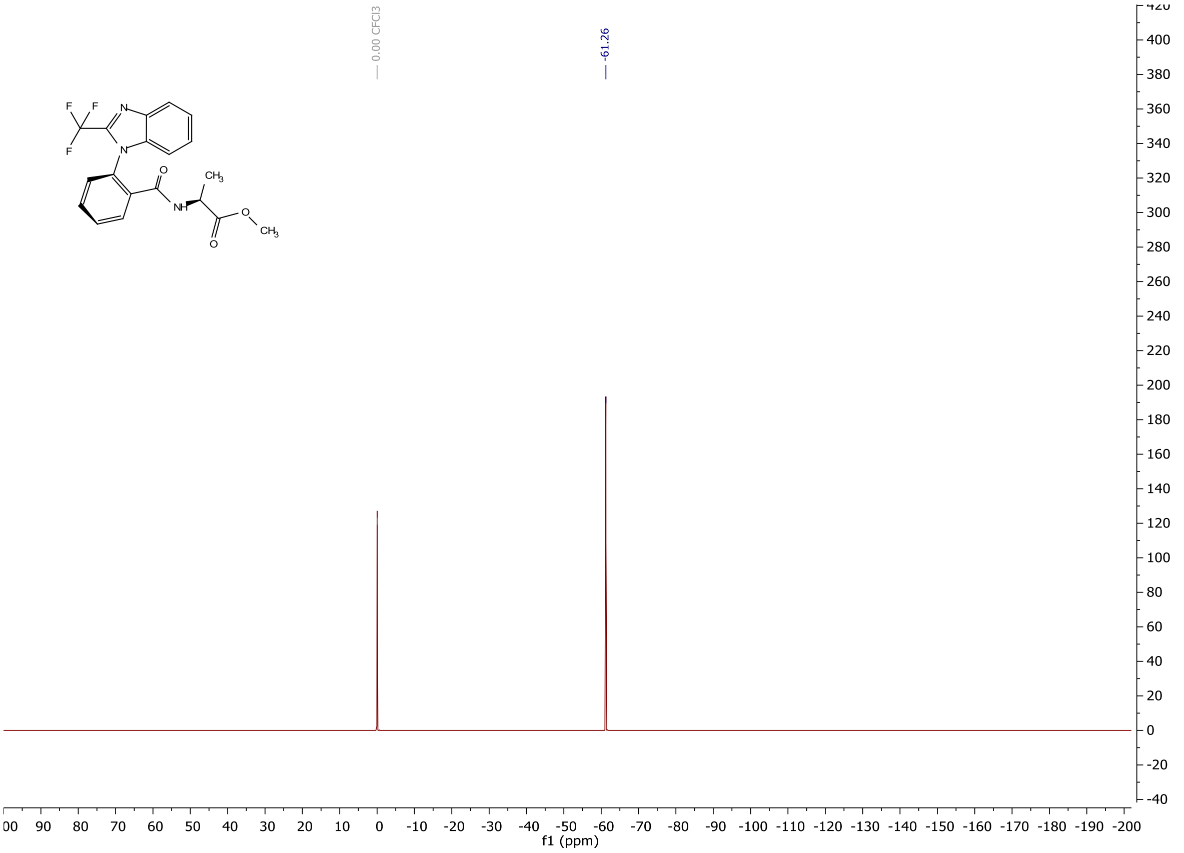

Methyl (2-((*M*)-2-(trifluoromethyl)-1*H*-benzo[*d*]imidazol-1-yl)benzoyl)-*L*-alaninate **(*M*)-21**

^{19}F NMR (376 MHz, $CDCl_3$)

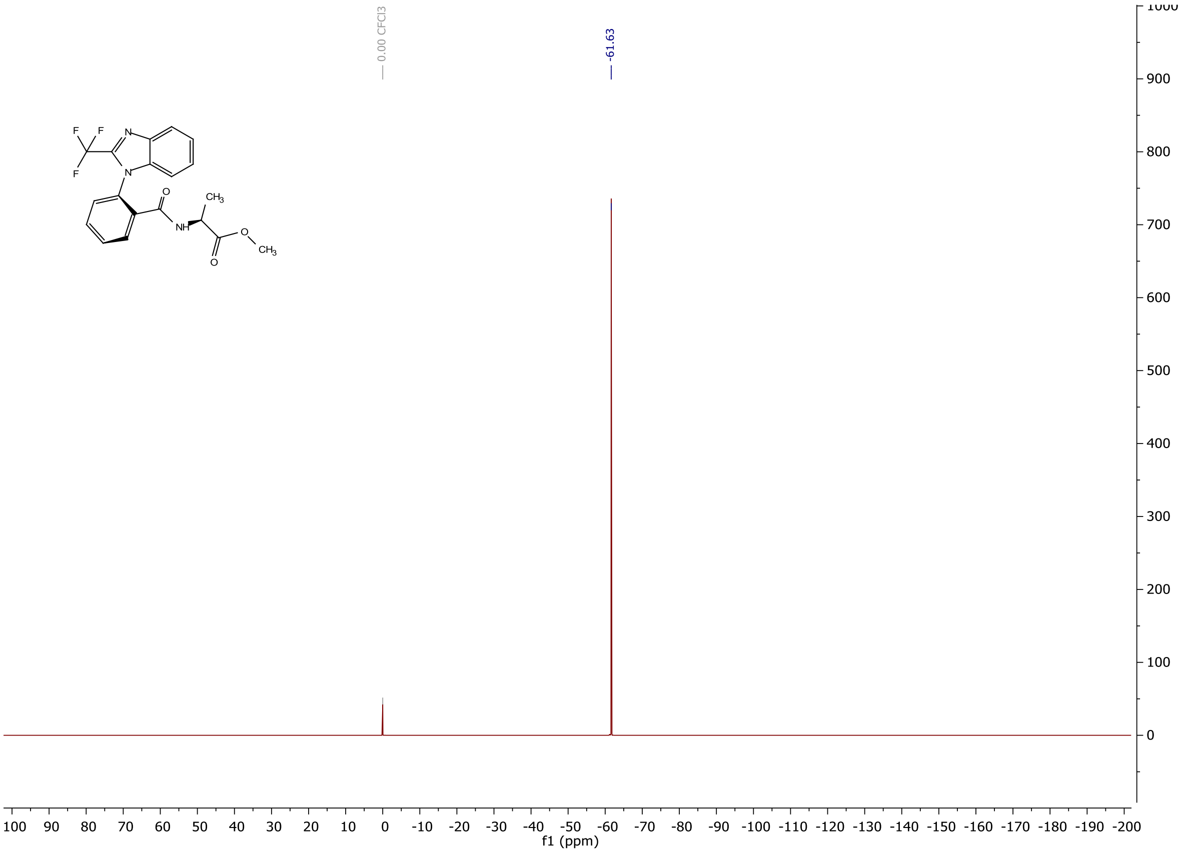

N-((*S*)-1-Amino-1-oxopropan-2-yl)-2-((*P*)-2-(trifluoromethyl)-1*H*-benzo[*d*]imidazol-1-yl)benzamide **(*P*)-22**

^{19}F NMR (376 MHz, $CDCl_3$)

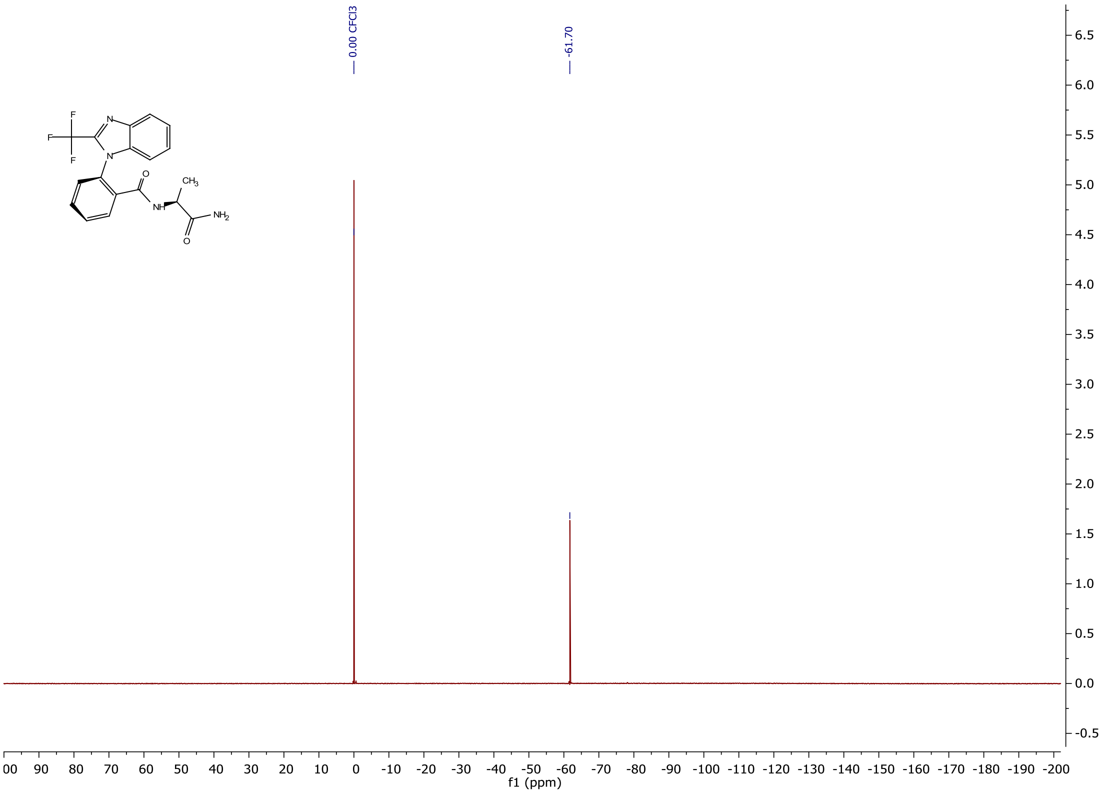

N-((*S*)-1-Amino-1-oxopropan-2-yl)-2-((*M*)-2-(trifluoromethyl)-1*H*-benzo[*d*]imidazol-1-yl)benzamide **(*M*)-22**

^{19}F NMR (376 MHz, $CDCl_3$)

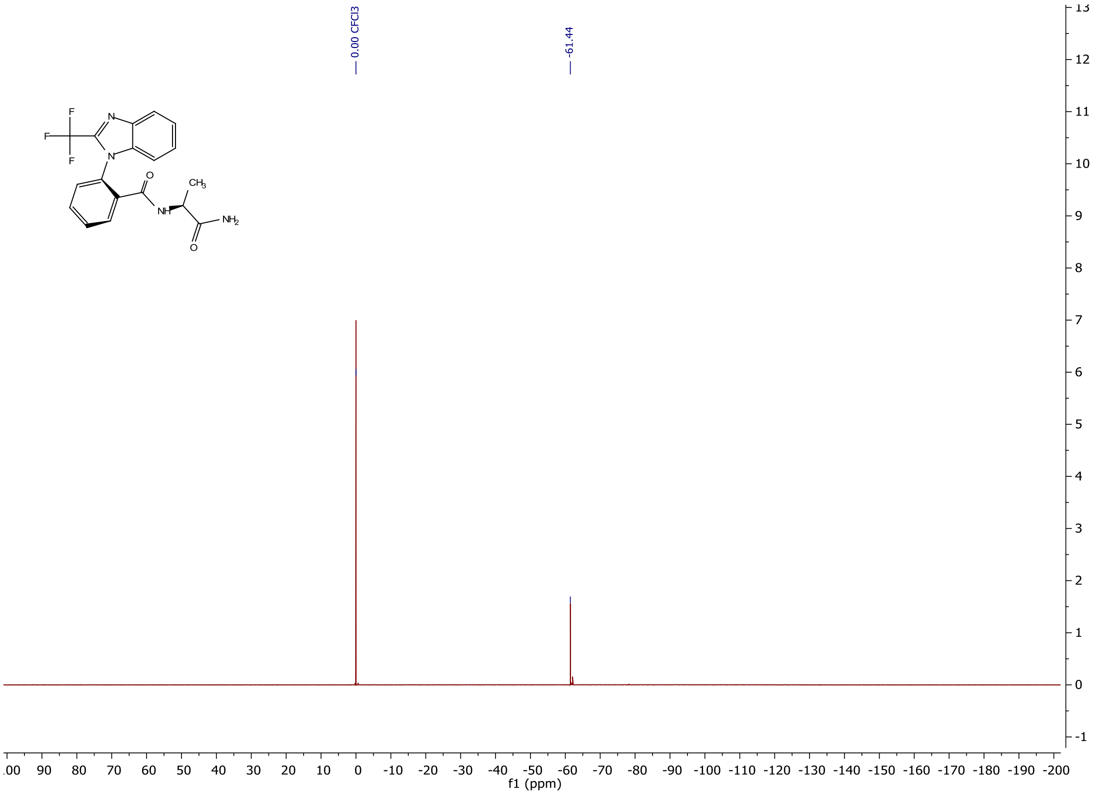

N-((*S*)-1-(Diethylamino)-1-oxopropan-2-yl)-2-((*P*)-2-(trifluoromethyl)-1*H*-benzo[*d*]imidazol-1-yl)benzamide **(*P*)-23**

^{19}F NMR (376 MHz, $CDCl_3$)

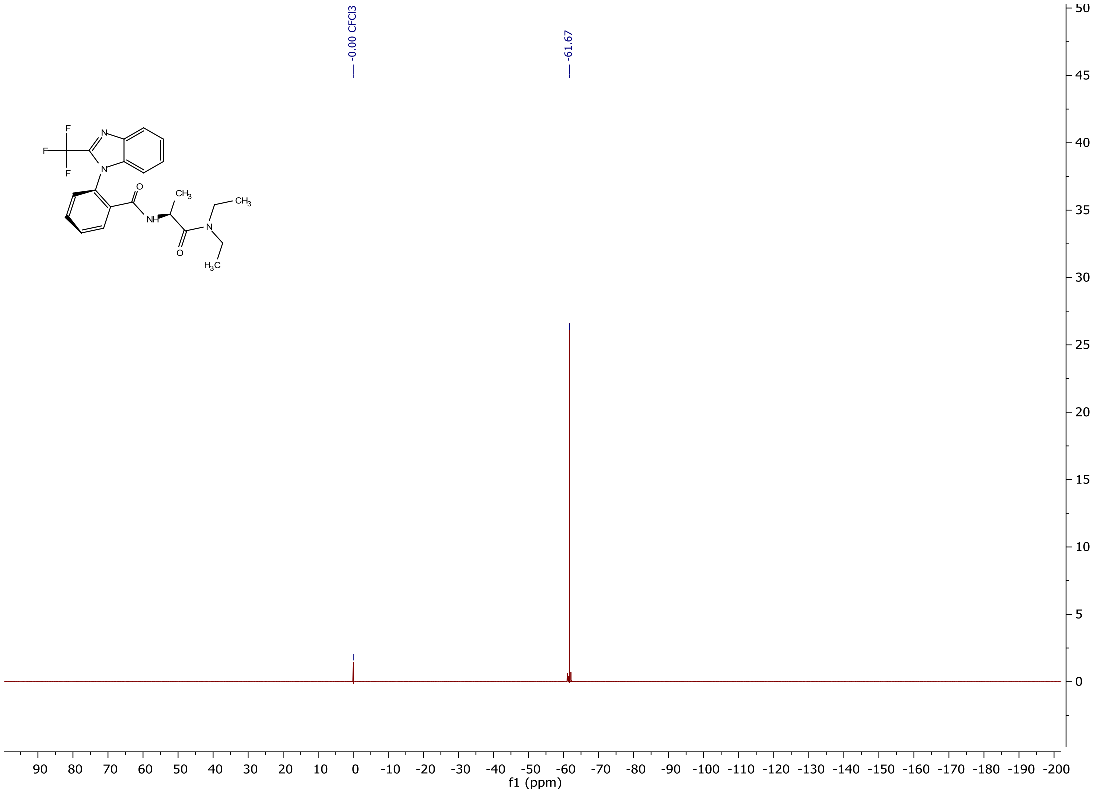

N-((*S*)-1-(Diethylamino)-1-oxopropan-2-yl)-2-((*M*)-2-(trifluoromethyl)-1*H*-benzo[*d*]imidazol-1-yl)benzamide **(*M*)-23**

^{19}F NMR (376 MHz, $CDCl_3$)

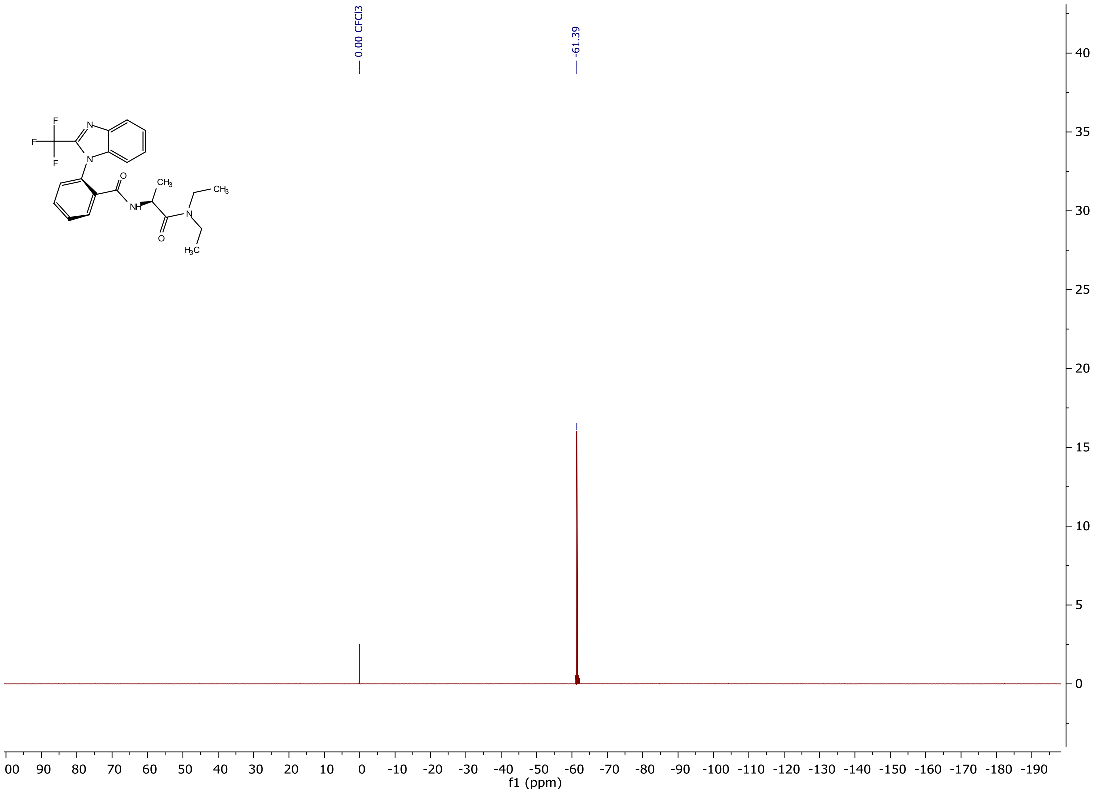

N-((*S*)-3,3-Dimethylbutan-2-yl)-2-((*P*)-2-(trifluoromethyl)-1*H*-benzo[*d*]imidazol-1-yl)benzamide **(*P*)-24**

^{19}F NMR (376 MHz, $CDCl_3$)

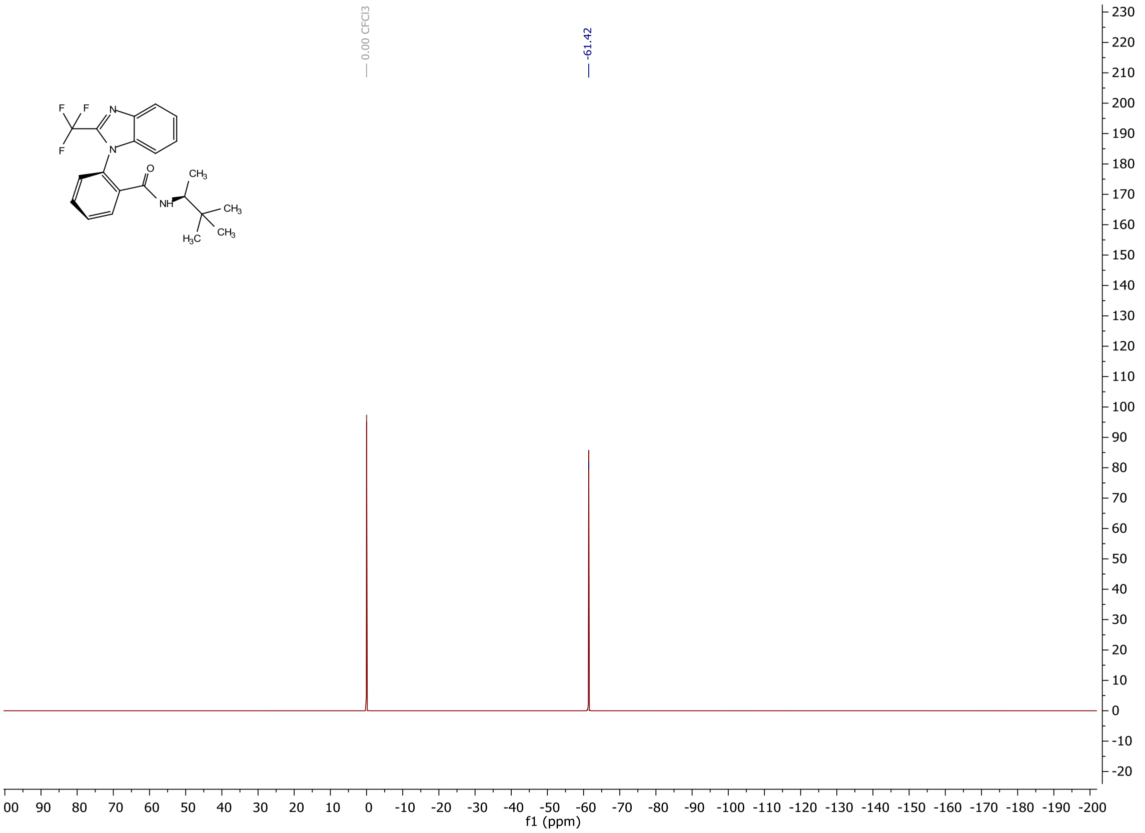

N-((*S*)-3,3-Dimethylbutan-2-yl)-2-((*M*)-2-(trifluoromethyl)-1*H*-benzo[*d*]imidazol-1-yl)benzamide **(*M*)-24**

^{19}F NMR (376 MHz, $CDCl_3$)

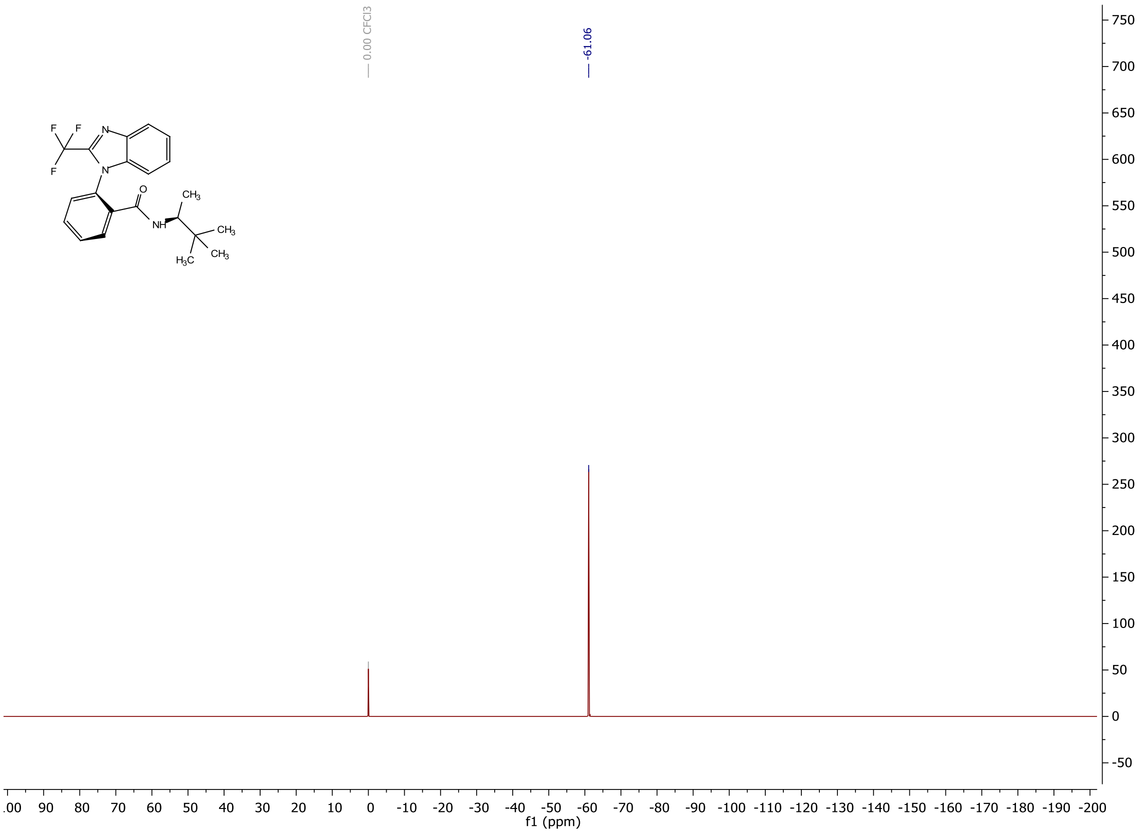

N-((*S*)-1-Cyclohexylethyl)-2-((*P*)-2-(trifluoromethyl)-1*H*-benzo[*d*]imidazol-1-yl)benzamide **(*P*)-25**

^{19}**F NMR** (376 MHz, $CDCl_3$)

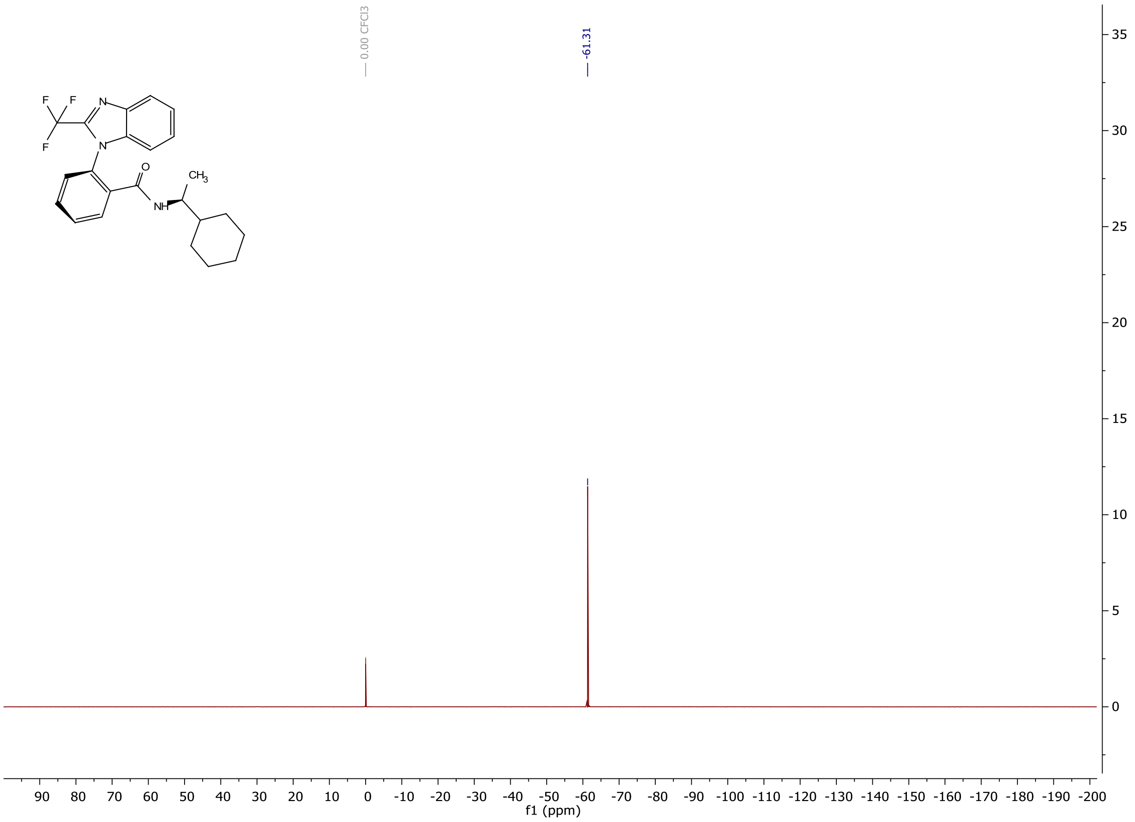

N-((*S*)-1-Cyclohexylethyl)-2-((*M*)-2-(trifluoromethyl)-1*H*-benzo[*d*]imidazol-1-yl)benzamide **(*M*)-25**

^{19}**F NMR** (376 MHz, $CDCl_3$)

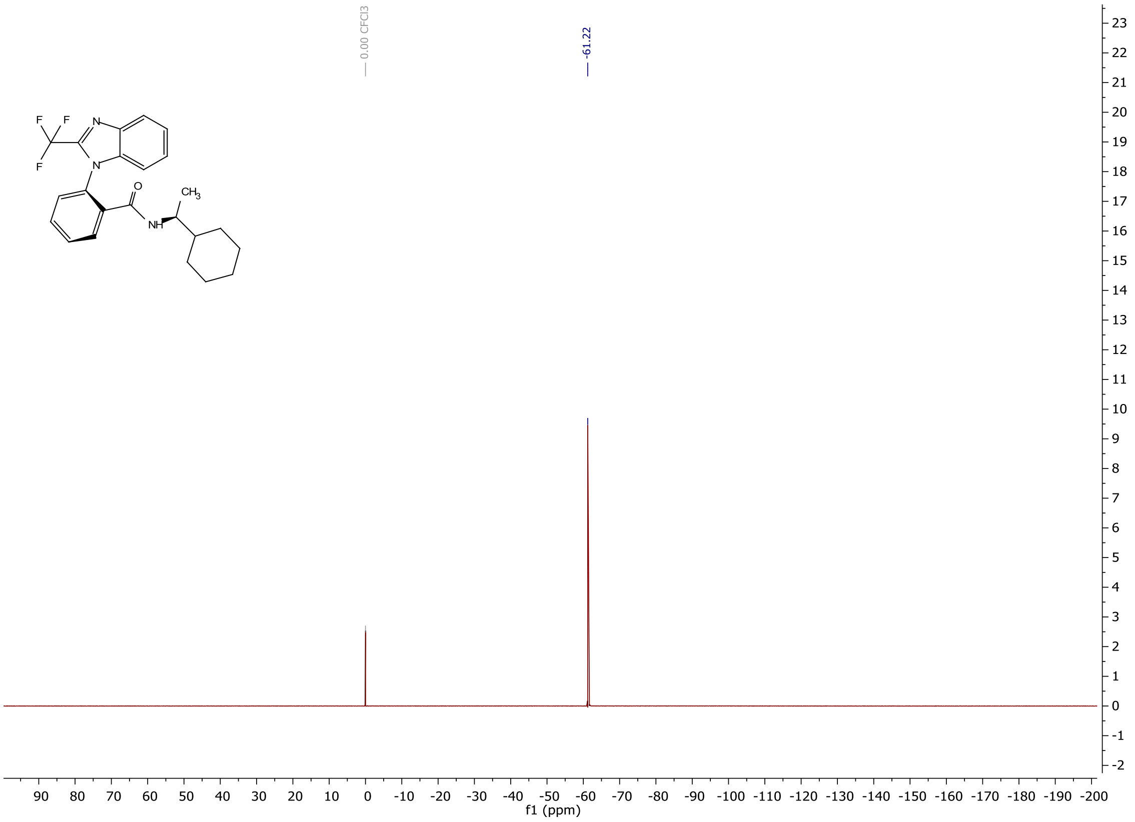

N-((*S*)-2-Methyl-1-phenylpropyl)-2-((*P*)-2-(trifluoromethyl)-1*H*-benzo[*d*]imidazol-1-yl)benzamide **(*P*)-26**

^{19}F NMR (376 MHz, $CDCl_3$)

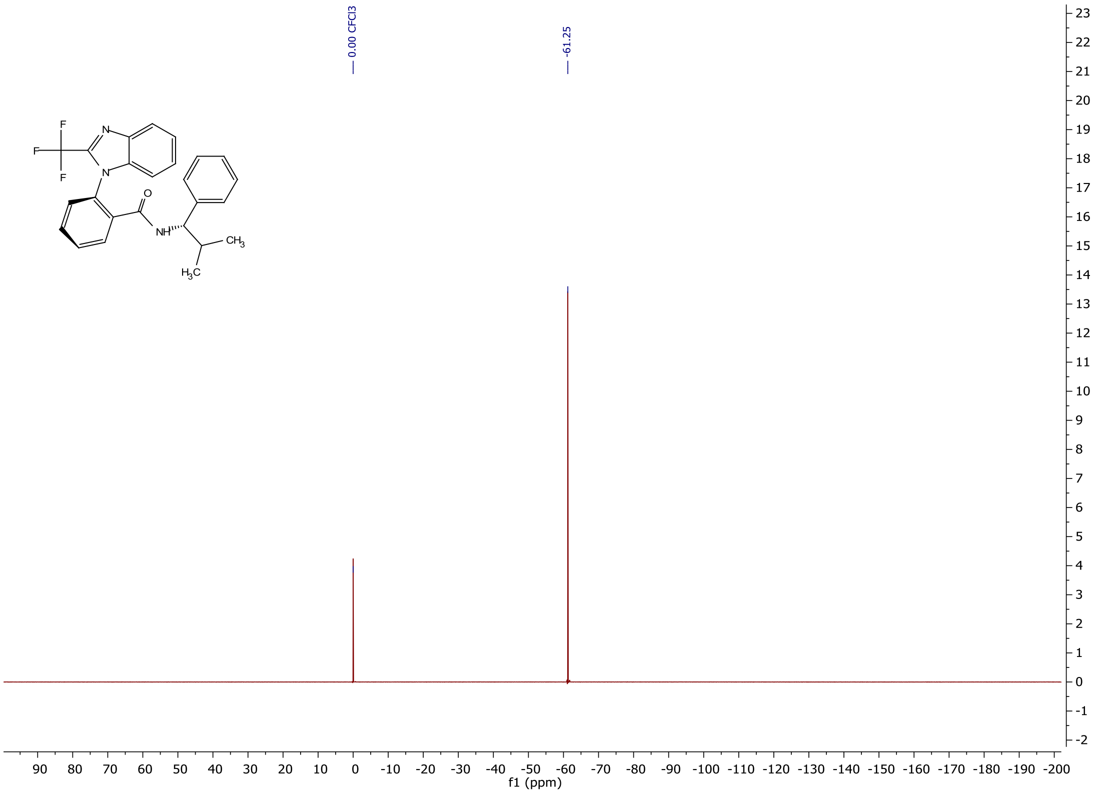

N-((*S*)-2-Methyl-1-phenylpropyl)-2-((*M*)2-(trifluoromethyl)-1*H*-benzo[*d*]imidazol-1-yl)benzamide **(*M*)-26**

^{19}F NMR (376 MHz, $CDCl_3$)

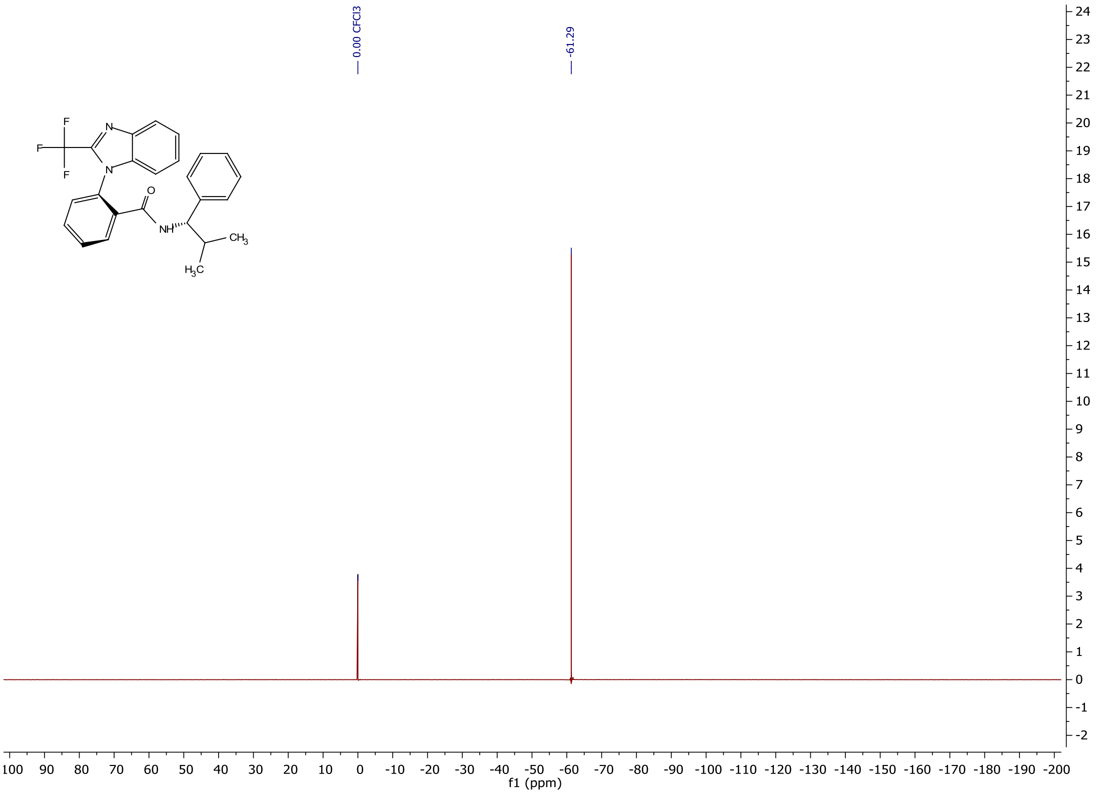

N-((*R*)-1-Phenylethyl)-2-((*P*)-2-(trifluoromethyl)-1*H*-benzo[*d*]imidazol-1-yl)benzamide **(*P*)-27**

^{19}F NMR (376 MHz, $CDCl_3$)

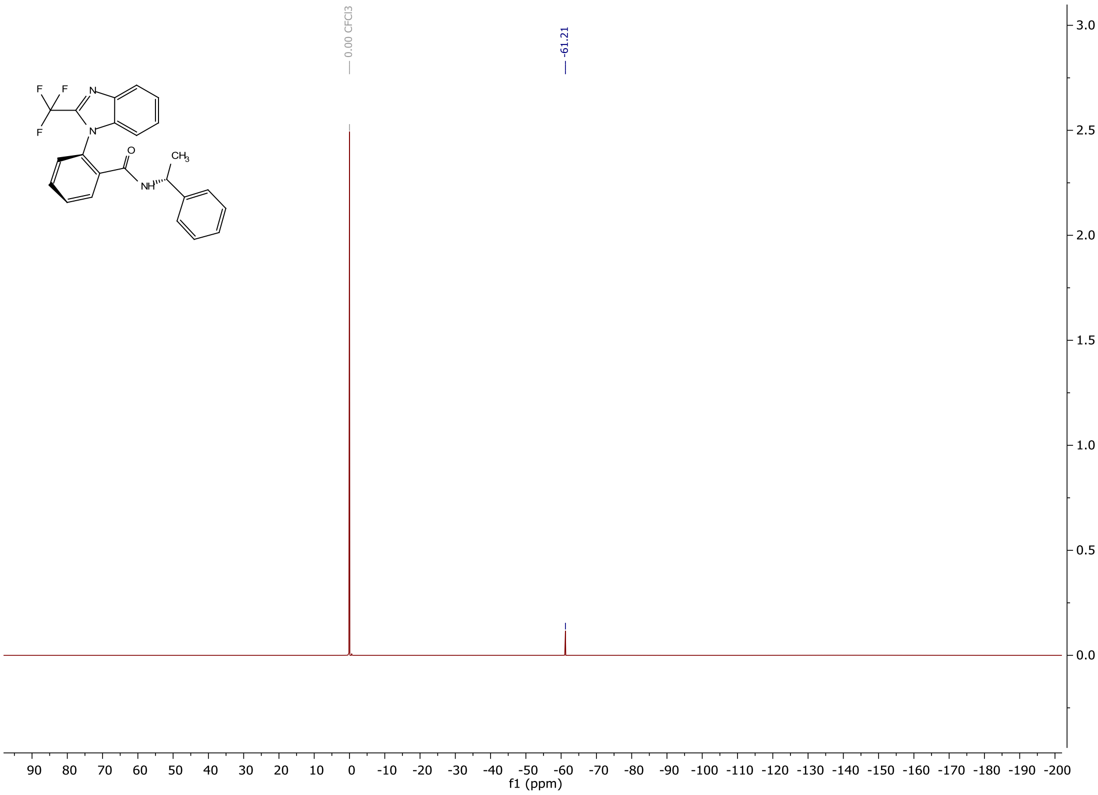

N-((*R*)-1-Phenylethyl)-2-((*M*)-2-(trifluoromethyl)-1*H*-benzo[*d*]imidazol-1-yl)benzamide **(*M*)-27**

^{19}F NMR (376 MHz, $CDCl_3$)

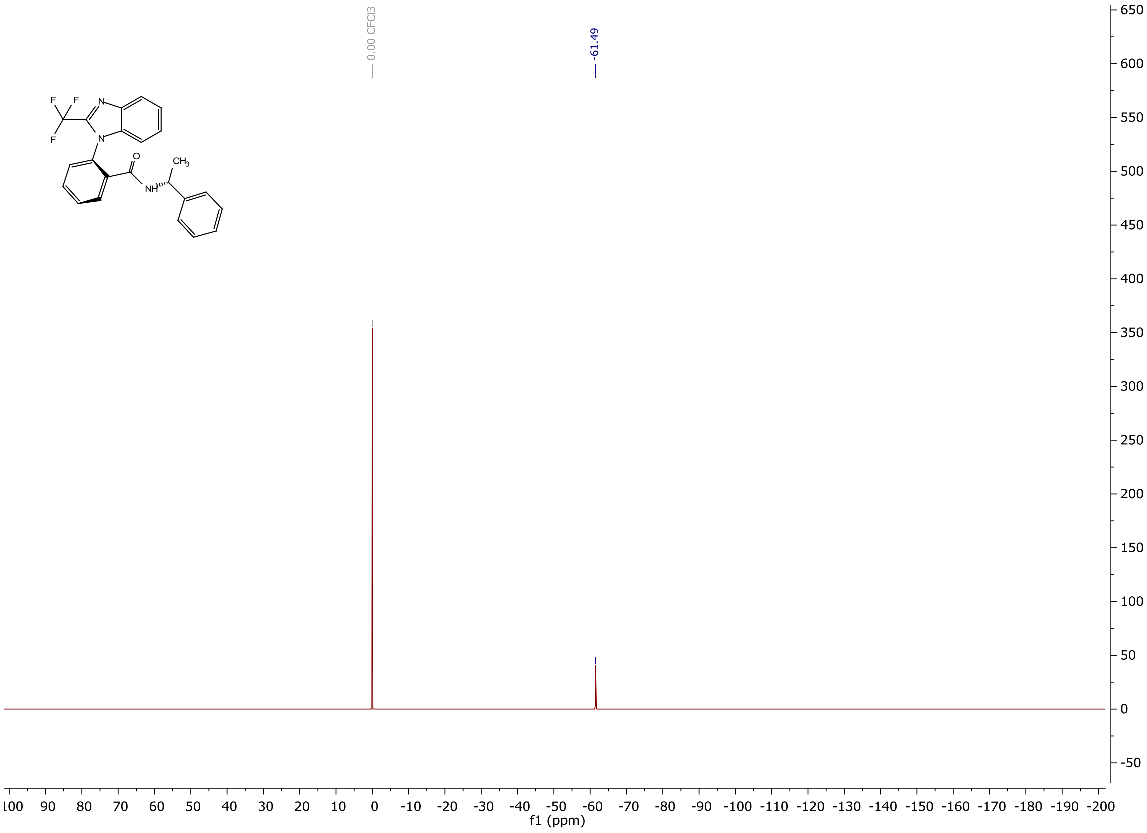

N-((*R*)-1-(Naphthalen-1-yl)ethyl)-2-((*P*)-2-(trifluoromethyl)-1*H*-benzo[*d*]imidazol-1-yl)benzamide **(*P*)-28**

^{19}F NMR (376 MHz, $CDCl_3$)

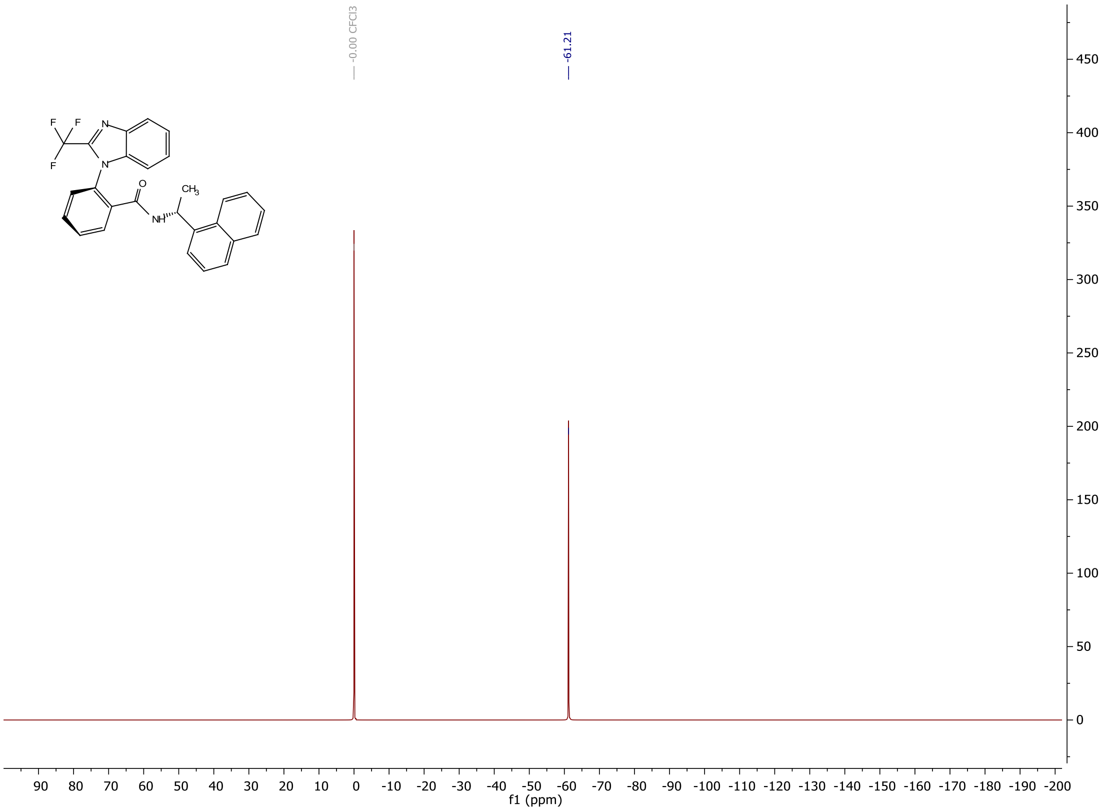

N-((*R*)-1-(Naphthalen-1-yl)ethyl)-2-((*M*)-2-(trifluoromethyl)-1*H*-benzo[*d*]imidazol-1-yl)benzamide **(*M*)-28**

^{19}F NMR (376 MHz, $CDCl_3$)

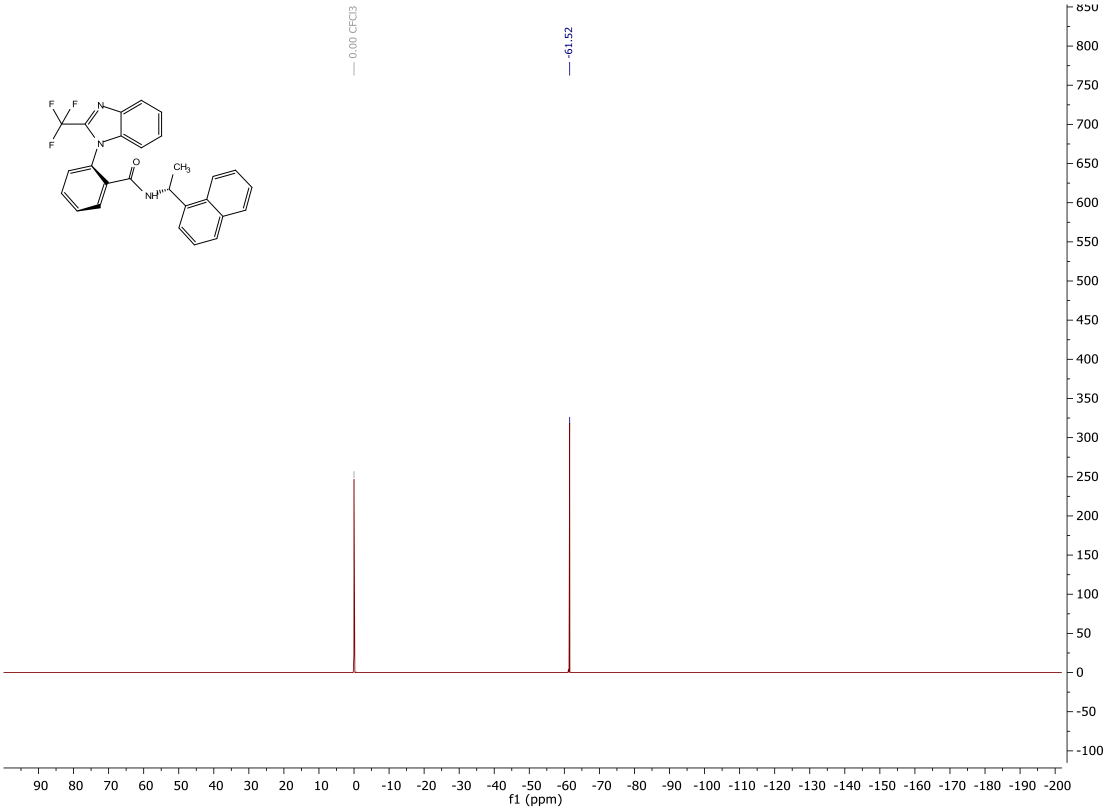

N-((*R*)-1-Phenylpropan-2-yl)-2-((*P*)-2-(trifluoromethyl)-1*H*-benzo[*d*]imidazol-1-yl)benzamide **(*P*)-29**
^{19}F NMR (376 MHz, $CDCl_3$)

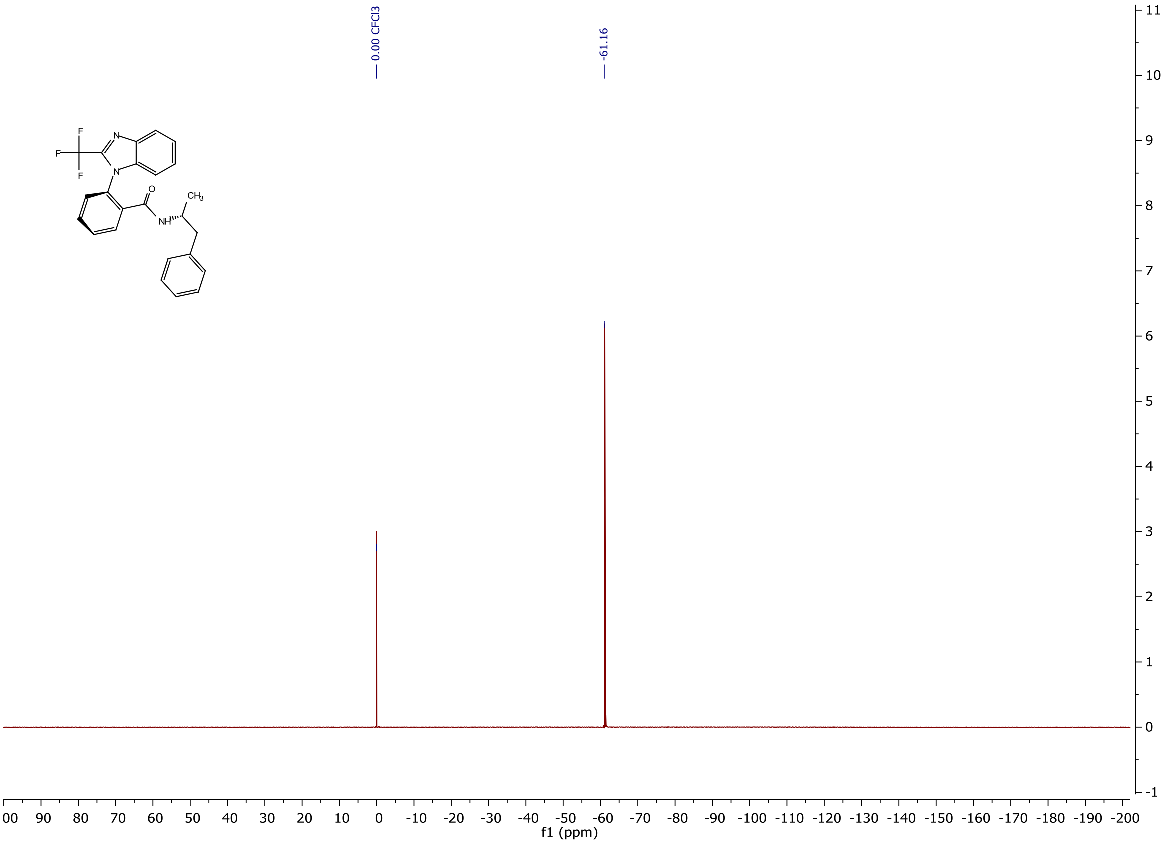

N-((*R*)-1-Phenylpropan-2-yl)-2-((*M*)-2-(trifluoromethyl)-1*H*-benzo[*d*]imidazol-1-yl)benzamide **(*M*)-29**
^{19}F NMR (376 MHz, $CDCl_3$)

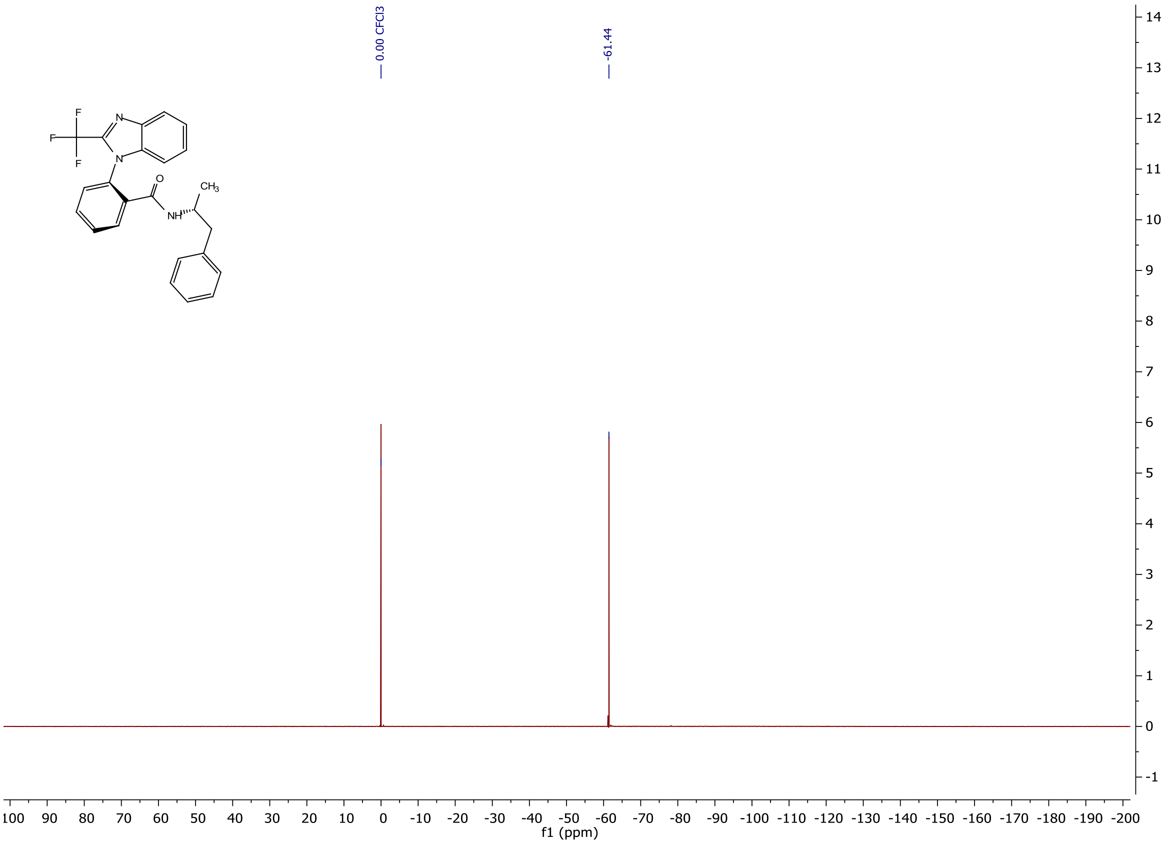

N-((*S*)-1-Hydroxy-3-phenylpropan-2-yl)-2-((*P*)-2-(trifluoromethyl)-1*H*-benzo[*d*]imidazol-1-yl)benzamide **(*P*)-30**

^{19}F NMR (376 MHz, $CDCl_3$)

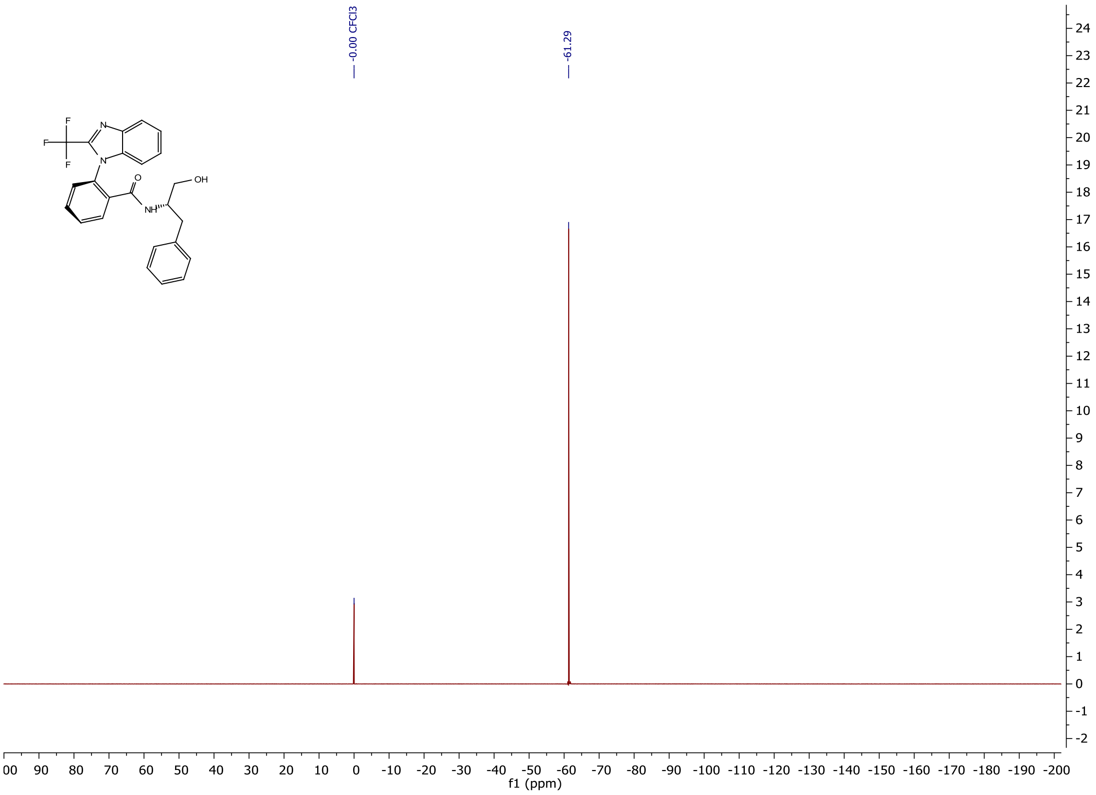

N-((*S*)-1-Hydroxy-3-phenylpropan-2-yl)-2-((*M*)-2-(trifluoromethyl)-1*H*-benzo[*d*]imidazol-1-yl)benzamide **(*M*)-30**

^{19}F NMR (376 MHz, $CDCl_3$)

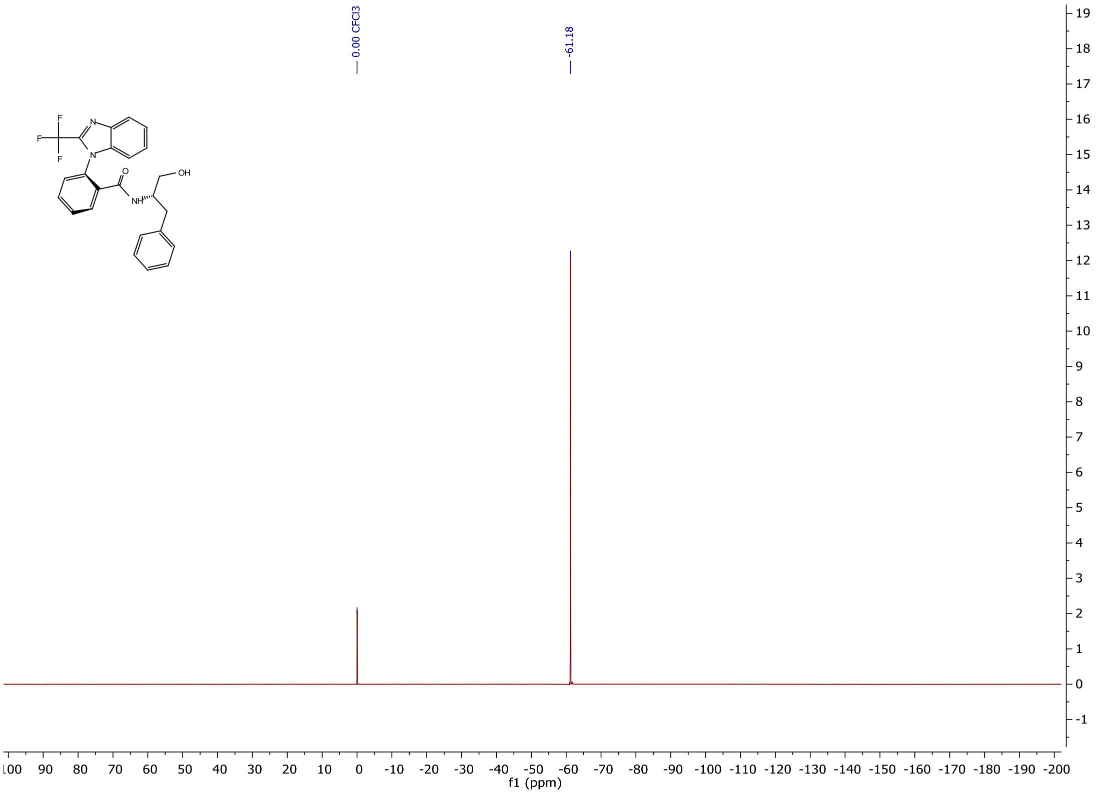

N-((*S*)-2-Hydroxy-1-phenylethyl)-2-((*P*)-2-(trifluoromethyl)-1*H*-benzo[*d*]imidazol-1-yl)benzamide **(*P*)-31**

^{19}F NMR (376 MHz, $CDCl_3$)

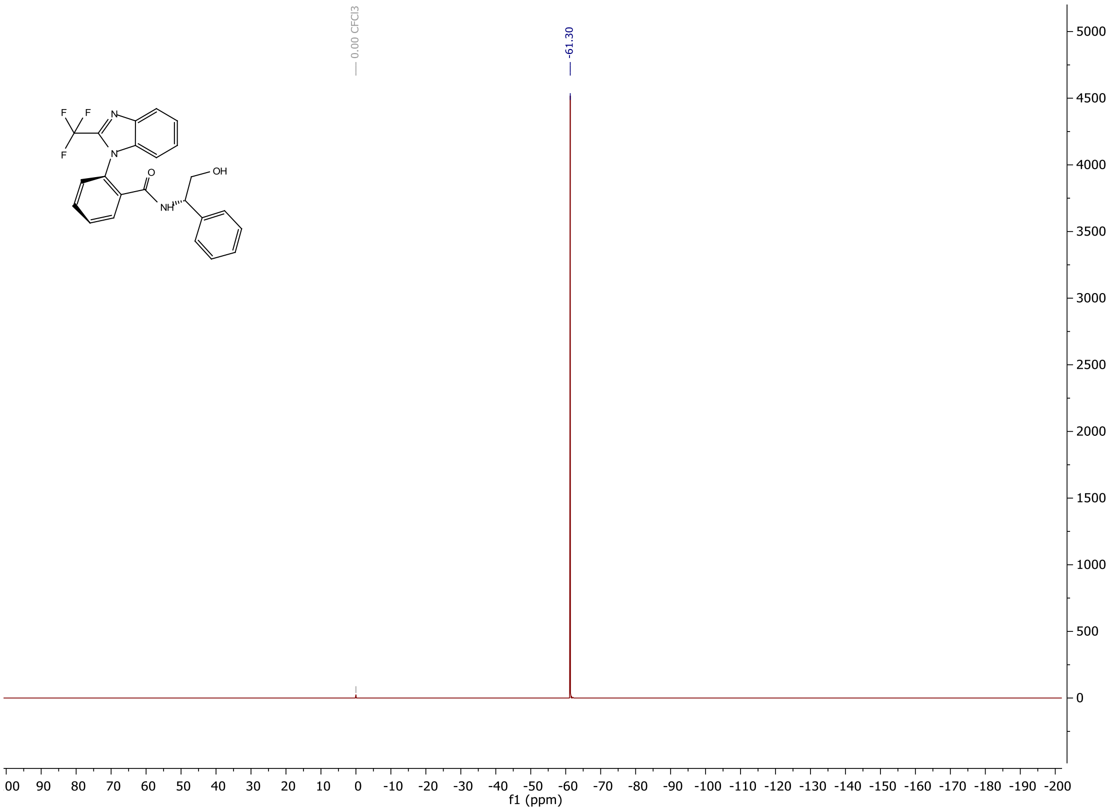

N-((*S*)-2-Hydroxy-1-phenylethyl)-2-((*M*)-2-(trifluoromethyl)-1*H*-benzo[*d*]imidazol-1-yl)benzamide **(*M*)-31**

^{19}F NMR (376 MHz, $CDCl_3$)

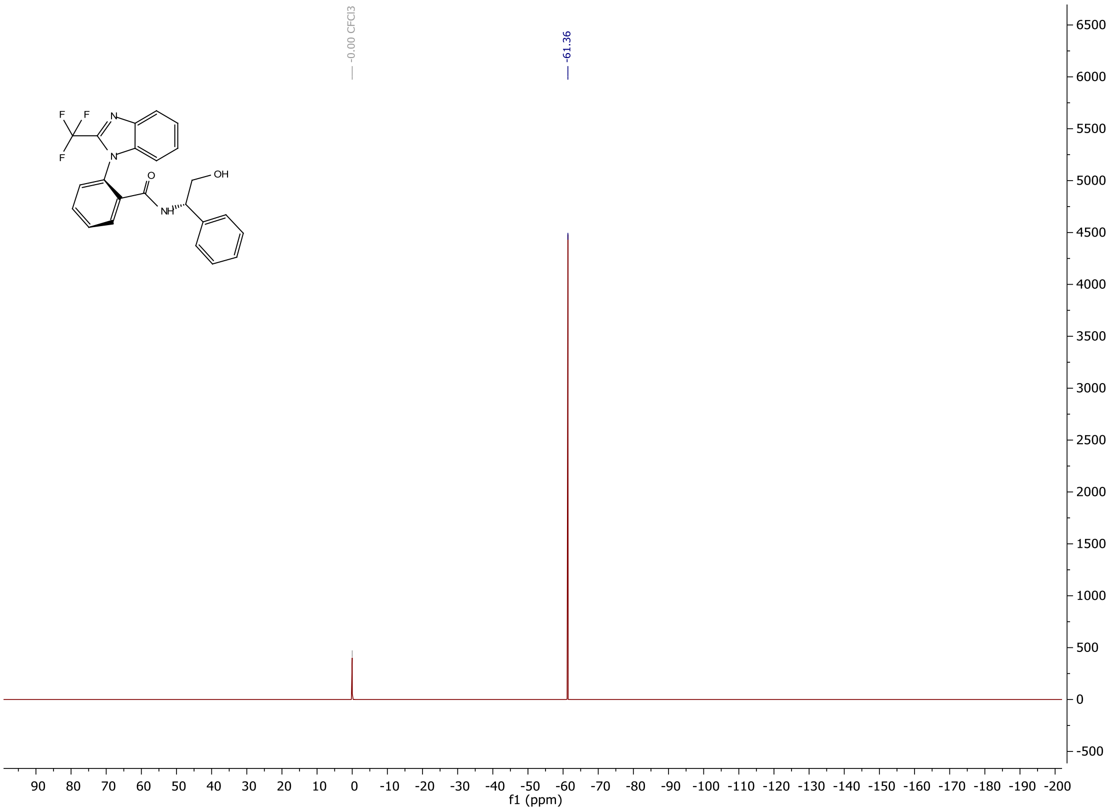

N-((*S*)-3-Hydroxy-1-phenylpropyl)-2-((*P*)-2-(trifluoromethyl)-1*H*-benzo[*d*]imidazol-1-yl)benzamide **(*P*)-32**

^{19}F NMR (376 MHz, $CDCl_3$)

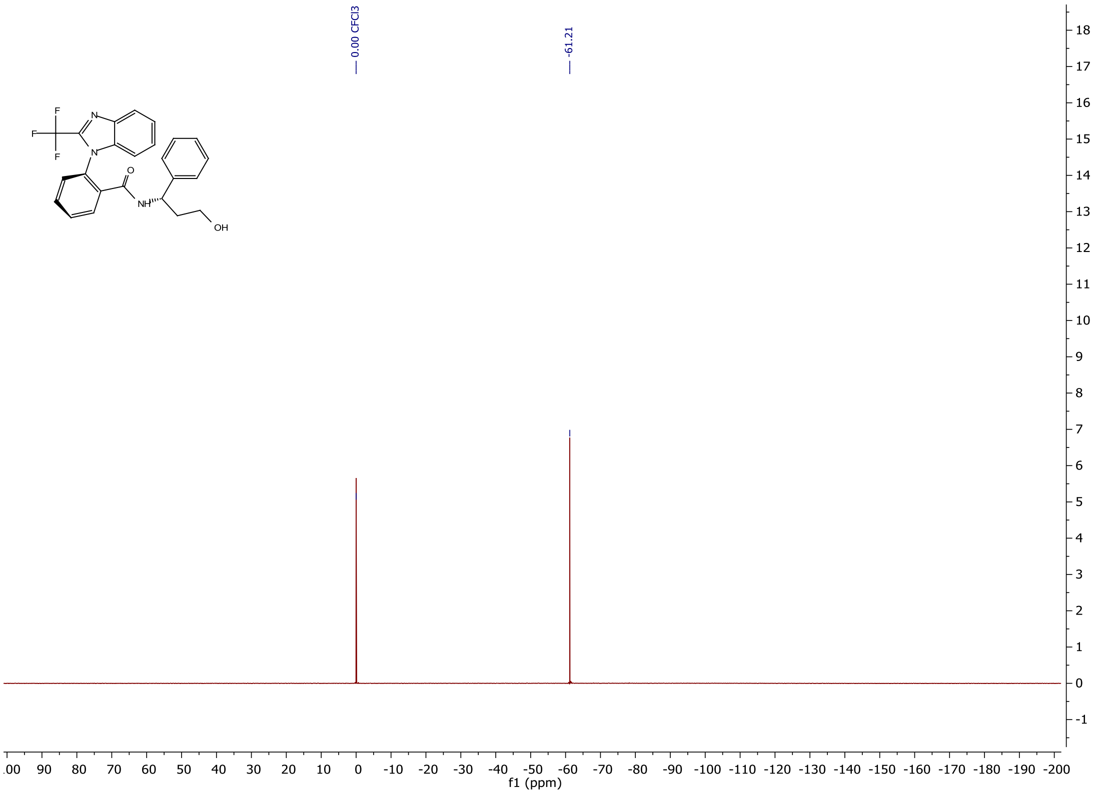

N-((*S*)-3-hydroxy-1-phenylpropyl)-2-((*M*)-2-(trifluoromethyl)-1*H*-benzo[*d*]imidazol-1-yl)benzamide **(*M*)-32**

^{19}F NMR (376 MHz, $CDCl_3$)

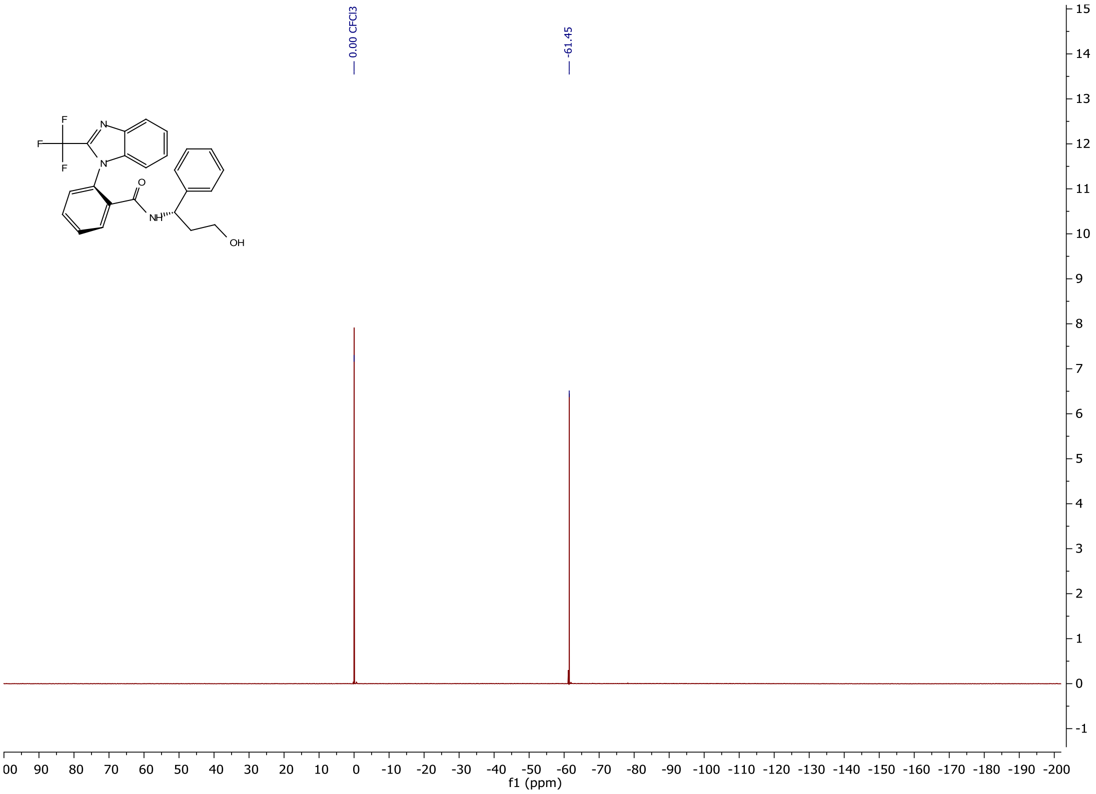

N-((*S*)-1-Hydroxy-3-methylbutan-2-yl)-2-((*P*)-2-(trifluoromethyl)-1*H*-benzo[*d*]imidazol-1-yl)benzamide **(*P*)-33**

^{19}F NMR (376 MHz, $CDCl_3$)

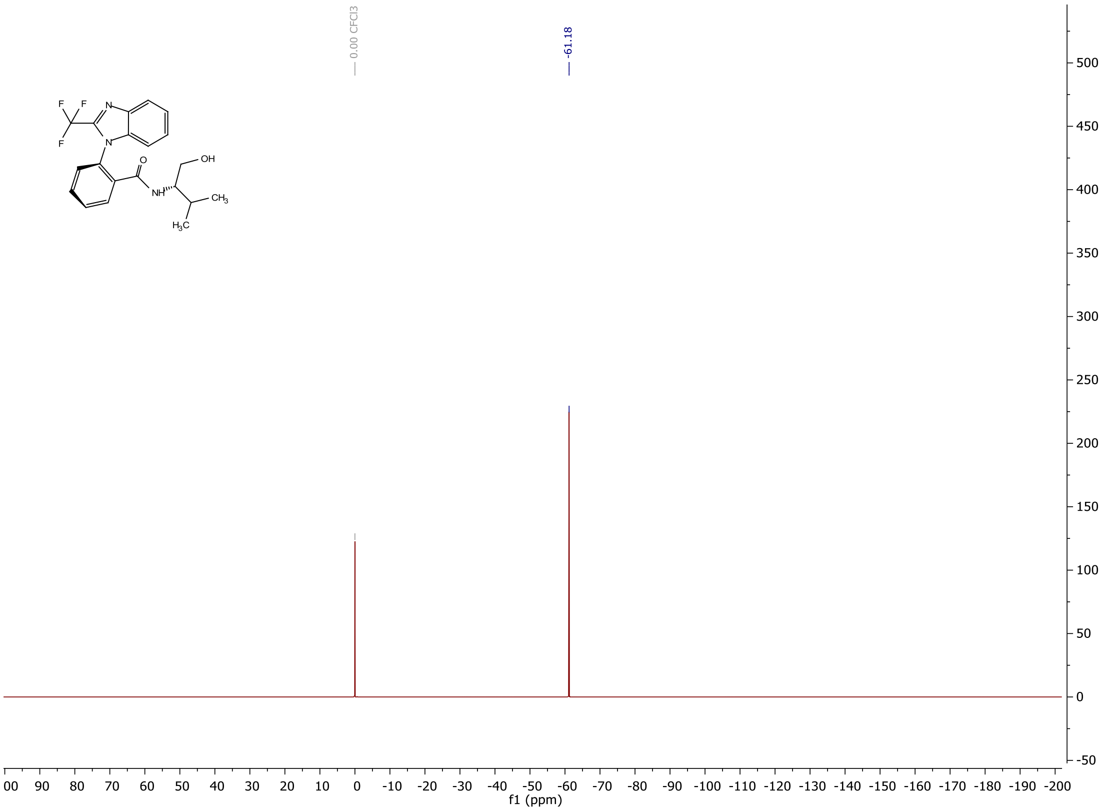

N-((*S*)-1-Hydroxy-3-methylbutan-2-yl)-2-((*M*)-2-(trifluoromethyl)-1*H*-benzo[*d*]imidazol-1-yl)benzamide **(*M*)-33**

^{19}F NMR (376 MHz, $CDCl_3$)

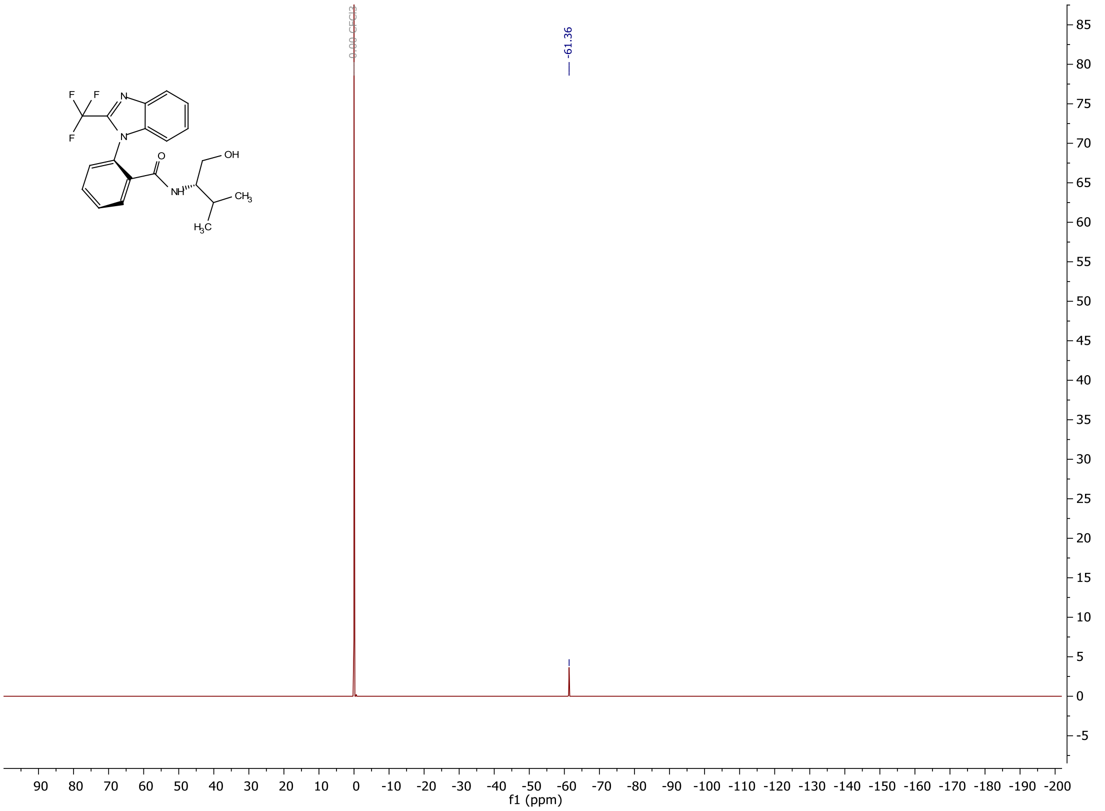

N-((*R*)-1-Hydroxybutan-2-yl)-2-((*P*)-2-(trifluoromethyl)-1*H*-benzo[*d*]imidazol-1-yl)benzamide **(*P*)-34**
^{19}F NMR (376 MHz, $CDCl_3$)

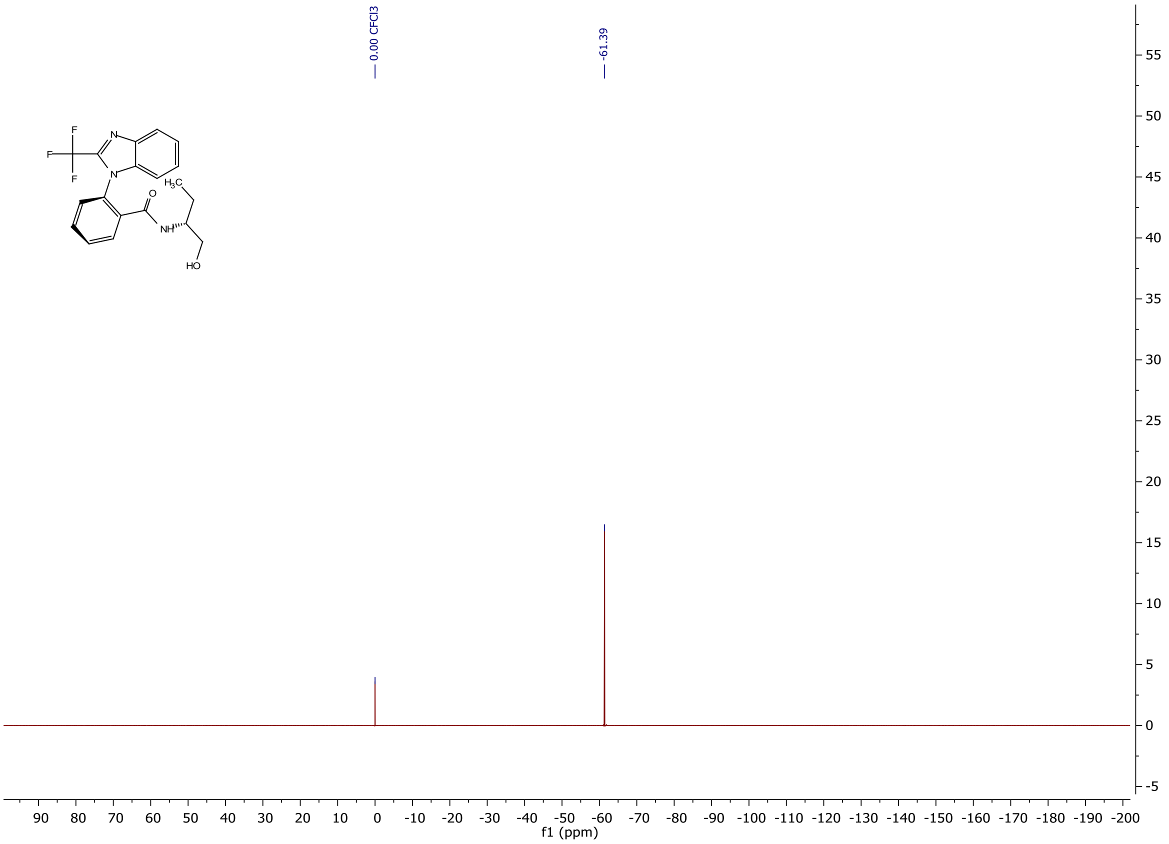

N-((*R*)-1-Hydroxybutan-2-yl)-2-((*M*)-2-(trifluoromethyl)-1*H*-benzo[*d*]imidazol-1-yl)benzamide **(*M*)-34**
^{19}F NMR (376 MHz, $CDCl_3$)

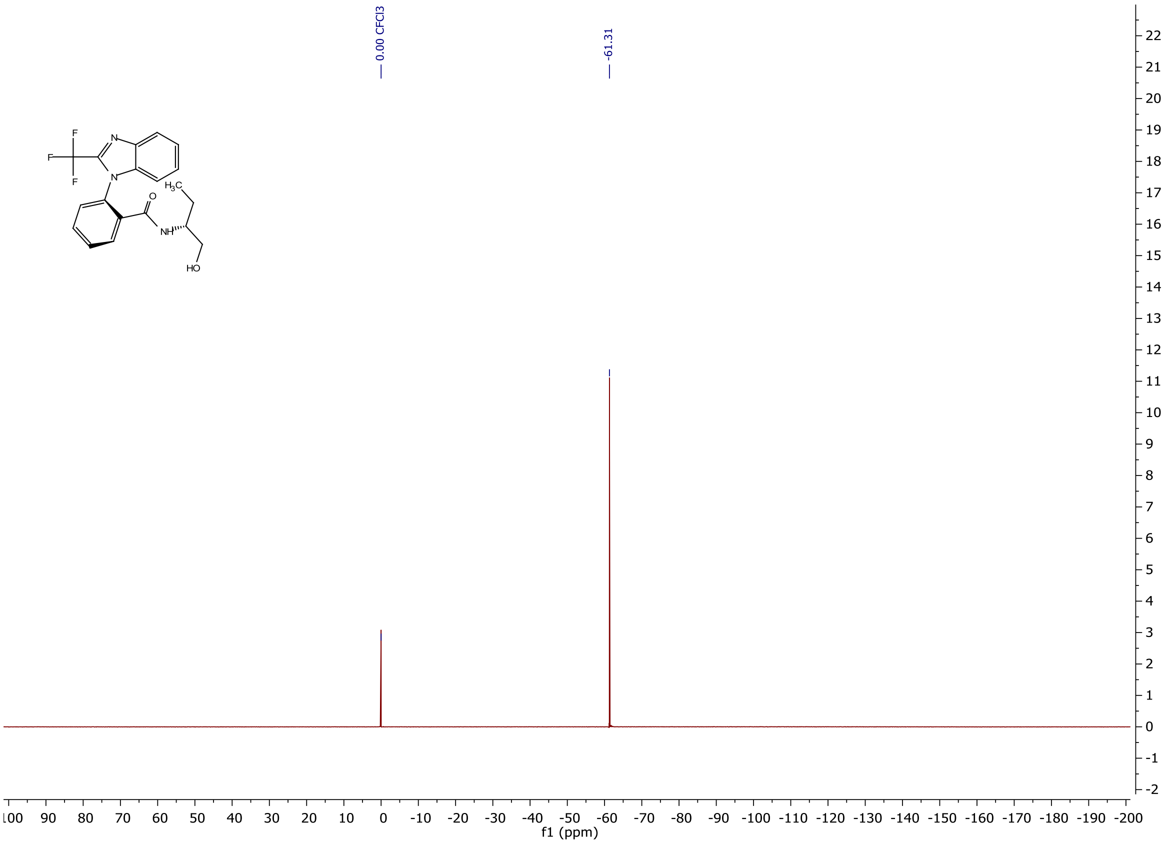

N-((*S*)-1-Hydroxypropan-2-yl)-2-((*P*)-2-(trifluoromethyl)-1*H*-benzo[*d*]imidazol-1-yl)benzamide **(*P*)-35**

^{19}F NMR (376 MHz, $CDCl_3$)

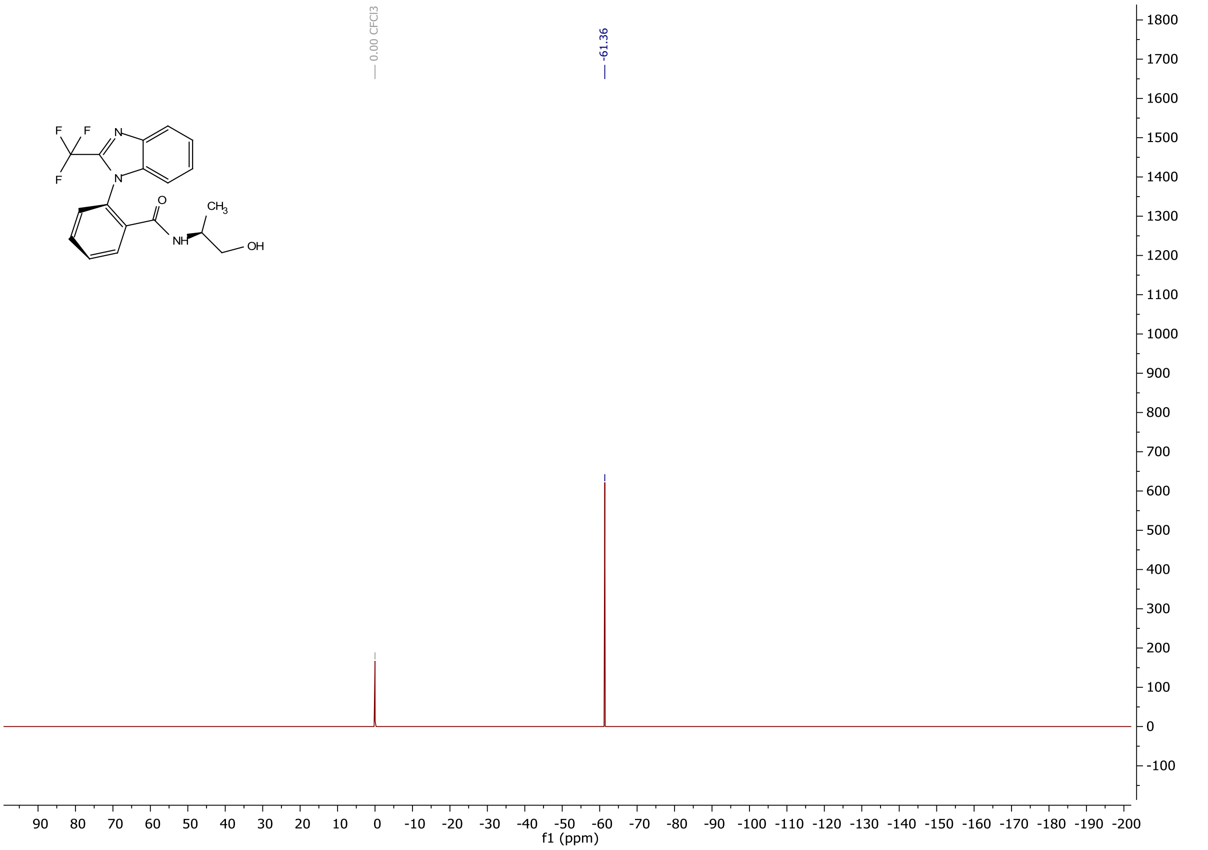

N-((*S*)-1-Hydroxypropan-2-yl)-2-((*M*)-2-(trifluoromethyl)-1*H*-benzo[*d*]imidazol-1-yl)benzamide **(*M*)-35**

^{19}F NMR (376 MHz, $CDCl_3$)

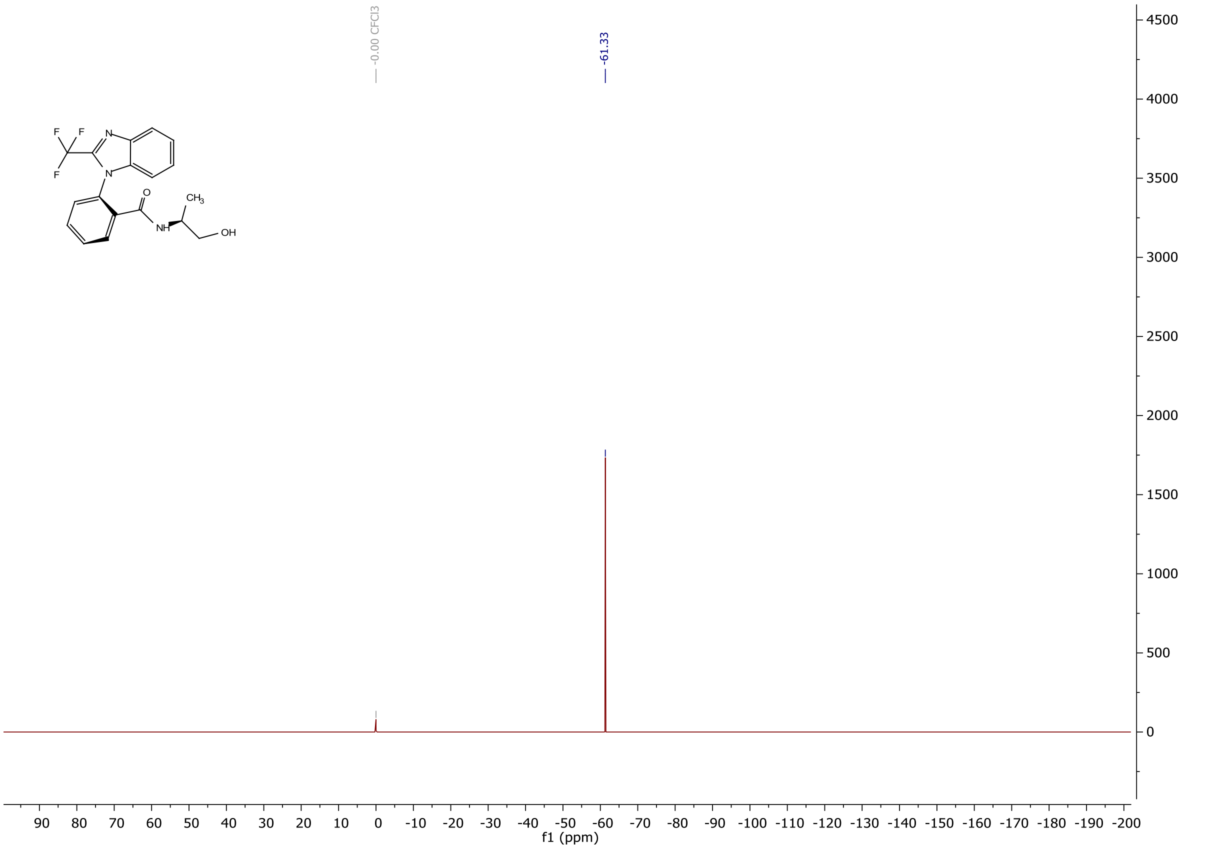

N-((*R*)-1-Methoxypropan-2-yl)-2-((*P*)-2-(trifluoromethyl)-1*H*-benzo[*d*]imidazol-1-yl)benzamide **(*P*)-36**

^{19}F NMR (376 MHz, $CDCl_3$)

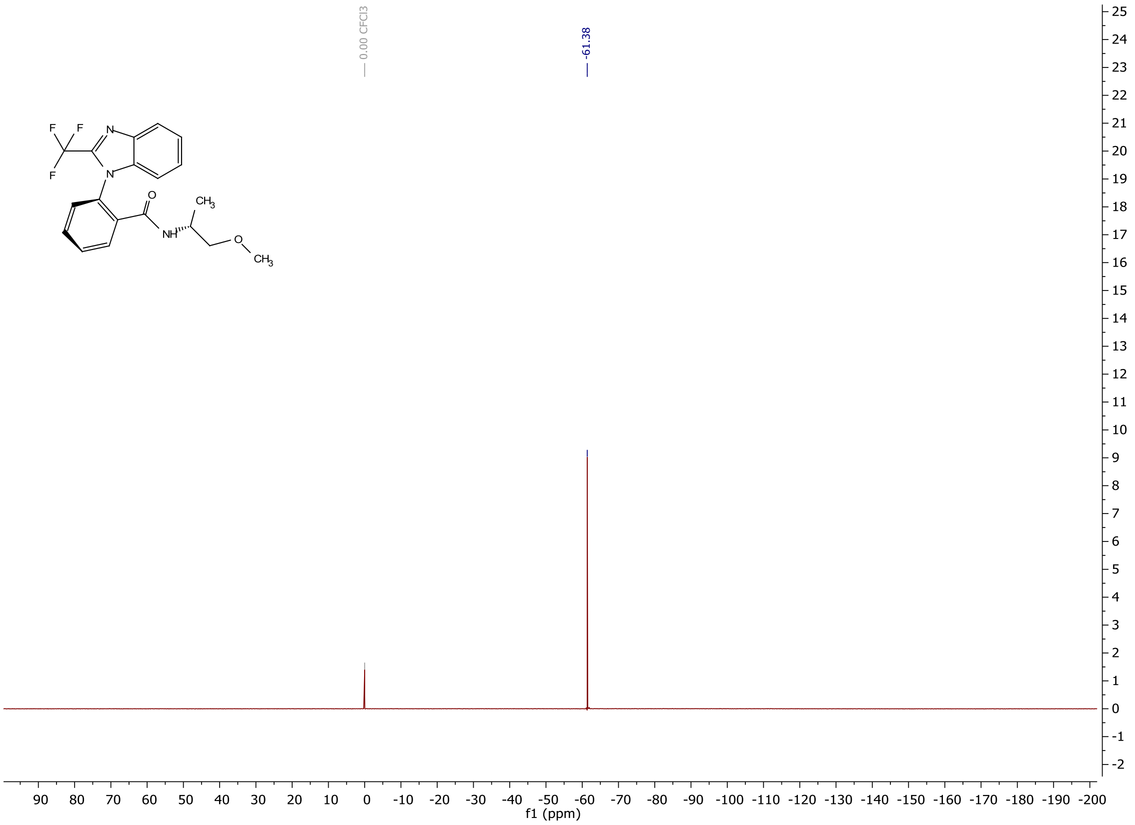

N-((*R*)-1-Methoxypropan-2-yl)-2-((*M*)-2-(trifluoromethyl)-1*H*-benzo[*d*]imidazol-1-yl)benzamide **(*M*)-36**

^{19}F NMR (376 MHz, $CDCl_3$)

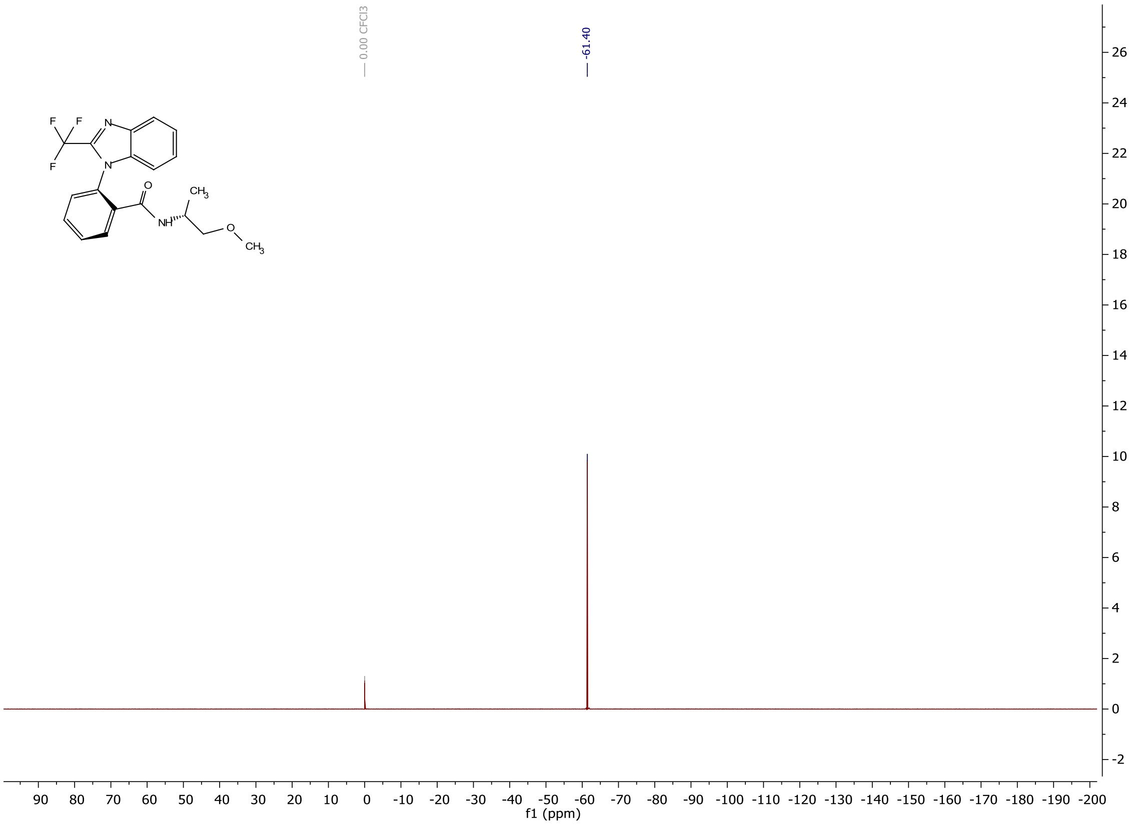

N-((1*R*,2*R*)-2-Aminocyclohexyl)-2-((*P*)-2-(trifluoromethyl)-1*H*-benzo[*d*]imidazol-1-yl)benzamide **(*P*)-37**

^{19}F NMR (376 MHz, $CDCl_3$)

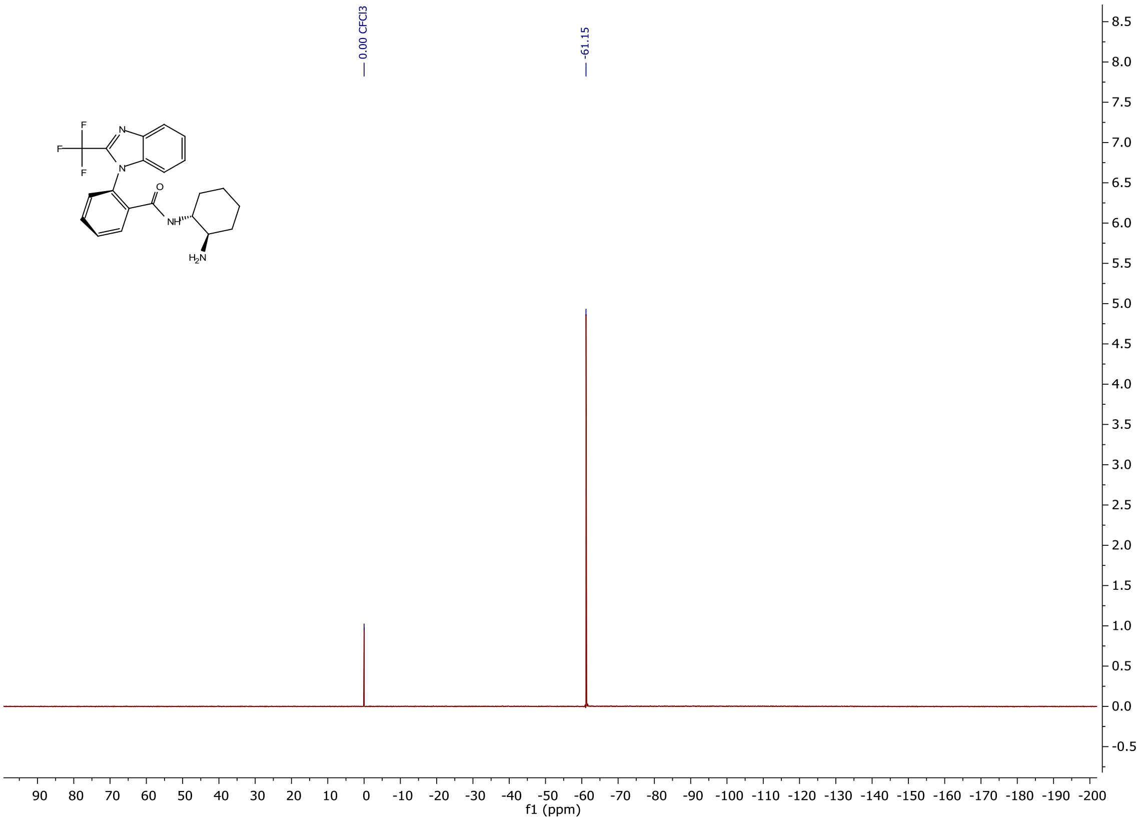

N-((1*R*,2*R*)-2-Aminocyclohexyl)-2-((*M*)-2-(trifluoromethyl)-1*H*-benzo[*d*]imidazol-1-yl)benzamide **(*M*)-37**

^{19}F NMR (376 MHz, $CDCl_3$)

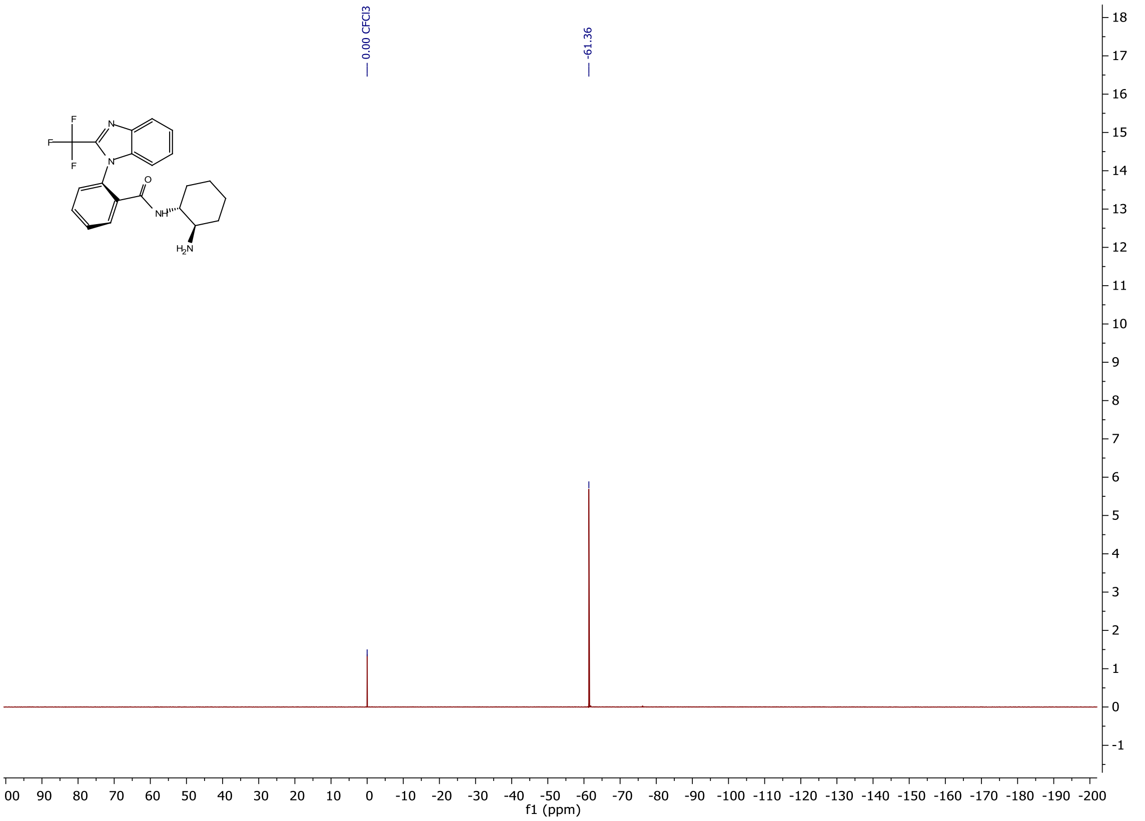

tert-Butyl ((1*R*,2*R*)-2-(2-((*P*)-2-(trifluoromethyl)-1*H*-benzo[*d*]imidazol-1-yl)benzamido)cyclohexyl)carbamate **(*P*)-38**

^{19}F NMR (376 MHz, $CDCl_3$)

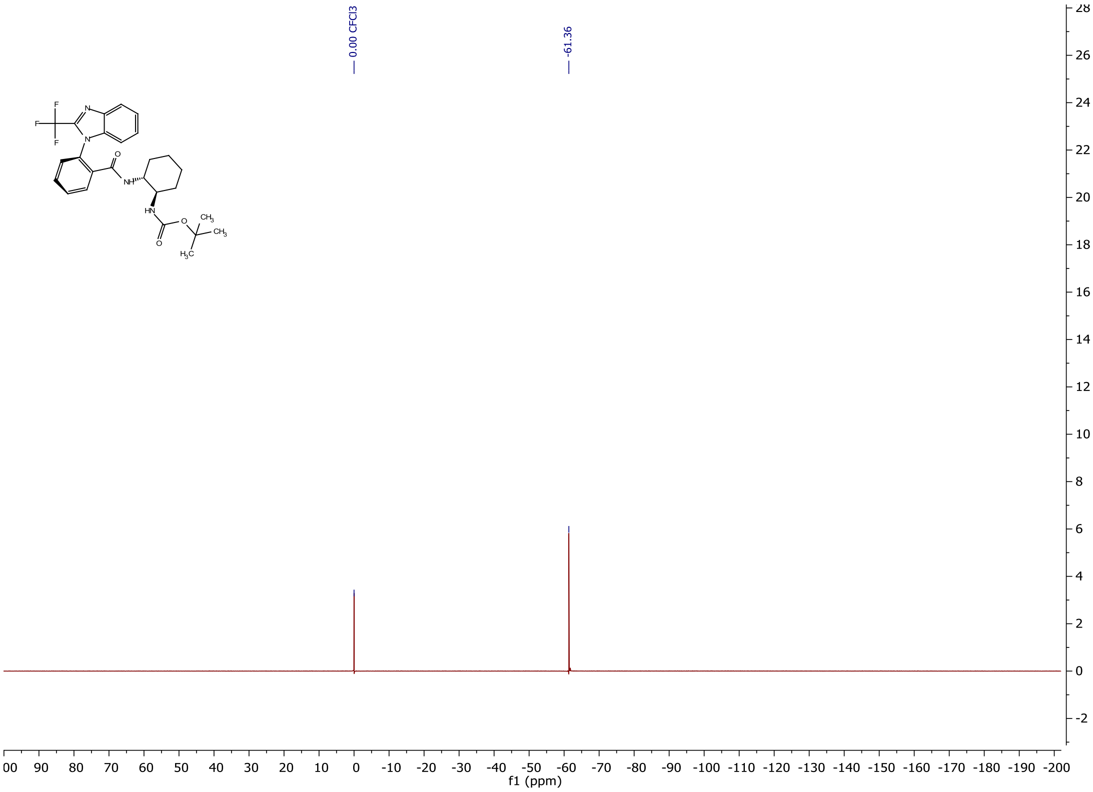

tert-Butyl ((1*R*,2*R*)-2-(2-((*M*)-2-(trifluoromethyl)-1*H*-benzo[*d*]imidazol-1-yl)benzamido)cyclohexyl)carbamate **(*M*)-38**

^{19}F NMR (376 MHz, $CDCl_3$)

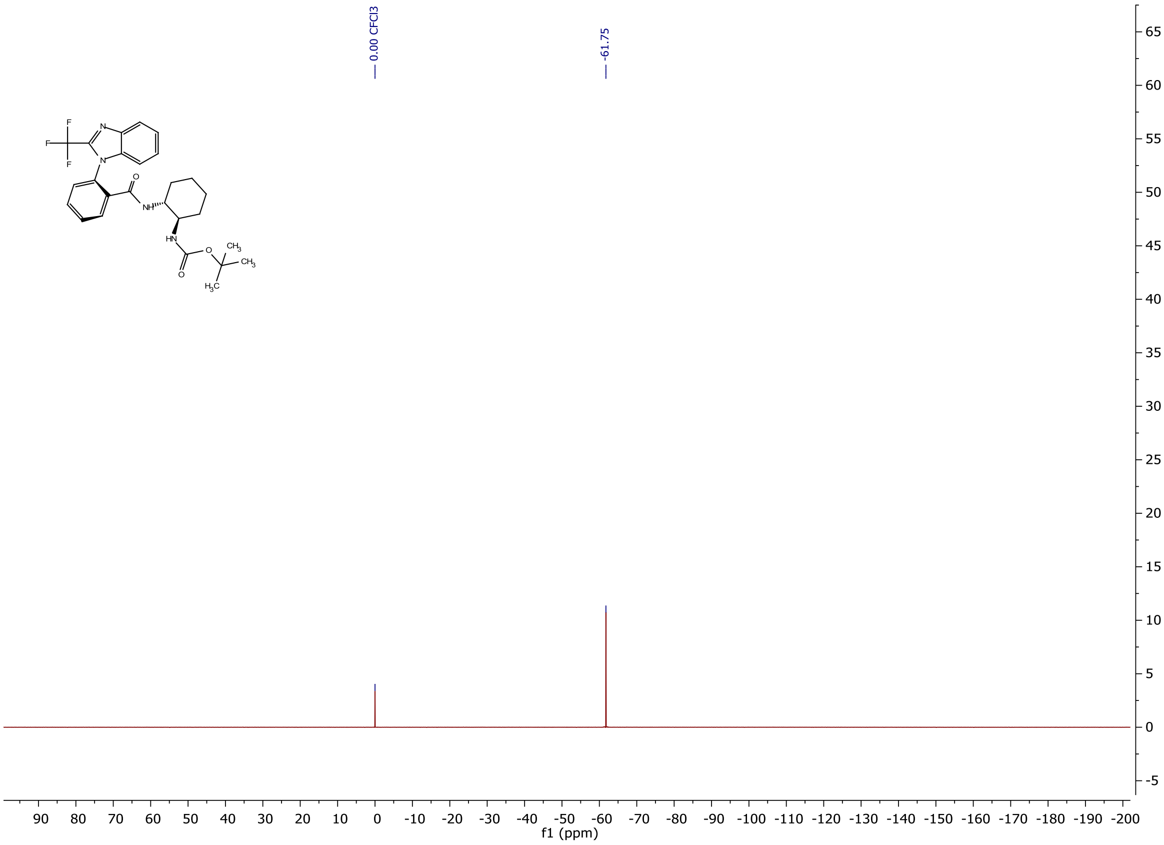

tert-Butyl (*R*)-3-(2-((*P*)-2-(trifluoromethyl)-1*H*-benzo[*d*]imidazol-1-yl)benzamido)piperidine-1-carboxylate **(*P*)-39**

^{19}F NMR (376 MHz, $CDCl_3$)

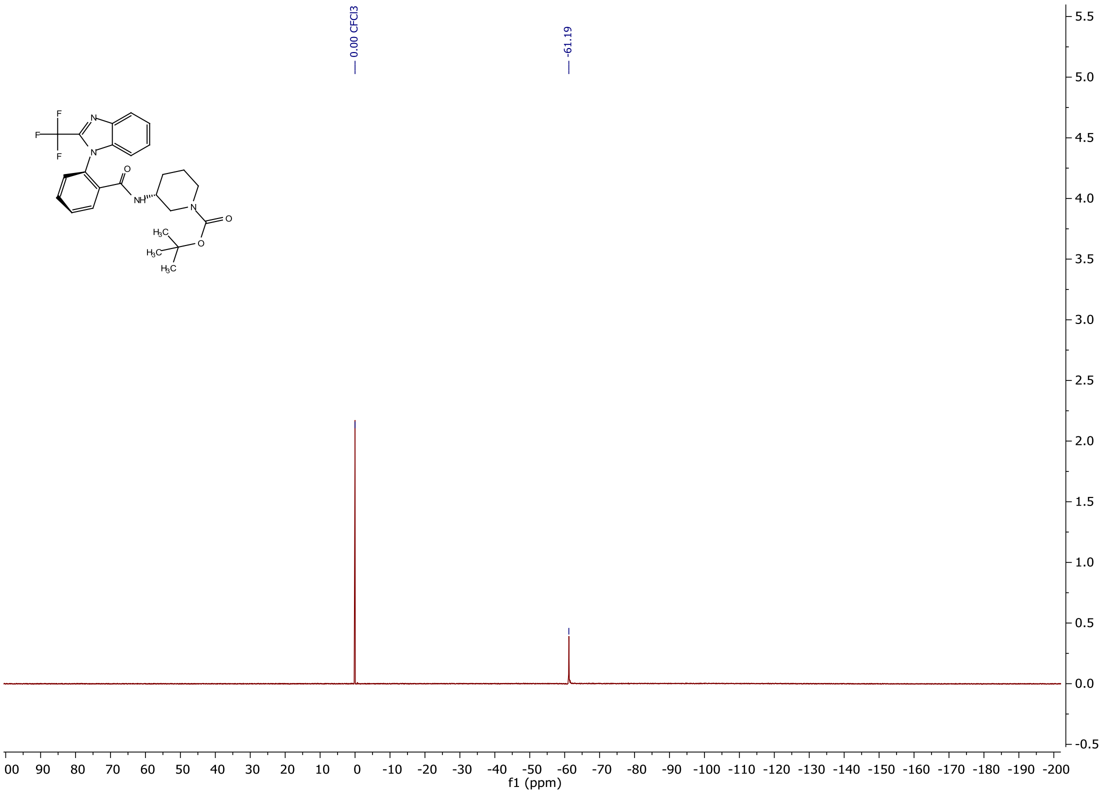

tert-Butyl (*R*)-3-(2-((*M*)-2-(trifluoromethyl)-1*H*-benzo[*d*]imidazol-1-yl)benzamido)piperidine-1-carboxylate **(*M*)-39**

^{19}F NMR (376 MHz, $CDCl_3$)

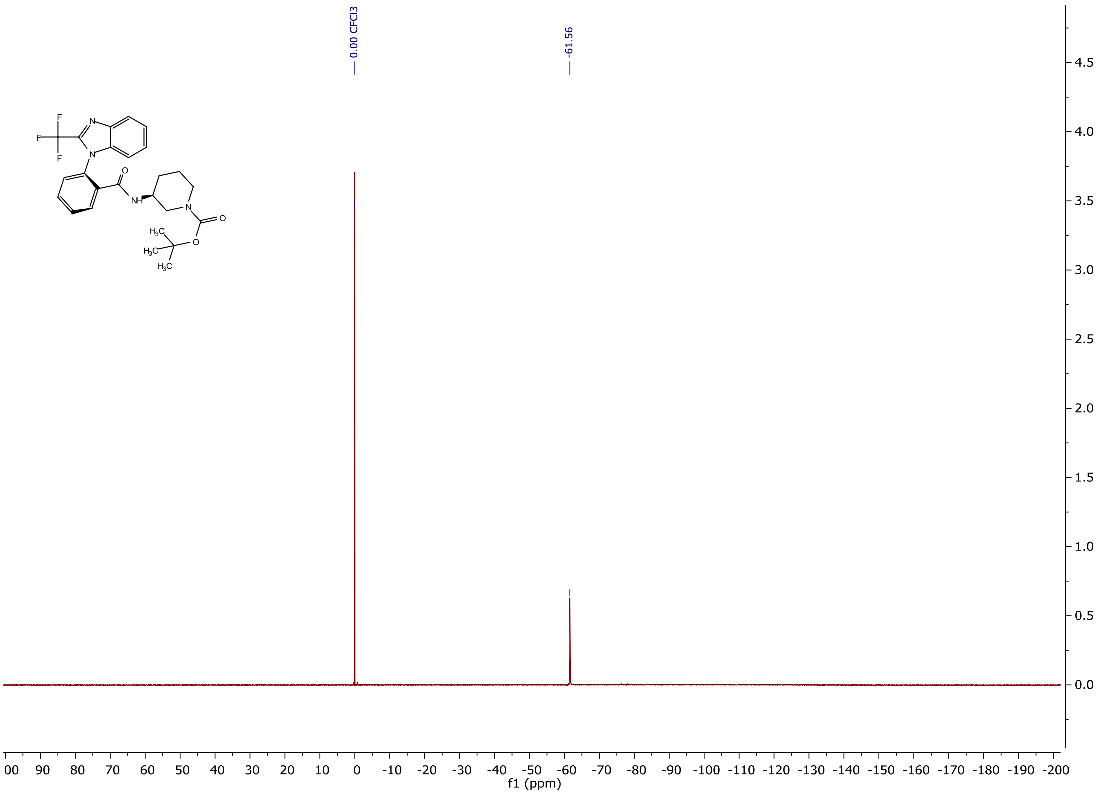

N-((1*R*,2*S*)-2-Hydroxy-2,3-dihydro-1*H*-inden-1-yl)-2-((*P*)-2-(trifluoromethyl)-1*H*-benzo[*d*]imidazol-1-yl)benzamide **(*P*)-40**

^{19}F NMR (376 MHz, $CDCl_3$)

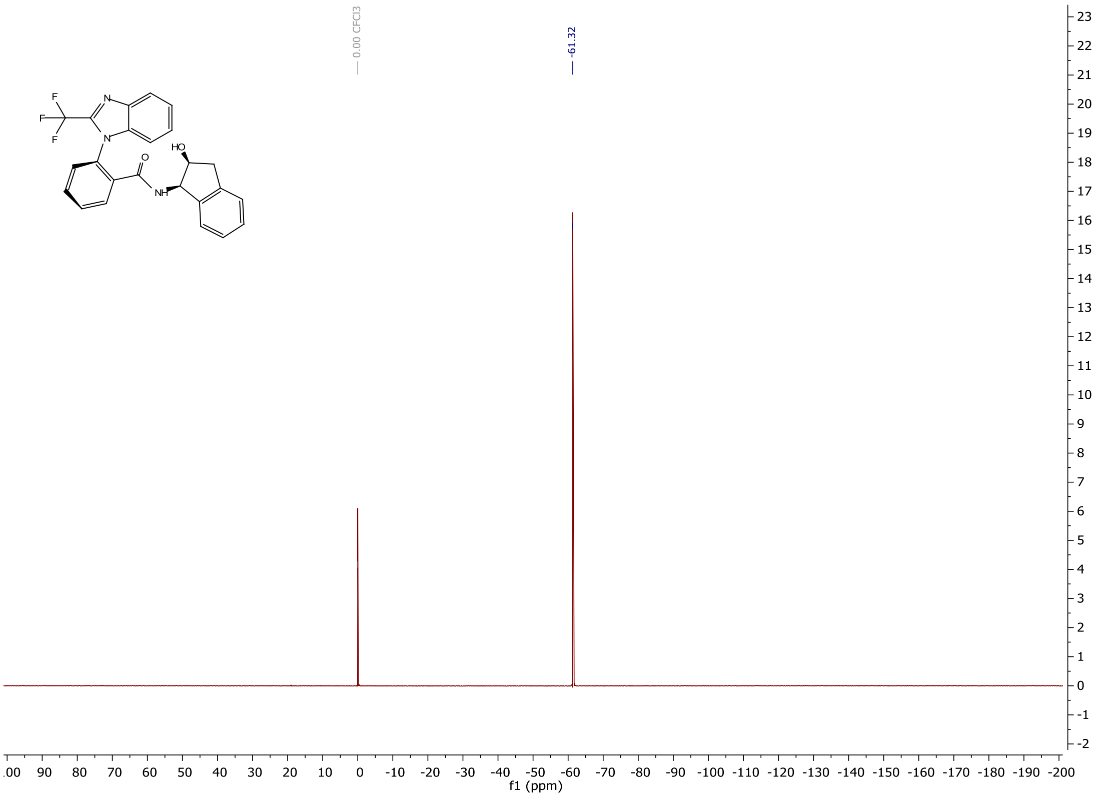

^{19}F NMR (376 MHz, DMSO-d_6)

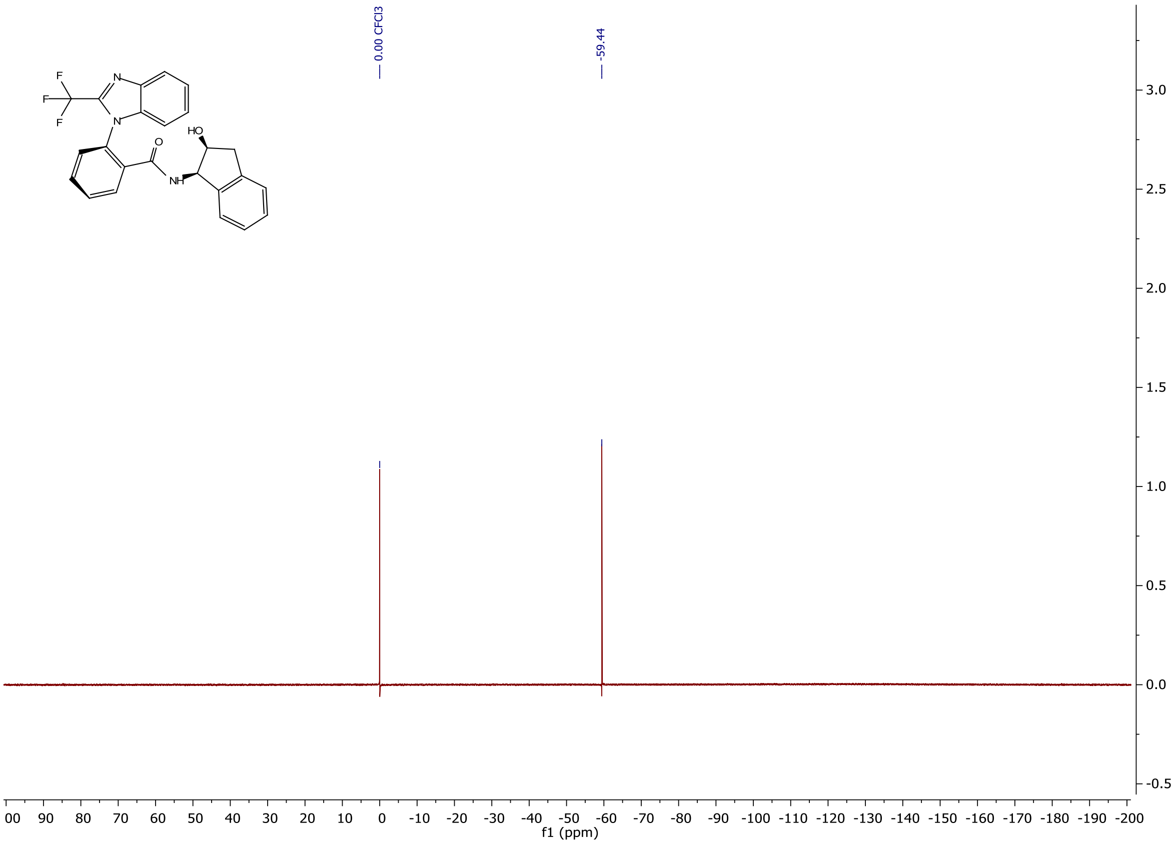

N-((1*R*,2*S*)-2-Hydroxy-2,3-dihydro-1*H*-inden-1-yl)-2-((*M*)-2-(trifluoromethyl)-1*H*-benzo[*d*]imidazol-1-yl)benzamide **(*M*)-40**

^{19}F NMR (376 MHz, $CDCl_3$)

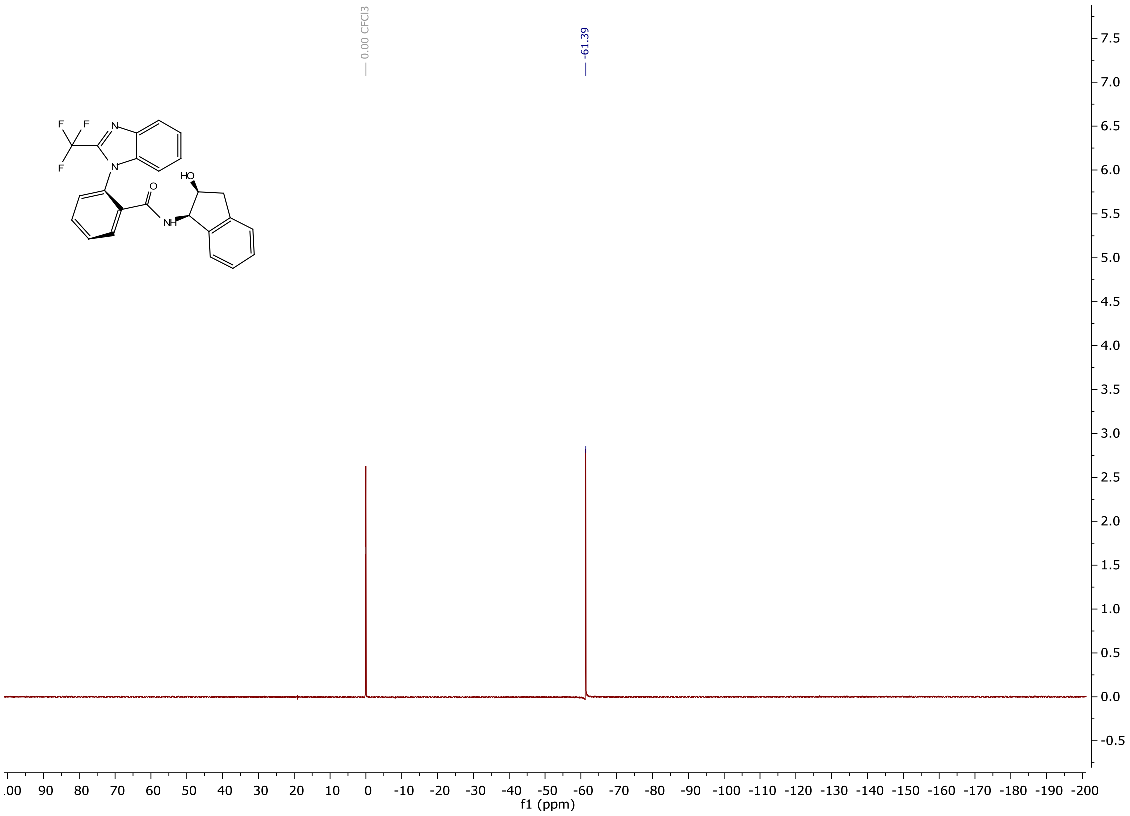

^{19}F NMR (376 MHz, DMSO-d_6)

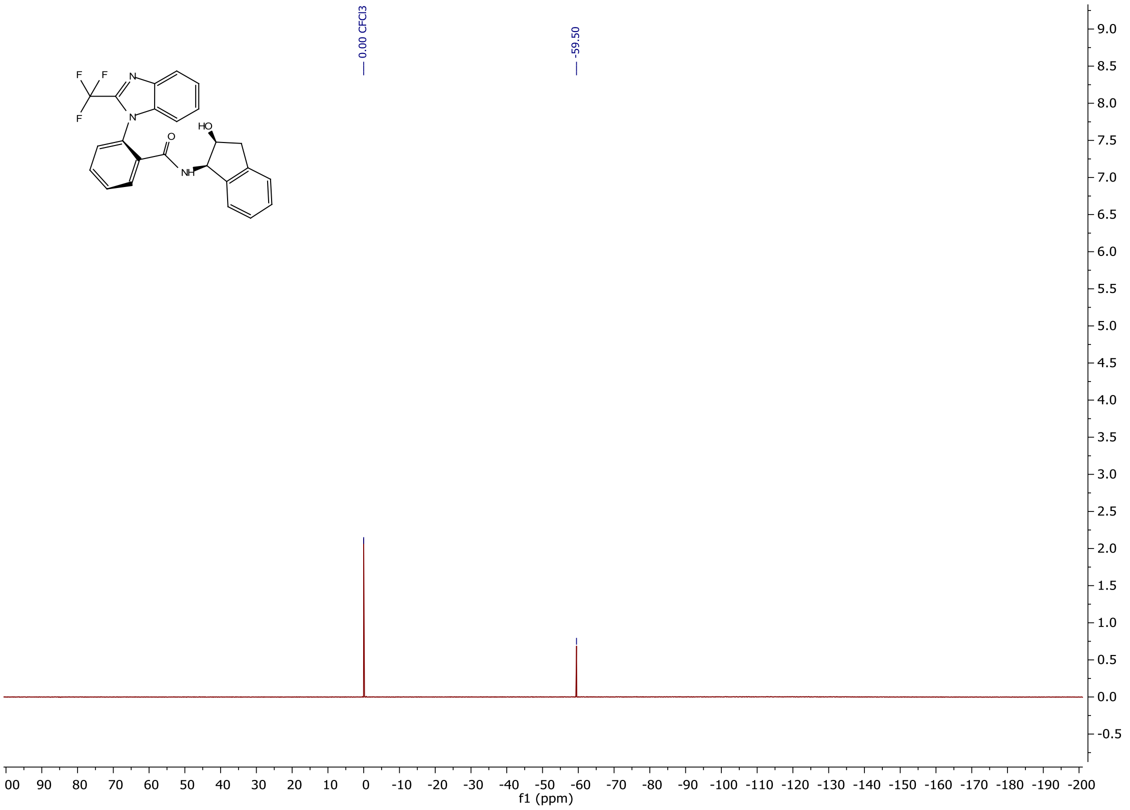

(1*R*,2*S*)-1-(2-((*P*)-2-(trifluoromethyl)-1*H*-benzo[*d*]imidazol-1-yl)benzamido)-2,3-dihydro-1*H*-inden-2-yl acetate **(*P*)-41**

^{19}F NMR (376 MHz, $CDCl_3$)

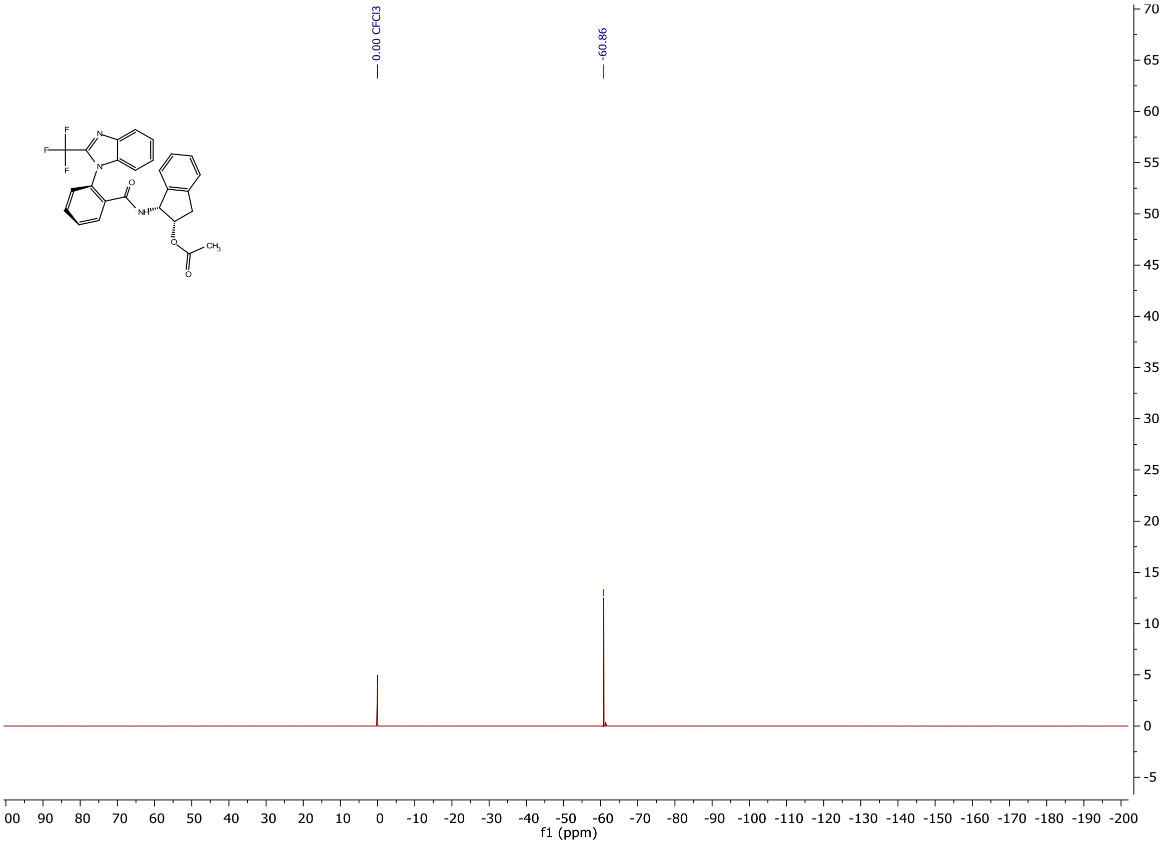

^{19}F NMR (376 MHz, DMSO-d_6)

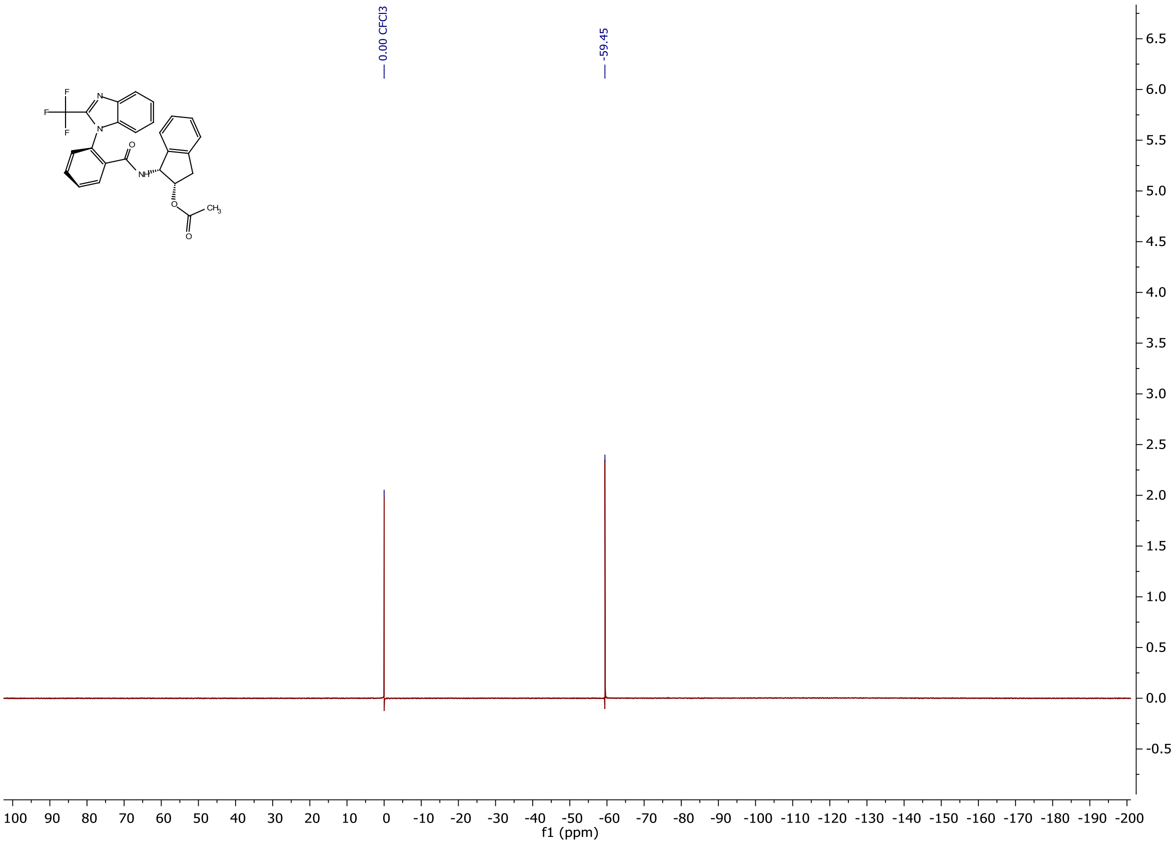

(1*R*,2*S*)-1-(2-((*M*)-2-(trifluoromethyl)-1*H*-benzo[*d*]imidazol-1-yl)benzamido)-2,3-dihydro-1*H*-inden-2-yl acetate **(*M*)-41**

^{19}**F NMR** (376 MHz, $CDCl_3$)

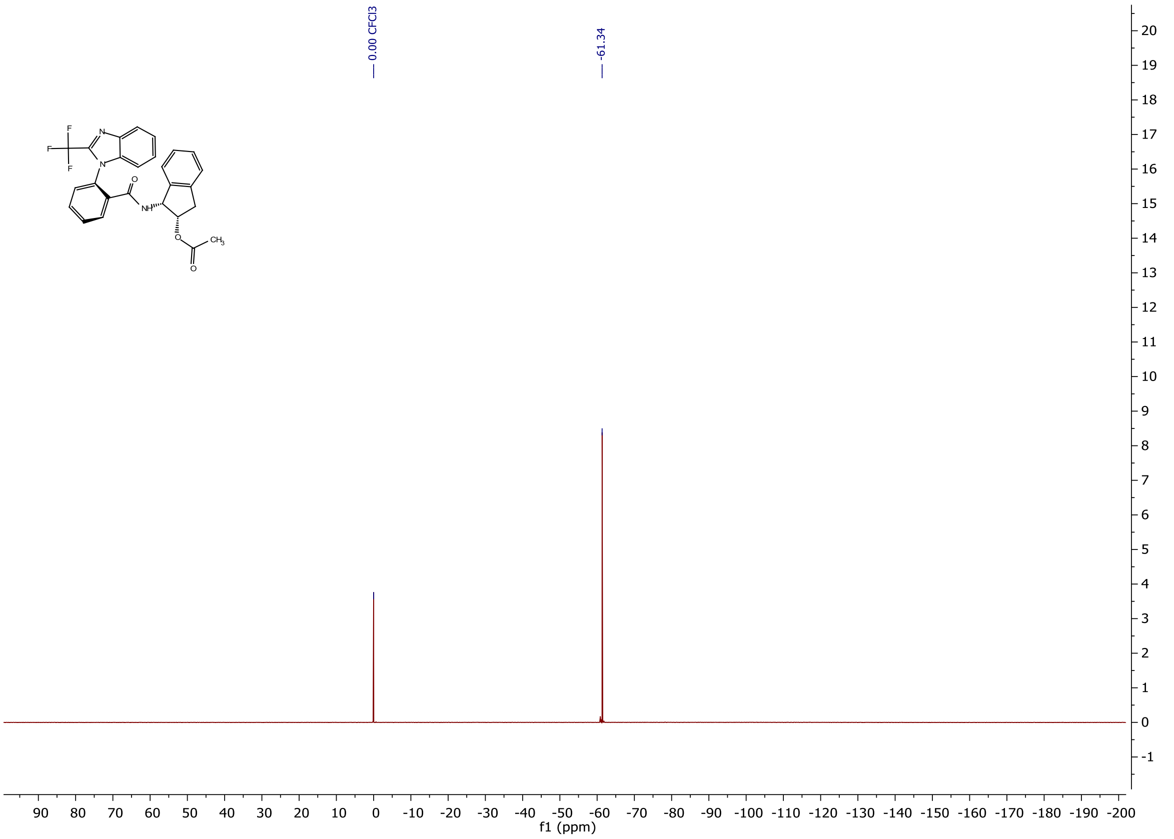

^{19}**F NMR** (376 MHz, DMSO-d_6)

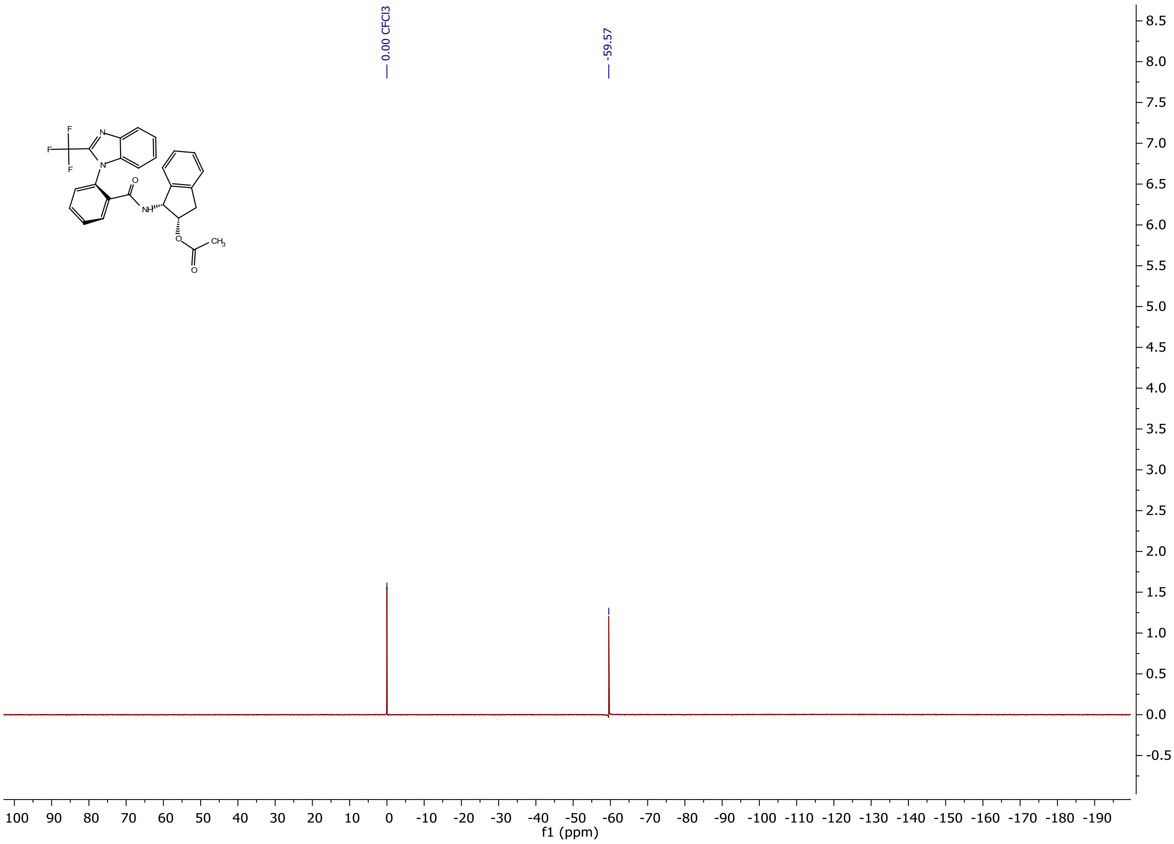

N-((*S*)-1-Hydroxy-4-methylpentan-2-yl)-2-((*P*)-2-(trifluoromethyl)-1*H*-benzo[*d*]imidazol-1-yl)benzamide **(*P*)-42**

^{19}F NMR (376 MHz, $CDCl_3$)

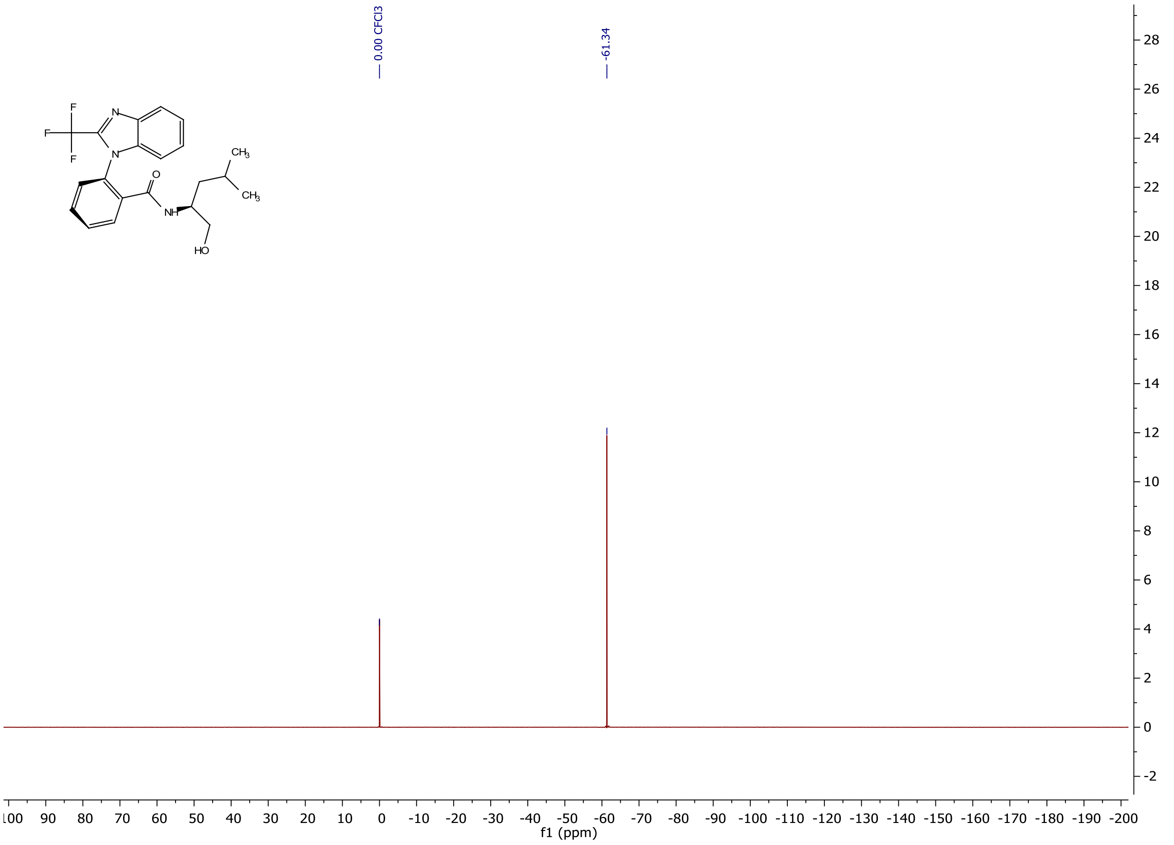

^{19}F NMR (376 MHz, DMSO-d_6)

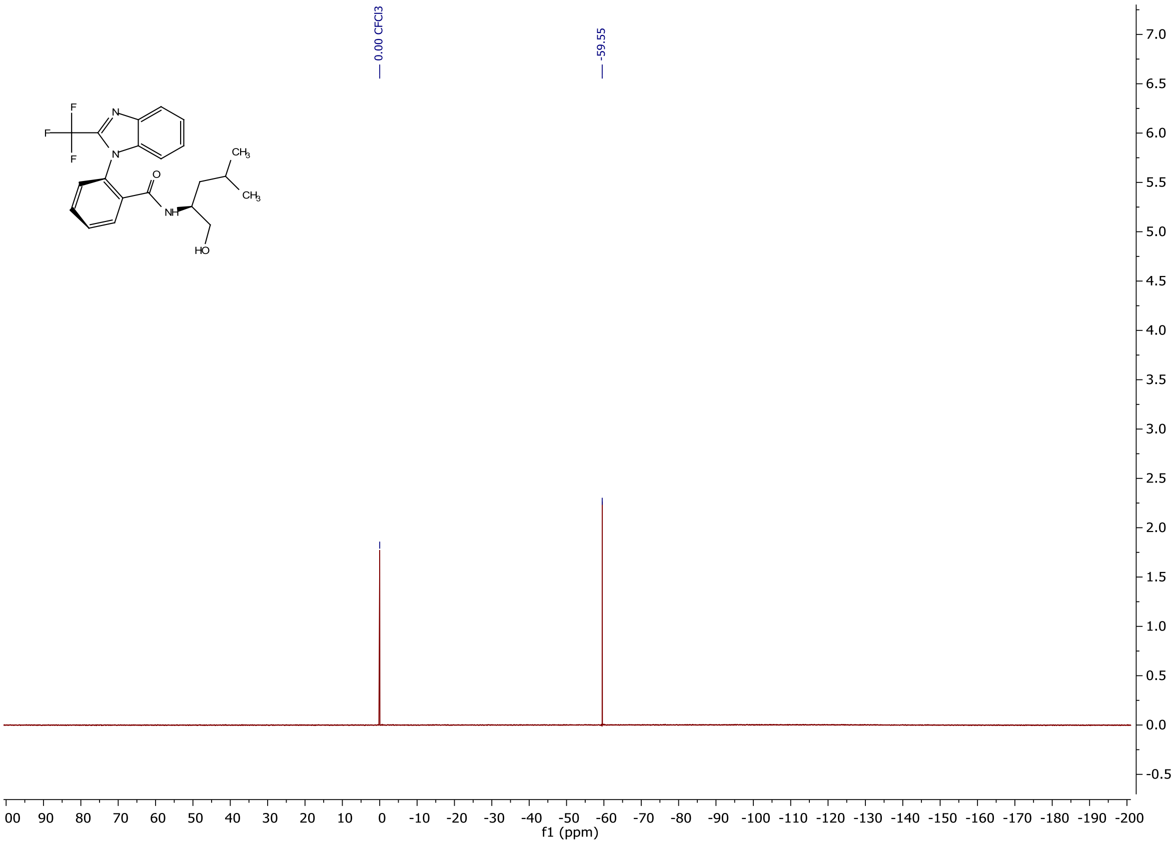

N-((*S*)-1-Hydroxy-4-methylpentan-2-yl)-2-((*M*)-2-(trifluoromethyl)-1*H*-benzo[*d*]imidazol-1-yl)benzamide **(*M*)-42**

^{19}F NMR (376 MHz, $CDCl_3$)

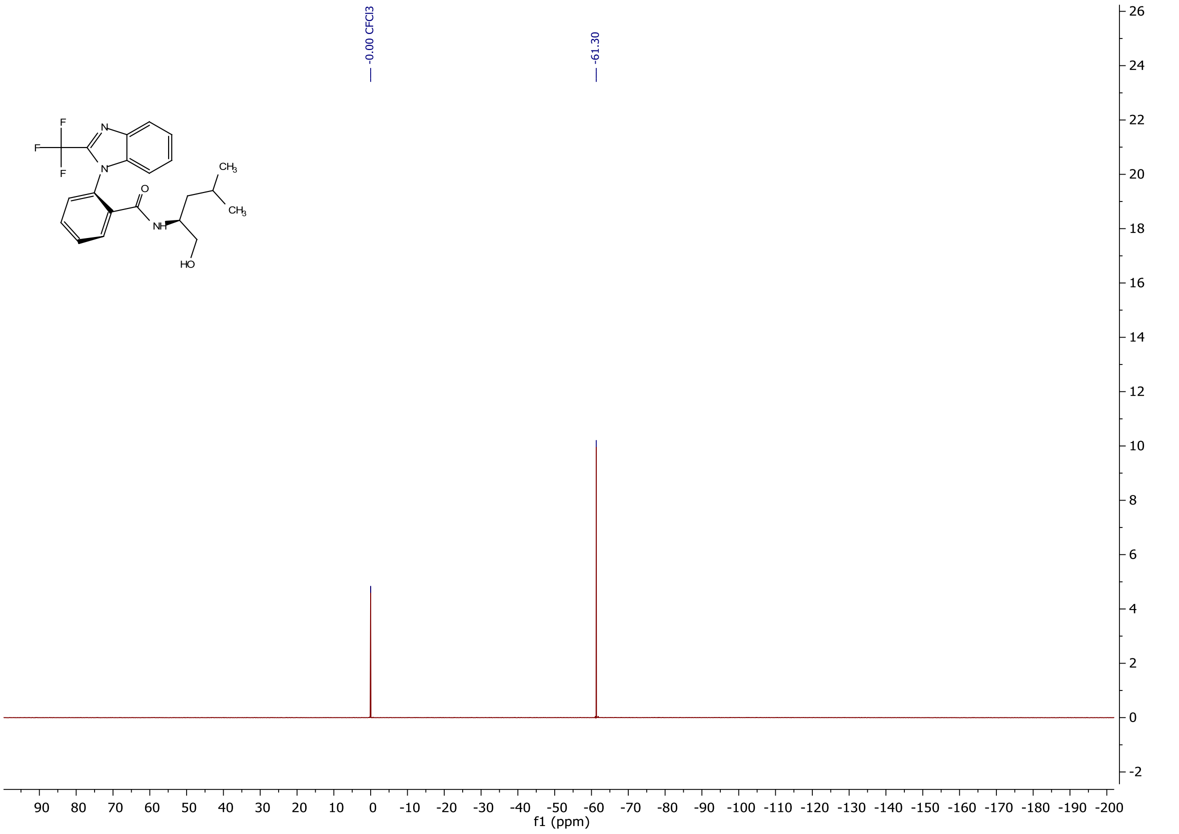

^{19}F NMR (376 MHz, DMSO-d_6)

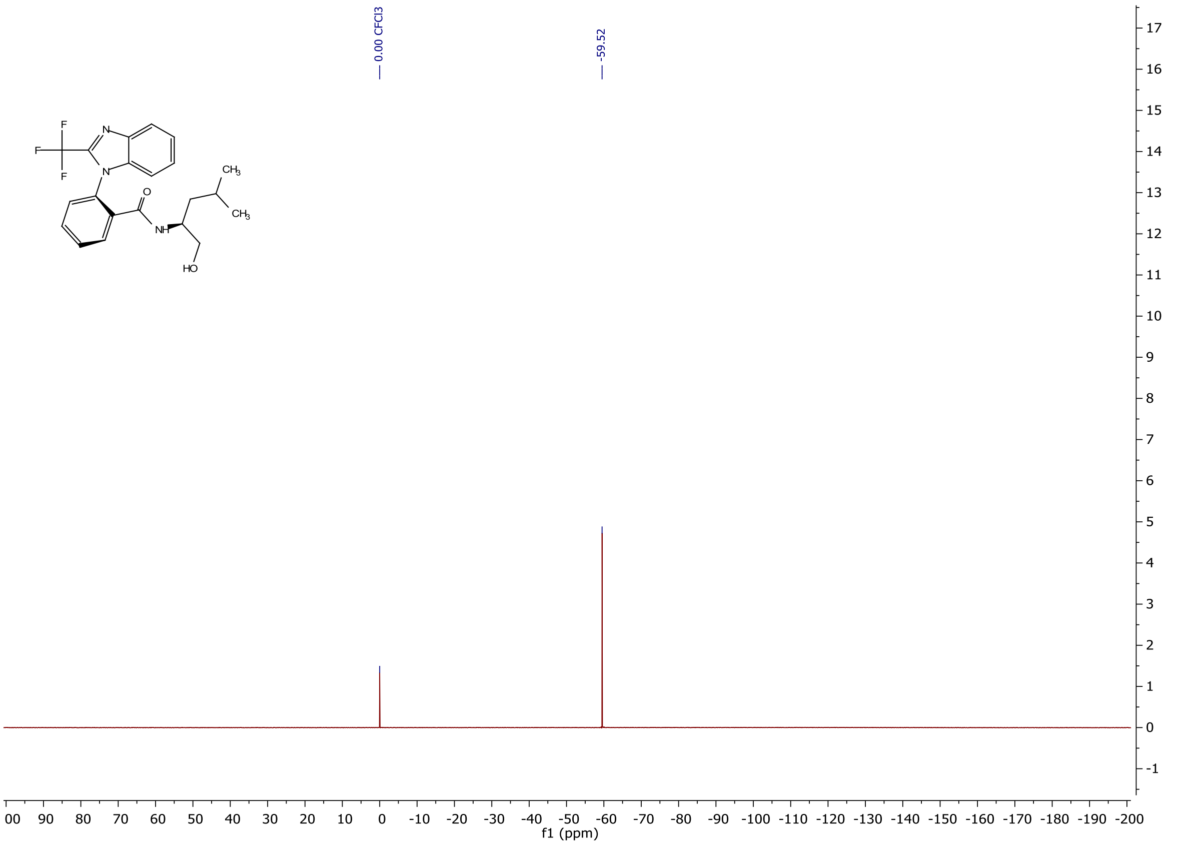

(*S*)-4-Methyl-2-(2-((*P*)-2-(trifluoromethyl)-1*H*-benzo[*d*]imidazol-1-yl)benzamido)pentyl acetate **(*P*)-43**

^{19}F NMR (376 MHz, $CDCl_3$)

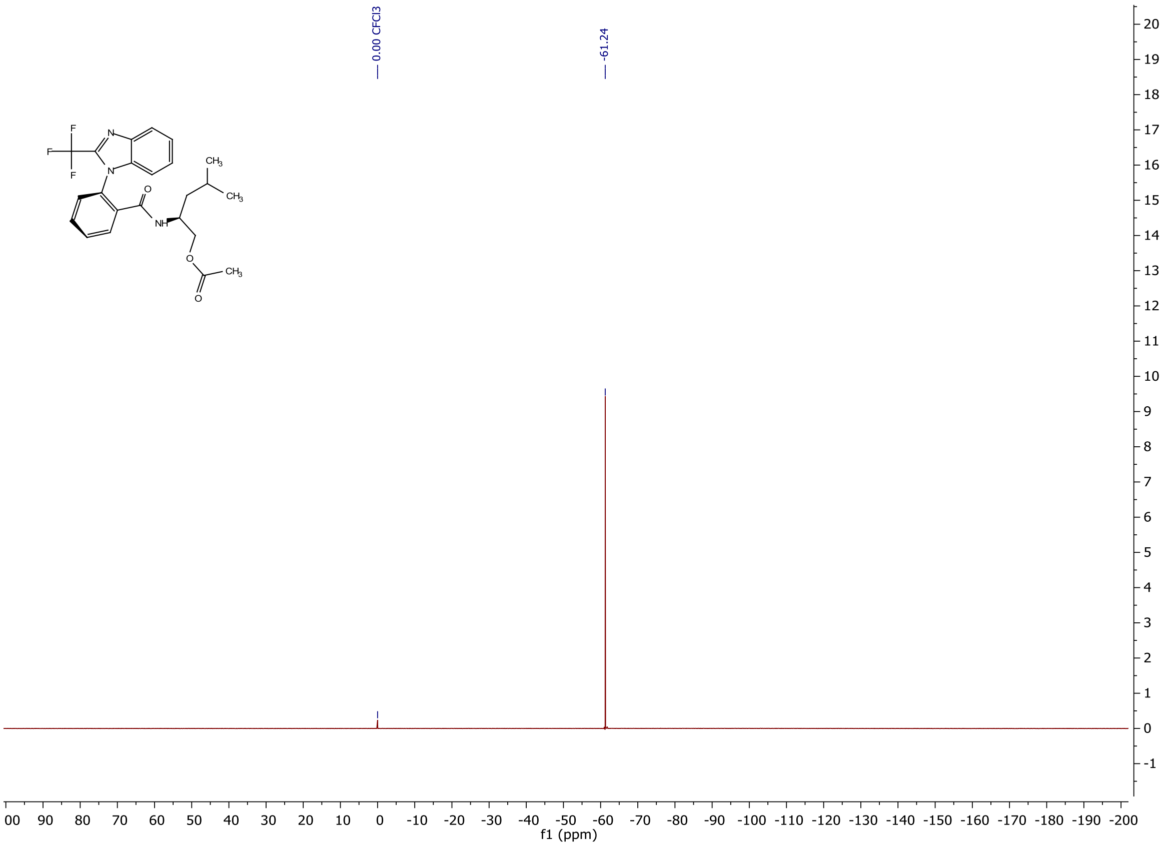

^{19}F NMR (376 MHz, DMSO-d_6)

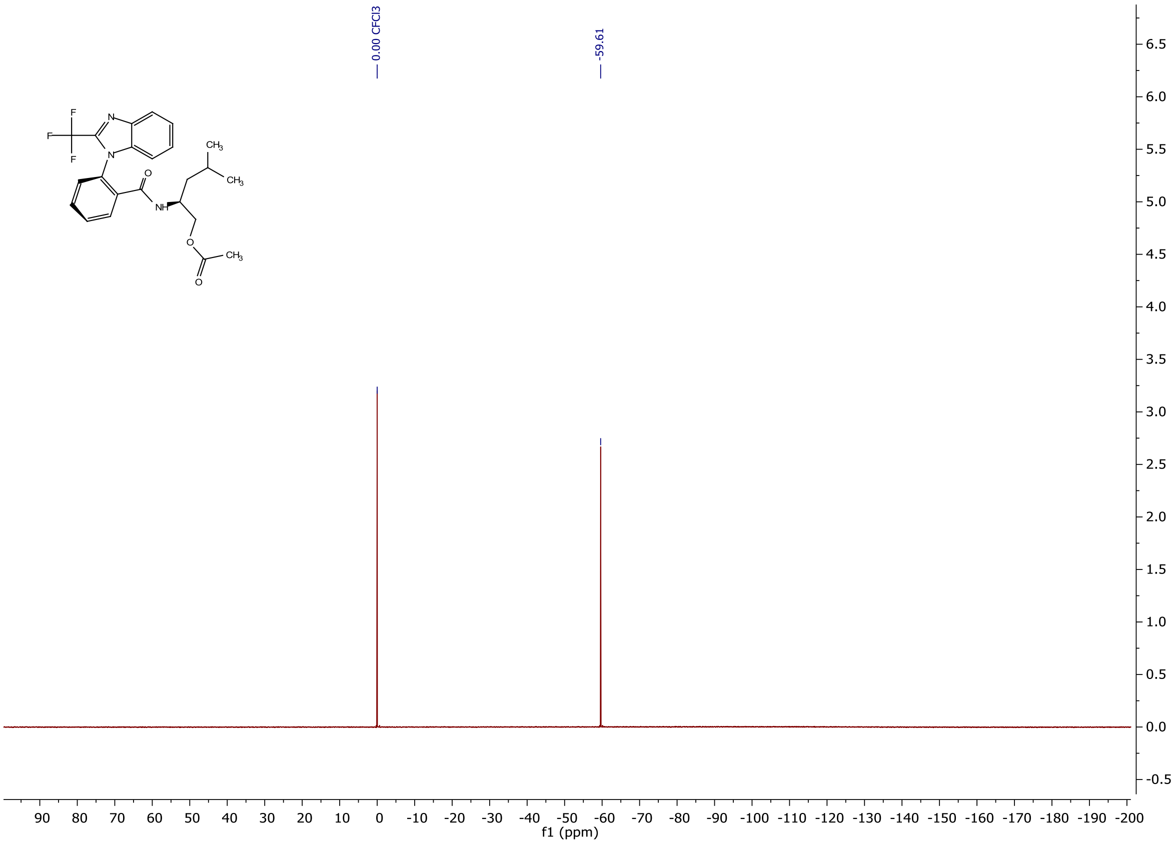

(*S*)-4-Methyl-2-(2-((*M*)-2-(trifluoromethyl)-1*H*-benzo[*d*]imidazol-1-yl)benzamido)pentyl acetate **(*M*)-43**

^{19}F NMR (376 MHz, $CDCl_3$)

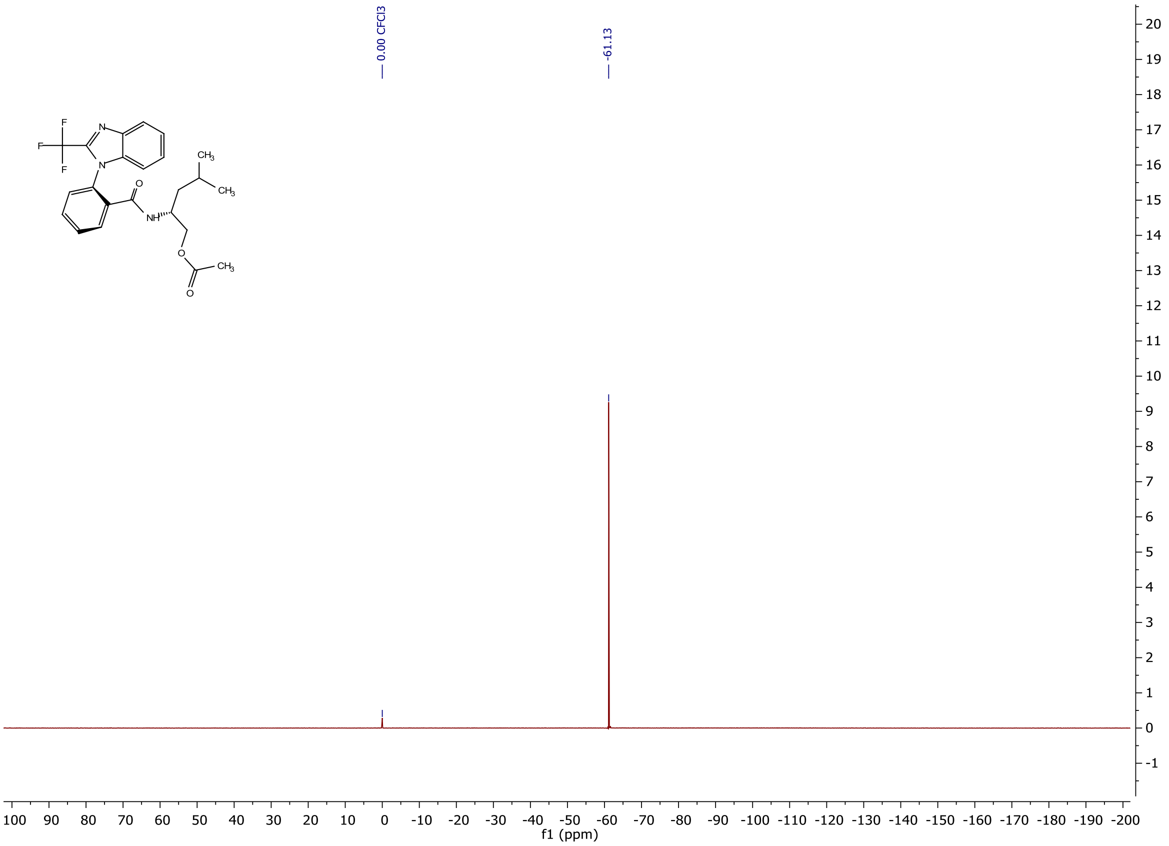

^{19}F NMR (376 MHz, DMSO-d_6)

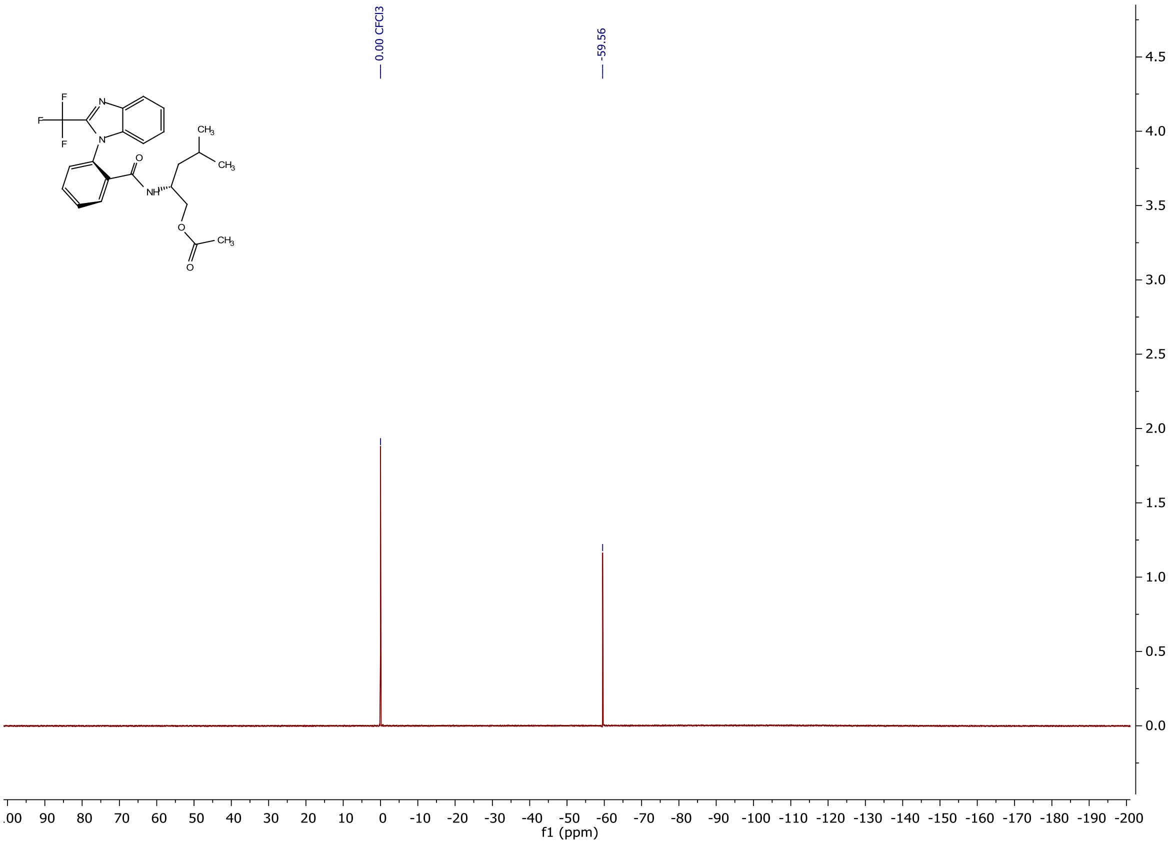

N-((1*R*,2*R*)-1,3-Dihydroxy-1-(4-nitrophenyl)propan-2-yl)-2-((*P*)-2-(trifluoromethyl)-1*H*-benzo[*d*]imidazol-1-yl)benzamide **(*P*)-44**

^{19}F NMR (376 MHz, $CDCl_3$)

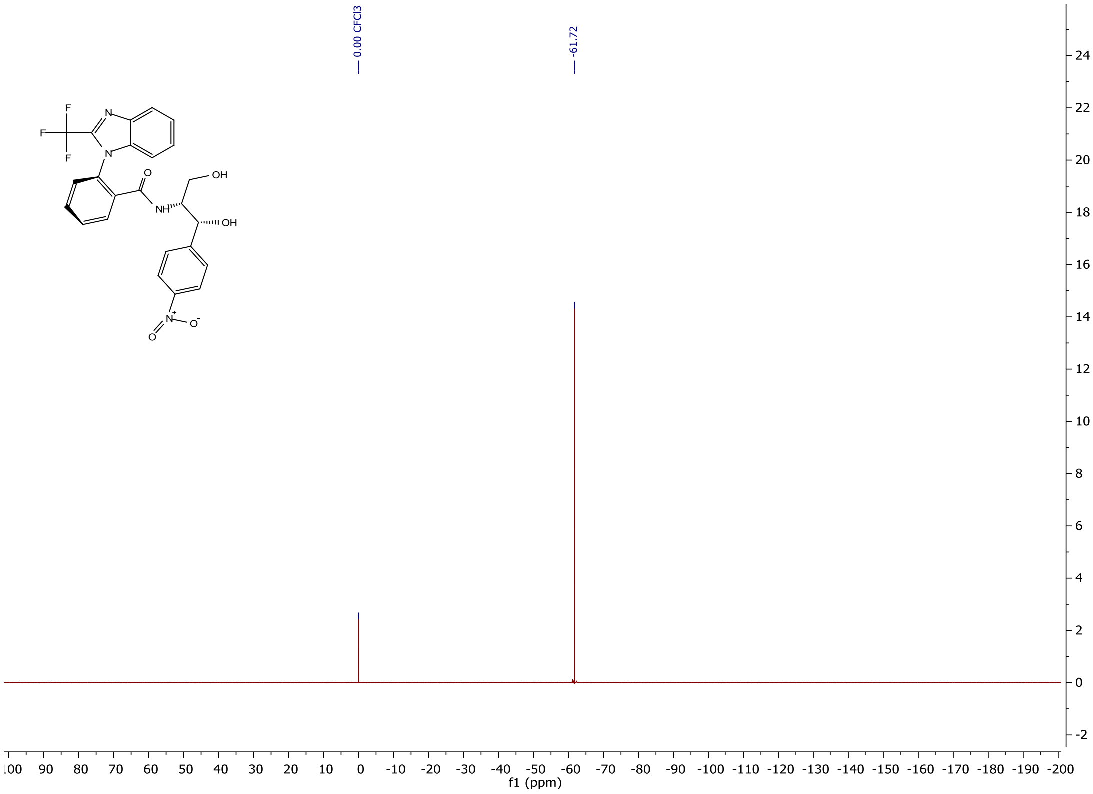

^{19}F NMR (376 MHz, DMSO-d_6)

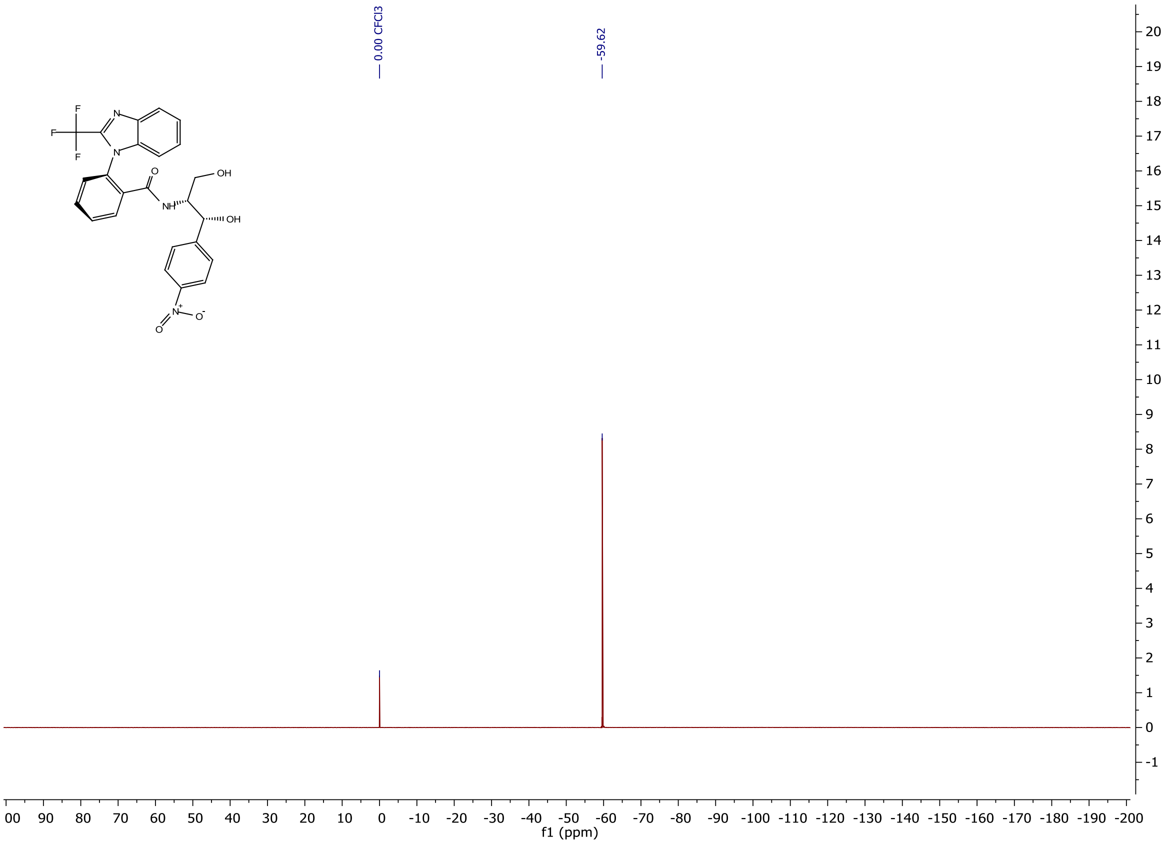

N-((1*R*,2*R*)-1,3-Dihydroxy-1-(4-nitrophenyl)propan-2-yl)-2-((*M*)-2-(trifluoromethyl)-1*H*-benzo[*d*]imidazol-1-yl)benzamide **(*M*)-44**

^{19}F NMR (376 MHz, $CDCl_3$)

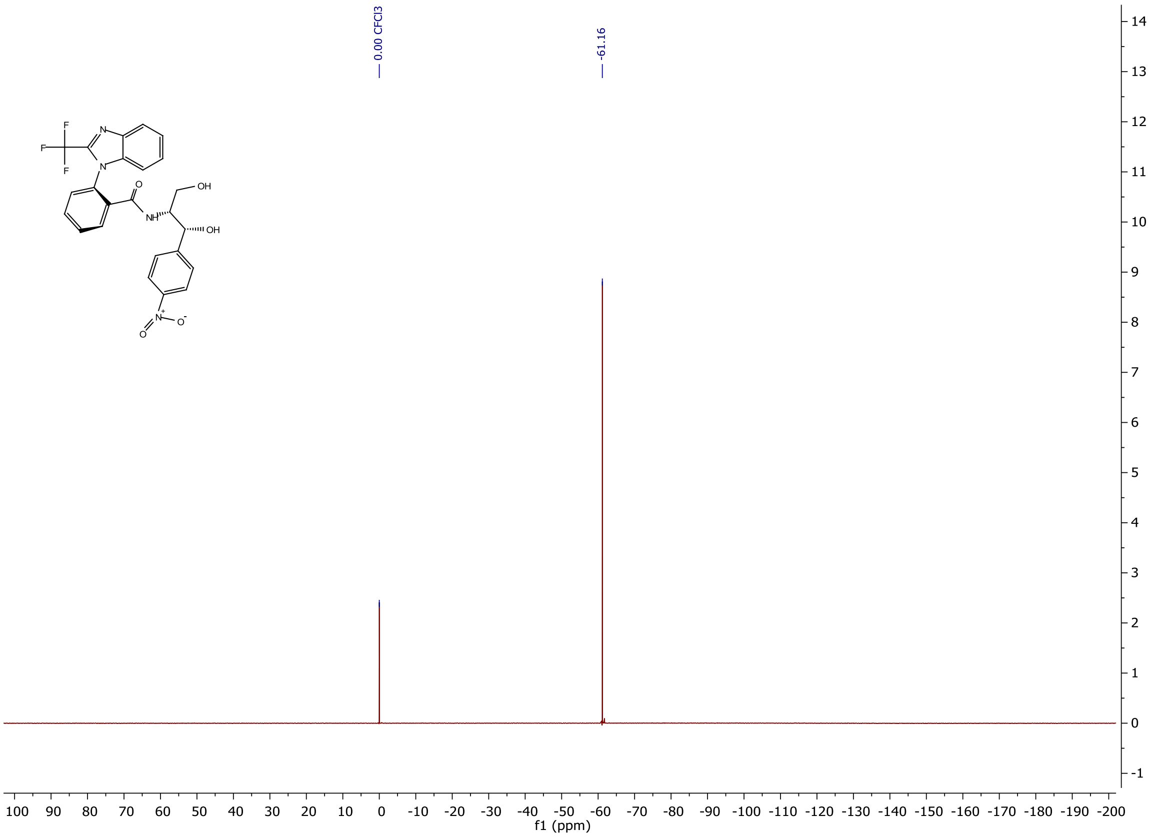

^{19}F NMR (376 MHz, DMSO-d_6)

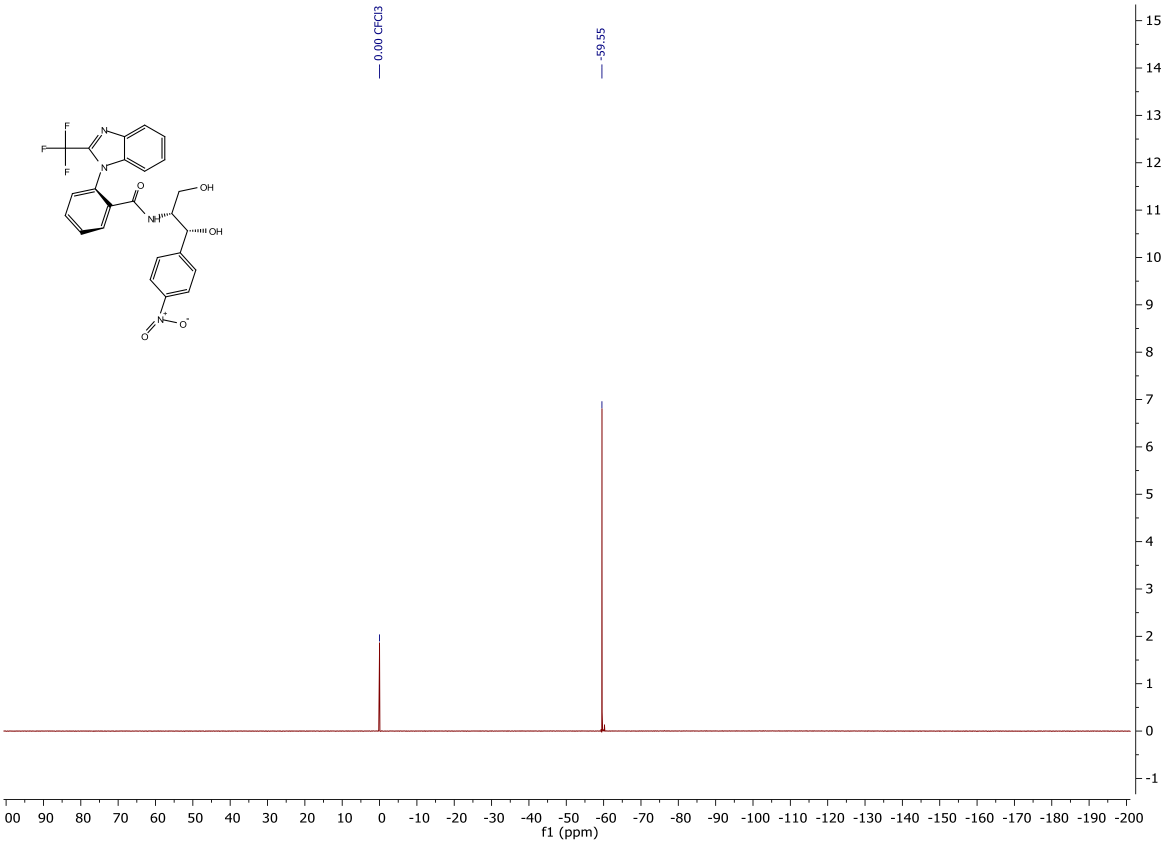

(1*R*,2*R*)-1-(4-Nitrophenyl)-2-(2-((*P*)-2-(trifluoromethyl)-1*H*-benzo[*d*]imidazol-1-yl)benzamido)propane-1,3-diyl diacetate **(*P*)-45**

^{19}F NMR (376 MHz, $CDCl_3$)

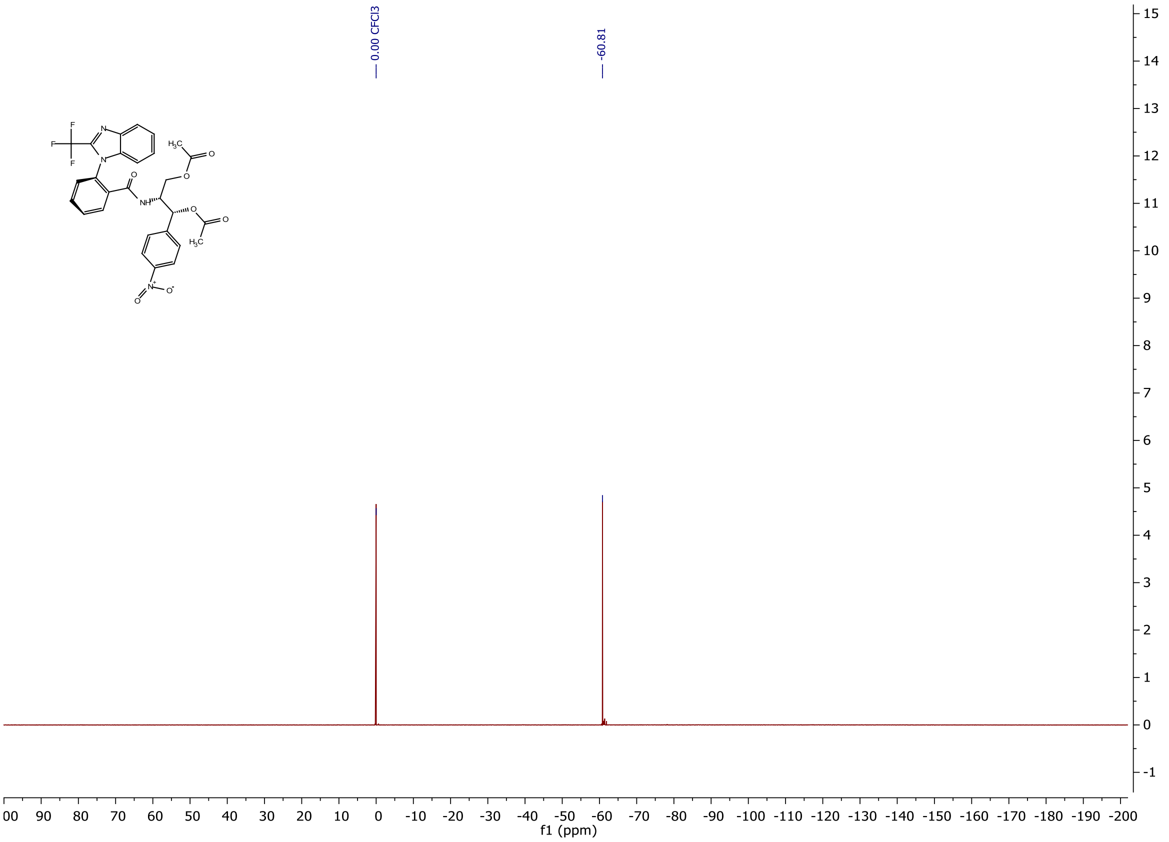

^{19}F NMR (376 MHz, DMSO-d_6)

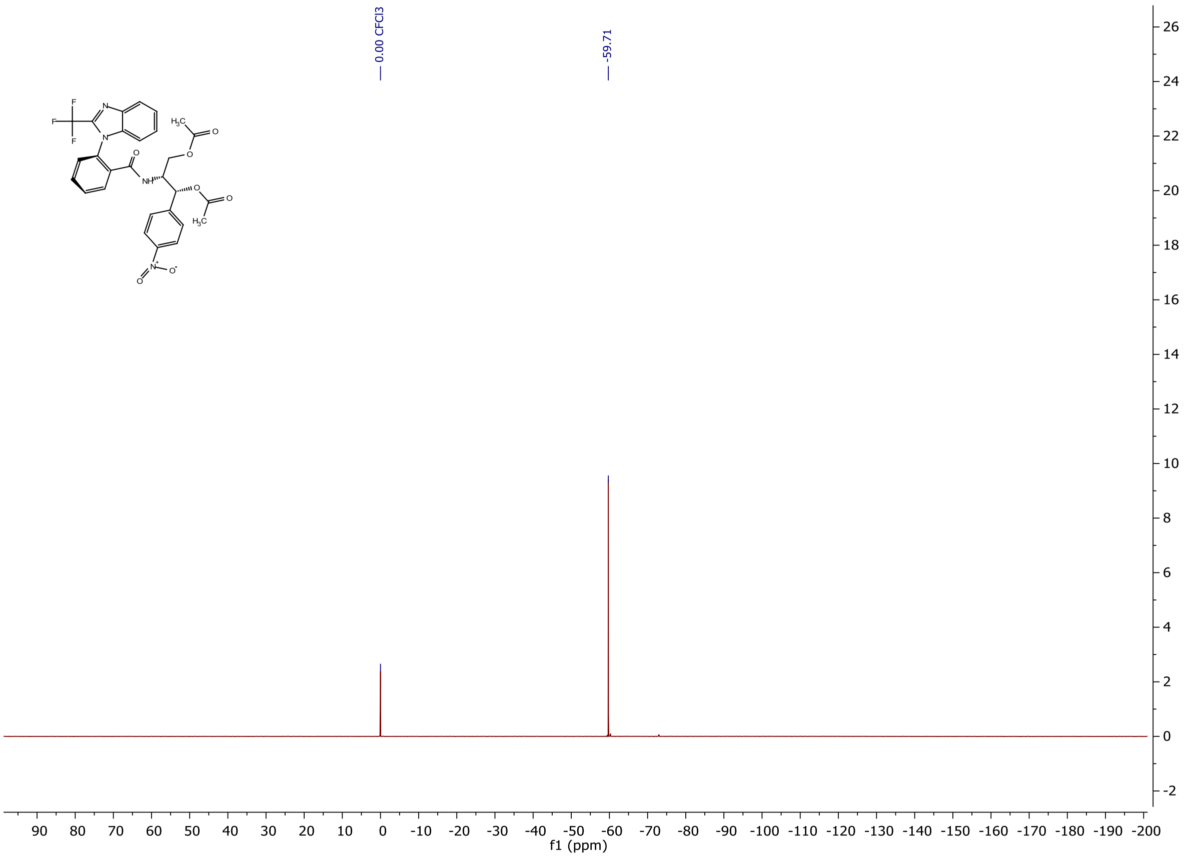

(1*R*,2*R*)-1-(4-Nitrophenyl)-2-(2-((*M*)-2-(trifluoromethyl)-1*H*-benzo[*d*]imidazol-1-yl)benzamido)propane-1,3-diyl diacetate **(*M*)-45**

^{19}F NMR (376 MHz, $CDCl_3$)

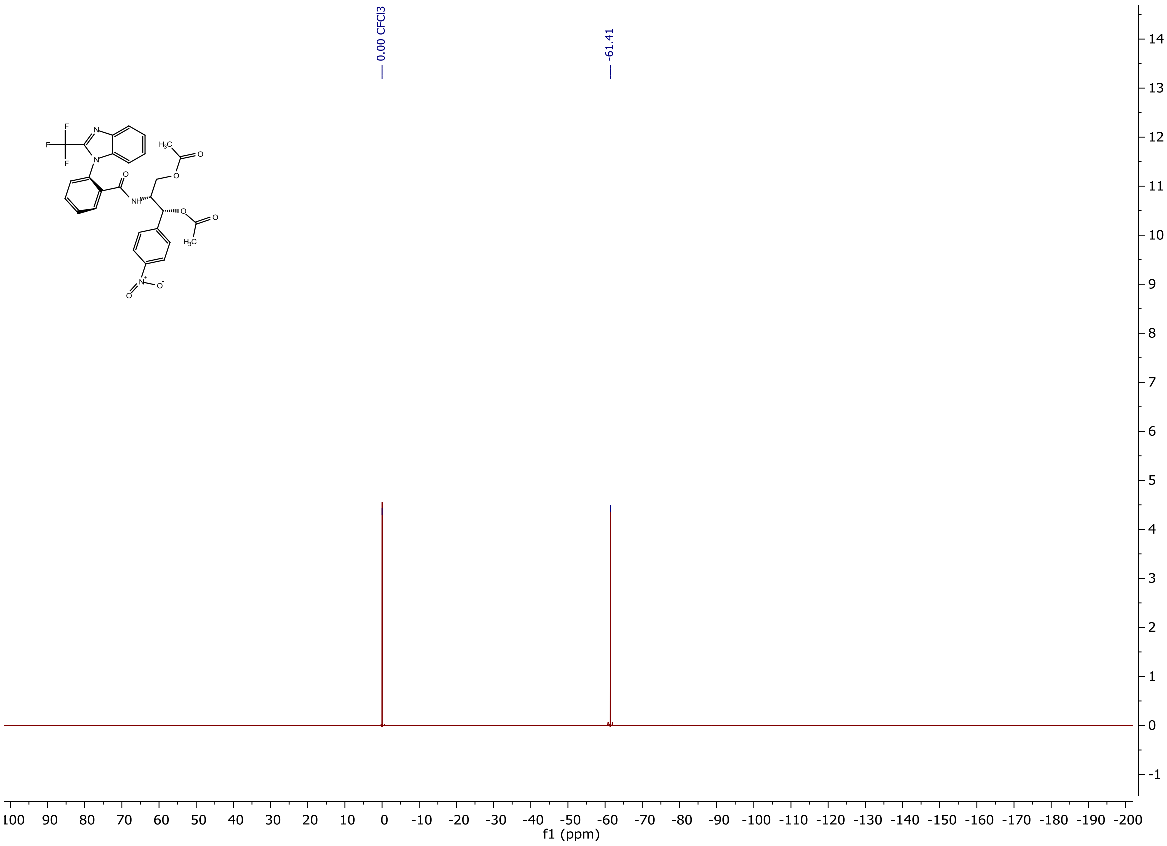

^{19}F NMR (376 MHz, DMSO-d_6)

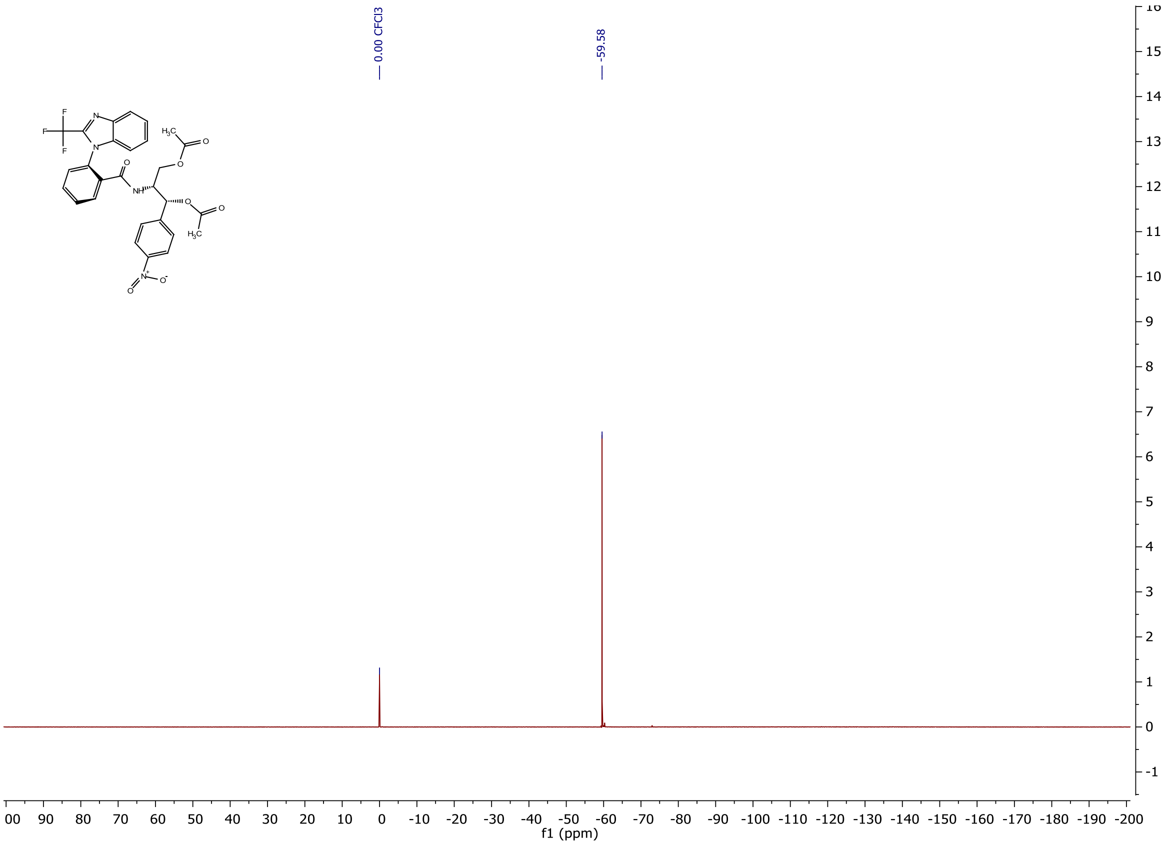

N-((4*R*,5*R*)-2,2-Dimethyl-4-(4-nitrophenyl)-1,3-dioxan-5-yl)-2-((*P*)-2-(trifluoromethyl)-1*H*-benzo[*d*]imidazol-1-yl)benzamide **(*P*)-46**

^{19}F NMR (376 MHz, $CDCl_3$)

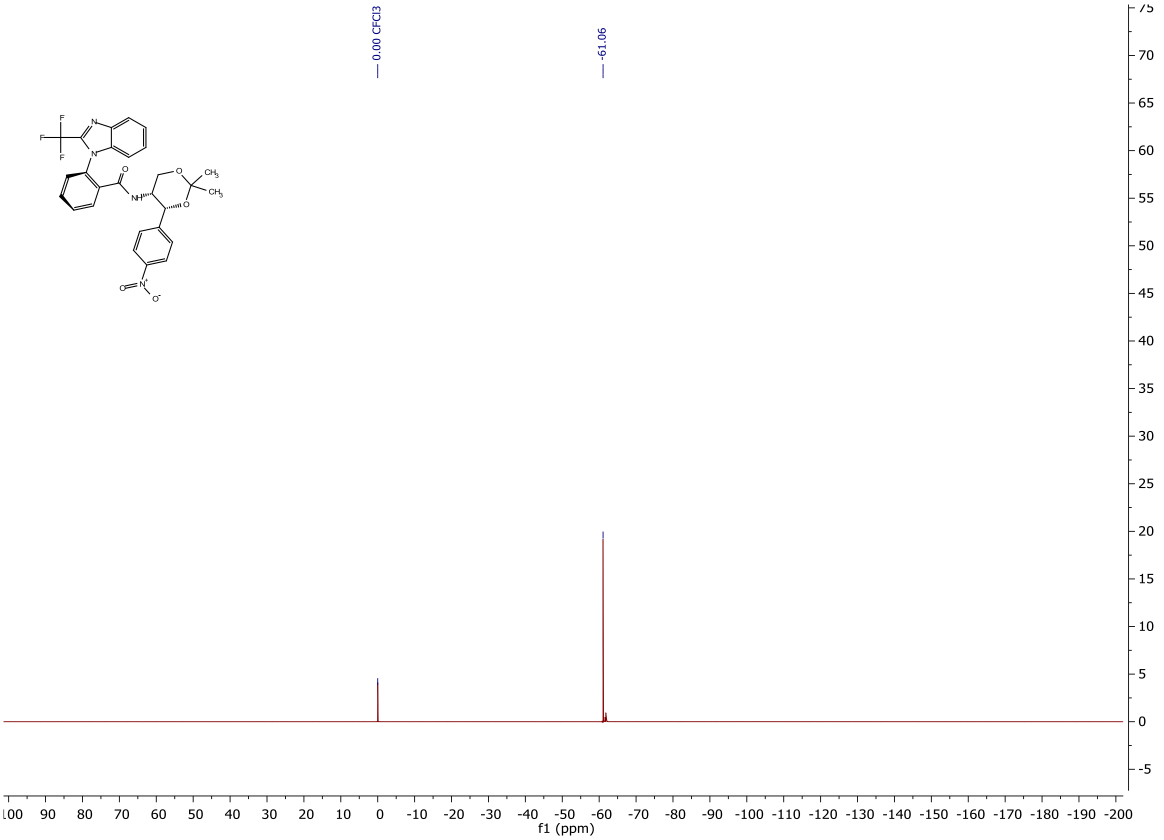

^{19}F NMR (376 MHz, DMSO-d_6)

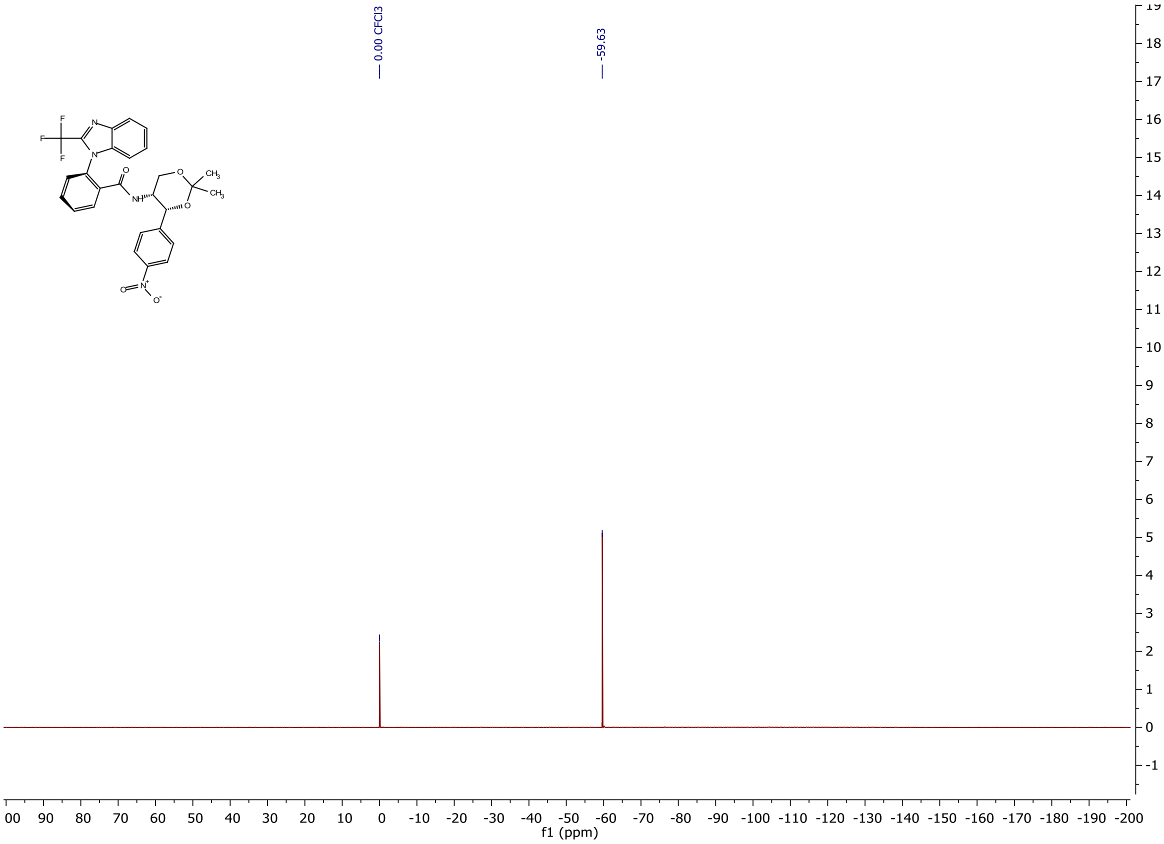

N-((4*R*,5*R*)-2,2-Dimethyl-4-(4-nitrophenyl)-1,3-dioxan-5-yl)-2-((*M*)-2-(trifluoromethyl)-1*H*-benzo[*d*]imidazol-1-yl)benzamide **(*M*)-46**

^{19}F NMR (376 MHz, $CDCl_3$)

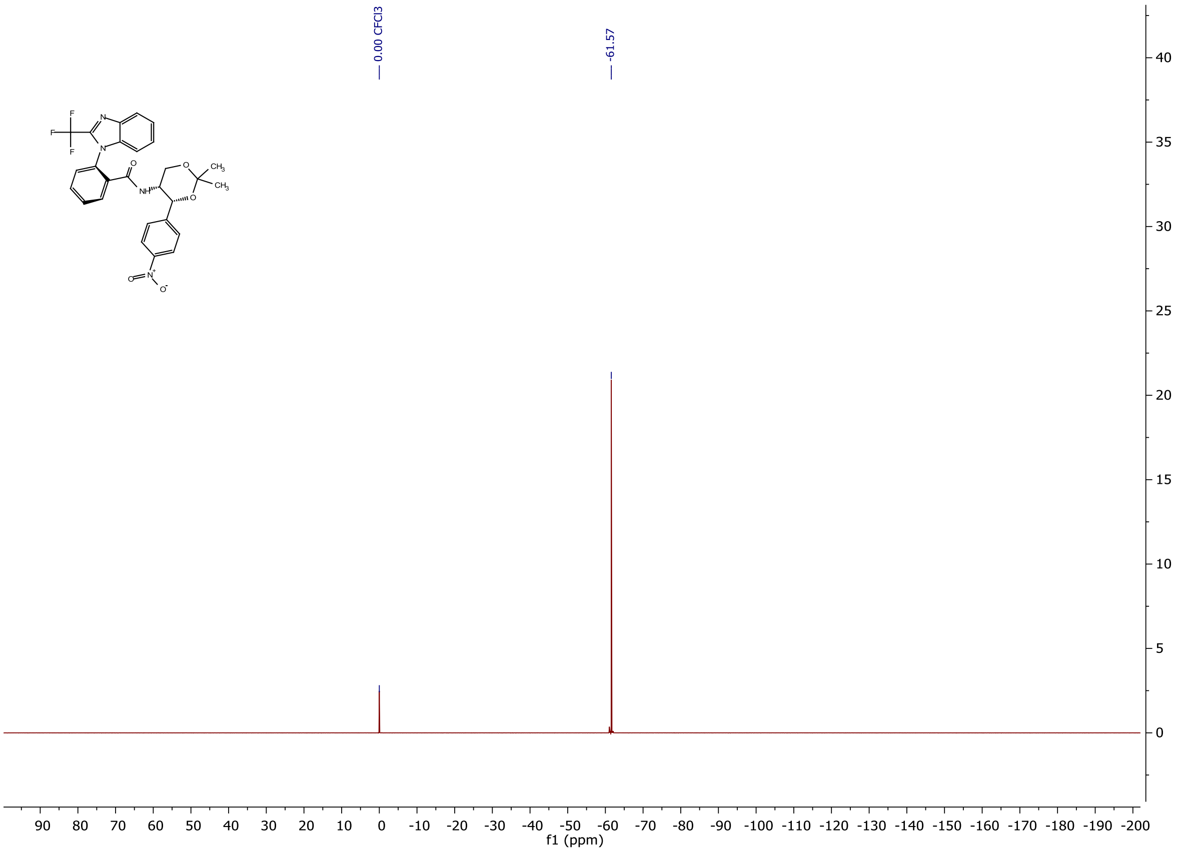

^{19}F NMR (376 MHz, DMSO-d_6)

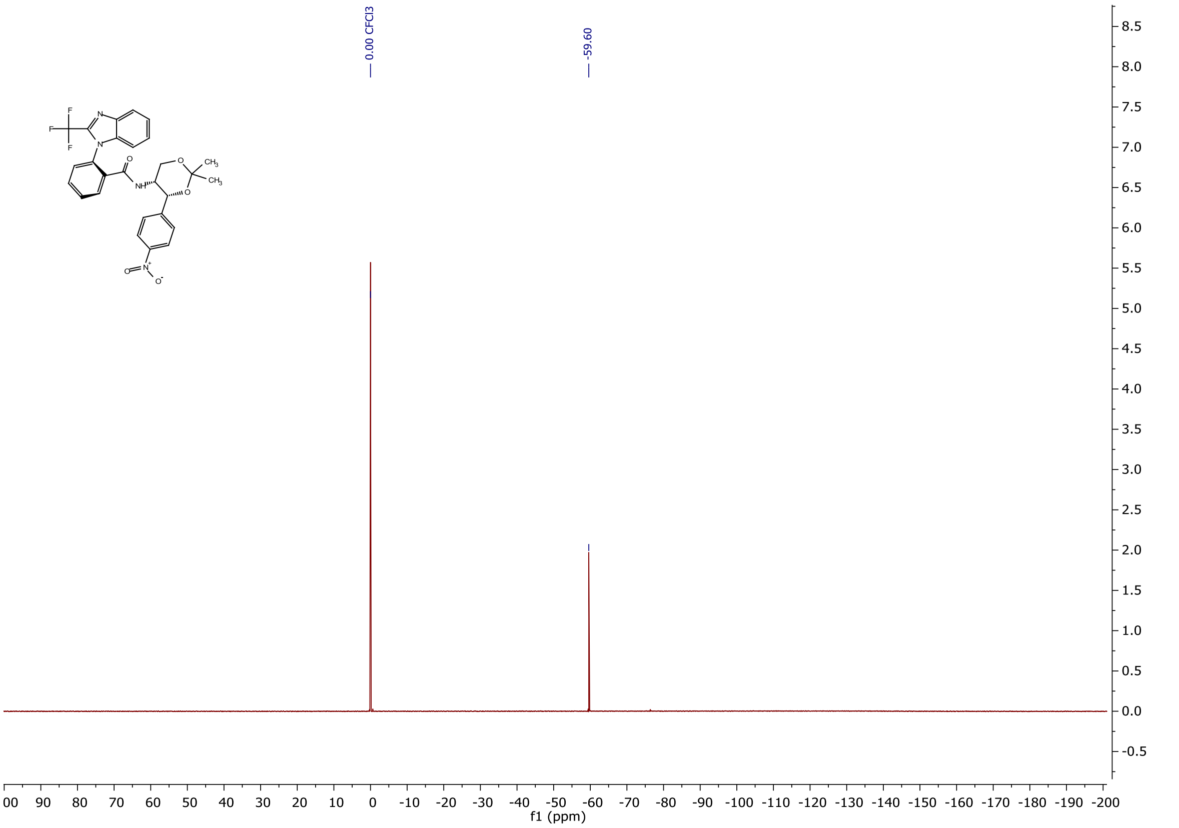

N-((4*R*,5*R*)-4-(4-Aminophenyl)-2,2-dimethyl-1,3-dioxan-5-yl)-2-((*P*)-2-(trifluoromethyl)-1*H*-benzo[*d*]imidazol-1-yl)benzamide **(*P*)-47**

^{19}F NMR (376 MHz, $CDCl_3$)

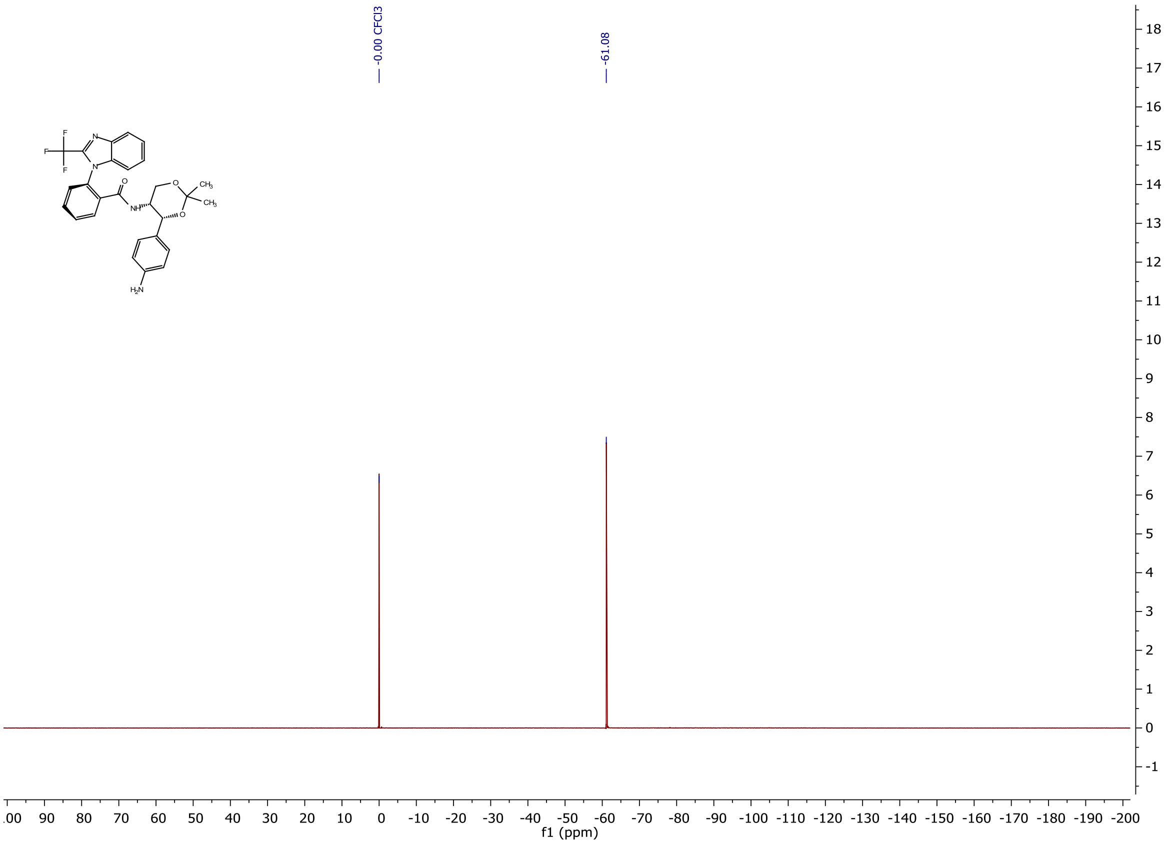

^{19}F NMR (376 MHz, DMSO-d_6)

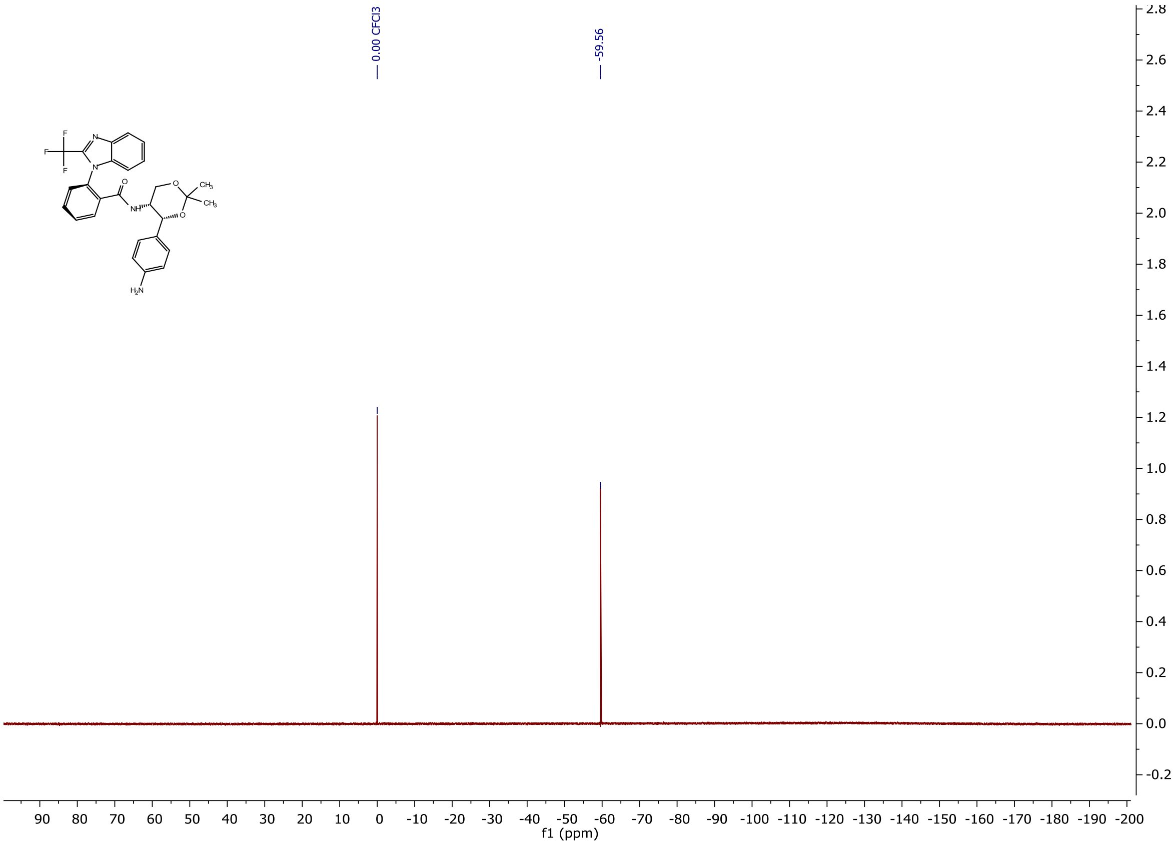

N-((4*R*,5*R*)-4-(4-Aminophenyl)-2,2-dimethyl-1,3-dioxan-5-yl)-2-((*M*)-2-(trifluoromethyl)-1*H*-benzo[*d*]imidazol-1-yl)benzamide **(*M*)-47**

^{19}F NMR (376 MHz, $CDCl_3$)

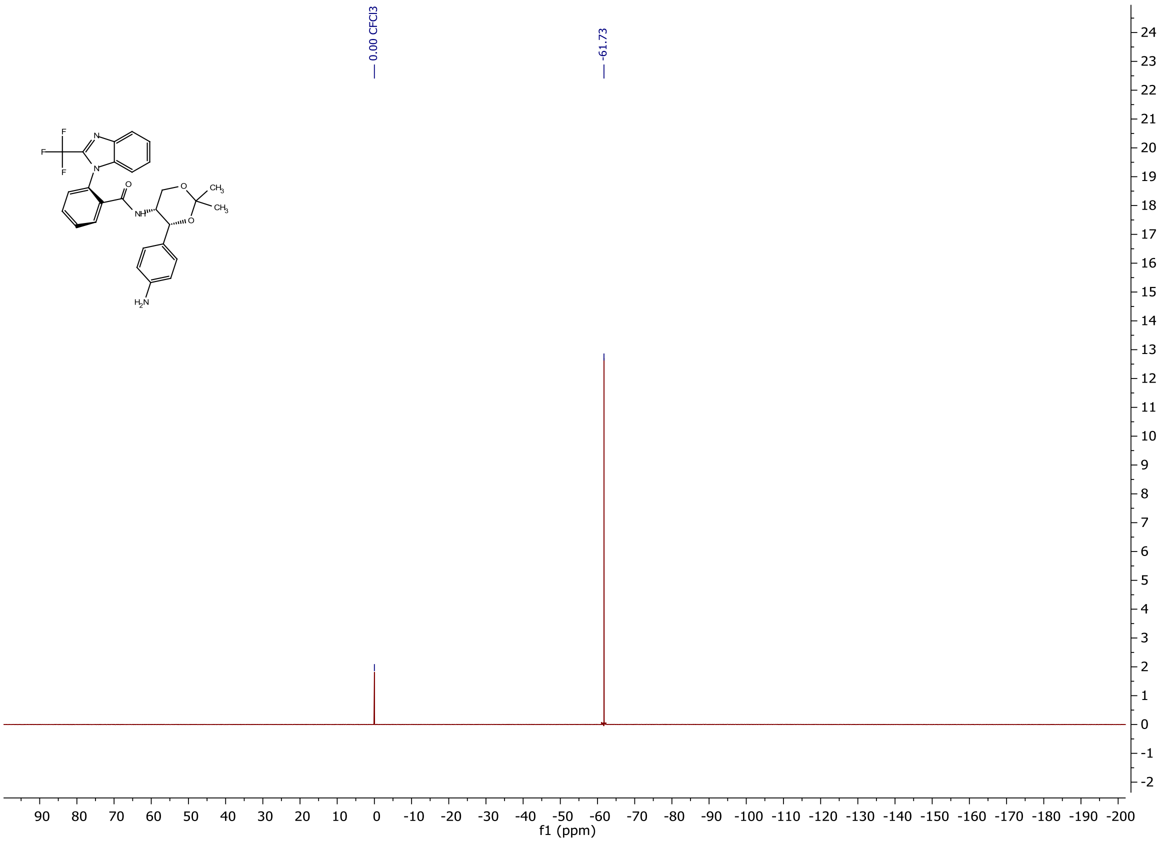

^{19}F NMR (376 MHz, DMSO-d_6)

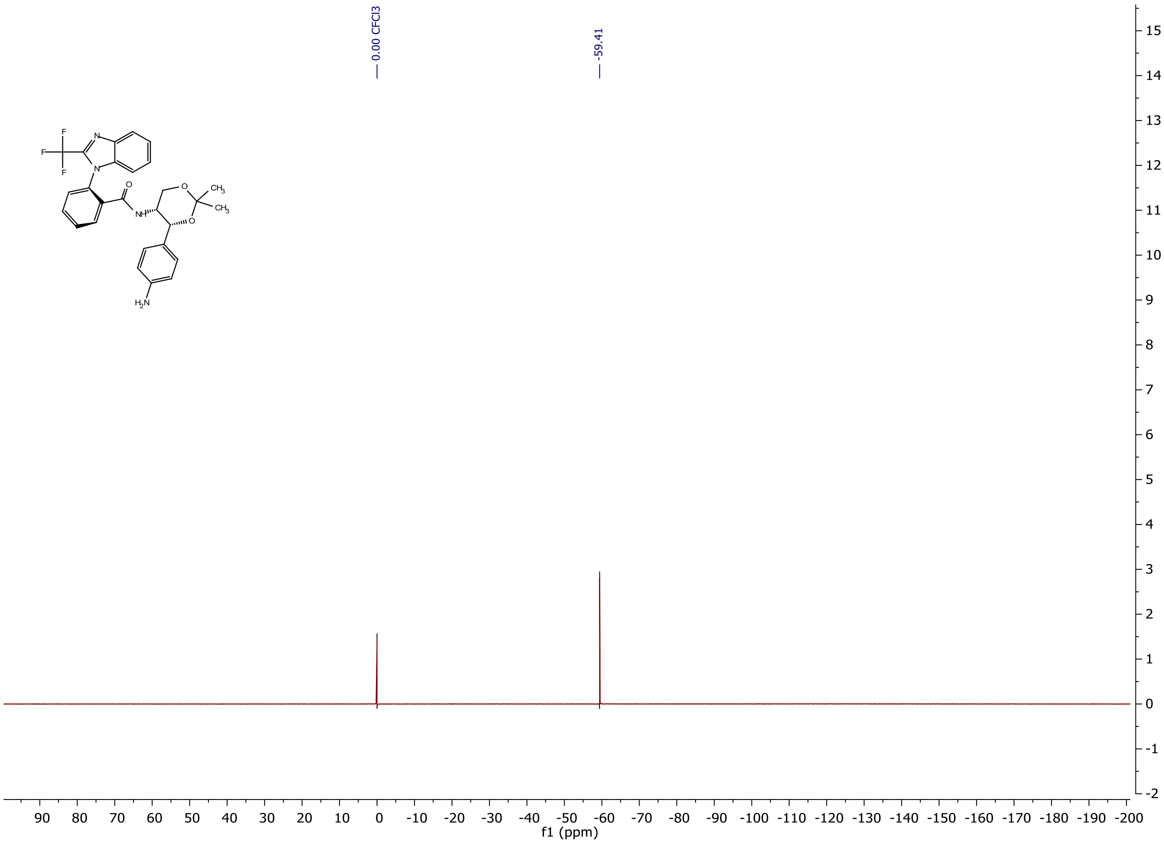

N-((1*S*,2*S*)-2-Amino-1,2-diphenylethyl)-2-((*P*)-2-(trifluoromethyl)-1*H*-benzo[*d*]imidazol-1-yl)benzamide **(*P*)-48**

^{19}F NMR (376 MHz, $CDCl_3$)

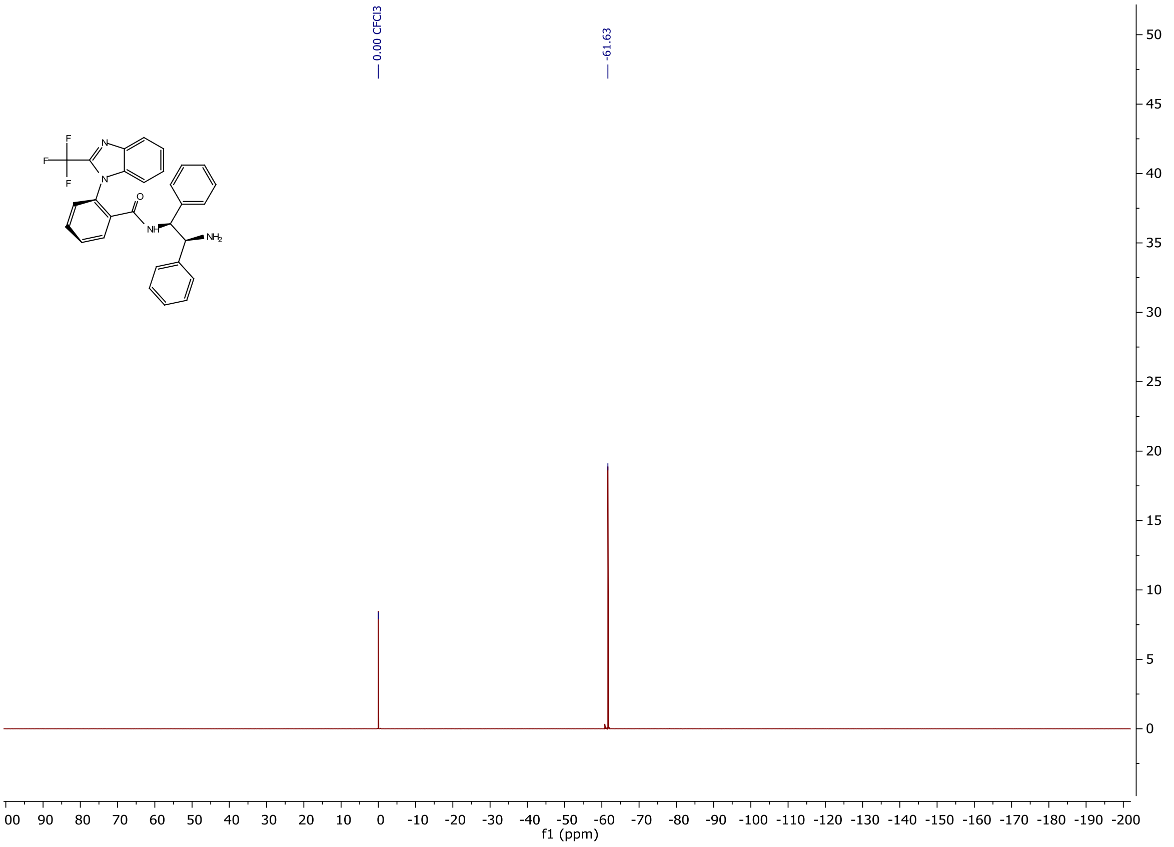

^{19}F NMR (376 MHz, DMSO-d_6)

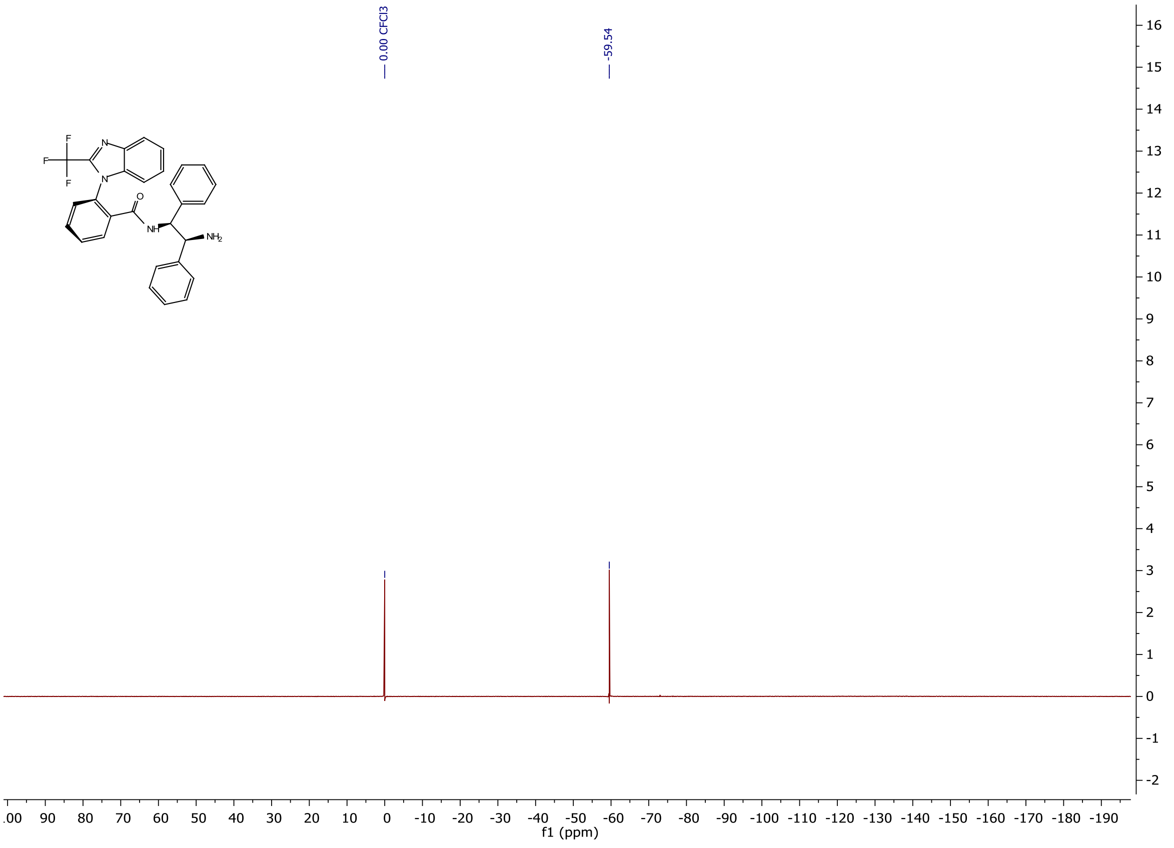

N-((1*S*,2*S*)-2-Amino-1,2-diphenylethyl)-2-((*M*)-2-(trifluoromethyl)-1*H*-benzo[*d*]imidazol-1-yl)benzamide **(*M*)-48**

^{19}F NMR (376 MHz, $CDCl_3$)

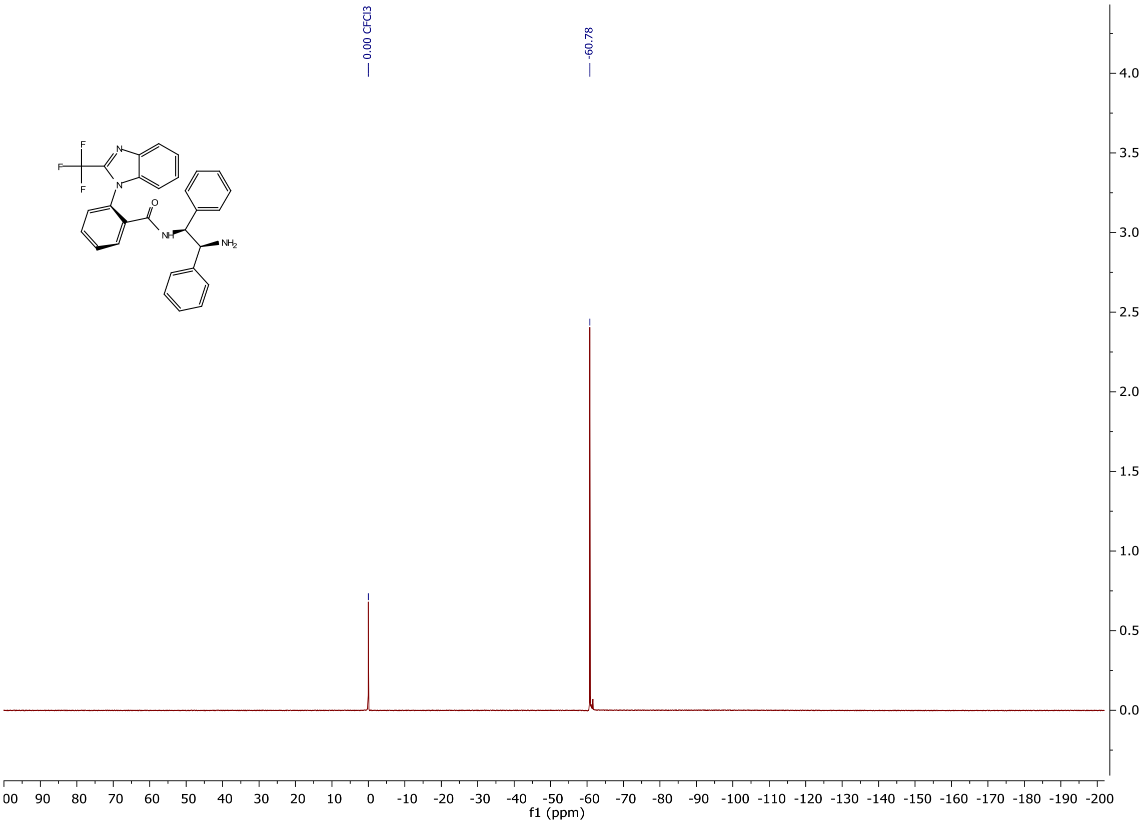

^{19}F NMR (376 MHz, DMSO-d_6)

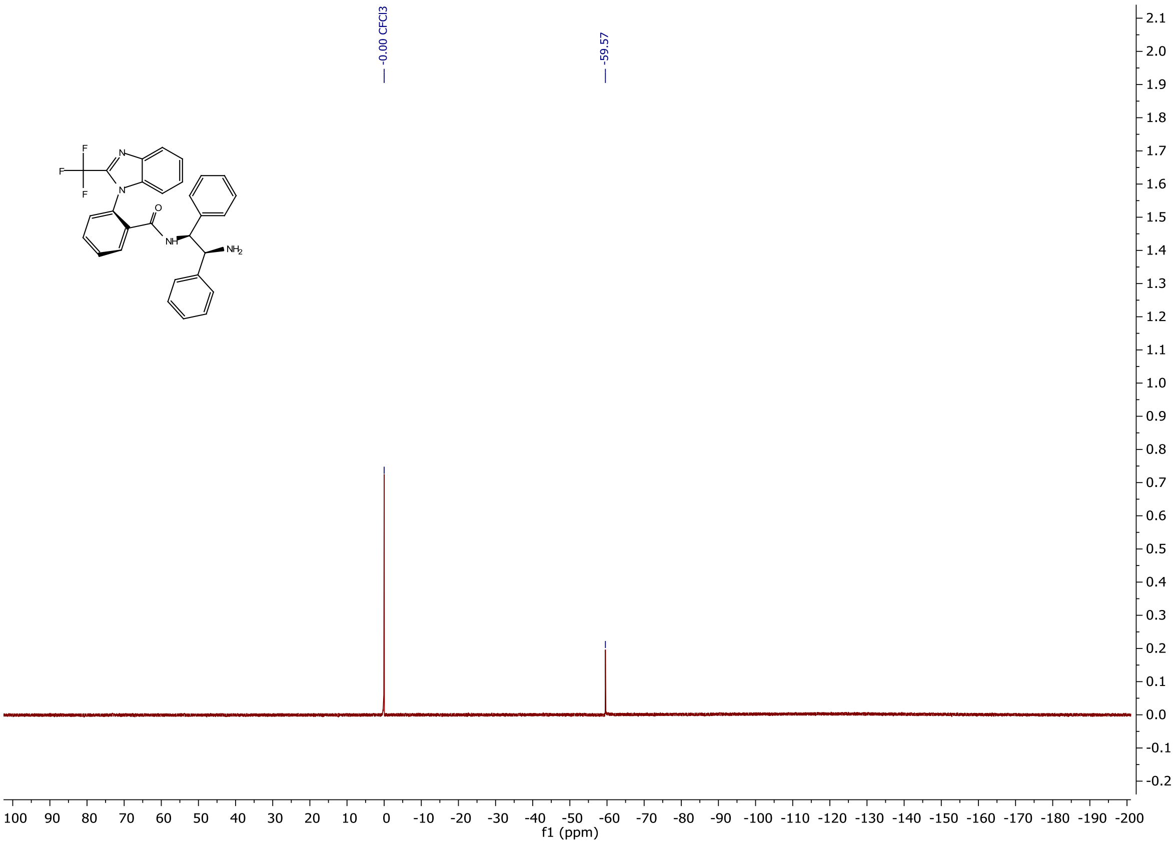

N-((1*S*,2*S*)-2-(Dimethylamino)-1,2-diphenylethyl)-2-((*P*)-2-(trifluoromethyl)-1*H*-benzo[*d*]imidazol-1-yl)benzamide **(*P*)-49**

^{19}F NMR (376 MHz, $CDCl_3$)

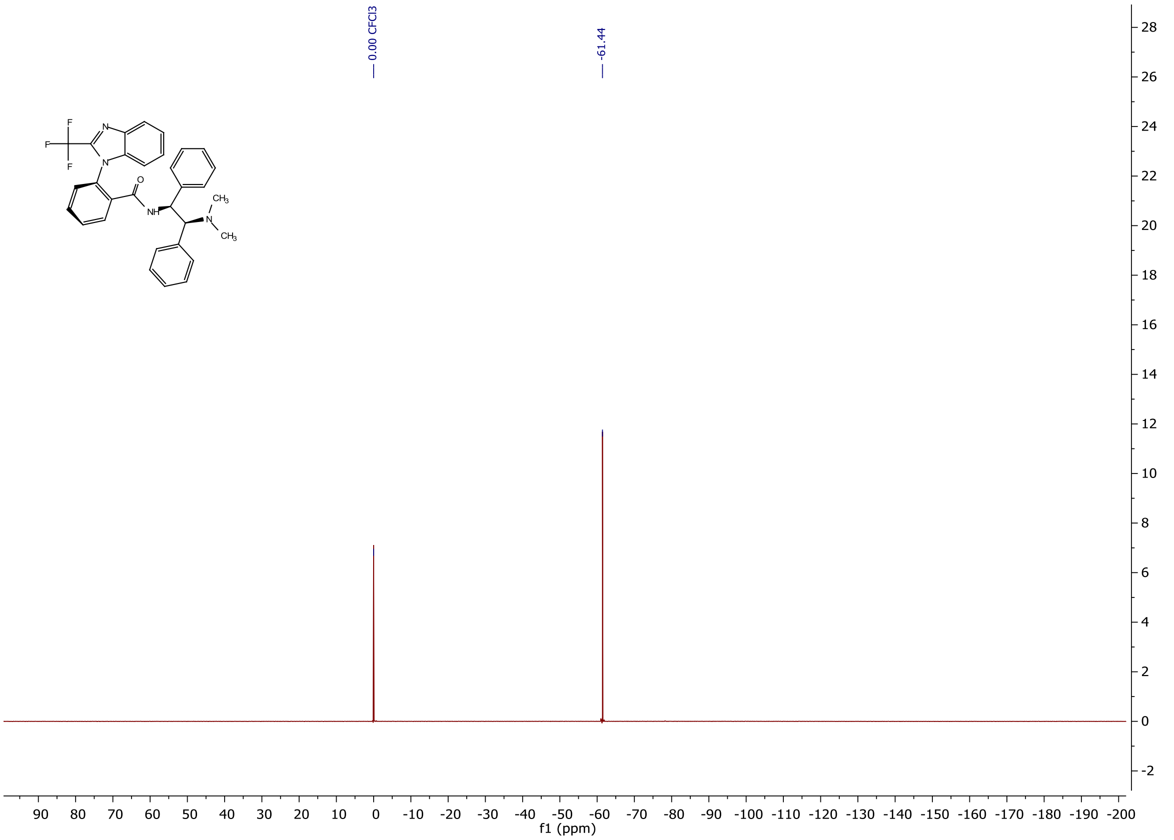

^{19}F NMR (376 MHz, DMSO-d_6)

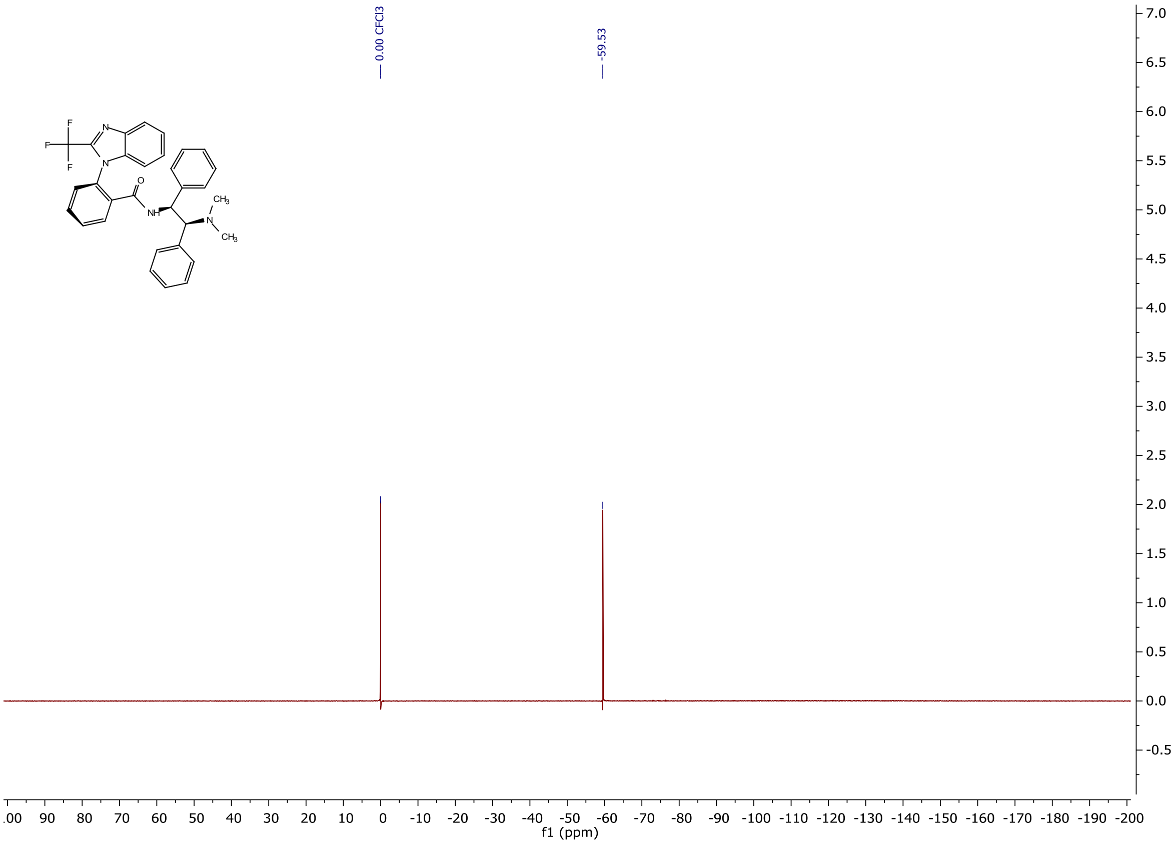

N-((1*S*,2*S*)-2-(Dimethylamino)-1,2-diphenylethyl)-2-((*M*)-2-(trifluoromethyl)-1*H*-benzo[*d*]imidazol-1-yl)benzamide **(*M*)-49**

^{19}F NMR (376 MHz, $CDCl_3$)

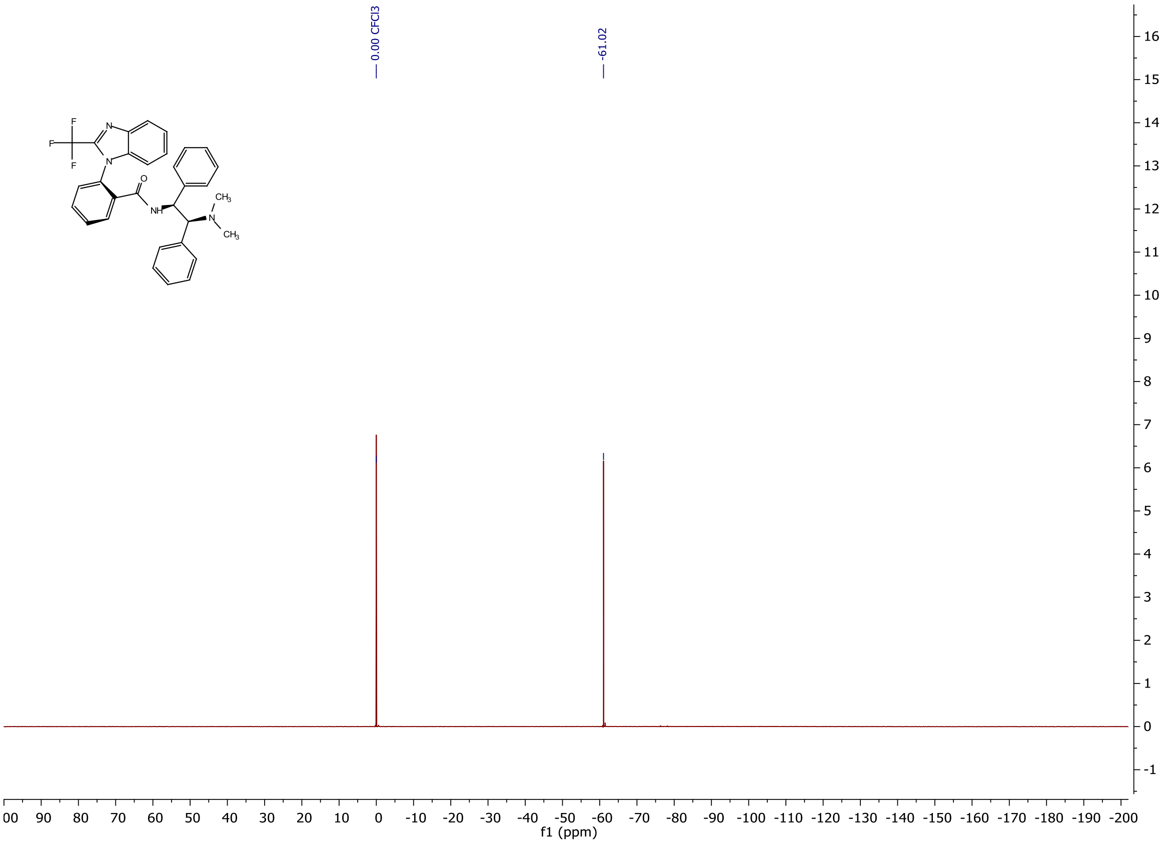

^{19}F NMR (376 MHz, DMSO-d_6)

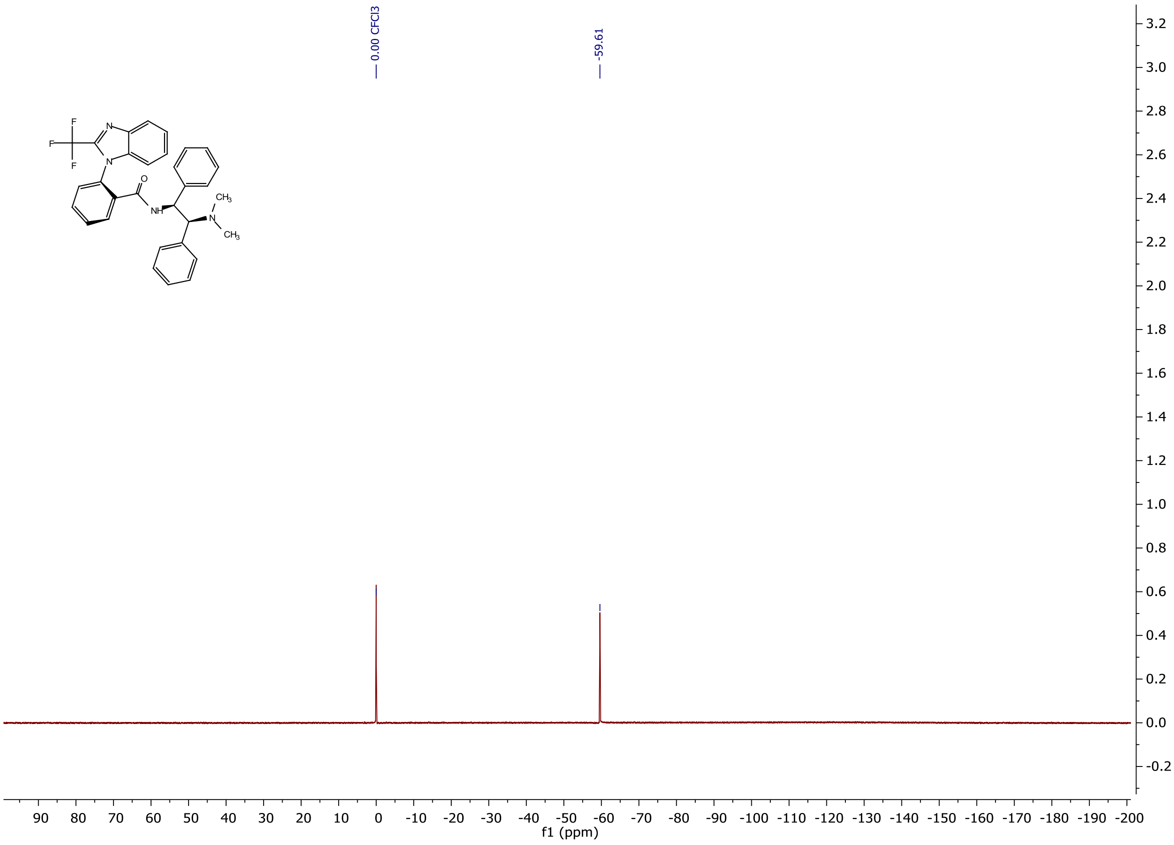

N-((1*S*,2*S*)-2-Acetamido-1,2-diphenylethyl)-2-((*P*)-2-(trifluoromethyl)-1*H*-benzo[*d*]imidazol-1-yl)benzamide **(*P*)-50**

^{19}F NMR (376 MHz, $CDCl_3$)

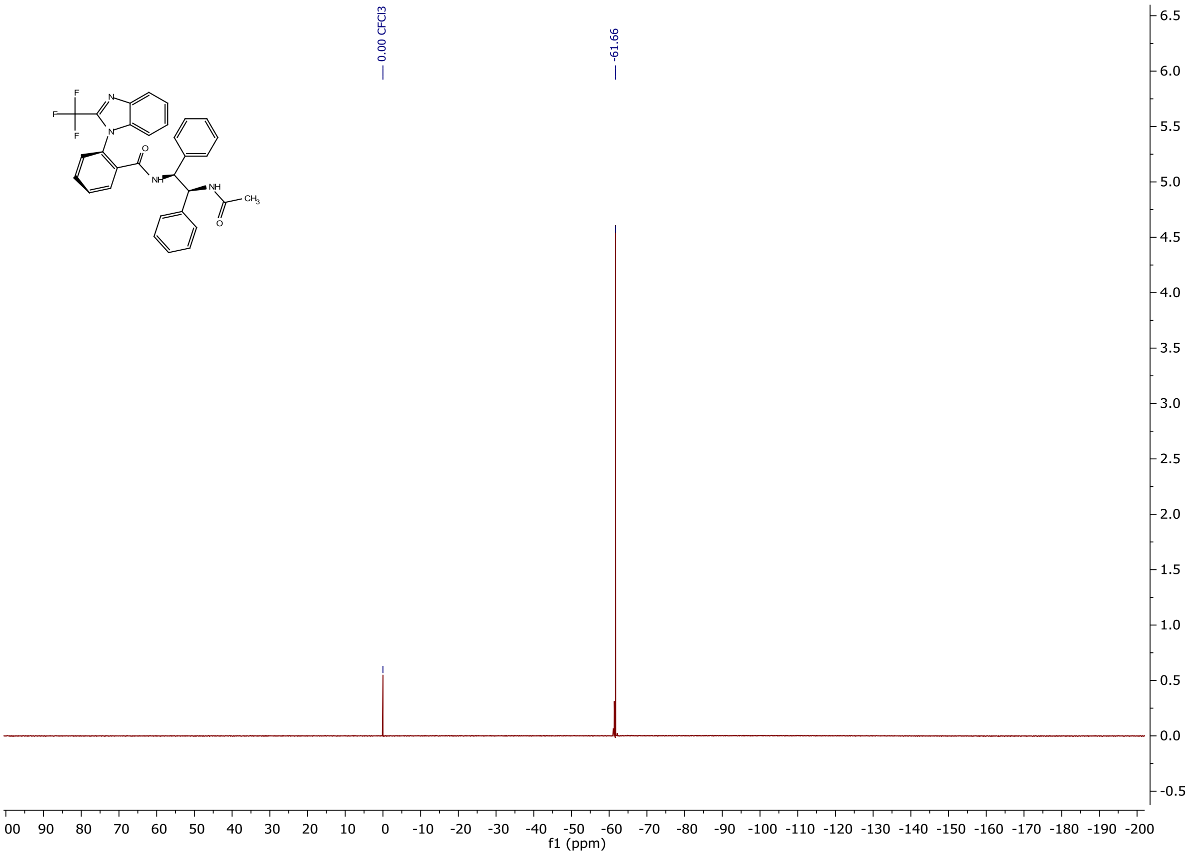

^{19}F NMR (376 MHz, DMSO-d_6)

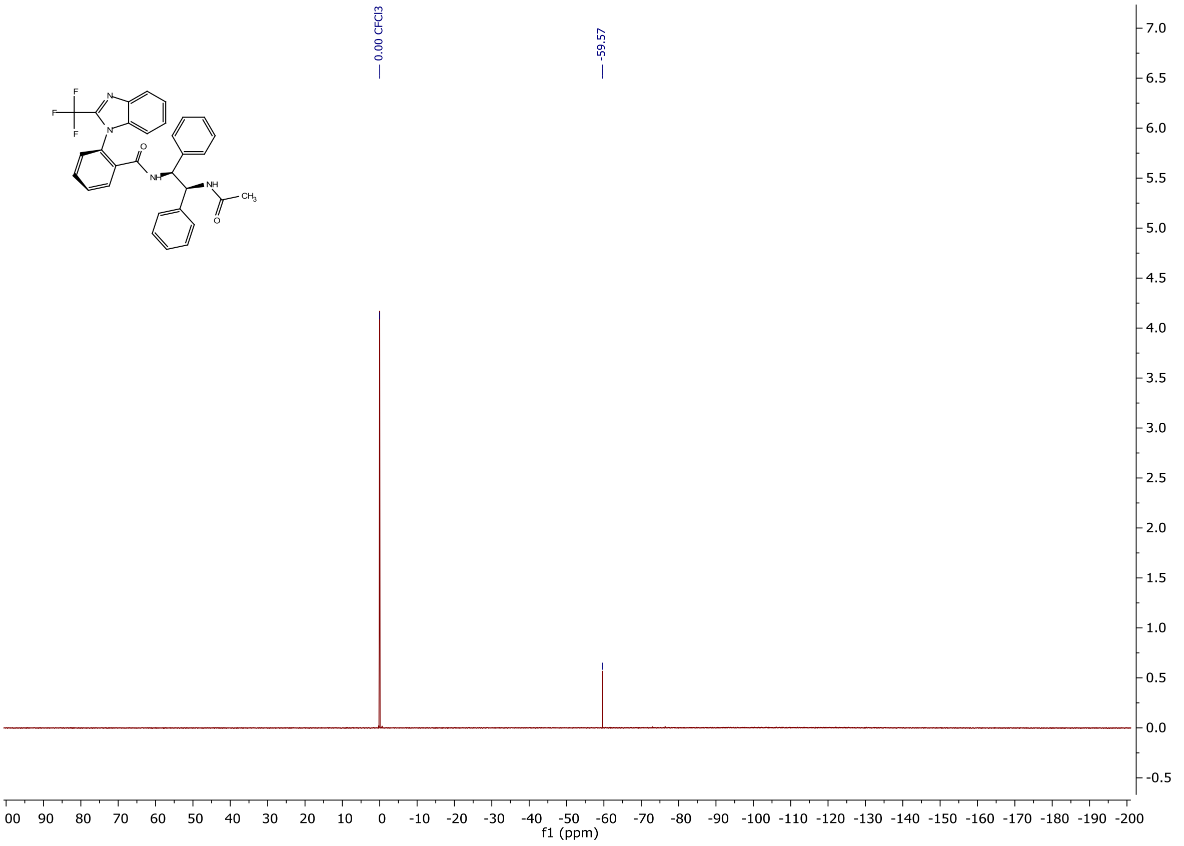

N-*N*-((1*S*,2*S*)-2-Acetamido-1,2-diphenylethyl)-2-((*M*)-2-(trifluoromethyl)-1*H*-benzo[*d*]imidazol-1-yl)benzamide **(*M*)-50**

^{19}F NMR (376 MHz, $CDCl_3$)

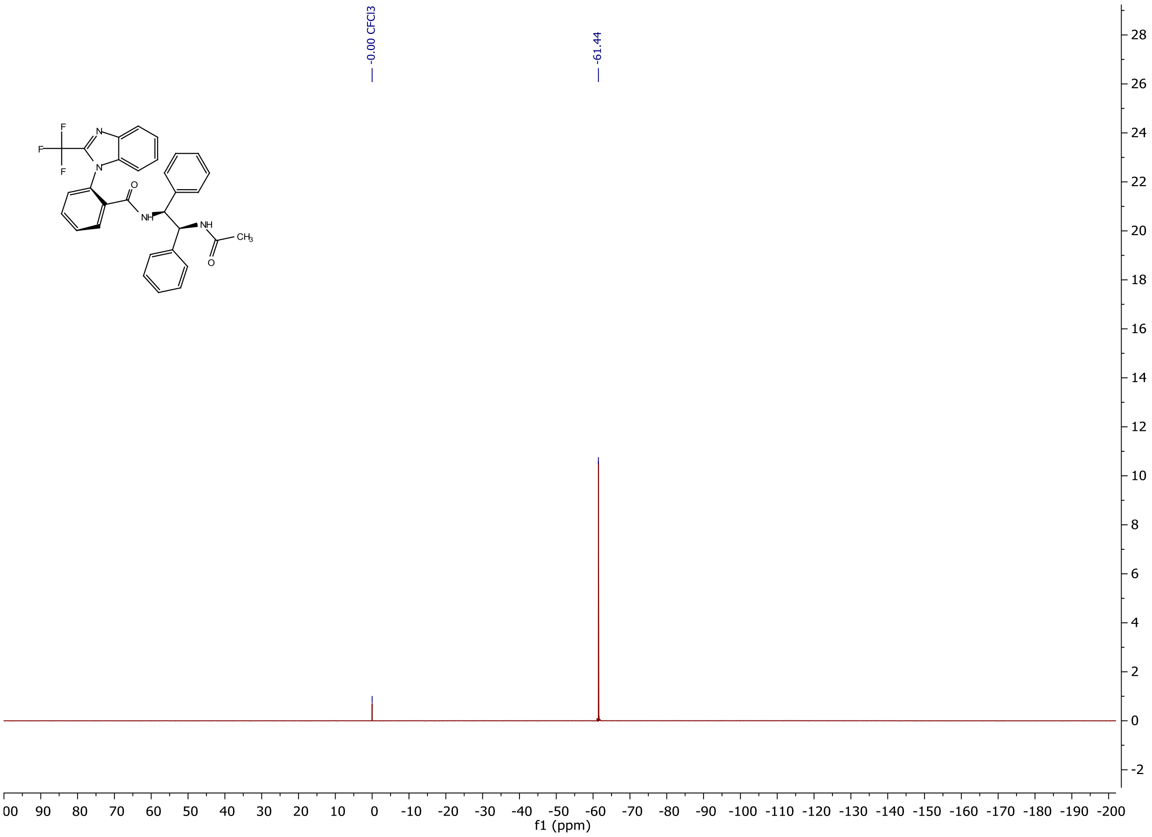

^{19}F NMR (376 MHz, DMSO-d_6)

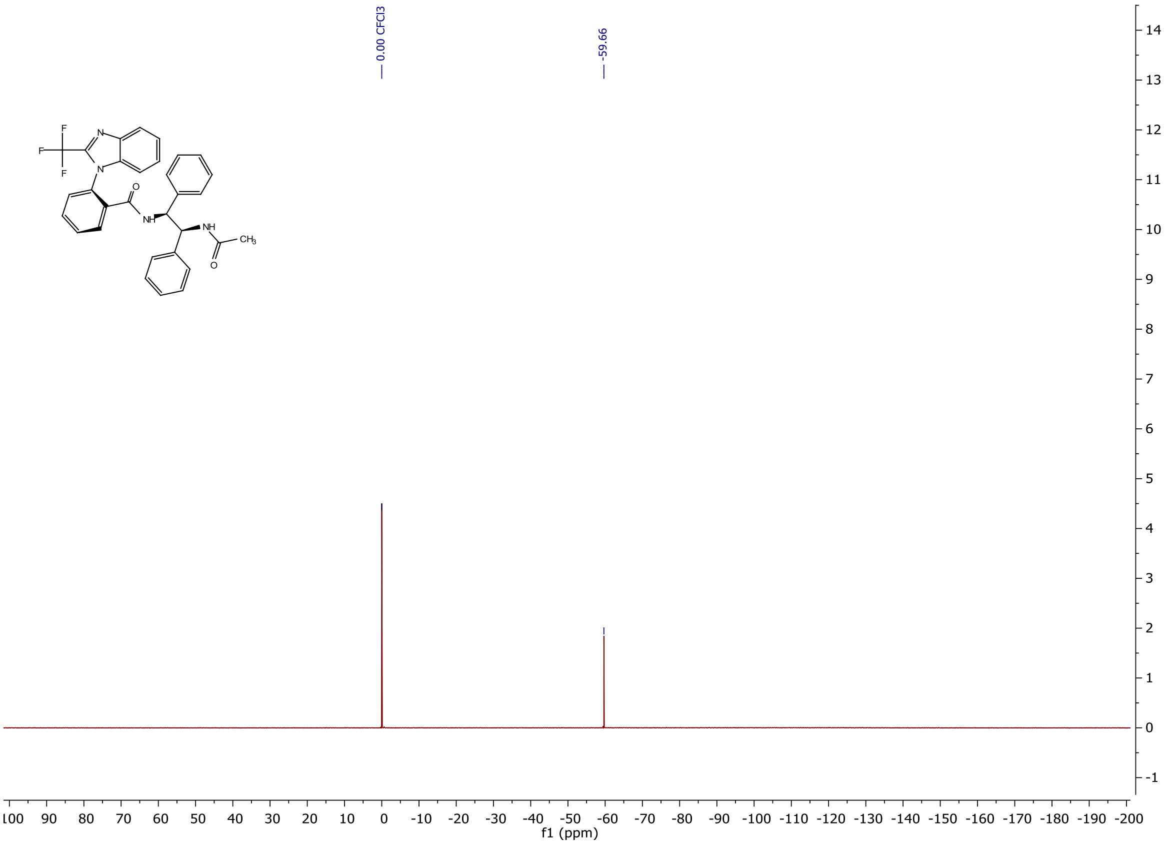

tert-Butyl ((1*S*,2*S*)-1,2-diphenyl-2-(2-((*P*)-2-(trifluoromethyl)-1*H*-benzo[*d*]imidazol-1-yl)benzamido)ethyl)carbamate **(*P*)-51**

^{19}F NMR (376 MHz, $CDCl_3$)

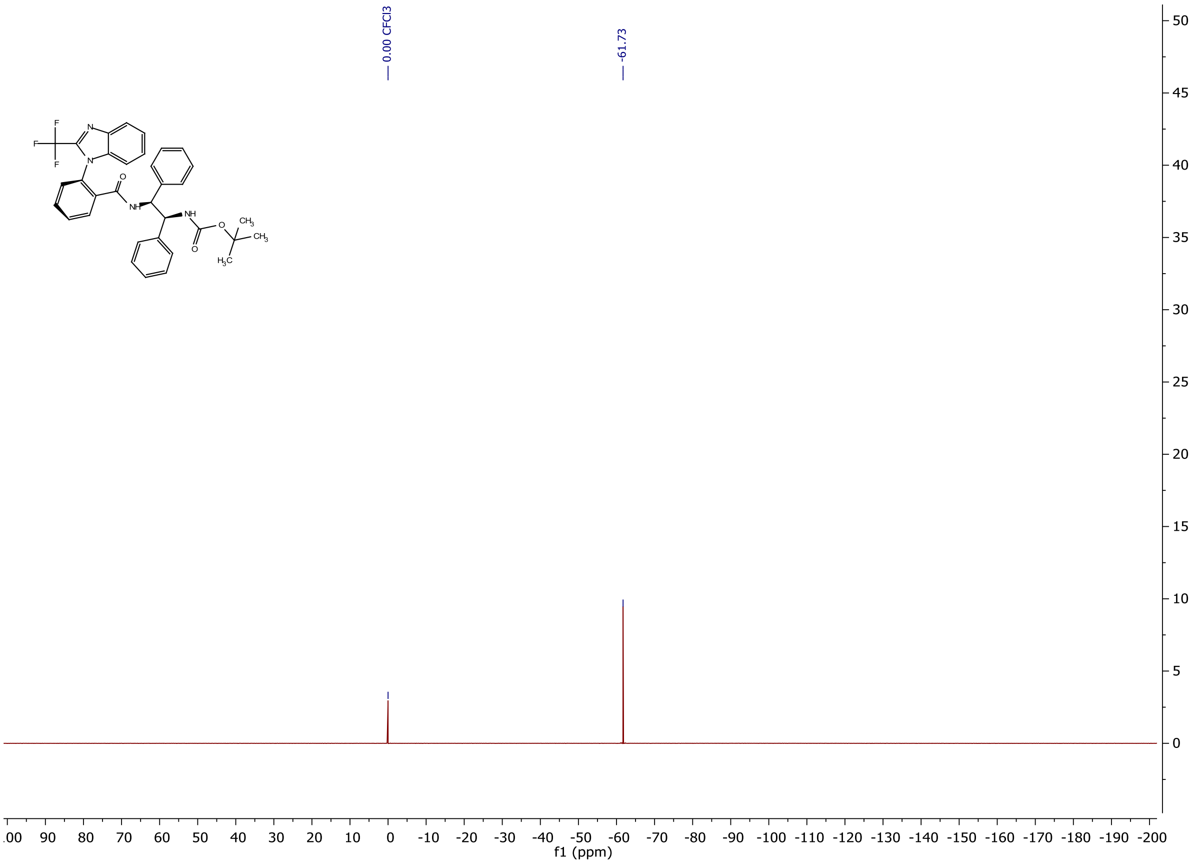

^{19}F NMR (376 MHz, DMSO-d_6)

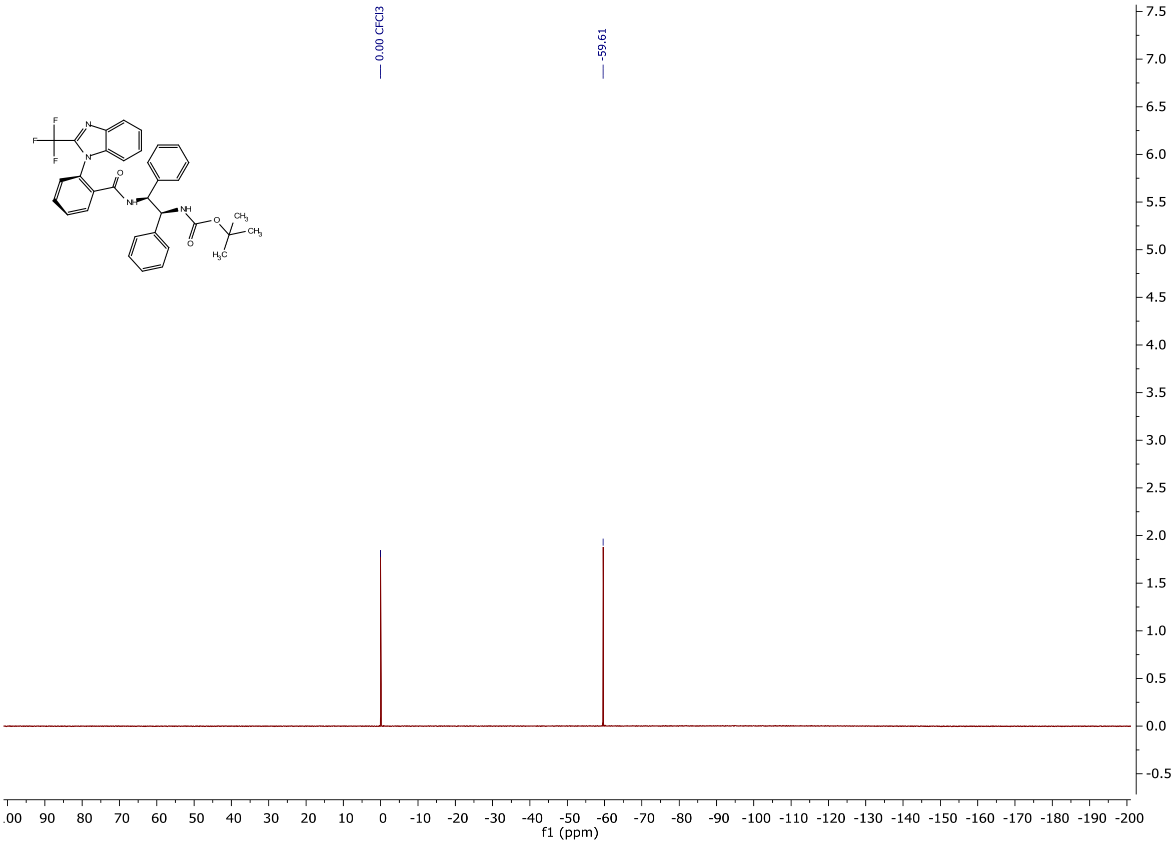

tert-Butyl ((1*S*,2*S*)-1,2-diphenyl-2-(2-((*M*)-2-(trifluoromethyl)-1*H*-benzo[*d*]imidazol-1-yl)benzamido)ethyl)carbamate **(*M*)-51**

^{19}F NMR (376 MHz, $CDCl_3$)

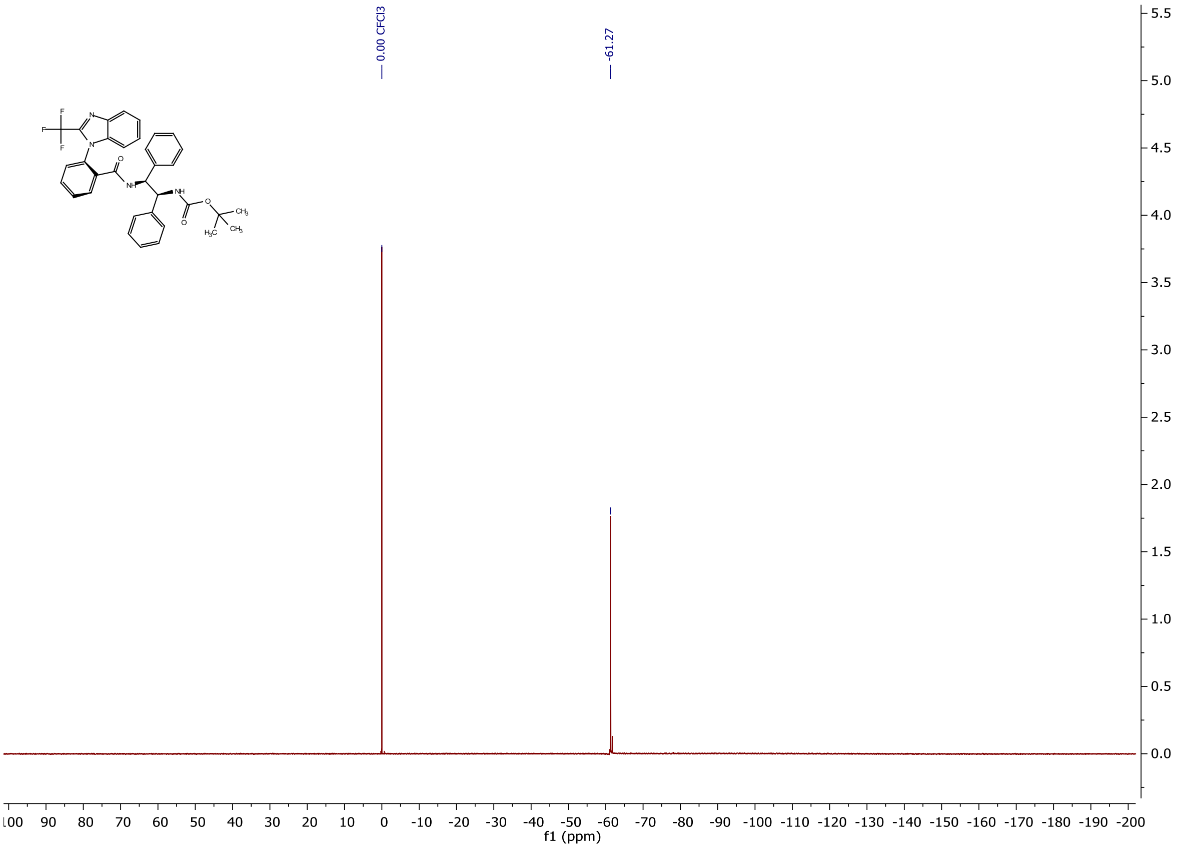

^{19}F NMR (376 MHz, DMSO-d_6)

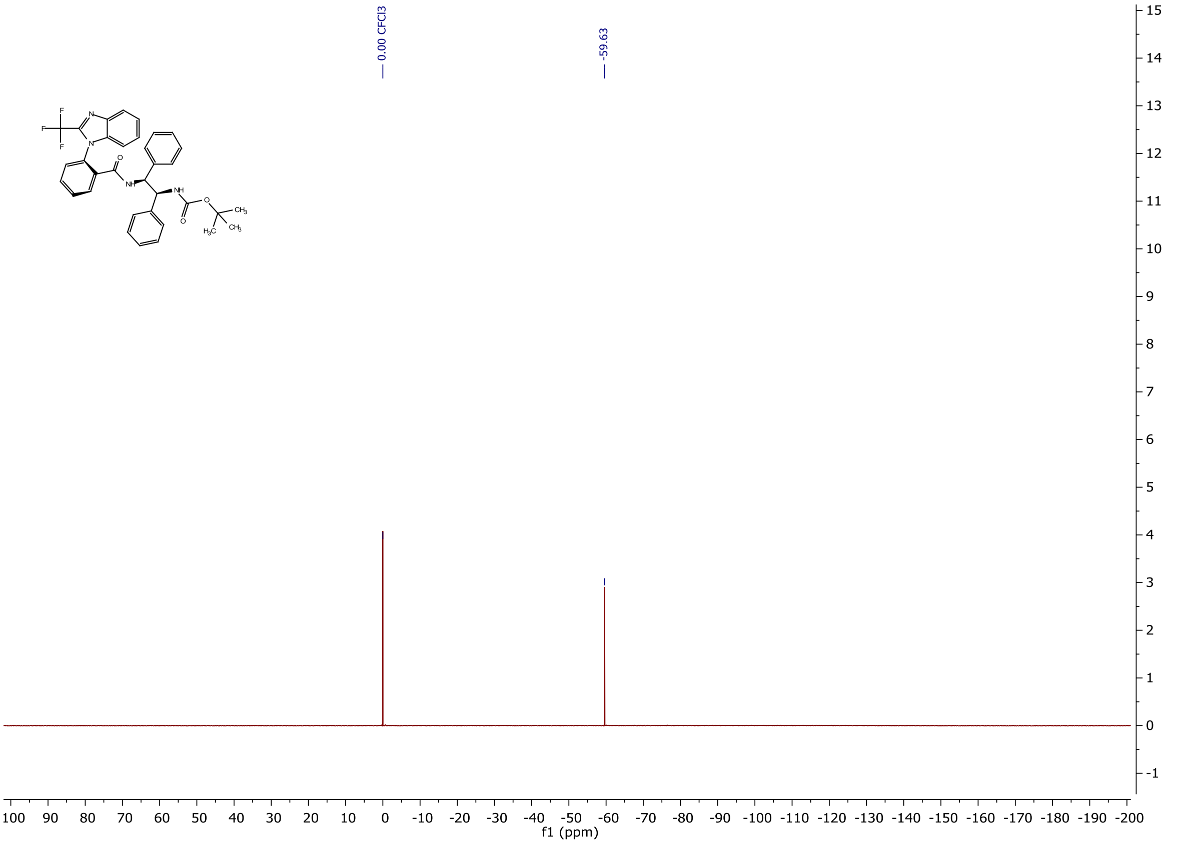

Evaluation of ^{19}F NMR chemical shifts of a mixture of TBBA amides 34

A mixture of **(*P*)-34** and **(*M*)-34** in a molar ratio 2:1, respectively.

^{19}F NMR (376 MHz, $CDCl_3$)

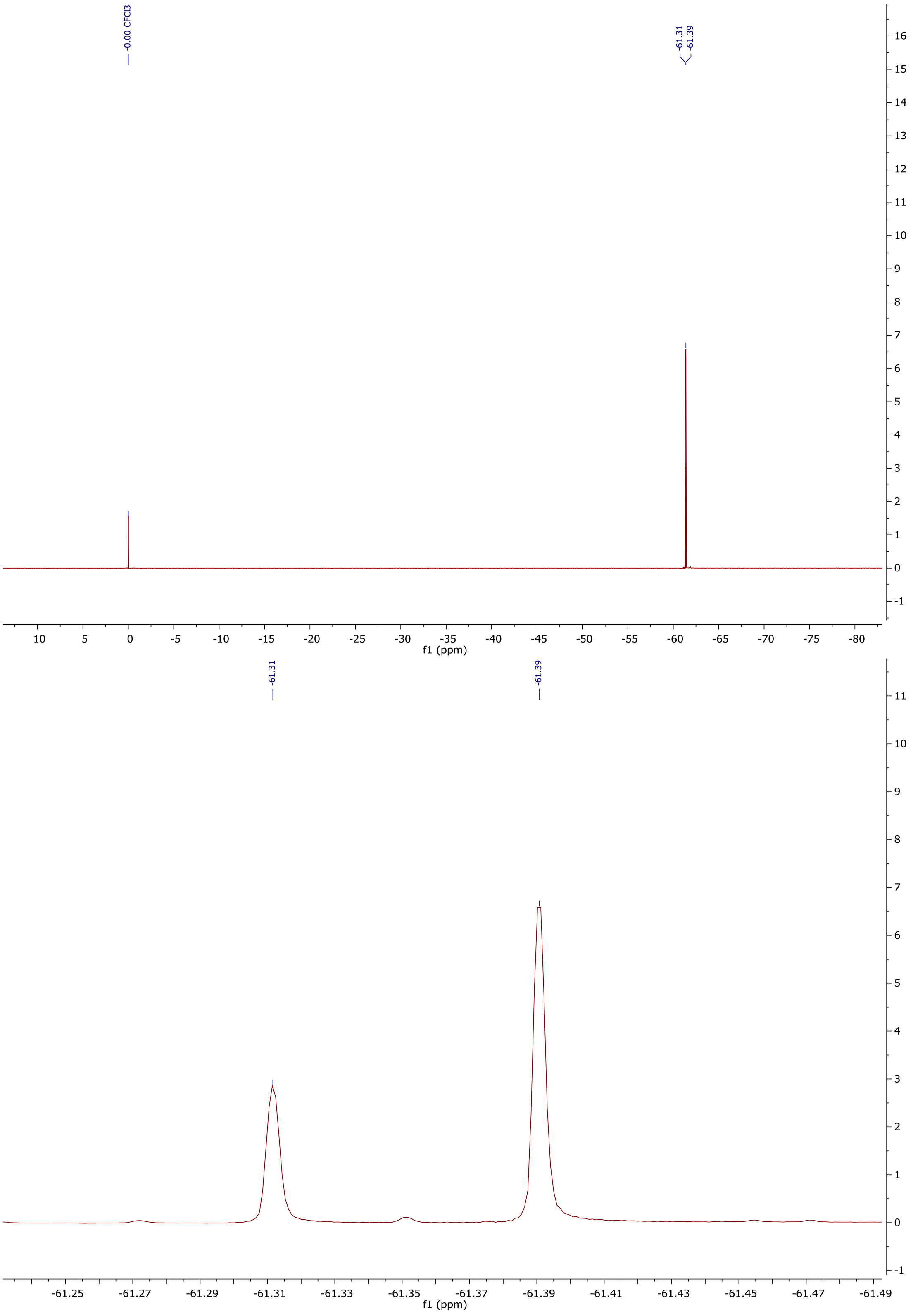

Synthesis of starting materials

(*R*)-2-Methyl-1-((4-nitrophenyl)sulfonyl)aziridine (**52)**

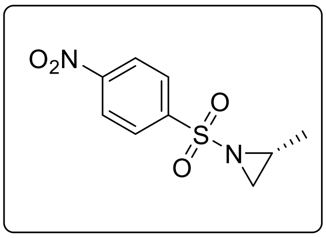

The procedure was inspired by the literature. [1]

D – alaninol (9.98 mmol, 1 equiv, 795 µL) was dissolved in a mixture of CH_2Cl_2/pyridyne (2:1, 100 mL), and 4-nitrobenzenesulfonyl chloride (29.95 mmol, 3 equiv, 6.6 g) was added. The reaction was stirred at room temperature for 16 hours. After completion, the reaction mixture was evaporated under reduced pressure, and the residue was dissolved in dichloromethane (100 mL) and extracted with 10 % (v/v) HCl (4 x 100 mL), 2 M KOH (7 x 100 mL), and brine (1 x 100 mL). The organic layer was dried over $MgSO_4$ and evaporated under reduced pressure. The residue was purified by column chromatography (column dimensions: 10 cm × 6 cm, hexane/EtOAc 6:1), yielding 1.64 g (67 %) of a yellow solid. **^{1}H NMR** (400 MHz, $CDCl_3$) δ (ppm) 8.41 – 8.37 (m, 2H), 8.18 – 8.13 (m, 2H), 3.05 – 2.90 (m, 1H), 2.73 (d, *J* = 7.0 Hz, 1H), 2.13 (d, *J* = 4.7 Hz, 1H), 1.30 (d, *J* = 5.6 Hz, 3H); **^{13}C{^{1}H} NMR** (101 MHz, $CDCl_3$) δ (ppm) 150.8, 144.5, 129.2, 124.4, 37.0, 35.6, 16.9; **HRMS** (ESI) m/z $[M + H]^+$ Calcd for $C_9H_{11}N_2O_4S$ 443.0434; Found 443.0437; $[\alpha]_d^{22}$ = -40.00° (c = 0.10 $CHCl_3$).

(*R*)-*N*-(1-Methoxypropan-2-yl)-4-nitrobenzenesulfonamide (**53)**

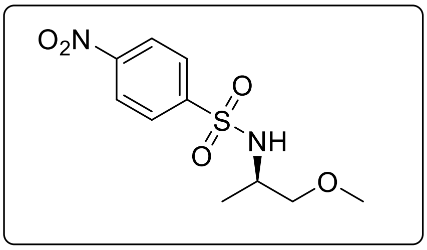

(*R*)-2-Methyl-1-((4-nitrophenyl)sulfonyl)aziridine (4.12 mmol, 1 equiv, 1 g, **52**) was dissolved in methanol (50 mL) and sodium methoxide (12.36 mmol, 3 equiv, 0.67 g) was added. The reaction was stirred at room temperature for 16 hours. After completion, the reaction was evaporated under reduced pressure, and the residue was dissolved in dichloromethane (50 mL) and extracted with 10 % (v/v) HCl (3 x 30 mL) and brine (1 x 30 mL). The organic layer was dried over $MgSO_4$ and evaporated under reduced pressure. The residue was purified by column chromatography (column dimensions: 10 cm × 6 cm, hexane/EtOAc 6:1), yielding 0.81 g (71 %) of a yellow solid. **^{1}H NMR** (400 MHz, $CDCl_3$) δ (ppm) 8.38 – 8.33 (m, 2H), 8.10 – 8.04 (m, 2H), 5.05 (d, *J* = 7.0 Hz, 1H), 3.59 – 3.50 (m, 1H), 3.27 – 3.18 (m, 5H), 1.14 (d, *J* = 6.7 Hz, 3H); **^{13}C{^{1}H} NMR** (101 MHz, $CDCl_3$) δ (ppm) 150.1, 147.1, 128.4, 124.4, 75.5, 59.0, 50.0, 18.6; **HRMS** (ESI) m/z $[M + H]^+$ Calcd for $C_{10}H_{13}N_2O_5S$ 275.0696; Found 275.0696; $[\alpha]_d^{22}$ = -5.05° (c = 0.33 $CHCl_3$).

(*R*)-1-Methoxypropan-2-amine (**54**)

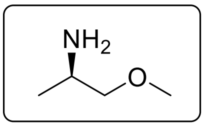

(*R*)-*N*-(1-Methoxypropan-2-yl)-4-nitrobenzenesulfonamide (3.57 mmol, 1 equiv, 0.75 g, **53**) was dissolved in acetonitrile (40 mL). Thiophenol (12.4 mmol, 3 equiv, 1.1 mL) and potassium carbonate (12.5 mmol, 3.5 equiv, 1.72 g) were added subsequently. The reaction was stirred at 50 °C for 10 hours. After 10 hours, the reaction mixture was allowed to cool to room temperature, and concentrated HCl was added dropwise until the pH reached 1. The acetonitrile was evaporated under reduced pressure, and the residue was dissolved in dichloromethane (45 mL) and extracted with water (3 x 25 mL). The aqueous layers were combined, the pH was adjusted to above 10 using saturated K_2CO_3 solution, and the mixture was extracted with diethyl ether (4 x 20 mL). The organic layer was dried over $MgSO_4$ and evaporated slowly at room temperature under reduced pressure (>400 mbar), yielding 84 mg (27 %) of a yellow oil, which was immediately applied to the following reaction due to stability issues. **^{1}H NMR** (400 MHz, DMSO-*d*$_6$) δ (ppm) 3.5 – 3.3 (m, 3H), 3.3 (s, 3H), 1.2 (d, *J* = 6.5 Hz, 3H); **HRMS** (ESI) m/z $[M + H]^+$ Calcd. for $C_4H_{12}NO$ 90.0913; Found 90.0919.

(*S*)-2-((4-Methylphenyl)sulfonamido)-3-phenylpropyl 4-methylbenzenesulfonate (**55**)

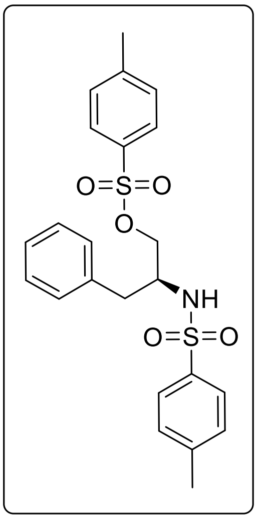

The procedure was inspired by the literature. [2]

(*S*)-2-Amino-3-phenylpropan-1-ol (17.55 mmol, 1 equiv, 2.7 g) was dissolved in pyridine (25 mL), and the solution was cooled to 5 °C in an ice bath. Under stirring, a cooled solution of *p*-toluenesulfonyl chloride (46.55 mmol, 2.65 equiv, 8.85 g) dissolved in pyridine (25 mL) was added dropwise over 10 minutes. The reaction was allowed to warm to room temperature and stirred for 24 hours. The reaction mixture was poured onto ice (200 g) and extracted with ethyl acetate (5 x 50 mL). The combined organic phases were washed with 1 M HCl (5 x 100 mL), water (3 x 100 mL), and brine (1 x 100 mL), dried over $MgSO_4$, and evaporated under reduced pressure. The residue was dissolved in benzene (25 mL), and activated carbon (150 mg) was added. The mixture was heated to reflux for 10 minutes and filtered. The filtrate was covered with heptane (50 mL), causing the mixture to turn cloudy white. The product was crystallized in the freezer for 3 days. The crystals were filtered and purified by column chromatography (column dimensions: 10 cm × 10 cm, hexane/EtOAc 3:1), yielding 5.4 g (67 %) of a white solid. **^{1}H NMR** (500 MHz, $CDCl_3$) δ 7.77 – 7.73 (m, 2H), 7.54 – 7.51 (m, 2H), 7.35 (d, *J* = 8.2 Hz, 2H), 7.19 – 7.11 (m, 5H), 6.90 – 6.85 (m, 2H), 4.73 (d, *J* = 7.9 Hz, 1H), 3.98 (dd, *J* = 10.1, 3.5 Hz, 1H), 3.86 (dd, *J* = 10.1, 5.3 Hz, 1H), 3.57 (tdd, *J* = 8.9, 4.5, 2.6 Hz, 1H), 2.81 (dd, *J* = 13.9, 7.3 Hz, 1H), 2.67 (dd, *J* = 13.9, 7.0 Hz, 1H), 2.47 (s, 3H), 2.40 (s, 3H); **^{13}C{^{1}H} NMR** (126 MHz, $CDCl_3$) δ

145.4, 143.6, 136.9, 135.8, 132.4, 130.2, 129.8, 129.2, 128.9, 128.2, 127.1, 70.2, 53.7, 37.7, 21.8, 21.6.); **HRMS** (ESI) m/z $[M + H]^+$ Calcd for $C_{23}H_{26}NO_5S_2$ 460.1247; Found 460.1248; $[\alpha]_d^{22}$ = -53.64° (c = 0.33 $CHCl_3$). Spectral data in agreement with the literature.[3]

(*R*)-4-Methyl-*N*-(1-phenylpropan-2-yl)benzenesulfonamide (**56**)

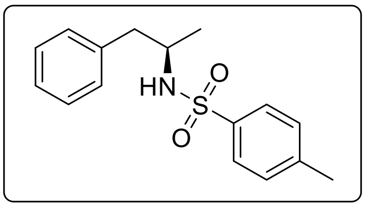

The procedure was inspired by the literature. [2]

$LiAlH_4$ (10.5 mmol, 5 equiv, 0.4 g) was suspended under an inert atmosphere in dry THF (10 mL). The slurry was cooled to 0 °C and a cooled solution of (*S*)-**55** (2.1 mmol, 1 equiv, 1 g) in dry THF (10 mL) was added dropwise over 10 min with stirring. The mixture was refluxed for 4 hours under continuous stirring, ensuring exclusion of moisture. Afterwards, it was cooled with an ice bath, and water (2 mL), 2 M NaOH (5 mL), and again water (10 mL) were added dropwise. The precipitated salts were filtered and washed with ethyl acetate (100 mL). The filtrate was washed with 1 M HCl (3 x 50 mL) and brine (1 x 50 mL), dried over $MgSO_4$, and evaporated under reduced pressure. The residue was purified by column chromatography (column dimensions: 5 cm × 10 cm, hexane/EtOAc 2:1), yielding 0.54 g (89 %) of a colourless oil. **^{1}H NMR** (500 MHz, $CDCl_3$) δ 7.65 – 7.61 (m, 2H), 7.24 – 7.17 (m, 5H), 7.04 – 6.99 (m, 2H), 4.49 (d, *J* = 7.3 Hz, 1H), 3.52 (dq, *J* = 13.1, 6.6 Hz, 1H), 2.70 (dd, *J* = 13.6, 6.4 Hz, 1H), 2.65 (dd, *J* = 13.6, 6.9 Hz, 1H), 2.41 (s, 3H), 1.09 (d, *J* = 6.6 Hz, 3H); **^{13}C{^{1}H} NMR** (126 MHz, $CDCl_3$) δ 143.2, 137.8, 137.3, 129.7, 129.5, 128.6, 127.1, 126.7, 51.0, 43.6, 21.6, 21.4; **HRMS** (ESI) m/z $[M + H]^+$ Calcd for $C_{16}H_{20}NO_2S$ 290.1209; Found 290.1216; $[\alpha]_d^{22}$ = -6.82° (c = 0.22 $CHCl_3$).

(*R*)-1-Phenylpropan-2-amine (**57**)

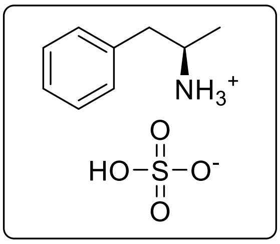

To (*R*)-**56** (2.59 mmol, 1 equiv, 0.75 g) was gradually added HBr (5 mL, 48 % in water) and phenol (7.77 mmol, 3 equiv, 0.73 g). The mixture was heated to 100 °C for 12 hours with stirring. After cooling to room temperature, the pH was adjusted to 10 using 2 M NaOH solution. The reaction mixture was washed with diethyl ether (4 x 30 mL), and the combined organic phases were dried over $MgSO_4$, and evaporated under reduced pressure. The residue was dissolved in diethyl ether (10 mL) and conc. sulfuric acid (1.0 mmol, 0.1 g) in ethanol (3 mL) was added dropwise. The resulting sulphate salt was filtered, washed with diethyl ether, and lyophilized, yielding 0.23 g (23 %) of a white solid. **^{1}H NMR** (400 MHz, D_2O) δ 7.59 – 7.33 (m, 5H), 3.68 (h, *J* = 7.4, 6.8 Hz, 1H), 2.99 (d, *J* = 7.1 Hz, 2H), 1.35 (d, *J* = 6.6 Hz, 3H); **^{13}C{^{1}H} NMR** (101 MHz, D_2O + DMSO-d_6 (20:1)) δ 137.7, 131.0, 130.6, 129.0, 50.7, 41.7, 19.1; **HRMS** (ESI) m/z $[M + H]^+$ Calcd for $C_9H_{14}N$ 136.1121; Found 136.1120; $[\alpha]_d^{22}$ = -14.29° (c = 0.14 H_2O).

tert-Butyl ((1*S*,2*S*)-2-amino-1,2-diphenylethyl)carbamate (**58**)

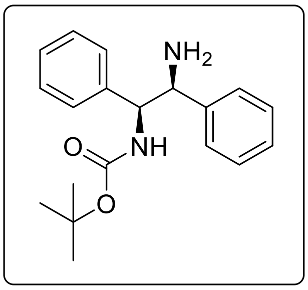

The procedure was inspired by the literature.[4]

TMSCl (20 mmol, 1 equiv, 2.52 mL) was added to MeOH (20 mL) pre-cooled to 0 °C, and the mixture was stirred under cooling for 20 minutes. While cooling, (1*S*,2*S*)-1,2-diphenylethane-1,2-diamine (20 mmol, 1 equiv, 4.26 g) was added, and the reaction mixture was gradually allowed to reach room temperature, where it was stirred for 20 minutes. Then, a solution of $(Boc)_2O$ (20 mmol, 1 equiv, 4.32 g) in MeOH (20 mL) was added dropwise for 15 minutes. The reaction mixture was stirred at room temperature for 5 hours, and the MeOH was evaporated under reduced pressure. Diethyl ether (50 mL) was added to the residue, and the mixture was filtered. The precipitate was then treated with diethyl ether (3 x 50 mL), 3 M NaOH (2 x 50 mL), and water (3 x 50 mL). The product was dried by lyophilization, yielding 5.26 g (84 %) of a light-yellow solid. **^{1}H NMR** (400 MHz, DMSO-d_6) δ 7.31 (d, *J* = 8.8 Hz, 1H), 7.24 – 7.10 (m, 10H), 4.63 (t, *J* = 7.7 Hz, 1H), 4.02 (d, *J* = 6.7 Hz, 1H), 1.85 (s, 2H), 1.35 – 1.06 (m, 9H); **^{13}C{^{1}H} NMR** (101 MHz, DMSO-d_6) δ 155.2, 143.5, 142.1, 127.7, 127.6, 127.2, 127.0, 126.4, 126.4, 77.8, 61.2, 60.0, 28.2; **HRMS** (ESI) m/z $[M + H]^+$ Calcd for $C_{19}H_{25}N_2O_2$ 313.1911; Found 313.1908; $[\alpha]_d^{22}$ = -50.53° (c = 0.19 $CHCl_3$). Spectral data in agreement with the literature.[4]

(1*S*,2*S*)-*N*[1],*N*[1]-Dimethyl-1,2-diphenylethane-1,2-diamine (**59**)

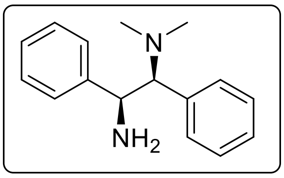

The procedure was inspired by the literature.[5]

Carbamate **58** (1.6 mmol, 1 equiv, 0.5 g) was dissolved in THF (25 mL). Paraformaldehyde (16 mmol, 10 equiv, 0.48 g) and Bu_2SnCl_2 (1.6 mmol, 1 equiv, 0.49 g) were added to the solution. The reaction mixture was stirred at room temperature for 20 minutes, and then phenylsilane (8 mmol, 5 equiv, 0.98 mL) was added dropwise. The reaction mixture was brought to reflux and stirred for 3 hours. Subsequently, it was cooled to room temperature, and THF was evaporated under reduced pressure. EtOAc (200 mL) and saturated NH_4Cl (200 mL) were added to the residue, and the mixture was extracted with EtOAc (3 x 200 mL). The organic phases were combined, dried over $MgSO_4$, and evaporated under reduced pressure. The residue was purified by column chromatography (column dimensions: 10 cm × 10 cm, CH_2Cl_2/MeOH 10:1). The oily product obtained was treated with a CH_2Cl_2/TFA solution (10:2, 10 mL), and the mixture was stirred at room temperature for 12 hours. The pH was adjusted to 12 using 2 M NaOH. The mixture was then extracted with CH_2Cl_2 (3 x 50 mL), dried over $MgSO_4$, and evaporated under reduced pressure, yielding 223 mg (58 %) of a colourless oil. **^{1}H NMR** (400 MHz, $CDCl_3$) δ 7.25 – 7.21 (m, 2H), 7.19 – 7.09 (m, 5H), 7.07 – 7.02 (m, 1H), 7.02 – 6.98 (m, 2H), 4.44 (d, *J* = 10.6 Hz, 1H), 3.69

(d, J = 10.6 Hz, 1H), 2.36 (br. s, 2H), 2.23 (s, 6H); **^{13}C{^{1}H} NMR** (101 MHz, $CDCl_3$) δ 143.0, 133.8, 130.0, 128.2, 128.2, 127.5, 127.1, 127.1, 75.2, 55.8, 41.1; **HRMS** (ESI) m/z $[M + H]^+$ Calcd for $C_{16}H_{21}N_2$ 241.1699; Found 241.1705.

(9*H*-Fluoren-9-yl)methyl (*S*)-(1-(diethylamino)-1-oxopropan-2-yl)carbamate (**60**)

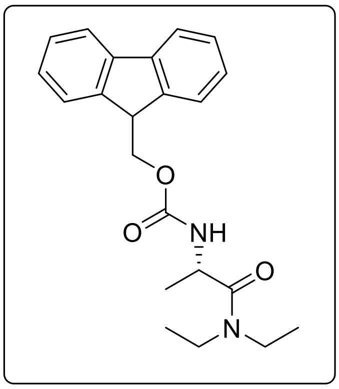

Fmoc-*L*-alanine (3.21 mmol, 1 equiv, 1 g) was dissolved in DMF (8 mL), and then diethylamine (3.53 mmol, 1.1 equiv, 0.36 mL), EDCI (6.42 mmol, 2 equiv, 1.23 g), and HOBt (6.42 mmol, 2 equiv, 0.98 g) were sequentially added. The reaction mixture was stirred for 12 hours at room temperature. Subsequently, EtOAc (40 mL) was added, and the mixture was extracted successively with 1 M HCl (3 x 30 mL), 10% K_2CO_3 (3 x 30 mL), and brine (1 x 30 mL). The organic phase was dried over $MgSO_4$ and evaporated under reduced pressure. The residue was purified by column chromatography (column dimensions: 5 cm × 10 cm, EtOAc/CH_2Cl_2/MeOH 5:2:0.1). The product was lyophilized, yielding 0.89 g (76 %) of a white solid. **^{1}H NMR** (400 MHz, DMSO-d_6) δ 7.88 (d, J = 7.5 Hz, 2H), 7.73 (t, J = 7.4 Hz, 2H), 7.57 (d, J = 7.9 Hz, 1H), 7.41 (t, J = 7.3 Hz, 2H), 7.32 (t, J = 7.4 Hz, 2H), 4.42 (p, J = 6.9 Hz, 1H), 4.30 – 4.14 (m, 3H), 3.43 – 3.28 (m, 3H), 3.13 (dq, J = 13.8, 6.9 Hz, 1H), 1.19 (d, J = 6.9 Hz, 3H), 1.14 (t, J = 7.0 Hz, 3H), 1.00 (t, J = 7.0 Hz, 3H); **^{13}C{^{1}H} NMR** (101 MHz, DMSO-d_6) δ 171.4, 155.6, 143.8, 143.8, 140.7, 127.6, 127.6, 127.0, 125.3, 125.3, 120.0, 65.6, 46.6, 46.3, 41.0, 17.9, 14.3, 12.9; **HRMS** (ESI) m/z $[M + H]^+$ Calcd for $C_{21}H_{27}N_2O$ 323.2118; Found 323.2119; $[\alpha]_d^{22}$ = -33.57 ° (c = 0.42 $CHCl_3$).

(4*R*,5*R*)-4-(4-Aminophenyl)-2,2-dimethyl-1,3-dioxan-5-amine (**61**)

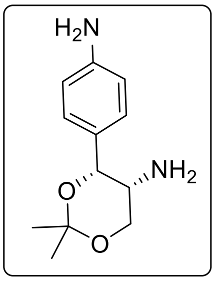

The procedure was inspired by the literature.[6]

(1*R*,2*R*)-2-Amino-1-(4-nitrophenyl)propane-1,3-diol (3 mmol, 1 equiv, 0,64 g) was suspended in MeOH (20 mL). Ethyl formate (3.3 mmol, 1 equiv, 0.27 mL) and NaOMe (0.6 mmol, 0.1 equiv, 32 mg) were added sequentially at room temperature. The mixture was stirred at room temperature for 6 hours until a fine yellow solution was formed. MeOH was evaporated under reduced pressure. The residue was dissolved in acetone (20 mL), followed by the addition of *p*-tolylsulfonic acid monohydrate (0.9 mmol, 0.15 equiv, 0.17 g) and 2,2-dimethoxypropane (15 mmol, 5 equiv, 1.8 mL). The reaction mixture was stirred at room temperature for 2 hours. Subsequently, the acetone was evaporated under reduced pressure. The resulting residue was dissolved in CH_2Cl_2 (100 mL) and washed with $NaHCO_3$ (3 x 30 mL). The aqueous phases were combined and extracted with CH_2Cl_2 (3 x 30 mL). The organic phases were then combined, dried over $MgSO_4$, and evaporated under reduced pressure. The fine yellow solid was suspended in hydrazine hydrate (10 mL) and heated to 100 °C. It

was stirred at this temperature for 2 hours. The reaction mixture was cooled to room temperature, and water (20 mL) was added. The reaction mixture was extracted with CH_2Cl_2 (3 x 30 mL). The organic phases were combined, dried over $MgSO_4$, and evaporated under reduced pressure. The residue was purified by column chromatography (column dimensions: 7 cm × 10 cm, CH_2Cl_2/MeOH 8:1). The product was lyophilized, yielding 312 mg (47 %) of a yellow oil. **^{1}H NMR** (400 MHz, $CDCl_3$) δ 7.12 – 7.07 (m, 2H), 6.71 – 6.65 (m, 2H), 4.99 (s, 1H), 4.26 (dd, *J* = 11.7, 2.3 Hz, 1H), 3.88 (dd, *J* = 11.7, 1.8 Hz, 1H), 2.65 (q, *J* = 1.9 Hz, 1H), 1.53 (s, 3H), 1.51 (s, 3H); **^{13}C{^{1}H} NMR** (101 MHz, $CDCl_3$) δ 145.8, 129.6, 126.9, 115.2, 99.2, 73.7, 66.0, 49.9, 29.9, 18.7; **HRMS** (ESI) m/z $[M + H]^+$ Calcd for $C_{12}H_{19}N_2O_2$ 223.1441; Found 223.1437; $[\alpha]_d^{22}$ = -42.06° (c = 0.73 $CHCl_3$). Spectral data in agreement with the literature.[7]

(4*R*,5*R*)-2,2-Dimethyl-4-(4-nitrophenyl)-1,3-dioxan-5-amine (**62**)

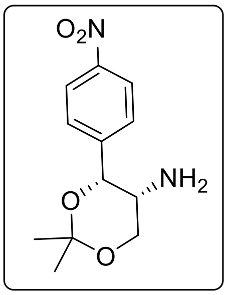

The procedure was inspired by the literature.[6]

(1*R*,2*R*)-2-Amino-1-(4-nitrophenyl)propane-1,3-diol (3 mmol, 1 equiv, 0.64 g) was suspended in MeOH (20 mL). Ethyl formate (3.3 mmol, 1 equiv, 0.27 mL) and NaOMe (0.6 mmol, 0.1 equiv, 32 mg) were added sequentially at room temperature. The mixture was stirred at room temperature for 6 hours until a fine yellow solution was formed. MeOH was evaporated under reduced pressure. The residue was dissolved in acetone (20 mL), followed by the addition of *p*-tolylsulfonic acid monohydrate (0.9 mmol, 0.15 equiv, 0.17 g) and 2,2-dimethoxypropane (15 mmol, 5 equiv, 1.8 mL). The reaction mixture was stirred at room temperature for 2 hours. Subsequently, the acetone was evaporated under reduced pressure. The resulting residue was dissolved in CH_2Cl_2 (100 mL) and washed with $NaHCO_3$ (3 x 30 mL). The aqueous phases were combined and extracted with CH_2Cl_2 (3 x 30 mL). The organic phases were combined, dried over $MgSO_4$, and evaporated under reduced pressure. The fine yellow solid was dissolved acetonitrile (5 mL), and hydrazine hydrate (5 mL) was added. The reaction mixture was stirred at 90 °C for 3 hours. When HPLC analysis showed almost complete conversion to the product (80 %), the reaction was quenched by adding water (10 mL) and cooling to room temperature due to the formation of by-products. The reaction mixture was extracted with diethyl ether (5 x 30 mL). The organic phases were combined, dried over $MgSO_4$, and evaporated under reduced pressure. The residue was purified by column chromatography (column dimensions: 5 cm × 10 cm, EtOAc/CH_2Cl_2/MeOH 4:1:0.1) to obtain 437 mg (58 %) of a yellow solid. **^{1}H NMR** (400 MHz, $CDCl_3$) δ 8.28 – 8.18 (m, 2H), 7.55 – 7.47 (m, 2H), 5.22 – 5.13 (m, 1H), 4.32 (dd, *J* = 11.8, 2.2 Hz, 1H), 3.88 (dd, *J* = 11.8, 1.8 Hz, 1H), 2.85 (q, *J* = 2.0 Hz, 1H), 1.56 (s, 6H), 1.32 (br. s, 2H); **^{13}C{^{1}H} NMR** (101 MHz, $CDCl_3$) δ 147.3, 126.8, 123.7, 99.7, 73.6, 66.5, 49.6, 29.8, 18.7; **HRMS** (ESI) m/z $[M + H]^+$ Calcd for $C_{12}H_{17}N_2O_4$ 253.1183; Found 253.1179; $[\alpha]_d^{22}$ = -72.35° (c = 0.34 $CHCl_3$).

tert-Butyl ((1*R*,2*R*)-2-aminocyclohexyl)carbamate (**63**)

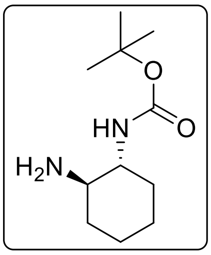

The procedure was inspired by the literature.[8]

While cooling with an ice bath at 0 °C, a solution of 37 % HCl (21.54 mmol, 1.05 equiv, 1,8 mL) in MeOH (15 mL) was added dropwise over 10 minutes to a solution of (1*R*,2*R*)-cyclohexane-1,2-diamine (20.5 mmol, 1 equiv, 2.34 g) in MeOH (35 mL). The mixture was allowed to warm to room temperature and stirred for 1 hour. Afterwards, it was cooled again to 0 °C, and a cooled solution of di-*tert*-butyl dicarbonate (30.75 mmol, 1.5 equiv, 6.66 g) in MeOH (20 mL) was added dropwise. The reaction mixture was stirred at room temperature for 6 hours. The MeOH was evaporated under reduced pressure, and water (40 mL) was added to the residue. The mixture was filtered, and the precipitate was washed with water (3 x 15 mL). The pH of the filtrate was adjusted to 10-12 with 2 M NaOH. The mixture was extracted with CH_2Cl_2 (5 x 30 mL), and the organic phases were combined, dried over $MgSO_4$, and evaporated under reduced pressure to obtain 3.64 g (83 %) of a white solid. **^{1}H NMR** (400 MHz, DMSO-d_6) δ 6.61 (d, *J* = 7.6 Hz, 1H), 2.88 (q, *J* = 9.9, 7.9 Hz, 1H), 2.31 (td, *J* = 10.6, 4.0 Hz, 1H), 1.80 – 1.72 (m, 2H), 1.65 – 1.52 (m, 2H), 1.38 (s, 9H), 1.24 – 0.97 (m, 4H); **^{13}C{^{1}H} NMR** (101 MHz, DMSO-d_6) δ 155.5, 77.3, 57.1, 53.7, 34.5, 32.1, 28.3, 24.9, 24.7; **HRMS** (ESI) m/z $[M + H]^+$ Calcd for $C_{11}H_{23}N_2O_2$ 215.1754; Found 215.1755; $[\alpha]_d^{22}$ = -35.50° (c = 0.33 MeOH). Spectral data in agreement with the literature.[9]

Starting Materials: Copies of ^{1}H and ^{13}C NMR Spectra

(*R*)-2-Methyl-1-((4-nitrophenyl)sulfonyl)aziridine (**52**)

^{1}H NMR (400 MHz, $CDCl_3$)

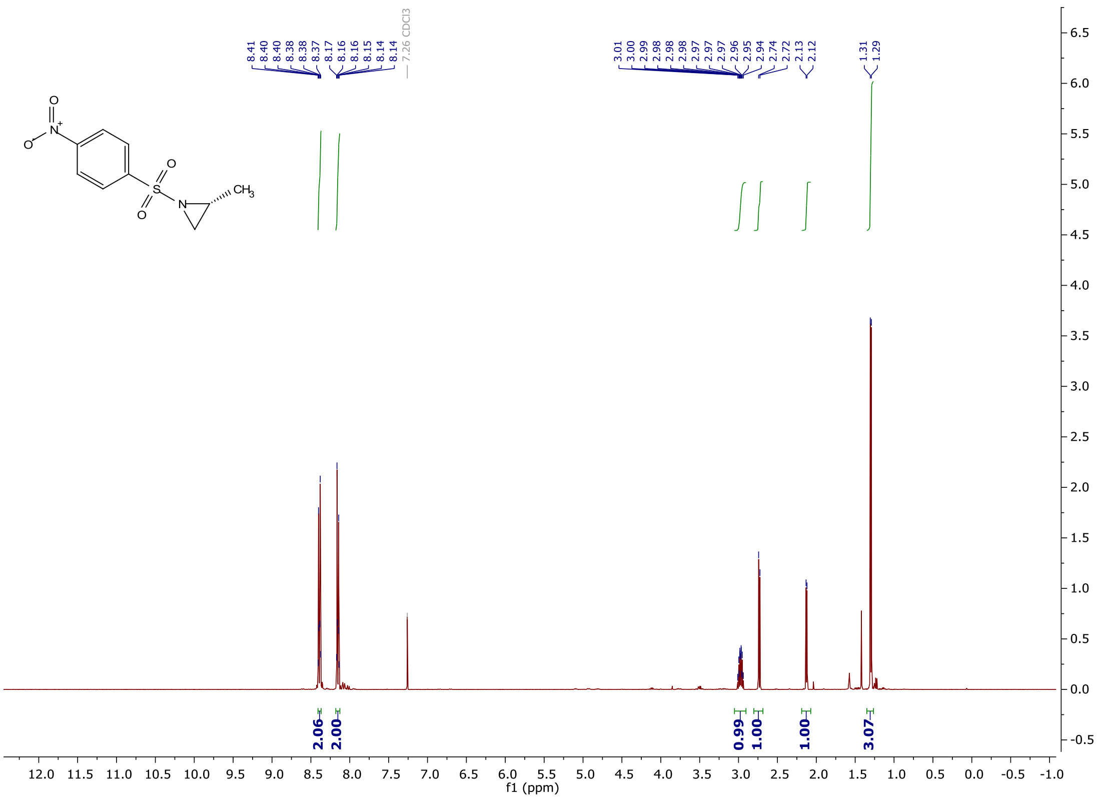

^{13}C{^{1}H} NMR (101 MHz, $CDCl_3$)

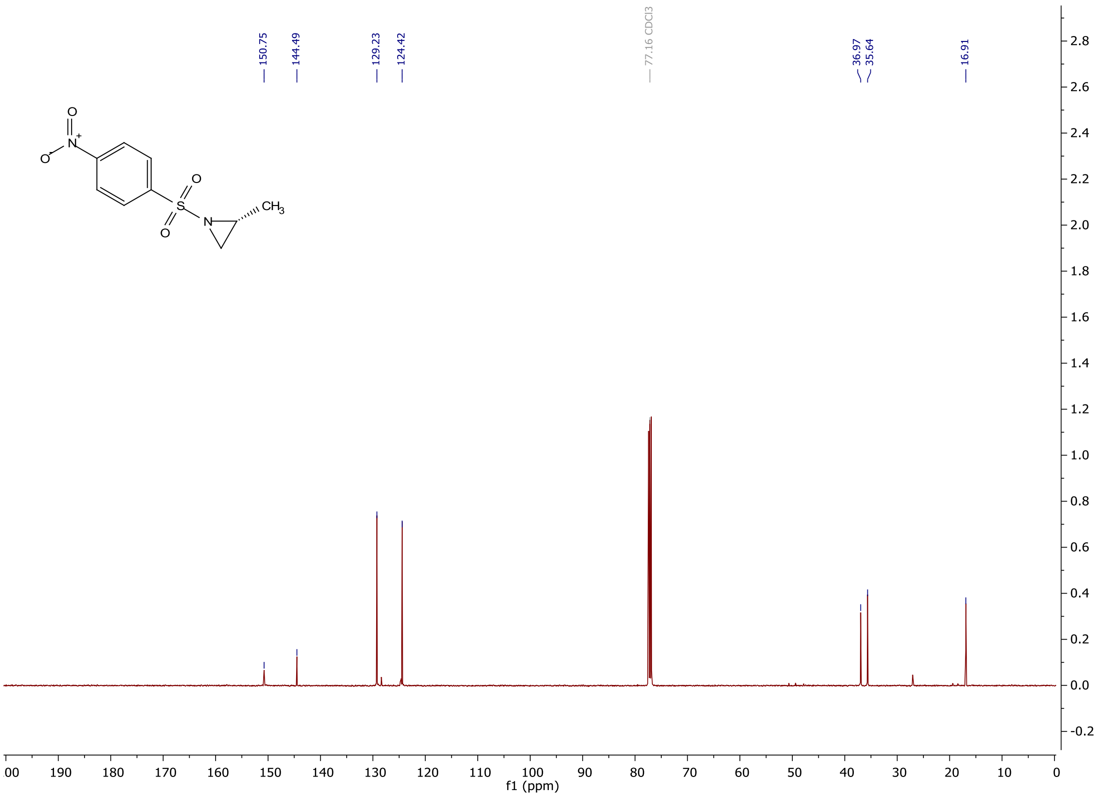

(*R*)-*N*-(1-Methoxypropan-2-yl)-4-nitrobenzenesulfonamide (**53**)

^{1}H NMR (400 MHz, $CDCl_3$)

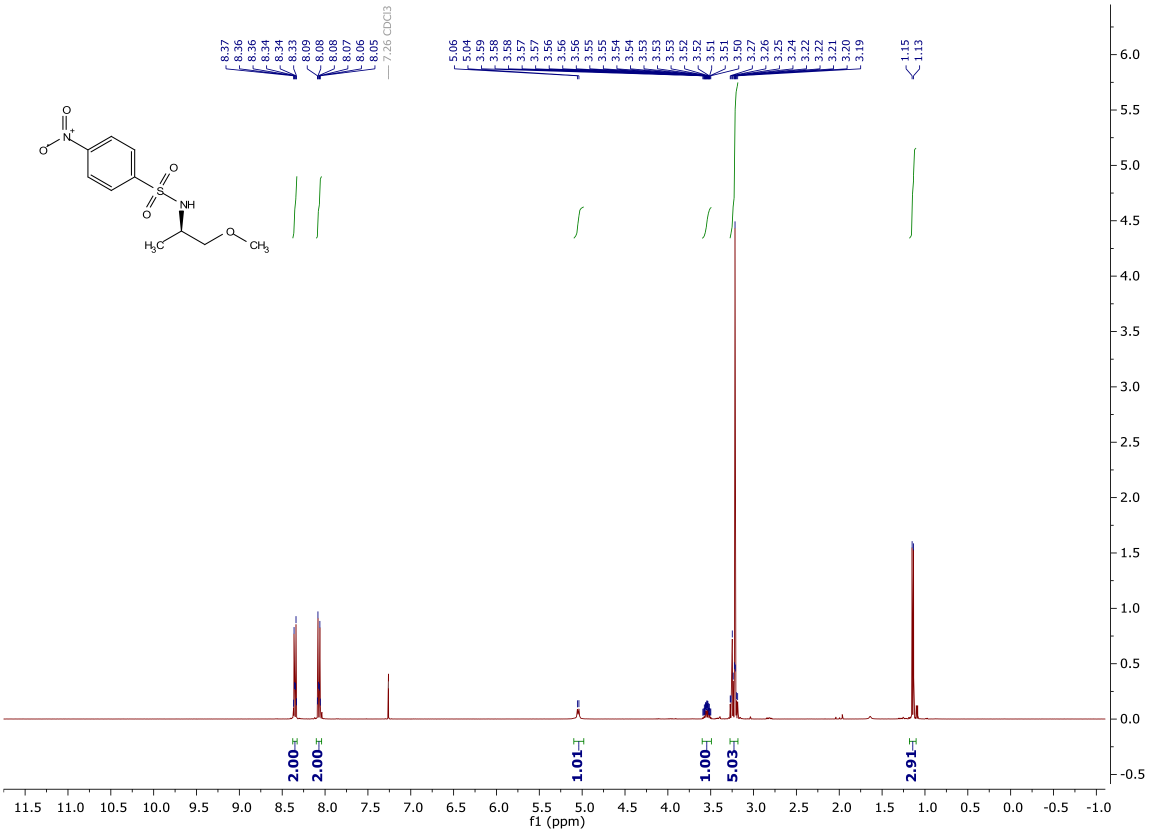

^{13}C{^{1}H} NMR (101 MHz, $CDCl_3$)

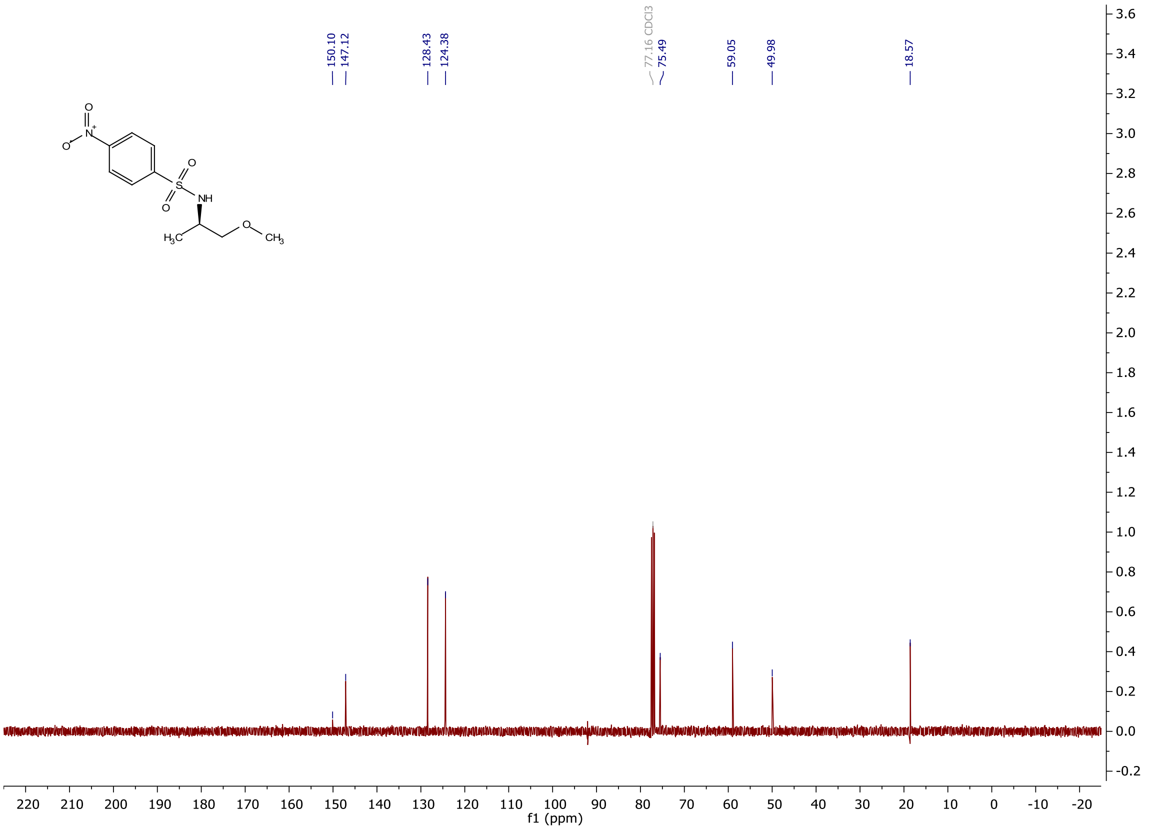

(*R*)-1-Methoxypropan-2-amine (**54**)

^{1}**H NMR** (400 MHz, $CDCl_3$)

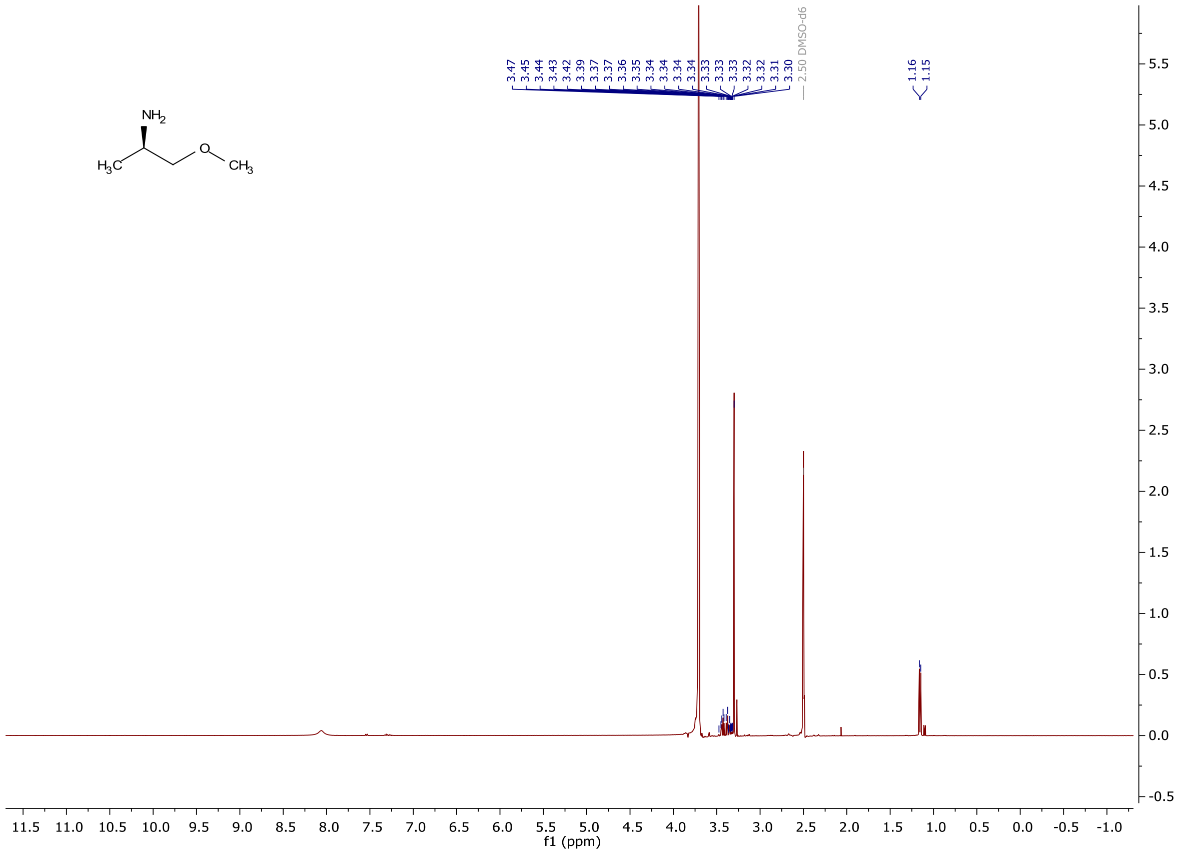

(*S*)-2-((4-Methylphenyl)sulfonamido)-3-phenylpropyl 4-methylbenzenesulfonate (**55**)

^{1}H NMR (500 MHz, $CDCl_3$)

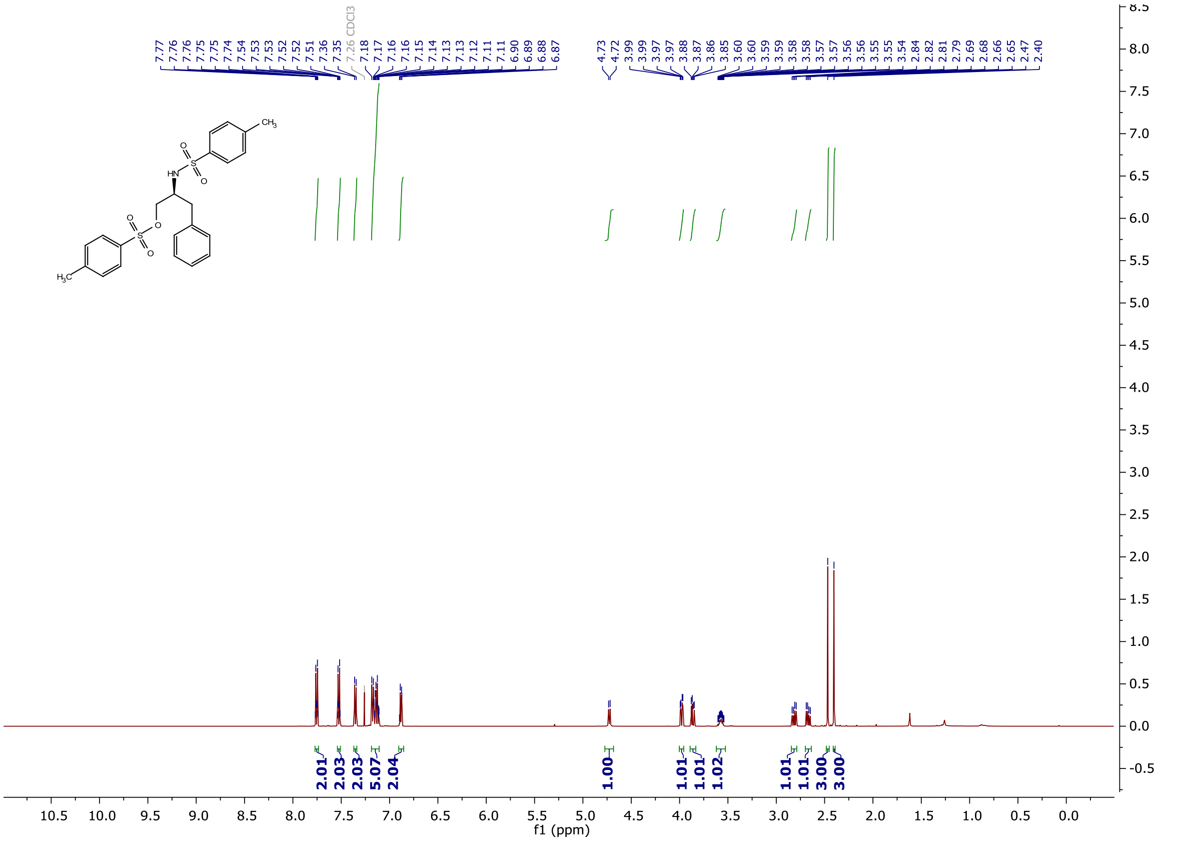

^{13} C{^{1}H} NMR (126 MHz, $CDCl_3$)

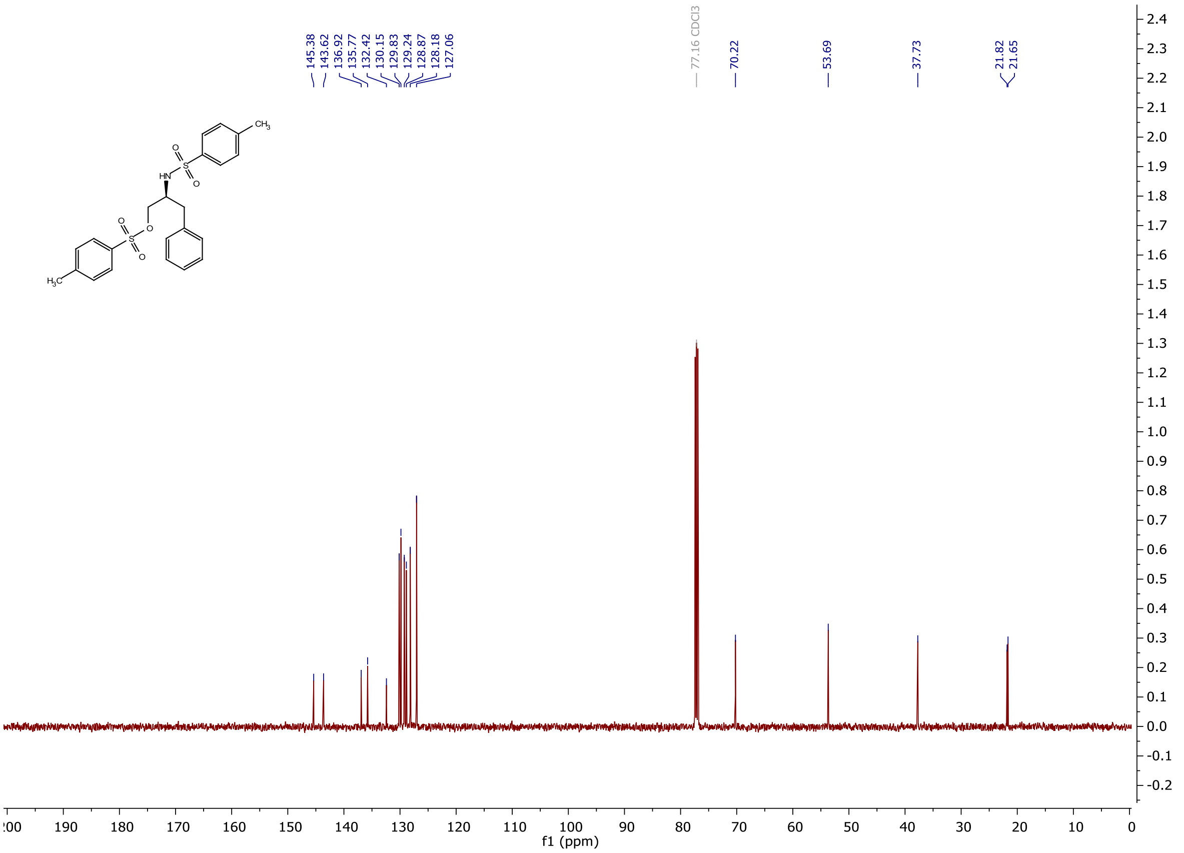

(*R*)-4-Methyl-N-(1-phenylpropan-2-yl)benzenesulfonamide (**56**)

^{1}**H NMR** (500 MHz, $CDCl_3$)

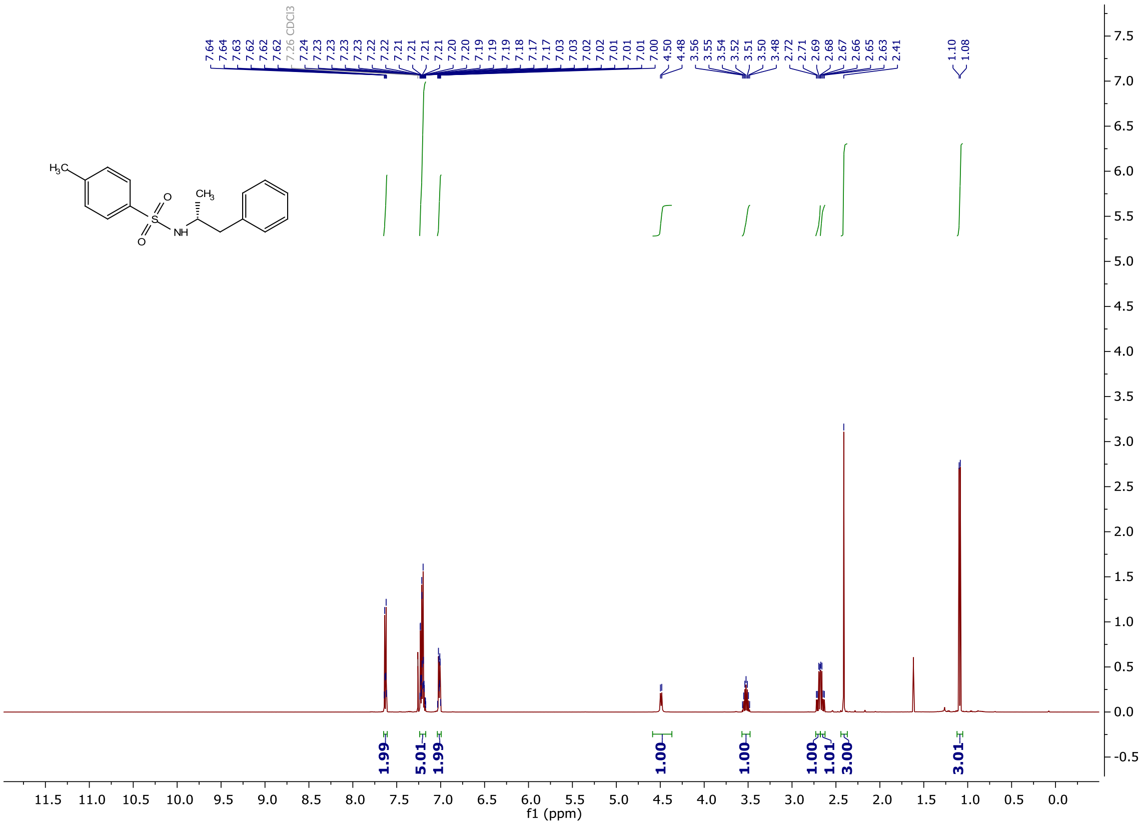

^{13}**C{^{1}H} NMR** (126 MHz, $CDCl_3$)

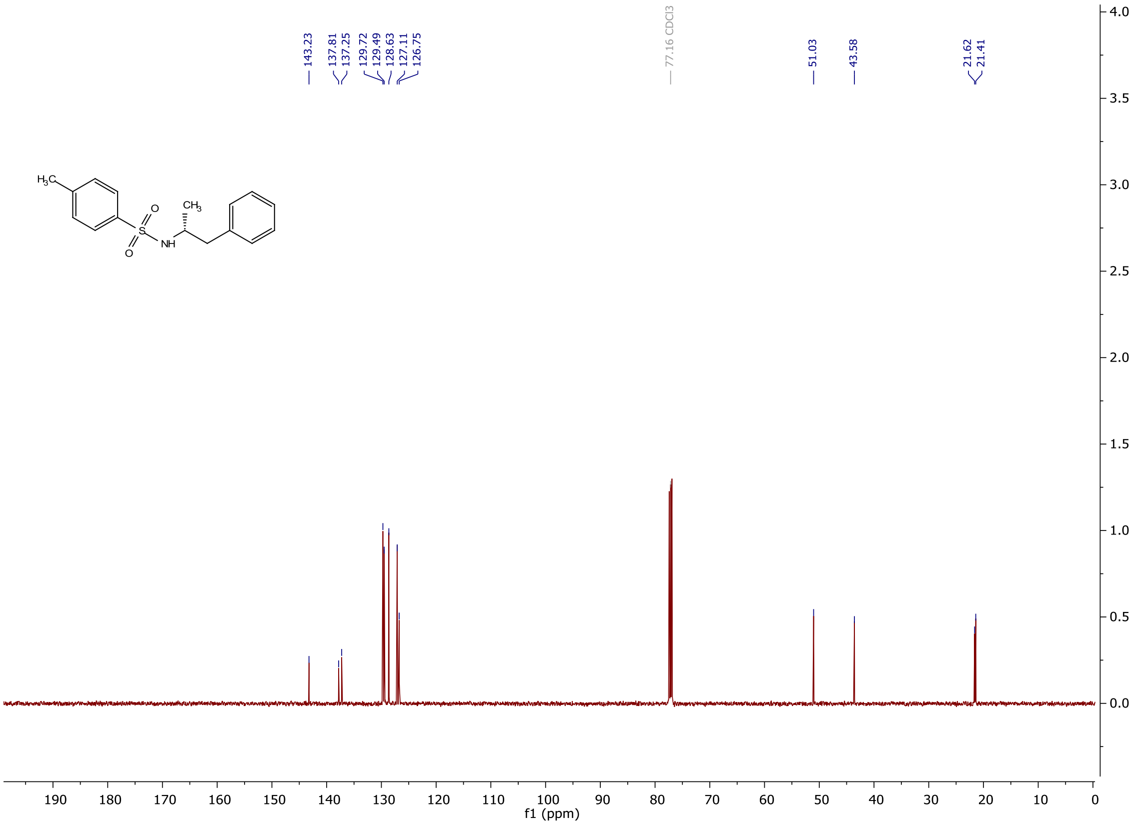

(*R*)-1-Phenylpropan-2-amine (**57**)

^{1}H NMR (400 MHz, D_2O)

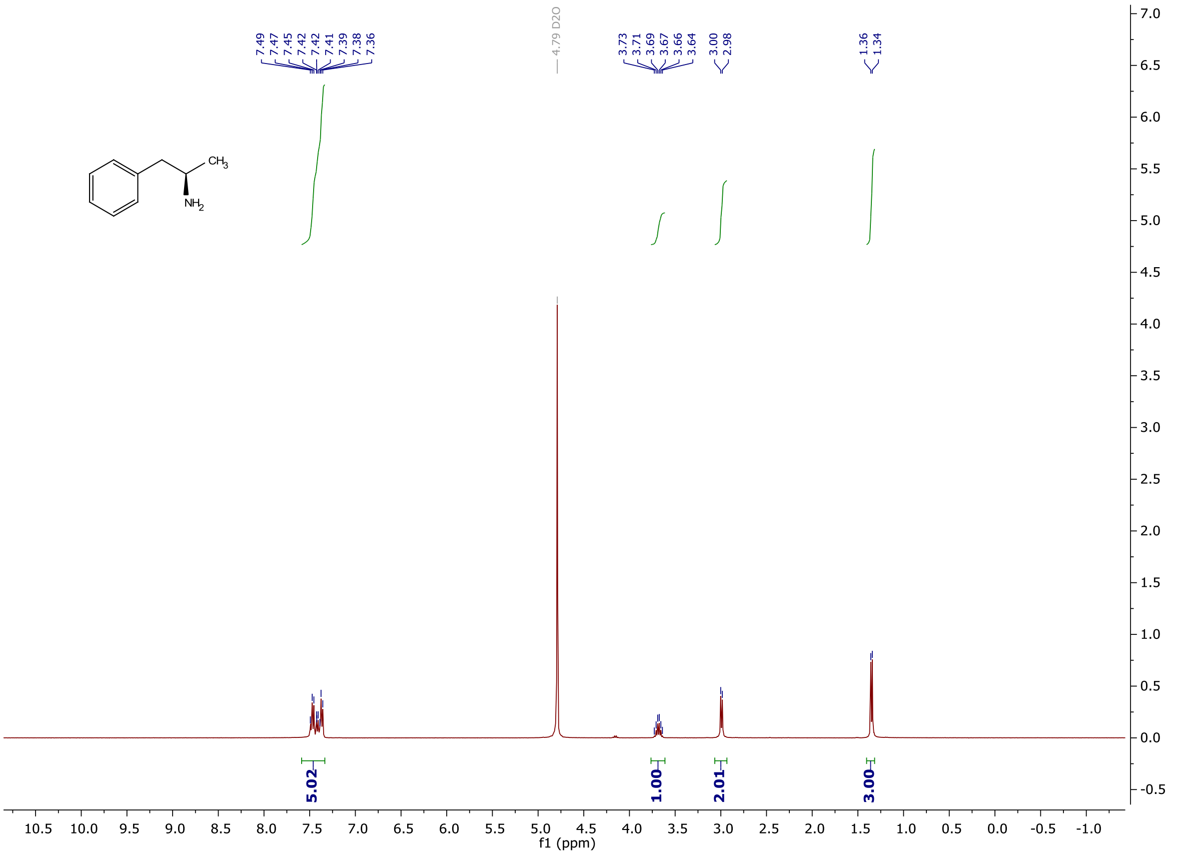

^{13}C{^{1}H} NMR (101 MHz, D_2O + DMSO-d_6 *(20:1)*)

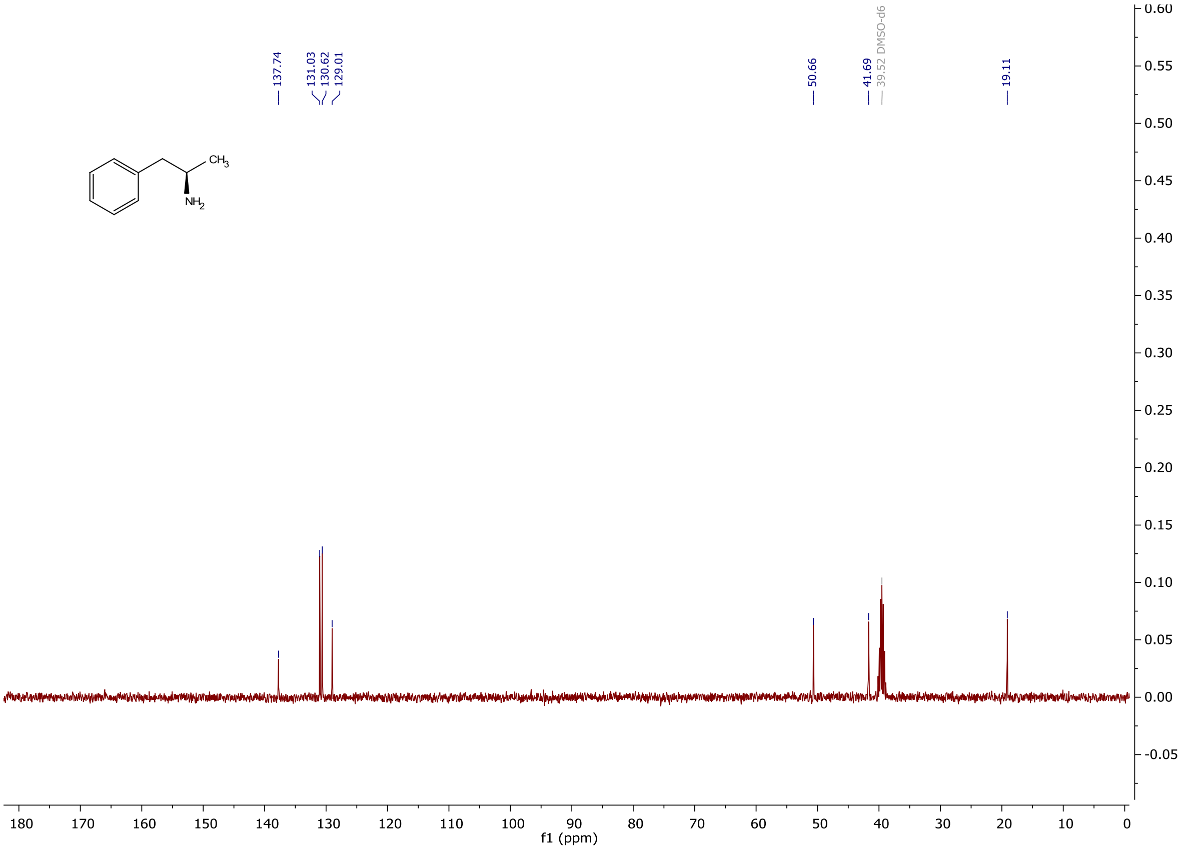

tert-Butyl ((1*S*,2*S*)-2-amino-1,2-diphenylethyl)carbamate (**58**)

^{1}H NMR (400 MHz, DMSO-d_6)

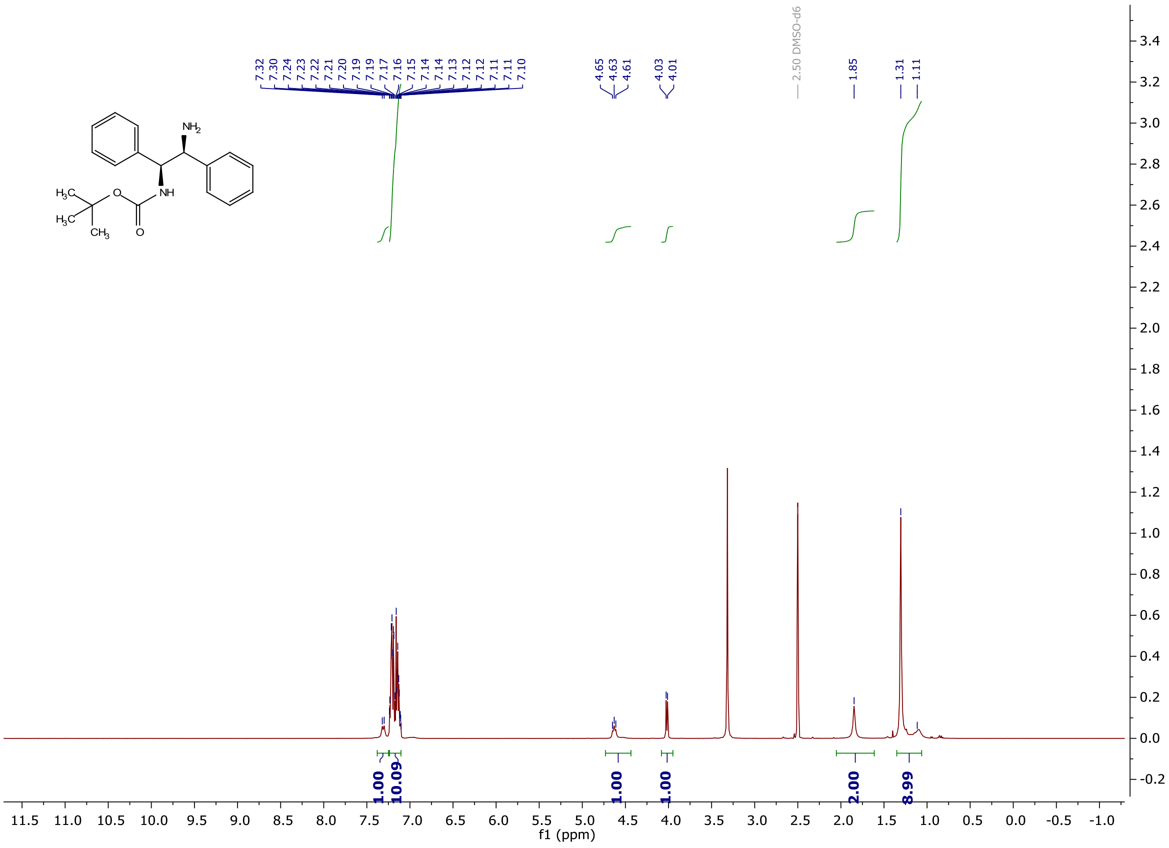

^{13}C{^{1}H} NMR (101 MHz, DMSO-d_6)

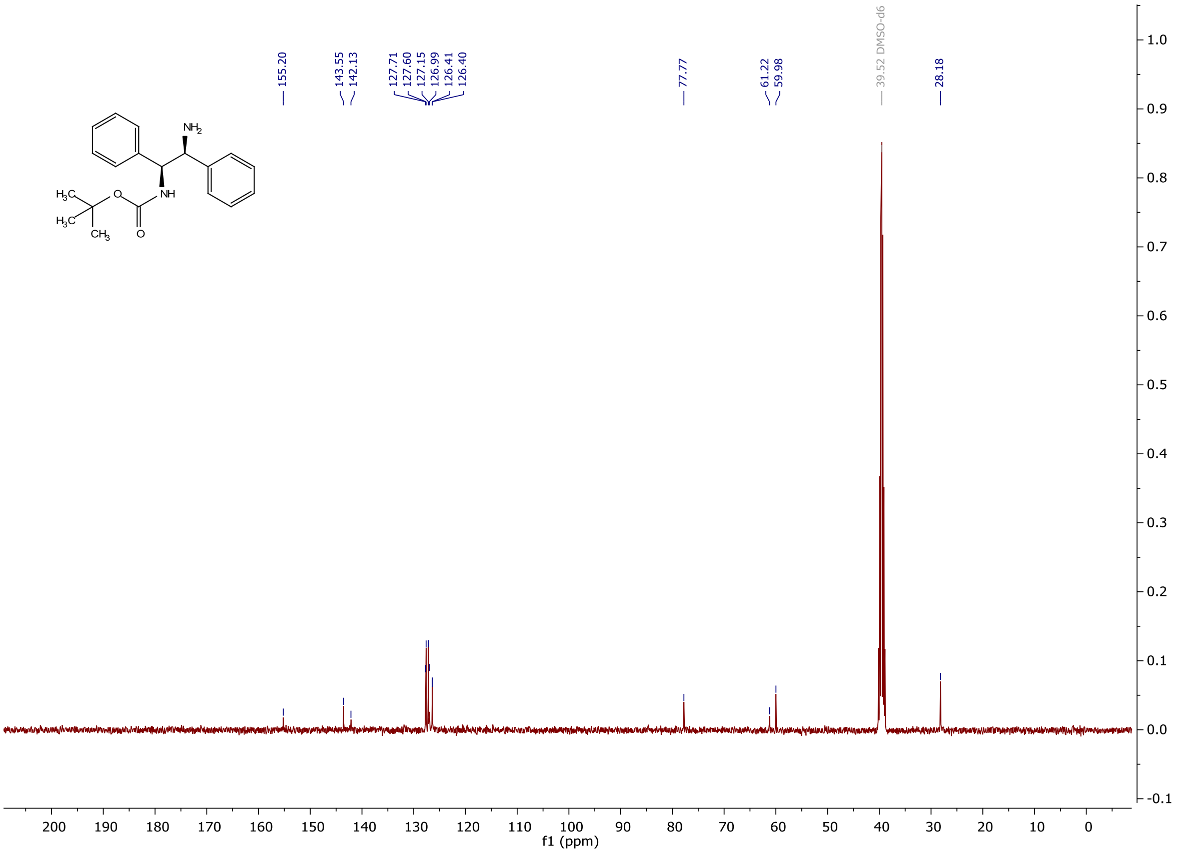

(1*S*,2*S*)-*N*[1],*N*[1]-Dimethyl-1,2-diphenylethane-1,2-diamine (**59**)

^{1}H NMR (400 MHz, $CDCl_3$)

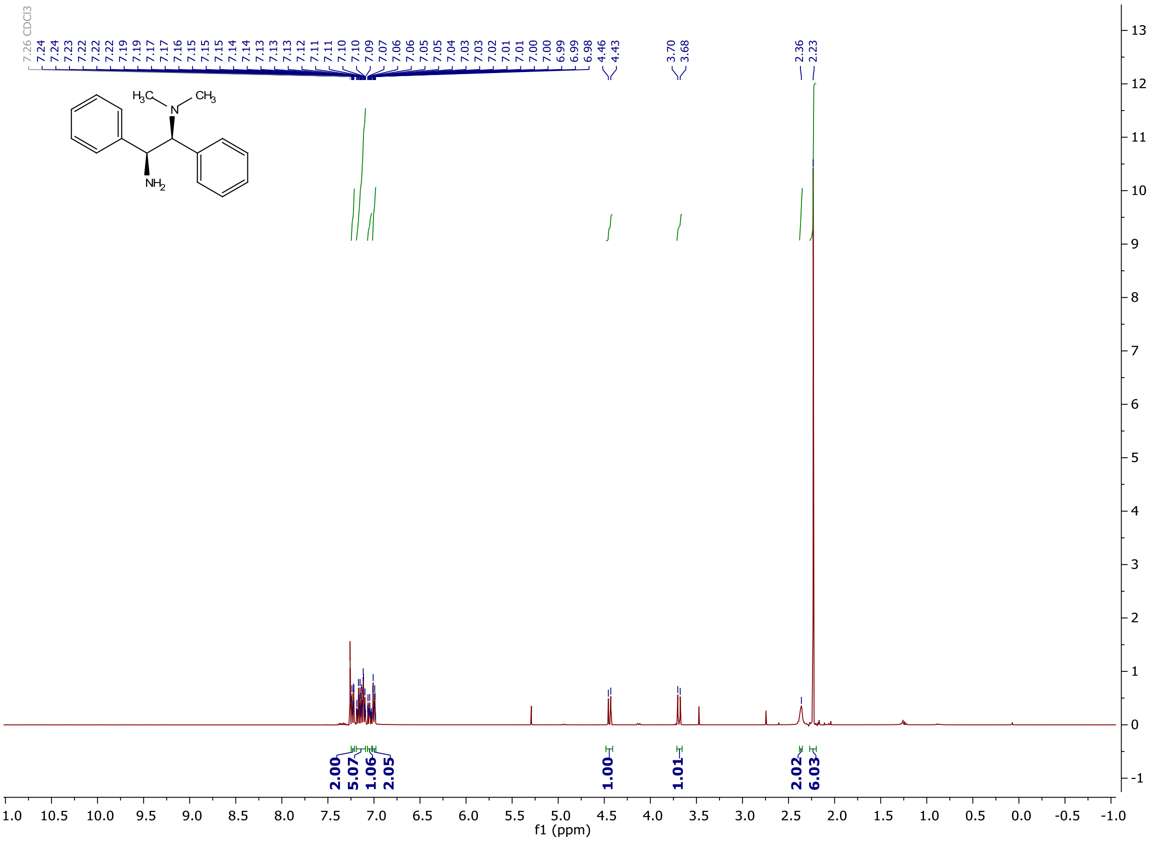

^{13}C{^{1}H} NMR (101 MHz, $CDCl_3$)

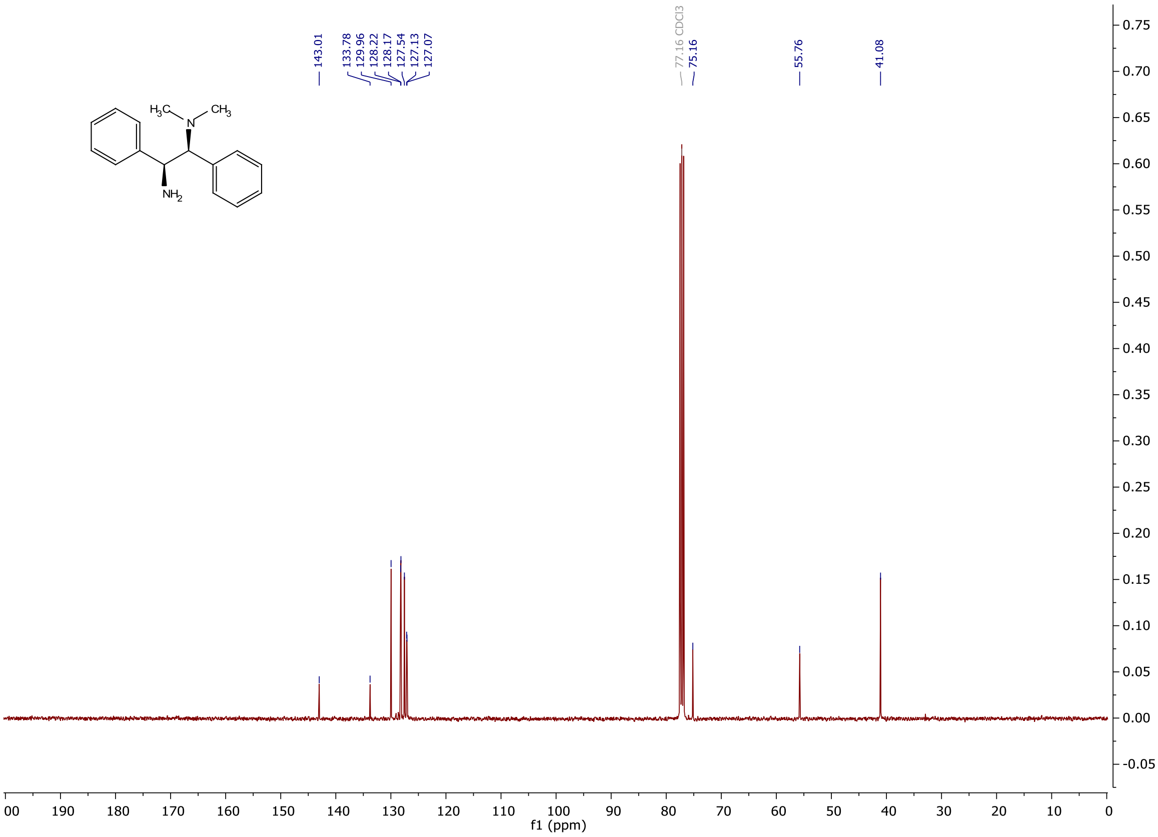

(9*H*-Fluoren-9-yl)methyl (*S*)-(1-(diethylamino)-1-oxopropan-2-yl)carbamate (**60**)

^{1}H NMR (400 MHz, DMSO-*d*$_6$)

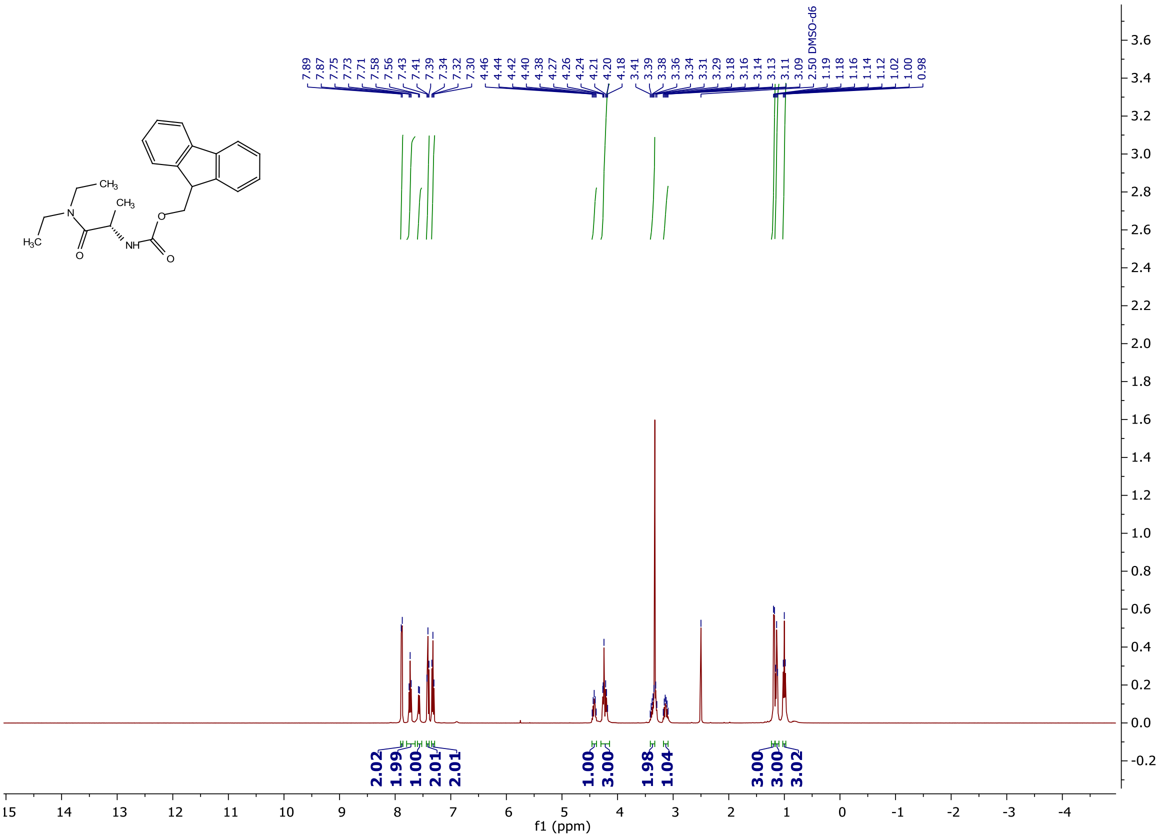

^{13}C{^{1}H} NMR (101 MHz, DMSO-*d*$_6$)

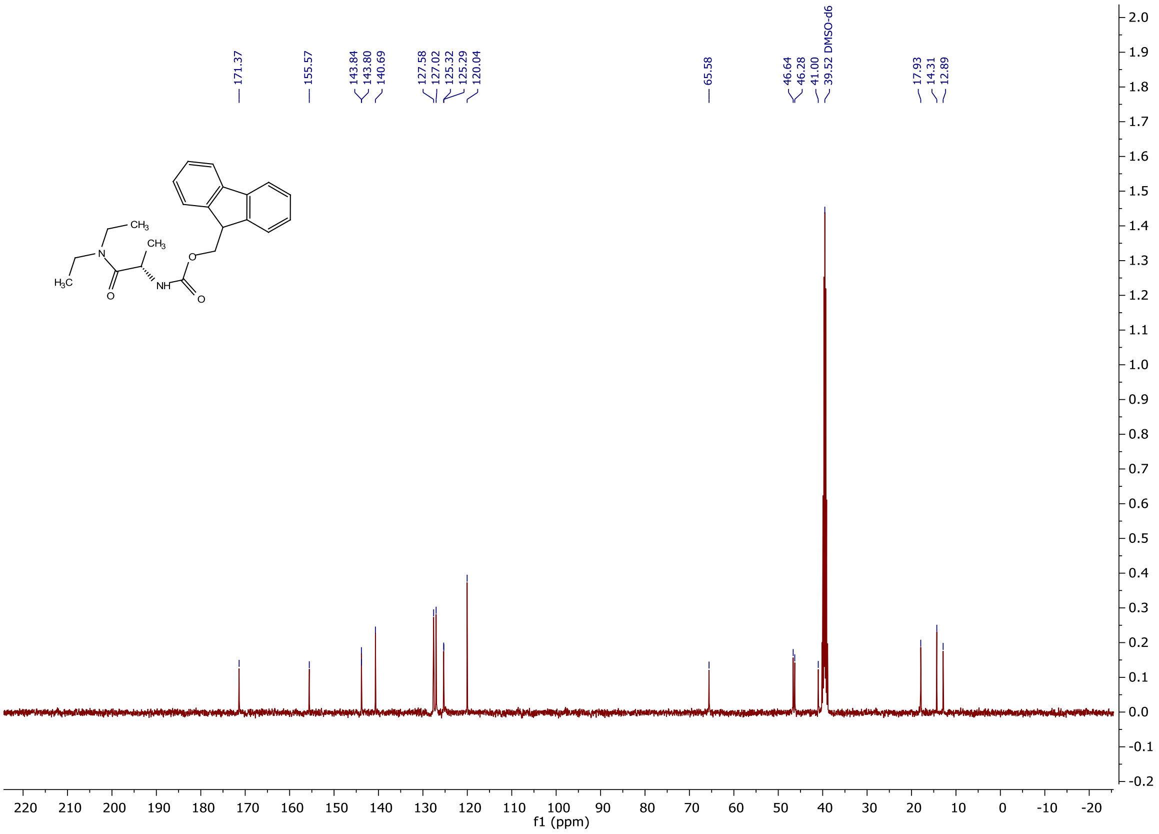

(4*R*,5*R*)-4-(4-Aminophenyl)-2,2-dimethyl-1,3-dioxan-5-amine (**61**)

^{1}H NMR (400 MHz, $CDCl_3$)

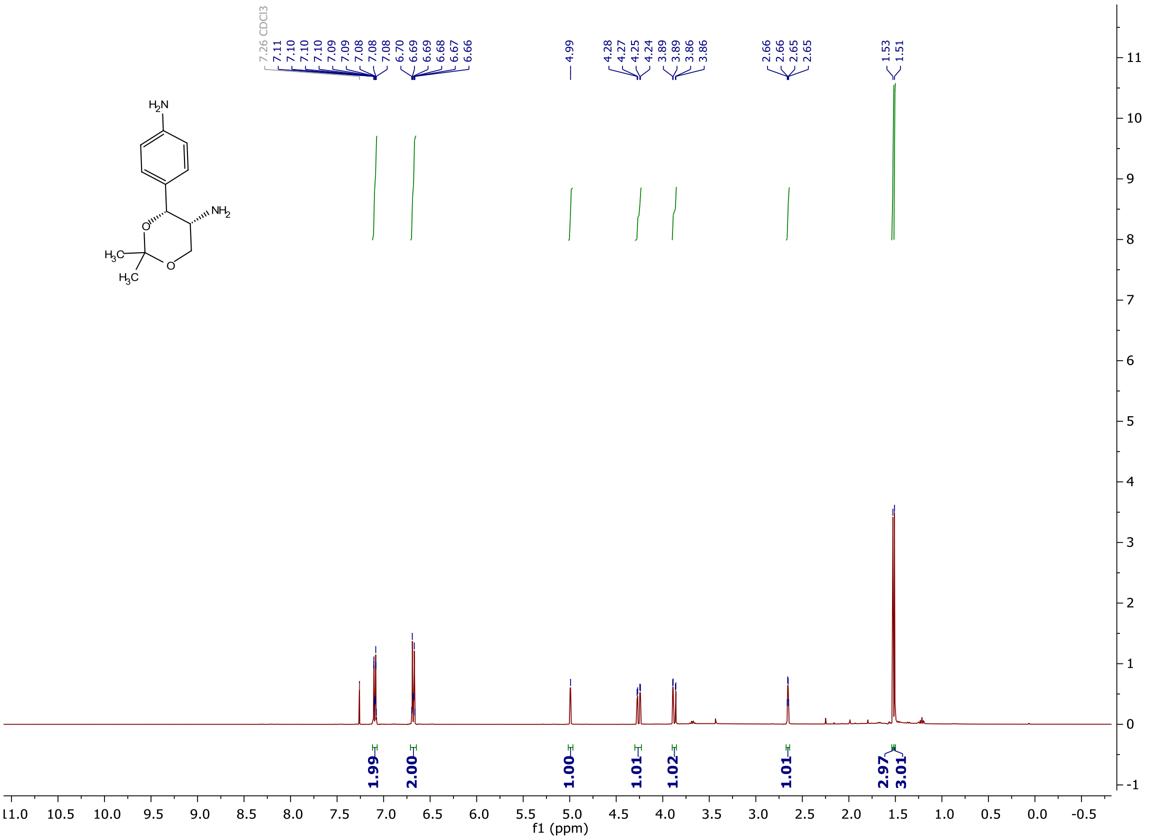

^{13}C{^{1}H} NMR (101 MHz, $CDCl_3$)

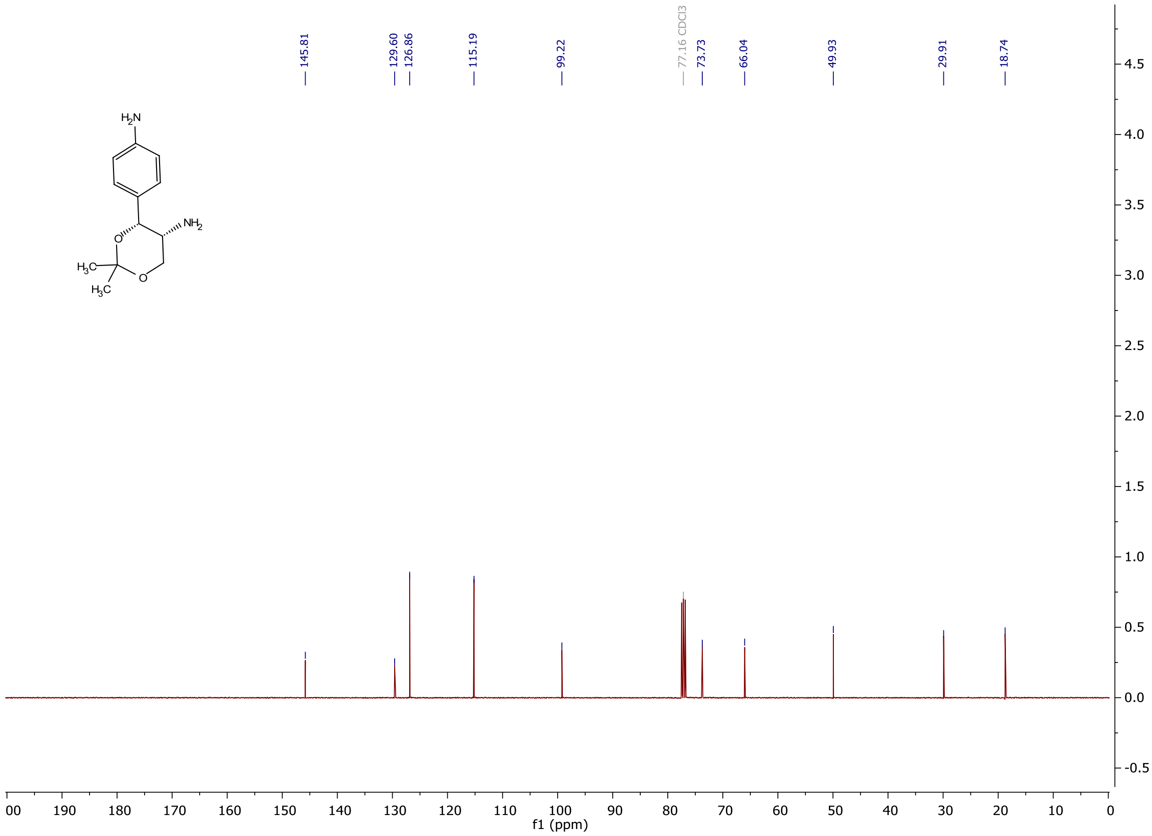

(4*R*,5*R*)-2,2-Dimethyl-4-(4-nitrophenyl)-2,2-dimethyl-1,3-dioxan-5-amine (**62**)

^{1}H NMR (400 MHz, $CDCl_3$)

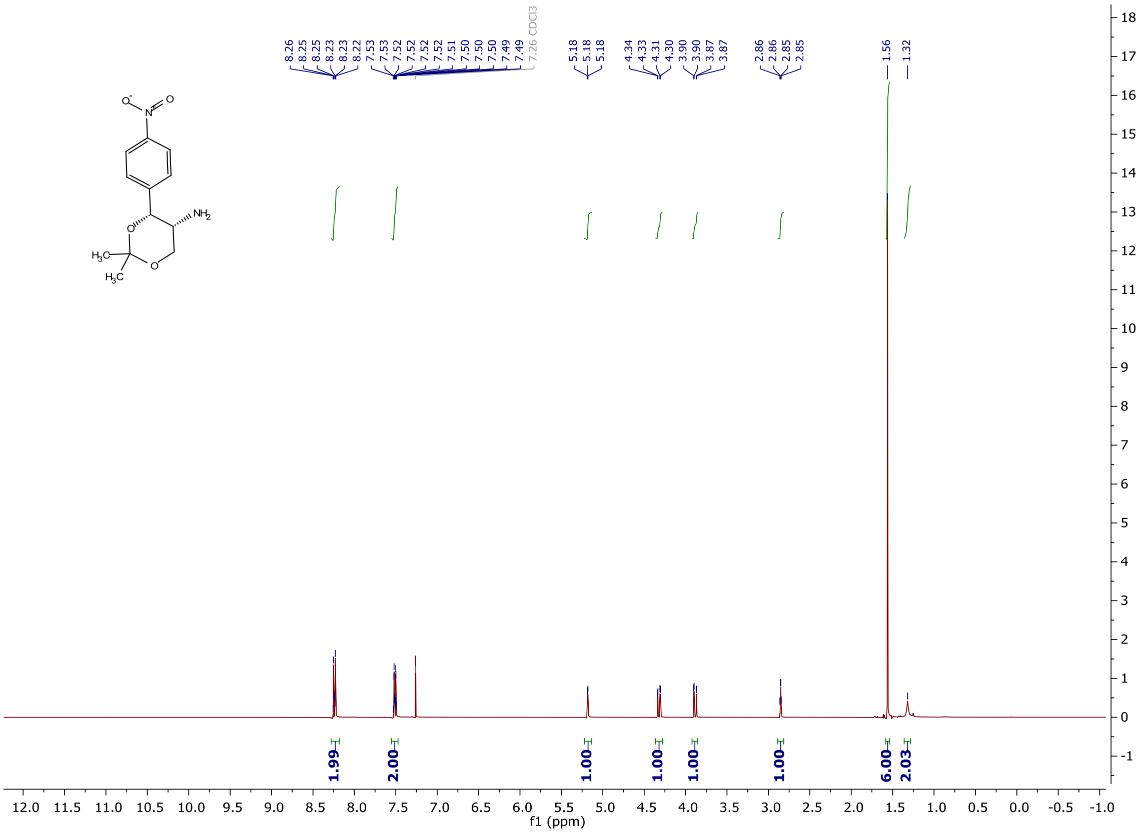

^{13}C{^{1}H} NMR (101 MHz, $CDCl_3$)

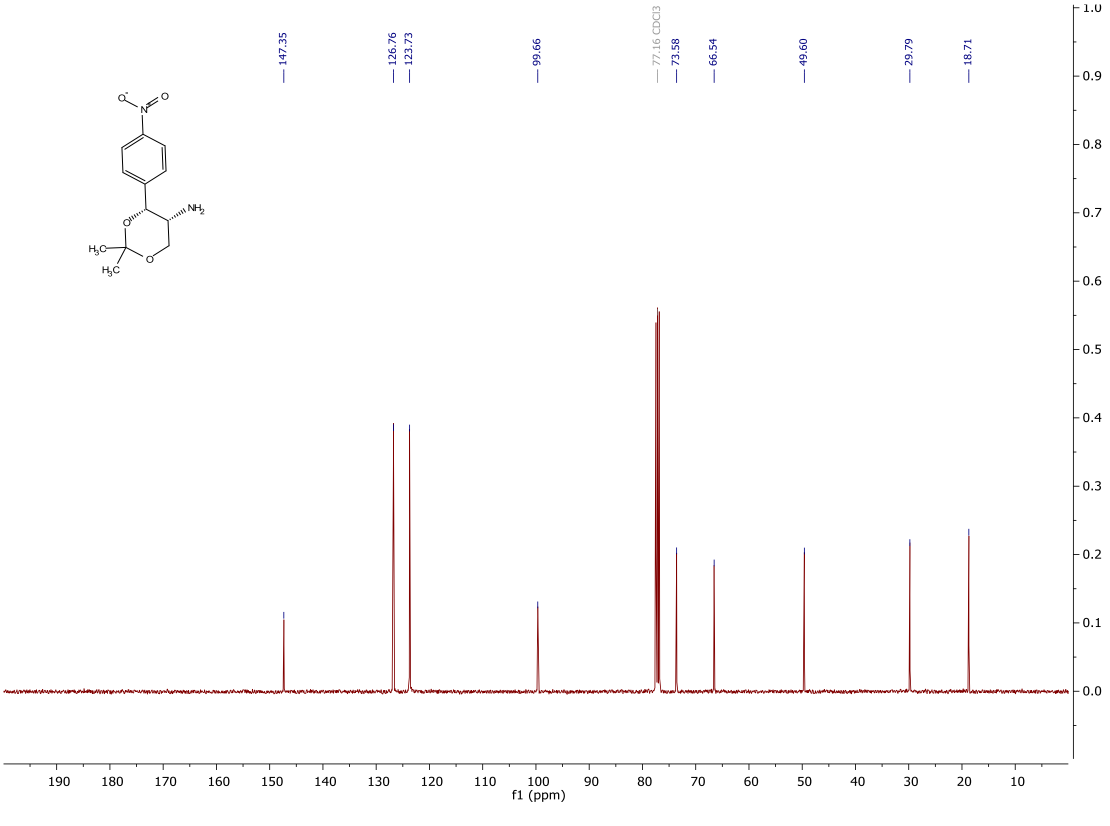

tert-Butyl ((1*R*,2*R*)-2-aminocyclohexyl)carbamate (**63**)

^{1}H NMR (400 MHz, DMSO-d_6)

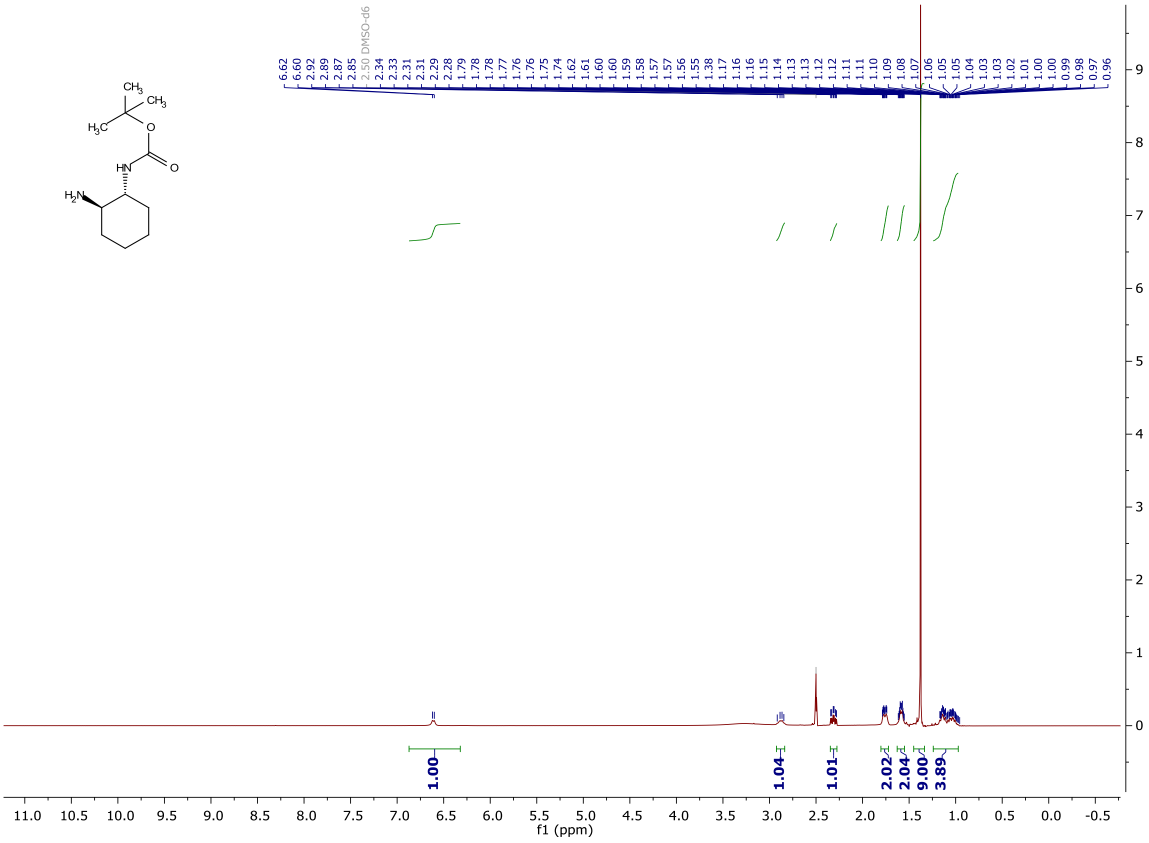

^{13}C{^{1}H} NMR (101 MHz, DMSO-d_6)

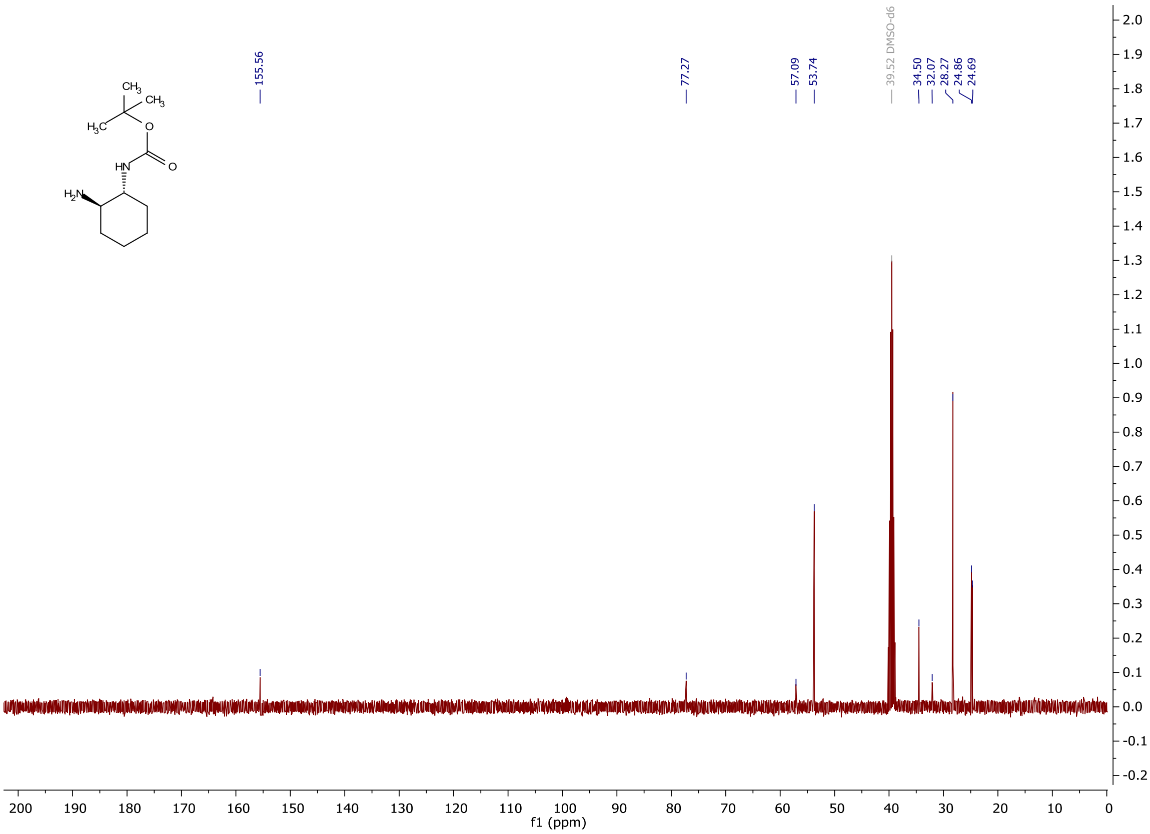

Conformational sampling and DFT calculations

Methods

Conformations of compounds 48-51 were sampled using Spartan'24 software,[10] employing the ML corrected MMFF force field. The ten lowest-energy conformers for each molecule were reoptimized using the def2-TZVP basis set[11] and the B97 DFT functional with the D3 empirical dispersion term and BJ-damping,[12,13] all performed with the TurboMole 7.2 program.[14,15] Solvation effects were modeled with the conductor-like screening model COSMO,[16] using e = 4.81 (chloroform).

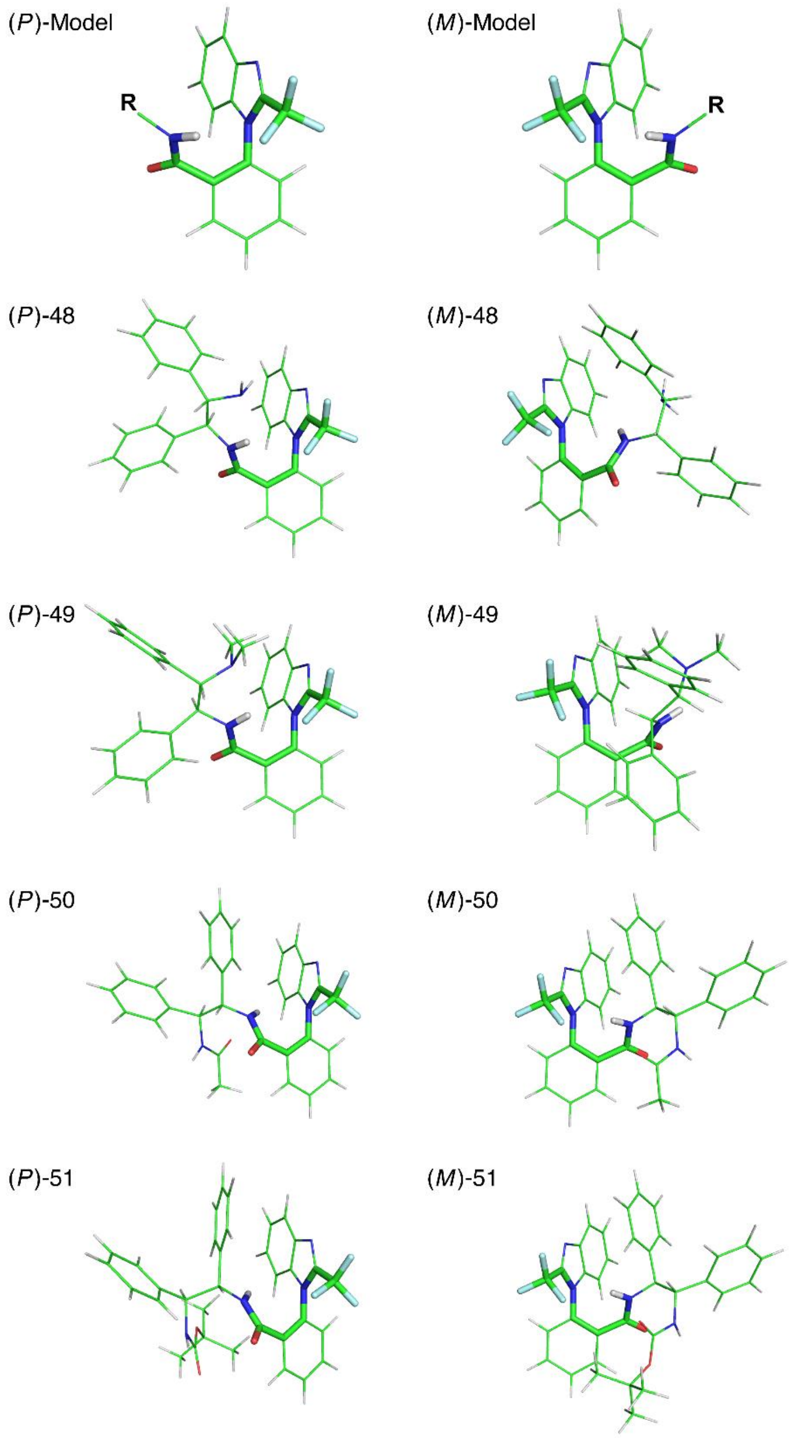

Figure S1. Idealized model geometry assumed in this work (*P* and *M* model) is compared with the minimum energy structures of compounds **48**-**51**. In some atropisomers, the conformation deviates from the model, which presumes that the TBBA amidic hydrogens are oriented toward the trifluoromethyl group, with the carbonyl oxygen pointing in the opposite direction. However, in the lowest energy conformers of **48**, **50**, and **51**, the carbonyl oxygen instead orients toward the trifluoromethyl group in some atropoisomers: **(*M*)-48** (but not in **(*P*)-48**), **(*P*)-50** (but not in **(*M*)-50**), and **(*P*)-51** (but not in **(*M*)-51**). In **(*M*)-49** (but not in **(*P*)-49**), both CO and NH point away from the trifluoromethyl group, again deviating from the model.

Geometries of the lowest-energy conformers, in XZY format. Line 1 indicates the number of atoms, and line 2 contains a comment specifying the compound number and the atropisomer description (*M* or *P*).

60
48 *M*

O	1.5369	1.4867	-2.0368
C	0.9472	1.3006	-0.9700
N	1.1691	0.2086	-0.1998
C	2.2876	-0.6788	-0.4745
C	2.0907	-2.0007	0.3233
N	1.8746	-1.6588	1.7312
C	0.9499	-2.8148	-0.2535
C	1.1509	-3.4899	-1.4658
C	0.1231	-4.2254	-2.0522
C	-1.1257	-4.3018	-1.4281
C	-1.3312	-3.6389	-0.2193
C	-0.2997	-2.8994	0.3637
C	3.6348	-0.0495	-0.1476
C	3.7726	0.9716	0.7972
C	5.0332	1.4857	1.1100
C	6.1727	0.9836	0.4798
C	6.0431	-0.0345	-0.4688
C	4.7828	-0.5451	-0.7778
C	0.0167	2.3589	-0.4307
C	-1.2039	2.1499	0.2262
N	-1.7465	0.8393	0.4565
C	-2.6187	0.1135	-0.3454
C	-2.8572	0.4591	-1.7923
F	-1.6998	0.4443	-2.5041
F	-3.7062	-0.4088	-2.3658
F	-3.3854	1.7075	-1.9294
N	-3.1854	-0.8961	0.2715
C	-2.6882	-0.8599	1.5680
C	-1.7889	0.2272	1.7066
C	-1.1217	0.5093	2.8979
C	-1.3746	-0.3494	3.9654
C	-2.2616	-1.4403	3.8451
C	-2.9303	-1.7096	2.6542
C	-1.9640	3.2278	0.6895
C	-1.5172	4.5324	0.5081
C	-0.3099	4.7595	-0.1563
C	0.4367	3.6849	-0.6269
H	0.7542	0.1120	0.7197
H	2.2753	-0.9228	-1.5394
H	3.0161	-2.5762	0.1636
H	1.6538	-2.4927	2.2679
H	2.7299	-1.2708	2.1214
H	2.1231	-3.4400	-1.9529
H	0.2960	-4.7416	-2.9933

H	-1.9306	-4.8734	-1.8832
H	-2.3013	-3.6806	0.2664
H	-0.4735	-2.3765	1.2975
H	2.8918	1.3787	1.2854
H	5.1226	2.2826	1.8442
H	7.1533	1.3869	0.7188
H	6.9231	-0.4247	-0.9739
H	4.6866	-1.3304	-1.5251
H	-0.4411	1.3502	2.9902
H	-0.8738	-0.1761	4.9137
H	-2.4244	-2.0834	4.7055
H	-3.6108	-2.5507	2.5605
H	-2.9113	3.0264	1.1805
H	-2.1133	5.3642	0.8721
H	0.0471	5.7737	-0.3116
H	1.3695	3.8514	-1.1554

60
48 *P*

O	0.0753	2.5679	-1.3488
C	-0.5075	1.9312	-0.4690
N	0.1145	1.1062	0.4018
C	1.4564	0.5862	0.1660
C	1.7543	-0.4809	1.2654
N	0.5459	-1.2684	1.5453
C	2.9795	-1.2858	0.8748
C	4.1798	-1.1408	1.5758
C	5.3171	-1.8598	1.2017
C	5.2650	-2.7346	0.1157
C	4.0693	-2.8860	-0.5926
C	2.9372	-2.1656	-0.2152
C	2.5382	1.6447	0.1736
C	2.5340	2.6664	1.1280
C	3.5758	3.5924	1.1778
C	4.6399	3.5007	0.2765
C	4.6502	2.4804	-0.6766
C	3.6027	1.5598	-0.7267
C	-2.0048	2.0467	-0.3203
C	-2.8708	0.9415	-0.3768
N	-2.3500	-0.3867	-0.5225
C	-2.4847	-1.4754	0.3290
C	-3.1888	-1.4084	1.6612
F	-4.5449	-1.4492	1.5267
F	-2.8355	-2.4417	2.4442
F	-2.8946	-0.2613	2.3271
N	-1.8972	-2.5645	-0.1122
C	-1.3157	-2.2044	-1.3204
C	-1.5898	-0.8417	-1.6003
C	-1.1259	-0.2050	-2.7518
C	-0.3433	-0.9724	-3.6110
C	-0.0446	-2.3247	-3.3387
C	-0.5285	-2.9603	-2.2000

C	-4.2556	1.1193	-0.3518
C	-4.7902	2.3986	-0.2211
C	-3.9411	3.5054	-0.1543
C	-2.5621	3.3273	-0.2263
H	-0.4336	0.5198	1.0266
H	1.4620	0.0906	-0.8146
H	1.9809	0.0698	2.1847
H	0.3684	-1.9515	0.8129
H	0.6711	-1.7952	2.4050
H	4.2272	-0.4484	2.4134
H	6.2436	-1.7332	1.7563
H	6.1487	-3.2947	-0.1790
H	4.0214	-3.5631	-1.4420
H	2.0171	-2.2877	-0.7837
H	1.7038	2.7432	1.8258
H	3.5572	4.3875	1.9194
H	5.4521	4.2222	0.3145
H	5.4725	2.4021	-1.3835
H	3.6136	0.7650	-1.4685
H	-1.3388	0.8371	-2.9602
H	0.0515	-0.5140	-4.5131
H	0.5755	-2.8788	-4.0378
H	-0.3072	-4.0023	-1.9883
H	-4.9046	0.2552	-0.4440
H	-5.8679	2.5297	-0.1905
H	-4.3537	4.5058	-0.0606
H	-1.8938	4.1832	-0.2069

66

49 *M*

O	-2.9326	1.6488	1.5383
C	-1.9179	0.9958	1.2735
N	-1.9026	0.0378	0.3236
C	-0.9098	-1.0128	0.1318
C	-1.4520	-1.9650	-0.9739
N	-2.1238	-1.2194	-2.0515
C	-0.3893	-2.9537	-1.4217
C	-0.6754	-4.3238	-1.4046
C	0.2664	-5.2614	-1.8302
C	1.5161	-4.8380	-2.2864
C	1.8131	-3.4737	-2.3127
C	0.8680	-2.5420	-1.8836
C	-0.6514	-1.8146	1.3974
C	-1.7084	-2.1451	2.2531
C	-1.4807	-2.8966	3.4048
C	-0.1902	-3.3348	3.7125
C	0.8651	-3.0223	2.8546
C	0.6331	-2.2685	1.7033
C	-0.6425	1.2691	2.0352
C	0.4911	1.8827	1.4757
N	0.5385	2.2161	0.0818
C	1.4599	1.8206	-0.8856

C	2.7007	1.0044	-0.6240
F	2.5168	0.0505	0.3222
F	3.1143	0.3878	-1.7483
F	3.7306	1.7882	-0.1958
N	1.2164	2.3202	-2.0769
C	0.0844	3.1057	-1.9258
C	-0.3499	3.0705	-0.5769
C	-1.4305	3.8270	-0.1210
C	-2.0945	4.5944	-1.0736
C	-1.6952	4.6144	-2.4277
C	-0.6028	3.8786	-2.8715
C	1.5838	2.2185	2.2794
C	1.5825	1.8962	3.6330
C	0.4690	1.2710	4.1973
C	-0.6388	0.9849	3.4062
H	-2.7261	-0.0415	-0.2688
H	0.0342	-0.5733	-0.2020
H	-2.2523	-2.5452	-0.4989
H	-1.6446	-4.6589	-1.0415
H	0.0264	-6.3214	-1.8008
H	2.2540	-5.5647	-2.6161
H	2.7842	-3.1331	-2.6630
H	1.1252	-1.4891	-1.9046
H	-2.7125	-1.7950	2.0289
H	-2.3106	-3.1369	4.0649
H	-0.0100	-3.9153	4.6137
H	1.8728	-3.3608	3.0822
H	1.4574	-2.0352	1.0389
H	-1.7509	3.8080	0.9134
H	-2.9447	5.1956	-0.7636
H	-2.2500	5.2279	-3.1323
H	-0.2777	3.9023	-3.9074
H	2.4286	2.7358	1.8378
H	2.4429	2.1479	4.2461
H	0.4585	1.0191	5.2538
H	-1.5149	0.5151	3.8412
C	-1.2740	-0.3471	-2.8555
H	-1.9133	0.2874	-3.4777
H	-0.5868	-0.8976	-3.5201
H	-0.6831	0.3070	-2.2120
C	-2.9520	-2.0828	-2.8876
H	-3.5490	-1.4593	-3.5612
H	-3.6313	-2.6657	-2.2571
H	-2.3606	-2.7852	-3.5019

66
49 *P*

O	0.0852	2.4407	-1.6850
C	-0.4191	1.8109	-0.7528
N	0.2364	0.8527	-0.0625
C	1.6102	0.4620	-0.3509

C	1.9845	-0.6479	0.6662
N	0.9127	-1.6586	0.7632
C	3.3781	-1.2033	0.4332
C	4.2546	-1.3226	1.5184
C	5.5304	-1.8652	1.3590
C	5.9502	-2.3015	0.1013
C	5.0863	-2.1880	-0.9900
C	3.8125	-1.6441	-0.8243
C	2.5827	1.6198	-0.2179
C	2.5128	2.4824	0.8805
C	3.4449	3.5077	1.0377
C	4.4641	3.6787	0.0973
C	4.5373	2.8219	-1.0027
C	3.5990	1.8010	-1.1579
C	-1.8040	2.1572	-0.2622
C	-2.8302	1.2309	-0.0262
N	-2.6319	-0.1629	-0.2920
C	-2.7397	-1.2456	0.5713
C	-2.8792	-1.0950	2.0643
F	-4.1430	-0.7484	2.4348
F	-2.5800	-2.2414	2.6974
F	-2.0516	-0.1247	2.5433
N	-2.6647	-2.4092	-0.0332
C	-2.4984	-2.1133	-1.3799
C	-2.4672	-0.7088	-1.5644
C	-2.2941	-0.1153	-2.8147
C	-2.1388	-0.9833	-3.8929
C	-2.1621	-2.3853	-3.7280
C	-2.3427	-2.9685	-2.4782
C	-4.0955	1.6541	0.3876
C	-4.3381	3.0063	0.6138
C	-3.3253	3.9404	0.3869
C	-2.0785	3.5172	-0.0650
H	-0.2533	0.2398	0.5804
H	1.6670	0.0791	-1.3762
H	1.9958	-0.1564	1.6465
H	3.9353	-0.9755	2.4987
H	6.1967	-1.9423	2.2147
H	6.9439	-2.7224	-0.0291
H	5.4061	-2.5197	-1.9747
H	3.1629	-1.5533	-1.6891
H	1.7165	2.3595	1.6108
H	3.3757	4.1744	1.8939
H	5.1916	4.4773	0.2186
H	5.3226	2.9510	-1.7434
H	3.6564	1.1392	-2.0180
H	-2.2669	0.9616	-2.9426
H	-1.9937	-0.5691	-4.8867
H	-2.0342	-3.0201	-4.6005
H	-2.3574	-4.0465	-2.3475
H	-4.8843	0.9208	0.5162
H	-5.3206	3.3285	0.9463

H	-3.5100	4.9981	0.5514
H	-1.2912	4.2369	-0.2667
C	0.7666	-2.5253	-0.4030
H	-0.1242	-3.1411	-0.2637
H	0.6234	-1.9277	-1.3072
H	1.6313	-3.1929	-0.5553
C	0.9989	-2.4414	1.9937
H	0.1088	-3.0722	2.0742
H	1.0289	-1.7688	2.8570
H	1.8895	-3.0937	2.0289

65
50 *M*

O	-0.7384	2.9041	1.9091
C	-1.2951	3.3302	0.8934
C	-1.0221	4.7217	0.3577
N	-2.1940	2.5947	0.1823
C	-2.4614	1.1957	0.4783
C	-3.9087	0.8240	0.2491
C	-4.6486	0.2222	1.2710
C	-5.9710	-0.1691	1.0559
C	-6.5690	0.0410	-0.1878
C	-5.8365	0.6405	-1.2154
C	-4.5131	1.0239	-0.9985
C	-1.5290	0.2622	-0.3595
C	-1.6173	-1.1721	0.1121
C	-1.2023	-1.5282	1.4002
C	-1.2856	-2.8507	1.8305
C	-1.7977	-3.8315	0.9789
C	-2.2183	-3.4829	-0.3051
C	-2.1248	-2.1599	-0.7355
N	-0.1562	0.7388	-0.3341
C	0.4276	1.3714	-1.3777
C	1.8802	1.7202	-1.1925
C	2.8449	0.8224	-0.7022
N	2.4828	-0.5175	-0.3497
C	2.5334	-1.1435	0.8915
C	2.9276	-0.4270	2.1561
F	2.6089	-1.1494	3.2418
F	4.2612	-0.1642	2.2181
F	2.2868	0.7749	2.2543
N	2.1346	-2.3938	0.8650
C	1.7839	-2.6404	-0.4551
C	1.9915	-1.4748	-1.2350
C	1.7092	-1.4219	-2.5997
C	1.1979	-2.5856	-3.1692
C	0.9834	-3.7543	-2.4079
C	1.2726	-3.8005	-1.0492
C	4.1864	1.1985	-0.6162
C	4.5737	2.4872	-0.9766
C	3.6254	3.3899	-1.4615
C	2.2952	2.9981	-1.5852

O	-0.1610	1.6577	-2.4241
H	0.0439	4.7971	0.1195
H	-1.6064	4.9673	-0.5330
H	-1.2367	5.4518	1.1439
H	-2.5386	2.9724	-0.6878
H	-2.2148	1.0619	1.5334
H	-4.1821	0.0524	2.2382
H	-6.5330	-0.6380	1.8594
H	-7.5989	-0.2621	-0.3574
H	-6.2938	0.8042	-2.1878
H	-3.9512	1.4727	-1.8152
H	-1.8402	0.3214	-1.4042
H	-0.8056	-0.7749	2.0779
H	-0.9443	-3.1163	2.8272
H	-1.8599	-4.8642	1.3126
H	-2.6081	-4.2433	-0.9769
H	-2.4440	-1.8899	-1.7390
H	0.3499	0.6819	0.5368
H	1.8631	-0.5219	-3.1858
H	0.9541	-2.5910	-4.2280
H	0.5739	-4.6336	-2.8973
H	1.0967	-4.6929	-0.4571
H	4.9191	0.4743	-0.2774
H	5.6173	2.7768	-0.8957
H	3.9238	4.3927	-1.7531
H	1.5533	3.6817	-1.9867

65
50 *P*

O	-0.8802	2.3098	-2.1231
C	-1.3530	1.4168	-2.8340
C	-1.0459	1.3306	-4.3150
N	-2.1830	0.4543	-2.3487
C	-2.5365	0.3562	-0.9403
C	-3.9670	-0.0906	-0.7442
C	-4.8433	0.6801	0.0251
C	-6.1531	0.2541	0.2510
C	-6.6011	-0.9499	-0.2951
C	-5.7313	-1.7271	-1.0641
C	-4.4210	-1.3017	-1.2816
C	-1.5581	-0.5963	-0.1704
C	-1.7949	-0.4912	1.3195
C	-1.4566	0.6719	2.0207
C	-1.7236	0.7776	3.3847
C	-2.3405	-0.2760	4.0627
C	-2.6844	-1.4376	3.3688
C	-2.4115	-1.5422	2.0044
N	-0.1831	-0.2986	-0.5364
C	0.4957	-1.0342	-1.4541
C	1.8065	-0.4552	-1.9245
C	2.7460	0.2062	-1.1161

N	2.5383	0.3808	0.2930
C	2.8185	-0.4881	1.3364
C	3.4049	-1.8578	1.1098
F	3.6272	-2.4900	2.2730
F	2.5808	-2.6393	0.3622
F	4.5896	-1.7879	0.4413
N	2.5334	0.0029	2.5206
C	2.0291	1.2738	2.2781
C	2.0278	1.5324	0.8835
C	1.5318	2.7141	0.3314
C	1.0397	3.6549	1.2346
C	1.0507	3.4244	2.6268
C	1.5416	2.2395	3.1668
C	3.9243	0.7206	-1.6593
C	4.1851	0.5837	-3.0201
C	3.2713	-0.0888	-3.8340
C	2.1037	-0.6105	-3.2858
O	0.0769	-2.0957	-1.9212
H	-1.5021	0.4659	-4.8041
H	-1.3987	2.2460	-4.8007
H	0.0400	1.2851	-4.4417
H	-2.4848	-0.2804	-2.9710
H	-2.4089	1.3600	-0.5283
H	-4.4933	1.6141	0.4575
H	-6.8224	0.8623	0.8540
H	-7.6208	-1.2832	-0.1210
H	-6.0712	-2.6682	-1.4887
H	-3.7497	-1.9290	-1.8649
H	-1.7422	-1.6203	-0.4990
H	-0.9789	1.5051	1.5115
H	-1.4455	1.6827	3.9172
H	-2.5493	-0.1928	5.1262
H	-3.1636	-2.2631	3.8890
H	-2.6842	-2.4450	1.4632
H	0.1336	0.6454	-0.3634
H	1.5075	2.8855	-0.7397
H	0.6329	4.5875	0.8544
H	0.6549	4.1890	3.2894
H	1.5335	2.0533	4.2365
H	4.6324	1.2154	-1.0016
H	5.1017	0.9894	-3.4381
H	3.4701	-0.2095	-4.8951
H	1.3936	-1.1467	-3.9069

75
51 *M*

O	-0.4785	1.8039	-2.5664
C	-0.7514	0.6589	-2.8999
O	-0.1014	-0.0809	-3.8302
C	0.9735	0.5116	-4.6702
C	2.1375	0.9881	-3.8010

C	1.4010	-0.6718	-5.5369
C	0.3909	1.6328	-5.5293
N	-1.7975	-0.0664	-2.3864
C	-2.3732	0.3257	-1.1089
C	-3.8566	0.0487	-1.0189
C	-4.7306	1.0537	-0.5939
C	-6.0955	0.7971	-0.4521
C	-6.6012	-0.4725	-0.7362
C	-5.7342	-1.4839	-1.1585
C	-4.3703	-1.2252	-1.2929
C	-1.6379	-0.3760	0.0860
C	-1.9271	0.3159	1.3976
C	-1.4995	1.6301	1.6205
C	-1.7634	2.2642	2.8327
C	-2.4739	1.5947	3.8311
C	-2.9108	0.2878	3.6124
C	-2.6337	-0.3486	2.4029
N	-0.2047	-0.4662	-0.1493
C	0.3885	-1.5981	-0.5962
C	1.8903	-1.5751	-0.6829
C	2.7398	-1.0021	0.2807
N	2.2052	-0.4123	1.4714
C	2.2752	0.9065	1.9121
C	2.8995	2.0114	1.1008
F	2.5710	3.2170	1.5908
F	4.2580	1.9389	1.0724
F	2.4772	1.9601	-0.1963
N	1.6856	1.1039	3.0681
C	1.1804	-0.1342	3.4413
C	1.4927	-1.1004	2.4523
C	1.0948	-2.4334	2.5490
C	0.3591	-2.7764	3.6808
C	0.0391	-1.8278	4.6754
C	0.4426	-0.5019	4.5731
C	4.1265	-1.0664	0.1333
C	4.6819	-1.6689	-0.9933
C	3.8510	-2.2467	-1.9547
C	2.4701	-2.2131	-1.7865
O	-0.2444	-2.6036	-0.9328
H	1.8577	1.8513	-3.1979
H	2.4739	0.1852	-3.1396
H	2.9731	1.2659	-4.4526
H	2.1936	-0.3562	-6.2222
H	1.7842	-1.4866	-4.9149
H	0.5577	-1.0452	-6.1261
H	0.0790	2.4794	-4.9152
H	1.1536	1.9762	-6.2362
H	-0.4683	1.2673	-6.1013
H	-1.7980	-1.0515	-2.6160
H	-2.2055	1.4005	-1.0286
H	-4.3373	2.0416	-0.3671
H	-6.7625	1.5889	-0.1207

H	-7.6637	-0.6747	-0.6285
H	-6.1200	-2.4759	-1.3793
H	-3.7065	-2.0270	-1.6088
H	-1.9909	-1.4080	0.1331
H	-0.9549	2.1688	0.8474
H	-1.4112	3.2786	2.9990
H	-2.6792	2.0882	4.7776
H	-3.4565	-0.2423	4.3887
H	-2.9641	-1.3708	2.2368
H	0.3610	0.3509	0.0192
H	1.3316	-3.1632	1.7818
H	0.0189	-3.8016	3.7975
H	-0.5459	-2.1433	5.5347
H	0.1861	0.2338	5.3285
H	4.7612	-0.6554	0.9106
H	5.7615	-1.7038	-1.1063
H	4.2784	-2.7272	-2.8299
H	1.8110	-2.6705	-2.5170

75
51 *P*

O	-1.4739	-1.0186	-3.8204
C	-1.7836	-0.3791	-2.8231
O	-1.5437	0.9386	-2.6156
C	-0.8847	1.7802	-3.6434
C	-1.7532	1.8469	-4.8983
C	-0.8232	3.1424	-2.9532
C	0.5212	1.2569	-3.9364
N	-2.4631	-0.9286	-1.7698
C	-2.6366	-0.3060	-0.4648
C	-3.9170	-0.7508	0.2076
C	-4.8351	0.1989	0.6651
C	-5.9973	-0.1965	1.3293
C	-6.2537	-1.5520	1.5422
C	-5.3403	-2.5081	1.0908
C	-4.1771	-2.1086	0.4329
C	-1.4273	-0.5887	0.4939
C	-1.4541	0.3415	1.6851
C	-1.3988	1.7293	1.5068
C	-1.4305	2.5861	2.6050
C	-1.5179	2.0646	3.8969
C	-1.5696	0.6822	4.0831
C	-1.5386	-0.1732	2.9813
N	-0.1506	-0.4938	-0.1965
C	0.4648	-1.5654	-0.7556
C	1.7912	-1.2856	-1.4110
C	2.8064	-0.4887	-0.8562
N	2.6479	0.1442	0.4223
C	2.7321	-0.3902	1.7007
C	3.0641	-1.8373	1.9625
F	3.3675	-2.0369	3.2564

F	2.0243	-2.6568	1.6537
F	4.1239	-2.2478	1.2163
N	2.4998	0.4847	2.6517
C	2.2405	1.6807	1.9969
C	2.3347	1.4893	0.5948
C	2.1142	2.5163	-0.3238
C	1.7947	3.7643	0.2088
C	1.7069	3.9747	1.6004
C	1.9274	2.9449	2.5093
C	4.0004	-0.2682	-1.5445
C	4.1962	-0.8408	-2.7988
C	3.2035	-1.6512	-3.3543
C	2.0199	-1.8789	-2.6595
O	-0.0219	-2.6972	-0.7660
H	-1.8040	0.8767	-5.3946
H	-2.7664	2.1728	-4.6414
H	-1.3234	2.5762	-5.5933
H	-0.3423	3.8696	-3.6144
H	-1.8298	3.4992	-2.7139
H	-0.2451	3.0808	-2.0263
H	0.4896	0.3007	-4.4578
H	1.0519	1.9855	-4.5582
H	1.0841	1.1256	-3.0082
H	-2.5046	-1.9373	-1.8140
H	-2.6888	0.7676	-0.6381
H	-4.6335	1.2556	0.5065
H	-6.7010	0.5539	1.6806
H	-7.1582	-1.8629	2.0586
H	-5.5309	-3.5654	1.2558
H	-3.4687	-2.8652	0.1020
H	-1.5067	-1.6233	0.8337
H	-1.3276	2.1522	0.5071
H	-1.3769	3.6600	2.4519
H	-1.5399	2.7324	4.7542
H	-1.6331	0.2687	5.0863
H	-1.5802	-1.2498	3.1268
H	0.2624	0.4215	-0.2962
H	2.2060	2.3595	-1.3933
H	1.6153	4.5963	-0.4662
H	1.4534	4.9652	1.9673
H	1.8464	3.1029	3.5800
H	4.7695	0.3430	-1.0822
H	5.1245	-0.6628	-3.3337
H	3.3514	-2.1038	-4.3306
H	1.2382	-2.4999	-3.0842
